# Ternary host-guest complexes with rapid exchange kinetics and photoswitchable fluorescence

**DOI:** 10.1016/j.chempr.2022.05.008

**Published:** 2022-09-08

**Authors:** Julius Gemen, Michał J. Białek, Miri Kazes, Linda J.W. Shimon, Moran Feller, Sergey N. Semenov, Yael Diskin-Posner, Dan Oron, Rafal Klajn

**Affiliations:** 1 Department of Molecular Chemistry & Materials Science, Weizmann Institute of Science, Rehovot 76100, Israel; 2 Department of Chemistry, University of Wrocław, 14 F. Joliot-Curie St., 50383 Wrocław, Poland; 3 Department of Chemical Research Support, Weizmann Institute of Science, Rehovot 76100, Israel

**Keywords:** supramolecular chemistry, coordination cages, host-guest chemistry, stimuli-responsive materials, BODIPY, anthracene, confinement

## Abstract

Confinement within molecular cages can dramatically modify the physicochemical properties of the encapsulated guest molecules, but such host-guest complexes have mainly been studied in a static context. Combining confinement effects with fast guest exchange kinetics could pave the way toward stimuli-responsive supramolecular systems—and ultimately materials—whose desired properties could be tailored “on demand” rapidly and reversibly. Here, we demonstrate rapid guest exchange between inclusion complexes of an open-window coordination cage that can simultaneously accommodate two guest molecules. Working with two types of guests, anthracene derivatives and BODIPY dyes, we show that the former can substantially modify the optical properties of the latter upon noncovalent heterodimer formation. We also studied the light-induced covalent dimerization of encapsulated anthracenes and found large effects of confinement on reaction rates. By coupling the photodimerization with the rapid guest exchange, we developed a new way to modulate fluorescence using external irradiation.

## Highlights

• A coordination cage encapsulates various anthracenes and BODIPY dyes as homodimers
• Mixing the two homodimers leads to the formation of anthracene-BODIPY heterodimers
• Encapsulation can either promote or suppress the photodimerization of anthracenes
• The homodimer/heterodimer equilibrium can be tuned by light-induced guest exchange

## The bigger picture

Confinement of small molecules within the cavities of natural and synthetic hosts can greatly affect the physicochemical properties of the bound species; however, to date, such host-guest complexes have been studied mainly in a static context. An important direction is the development of host-guest systems, whereby encapsulation and release of guest molecules can be reversibly controlled using light. Here, we report ternary inclusion complexes comprising an open-window coordination cage and two kinds of photoactive guests, namely, the photodimerizable anthracenes and BODIPY dyes. Alternating exposure to two different colors of light shifts the equilibrium between the encapsulated homodimers and heterodimers, thus dramatically affecting the system’s optical properties. We also find that the rates of both processes—anthracene dimerization and guest exchange—strongly depend on the substitution pattern on both types of guests, which highlights the importance of confinement effects.

## Graphical abstract

**Figure:**
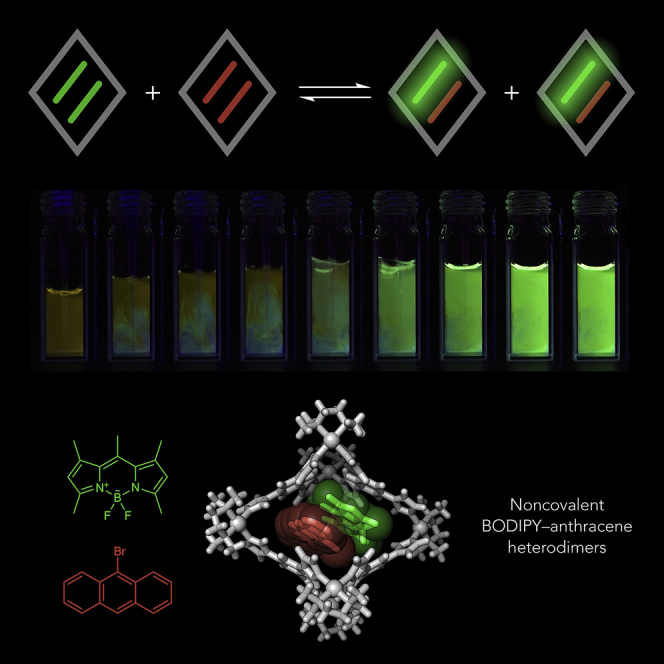

## Teaser

The optical properties of dyes are strongly dependent on their immediate environment. For example, the emission of fluorescent dyes can be effectively quenched upon noncovalent dimer formation. Here, we show that combinations of fluorescent BODIPY dyes and photodimerizable anthracenes can bind as heterodimers inside the cavity of a water-soluble coordination cage. Exposure to light triggers anthracene dimerization, which shifts the homodimer-heterodimer equilibrium and consequently controls the optical properties of the system.

## Introduction

Confining molecules in spaces not much larger than the molecules themselves can profoundly affect their physical and chemical properties.^1^ Diverse types of nanoconfinement have been shown to modulate chemical reactivity of the encapsulated species.^2^^,^^3^ For example, Rebek and co-workers showed that co-encapsulation of phenylacetylene and phenyl azide within a hydrogen-bonded capsule accelerates the 1,3-dipolar cycloaddition reaction between them by a factor of >200.^4^ The same system enabled complete regioselectivity, with only one of two possible triazole isomers formed, in contrast to a 1:1 mixture obtained in a solution of free molecules.^4^ Among other examples, confinement between long alkyl chains of thiolate self-assembled monolayers on gold accelerated a silane alcoholysis reaction,^5^ and confinement between densely packed nanoparticles induced an unusual regioselectivity in a [4+4] cycloaddition, along with rate acceleration.^6^ Conversely, confinement can also decelerate chemical reactions by stabilizing otherwise reactive and/or unstable species. Since Cram’s seminal report on “taming”^7^ cyclobutadiene within a hemicarcerand host, numerous other species were stabilized within—but made to react upon release from—molecular cages, including white phosphorus,^8^ silanol oligomers,^9^ the C_60_ radical anion,^10^ and radical initiators,^11^^,^^12^ whose on-demand release can be used to trigger a free-radical polymerization reaction.^12^ Similarly, encapsulation within molecular containers was found to increase the photochemical stability of fluorescent dyes (such as rhodamine^13^) and to reduce fatigue during the reversible isomerization of the dihydropyrene photoswitch.^14^

Nanoconfinement can also modulate the optical properties of the encapsulated species.^15^^,^^16^ Both absorption and emission of dyes can be altered by encapsulation within the cavities of metal-organic frameworks,^17^ molecular cages,^18^ and protein molecules.^19^ For example, a water-soluble Pt-based coordination cage stabilizes the monomeric form of tetraazaporphine, thus preventing undesired aggregation and enabling strong fluorescence in aqueous media.^20^ Confinement can also promote emission of the bound guest by restricting its conformation.^21^ Hosts with larger cavities can simultaneously encapsulate two guest molecules; such noncovalent dimerization was shown to red-shift and suppress the emission of BODIPY dyes.^22^^,^^23^ In a related study, host-guest interactions were used to assemble coumarin dyes into either H- or J-dimers, depending on the substitution pattern on the coumarin scaffold.^24^ Interestingly, confinement of isomerizable dyes can reverse the relative stability of two isomers, as was demonstrated for phenolphthalein^18^ and a donor-acceptor Stenhouse adduct,^25^ both within Pd-based coordination cages. The properties of confined guests can further be tuned by co-encapsulating them with other guests as ternary complexes of the form (guest⋅guest′)⊂host.^26^, ^27^, ^28^, ^29^, ^30^

However, relatively little attention has been devoted to tuning the above properties rapidly and reversibly by means of repeated encapsulation and release of the guests. This deficiency is most likely due to the closed structure of many molecular hosts, which necessitates a partial disassembly of the host for guest encapsulation/release to take place.^31^, ^32^, ^33^, ^34^, ^35^, ^36^, ^37^ The ability to combine fast guest exchange with encapsulation-induced change in physicochemical properties could facilitate the development of new stimuli-responsive supramolecular systems—and ultimately materials—with rapid response times.

**Scheme 1:**
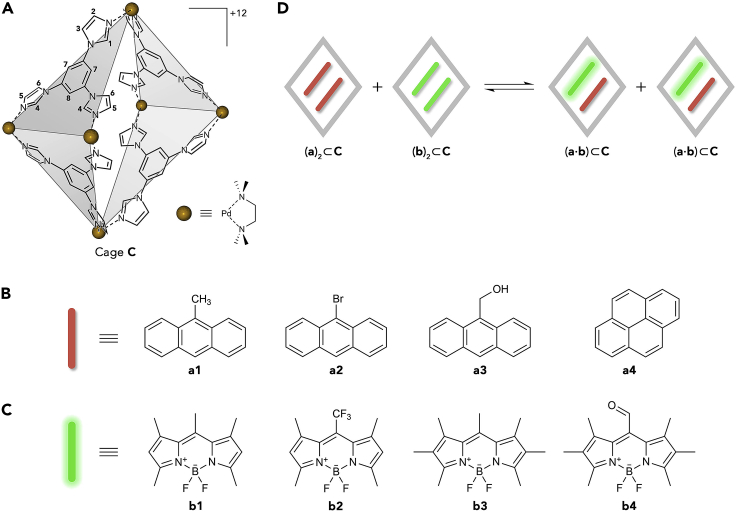
Building blocks of noncovalent homodimers and BODIPY-PAH heterodimers inside the cavity of a coordination cage (A) Structural formula of coordination cage **C**. Counterions = 12 NO_3_^−^. (B) Structural formulas of PAHs used as guests for cage **C** (PAH = polycyclic aromatic hydrocarbon; parent or substituted). (C) Structural formulas of BODIPY dyes used for heterodimer formation. (D) Dynamic equilibrium between weakly fluorescent BODIPY homodimers, weakly fluorescent PAH homodimers, and strongly fluorescent BODIPY-PAH heterodimers.

To this end, we worked with a flexible coordination cage assembled from six *cis*-blocked Pd^2+^ ions and four triimidazolylbenzene (TImB) panels (**C** in Scheme 1A).^38^ Similar to many other cages based on metal-ligand coordination, **C** combines excellent aqueous solubility with the presence of a hydrophobic cavity, which enables it to effectively solubilize various nonpolar molecules in water.^14^^,^^23^^,^^38^ However, **C** offers two additional advantages: first, it contains two large windows, which makes the hydrophobic cavity readily accessible; second, the cage is flexible (owing to the rotation around the C–N bonds connecting the imidazole groups to the panel’s central benzene ring^39^) and can adopt a variety of conformations. This structural flexibility has enabled encapsulation of a variety of structurally diverse guests^23^^,^^39^, ^40^, ^41^, ^42^ and efficient photoisomerization reactions of the encapsulated molecules, even when accompanied by large structural changes.^43^^,^^44^

As a proof-of-concept, we worked with combinations of (1) aromatic compounds **a1**–**a4** (Scheme 1B), which we collectively refer to as polycyclic aromatic hydrocarbons (PAHs; native or substituted), and (2) BODIPY dyes **b1**–**b4** (Scheme 1C). We hypothesized that cage **C** should randomly bind two copies of each of these structurally similar molecules, making the formation of ternary complexes feasible (Scheme 1D). It is well known that, on the one hand, the optical properties of BODIPYs^23^^,^^45^, ^46^, ^47^, ^48^ (and other dyes^49^, ^50^, ^51^, ^52^, ^53^) are strongly dependent on their supramolecular environment; on the other hand, when placed in close proximity, anthracenes can undergo a fast [4+4] cycloaddition reaction,^54^, ^55^, ^56^, ^57^, ^58^ making the BODIPY/PAH combination ideally suited to investigate the exchange dynamics in host-guest inclusion complexes.

## Results and discussion

### Encapsulation of polycyclic aromatic hydrocarbons (PAHs)

**Figure 1:**
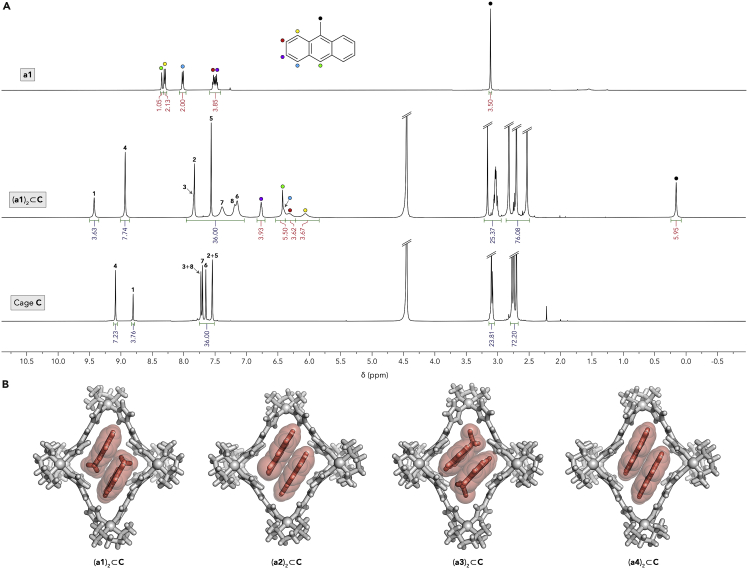
Formation of noncovalent homodimers of PAHs within cage **C** (A) (Top) ^1^H NMR spectrum of **a1** in CDCl_3_ (500 MHz, 298 K). (Middle) ^1^H NMR spectrum of (**a1**)_2_⊂**C** in D_2_O (600 MHz, 330 K). The integrals denoted in blue refer to **C**’s protons; those denoted in red refer to **a1**. The signals originating from **C**’s aromatic protons are denoted with numbers according to the numbering in Scheme 1A. (Bottom) ^1^H NMR spectrum of **C** in D_2_O (400 MHz, 330 K). (B) X-ray crystal structures of homodimeric inclusion complexes (**a1**)_2_⊂**C**, (**a2**)_2_⊂**C**, (**a3**)_2_⊂**C**, and (**a4**)_2_⊂**C**. Nitrate counterions were omitted for clarity.

To encapsulate PAHs **a1**–**a4** within cage **C**, we stirred them (white powders insoluble in water; used in excess) with an aqueous solution of **C**. Encapsulation could be followed by UV-vis absorption spectroscopy, which showed a gradual increase of absorption patterns characteristic of the four PAHs (Figure S15). After 10 h of stirring, no further increase in the intensity of the absorption peaks was observed; we thus concluded that **C**’s cavities were saturated with the PAH guests. After discarding excess (undissolved) PAHs, the solutions of the inclusion complexes were characterized by NMR spectroscopy. As an example, Figure 1A (middle panel) shows the ^1^H NMR spectrum of **a1** encapsulated within **C** (in D_2_O). A comprehensive analysis using a suite of 2D NMR techniques (see Section 5 of the supplemental information) allowed us to assign all the signals in the 1D spectra of this complex (and the other PAH·**C** complexes). Compared with the spectrum of free **a1** in an organic solvent (Figure 1A, top), all the guest protons were upfield-shifted, which can be explained by their residence inside the hydrophobic cavity of the cage, where they experience magnetic shielding by the aromatic walls of the cage. Indeed, the largest shift of ∼2.95 ppm was observed for the CH_3_ group at the central position of the anthracene scaffold. Integrating the signals of the guest versus those of the cage allowed us to confirm that the stoichiometry of the complex is 2:1 and that it forms in a near-quantitative yield—i.e., practically all the cages could be filled with two guest molecules.

Diffusion-ordered NMR spectroscopy (DOSY) showed that all of **a1**’s and **C**’s protons diffused at the same rate, confirming that they constitute a single supramolecular entity (Figure S19). Furthermore, ^1^H–^1^H nuclear Overhauser effect spectroscopy (NOESY) revealed multiple through-space interactions between **a1**’s and **C**’s protons. An in-depth analysis of the NOESY spectra provided important insights into (**a1**)_2_⊂**C**’s solution structure. For example, we found that **a1**’s proton at position 10 (green in Figure 1A) showed a correlation with **C**’s acidic axial imidazole protons (denoted **1** in Scheme 1A and Figure 1A), whereas **a1**’s C**H**_3_ correlated with **C**’s equatorial protons (**6** and **8** in Scheme 1A and Figure 1A). Together, these correlations suggest that the two encapsulated **a1** molecules are oriented antiparallel to each other, with their methyl groups residing in the equatorial area of the cage—an arrangement that was confirmed by single-crystal X-ray crystallography (see below). Detailed NMR characterization of (**a1**)_2_⊂**C** and the other (**a**)_2_⊂**C** complexes is presented in the supplemental information (Section 5).

Single crystals of all four (**a**)_2_⊂**C** complexes were obtained by slow water evaporation from aqueous solutions of the respective complexes. Analysis of the X-ray diffraction data confirmed the presence of two guest molecules inside the hydrophobic pocket of the cage (Figure 1B). The guests’ planes were oriented parallel to each other and to two TImB walls of the cage, forming an extended TImB⋅⋅⋅**a**⋅⋅⋅**a**⋅⋅⋅TImB π-π stack. Upon encapsulating guest molecules, the cage underwent significant axial elongation, with the distance between the two axial Pd nodes increasing from 16.9 Å (for empty **C**) to up to 18.6 Å (for (**a1**)_2_⊂**C**), and the TImB-Pd-TImB angle at the axial Pd decreasing from 88.6° to 74.7°. Similar deformation was observed previously in the complexes of **C** with other small-molecule guests.^14^^,^^23^^,^^38^, ^39^, ^40^^,^^44^ For further structural analysis of (**a1**)_2_⊂**C** and the other (**a**)_2_⊂**C** complexes, see Section 7 of the supplemental information.

Compared with free PAHs in organic solvents, absorption bands of encapsulated PAHs were broader, red-shifted by ∼10 nm, and significantly dampened (Figures S84 and S85). Furthermore, encapsulation was accompanied by a substantial loss of fluorescence (Figure S86), as previously reported for noncovalent dimerization of BODIPY dyes within cage **C**.^23^

### Formation and characterization of noncovalent BODIPY-PAH heterodimers

**Figure 2:**
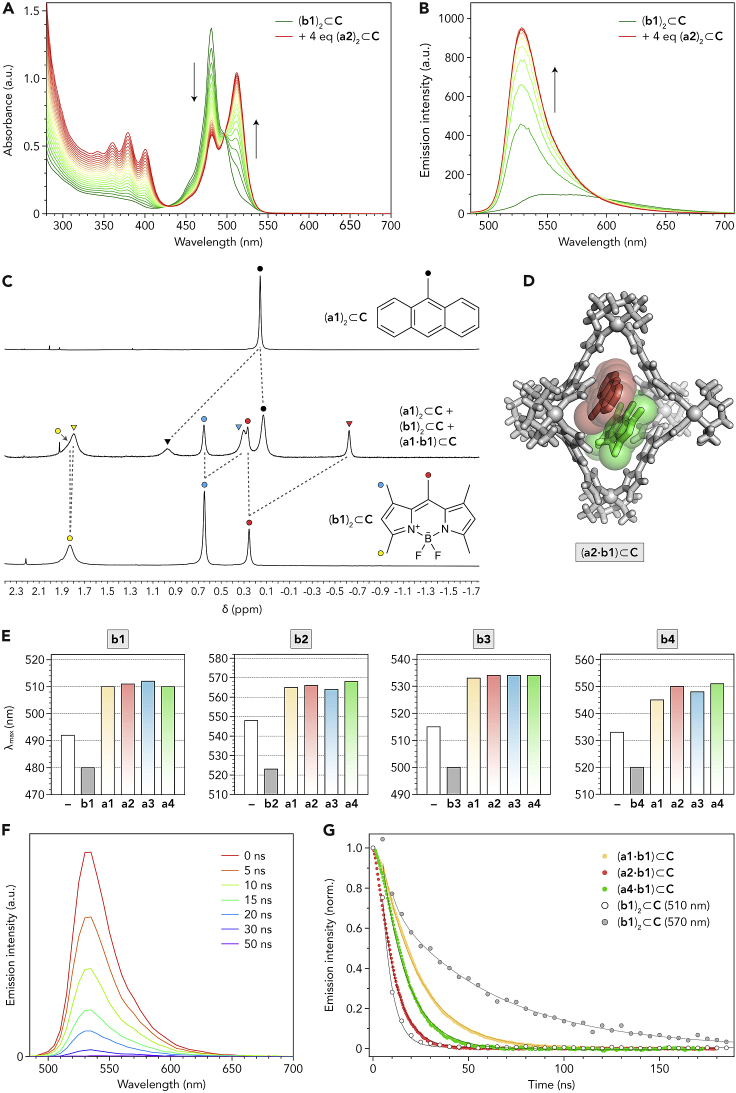
Formation and characterization of noncovalent BODIPY-PAH heterodimers (A) Changes in the UV-vis absorption spectra of an aqueous solution of (**b1**)_2_⊂**C** upon titration with (**a2**)_2_⊂**C** (each spectrum corresponds to an additional 0.2 equiv of (**a2**)_2_⊂**C**). (B) Changes in the emission spectra of an aqueous solution of (**b1**)_2_⊂**C** upon titration with (**a2**)_2_⊂**C** (λ_exc_ = 460 nm; each spectrum corresponds to an additional 0.4 equiv of (**a2**)_2_⊂**C**). (C) Partial NMR spectra of (**a1**)_2_⊂**C** (top; 600 MHz, 330 K), (**b1**)_2_⊂**C** (bottom; 500 MHz, 298 K), and their 2:1 mixture (center; 600 MHz, 320 K) (all in D_2_O), focusing on the aliphatic protons of the encapsulated guests. The circles denote protons of guests within the homodimers, and the triangles denote protons of guests within the heterodimer. (D) X-ray crystal structure of (**a2⋅b1**)⊂**C** (red = **a2**, green = **b1**; counterions and guests’ protons omitted for clarity). (E) Wavelengths of maximum absorption (λ_max_) of **b1**–**b4** as a function of the PAH co-guest within (**a**⋅**b**)⊂**C** ternary complexes (colored bars). Gray bars correspond to (**b**)_2_⊂**C** homodimers; white bars correspond to free **b1**–**b4** dissolved in MeCN. (F) Time-resolved fluorescence spectra of (**a2⋅b1**)⊂**C** in water (λ_exc_ = 460 nm) (to maximize the molar fraction of **b1** within the heterodimer, 40 equiv of (**a2**)_2_⊂**C** with respect to (**b1**)_2_⊂**C** were used). (G) Fluorescence decay traces of **b1** (λ_em_ = 540 nm) within (**a1**⋅**b1**)⊂**C** (yellow), (**a2**⋅**b1**)⊂**C** (red), (**a4**⋅**b1**)⊂**C** (green). Also shown are fluorescence decays for (**b1**)_2_⊂**C** at two different wavelengths (λ_em_ = 510 and 570 nm; empty and solid gray markers, respectively).

Having established that all four PAHs can form homodimeric inclusion complexes (**a**)_2_⊂**C**, we focused on heterodimeric complexes incorporating both PAH and BODIPY, i.e., (**a**⋅**b**)⊂**C** (where “**a**” denotes any of the four PAHs and “**b**” denotes any of the four BODIPYs). In the initial experiments, we titrated aqueous solutions of (**b**)_2_⊂**C** with (**a**)_2_⊂**C** (at room temperature) and followed the reaction:

(**a**)_2_⊂**C** + (**b**)_2_⊂**C** → 2 (**a**⋅**b**)⊂**C**

by UV-vis spectroscopy. For example, Figure 2A shows the results of titrating (**b1**)_2_⊂**C** with (**a2**)_2_⊂**C**, where the absorption at 480 nm due to (**b1**)_2_⊂**C** declined at the expense of a new peak centered at 511 nm. Complex (**a2**)_2_⊂**C** does not absorb in the visible region, and the new peak can be attributed to the heterodimer (**a2⋅b1**)⊂**C**. To maximize the fraction of **b1** within the heterodimer, we continued the titration until 4 equiv of (**a2**)_2_⊂**C** were added. We also followed the titration using fluorescence spectroscopy and found a dramatic increase in the emission intensity (Figure 2B), with the emission spectrum of (**a2⋅b1**)⊂**C** resembling that of free **b1** in an organic solvent more than that of the weakly fluorescent (**b1**)_2_⊂**C** H-dimer.^23^

Alternatively, the heterodimeric inclusion complexes could be formed by treating any (**b**)_2_⊂**C** homodimer with solid **a** (and vice versa), according to the reaction equation:

(**b**)_2_⊂**C** + **a**↓ → (**a**⋅**b**)⊂**C** + **b↓**

For example, stirring an aqueous solution of (**b1**)_2_⊂**C** with an excess of **a2** (insoluble in water) resulted in a UV-vis spectrum very similar to that obtained by mixing (**b1**)_2_⊂**C** with (**a2**)_2_⊂**C**; at the same time, we observed that the white solid **a2** turned red, indicating partial expulsion of **b1** from the cage, followed by its precipitation from water (supplemental information, Section 10).

Depending on the identity of PAH and BODIPY, the equilibrium between the homodimers and the heterodimer could be shifted in either direction. For example, upon adding 4 equiv of (**a4**)_2_⊂**C** to a solution of (**b1**)_2_⊂**C**, an intense heterodimer peak at 510 nm was observed, accompanied by low absorption at 480 nm due to the residual homodimer (Figure S90C). When, however, (**b1**)_2_⊂**C** was treated with 4 equiv of (**a1**)_2_⊂**C** instead, the absorbance values at 480 and 510 nm were similar (despite the large excess of **a1**), indicating that the formation of heterodimer was less favorable (Figure S90A). Similarly, replacing one BODIPY with another for a given PAH significantly affected the homodimer/heterodimer ratio. This effect was particularly pronounced for **b2**, which has a strong tendency to form heterodimers. As shown in Figure S91A, the addition of only ∼1 equiv of (**a1**)_2_⊂**C** converted a vast majority of (**b2**)_2_⊂**C** into (**a1**⋅**b2**)⊂**C**, in sharp contrast to the small amount of (**a1⋅b1**)⊂**C** formed by mixing the corresponding homodimers in a 1:1 ratio (Figure S90A).

The formation of encapsulated heterodimers could also be followed by NMR spectroscopy. Starting with homodimeric complexes of four PAHs and four BODIPYs (Scheme 1C), we obtained all 16 heterodimer combinations; NMR characterization of representative examples is shown in the supplemental information, Section 6. As an example, Figure 2C shows a partial ^1^H NMR spectrum, obtained by mixing (**a1**)_2_⊂**C** with (**b1**)_2_⊂**C** in D_2_O. In addition to the two homodimers, the spectrum shows a new set of peaks, which can be assigned to both guests residing within (**a1⋅b1**)⊂**C** (see the exchange correlations in Figure S66). Interestingly, upon heterodimer formation, **a1**’s and **b1**’s proton resonances were shifted in opposite directions (Figure 2C). Upon replacing one guest in (**a1**)_2_⊂**C** with **b1**, the methyl protons of the remaining **a1** moved downfield by ∼0.8 ppm. By contrast, **b1**’s methyl protons at the *meso* and β′ positions (red and blue in Figure 2C) shifted upfield (by ∼0.9 and ∼0.35 ppm, respectively). These results are a manifestation of a higher degree of aromaticity of anthracene compared with BODIPY, and they remind us that chemical shifts of encapsulated guests depend strongly not only on the host (here, cage **C**) but also on the co-guests with which they are co-confined. Notably, the splitting of **b1**’s methyl protons at the α position (yellow in Figure 2C) was much less pronounced—i.e., this singlet does not shift noticeably upon replacing one of the two **b1** within the (**b1**)_2_⊂**C** homodimer with **a1**. This observation suggests that these protons do not reside directly above **a1**’s aromatic system—a conclusion that was confirmed by an X-ray structure of a similar heterodimer (see below).

To prove the existence of heterodimeric complexes (**a**⋅**b**)⊂**C** directly, we attempted to determine the X-ray crystal structure of a representative heterodimer. We focused on (**a2⋅b1**)⊂**C** since it contains the prototypical member of the BODIPY family—the penta-methyl-substituted **b1**—and a PAH with a high electron density on the Br substituent to facilitate structure determination. To this end, we mixed aqueous solutions of (**a2**)_2_⊂**C** and (**b1**)_2_⊂**C** and left the resulting solution undisturbed. Once most water had evaporated, we observed the formation of a mixture of colorless and orange crystals and manually collected the latter for X-ray diffraction. We found the coexistence of the (**b1**)_2_⊂**C** homodimer (with a structure nearly identical to that reported previously^23^) and two different conformations of (**a2⋅b1**)⊂**C** in the crystal lattice. Both conformations featured a TImB⋅⋅⋅**a2**⋅⋅⋅**b1**⋅⋅⋅TImB π-π stack, with **b1**’s BF_2_ moiety facing the axial Pd nodes (as in the structure of pure (**b1**)_2_⊂**C**^23^), but they differed in the orientation of **a2**. In the first conformer, **a2**’s Br substituent pointed toward the equatorial area of the cage, as shown in Figure 2D; in the second, it was oriented in the opposite direction, toward the axial region (Figure S80). Density functional theory (DFT) calculations suggested that the latter conformer is slightly more stable (supplemental information, Section 8). We also attempted to detect the ternary complex (**a2⋅b1**)⊂**C** by electrospray ionization mass spectrometry (ESI-MS), but measurements under various conditions repeatedly showed the empty cage and the two guests as separate species. Similarly, no signals for the intact homodimeric complexes (**a2**)_2_⊂**C** and (**b1**)_2_⊂**C** were detected. These results are in agreement with the fast expulsion of guests through the large windows of cage **C** during mass spectrometry measurements.

Next, we studied how varying the PAH guest within (**a**⋅**b**)⊂**C** heterodimers affects the optical properties of the encapsulated BODIPY. As described above, replacing one of two BODIPYs in (**b**)_2_⊂**C** homodimers with a PAH dramatically red-shifts the absorption maximum of the remaining BODIPY (by ∼30 nm for **b1**, **b3**, and **b4**, and by ∼40 nm for **b2**; gray versus colored bars in Figure 2E). Interestingly, however, remarkably little variation was found when one PAH was replaced with another for a given BODIPY; for example, **b1** within all four heterodimers absorbed at 511 nm (±1 nm) (colored bars in Figure 2E). Whereas the fluorescence emission for all (**a**⋅**b1**)⊂**C** complexes was centered at 528 nm, the PAHs had a large effect on **b1**’s fluorescence quantum yields (Φ_F_ = 0.31, 0.21, 0.41, and 0.50 for **b1** co-encapsulated with **a1**, **a2**, **a3**, and **a4**, respectively; Φ_F_ = 0.13 for the (**b1**)_2_⊂**C** homodimer^23^ and 1.00 for free **b1** in chloroform^59^). Similarly, we observed significant differences in the fluorescence decay profiles. Figure 2F shows a typical series of time-resolved fluorescence spectra recorded after exciting (**a2⋅b1**)⊂**C** with a 5-ns 460 nm pulse. The fluorescence decay can be fitted to a single-exponential curve (Figure 2G) with a time constant, τ = 8.66 (±0.43) ns (the errors correspond to deviations from the ideal single-exponential curve). This behavior is reminiscent of that of free **b1** in MeCN, whose fluorescence decays with τ = 6.71 (±0.15) ns,^23^ but stands in sharp contrast with the (**b1**)_2_⊂**C** homodimer, which exhibits a more complex decay profile with a slower decay at longer wavelengths (570 nm), indicative of H-aggregation.^23^ For (**a1⋅b1**)⊂**C** and (**a4⋅b1**)⊂**C**, we determined τ as 13.26 (±0.07) and 21.83 (±0.13) ns, respectively.

### Kinetics of guest exchange

**Figure 3:**
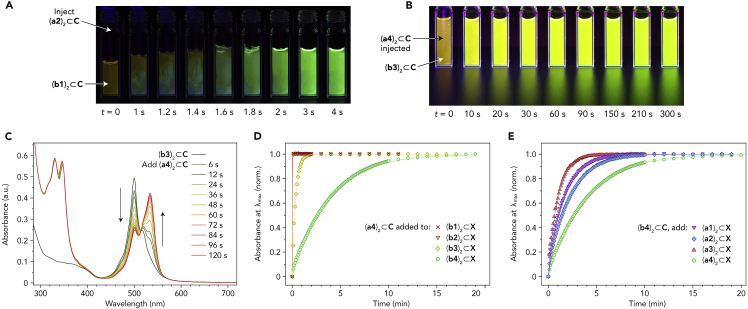
Kinetics of heterodimer formation via guest exchange (A) Photographs taken during and immediately after the injection of 2 equiv of (**a2**)_2_⊂**C** in water into an aqueous solution of (**b1**)_2_⊂**C**. (B) Photographs taken at various times following the injection of 10 equiv of (**a4**)_2_⊂**C** in water into an aqueous solution of (**b3**)_2_⊂**C**. (C) Changes in the UV-vis absorption spectra of an aqueous solution (**b3**)_2_⊂**C** following injection of 10 equiv of (**a4**)_2_⊂**C** in water. (D) Monitoring the formation kinetics of the four (**a4⋅b**)⊂**C** heterodimers by following the absorbance at the wavelength of maximum absorption (λ_max_) of each heterodimer. For **b1** and **b2**, a near-instantaneous equilibration was observed. Markers: experimental data points; lines: fits to an asymptotic exponent $1\;-\;e^{-kt}$. (E) Monitoring the formation kinetics of the four (**a**⋅**b4**)⊂**C** heterodimers by following the absorbance at each heterodimer’s λ_max_. Markers: experimental data points; lines: fits to an asymptotic exponent $1\;-\;e^{-kt}$.

Next, we studied the kinetics of heterodimer formation via guest exchange, (**a**)_2_⊂**C** + (**b**)_2_⊂**C** → 2 (**a**⋅**b**)⊂**C**, hypothesizing that changing the bulkiness of either guest might have a large effect on the reaction kinetics. In the initial experiments, we injected aqueous solutions of (**a**)_2_⊂**C** into solutions of (**b**)_2_⊂**C** and followed the reaction with the naked eye. Working with two relatively small guests **a2** and **b1**, we found that the mixture turned strongly fluorescent instantaneously (Figures 3A and S94). However, upon replacing **a2** and **b1** with the bulkier **a4** and **b3**, respectively, the system required several minutes to equilibrate (Figure 3B; see the gradual change in emission color and the gradual increase of emission intensity, which can be appreciated from the vial’s reflection on the benchtop).

The finding that the optical properties of **b3** (and other BODIPY dyes) depend strongly on the identity of the guest with which it shares the cavity of **C** allowed us to conveniently monitor the reaction using UV-vis absorption spectroscopy. Figure 3C shows the evolution of UV-vis spectra observed upon treating (**b3**)_2_⊂**C** with 10 equiv of (**a4**)_2_⊂**C**; the 500 nm absorption peak due to (**b3**)_2_⊂**C** decreased over 2 min, whereas the 534 nm peak due to (**a4⋅b3**)⊂**C** gradually increased. Interestingly, **a4**’s absorbance in the near-UV region remained largely unaltered throughout the reaction (compare the spectra at 6 versus 120 s). However, the visible part of the spectrum shows that the reaction (**a4**)_2_⊂**C** + (**b3**)_2_⊂**C** → 2 (**a4⋅b3**)⊂**C** proceeds to a negligible extent within the initial 6 s. Together, these observations allow us to conclude that the optical properties of pyrene, unlike those of BODIPYs, show little dependence on its co-guest (see also Figure S96E for the **a4**/**b4** combination). The same was found to be true for anthracenes **a1**–**a3**—see, e.g., Figure S97A.

To determine whether the dramatic deceleration of guest exchange (compare Figure 3A with 3B) was caused primarily by increasing the bulkiness of PAH (**a2** to **a4**) or that of BODIPY (**b1** to **b3**), we varied the identity of BODIPY for a given PAH and vice versa. First, we injected (**a4**)_2_⊂**C** into all four (**b**)_2_⊂**C** homodimers and monitored the exchange by following the absorbance at the wavelength of maximum absorption of each (**a4**⋅**b**)⊂**C** complex (Figure 3D). Whereas **b1** and **b2** equilibrated very rapidly and **b3** took ∼2 min to equilibrate, the bulkiest **b4** needed more than 20 min to reach equilibrium. By fitting the data to a single exponential function $y=1-e^{-kt}$ (see supplemental information, Section 11 for derivation), we determined the equilibration rates as *k* = 51, 2.8, and 0.26 min^−1^ for **b1**, **b3**, and **b4**, respectively. Notably, with **b2**, the reaction proceeded even faster than it did for the smaller **b1** and reached completion by the time the first data point (t = 6 s) was recorded; this finding can be explained by the relatively low stability of the (**b2**)_2_⊂**C** homodimer with respect to **b2**'s complexes with PAHs (see above). In the second set of experiments, we injected all four (**a**)_2_⊂**C** homodimers into four identical solutions of (**b4**)_2_⊂**C** and monitored the absorbance at the wavelength of maximum absorption of the respective (**a**⋅**b4**)⊂**C** complexes (Figure 3E). Interestingly, varying the PAH had relatively little influence on guest exchange kinetics (at least in this series); we found *k* = 0.65, 0.48, 0.27, and 1.1 min^−1^ for **a1**, **a2**, **a3**, and **a4**, respectively.

**Figure 4:**
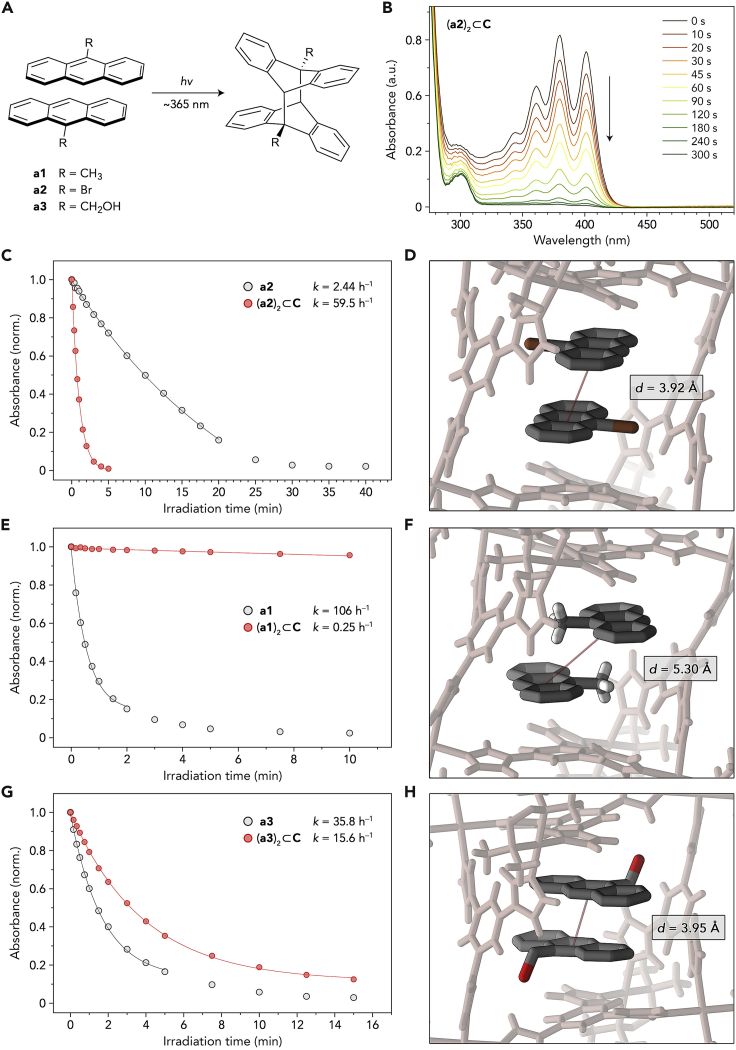
[4+4] photodimerization of confined anthracenes (A) Reaction scheme. (B) Changes in the UV-vis absorption spectra of an aqueous solution of (**a2**)_2_⊂**C** exposed to UV (365 nm) light. (C) Monitoring the [4+4] photodimerization of free **a2** in DCM (gray) and (**a2**)_2_⊂**C** in water (red) by following absorbance at 393 and 401 nm, respectively. Markers: experimental data points; lines: fits to the kinetic equation for a first-order reaction ($y\;=\;e^{-xk}$; note that photodimerization in DCM was accompanied by a slower, side reaction leading to a byproduct whose absorption overlapped with that of anthracene; therefore, only the initial points were used for obtaining the fits; also for E and G). (D) Excerpt from the X-ray crystal structure of (**a2**)_2_⊂**C**, focusing the orientation of guests within **C** (counterions and guests’ protons omitted for clarity; also for F and H). (E) Monitoring the [4+4] photodimerization of free **a1** in DCM (gray) and (**a1**)_2_⊂**C** in water (red) by following absorbance at 390 and 398 nm, respectively. Markers: experimental data points; lines: fits to a first-order reaction. (F) Excerpt from the X-ray crystal structure of (**a1**)_2_⊂**C**, focusing the orientation of guests within **C**. (G) Monitoring the [4+4] photodimerization of free **a3** in DCM (gray) and (**a3**)_2_⊂**C** in water (red) by following absorbance at 387 and 393 nm, respectively. Markers: experimental data points; lines: fits to a first-order reaction. (H) Excerpt from the X-ray crystal structure of (**a3**)_2_⊂**C**, focusing the orientation of guests within **C** (one of two conformers in the crystal; the structure of the second conformer is very similar, with a center-to-center distance of 3.85 Å; see CCDC: 2103576).

Having demonstrated that replacing one guest molecule in a (**b**)_2_⊂**C** complex with a PAH greatly increased the emission intensity of the encapsulated BODIPY, we hypothesized that selectively removing the PAH component from (**a**⋅**b**)⊂**C** could shift the equilibrium back toward (**b**)_2_⊂**C**, thus restoring the initial, low emission of the solution. An elegant way to effectively remove an anthracene from the system is to convert it into a covalent dimer, which can be achieved by irradiation with near-UV (∼365 nm) light (Figure 4A). We further speculated that the resulting dianthracene would occupy the entire cavity of cage **C**. Thus, covalent dimerization of an anthracene is expected to force noncovalent dimerization of BODIPY dye. To verify this hypothesis, we first studied the ability of anthracenes confined within cage **C** to photodimerize.

### Photoresponsiveness of encapsulated PAHs

Upon UV irradiation, anthracenes undergo [4+4] cycloaddition to afford dianthracenes (Figure 4A)—a reaction that has been investigated extensively in solution^60^, ^61^, ^62^ and in confined spaces.^63^, ^64^, ^65^, ^66^, ^67^, ^68^ We hypothesized that encapsulation within the cage will not only increase the effective molarity of anthracenes but might also preorganize them in a way that allows for favorable orbital overlap, thus greatly increasing the reaction kinetics. Figure 4B shows a series of UV-vis spectra recorded after exposing (**a2**)_2_⊂**C** in water to increasing periods of near-UV light. After 5 min of irradiation, anthracene’s characteristic absorption pattern disappeared, indicating complete conversion to the corresponding dianthracene, which we denote **a2a2**. No precipitation was observed, suggesting that the product remained encapsulated as (**a2a2**)⊂**C**. Interestingly, the reaction proceeded significantly faster than the photodimerization of free **a2** dissolved in DCM, which required UV exposure of >30 min under the same irradiation conditions for **a2**’s absorption to fully disappear (Figure S99). By plotting the absorption of **a2** over time and fitting the data to a first-order decay,^69^ we found *k* = 2.44 and 59.5 h^−1^ for the free and encapsulated **a2**, respectively, corresponding to reaction acceleration by a factor of ∼25 (Figure 4C).

In addition to the increased rate, the reaction proceeded cleaner, leaving behind a nearly featureless spectrum above 320 nm (Figure 4B). By contrast, photodimerization of free **a2** in DCM was accompanied by a gradual increase of absorption at ∼325 nm (Figure S99), indicating the formation of a side product, most likely a bianthracenyl.^70^ The high selectivity of photodimerization of confined **a2** could also be appreciated from NMR spectra of (**a2**)_2_⊂**C** (3 mm in D_2_O) exposed to UV light. Following 6 min of UV irradiation, the signals originating from encapsulated **a2** disappeared and were replaced by a new set of signals in a 1:1:1:1 ratio (Figures S100 and S101) due to the aromatic protons of encapsulated **a2a2**. Only one aliphatic signal was observed, indicating the formation of a single isomer of dianthracene (in our case, head-to-tail (*h-t*)-**a2a2**). After a total of 7 min of irradiation, CDCl_3_ was added, and the reaction product was extracted from the aqueous phase. An NMR spectrum of the organic phase revealed the presence of remarkably clean *h-t*-**a2a2**—in sharp contrast to free **a2** in CDCl_3_, whose irradiation for a longer time (160 min) led to only ∼65% of **a2a2**, in addition to side products (Figure S102).

Interestingly, the reaction (**a2**)_2_⊂**C** → (**a2a2**)⊂**C** was accompanied by pronounced changes in the chemical shifts of cage **C**’s protons (Figure S100), in particular, the acidic imidazole protons (**1** and **4** in Scheme 1A). These changes indicate a large structural distortion of the cage, which it has to undergo to adapt to **a2a2**, whose shape is significantly different^71^ from that of two stacked **a2** molecules. In fact, following UV irradiation, we observed slow precipitation of a white solid as a result of the expulsion of **a2a2** from **C**. No such precipitation was observed in UV-vis experiments, which were conducted at much lower (micromolar) concentrations. These results explain why, despite extensive efforts, we did not succeed in obtaining single crystals of (**a2a2**)⊂**C** suitable for X-ray diffraction.

To determine whether the encapsulation-induced acceleration of [4+4] cycloaddition is a general phenomenon, we studied the behavior of encapsulated **a1**. Surprisingly, UV irradiation of (**a1**)_2_⊂**C** resulted in very small changes in the UV-vis spectra, indicating that the cycloaddition reaction is significantly hampered—despite **C**’s ability to bring two copies of **a1** into close proximity. By fitting the collected data points to a first-order decay (Figure 4E), we obtained *k* = 0.25 h^−1^, corresponding to reaction deceleration (compared with free **a1** in DCM) by a factor of >400.

The X-ray structures of (**a1**)_2_⊂**C** and (**a2**)_2_⊂**C** provide insights into the contrasting impact of confinement on the photoreactivity of these two complexes (Figures 4D and 4F). In both cases, the guest molecules are oriented antiparallel to each other, with the same plane-to-plane distance of 3.54 Å (the distance between the planes defined by the anthracenes’ central rings). However, in the case of (**a1**)_2_⊂**C**, the guests were significantly offset with respect to each other, with a center-to-center distance of as much as 5.30 Å (compared with 3.92 Å for **a2**; the center-to-center distance is defined as the distance between the centroids of the central rings of the two anthracenes). The offset orientation in (**a1**)_2_⊂**C**, likely stabilized by C–H⋅⋅⋅π interactions between the two guest molecules, effectively suppresses the reaction. These results indicate that the “topochemical postulate,” originally formulated for the solid state,^72^^,^^73^ applies to molecules confined within the cavities of soluble cages as well. To support these results, we also worked with **a3**; upon UV irradiation, [4+4] cycloaddition within (**a3**)_2_⊂**C** took place (Figure 4G), albeit ∼4 times slower than in the case of (**a2**)_2_⊂**C**. The X-ray structure of (**a3**)_2_⊂**C** features two conformers of the inclusion complex, with center-to-center distances of 3.95 (Figure 4H) and 3.85 Å, rendering the photodimerization reaction topochemically allowed.^72^^,^^73^

### Noncovalent heterodimers with photoswitchable fluorescence

**Figure 5:**
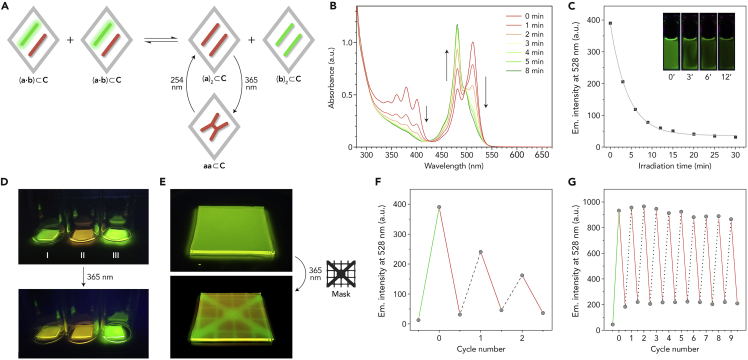
Controlling the homodimer/heterodimer equilibrium with light (A) Shifting the equilibrium between strongly fluorescent BODIPY heterodimers (left) and weakly fluorescent homodimers (right) using light-responsive anthracenes. (B) Changes in the UV-vis absorption spectra of a 4:1 mixture of (**a2**)_2_⊂**C** and (**b1**)_2_⊂**C** induced by UV (365 nm) irradiation for up to 8 min. (C) Decrease of emission intensity of a 4:1 mixture of (**a2**)_2_⊂**C** and (**b1**)_2_⊂**C** induced by UV (365 nm) irradiation for up to 30 min (see Figure S109 for the original spectra). Markers: experimental data points; curve: fit to a single exponential decay (τ = 4.17 (±0.11) min). Inset: photographs showing gradual disappearance of green fluorescence during exposure to UV light for up to 12 min. (D) Photographs of 10 × 10 × 1 mm pieces of agarose gels soaked with: (**a2**)_2_⊂**C** + (**b1**)_2_⊂**C** (gel I), (**b1**)_2_⊂**C** (gel II), and (**a4**)_2_⊂**C** + (**b1**)_2_⊂**C** (gel III) before (top) and after (bottom) UV irradiation for 3 min. (E) Photographs of a 35 × 25 × 1 mm piece of agarose gel soaked with a 4:1 mixture of (**a2**)_2_⊂**C** and (**b1**)_2_⊂**C** before (top) and after (bottom) UV irradiation through a mask. (F) Reversible changes in the emission intensity of a 4:1 mixture of (**a2**)_2_⊂**C** and (**b1**)_2_⊂**C** upon alternating irradiation with 254 nm light for 25 min (denoted with dashed black lines) and 365 nm light for 12 min (solid red lines). The first data point corresponds to (**b1**)_2_⊂**C** and the green line corresponds to addition of 4 equiv (**a2**)_2_⊂**C**. (G) Reversible changes in the emission intensity of a 4:1 mixture of (**a2**)_2_⊂**C** and (**b1**)_2_⊂**C** upon alternating irradiation with 365 nm light for 25 min (denoted with red lines) and addition of 4 equiv (**a2**)_2_⊂**C** (dotted black lines). The first data point corresponds to (**b1**)_2_⊂**C** and the green line—addition of 4 equiv of (**a2**)_2_⊂**C**.

Finally, we integrate the two findings described above—fast guest exchange and efficient cycloaddition of encapsulated anthracenes—to construct a supramolecular system exhibiting light-switchable fluorescence (Figure 5A). Having identified **a2** as an anthracene that undergoes a rapid cyclodimerization reaction and having determined that guests **a2** and **b1** shuttle between different cages rapidly, we speculated that the equilibrium between the highly fluorescent **a2⋅b1** and the weakly fluorescent (**b1**)_2_ H-dimer could be shifted toward the latter by effectively removing **a2** from the system as the covalent dimer **a2a2**:

$$2\;\left(a2\cdot b1\right)\subset C\;\overset{\text{UV}}{\to \;}\left(a2a2\right)\subset C+{\left(b1\right)}_{2}\subset C$$

To this end, we mixed (**a2**)_2_⊂**C** with (**b1**)_2_⊂**C** in a 4:1 ratio, thus placing a vast majority of **b1** within the (**a2⋅b1**)⊂**C** heterodimer (red spectrum in Figure 5B). Upon near-UV (365 nm) irradiation, the 511 nm peak decreased, whereas the 480 nm peak grew, indicating regeneration of (**b1**)_2_⊂**C**. The system reached equilibrium within ∼8 min of UV irradiation, corresponding to the time required to complete the [4+4] cycloaddition reaction, thus confirming that the rate-determining step was the cycloaddition rather than guest exchange. As expected, no changes in the UV-vis spectra were observed when **a2** was replaced with either **a1** (which dimerizes very slowly; Figure S107A) or **a4** (photochemically inactive; Figure S107B). However, replacing **b1** with other BODIPYs afforded systems that did exhibit the photoinduced modulation of optical properties—see Figures S107C and S107D for the **a2**/**b2** and **a2**/**b4** combinations, respectively. The reaction could also be followed by fluorescence spectroscopy, where the emission at 528 nm decreased by more than 12-fold (Figure 5C).

Next, we hypothesized that by UV-irradiating a sample containing the highly emissive (**a2⋅b1**)⊂**C** locally (i.e., through a mask), it should be possible to create fluorescent patterns. To this end, we first prepared three thin (10 × 10 × 1 mm) pieces of agarose hydrogels and soaked them in an aqueous solution of (**b1**)_2_⊂**C**; the gels exhibited weak orange emission. Then, the gels were transferred into vials containing the following: a solution of (**a2**)_2_⊂**C** (sample I), pure water (sample II), and a solution of (**a4**)_2_⊂**C** (sample III). After soaking for 1 h, gels I and III assumed bright green fluorescence, owing to the formation of heterodimers (**a2⋅b1**)⊂**C** and (**a4⋅b1**)⊂**C**, respectively (Figure 5D). Then, all three gels were exposed to near-UV light (365 nm) for 3 min. As expected, UV irradiation had no effect on gels II and III; the former did not contain any PAH, and the latter contained the photochemically inactive **a4**. However, irradiation of gel I triggered the formation of (**a2a2**)⊂**C**, thus forcing **b1** into the weakly fluorescent (**b1**)_2_⊂**C** homodimer; consequently, the emission of this sample, after exposure to UV, resembled that of the control gel II (Figure 5D). To demonstrate the ability to pattern gels containing the light-responsive heterodimer, we prepared a larger (35 × 25 × 1 mm) piece of agarose gel soaked with a 4:1 mixture (**a2**)_2_⊂**C** and (**b1**)_2_⊂**C** and brought it into conformal contact with a mask. UV irradiation induced the [4+4] cycloaddition coupled with guest exchange only in the exposed regions, decreasing emission intensity and turning the emission color orange (Figure 5E).

Anthracene cyclodimerization can be reversed upon irradiation with UV light of higher energy (<300 nm). Therefore, we hypothesized that the homodimer/heterodimer ratio—and, consequently, the solution’s emission intensity—could be tuned reversibly using UV light of two different wavelengths. Indeed, when a 4:1 mixture of encapsulated dianthracene (**a2a2**)⊂**C** and (**b1**)_2_⊂**C** was exposed to 254 nm light for 30 min, a substantial amount of the original anthracene **a2**—and, consequently, of the fluorescent (**a2⋅b1**)⊂**C** heterodimer—was regenerated (Figure S108). Unfortunately, the dedimerization reaction did not reach completion, and further deterioration of the system was observed in the subsequent cycles (Figure 5F). It is important to emphasize that the observed fatigue was most likely caused by the inherent instability of **a2**—rather than of host **C**—under 254 nm light (Figure S103); in fact, we previously reported highly reversible switching of another photoresponsive compound (dihydropyrene) within **C**, despite prolonged exposure to 254 nm light.^14^ To address the problem of photodegradation induced by 254 nm light, we developed an alternative system based on cycles of (1) irradiation with 365 nm light and (2) addition of a fresh aliquot of (**a2**)_2_⊂**C** (4 equiv). Completing each cycle “resets” the system to the initial, highly fluorescent state: (**b1**)_2_⊂**C** + (**a2**)_2_⊂**C** → 2 (**a2⋅b1**)⊂**C**. Although each cycle accumulates (**a2a2**)⊂**C**, this species is not fluorescent, unresponsive to 365 nm UV light, and, being a 1:1 complex, it does not interfere with the **b1** homodimer/heterodimer equilibrium; thus, it behaves as a non-invasive waste. Indeed, this strategy allowed us to perform at least ten on/off cycles without noticeable fatigue (Figure 5G).

### Conclusions

In summary, we developed a supramolecular system based on a combination of three components—a coordination cage, BODIPY dyes, and aromatic hydrocarbons (PAHs)—that allowed us to study the dynamics of host-guest inclusion complexes using optical spectroscopies. The cage’s cavity is of a size that enables the simultaneous encapsulation of two guest molecules—either two BODIPYs, two PAHs, or a BODIPY-PAH heterodimer. Mixing a BODIPY homodimer with a PAH homodimer initiates guest exchange, resulting in a heterodimer with distinct absorption and fluorescence features. We found that, depending on the identity of BODIPY and PAH, heterodimer formation can be either instantaneous or require tens of minutes to complete. In parallel, we investigated the UV-induced cyclodimerization of the confined PAHs (anthracenes). Compared with anthracenes dissolved in an organic solvent, the cyclodimerization of confined anthracenes could be either dramatically accelerated or remarkably decelerated, depending on the substitution pattern on the anthracene ring; these results could be rationalized by the "topochemical postulate." Finally, we integrated the UV-induced cyclodimerization reaction with the rapidly equilibrating inclusion complexes; beyond the development of a conceptually novel light-controlled fluorescence switch, this unique combination of covalent and noncovalent reactions brings supramolecular host-guest inclusion complexes closer to the realm of systems chemistry.

## Experimental procedures

Full experimental procedures can be found in the supplemental information.

### Resource availability

#### Lead contact

Further information and requests for resources and reagents should be directed to the lead contact, Rafal Klajn (rafal.klajn@weizmann.ac.il).

#### Materials availability

This study did not generate unique reagents.

### General procedure for the formation of homodimeric and heterodimeric inclusion complexes

A solution of cage **C** (10.0 mg, 3.1 μmol) in H_2_O or D_2_O (0.5 mL) was added to an excess (5–10 equiv) of solid PAH (**a1**, **a2**, **a3**, or **a4**) or BODIPY (**b1**, **b2**, **b3**, or **b4**) and the resulting suspension was stirred overnight at room temperature in the dark (all the PAHs and BODIPYs are insoluble in water in the unencapsulated form). The undissolved solids were removed by several cycles of centrifugation or by filtration through a syringe filter to afford solutions of homodimeric inclusion complexes (**a**)_2_⊂**C** and (**b**)_2_⊂**C**. Heterodimeric inclusion complexes (**a**⋅**b**)⊂**C** were formed by mixing aqueous solutions of the respective homodimeric complexes (**a**)_2_⊂**C** and (**b**)_2_⊂**C**. The resulting solutions of homodimeric and heterodimeric inclusion complexes were stable in the dark at ambient temperature for at least several months.

## Data Availability

All data supporting the findings of this study are included within the article and its supplemental information and are also available from the authors upon request. Crystallographic data for the structures reported in this paper have been deposited at the Cambridge Crystallographic Data Centre, under the deposition numbers CCDC 2103596 for (**a1**)_2_⊂**C**, CCDC 2103597 for (**a2**)_2_⊂**C**, CCDC 2103576 for (**a3**)_2_⊂**C**, CCDC 2103598 for (**a4**)_2_⊂**C**, and CCDC 2103596 for (**a2**⋅**b1**)⊂**C**. Copies of these data can be obtained free of charge via www.ccdc.cam.ac.uk/data_request/cif.

## Supplemental information

Document S1. Supplemental experimental procedures, Figures S1–S108, and supplemental references

Document S2. Article plus supplemental information
