# Supplementary material for: Ternary host-guest complexes with rapid exchange kinetics and photoswitchable fluorescence
Source: Chem. 2022 Sep 8;8(9):2362–79. doi: 10.1016/j.chempr.2022.05.008 (PMC9473544; doi:10.1016/j.chempr.2022.05.008)
Supplement: Document S1. Supplemental experimental procedures, Figures S1–S108, and supplemental references [file mmc1.pdf]

Chem, Volume *8*

## Supplemental information

# Ternary host-guest complexes

# with rapid exchange kinetics

# and photoswitchable fluorescence

**Julius Gemen, Michał J. Białek, Miri Kazes, Linda J.W. Shimon, Moran Feller, Sergey N. Semenov, Yael Diskin-Posner, Dan Oron, and Rafal Klajn**

Table of contents

I. Supplemental experimental procedures ........................................................................................2

1. Materials and methods ...........................................................................................................2

2. Synthesis and characterization of cage **C** and guests **a1**–**a4** and **b1**–**b4** ...............................................3

3. Following the frmation of homodimeric complexes............................................................................17

4. NMR characterization of BODIPY homodimers....................................................................18

5. NMR characterization of homodimers (**a1**)$_2$⊂**C**, (**a2**)$_2$⊂**C**, (**a3**)$_2$⊂**C**, and (**a4**)$_2$⊂**C** ..........................19

6. NMR characterization of heterodimers (**a1**·**b1**)⊂**C**, (**a2**·**b1**)⊂**C**, (**a1**·**b2**)⊂**C**, and (**a4**·**b4**)⊂**C** ..........63

7. X-ray data collection and structure refinement........................................................................77

8. DFT calculations of the (**a2**·**b1**)⊂**C** heterodimer......................................................................84

9. Steady-state optical properties of **a1**, **a2**, **a3**, and **a4** and their inclusion complexes..........................85

10. Formation of heterodimeric inclusion complexes and their steady-state optical properties.............88

11. Kinetics of heterodimer formation.........................................................................................93

12. Time-resolved fluorescence spectroscopy of heterodimeric inclusion complexes...........................97

13. Photodimerization of encapsulated **a1**–**a4** ...........................................................................98

14. Photoresponsiveness of heterodimeric inclusion complexes ...................................................103

15. Preparation of photoresponsive gels based on heterodimeric inclusion complexes .......................105

II. Supplemental references ...........................................................................................................105

# I. Supplemental experimental procedures

## 1. Materials and methods

All commercial chemicals were used as received unless stated otherwise. NMR spectra were recorded on a Bruker Avance III 400 MHz spectrometer, a Bruker Avance III HD 500 MHz spectrometer, or a Bruker Avance III 600 MHz spectrometer. Chemical shifts ($\delta$) are given in ppm relative to residual proton solvent resonances (4.79 ppm for $D_2O$ and 7.26 ppm for $CDCl_3$). For spectra recorded in $D_2O$ at higher temperatures, the resonance of residual solvent was set to 4.55 ppm for 320 K, 4.45 for 330 K, and 4.35 ppm for 340 K, according to Ref. 1. $^1$H DOSY measurements were performed on a Bruker Avance III 500 MHz spectrometer or a Bruker Avance III 600 MHz spectrometer at room temperature. Solution-state UV-vis absorption spectra were recorded with an Agilent Cary 60 spectrophotometer. Emission and excitation spectra were recorded with a Shimadzu spectrofluorophotometer RF-5301 PC. Fluorescence quantum yields were determined on a Quantaurus-QY Absolute PL quantum yield spectrometer. Mass spectra were recorded on a Waters Xevo G2-XS quadrupole time-of-flight high-resolution mass spectrometer with an electrospray ionization ion source, operated under MassLynx software. For details on the X-ray data collection and refinement, see Section 7. For details on density functional theory (DFT) calculations, see Section 8. For details on time-resolved fluorescence spectroscopy, see Section 12.

## 2. Synthesis and characterization of cage **C** and guests **a1**–**a4** and **b1**–**b4**

Cage **C** was synthesized according to a previously reported literature procedure.[2]

$^1$H NMR (500 MHz, $D_2O$, 298 K): $\delta$ = 9.12 (s, 8H, $\mathbf{C_4}$), 8.83 (s, 4H, $\mathbf{C_1}$), 7.75 (s, 4H, $\mathbf{C_8}$), 7.73 (s, 4H, $\mathbf{C_3}$), 7.71 (s, 8H, $\mathbf{C_7}$), 7.66 (s, 8H, $\mathbf{C_6}$), 7.56 (s, 12H, $\mathbf{C_{2+5}}$), 3.16–3.08 (s, 24H, $\mathbf{C_9}$), 2.82–2.69 (m, 72H, $\mathbf{C_{10}}$).

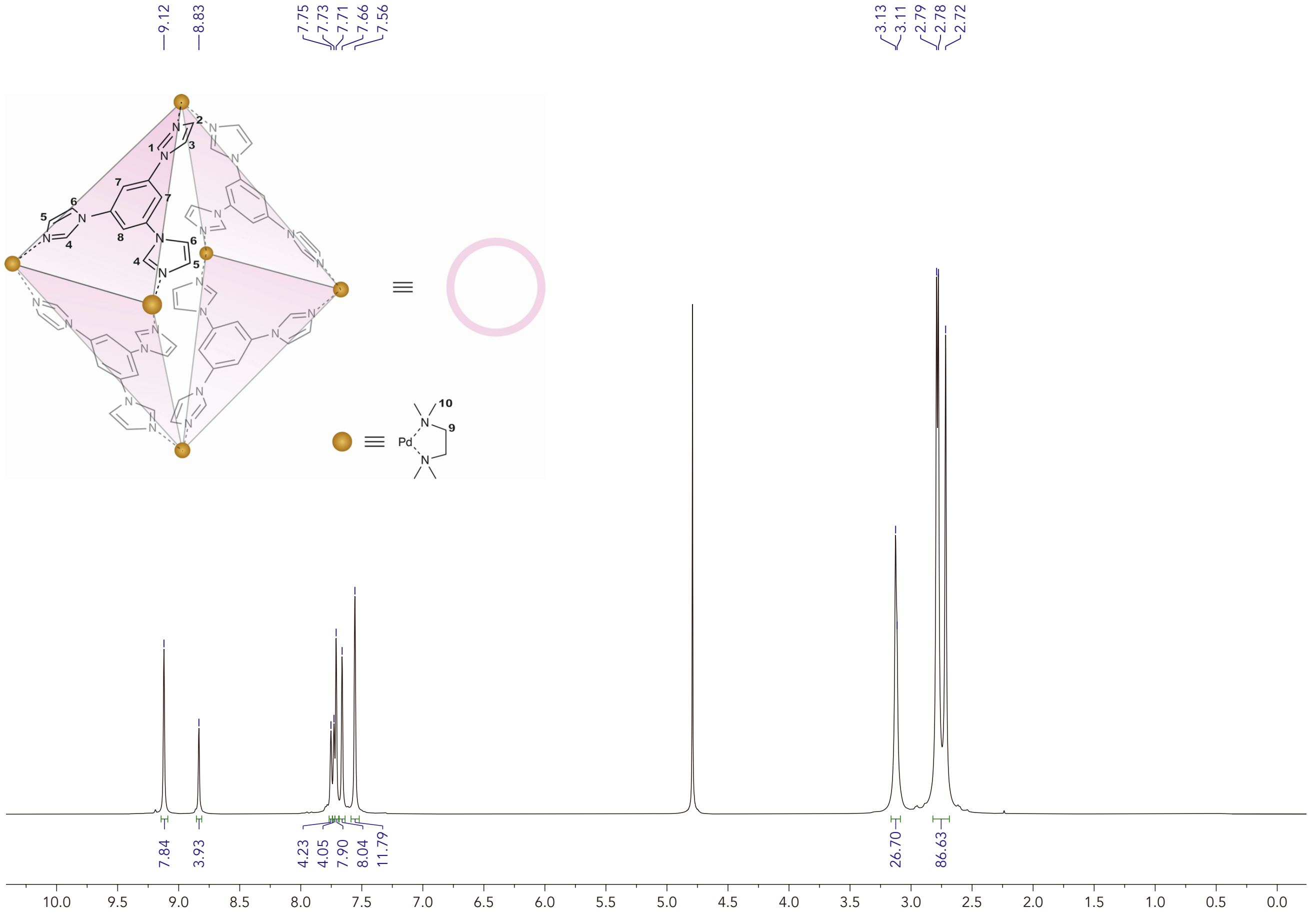


**Figure S1.** $^1$H NMR spectrum of cage **C** (500 MHz, $D_2O$, 298 K).

Anthracenes **a1**–**a3** and pyrene (**a4**) are commercially available compounds. Their NMR characterization is provided below to facilitate the characterization of their inclusion complexes (Sections 5 and 6).

**a1**:

$^{1}$H NMR (500 MHz, $CDCl_3$, 298 K): $\delta$ = 8.35 (s, 1H, **a1**$_{5}$), 8.30 (d, 2H, **a1**$_{1}$), 8.01 (d, 2H, **a1**$_{4}$), 7.52 (t, 2H, **a1**$_{2}$), 7.48 (t, 2H, **a1**$_{3}$), 3.11 (s, 3H, **a1**$_{CH_3}$).

$^{13}$C NMR (100 MHz, $CDCl_3$, 298 K): $\delta$ = 131.6 (**a1**$_{8}$), 130.2 (**a1**$_{7}$), 130.2 (**a1**$_{6}$), 129.2 (**a1**$_{4}$), 125.4 (**a1**$_{5}$), 125.3 (**a1**$_{3}$), 124.9 (**a1**$_{2}$), 124.8 (**a1**$_{1}$), 14.0 (**a1**$_{CH_3}$).

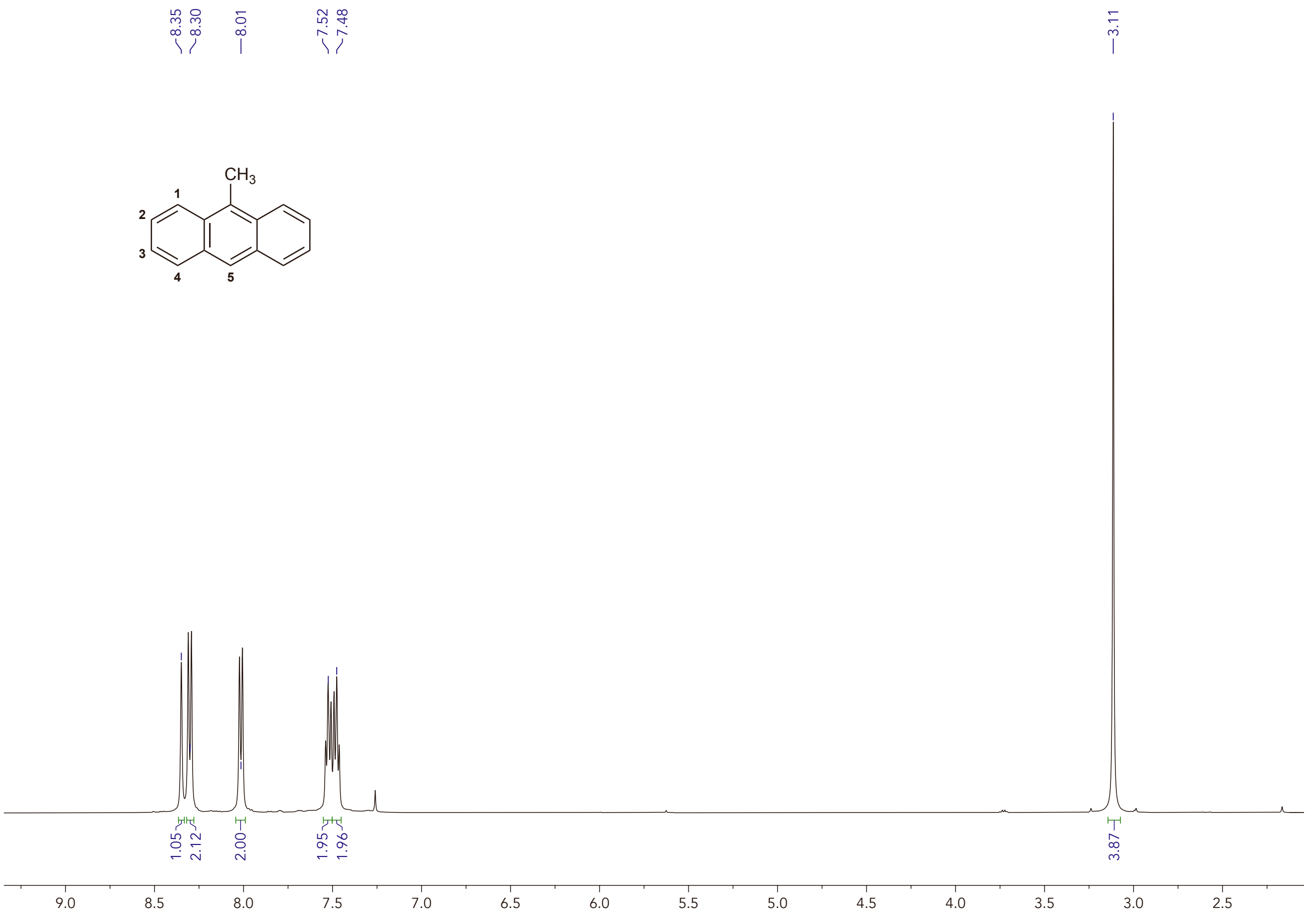


**Figure S2.** $^{1}$H NMR spectrum of **a1** (500 MHz, $CDCl_3$, 298 K).

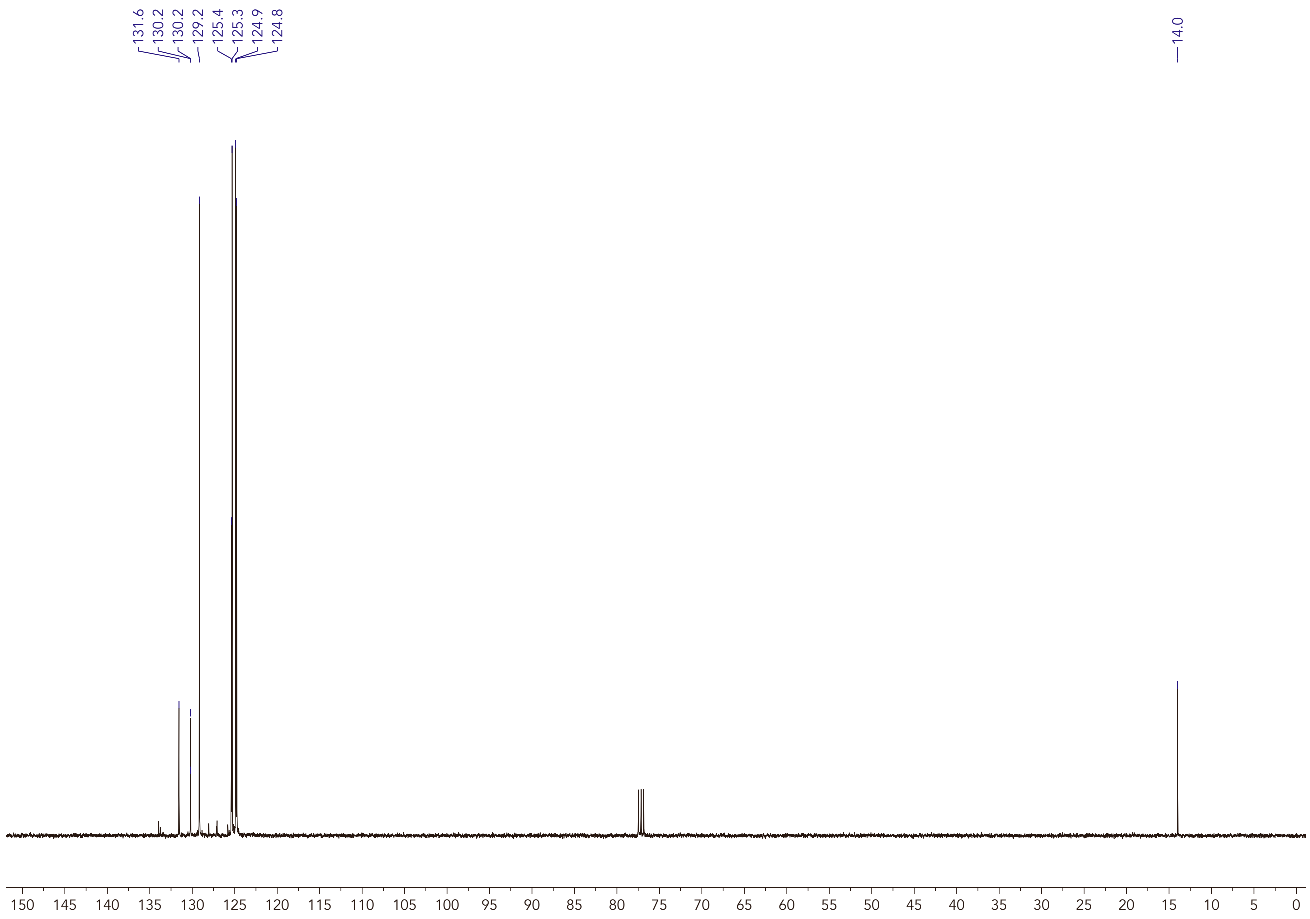


**Figure S3.** $^{13}C$ NMR spectrum of **a1** (100 MHz, $CDCl_3$, 298 K).

**a2**:

$^{1}$H NMR (500 MHz, $CDCl_3$, 298 K): $\delta$ = 8.53 (d, 2H, **a2$_1$**), 8.43 (s, 1H, **a2$_5$**), 7.99 (d, 2H, **a2$_4$**), 7.61 (t, 2H, **a2$_3$**), 7.51 (t, 2H, **a2$_2$**).

$^{13}$C NMR (100 MHz, $CDCl_3$, 298 K): $\delta$ = 132.2 (**a2$_8$**), 130.6 (**a2$_7$**), 128.7 (**a2$_4$**), 127.7 (**a2$_1$**), 127.3 (**a2$_2$**), 127.2 (**a2$_5$**), 125.7 (**a2$_3$**), 122.4 (**a2$_6$**).

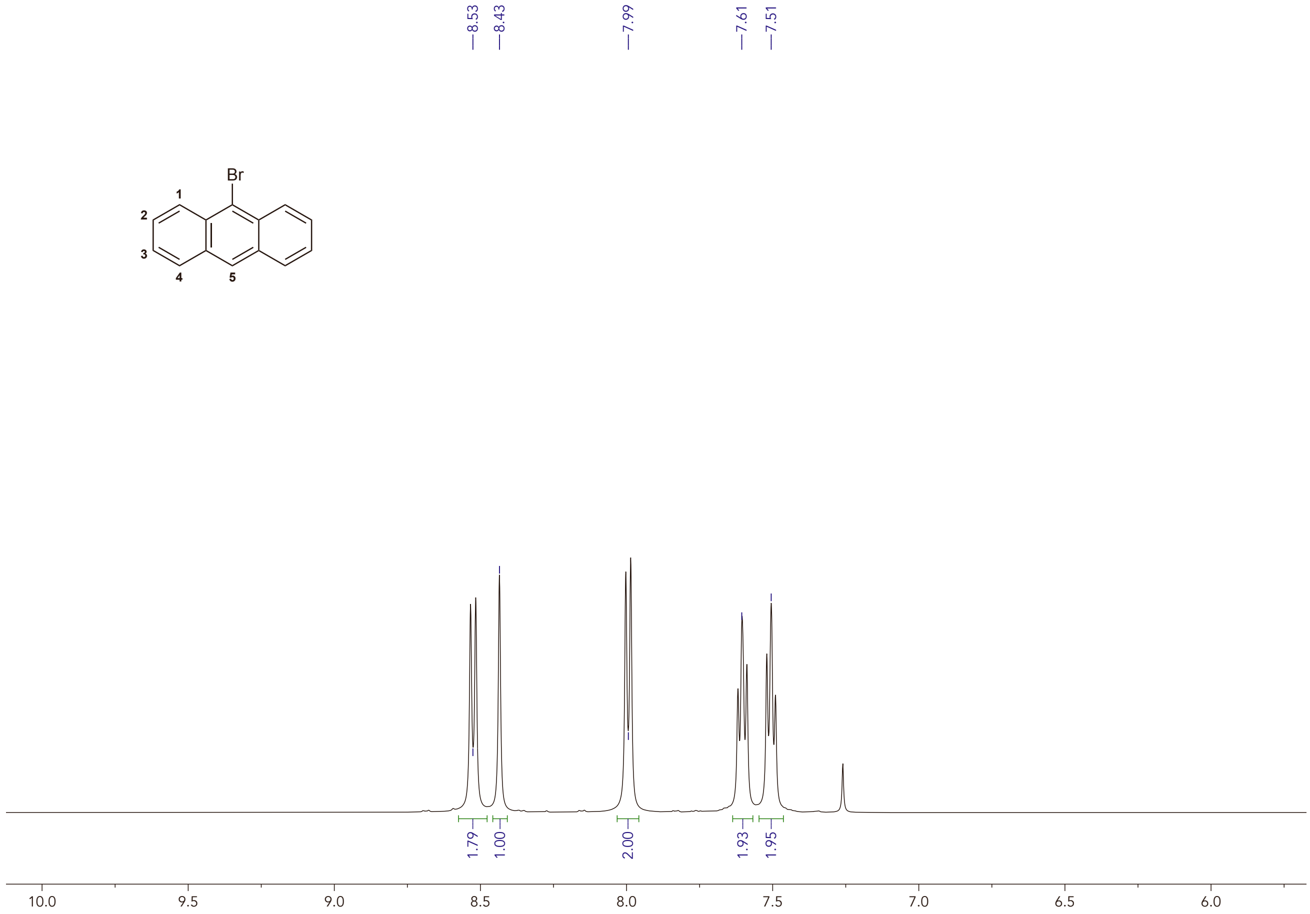


**Figure S4.** $^{1}$H NMR spectrum of **a2** (500 MHz, $CDCl_3$, 298 K).

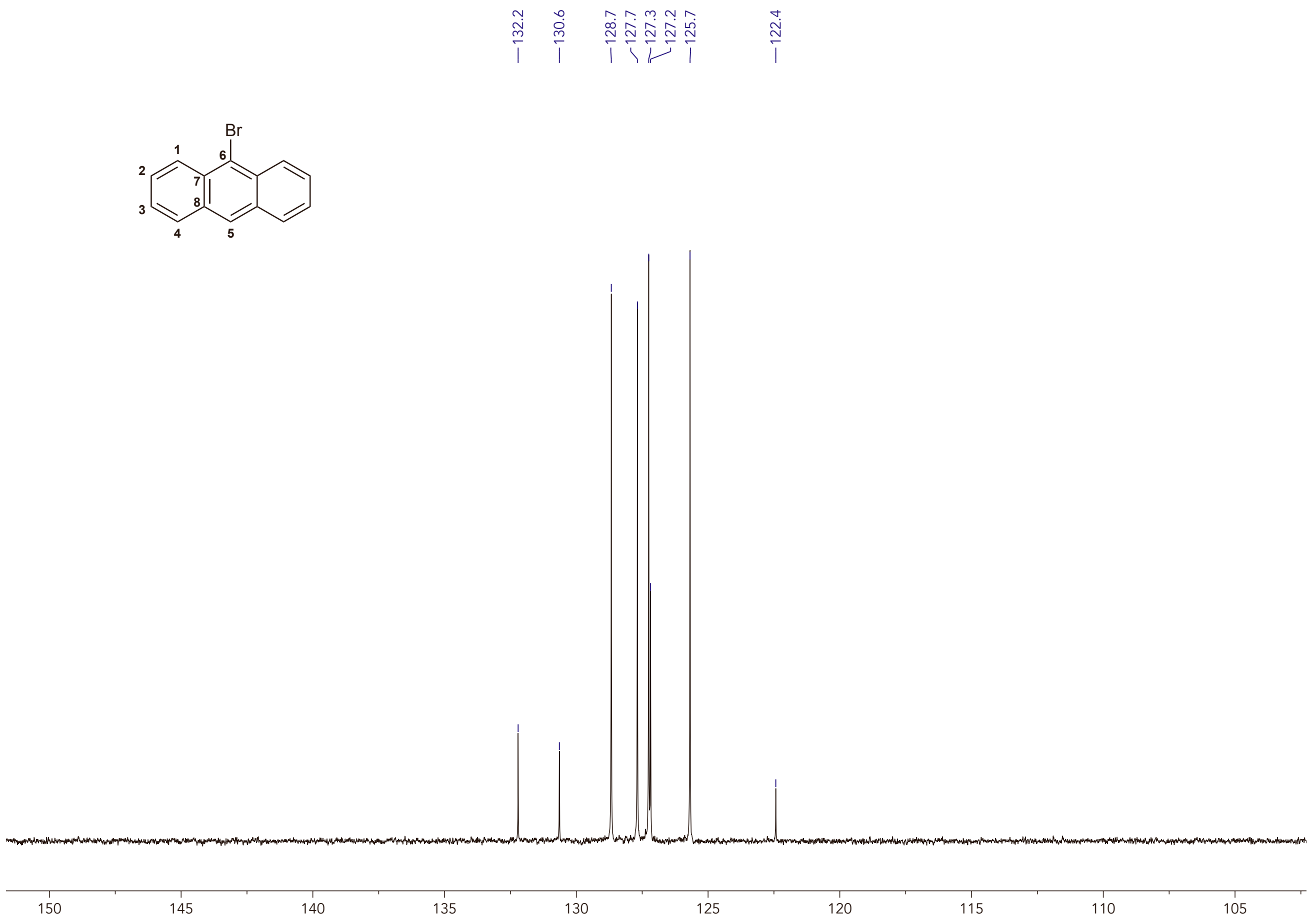


**Figure S5.** $^{13}C$ NMR spectrum of **a2** (100 MHz, $CDCl_3$, 298 K).

**a3**:

$^{1}$H NMR (400 MHz, $CDCl_3$, 298 K): $\delta$ = 8.45 (s, 1H, **a3**$_{5}$), 8.39 (d, 2H, **a3**$_{1}$), 8.02 (d, 2H, **a3**$_{4}$), 7.56 (t, 2H, **a3**$_{2}$), 7.48 (t, 2H, **a3**$_{3}$), 5.64 (s, 2H, **a3**$_{CH_2}$).

$^{13}$C NMR (100 MHz, $CDCl_3$, 298 K): $\delta$ = 131.7 (**a3**$_{6}$), 131.1 (**a3**$_{7}$), 130.4 (**a3**$_{8}$), 129.3 (**a3**$_{4}$), 128.5 (**a3**$_{5}$), 126.6 (**a3**$_{3}$), 125.2 (**a3**$_{2}$), 124.0 (**a3**$_{1}$), 57.5 (**a3**$_{CH_2}$).

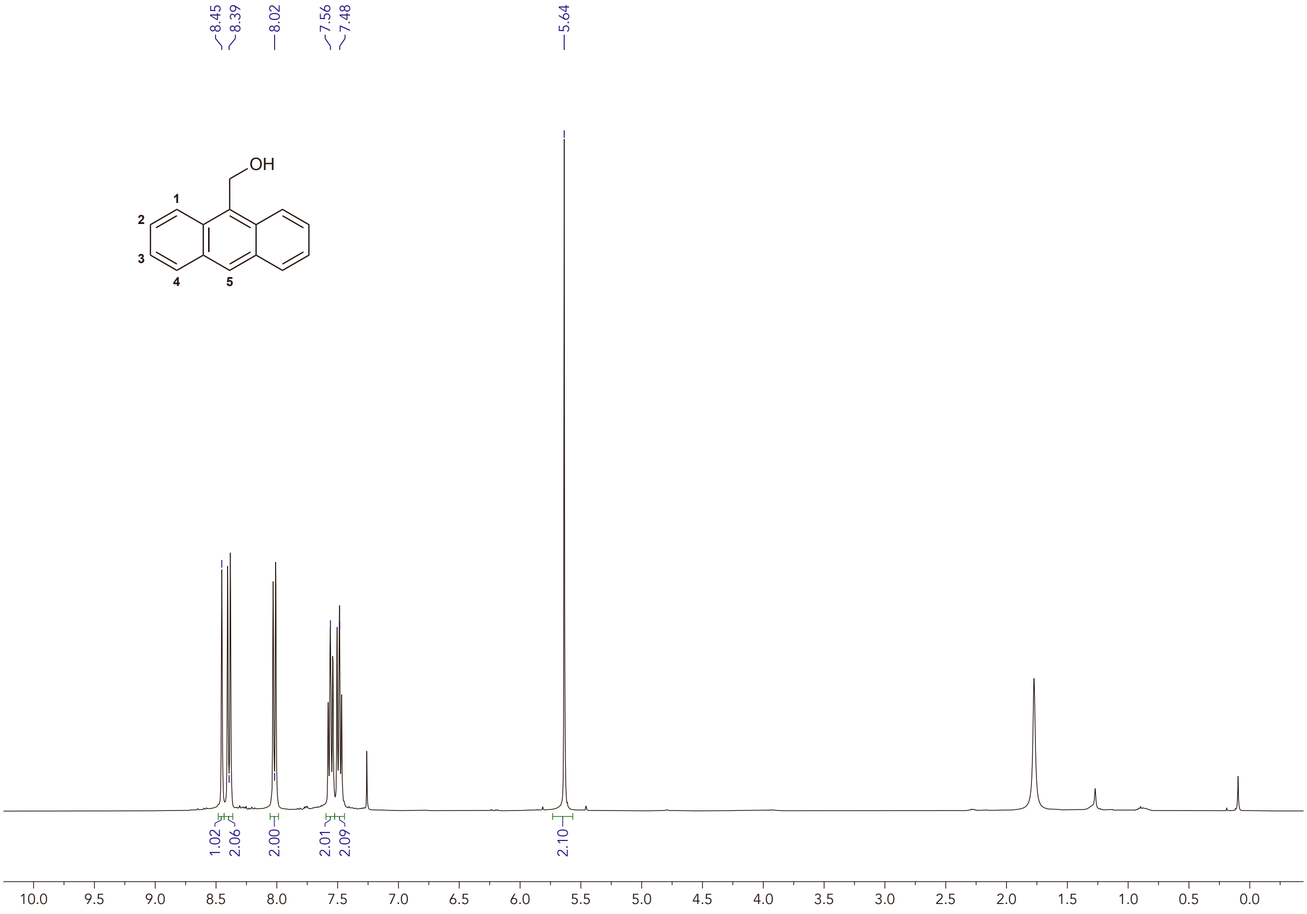


**Figure S6.** $^{1}$H NMR spectrum of **a3** (400 MHz, $CDCl_3$, 298 K).

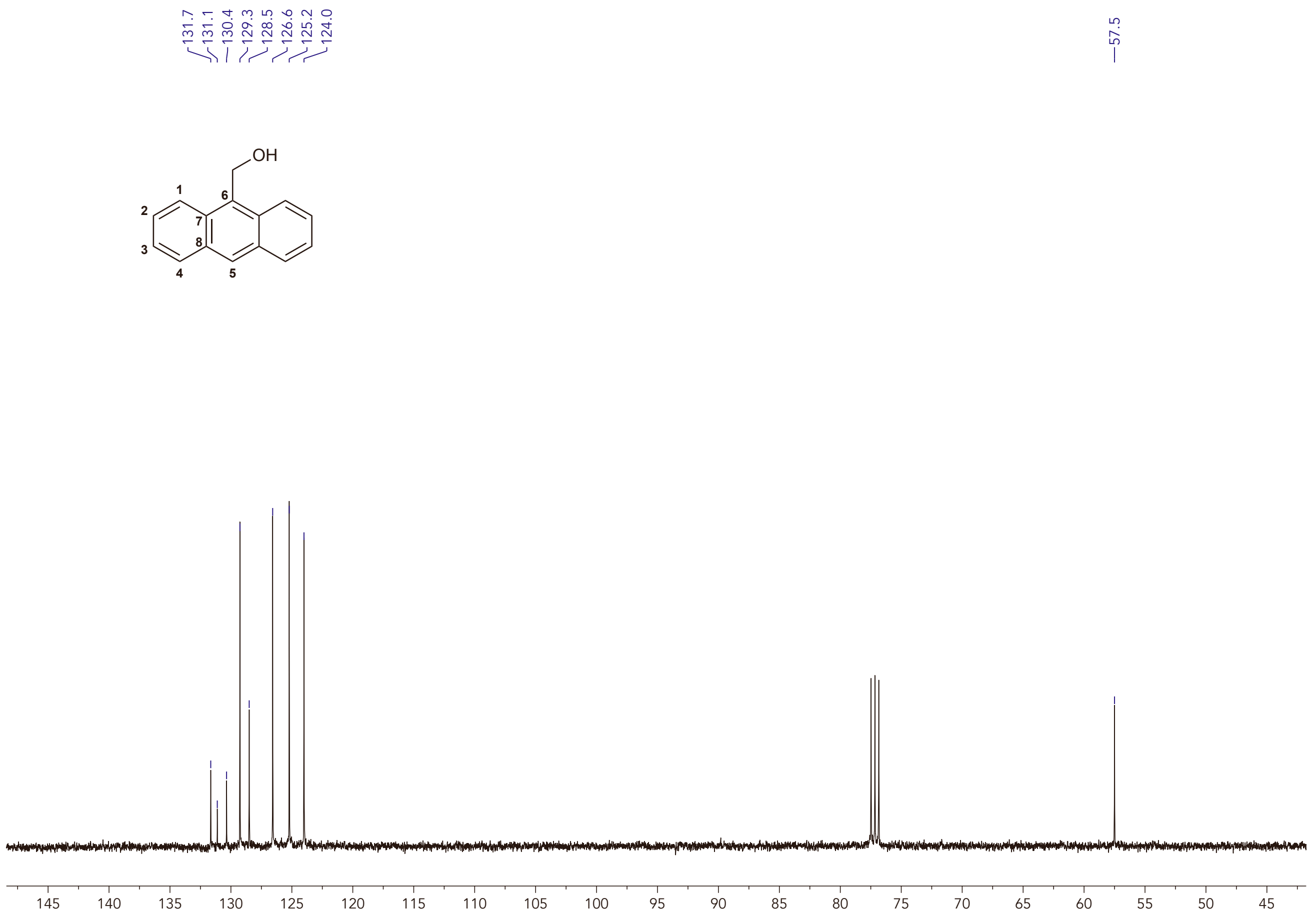


**Figure S7.** $^{13}C$ NMR spectrum of **a3** (100 MHz, $CDCl_3$, 298 K).

**a4**:

$^{1}$H NMR (500 MHz, $CDCl_3$, 298 K): $\delta$ = 8.20 (d, 4H, **a4$_1$**), 8.09 (s, 4H, **a4$_3$**), 8.02 (t, 2H, **a4$_2$**).

$^{13}$C NMR (100 MHz, $CDCl_3$, 298 K): $\delta$ = 131.2 (**a4$_4$**), 127.4 (**a4$_3$**), 125.9 (**a4$_2$**), 125.0 (**a4$_1$**), 124.7 (**a4$_5$**).

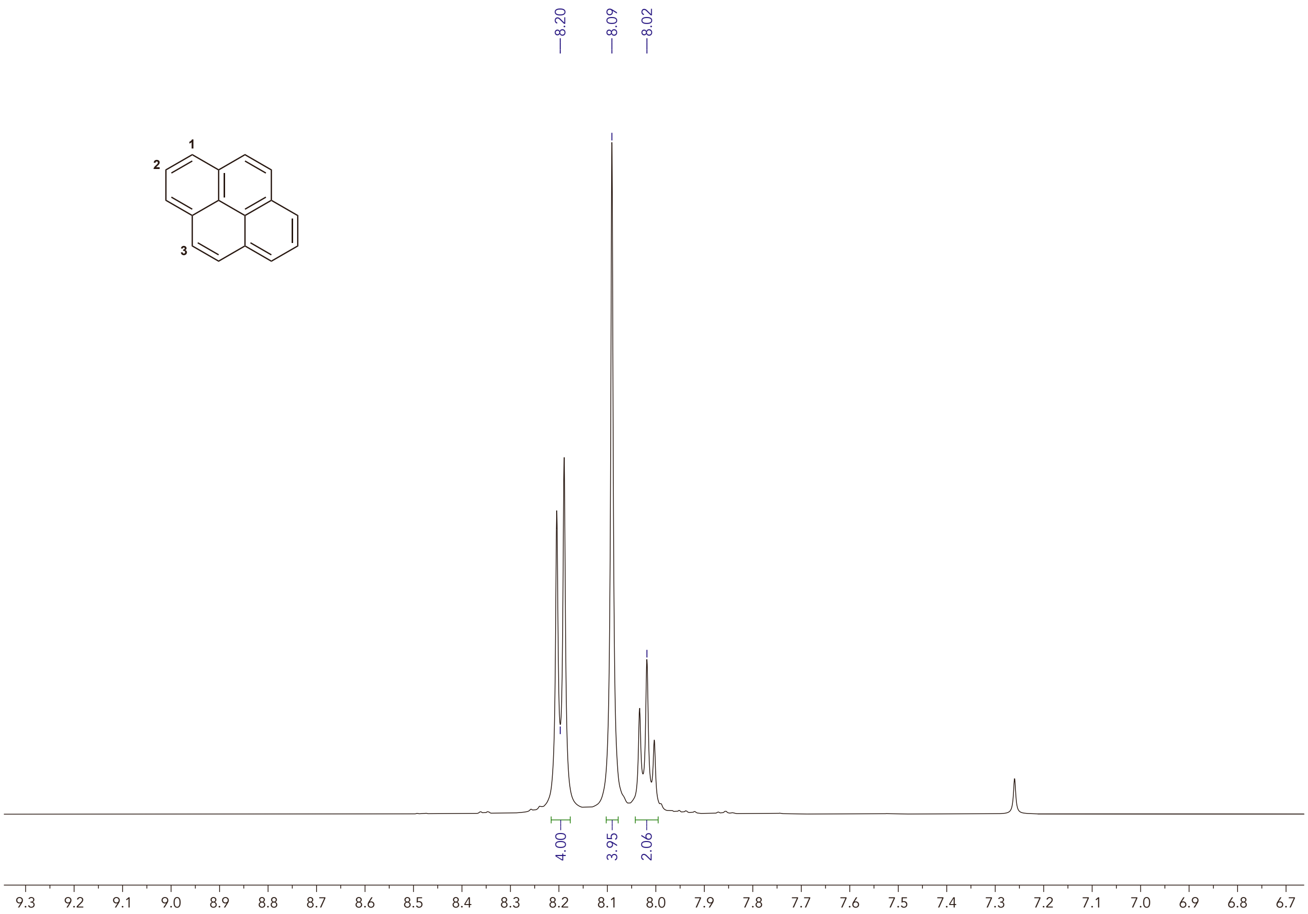


**Figure S8.** $^{1}$H NMR spectrum of **a4** (500 MHz, $CDCl_3$, 298 K).

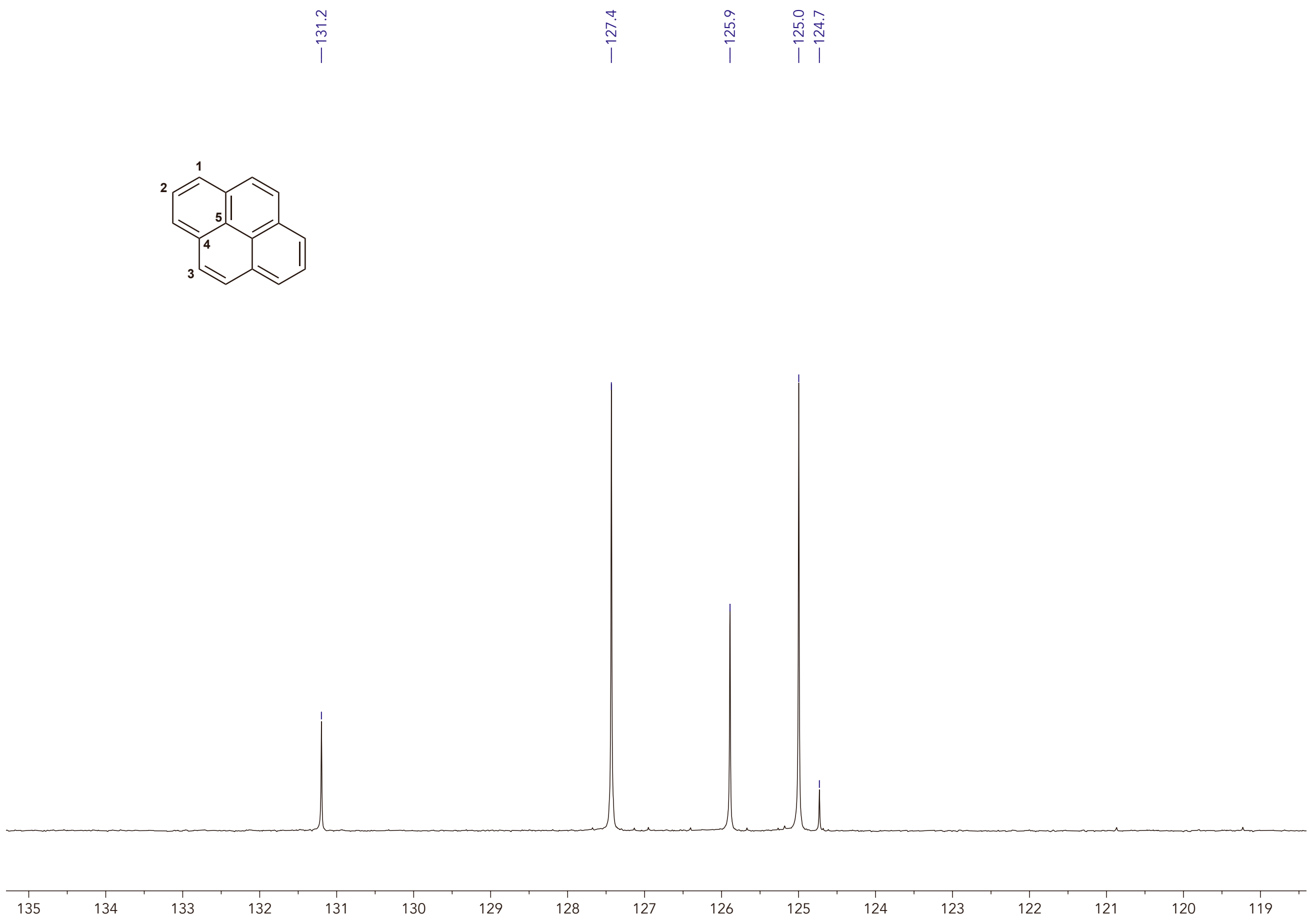


**Figure S9.** $^{13}$C NMR spectrum of **a4** (100 MHz, $CDCl_3$, 298 K).

BODIPYs **b1**, **b2**, and **b4** were synthesized as reported previously.[2] BODIPY **b3** was synthesized analogously to a previous synthesis of **b1**,[3] but using 2,3,4-trimethyl-1*H*-pyrrole as the precursor.

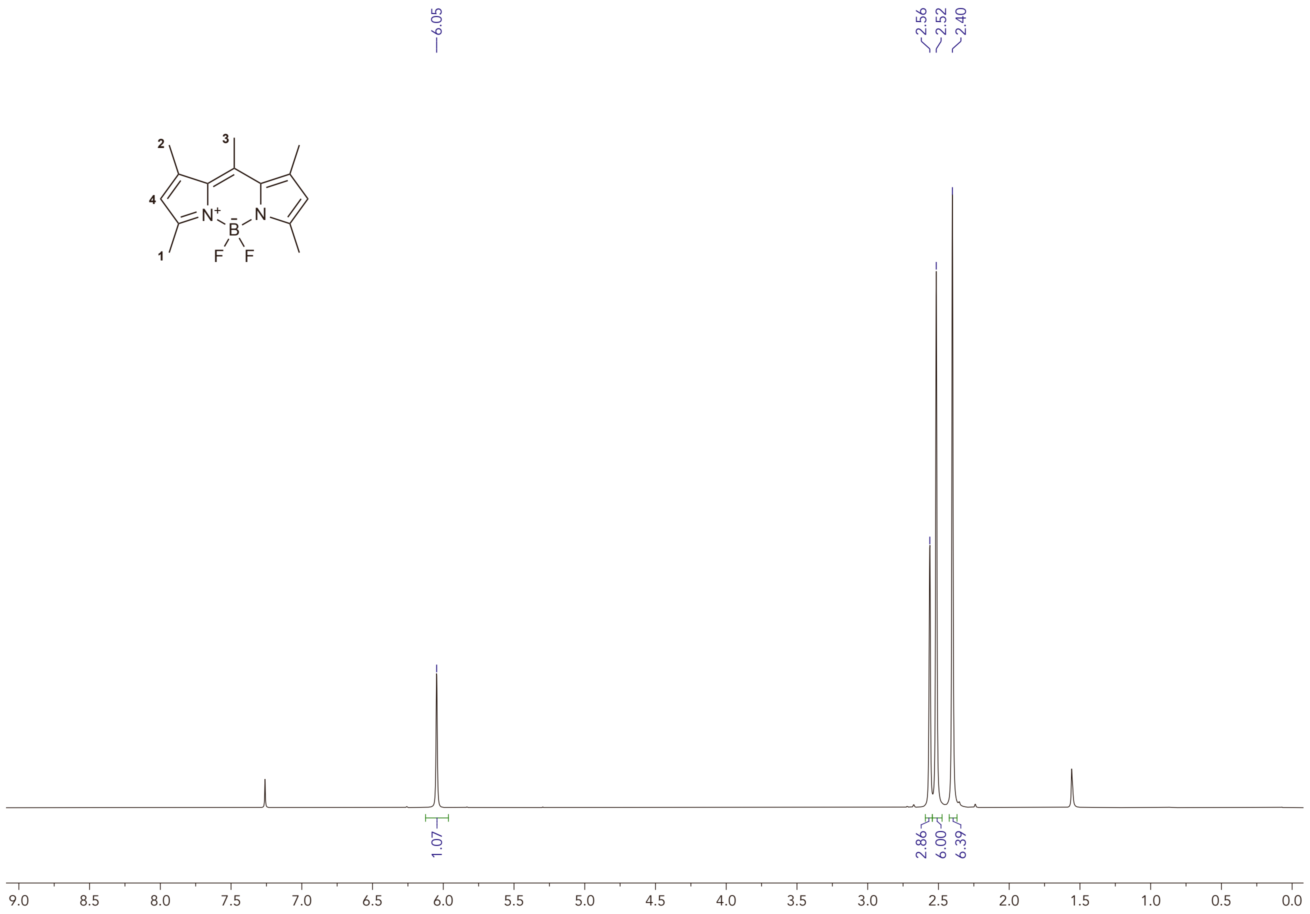


**Figure S10.** $^{1}$H NMR spectrum of **b1** (400 MHz, $CDCl_3$, 298 K).

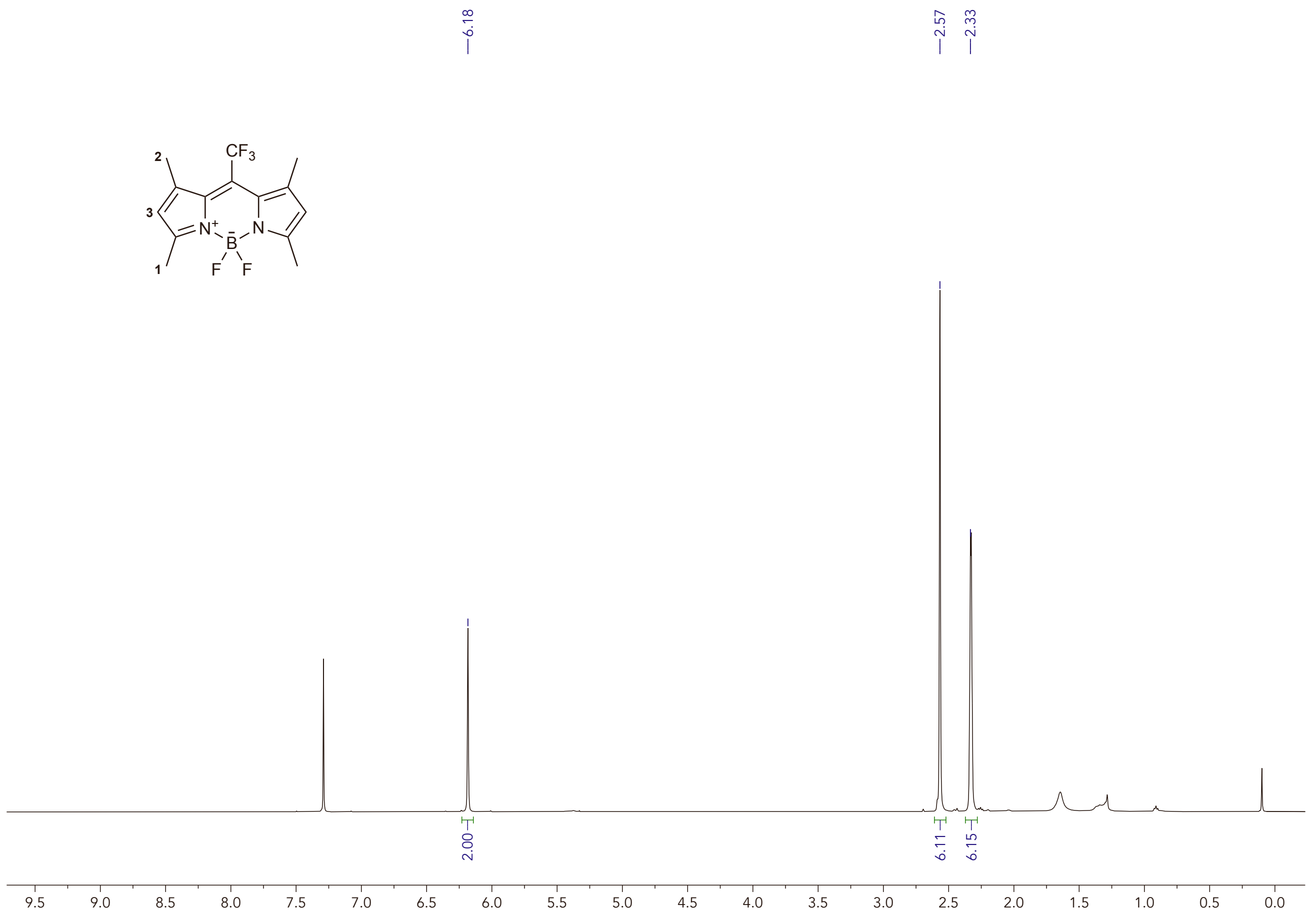


**Figure S11.** $^1$H NMR spectrum of **b2** (400 MHz, $CDCl_3$, 298 K).

**b3**:

$^{1}$H NMR (400 MHz, $CDCl_3$, 298 K): $\delta$ = 2.59 (s, 3H, **b3**$_3$), 2.48 (s, 6H, **b3**$_1$), 2.31 (s, 6H, **b3**$_2$), 1.94 (s, 6H, **b3**$_4$).

$^{13}$C NMR (100 MHz, $CDCl_3$, 298 K): $\delta$ = 152.2 (**b3**$_5$), 139.7 (**b3**$_9$), 137.0 (**b3**$_7$), 131.7 (**b3**$_8$), 126.0 (**b3**$_6$), 17.0 (**b3**$_3$), 14.7 (**b3**$_2$), 12.7 (**b3**$_1$), 9.2 (**b3**$_4$).

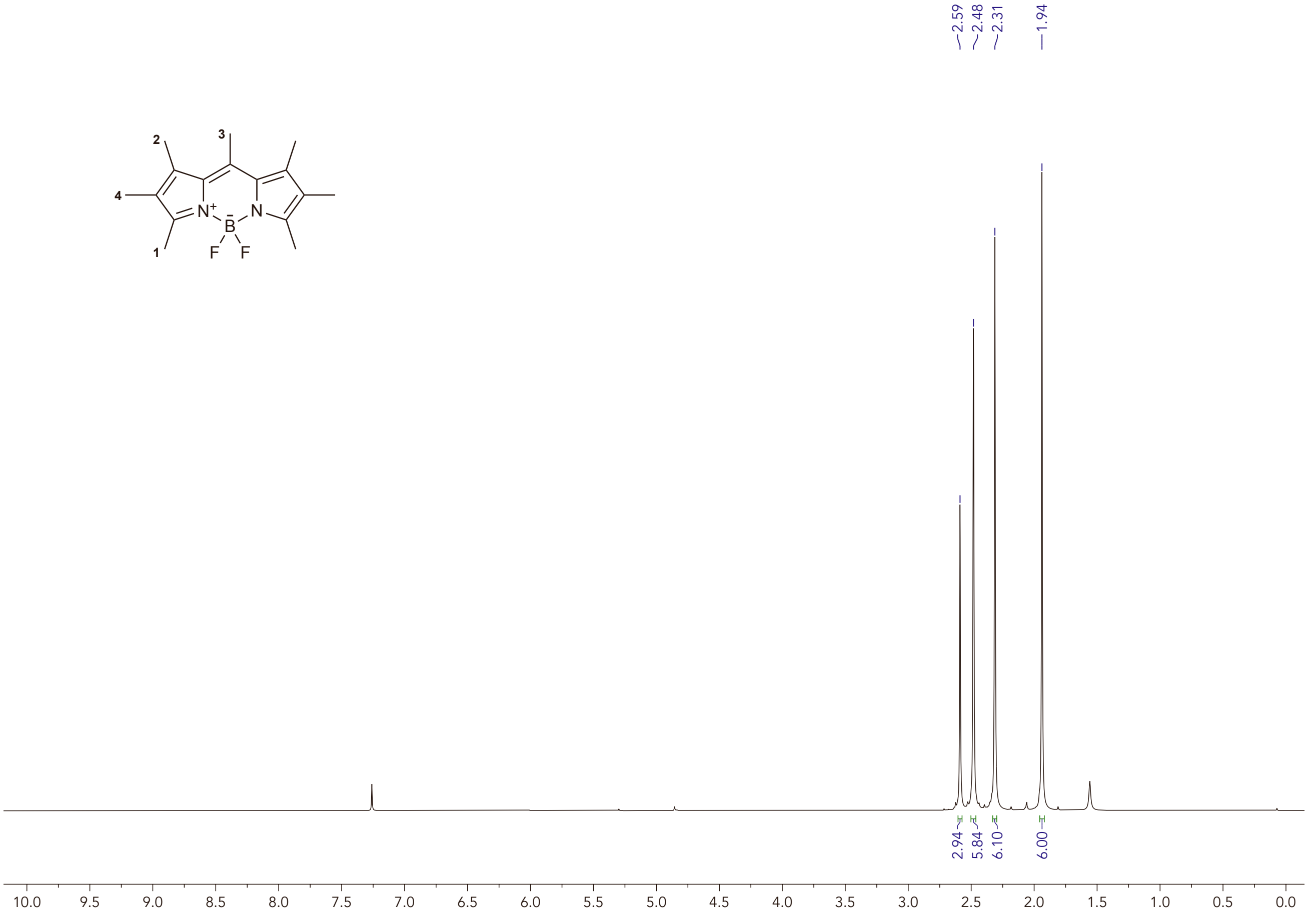


**Figure S12.** $^{1}$H NMR spectrum of **b3** (400 MHz, $CDCl_3$, 298 K).

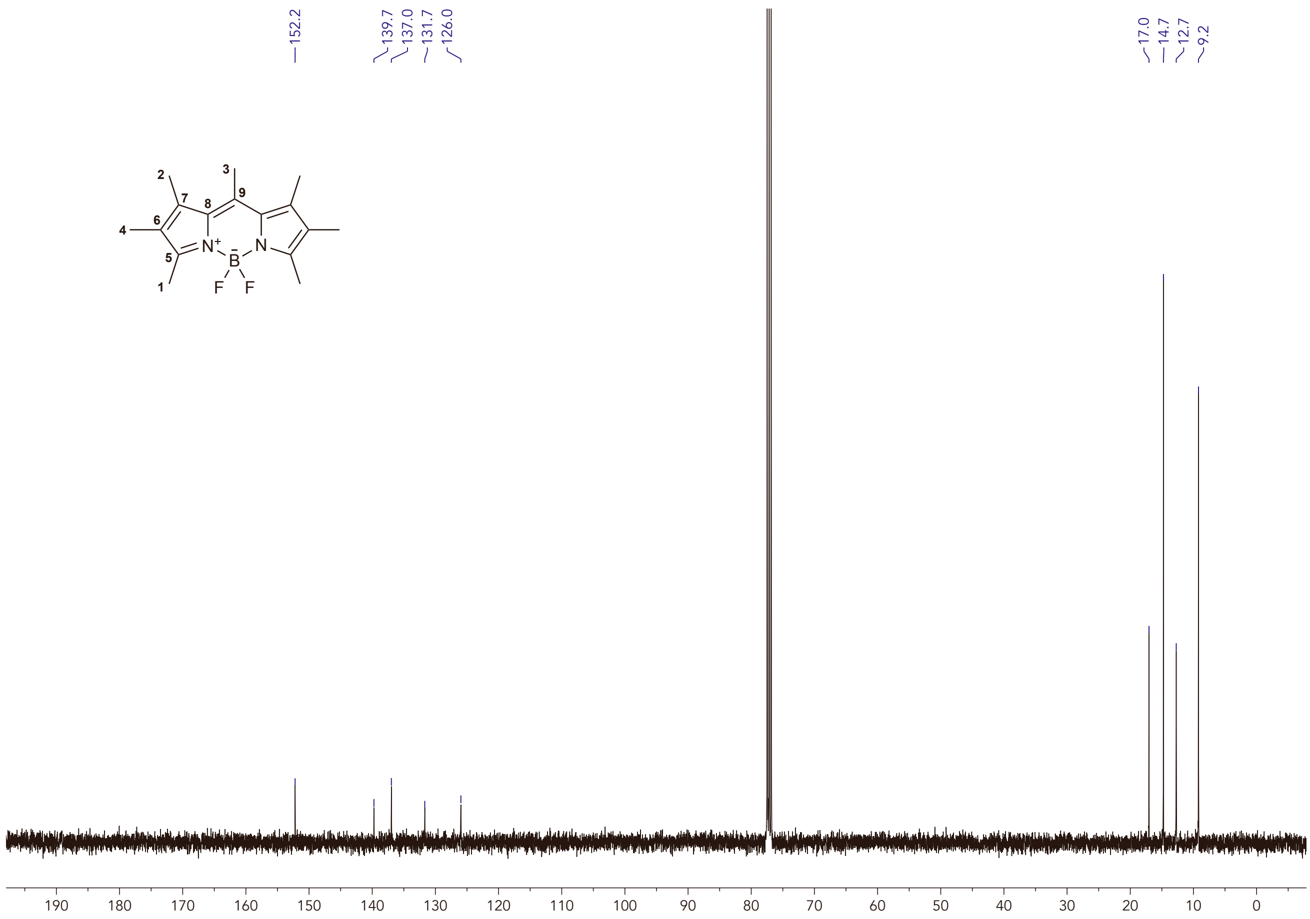


**Figure S13.** $^{13}C$ NMR spectrum of **b3** (100 MHz, $CDCl_3$, 298 K).

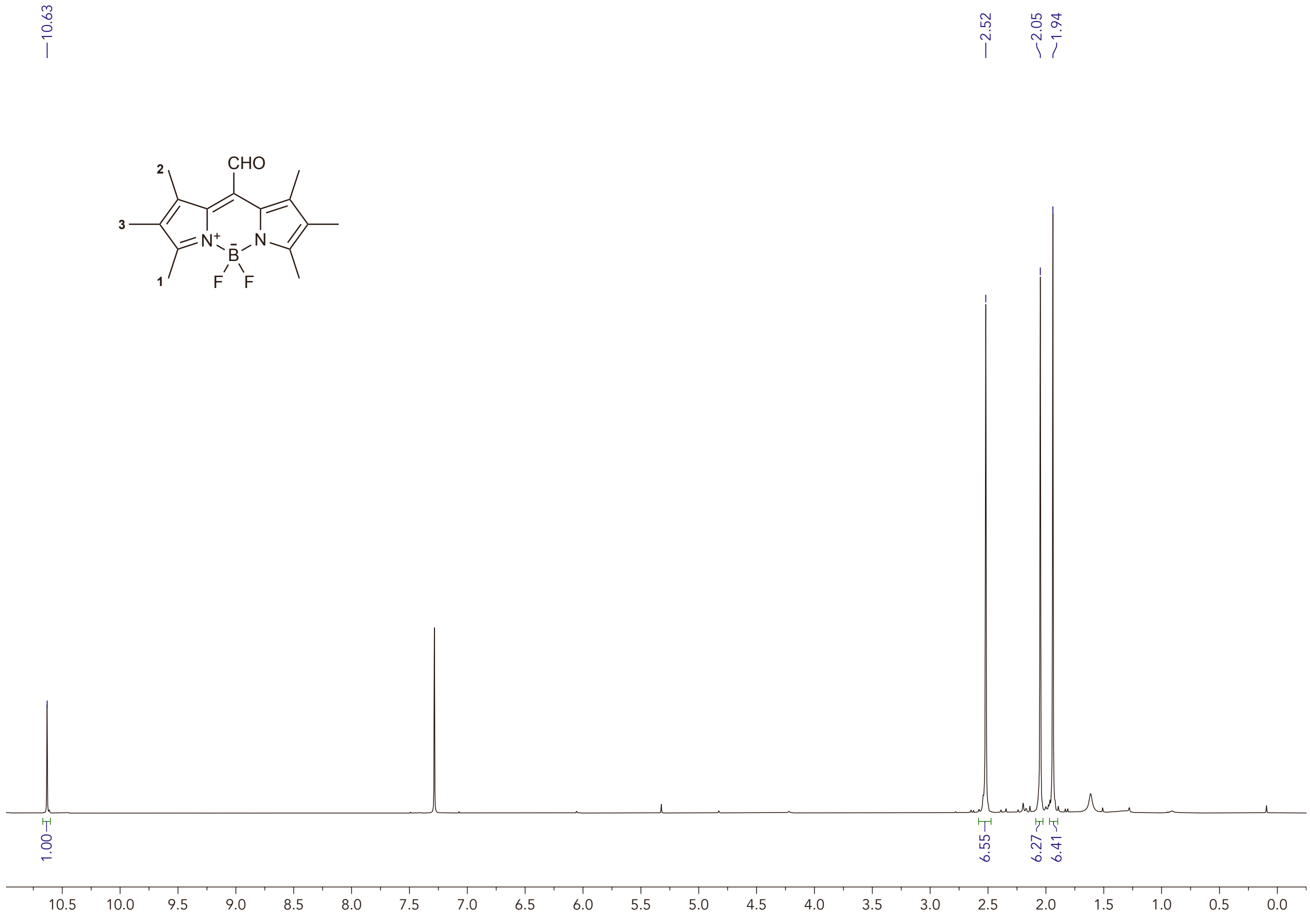


**Figure S14.** $^{1}H$ NMR spectrum of **b4** (400 MHz, $CDCl_3$, 298 K).

## 3. Following the formation of homodimeric complexes

The uptake of guests **a1**, **a2**, and **a4** by the cage over time was monitored by UV-vis absorption spectroscopy. To this end, aliquots from the suspensions were taken at various times, subjected to repeated centrifugations, and analyzed by UV-vis absorption spectroscopy. Representative UV-vis spectra are shown in Figure S15. Based on these results, we concluded that the uptake of all hydrocarbons is largely complete within <10 h. Similar results were obtained for **a3** (not shown). The uptake of BODIPYs was monitored as described previously.[2]

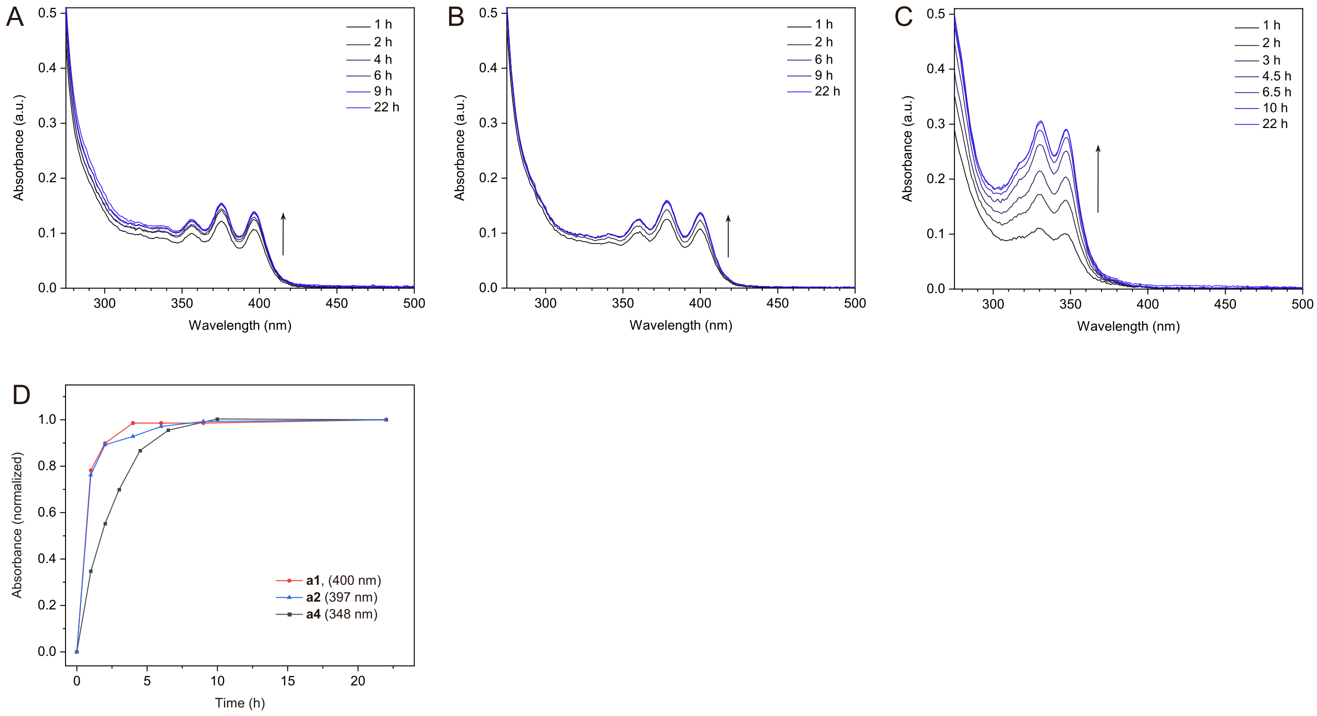


**Figure S15.** (A–C) Uptake of **a1** (A), **a2** (B), and **a4** (C) by cage **C** followed by UV-vis absorption spectroscopy. (D) Normalized profiles of the uptake of **a1**, **a2**, and **a4** by cage **C**. Absorbance was followed at 400 nm, 397 nm, and 348 nm for **a1**, **a2**, and **a4**, respectively.

## 4. NMR characterization of BODIPY homodimers

Complexes $(\mathbf{b1})_2\subset\mathbf{C}$, $(\mathbf{b2})_2\subset\mathbf{C}$, $(\mathbf{b3})_2\subset\mathbf{C}$, and $(\mathbf{b4})_2\subset\mathbf{C}$ were obtained as described in Section 3. Note that the yield of each complex (i.e., molar fraction of cages that became filled with the guest) depended strongly on the substitution pattern on BODIPY. Specifically, $(\mathbf{b1})_2\subset\mathbf{C}$ was obtained with a ~50% yield (i.e., approximately half of the cages remained empty), $(\mathbf{b2})_2\subset\mathbf{C}$ was obtained with a ~62% yield, and $(\mathbf{b4})_2\subset\mathbf{C}$ was obtained in a near-quantitative yield. The characterization of all three complexes was reported previously.[2]

For $(\mathbf{b3})_2\subset\mathbf{C}$, the encapsulation yield was found to be only ~10% (as determined by NMR; see Figure S16), despite prolonged stirring in the presence of excess of free guest **b3**. The NMR spectrum of the reaction mixture is shown in Figure S16, whereby the high-intensity peaks (e.g., the characteristic peak due to the acidic equatorial imidazole $\mathbf{C}_4$ at 9.10 ppm) can be assigned to empty cage **C**. The successful formation of $(\mathbf{b3})_2\subset\mathbf{C}$ is also evident from the similarity of the $^1$H NMR spectrum with that of $(\mathbf{b1})_2\subset\mathbf{C}$;[2] in both cases, the characteristic splitting of the filled cage's $\mathbf{C}_4$ proton is observed[2] (for $(\mathbf{b3})_2\subset\mathbf{C}$, into two singlets at 9.64 and 9.41 ppm). The peaks at 1.58, 0.45, and 0.30 ppm can be assigned to the methyl groups of encapsulated **b3**.

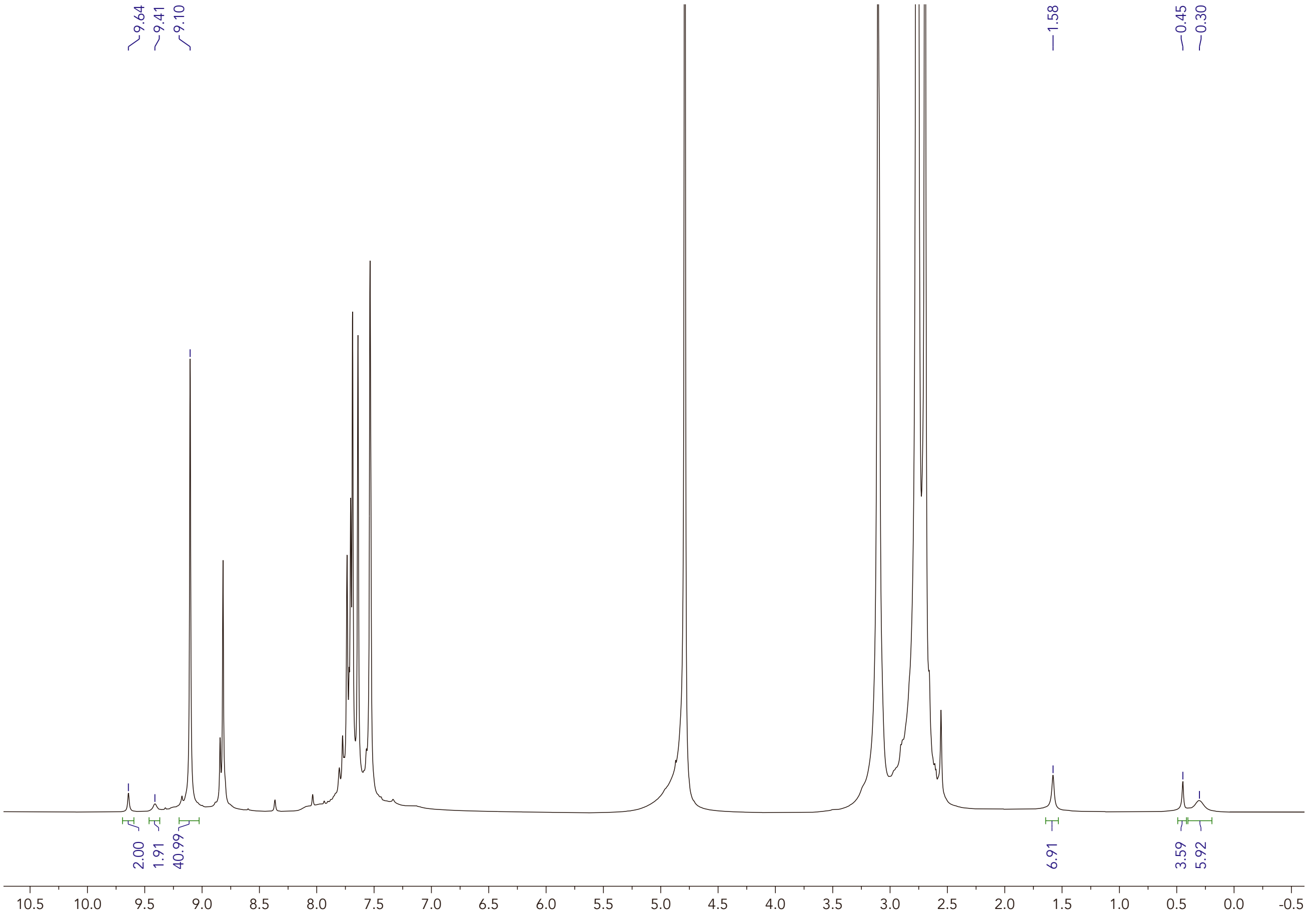


**Figure S16.** $^1$H NMR spectrum of $(\mathbf{b3})_2\subset\mathbf{C}$ (400 MHz, $CDCl_3$, 298 K).

## 5. NMR characterization of homodimers (**a1**)$_2$⊂**C**, (**a2**)$_2$⊂**C**, (**a3**)$_2$⊂**C**, and (**a4**)$_2$⊂**C**

Where significant line broadening in the room-temperature $^1$H NMR spectra was observed (see, e.g., Figure S17; compare with Figure 1A in the main text), the inclusion complexes were characterized at an elevated temperature (typically 330 K).

Inclusion complex (**a1**)$_2$⊂**C** was obtained in a near-quantitative yield, as determined by $^1$H NMR spectroscopy; the spectrum at 330 K is shown in Figure 1A (main text).

$^1$H NMR (600 MHz, $D_2O$, 330 K): $\delta$ = 9.43 (s, 4H, **C$_1$**), 8.93 (s, 8H, **C$_4$**), 7.84 (s, 4H, **C$_3$**), 7.83 (s, 4H, **C$_2$**), 7.56 (s, 8H, **C$_5$**), 7.39 (s, 8H, **C$_7$**), 7.19 (s, 4H, **C$_8$**), 7.15 (s, 8H, **C$_6$**), 6.77 (t, 8H, **a1$_3$**), 6.43 (s, 6H, **a1$_{4+5}$**), 6.32 (br, 4H, **a1$_2$**), 6.06 (s, 4H, **a1$_1$**), 3.16 (s, 8H, **C$_9$**), 3.03 (s, 16H, **C$_9$**), 2.85–2.50 (m, 72H, **C$_{10}$**), 0.16 (s, 6H, **a1**$_{CH_3}$).

$^{13}$C NMR (150 MHz, $D_2O$, 330 K): $\delta$ = 138.0 (**C$_q$**), 137.3 (**C$_4$**), 137.2 (**C$_{q'}$**), 137.0 (**C$_1$**), 130.2 (**C$_2$**), 130.0 (**a1$_8$**), 129.3 (**C$_5$**), 128.2 (**a1$_7$**), 128.1 (**a1$_6$**), 127.0 (**a1$_4$**), 125.5 (**a1$_3$**), 125.0 (**a1$_2$**), 123.1 (**a1$_1$**), 122.6 (**a1$_5$**), 121.0 (**C$_3$**), 120.6 (**C$_6$**), 111.9 (**C$_8$**), 111.1 (**C$_7$**), 63.2 (**C$_9$**), 63.0 (**C$_9$**), 50.8 (**C$_{10}$**), 50.7 (**C$_{10}$**), 50.4 (**C$_{10}$**), 11.0 (**a1**$_{CH_3}$). (Note: **C$_q$** and **C$_{q'}$** denote H-free C atoms connected to the axial and equatorial imidazoles, respectively.)

$^1$H DOSY NMR (500 MHz, $D_2O$, 298 K): $D = 0.14\cdot10^{-5}$ cm$^2$/s.

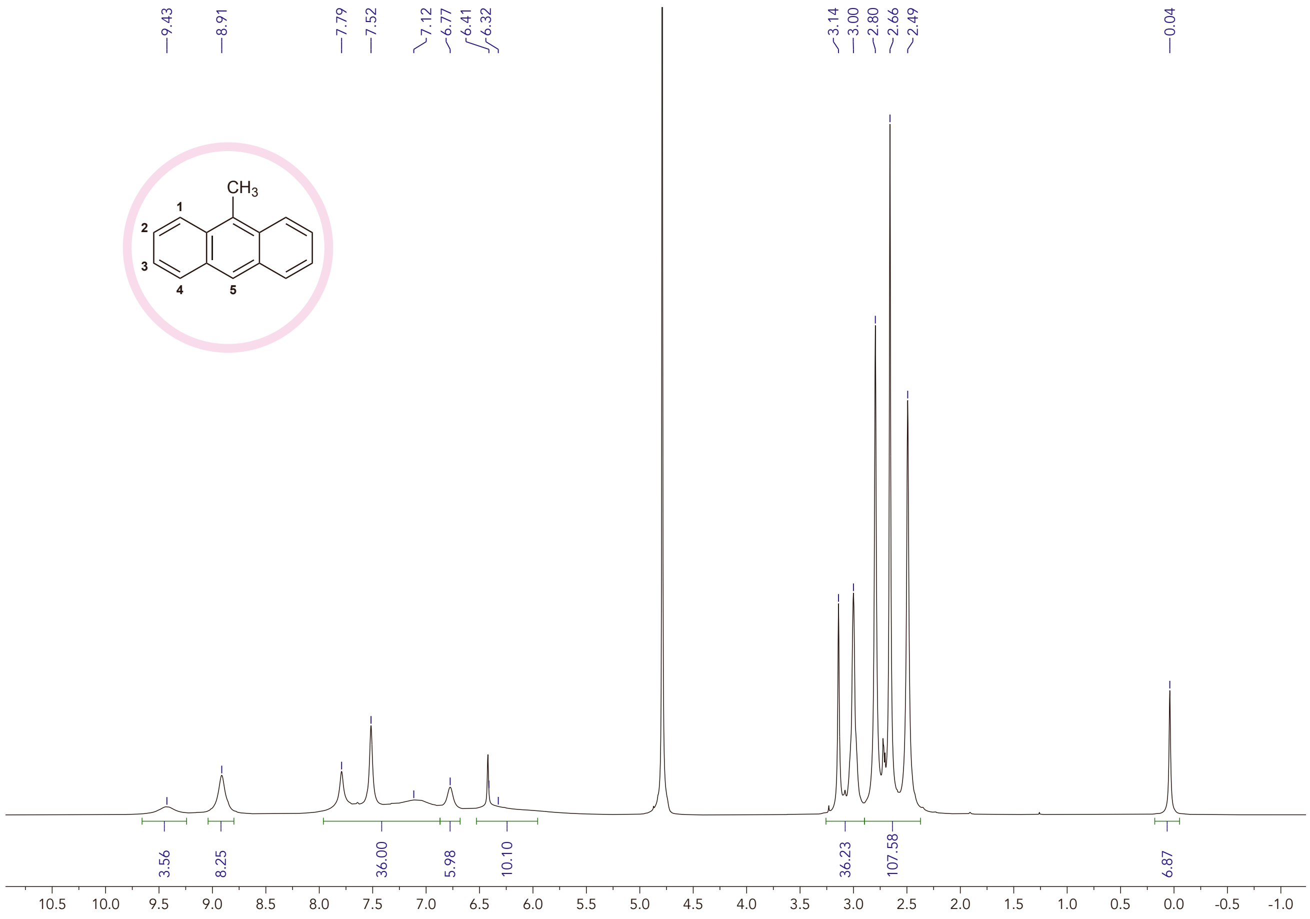


**Figure S17.** $^1$H NMR spectrum of (**a1**)$_2$⊂**C** (500 MHz, $D_2O$, 298 K; for a spectrum at an elevated temperature, see Figure 1A in the main text).

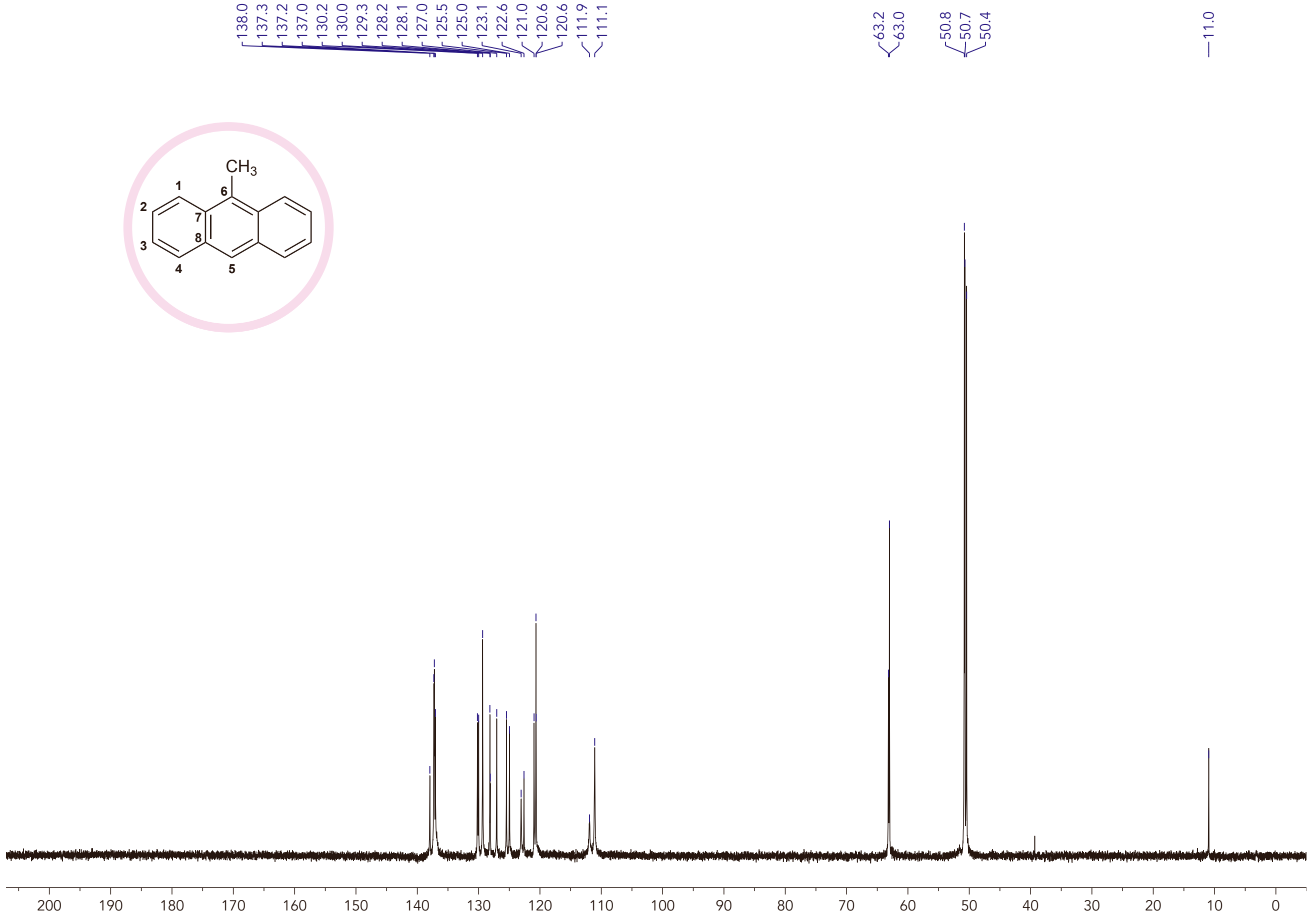


**Figure S18.** $^{13}$C NMR spectrum of (**a1**)$_2$⊂**C** (150 MHz, $D_2O$, 330 K).

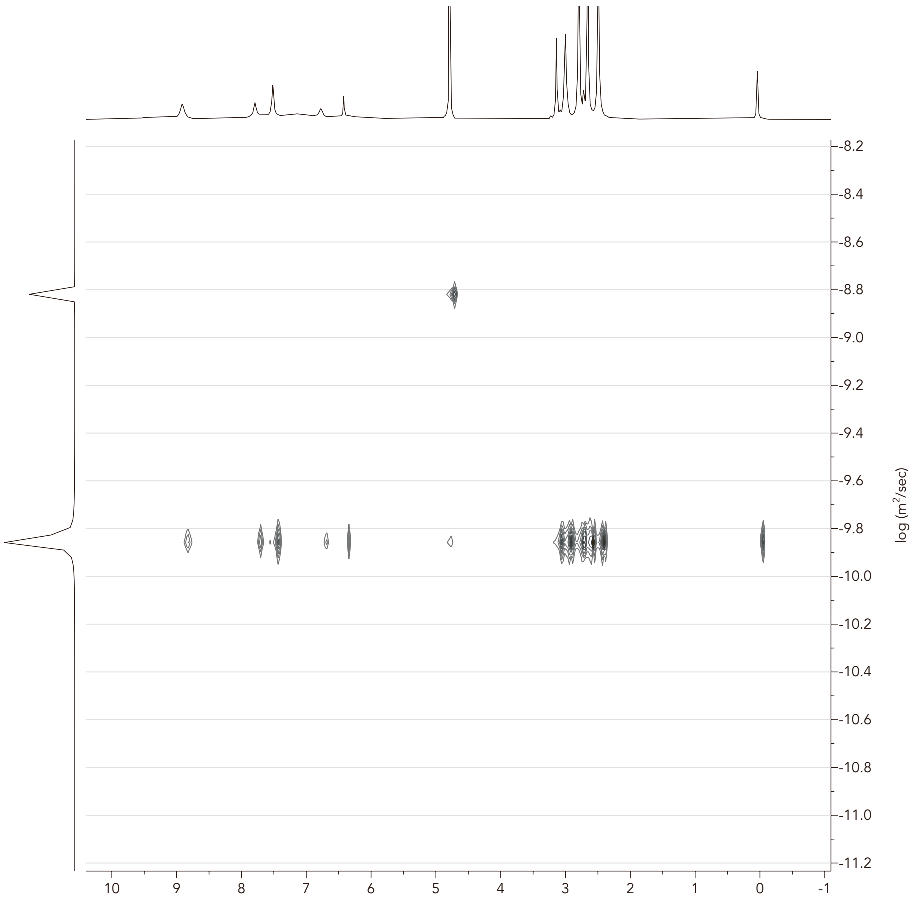


**Figure S19.** $^1$H DOSY NMR spectrum of (**a1**)$_2$⊂**C** (500 MHz, $D_2O$, 298 K).

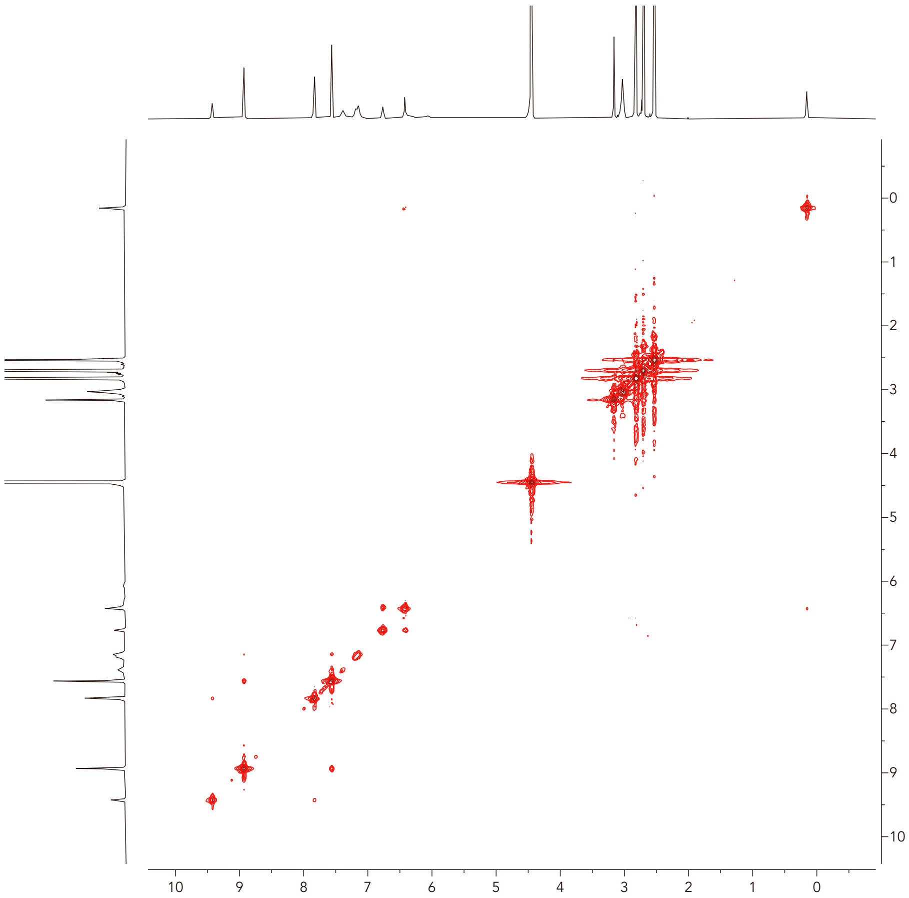


**Figure S20.** $^1H$–$^1H$ COSY NMR spectrum of (**a1**)$_2$⊂**C** (600 MHz, $D_2O$, 330 K).

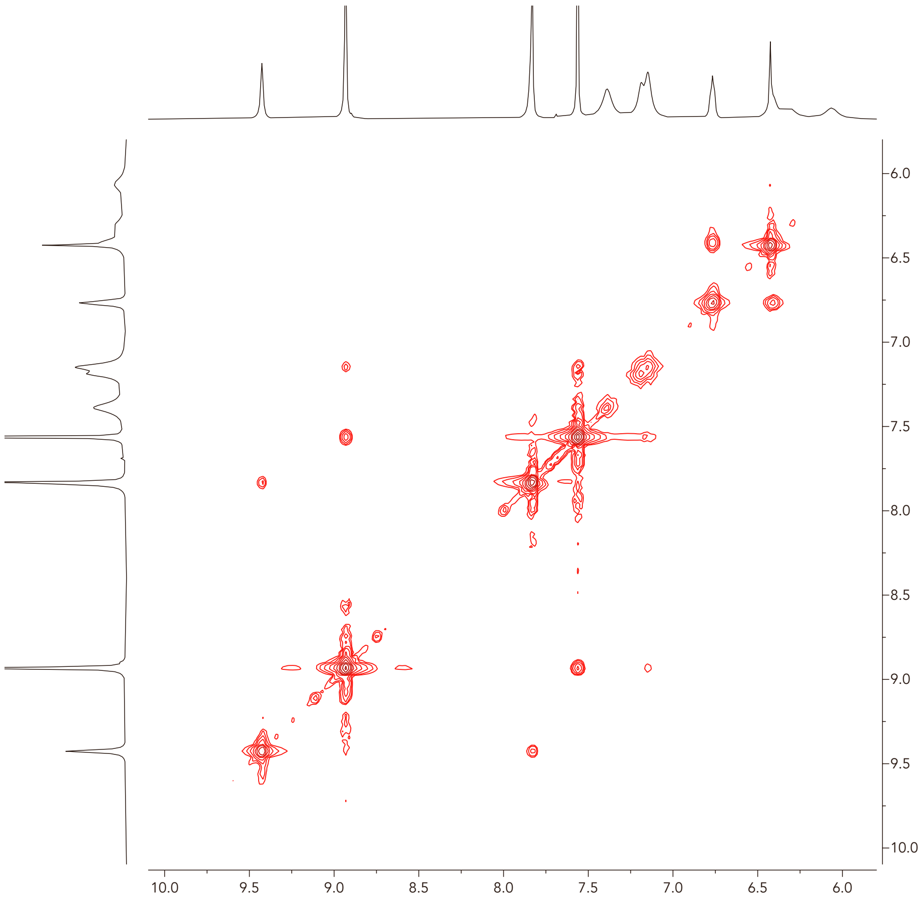


**Figure S21.** Partial $^{1}H$–$^{1}H$ COSY NMR spectrum of $(\mathbf{a1})_2\subset\mathbf{C}$ (600 MHz, $D_2O$, 330 K).

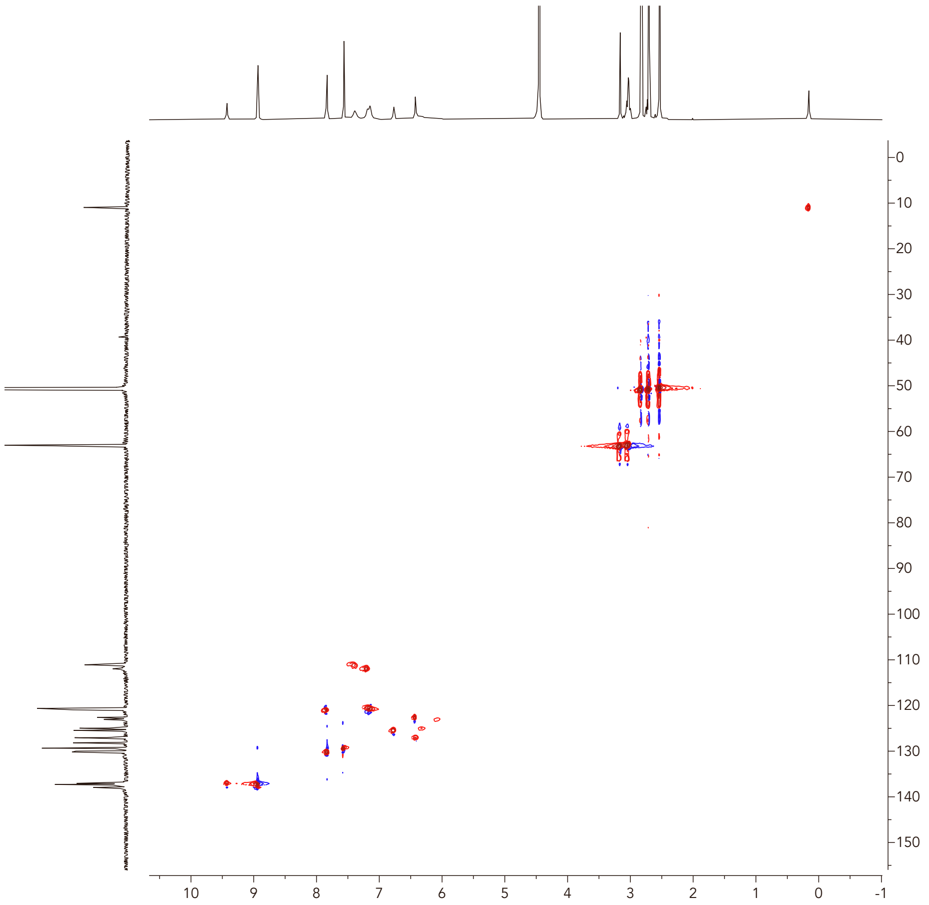


**Figure S22.** $^1$H–$^{13}$C HSQC NMR spectrum of (**a1**)$_2$⊂**C** (600 MHz, $D_2O$, 330 K).

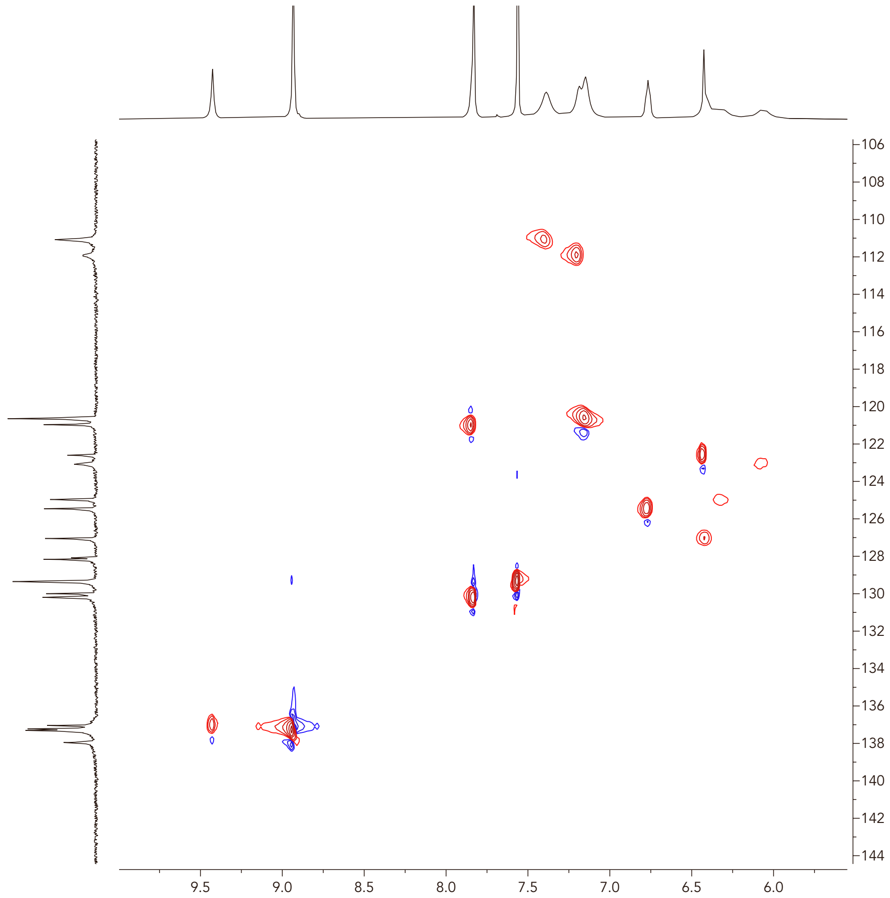


**Figure S23.** Partial $^{1}$H–$^{13}$C HSQC NMR spectrum of (**a1**)$_2$⊂**C** (600 MHz, $D_2O$, 330 K).

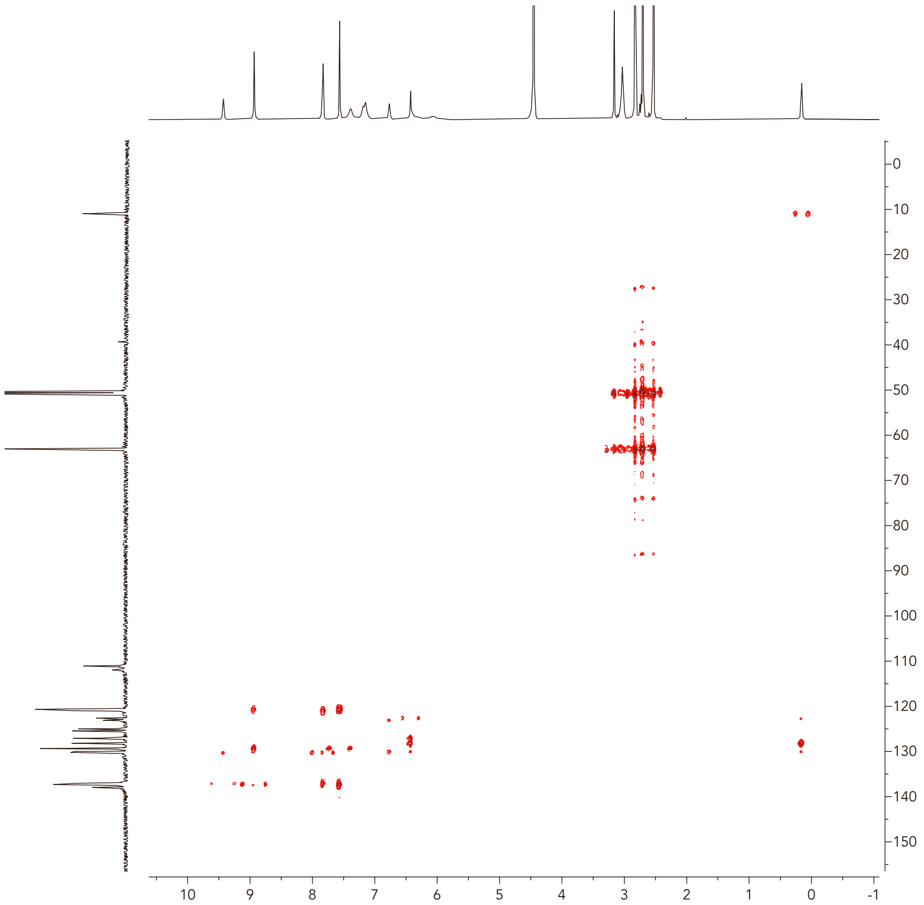


**Figure S24.** $^{1}H$–$^{13}C$ HMBC NMR spectrum of (**a1**)$_2$⊂**C** (600 MHz, $D_2O$, 330 K).

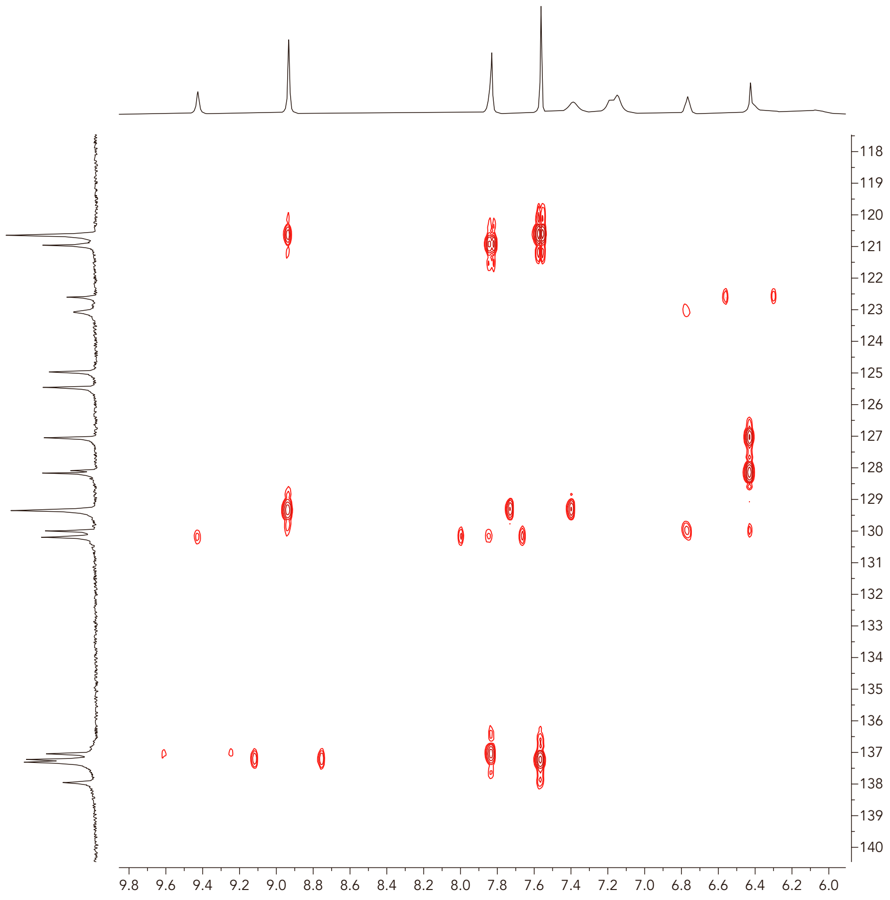


**Figure S25.** Partial $^{1}$H–$^{13}$C HMBC NMR spectrum of (**a1**)$_2$⊂**C** (600 MHz, $D_2O$, 330 K).

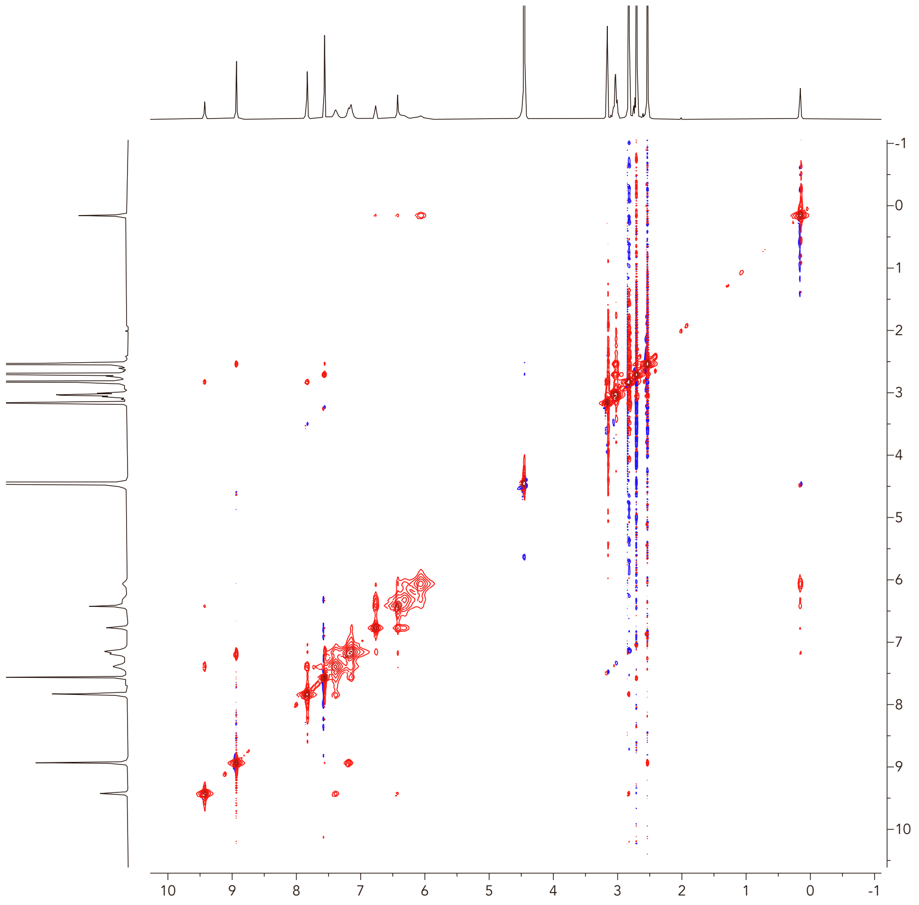


**Figure S26.** $^{1}H$–$^{1}H$ NOESY NMR spectrum of (**a1**)$_2$⊂**C** (600 MHz, $D_2O$, 330 K).

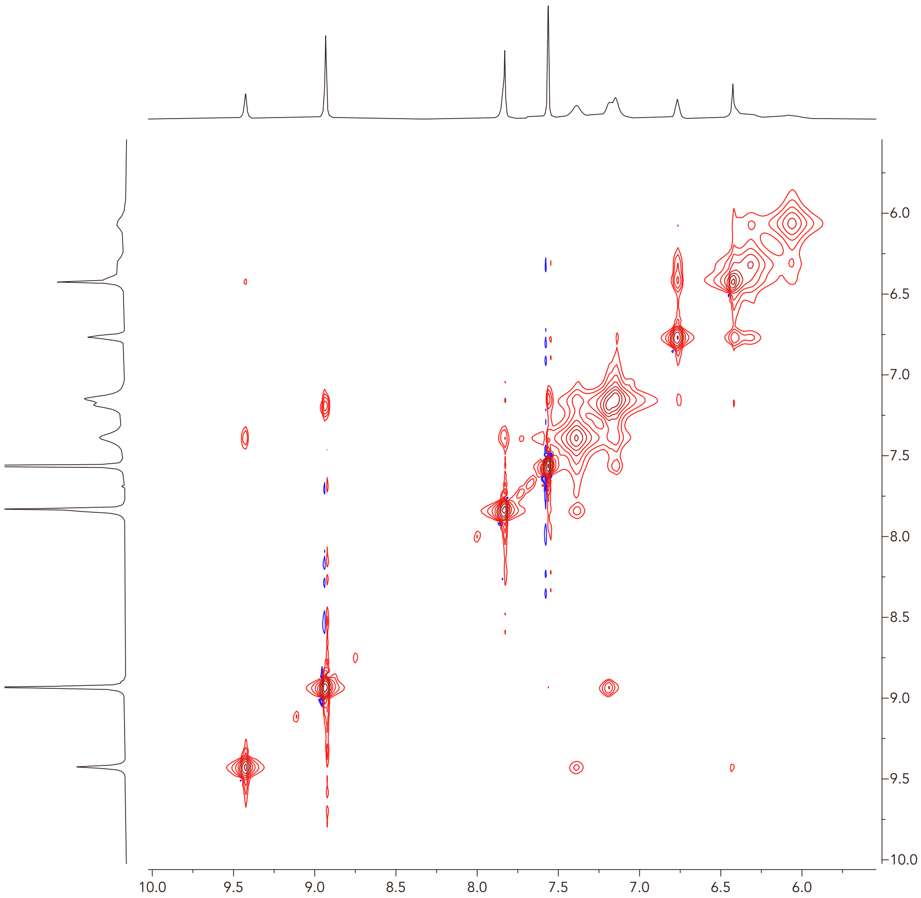


**Figure S27.** Partial $^{1}H$–$^{1}H$ NOESY NMR spectrum of (**a1**)$_2$⊂**C** (600 MHz, $D_2O$, 330 K).

Inclusion complex ($\mathbf{a2}$)$_2$⊂**C** was obtained in a near-quantitative yield, as determined by $^1$H NMR spectroscopy.

$^1$H NMR (600 MHz, $D_2O$, 330 K): $\delta$ = 9.32 (s, 4H, $\mathbf{C}_{1}$), 8.98 (s, 8H, $\mathbf{C}_{4}$), 7.83 (s, 4H, $\mathbf{C}_{3}$), 7.79 (s, 4H, $\mathbf{C}_{2}$), 7.54 (s, 8H, $\mathbf{C}_{5}$), 7.45 (s, 8H, $\mathbf{C}_{7}$), 7.33 (s, 12H, $\mathbf{C}_{6+8}$), 6.56 (s, 8H, $\mathbf{a2}_{1+3}$), 6.41 (s, 4H, $\mathbf{a2}_{2}$), 6.36 (s, 2H, $\mathbf{a2}_{5}$), 6.13 (s, 4H, $\mathbf{a2}_{4}$), 3.13 (s, 8H, $\mathbf{C}_{9}$), 3.03 (s, 16H, $\mathbf{C}_{9}$), 2.82–2.47 (m, 72H, $\mathbf{C}_{10}$).

$^{13}$C NMR (150 MHz, $D_2O$, 330 K): $\delta$ = 138.1 ($\mathbf{C}_{\mathbf{q}'}$), 137.4 ($\mathbf{C}_{4}$), 137.2 ($\mathbf{C}_{1}$), 136.9 ($\mathbf{C}_{\mathbf{q}}$), 130.4 ($\mathbf{a2}_{8}$), 130.0 ($\mathbf{C}_{2}$), 129.4 ($\mathbf{C}_{5}$), 128.3 ($\mathbf{a2}_{7}$), 127.0 ($\mathbf{a2}_{3}$), 126.6 ($\mathbf{a2}_{4}$), 125.7 ($\mathbf{a2}_{1}$), 125.6 ($\mathbf{a2}_{2}$), 124.6 ($\mathbf{a2}_{5}$), 120.8 ($\mathbf{C}_{3}$), 120.6 ($\mathbf{a2}_{6}$), 120.4 ($\mathbf{C}_{6}$), 111.9 ($\mathbf{C}_{8}$), 110.8 ($\mathbf{C}_{7}$), 63.1 ($\mathbf{C}_{9}$), 63.0 ($\mathbf{C}_{9}$), 50.8 ($\mathbf{C}_{10}$), 50.7 ($\mathbf{C}_{10}$), 50.4 ($\mathbf{C}_{10}$). (Note: $\mathbf{C}_{\mathbf{q}}$ and $\mathbf{C}_{\mathbf{q}'}$ denote H-free C atoms connected to the axial and equatorial imidazoles, respectively.)

$^1$H DOSY NMR (500 MHz, $D_2O$, 298 K): $D = 0.15{\cdot}10^{-5}$ cm$^2$/s.

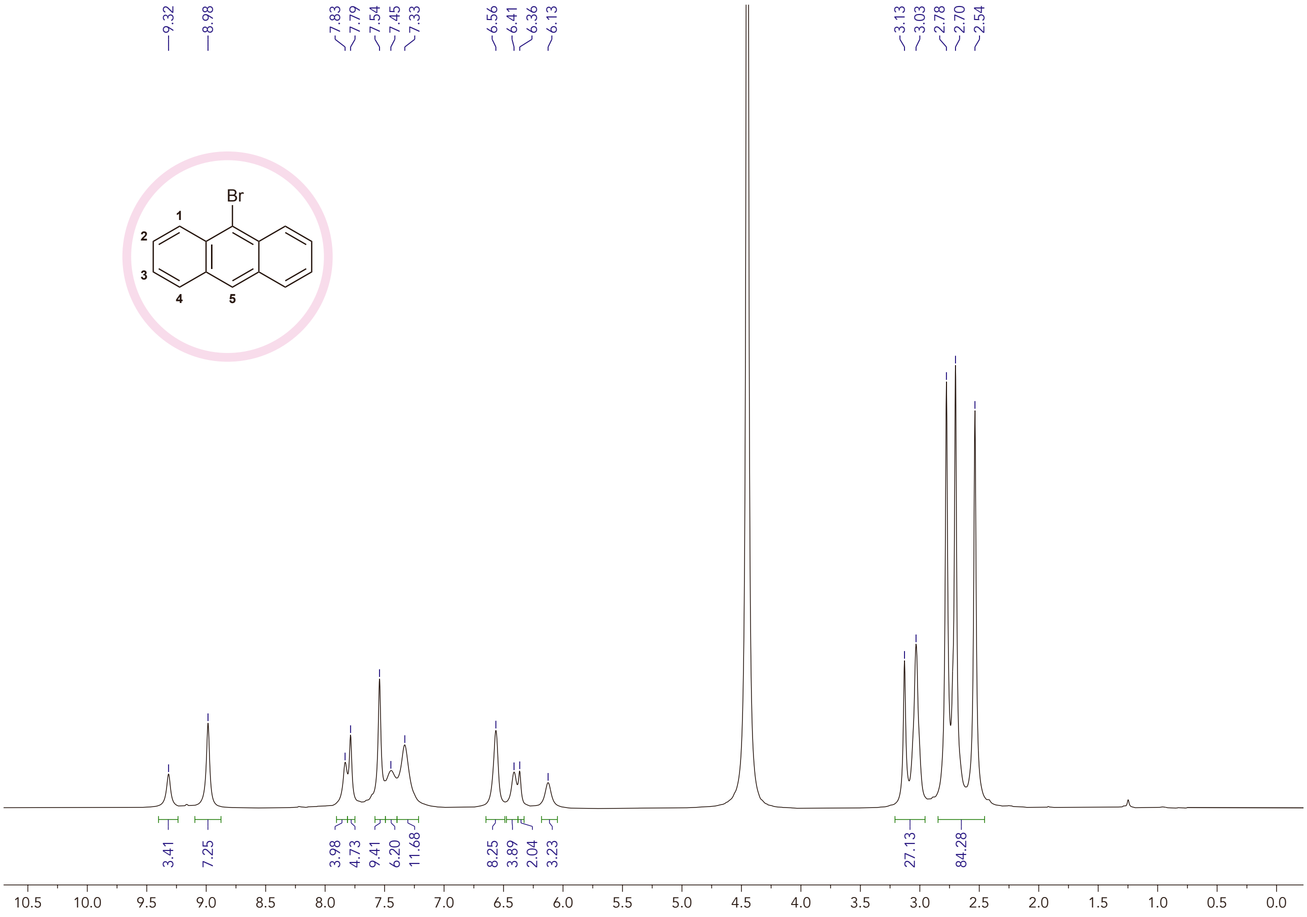


**Figure S28.** $^1$H NMR spectrum of ($\mathbf{a2}$)$_2$⊂**C** (600 MHz, $D_2O$, 330 K).

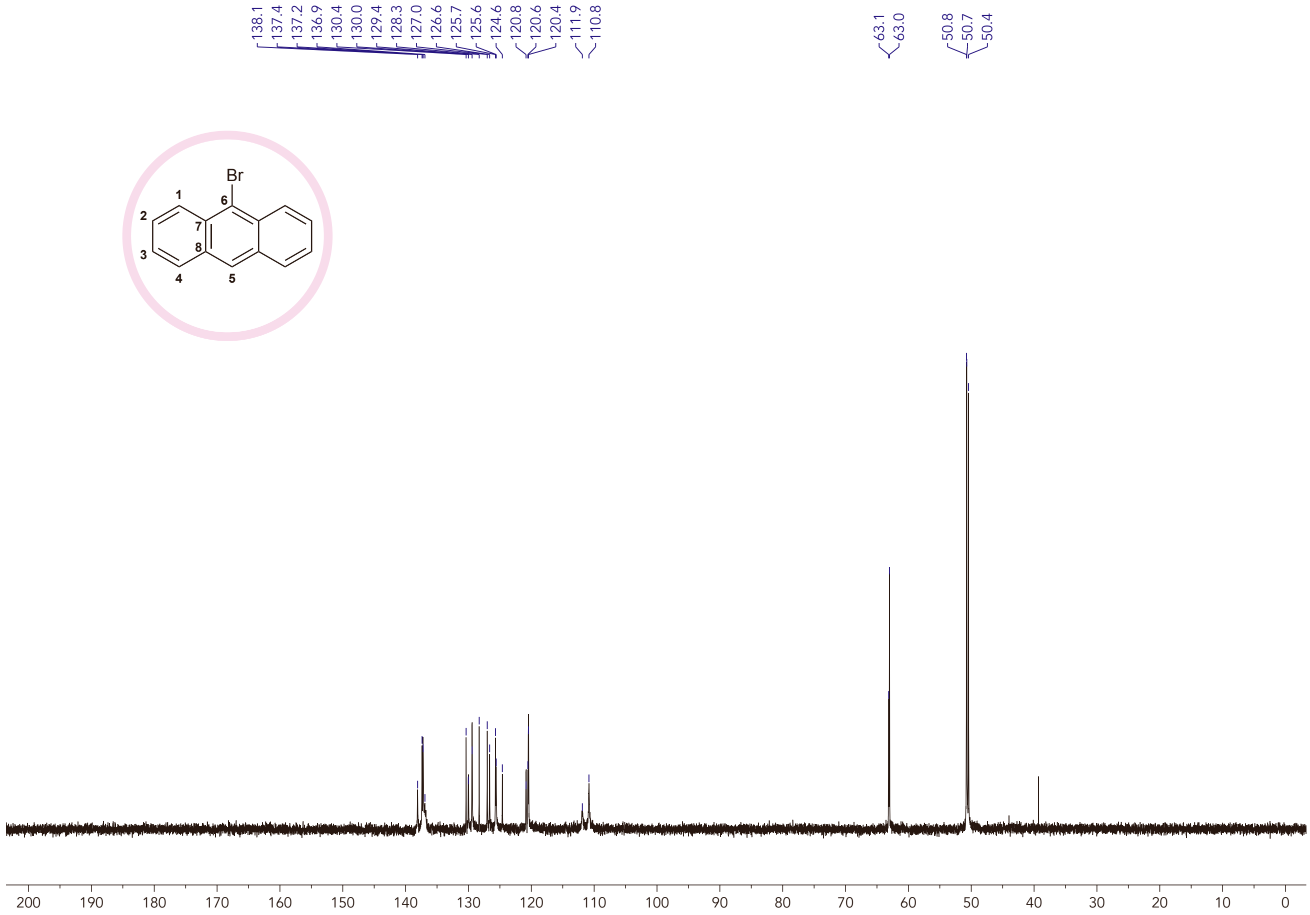


**Figure S29.** $^{13}C$ NMR spectrum of (**a2**)$_2$⊂**C** (150 MHz, $D_2O$, 330 K).

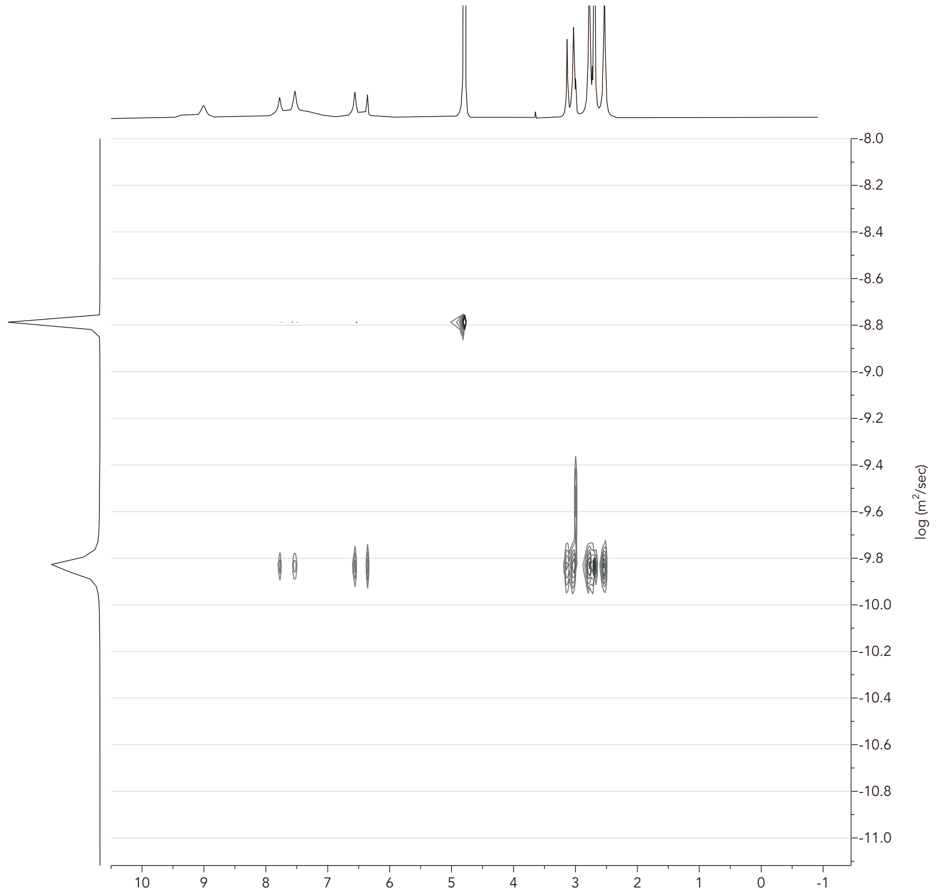


**Figure S30.** $^1$H DOSY NMR spectrum of (**a2**)$_2$⊂**C** (500 MHz, $D_2O$, 298 K).

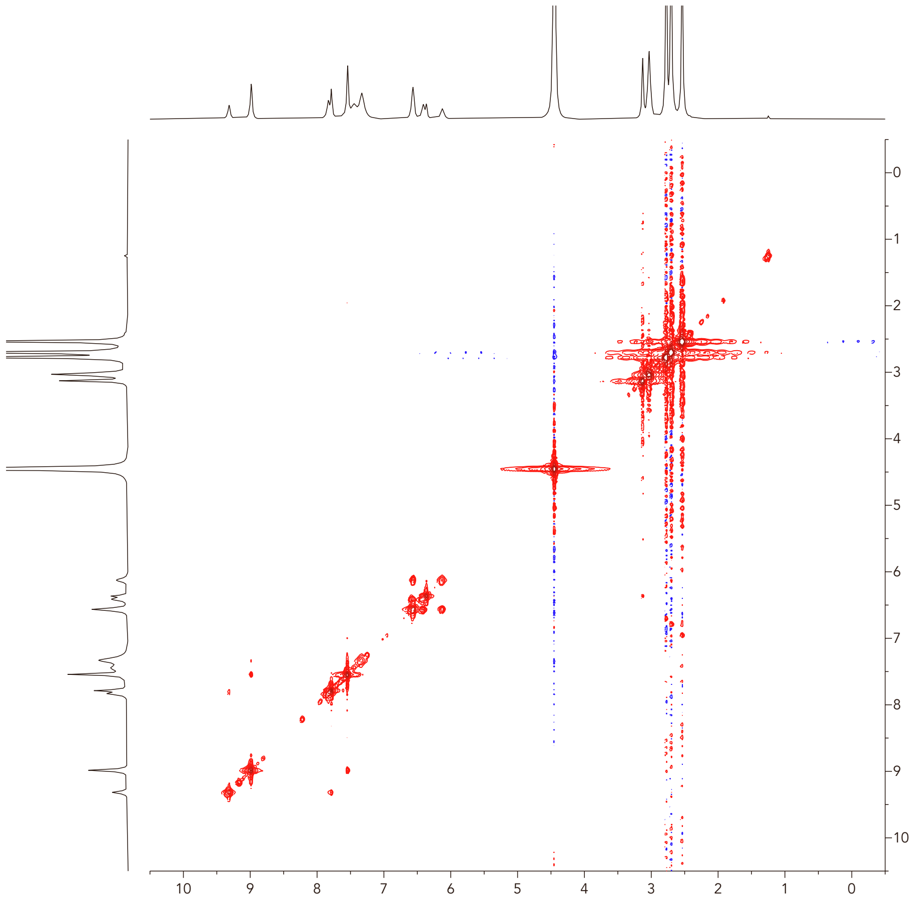


**Figure S31.** $^1H$–$^1H$ COSY NMR spectrum of (**a2**)$_2$⊂**C** (600 MHz, $D_2O$, 330 K).

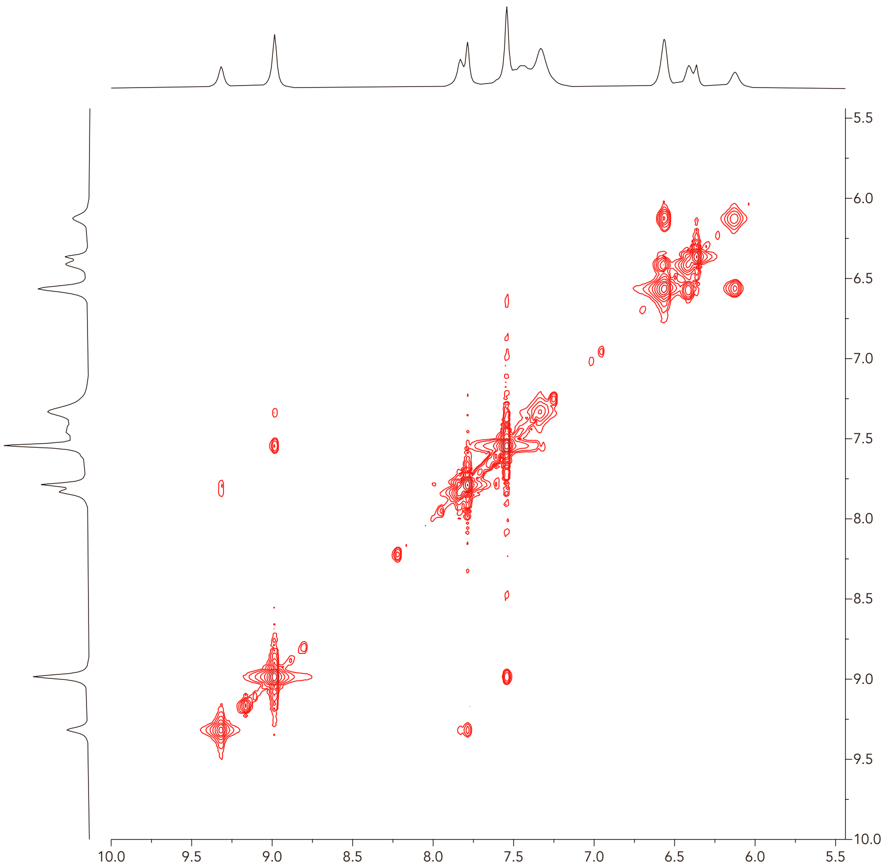


**Figure S32.** Partial $^{1}H$–$^{1}H$ COSY NMR spectrum of (**a2**)$_2$⊂**C** (600 MHz, $D_2O$, 330 K).

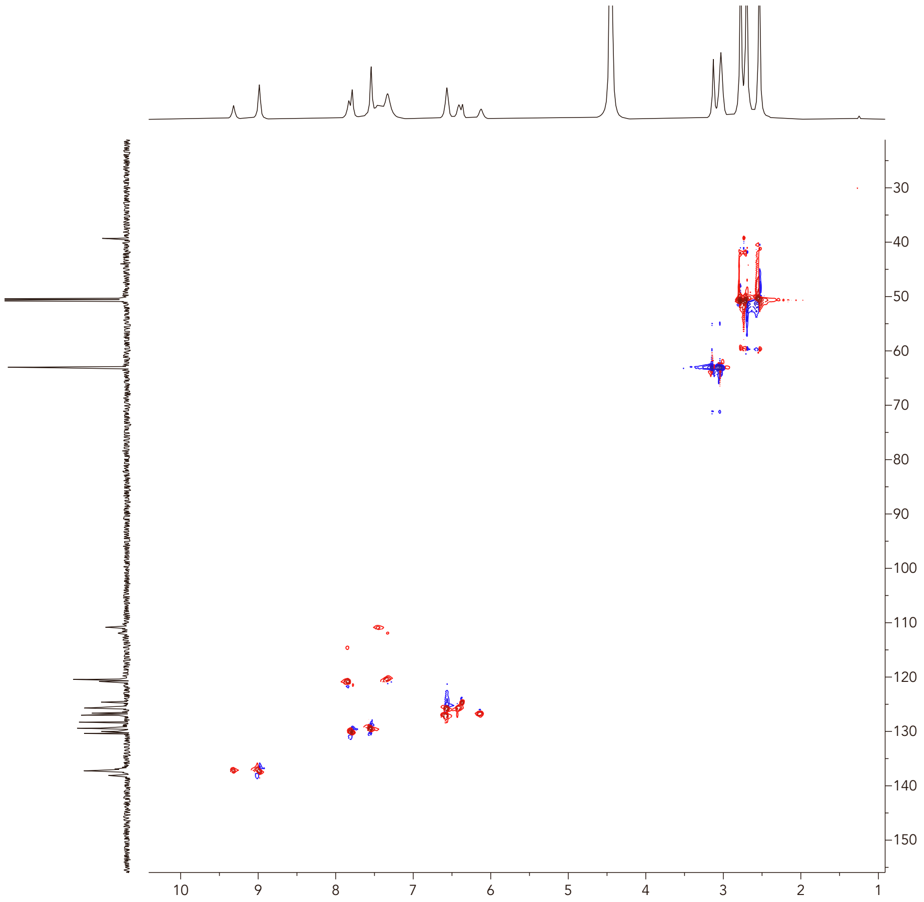


**Figure S33.** $^1H$–$^{13}C$ HSQC NMR spectrum of (**a2**)$_2$⊂**C** (600 MHz, $D_2O$, 330 K).

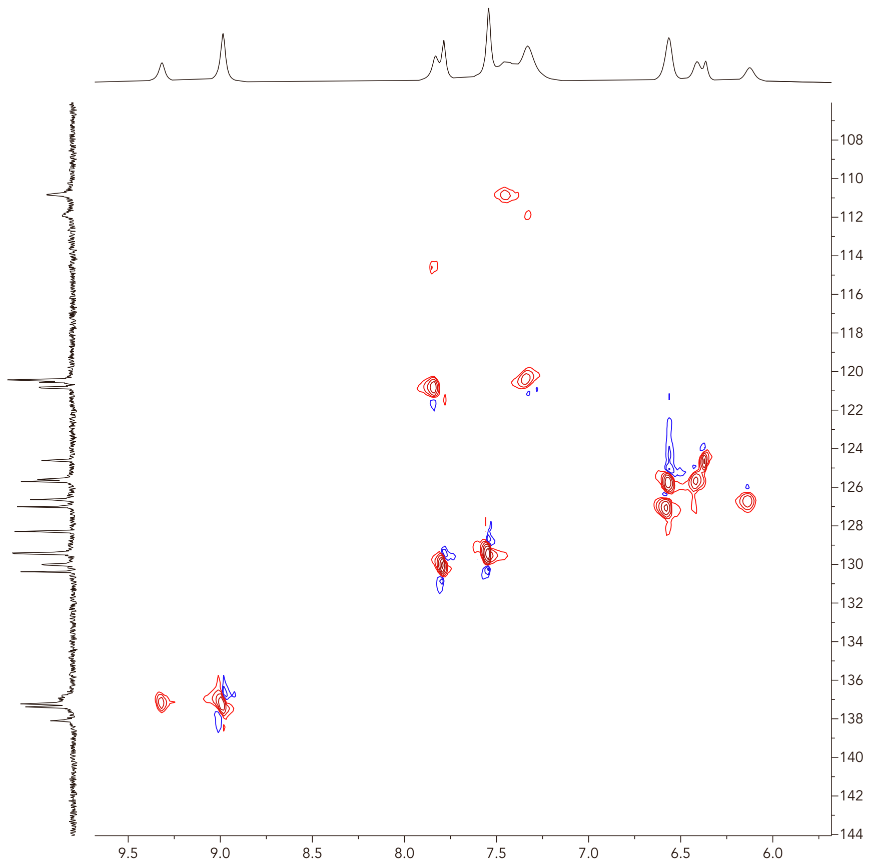


**Figure S34.** Partial $^{1}$H–$^{13}$C HSQC NMR spectrum of (**a2**)$_2$⊂**C** (600 MHz, $D_2O$, 330 K).

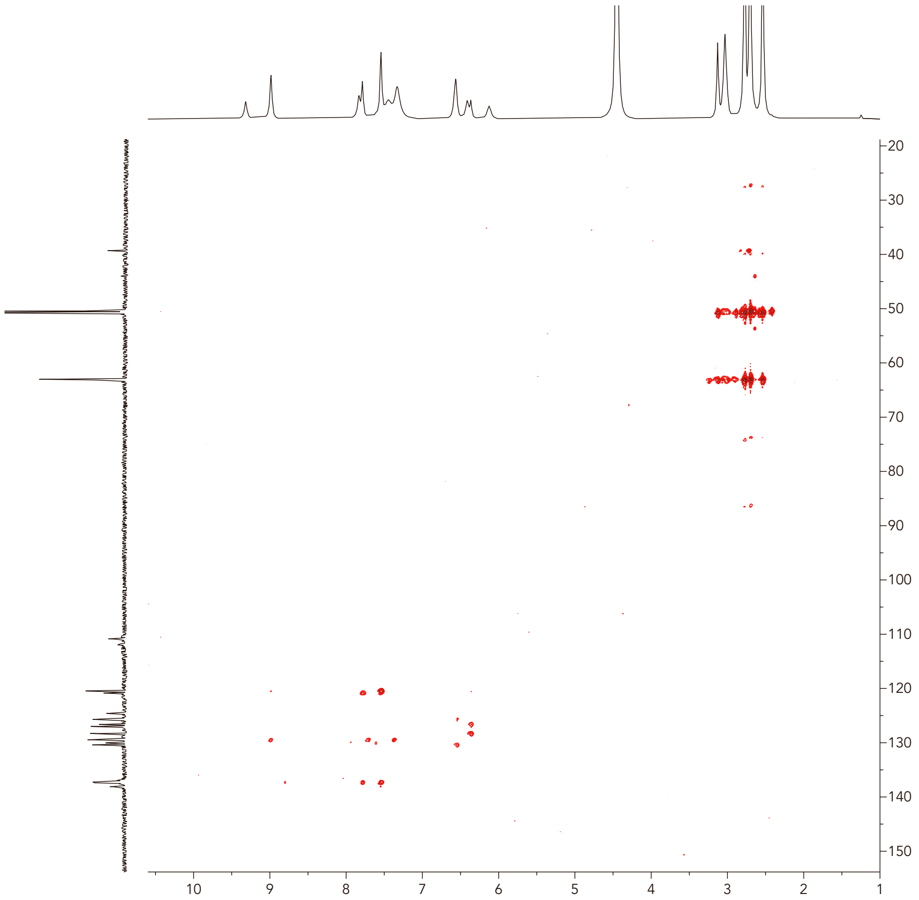


**Figure S35.** $^{1}H$–$^{13}C$ HMBC NMR spectrum of (**a2**)$_2$⊂**C** (600 MHz, $D_2O$, 330 K).

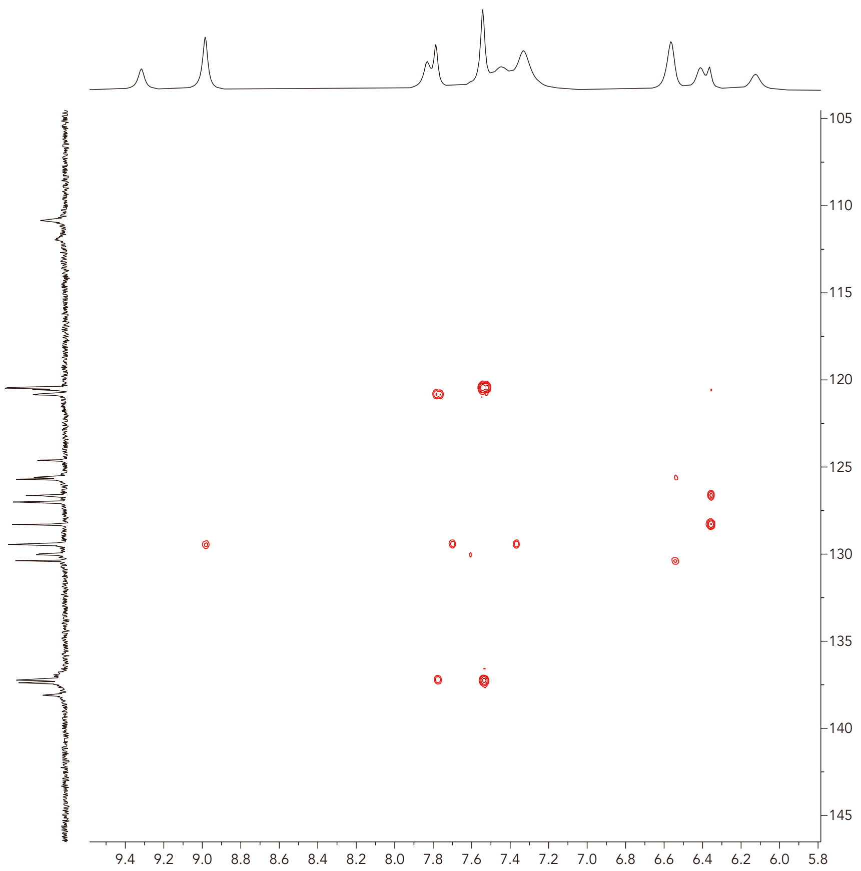


**Figure S36.** Partial $^{1}$H–$^{13}$C HMBC NMR spectrum of (**a2**)$_2$⊂**C** (600 MHz, $D_2O$, 330 K).

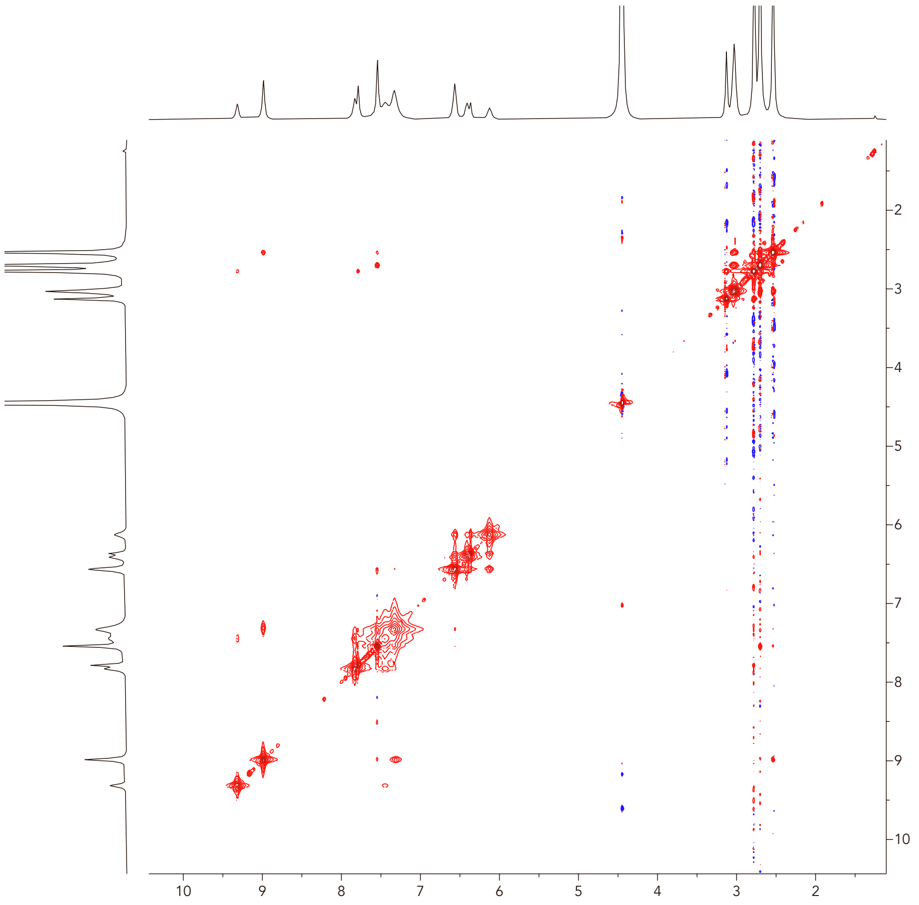


**Figure S37.** $^{1}$H–$^{1}$H NOESY NMR spectrum of (**a2**)$_2$⊂**C** (600 MHz, $D_2O$, 330 K).

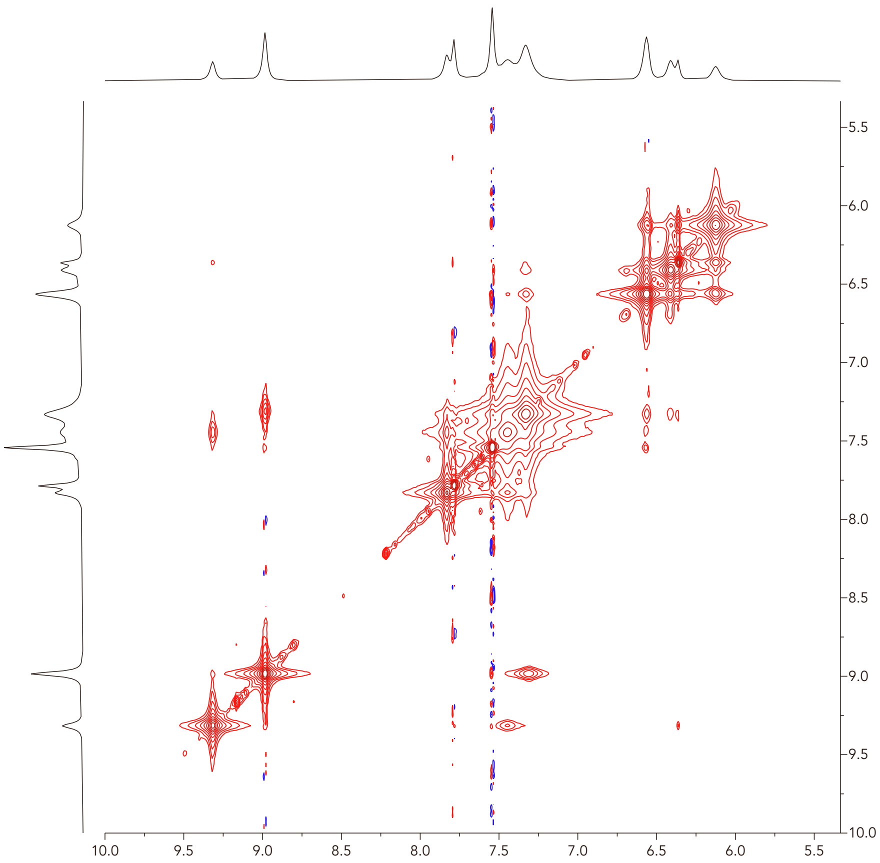


**Figure S38.** Partial $^1H$–$^1H$ NOESY NMR spectrum of (**a2**)$_2$⊂**C** (600 MHz, $D_2O$, 330 K).

Inclusion complex (**a3**)$_2$⊂**C** was obtained in a near-quantitative yield, as determined by $^1$H NMR spectroscopy.

$^1$H NMR (500 MHz, $D_2O$, 298 K): $\delta$ = 9.22 (s, 4H, **C$_1$**), 9.02 (s, 8H, **C$_4$**), 7.73 (s, 4H, **C$_3$**), 7.68 (s, 4H, **C$_2$**), 7.57 (s, 8H, **C$_5$**), 7.47 (s, 8H, **C$_7$**), 7.37 (s, 8H, **C$_6$**), 7.32 (s, 4H, **C$_8$**), 6.83 (s, 4H, **a3$_1$**), 6.45 (s, 4H, **a3$_2$**), 6.16 (s, 4H, **a3$_3$**), 6.00 (s, 2H, **a3$_5$**), 5.78 (s, 4H, **a3$_4$**), 3.64 (s, 4H, **a3**$_{CH_2}$), 3.12 (s, 8H, **C$_9$**), 3.05 (s, 16H, **C$_9$**), 2.76–2.56 (m, 72H, **C$_{10}$**).

$^{13}$C NMR (150 MHz, $D_2O$, 300 K): $\delta$ = 138.0 (**C$_{q+q'}$**), 137.1 (**C$_1$**), 137.1 (**C$_4$**), 129.3 (**a4$_7$**), 129.2 (**C$_2$**), 129.0 (**C$_5$+a4$_6$**), 127.8 (**a3$_8$**), 126.4 (**a3$_4$**), 125.2 (**a3$_2$**), 124.6 (**a3$_5$**), 124.3 (**a3$_3$**), 122.3 (**a3$_1$**), 120.5 (**C$_3$**), 120.3 (**C$_6$**), 112.5 (**C$_8$**), 111.4 (**C$_7$**), 62.6 (**C$_9$**), 62.5 (**C$_9$**), 54.4 (**a3**$_{CH_2}$), 50.3 (**C$_{10}$**), 50.0 (**C$_{10}$**). (Note: **C$_q$** and **C$_{q'}$** denote H-free C atoms connected to the axial and equatorial imidazoles, respectively.)

$^1$H DOSY NMR (500 MHz, $D_2O$, 300 K): $D = 0.18 \cdot 10^{-5}$ cm$^2$/s.

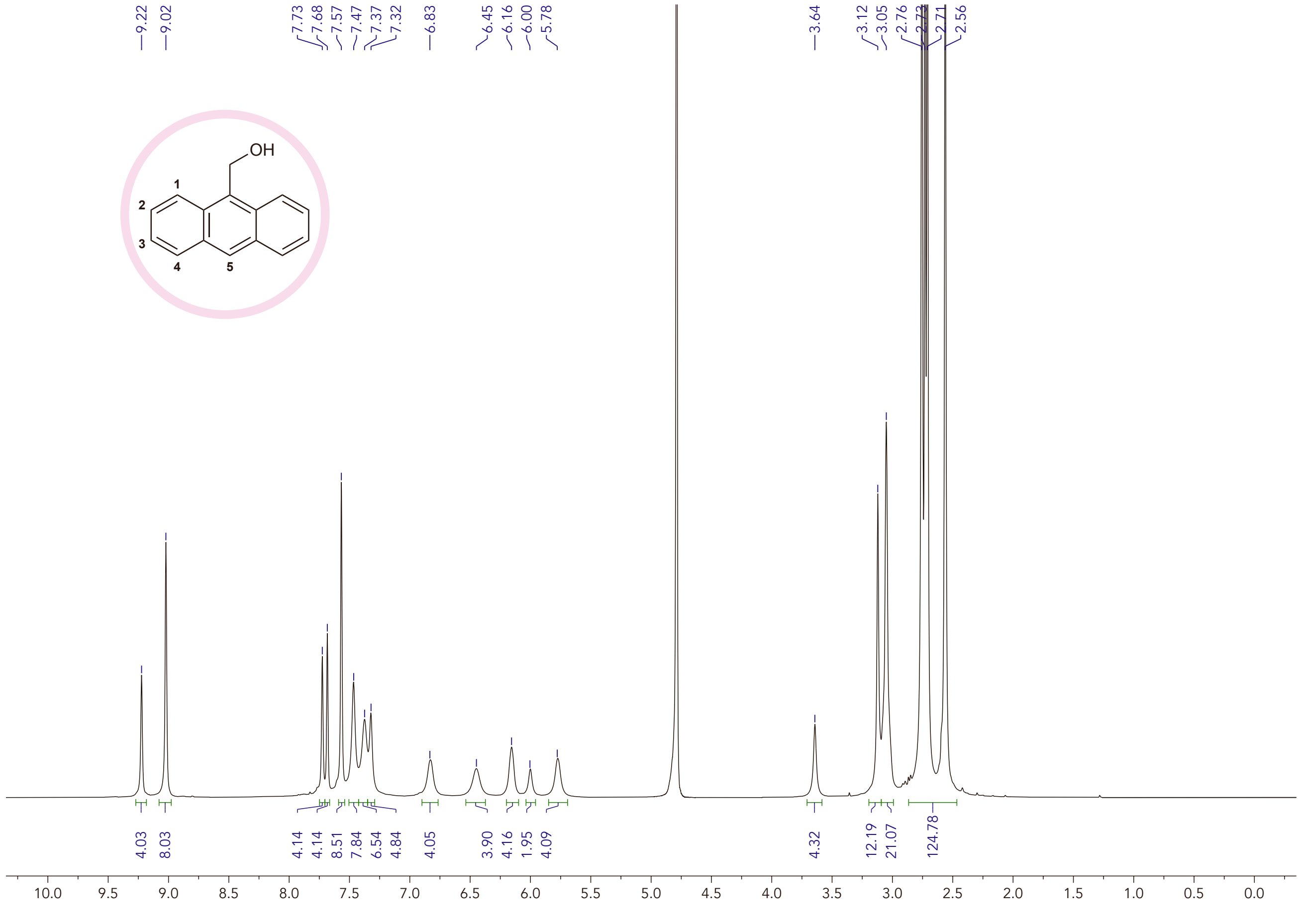


**Figure S39.** $^1$H NMR spectrum of (**a3**)$_2$⊂**C** (500 MHz, $D_2O$, 298 K).

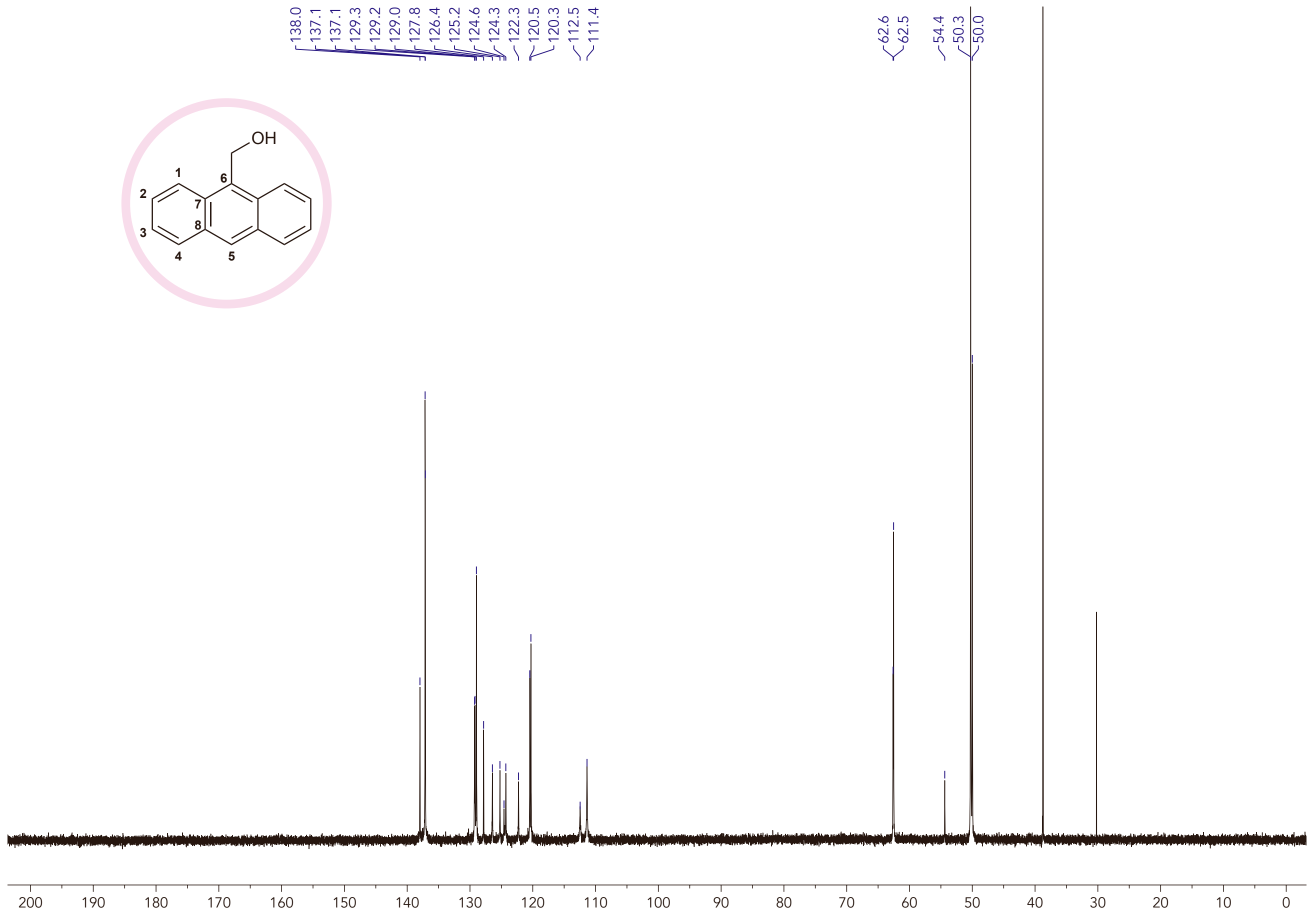


**Figure S40.** $^{13}C$ NMR spectrum of $(\mathbf{a3})_2 \subset \mathbf{C}$ (150 MHz, $D_2O$, 300 K).

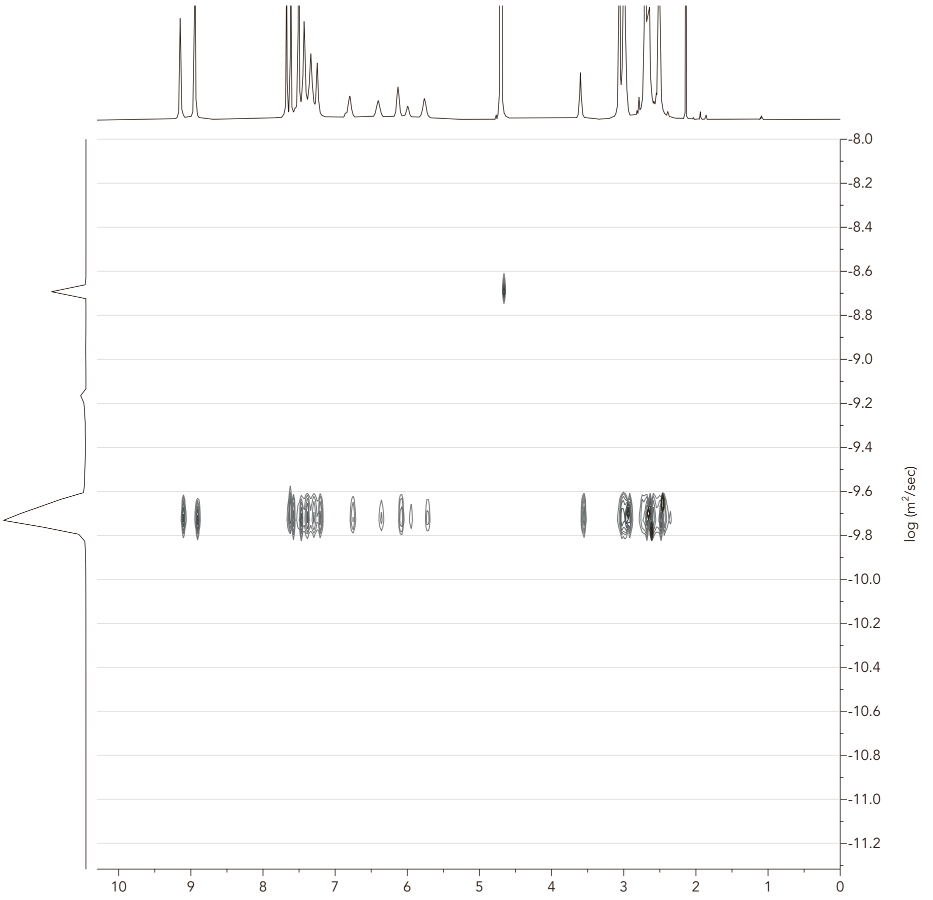


**Figure S41.** $^{1}$H DOSY NMR spectrum of $(\mathbf{a3})_2{\subset}\mathbf{C}$ (500 MHz, $D_2O$, 300 K).

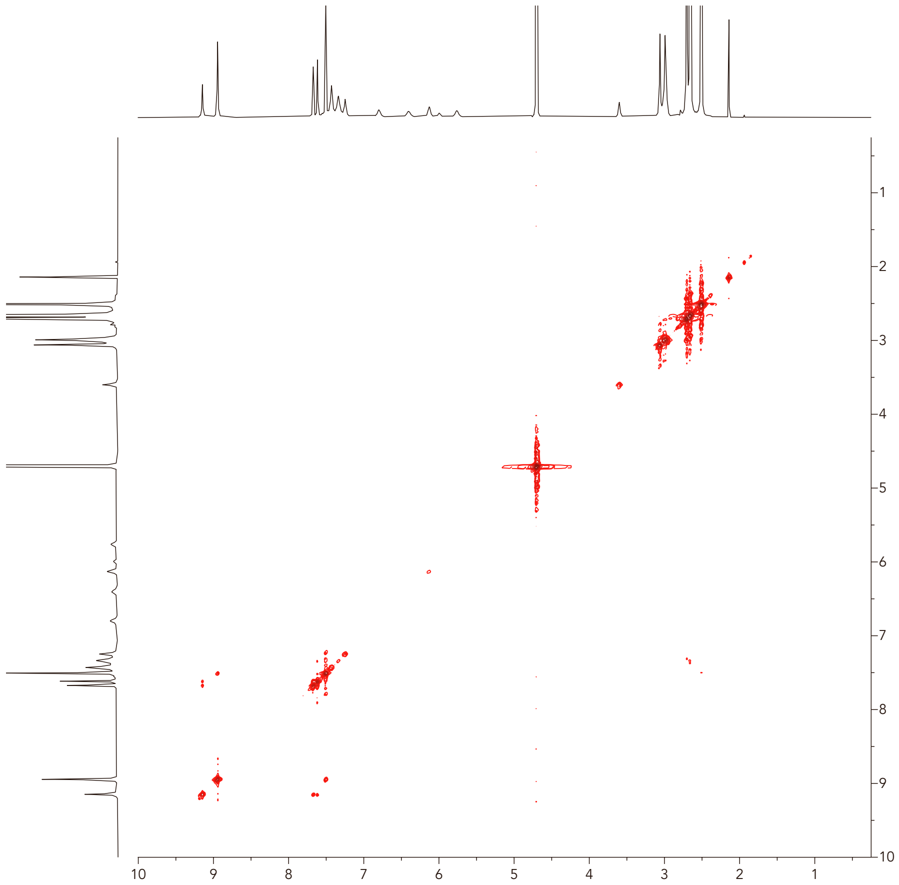


**Figure S42.** $^1H–^1H$ COSY NMR spectrum of (**a3**)$_2$⊂**C** (600 MHz, $D_2O$, 300 K).

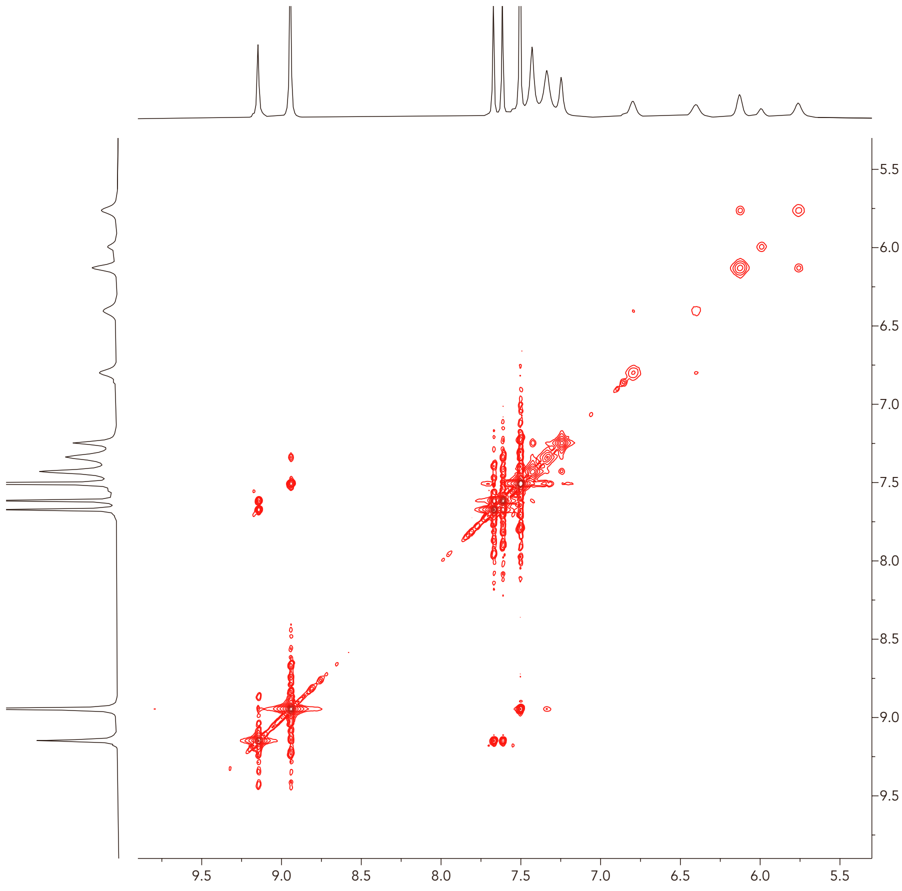


**Figure S43.** Partial $^{1}$H–$^{1}$H COSY NMR spectrum of $(\mathbf{a3})_2{\subset}\mathbf{C}$ (600 MHz, $D_2O$, 300 K).

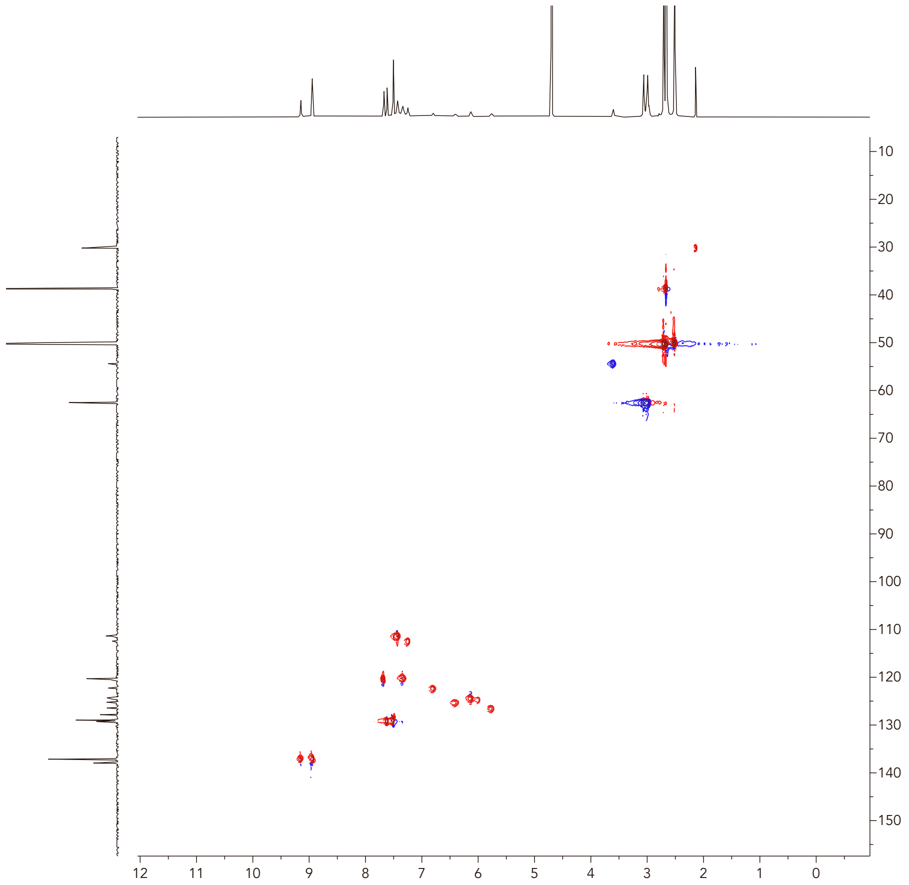


**Figure S44.** $^{1}H$–$^{13}C$ HSQC NMR spectrum of (**a3**)$_2$⊂**C** (600 MHz, $D_2O$, 300 K).

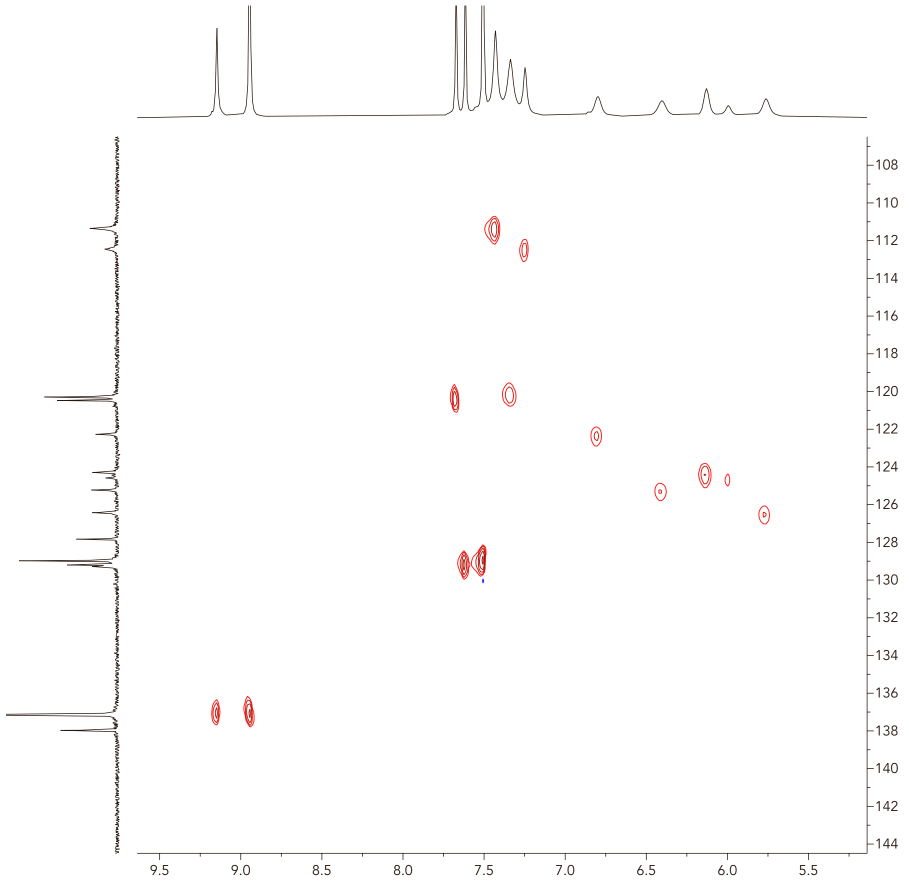


**Figure S45.** Partial $^{1}$H–$^{13}$C HSQC NMR spectrum of (**a3**)$_2$⊂**C** (600 MHz, $D_2O$, 300 K).

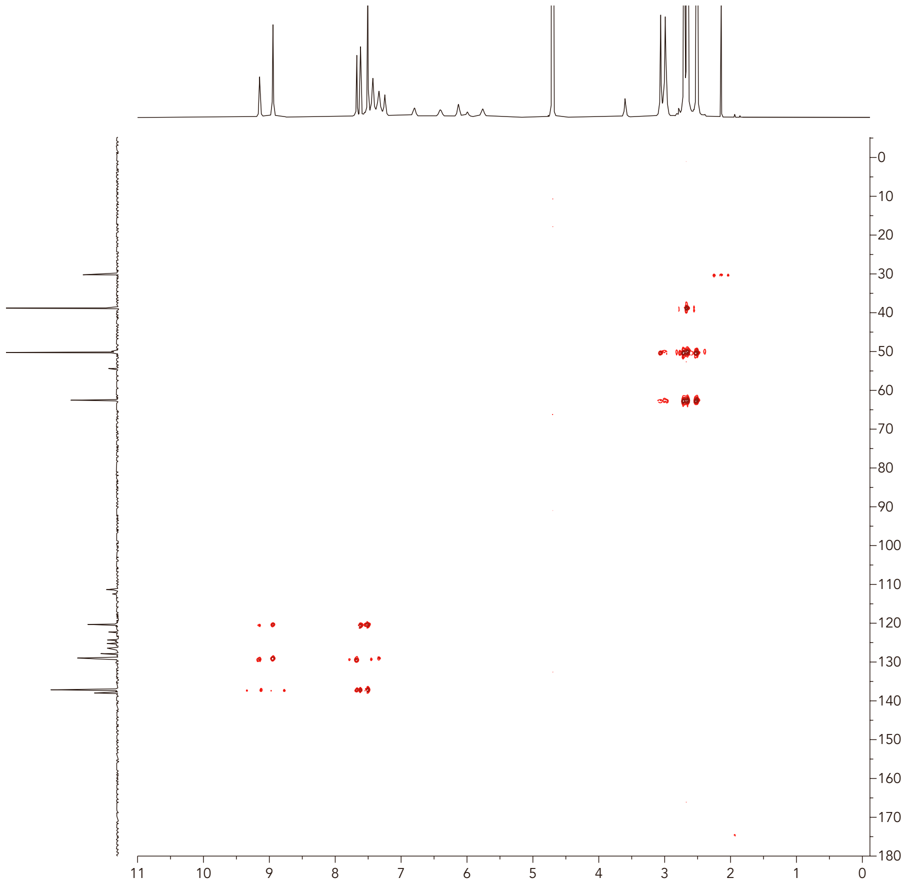


**Figure S46.** $^{1}H$–$^{13}C$ HMBC NMR spectrum of (**a3**)$_2$⊂**C** (600 MHz, $D_2O$, 300 K).

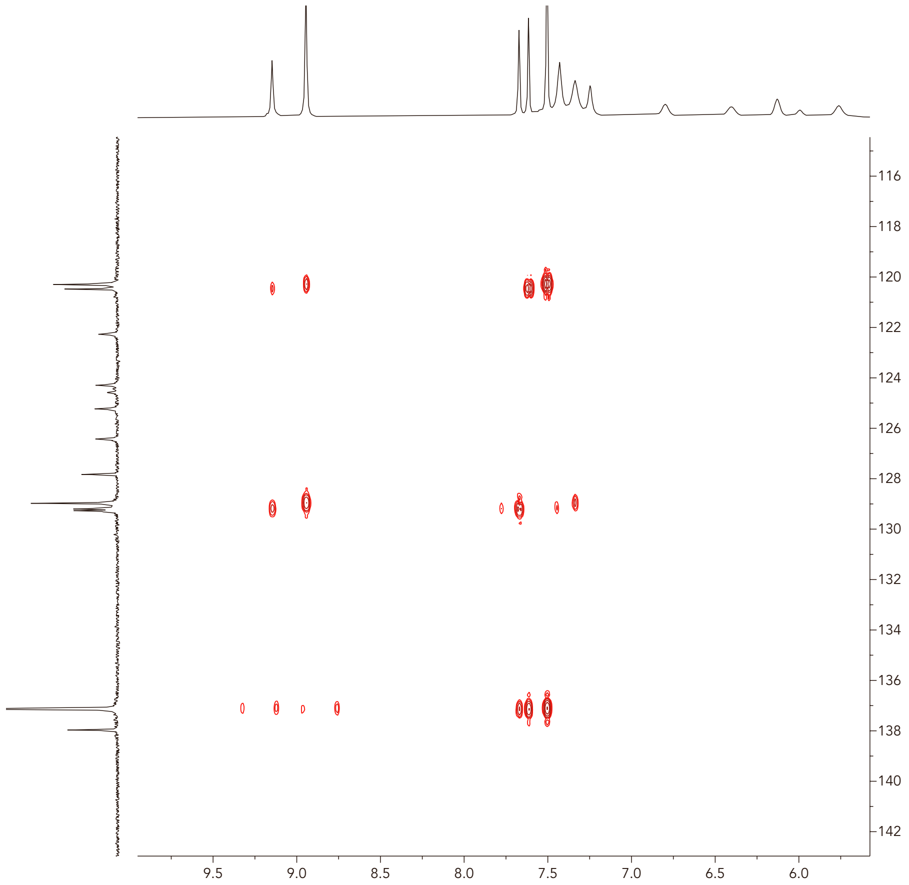


**Figure S47.** Partial $^{1}$H–$^{13}$C HMBC NMR spectrum of (**a3**)$_2$⊂**C** (600 MHz, $D_2O$, 300 K).

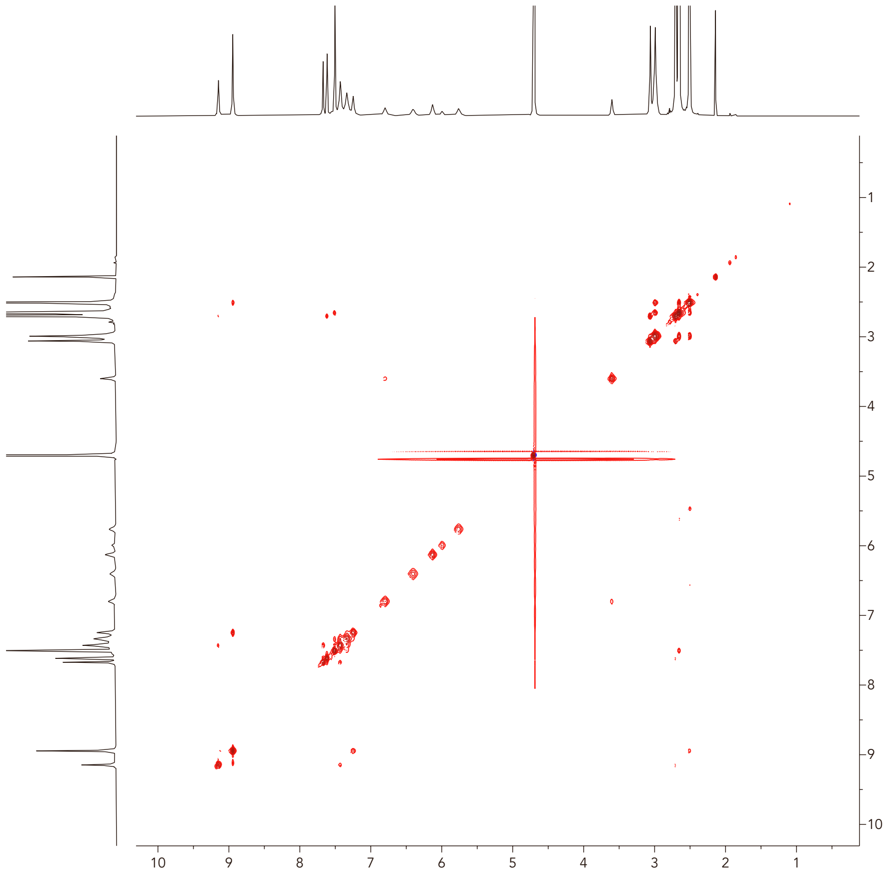


**Figure S48.** $^{1}$H–$^{1}$H NOESY NMR spectrum of (**a3**)$_2$⊂**C** (600 MHz, $D_2O$, 300 K).

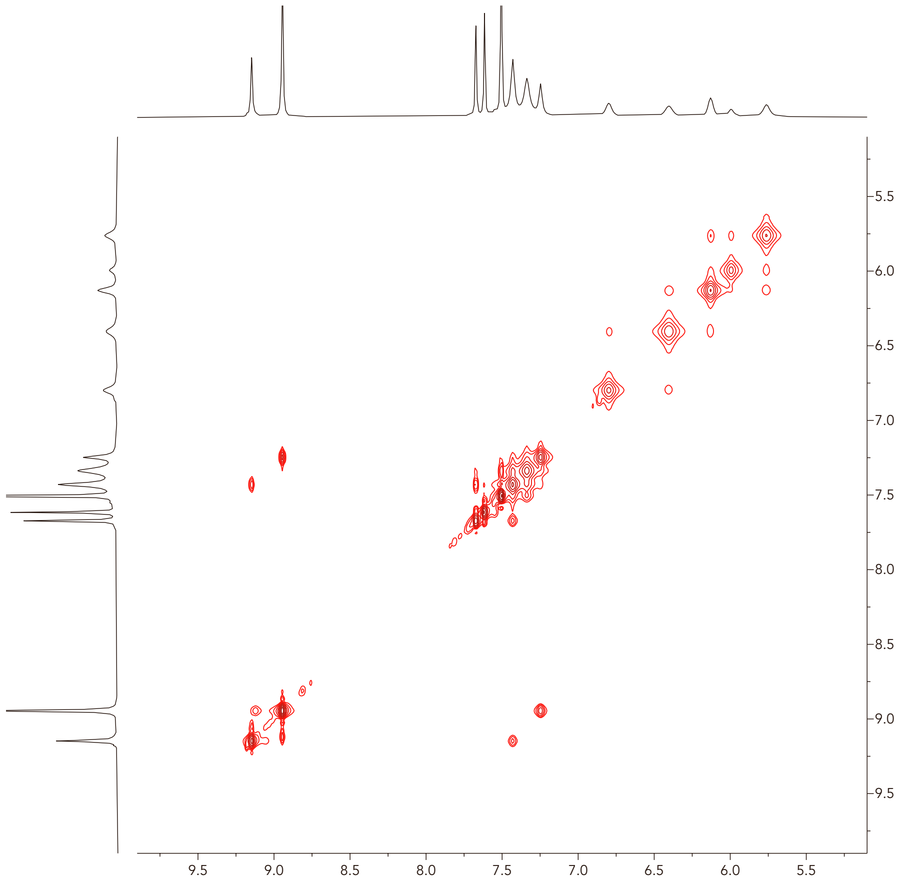


**Figure S49.** Partial $^{1}H$–$^{1}H$ NOESY NMR spectrum of **(a3)**$_2$⊂**C** (600 MHz, $D_2O$, 300 K).

Inclusion complex (**a4**)$_2$⊂**C** was obtained in a near-quantitative yield, as determined by $^1$H NMR spectroscopy.

$^1$H NMR (600 MHz, $D_2O$, 340 K): $\delta$ = 9.30 (s, 4H, **C$_1$**), 8.78 (s, 8H, **C$_4$**), 7.82 (br, 8H, **C$_{3+2}$**), 7.58 (s, 8H, **C$_5$**), 7.30 (s, 16H, **C$_{6+7}$**), 6.90 (br, 4H, **C$_8$**), 6.81 (s, 4H, **a4$_2$**), 6.46 (s, 8H, **a4$_1$**), 5.74 (s, 8H, **a4$_3$**), 3.14 (s, 8H, **C$_9$**), 3.00 (s, 16H, **C$_9$**), 2.84–2.44 (m, 72H, **C$_{10}$**).

$^{13}$C NMR (150 MHz, $D_2O$, 340 K): $\delta$ = 138.0 (**C$_{q'}$**), 137.2 (**C$_4$**), 137.0 (**C$_{1+q}$**), 130.2 (**C$_2$**), 129.4 (**C$_5$**), 128.9 (**a4$_4$**), 125.2 (**a4$_2$**), 124.8 (**a4$_3$**), 123.9 (**a4$_1$**), 122.5 (**a4$_5$**), 120.8 (**C$_3$**), 120.7 (**C$_6$**), 111.9 (**C$_8$**), 110.4 (**C$_7$**), 63.3 (**C$_9$**), 63.2 (**C$_9$**), 50.9 (**C$_{10}$**), 50.9 (**C$_{10}$**), 50.5 (**C$_{10}$**). (Note: **C$_q$** and **C$_{q'}$** denote H-free C atoms connected to the axial and equatorial imidazoles, respectively.)

$^1$H DOSY NMR (500 MHz, $D_2O$, 298 K): $D = 0.15 \cdot 10^{-5}$ cm$^2$/s.

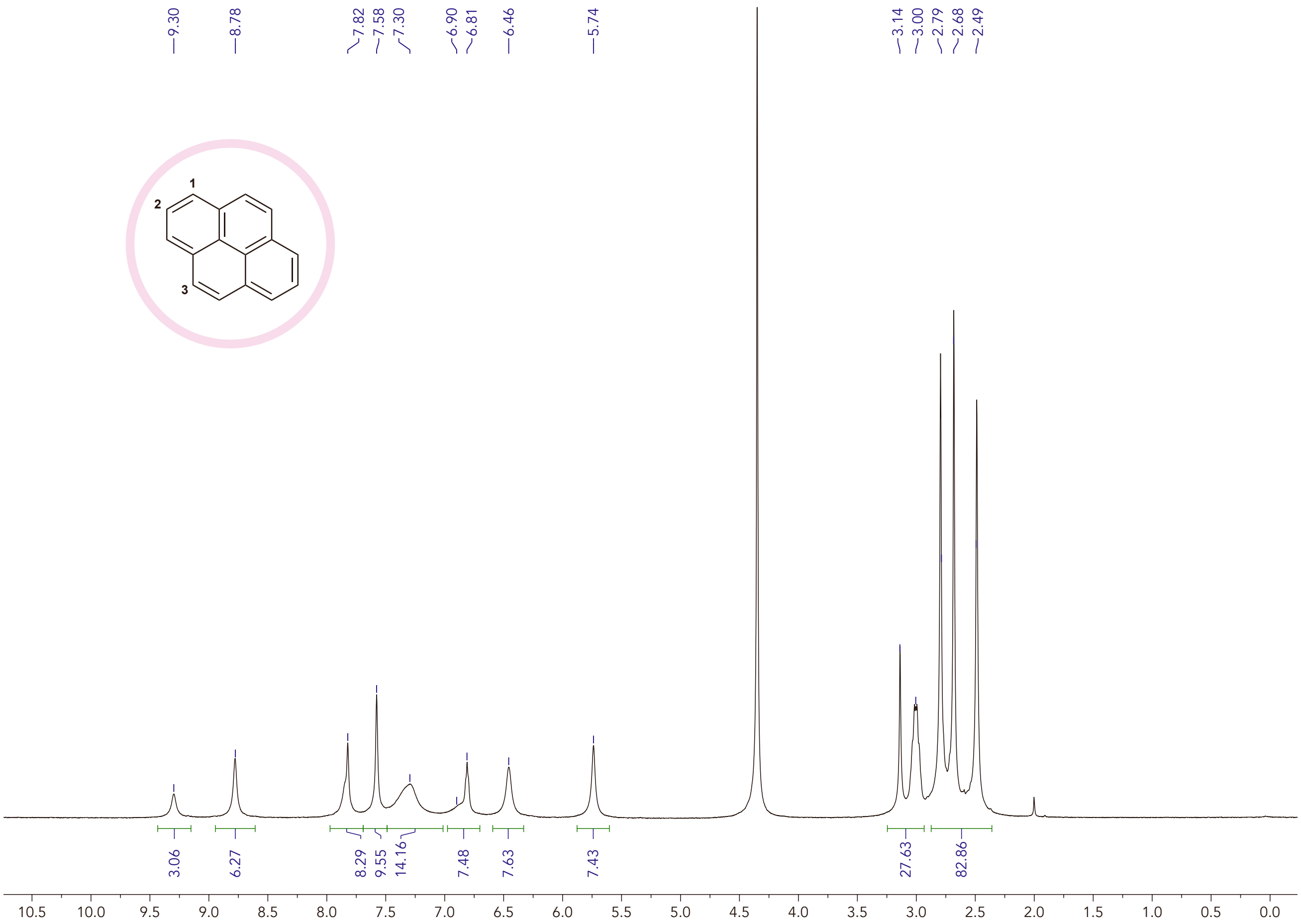


**Figure S50.** $^1$H NMR spectrum of (**a4**)$_2$⊂**C** (600 MHz, $D_2O$, 340 K).

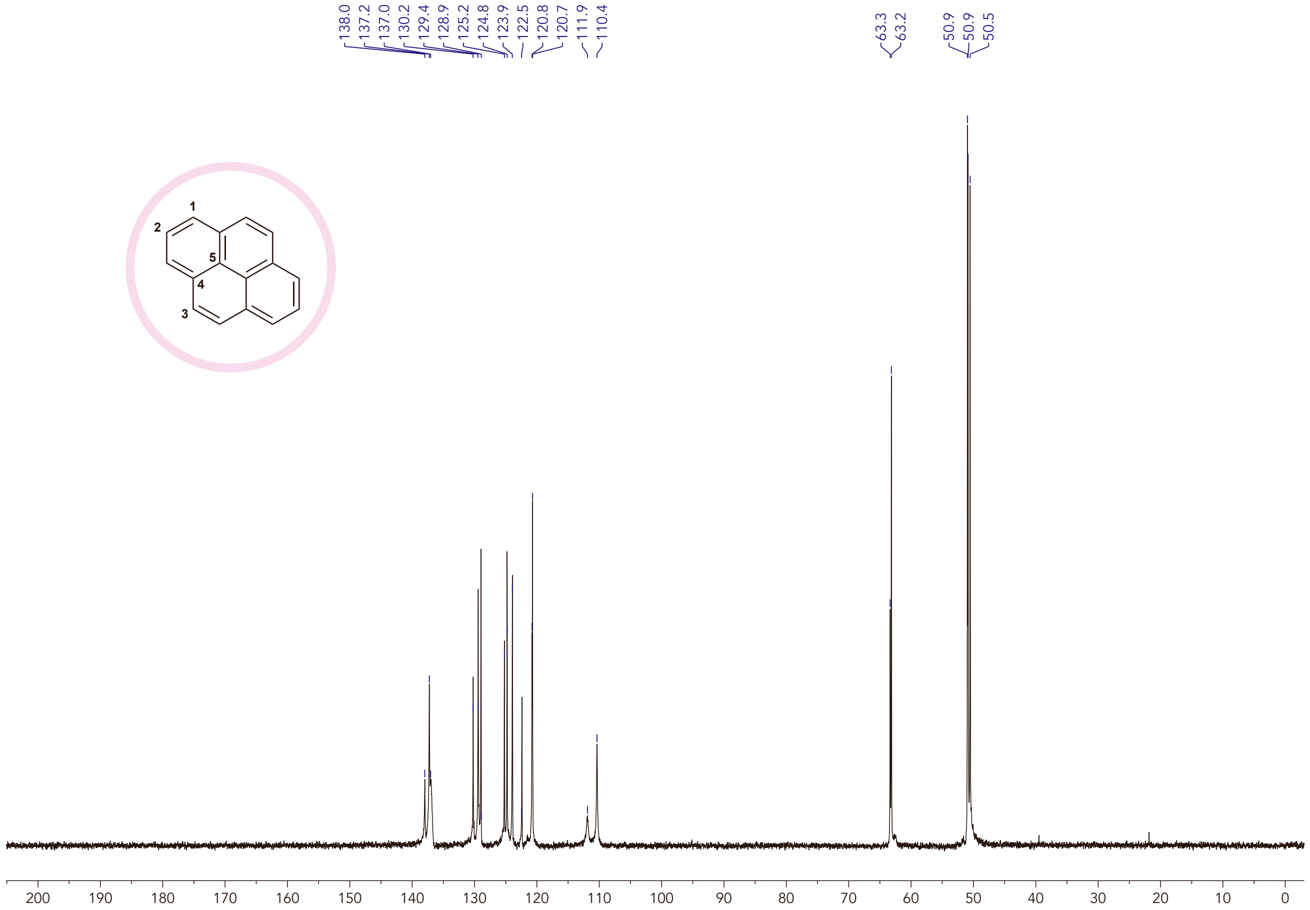


**Figure S51.** $^{13}$C NMR spectrum of (**a4**)$_2$⊂**C** (150 MHz, $D_2O$, 340 K).

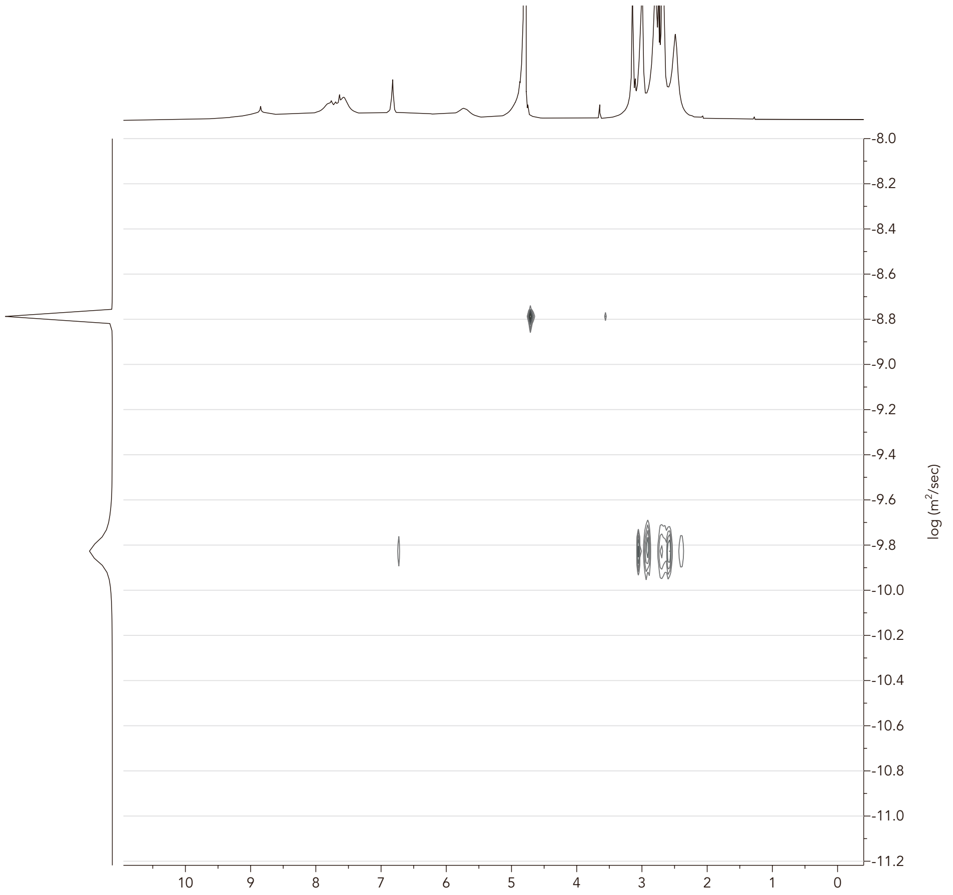


**Figure S52.** $^{1}$H DOSY NMR spectrum of (**a4**)$_2$⊂**C** (500 MHz, $D_2O$, 298 K). Note that the guest peaks are hardly visible due to the significant signals broadening at room temperature.

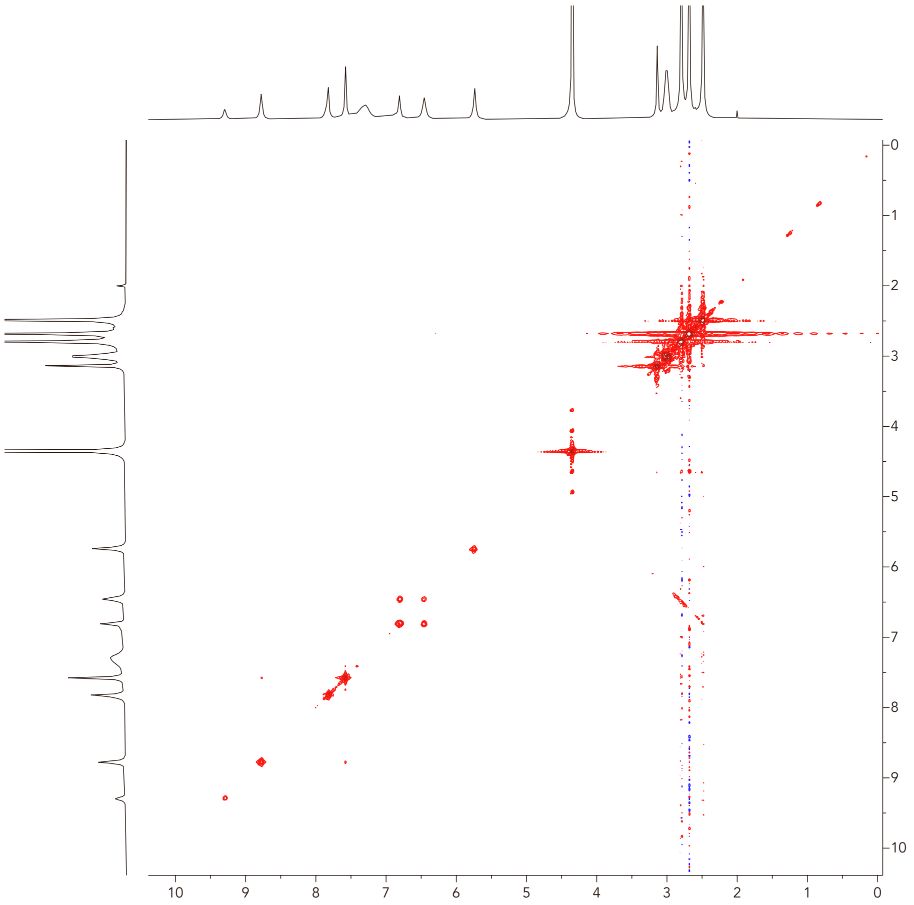


**Figure S53.** $^1H$–$^1H$ COSY NMR spectrum of (**a4**)$_2$⊂**C** (600 MHz, $D_2O$, 340 K).

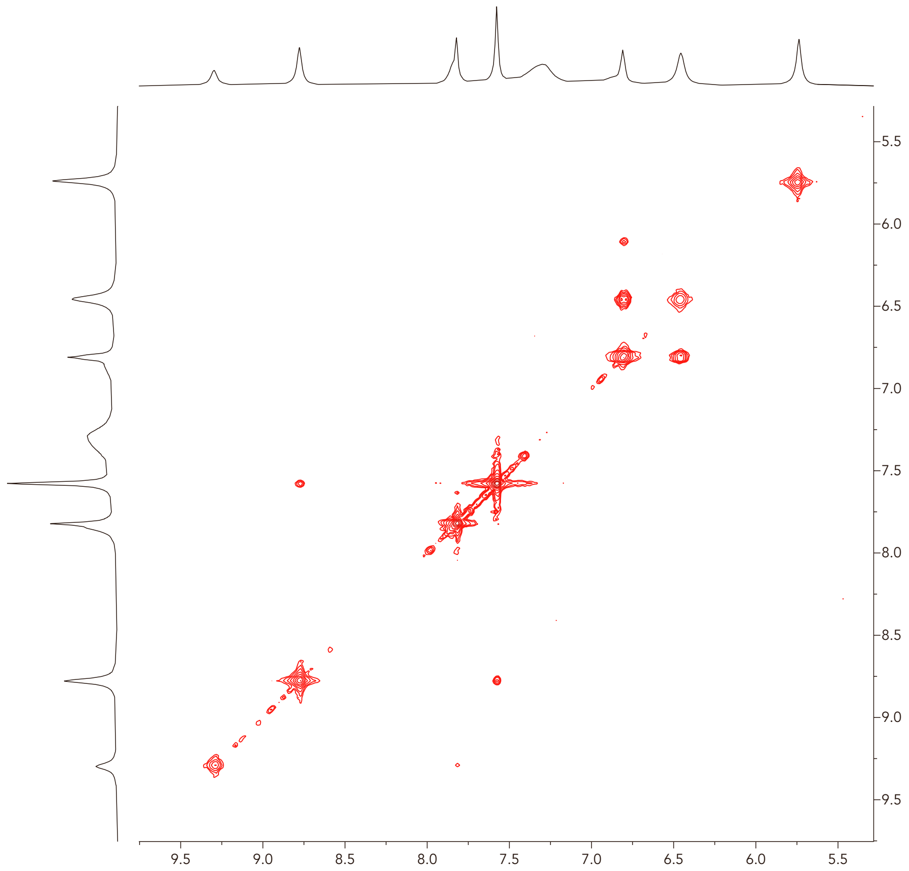


**Figure S54.** Partial $^{1}$H–$^{1}$H COSY NMR spectrum of $(\mathbf{a4})_2\subset\mathbf{C}$ (600 MHz, $D_2O$, 340 K).

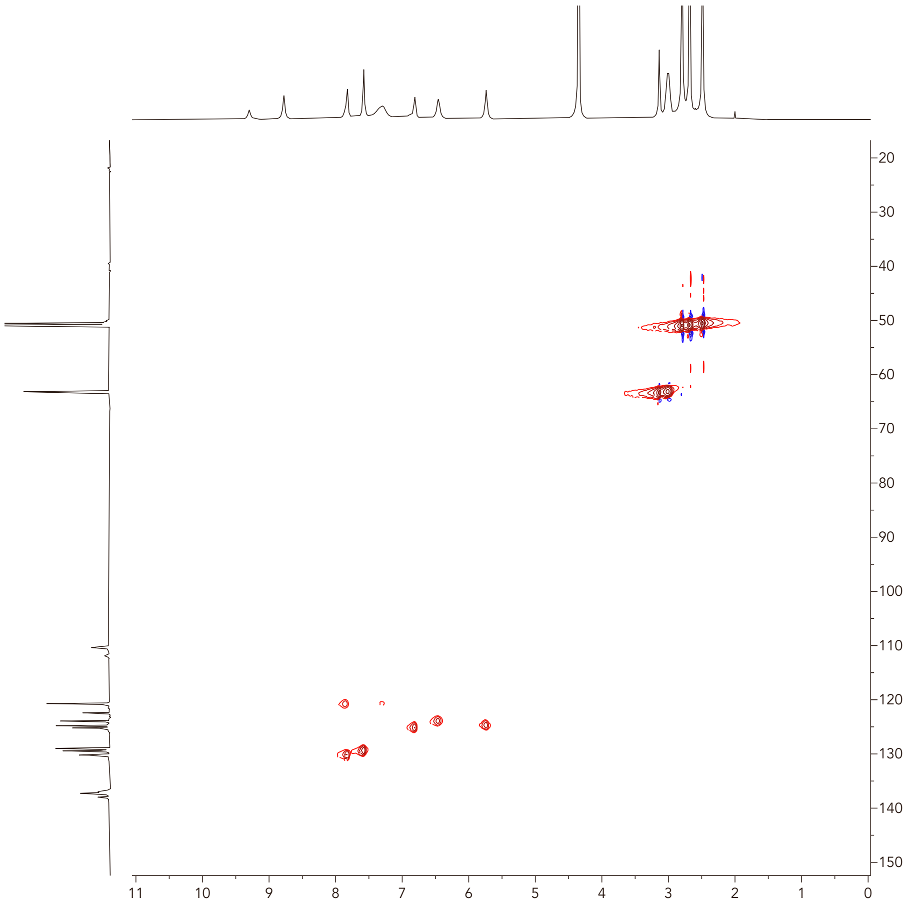


**Figure S55.** $^{1}H$–$^{13}C$ HSQC NMR spectrum of (**a4**)$_2$⊂**C** (600 MHz, $D_2O$, 340 K).

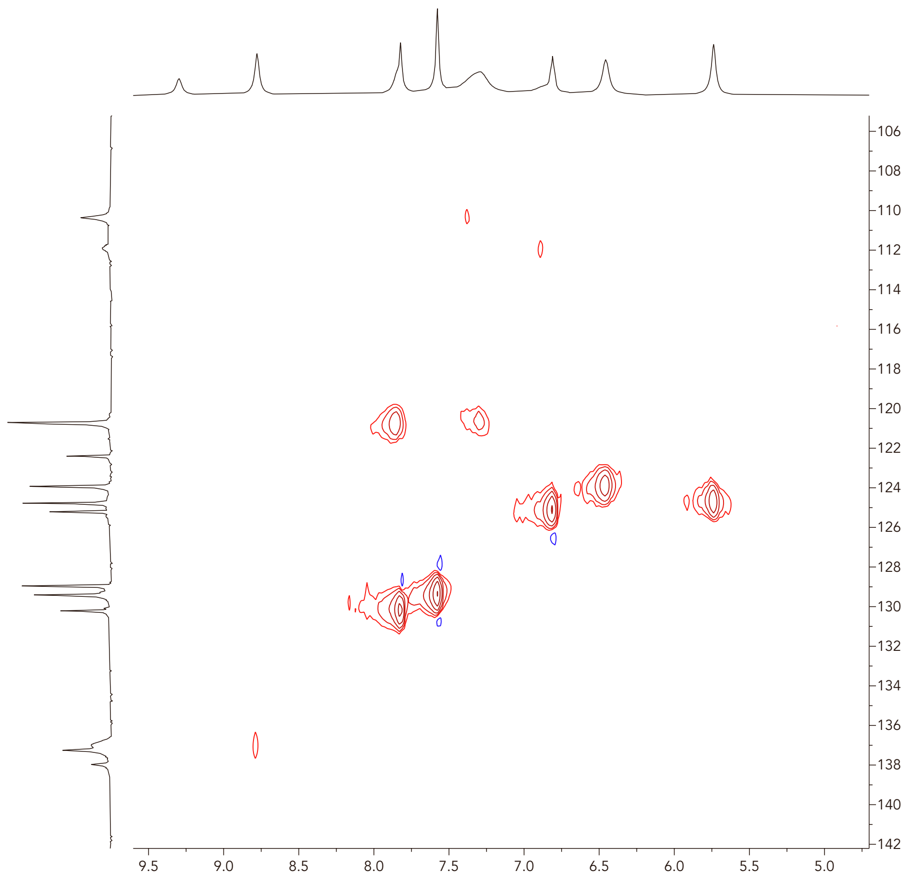


**Figure S56.** Partial $^{1}$H–$^{13}$C HSQC NMR spectrum of (**a4**)$_2$⊂**C** (600 MHz, $D_2O$, 340 K).

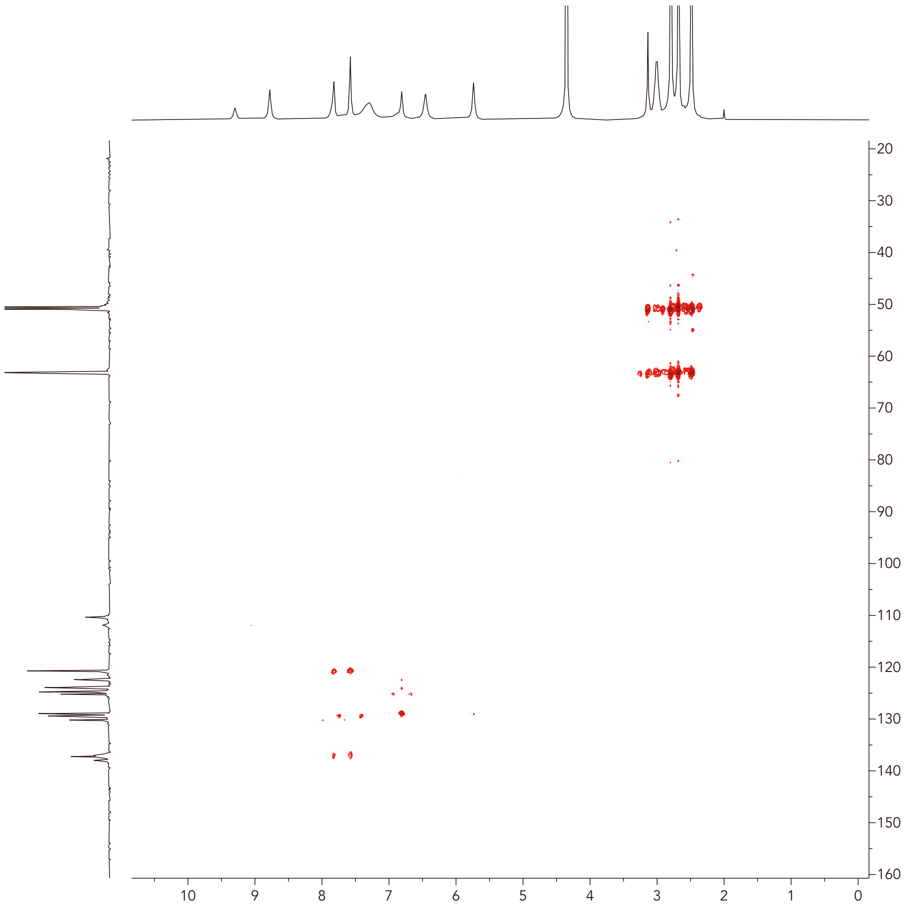


**Figure S57.** $^{1}H$–$^{13}C$ HMBC NMR spectrum of (**a4**)$_2$⊂**C** (600 MHz, $D_2O$, 340 K).

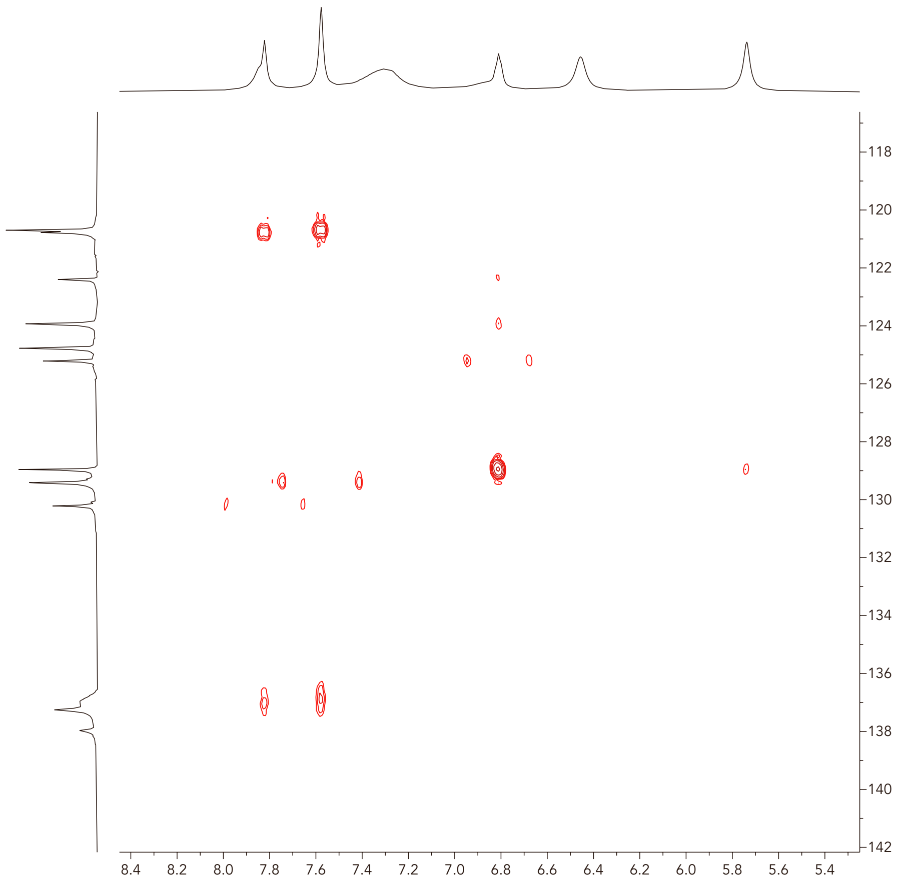


**Figure S58.** Partial $^{1}H$–$^{13}C$ HMBC NMR spectrum of (**a4**)$_2$⊂**C** (600 MHz, $D_2O$, 340 K).

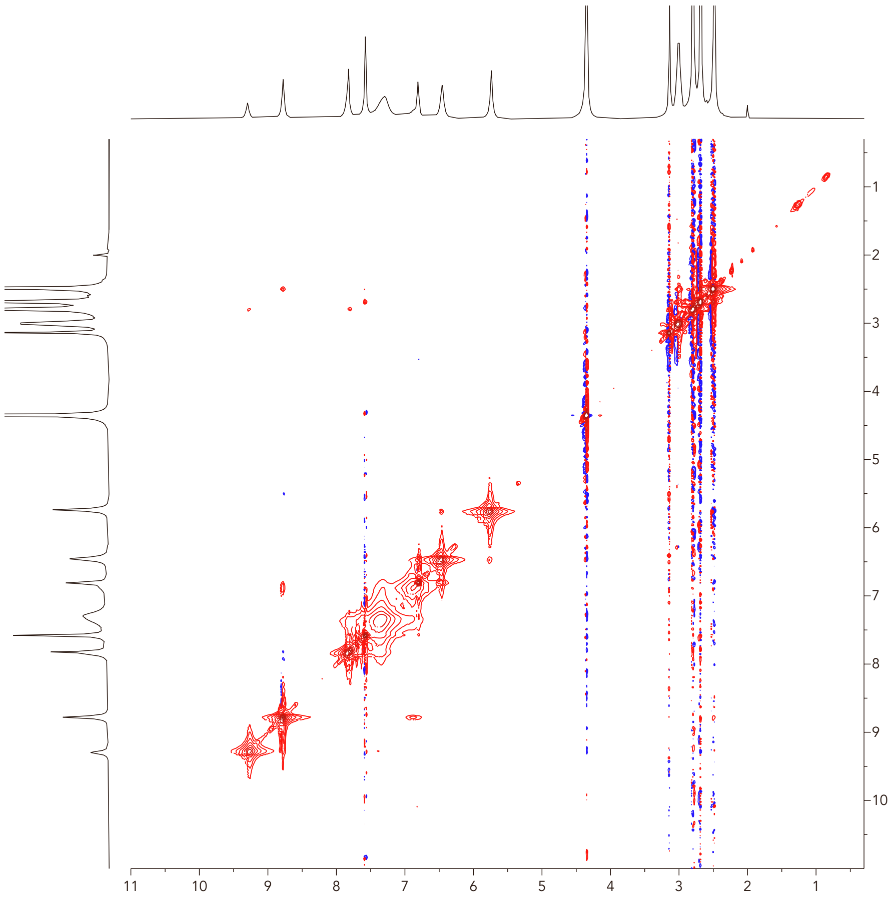


**Figure S59.** $^{1}H$–$^{1}H$ NOESY NMR spectrum of (**a4**)$_2$⊂**C** (600 MHz, $D_2O$, 340 K).

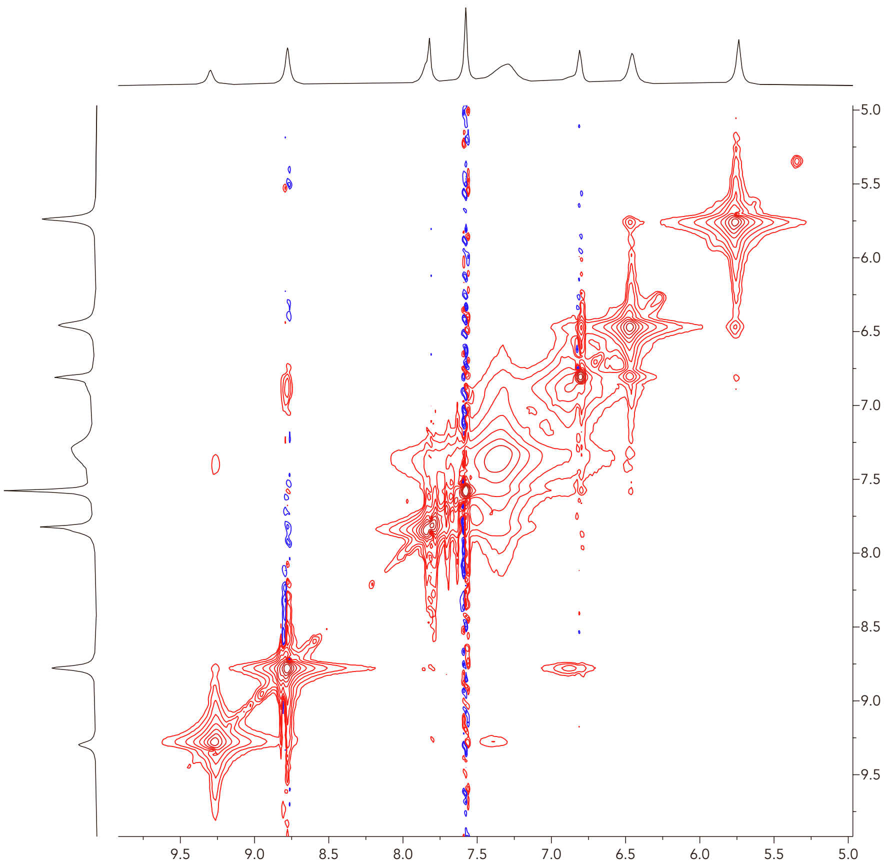


**Figure S60.** Partial $^{1}H$–$^{1}H$ NOESY NMR spectrum of (**a4**)$_2$⊂**C** (600 MHz, $D_2O$, 340 K).

## 6. NMR characterization of heterodimers (**a1·b1**)⊂**C**, (**a2·b1**)⊂**C**, (**a1·b2**)⊂**C**, and (**a4·b4**)⊂**C**

Heterodimeric inclusion complex (**a1·b1**)⊂**C** was obtained upon mixing homodimers (**a1**)$_2$⊂**C** and (**b1**)$_2$⊂**C** in a 1:1 ratio (with respect to the cage; note: the encapsulation yield for (**b1**)$_2$⊂**C** is ~50%, thus ~25% of the cage present in the system is unoccupied). The coexistence of the heterodimer with the two homodimers and free cage resulted in a complex NMR spectrum; consequently, only characteristic peaks originating from the guests are listed below (note: guest protons with an apostrophe denote guests encapsulated within heterodimer (**a1·b1**)⊂**C**; those without an apostrophe refer to guests within homodimers (**a1**)$_2$⊂**C** and (**b1**)$_2$⊂**C**[2]):

$^1$H NMR (600 MHz, $D_2O$, 320 K); guest peaks: $\delta$ = 5.59 (s, **b1**$_4$), 5.32 (s, **b1**$_{4'}$), 1.83−1.80 (s, **b1**$_1$+**b1**$_{1'}$), 0.97 (s, **a1**$_{CH_3'}$), 0.65 (s, **b1**$_2$), 0.31 (s, **b1**$_{2'}$), 0.27 (s, **b1**$_3$), 0.13 (s, **a1**$_{CH_3}$), –0.62 (s, **b1**$_{3'}$).

$^1$H DOSY NMR (500 MHz, $D_2O$, 300 K): $D = 0.22\cdot10^{-5}$ cm$^2$/s.

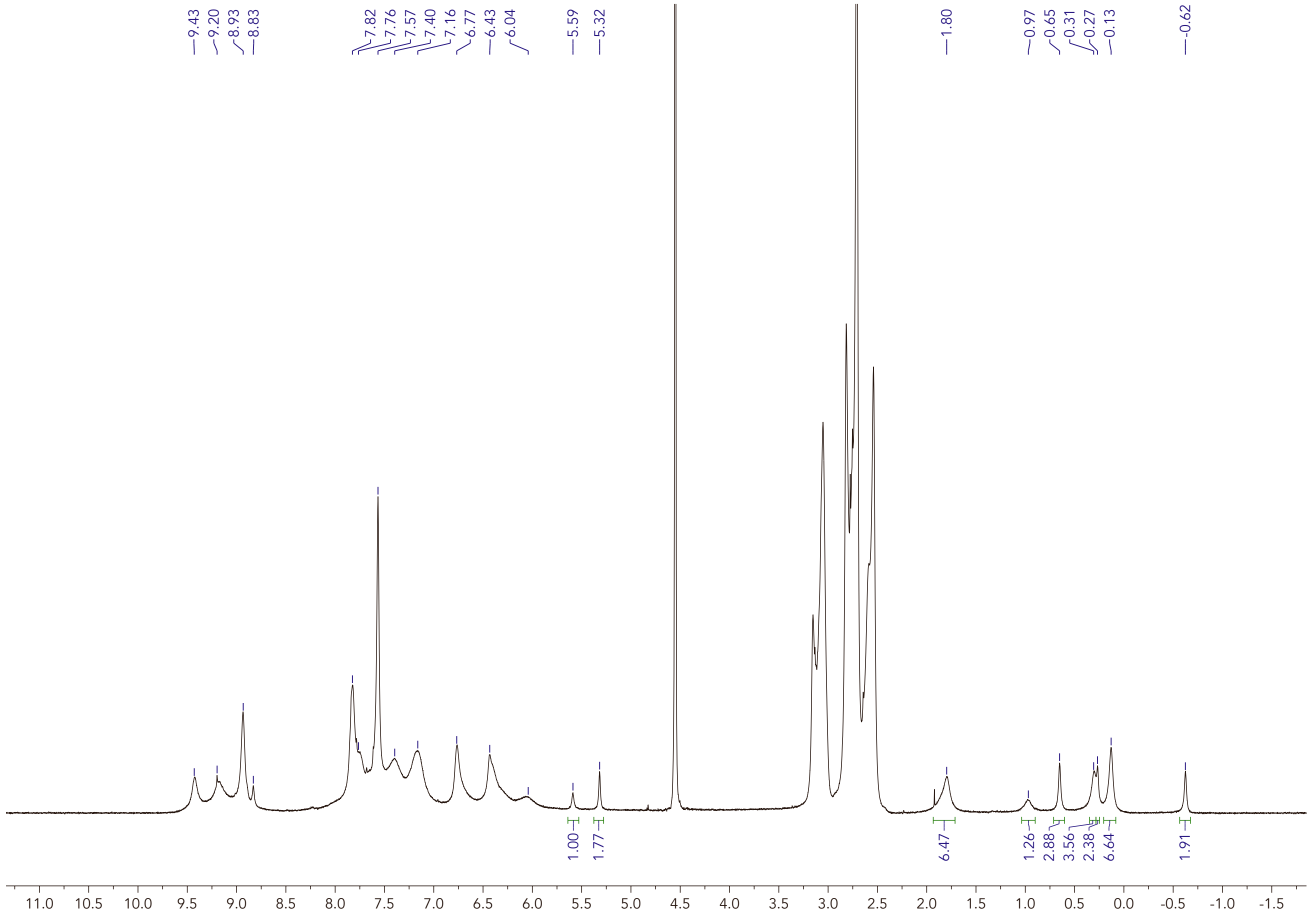


**Figure S61.** $^1$H NMR spectrum of (**a1·b1**)⊂**C** (in the presence of (**a1**)$_2$⊂**C**, (**b1**)$_2$⊂**C**, and free **C**) (600 MHz, $D_2O$, 320 K).

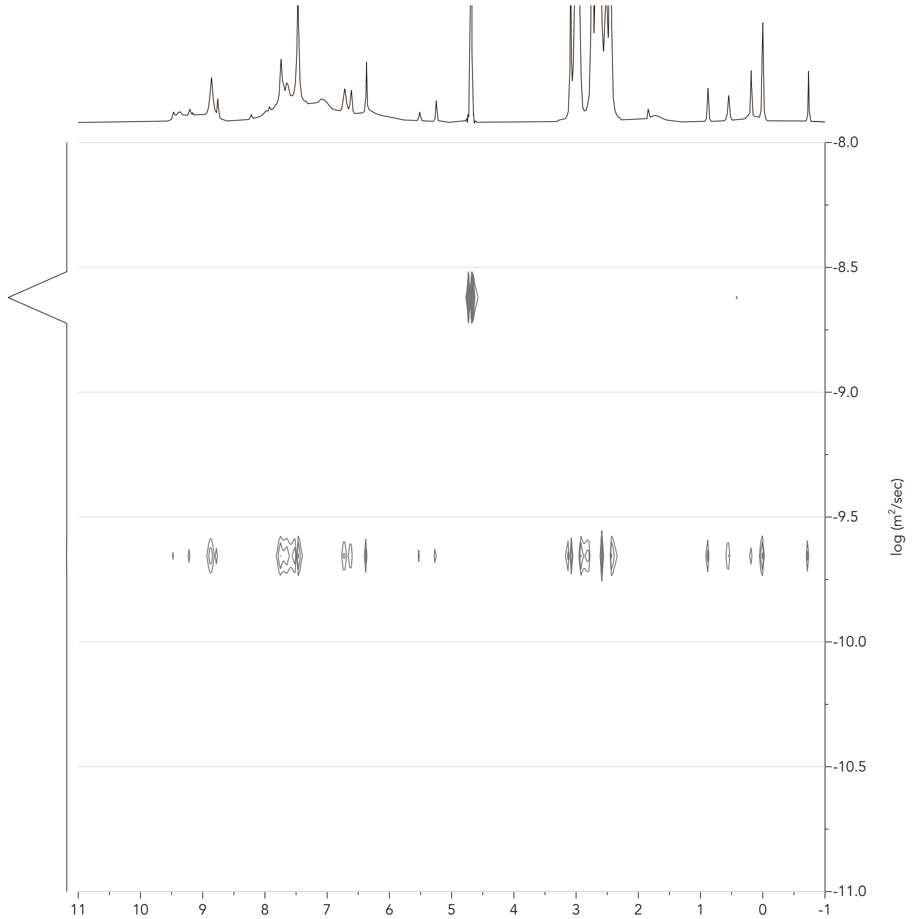


**Figure S62.** ¹H DOSY NMR spectrum of (**a1**·**b1**)⊂**C** (in the presence of (**a1**)$_2$⊂**C**, (**b1**)$_2$⊂**C**, and free **C**) (500 MHz, $D_2O$, 300 K).

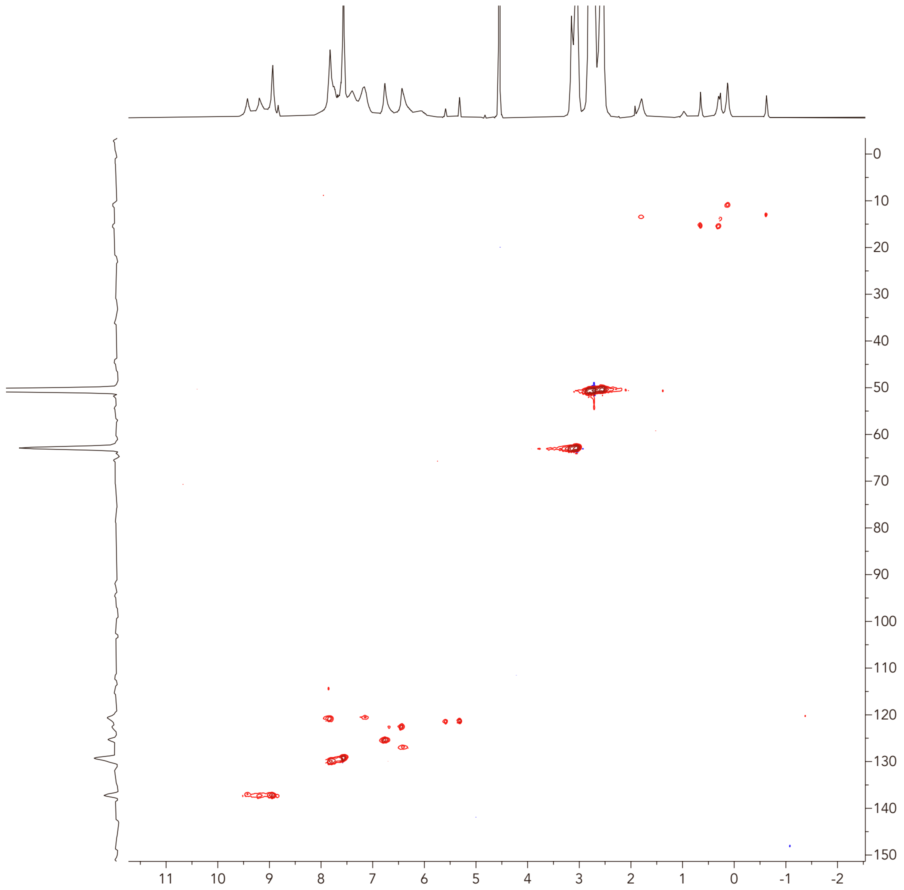


**Figure S63.** $^1$H–$^{13}$C HSQC NMR spectrum of (**a1**·**b1**)⊂**C** (in the presence of (**a1**)$_2$⊂**C**, (**b1**)$_2$⊂**C**, and free **C**) (600 MHz, $D_2O$, 320 K).

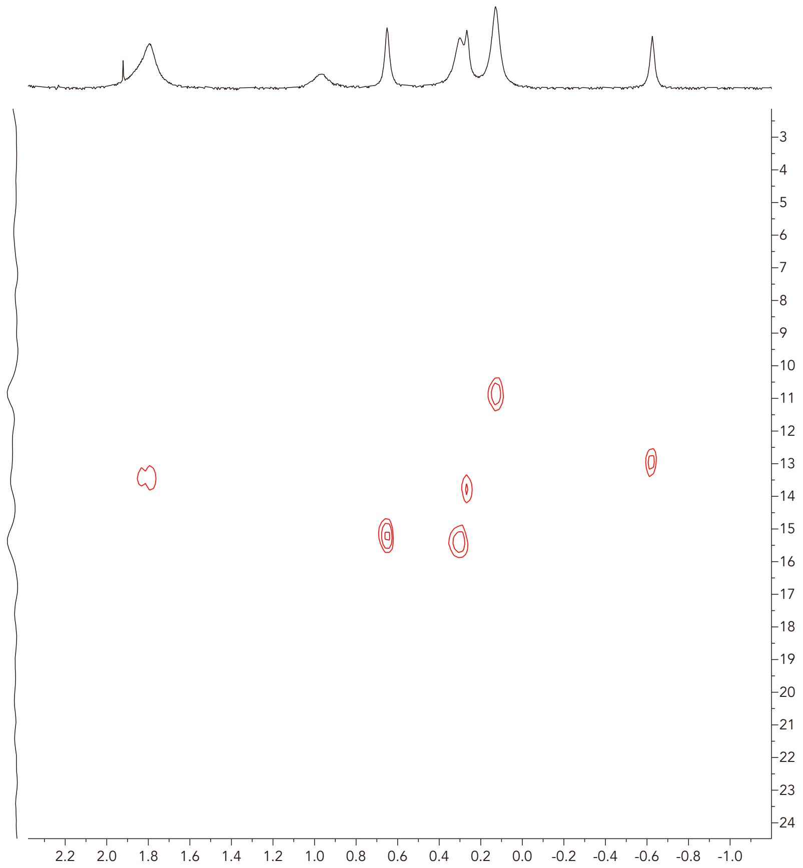


**Figure S64.** Partial $^{1}$H–$^{13}$C HSQC NMR spectrum of (**a1**·**b1**)⊂**C** (in the presence of $(\mathbf{a1})_2$⊂**C**, $(\mathbf{b1})_2$⊂**C**, and free **C**) (600 MHz, $D_2O$, 320 K).

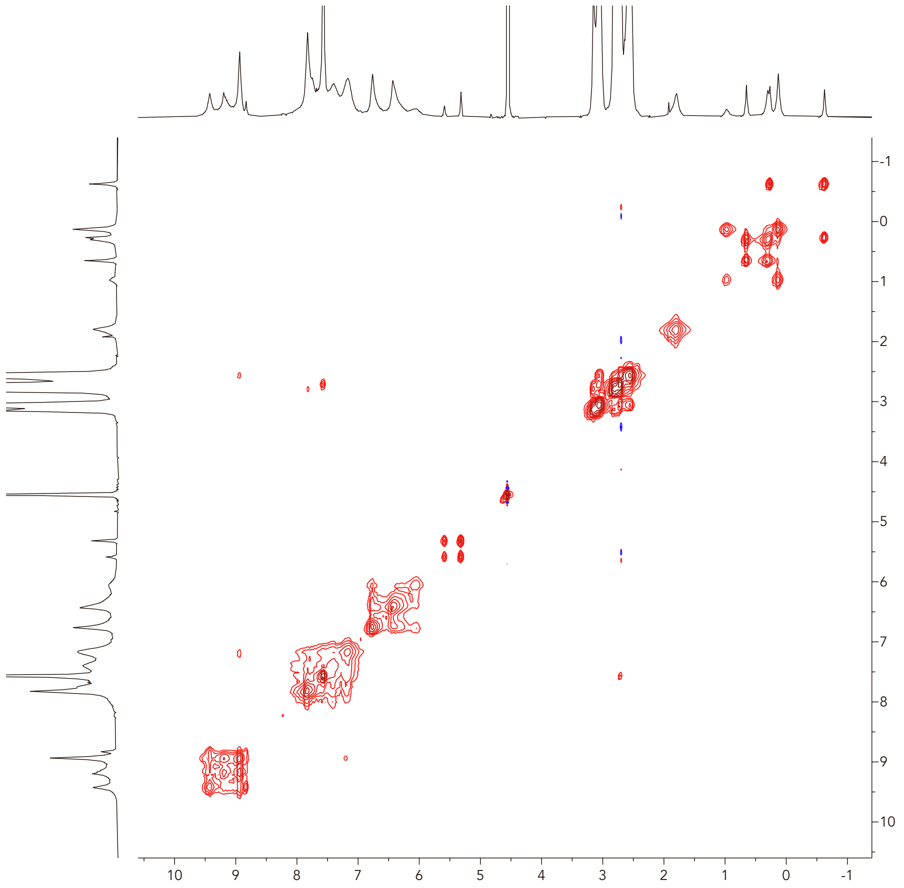


**Figure S65.** $^{1}$H–$^{1}$H NOESY NMR spectrum of (**a1**·**b1**)⊂**C** (in the presence of (**a1**)$_2$⊂**C**, (**b1**)$_2$⊂**C**, and free **C**) (600 MHz, $D_2O$, 320 K).

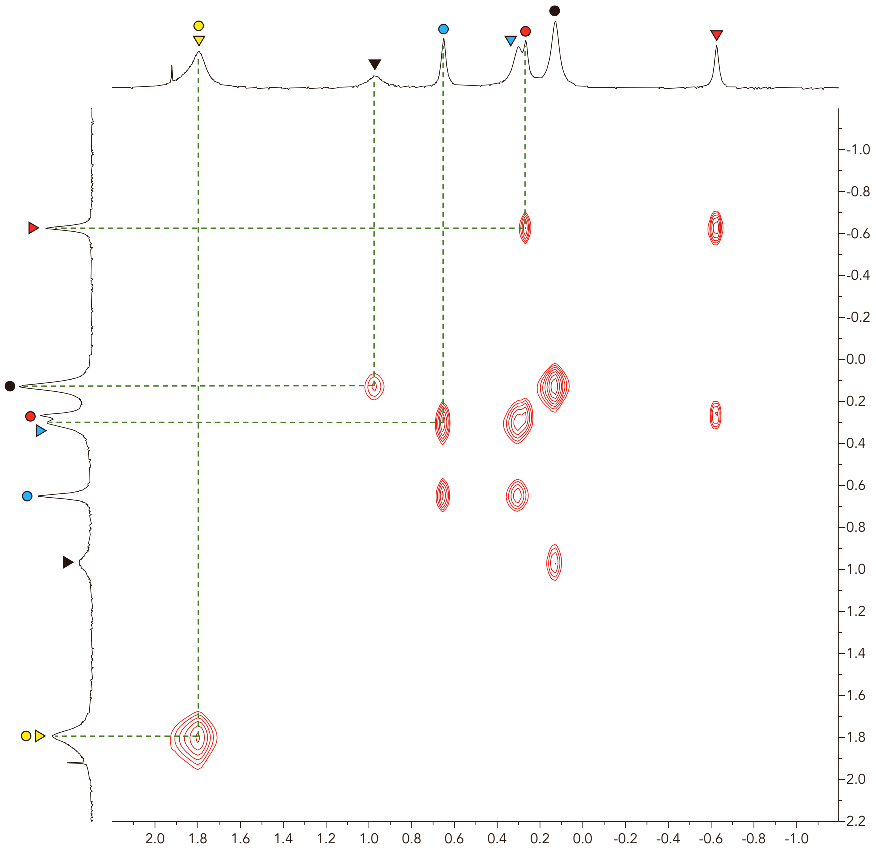


**Figure S66.** Partial $^1H$–$^1H$ EXSY NMR spectrum of (**a1**·**b1**)⊂**C** (in the presence of (**a1**)$_2$⊂**C** and (**b1**)$_2$⊂**C**) (600 MHz, $D_2O$, 320 K). Peak labels as in Figure 2C of the main text.

Heterodimeric inclusion complex (**a2**·**b1**)⊂**C** was obtained upon mixing homodimers (**a2**)$_2$⊂**C** and (**b1**)$_2$⊂**C** in a 1:1 ratio (with respect to the cage; note: the encapsulation yield for (**b1**)$_2$⊂**C** is ~50%, hence ~25% of the cage present in the system is unoccupied). The coexistence of the heterodimer with the two homodimers resulted in a complex NMR spectrum; consequently, only characteristic peaks originating from the guests are listed below (note: guest protons with an apostrophe denote guests encapsulated within heterodimer (**a2**·**b1**)⊂**C**; those without an apostrophe refer to guests within homodimer (**b1**)$_2$⊂**C** (Ref. 2)):

$^1$H NMR (600 MHz, $D_2O$, 320 K); guest peaks: $\delta$ = 5.59 (s, **b1$_4$**), 5.15 (s, **b1$_{4'}$**), 1.85 (s, **b1$_1$**), 1.75 (s, **b1$_{1'}$**), 0.65 (s, **b1$_2$**), 0.40 (s, **b1$_{2'}$**), 0.26 (s, **b1$_3$**), –0.37 (s, **b1$_{3'}$**).

$^1$H DOSY NMR (500 MHz, $D_2O$, 300 K): $D = 0.20 \cdot 10^{-5}$ cm$^2$/s.

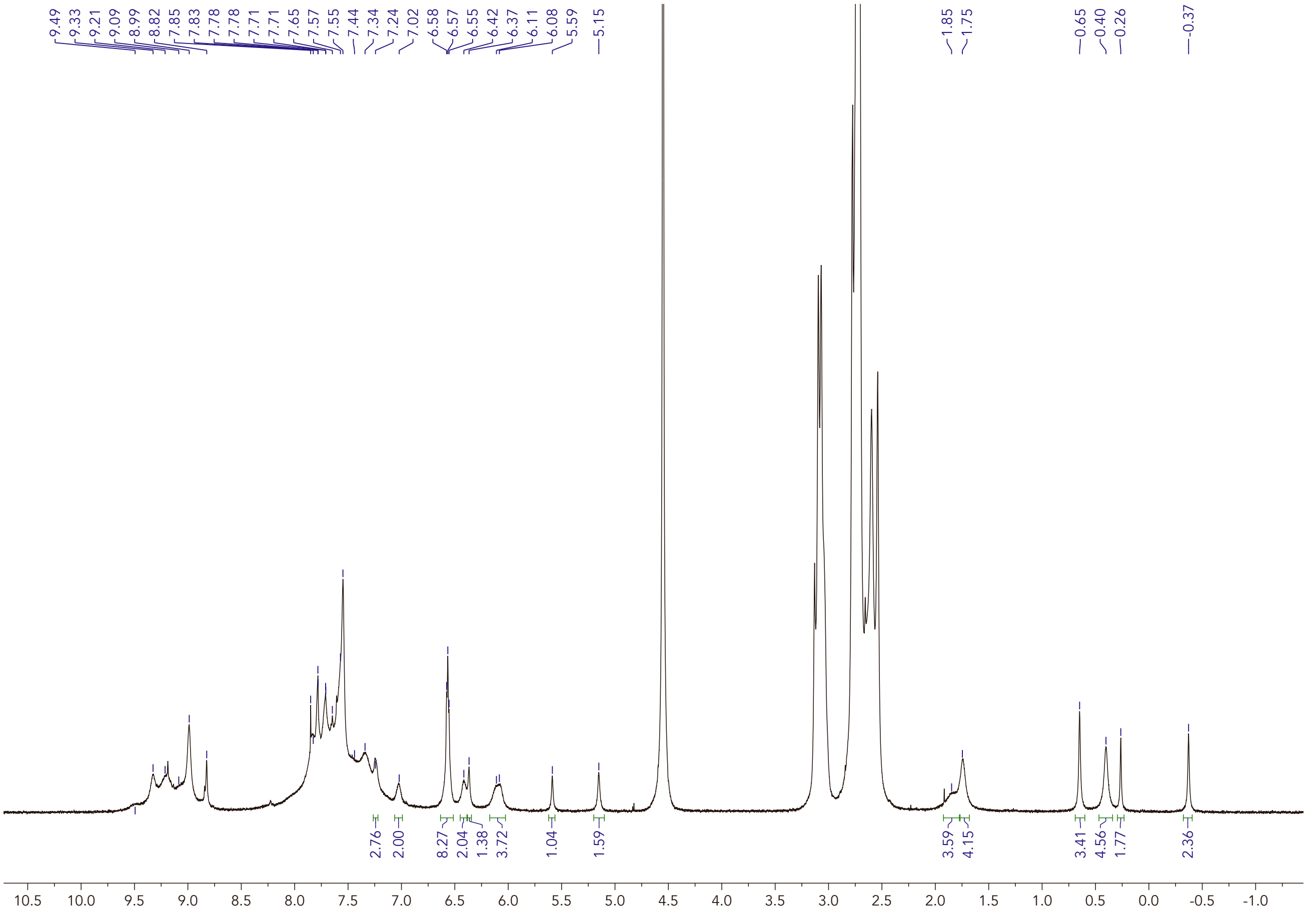


**Figure S67.** $^1$H NMR spectrum of (**a2**·**b1**)⊂**C** (in the presence of (**a2**)$_2$⊂**C**, (**b1**)$_2$⊂**C**, and free **C**) (600 MHz, $D_2O$, 320 K).

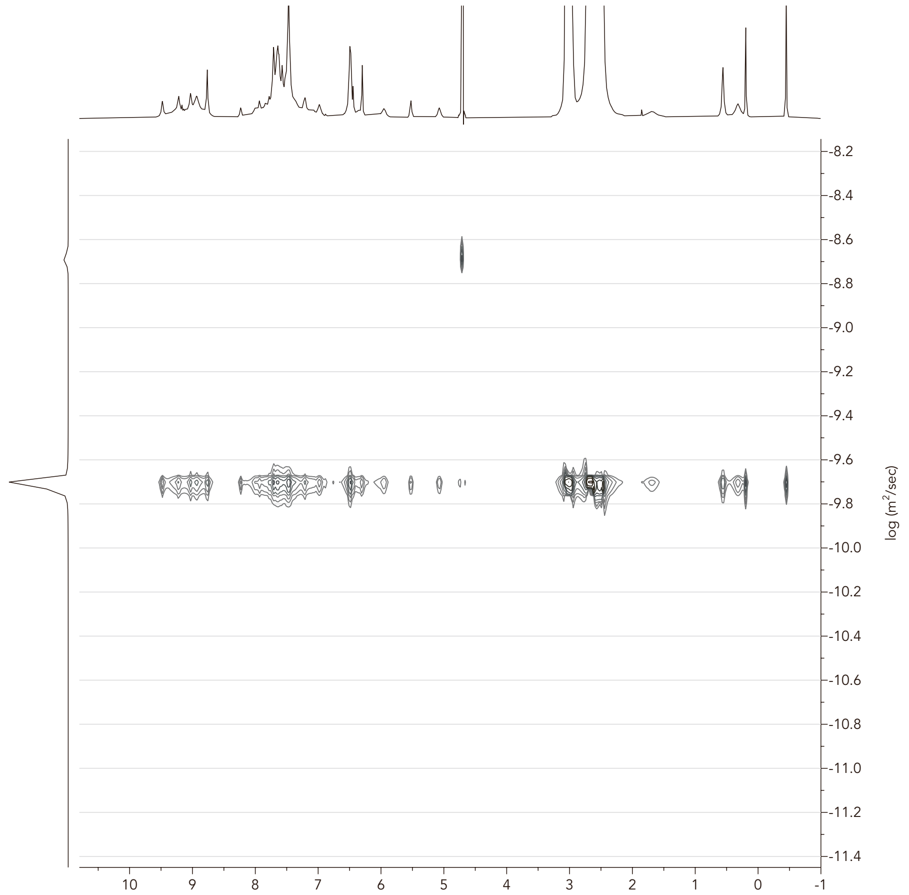


**Figure S68.** $^{1}$H DOSY NMR spectrum of (**a2**·**b1**)⊂**C** (in the presence of (**a2**)$_2$⊂**C**, (**b1**)$_2$⊂**C**, and free **C**) (500 MHz, $D_2O$, 300 K).

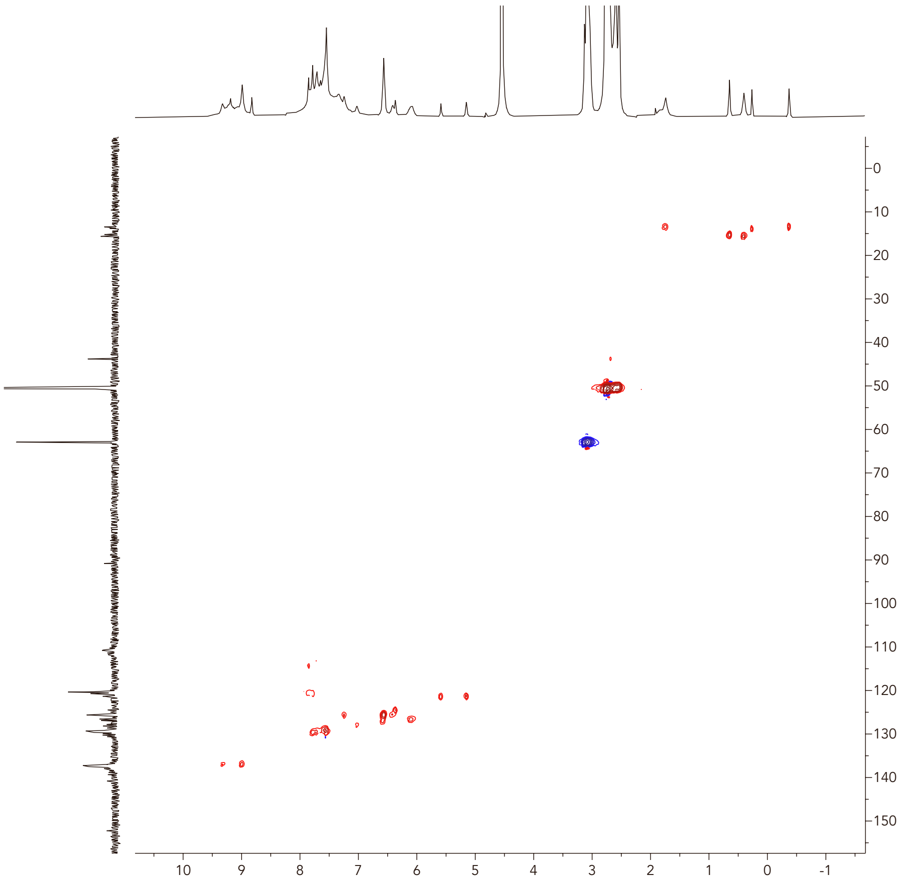


**Figure S69.** $^{1}H$–$^{13}C$ HSQC NMR spectrum of (**a2**·**b1**)⊂**C** (in the presence of (**a2**)$_2$⊂**C**, (**b1**)$_2$⊂**C**, and free **C**) (600 MHz, $D_2O$, 320 K).

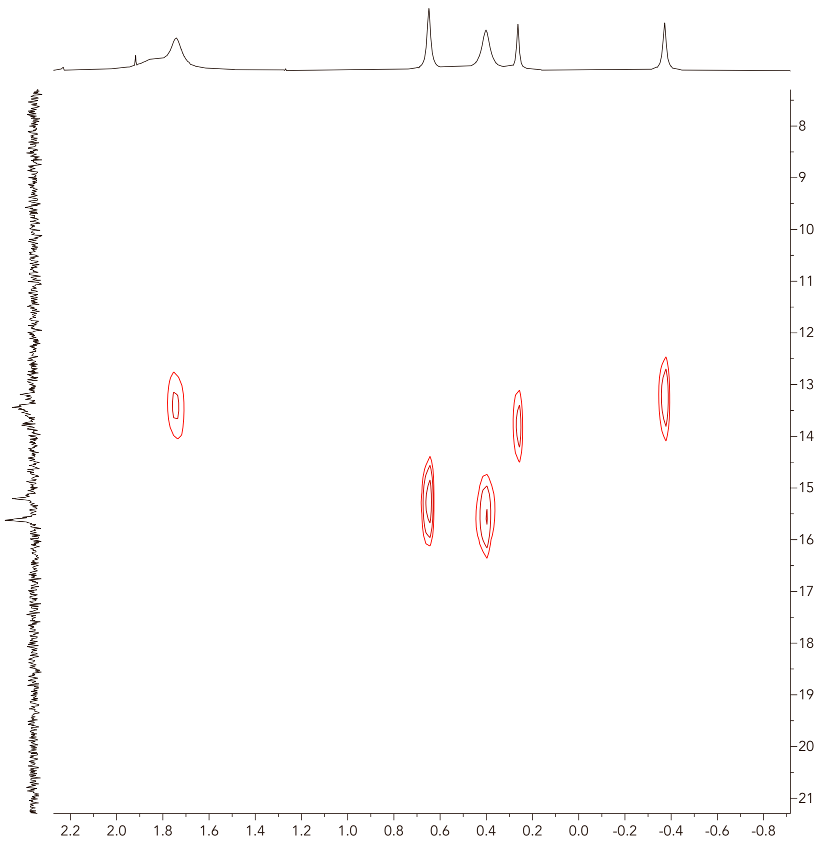


**Figure S70.** Partial $^{1}$H–$^{13}$C HSQC NMR spectrum of (**a2**·**b1**)⊂**C** (in the presence of ($\mathbf{a2}$)$_2$⊂**C**, ($\mathbf{b1}$)$_2$⊂**C**, and free **C**) (600 MHz, $D_2O$, 320 K).

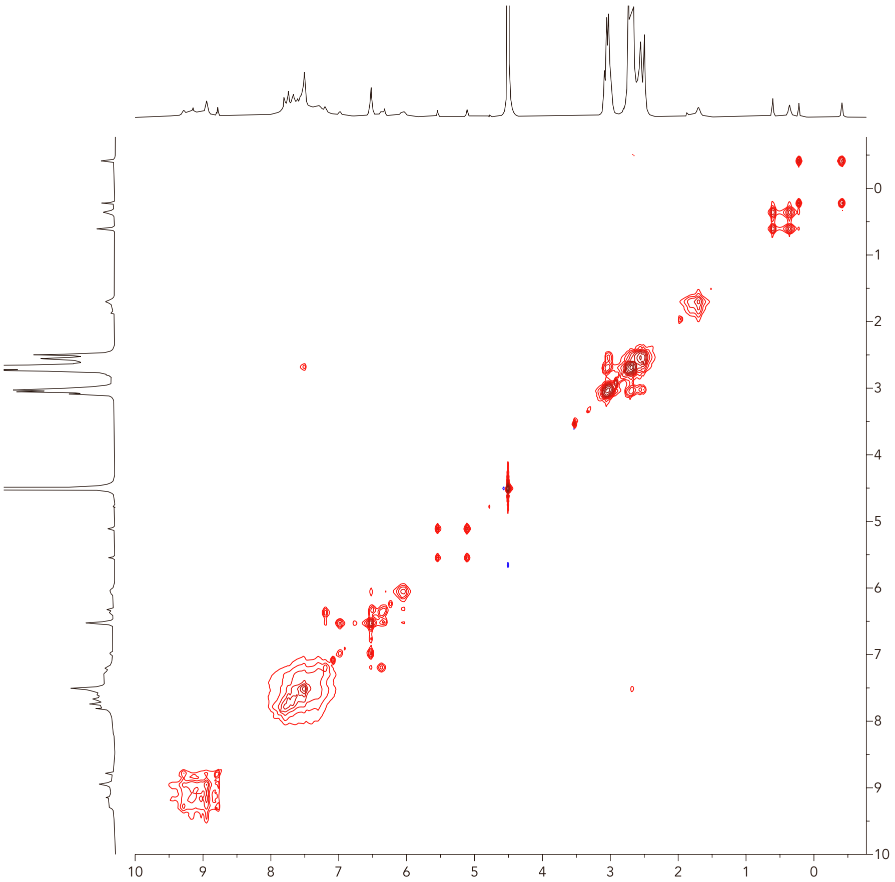


**Figure S71.** $^{1}$H–$^{1}$H NOESY NMR spectrum of (**a2**·**b1**)⊂**C** (in the presence of (**a2**)$_2$⊂**C**, (**b1**)$_2$⊂**C**, and free **C**) (600 MHz, $D_2O$, 320 K).

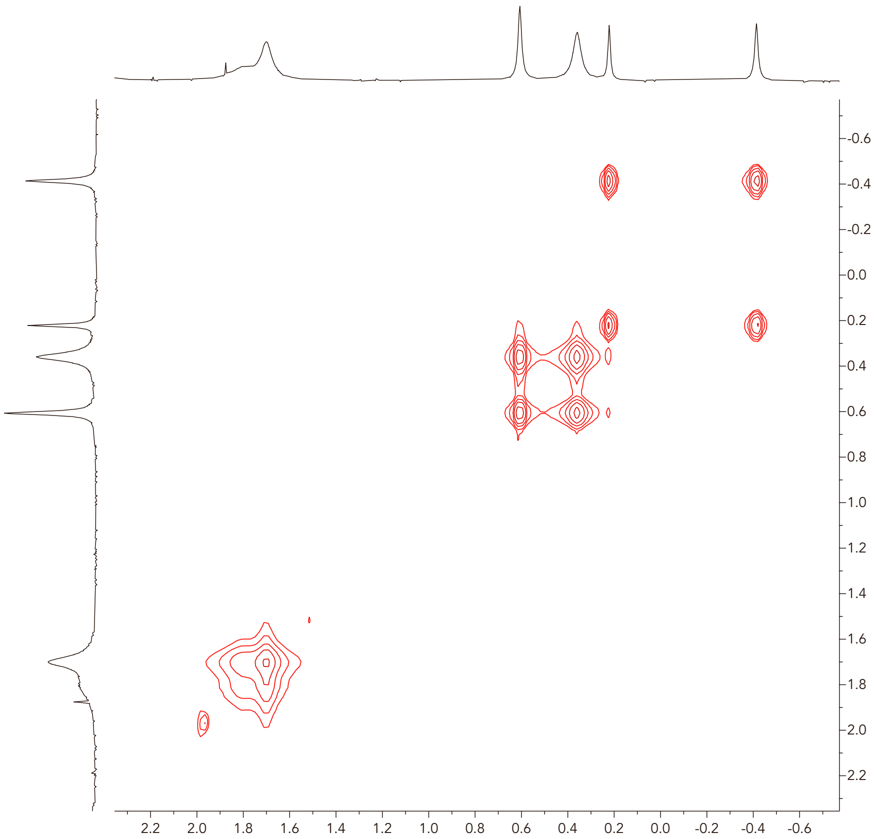


**Figure S72.** Partial $^{1}H$–$^{1}H$ NOESY NMR spectrum of (**a2**·**b1**)⊂**C** (in the presence of (**a2**)$_2$⊂**C**, (**b1**)$_2$⊂**C**, and free **C**) (600 MHz, $D_2O$, 320 K).

Heterodimeric inclusion complex (**a1**·**b2**)⊂**C** was obtained upon mixing homodimers (**a1**)$_2$⊂**C** and (**b2**)$_2$⊂**C** in a 1:1 ratio (with respect to the cage; note: the encapsulation yield for (**b2**)$_2$⊂**C** is ~60%, thus ~20% of the cage present in the system is unoccupied). The coexistence of the heterodimer with the (**a1**)$_2$⊂**C** homodimer (note: the spectrum does not show the presence of residual (**b2**)$_2$⊂**C**) resulted in a complex NMR spectrum; consequently, only characteristic peaks originating from the guests are listed below (guest protons with an apostrophe denote guests encapsulated within heterodimer (**a1**·**b2**)⊂**C**; those without an apostrophe refer to **a1** within homodimer (**a1**)$_2$⊂**C**):

$^1$H NMR (600 MHz, $D_2O$, 323 K); guest peaks: $\delta$ = 5.56 (s, **b2**$_{3'}$), 1.72 (s, **b2**$_{1'}$), 1.00 (s, **a1**$_{CH_3}$'), 0.26 (s, **b2**$_{2'}$), 0.13 (s, **a1**$_{CH_3}$).

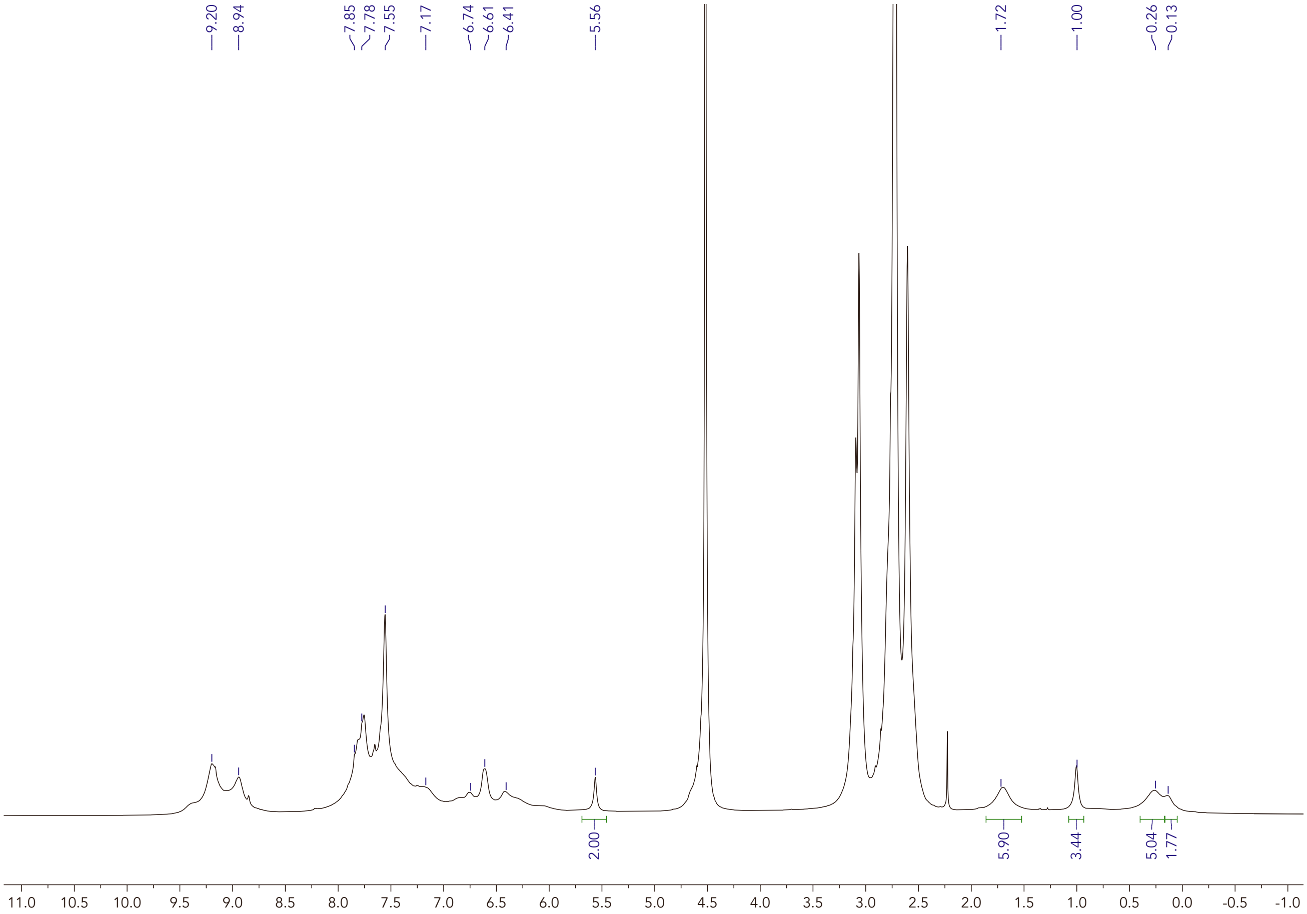


**Figure S73.** $^1$H NMR spectrum of (**a1**·**b2**)⊂**C** (in the presence of (**a1**)$_2$⊂**C** and free **C**) (500 MHz, $D_2O$, 323 K).

Heterodimeric inclusion complex (**a4·b4**)⊂**C** was obtained upon mixing homodimers (**a4**)$_2$⊂**C** and (**b4**)$_2$⊂**C** in a 1:1 ratio. The coexistence of the heterodimer with the two homodimers resulted in a complex NMR spectrum; consequently, only characteristic peaks originating from the guests are listed below (note: guest protons with an apostrophe denote guests encapsulated within heterodimer (**a4·b4**)⊂**C**; those without an apostrophe refer to guests within homodimer (**b4**)$_2$⊂**C** (Ref. 2)):

$^1$H NMR (600 MHz, $D_2O$, 328 K); guest peaks: $\delta$ = 1.84 (s, **b4$_1$**+**b4$_{1'}$**), 1.56 (s, **b4$_4$**), 1.51 (s, **b4$_{4'}$**), –0.05 (s, **b4$_2$**), –0.65 (s, **b4$_{2'}$**).

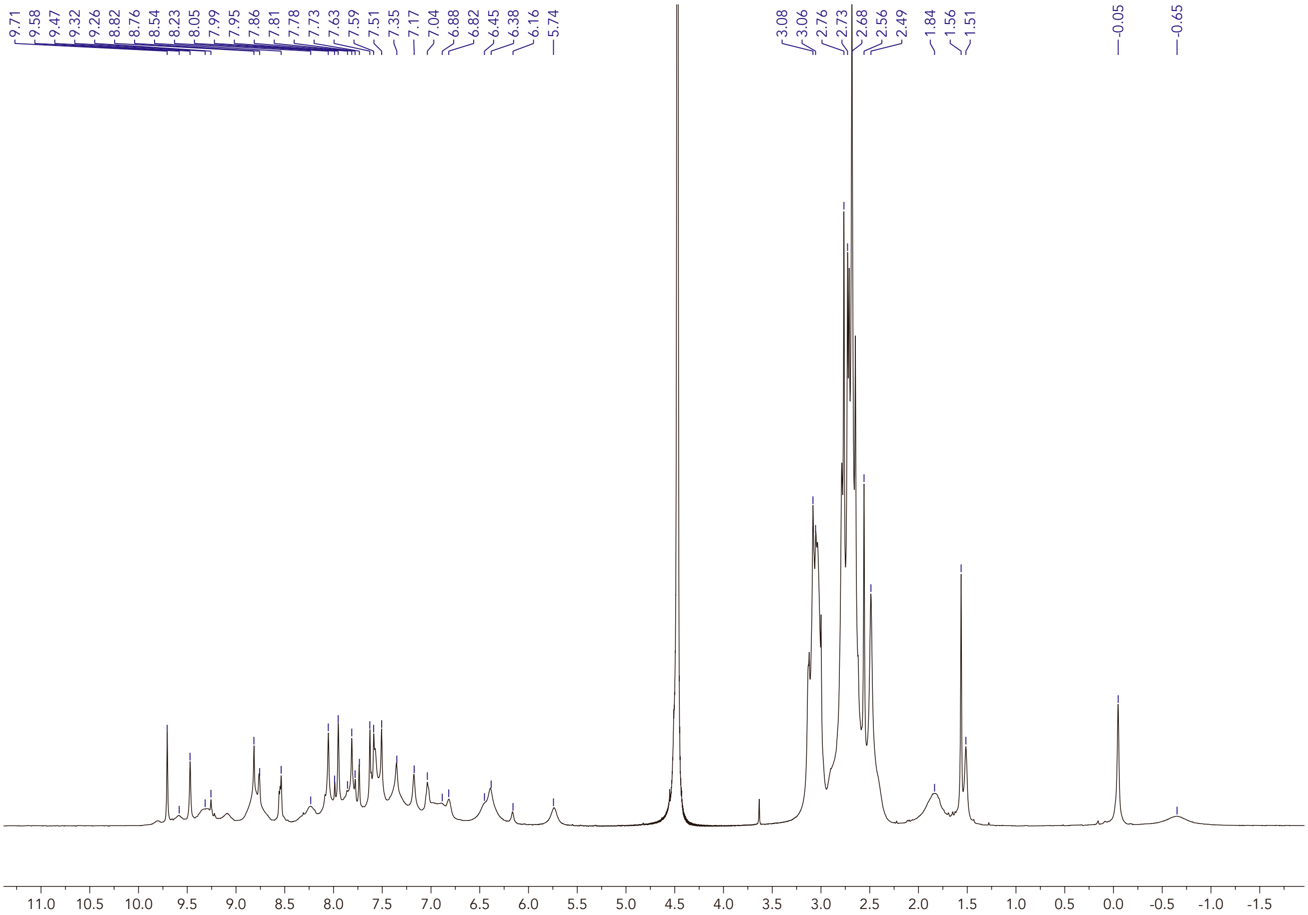


**Figure S74.** $^1$H NMR spectrum of (**a4·b4**)⊂**C** (in the presence of (**a4**)$_2$⊂**C** and (**b4**)$_2$⊂**C**) (500 MHz, $D_2O$, 328 K).

## 7. X-ray data collection and structure refinement

Single crystals of homodimer complexes (**a1**)$_2$⊂**C**, (**a2**)$_2$⊂**C**, (**a3**)$_2$⊂**C**, and (**a4**)$_2$⊂**C** were obtained by slow evaporation of water from the respective aqueous solutions. Single crystals of the (**a2**·**b1**)⊂**C** heterodimer were obtained after water evaporation from a mixture of (**a2**)$_2$⊂**C** and (**b1**)$_2$⊂**C** (a mixture of colorless and orange crystals was obtained; the latter were collected for further analysis). All crystals were coated in oil (as they tend to lose crystalline water quickly) and flash-frozen in a liquid nitrogen stream. Data were collected at either 80 K or 100 K. The diffraction data for (**a1**)$_2$⊂**C** and (**a2**)$_2$⊂**C** were collected on a Rigaku XtaLAB PRO diffractometer equipped with a Dectris Pilatus S200K detector and processed with CrysAlisPRO. The diffraction data for (**a3**)$_2$⊂**C** and (**a2**·**b1**)⊂**C** were collected on a Rigaku XtaLAB Synergy-R DW equipped with a HyPix ARC 150º detector and processed with CrysAlis$^{PRO}$. The diffraction data of (**a4**)$_2$⊂**C** were collected on a Bruker APEX-II Kappa CCD diffractometer and processed with Bruker SAINT. The structures were solved by direct methods using SHELXT as implemented in Olex2 GUI.[4] All non-hydrogen atoms were further refined by SHELXL[5] with anisotropic displacement coefficients. Hydrogens were placed in calculated positions and refined in a riding mode.

The unit cell parameters of (**a2**·**b1**)⊂**C** are the same as for previously published (**b1**)$_2$⊂**C**,[2] however, with a significantly higher number of electron density maxima. The refinement of (**a2**·**b1**)⊂**C** required assigning the additional electron density maxima to the atoms of **a2** molecules (which occupied the positions of **b1** within some cages). The electron density maxima originating from bromine atoms were treated as anchors, with the remainder of the **a2** molecule restrained. Two recognized anthracene frames were freely refined to yield ~41% occupancy of the guest positions (50% would be expected for each **a2** and **b1** in an ideal heterodimer). This result means either that the crystal is composed of the heterodimer and **b1** homodimer units and/or the residual disordered **b1** positions remain unaccounted for. A more complicated disorder is also possible because free refinement of **a2** occupancy yielded 44%. Contributions from disordered solvent and/or counterion molecules were removed with the SQUEEZE protocol of Platon[6]/Olex2.[4] The crystal data and structure refinement data are summarized in Table S1.

| Species | (a1)$_2$⊂C | (a2)$_2$⊂C | (a3)$_2$⊂C | (a4)$_2$⊂C | (a2·b1)⊂C |
|---|---|---|---|---|---|
| CCDC No. | 2103596 | 2103597 | 2103576 | 2103598 | 2103577 |
| Formula* | $C_{126}H_{168}N_{46}O_{30}Pd_6$ | $C_{124}H_{162}Br_2N_{48}O_{36}Pd_6$ | $C_{126}H_{168}N_{46}O_{47}Pd_6$ | $C_{128}H_{166}N_{48}O_{37}Pd_6$ | $C_{118.9}H_{165.56}B_{0.85}Br_{0.78}F_{1.71}N_{47.71}O_{30}Pd_6$ |
| Molecular weight* | 3445.47 | 3699.23 | 3477.47 | 3607.48 | 3485.88 |
| Crystal system | Monoclinic | Monoclinic | Triclinic | Triclinic | Monoclinic |
| Space group | $P2_1/c$ | $P2_1/c$ | $P\bar{1}$ | $P\bar{1}$ | $P2_1/c$ |
| Crystal size (mm) | 0.15×0.09×0.08 | 0.20×0.13×0.02 | 0.23×0.03×0.02 | 0.20×0.20×0.16 | 0.46×0.31×0.28 |
| Crystal color and shape | Yellow tablet | Colorless plate | Colorless needle | Colorless prism | Orange block |
| Temperature (K) | 100 | 100 | 100 | 100 | 80 |
| Wavelength (Å) | 0.71073 | 1.54184 | 1.54184 | 0.71073 | 0.71073 |
| a (Å) | 15.1278(3) | 21.8482(2) | 18.6116(4) | 18.6926(6) | 15.07752(13) |
| b (Å) | 41.2674(9) | 29.3112(3) | 19.4974(4) | 18.8109(7) | 41.6864(4) |
| c (Å) | 15.2858(3) | 15.2471(1) | 23.2282(5) | 27.1571(9) | 15.37691(14) |
| α (°) | 90 | 90 | 83.0525(18) | 101.046(2) | 90 |
| β (°) | 95.371(2) | 91.592(1) | 82.6694(18) | 100.108(2) | 95.1968(8) |
| γ (°) | 90 | 90 | 85.9915(18) | 90.272(2) | 90 |
| Volume (Å$^3$) | 9500.8(3) | 9760.43(15) | 8286.0(3) | 9219.4(6) | 9625.08(15) |
| Z | 2 | 2 | 2 | 1 | 2 |
| $r_{calcd}$ (g·cm$^{-1}$) | 1.204 | 1.259 | 1.394 | 1.299 | 1.203 |
| μ (mm$^{-1}$) | 0.623 | 5.436 | 5.801 | 0.648 | 0.778 |
| No. of reflections (unique) | 140752 (25542) | 303369 (19930) | 105235 (30069) | 293398 (37628) | 489814 (83039) |
| $R_{int}$ | 0.0512 | 0.1333 | 0.1147 | 0.0635 | 0.0440 |
| Completeness to θ (%) | 99.6 | 99.9 | 99.6 | 99.7 | 99.8 |
| Data / restraints / parameters | 25542 / 178 / 920 | 19930 / 97 / 1009 | 30069 / 50 / 1957 | 37628 / 171 / 2009 | 83039 / 1231 / 1340 |
| Goodness-of-fit on $F^2$ | 1.053 | 1.043 | 1.041 | 1.067 | 1.042 |
| Final $R_1$ and $wR_2$ indices [$I$>2s($I$)] | 0.0743, 0.1751 | 0.0751, 0.2004 | 0.0937, 0.2302 | 0.0679, 0.1546 | 0.0836, 0.2528 |
| $R_1$ and $wR_2$ indices (all data) | 0.0840, 0.1794 | 0.0805, 0.2065 | 0.1311, 0.2512 | 0.0940, 0.1685 | 0.1722, 0.2899 |

**Table S1.** Crystallographic data. (*Derived from the crystal structure; masked molecules not included)

Table S2 reports the structural parameters of cage **C** (empty and with guests **a1**, **a2**, **a3**, **a4**, **b1**, **b2**, and **b4**; crystal structures of **C**, (**b1**)$_2$⊂**C**, (**b2**)$_2$⊂**C** and (**b4**)$_2$⊂**C** were reported previously[2,7]) (note that whereas single crystals of inclusion complexes (**a1**)$_2$⊂**C** and (**a2**)$_2$⊂**C** contained a single conformation within one unit cell; those of (**a3**)$_2$⊂**C** and (**a4**)$_2$⊂**C** contained two different conformations of the inclusion complex).

| Species | $Pd_{ax}$–$Pd_{ax}$ distance | $Pd_{eq}$–$Pd_{eq}$ distance | Angle at $Pd_{ax}$ |
|---|---|---|---|
| **C** | 16.86 Å | 18.23 Å | 88.59° |
| (**b1**)$_2$⊂**C** | 18.44 Å | 16.84 Å | 76.38° |
| (**b2**)$_2$⊂**C** | 18.42 Å | 16.91 Å | 76.89° |
| (**b4**)$_2$⊂**C** | 18.16 Å | 17.09 Å | 78.65° |
| (**b4**)$_2$⊂**C′** | 18.59 Å | 16.70 Å | 75.37° |
| (**a1**)$_2$⊂**C** | 18.57 Å | 16.61 Å | 74.72° |
| (**a2**)$_2$⊂**C** | 17.65 Å | 17.55 Å | 82.10° |
| (**a3**)$_2$⊂**C** | 18.62 Å | 16.34 Å | 82.69° |
| (**a3**)$_2$⊂**C′** | 18.72 Å | 16.52 Å | 74.13° |
| (**a4**)$_2$⊂**C** | 17.70 Å | 17.37 Å | 81.79° |
| (**a4**)$_2$⊂**C′** | 18.32 Å | 16.76 Å | 76.77° |

**Table S2.** Structural parameters for empty **C** and **C** encapsulating different guests. $Pd_{ax}$ and $Pd_{eq}$ denote axial and equatorial palladium nodes, respectively. "$Pd_{ax}$–$Pd_{ax}$ distance" is defined as the distance between two axial palladium nodes; "$Pd_{eq}$–$Pd_{eq}$ distance" is defined as the average distance between two opposite equatorial palladium nodes. "Angle at $Pd_{ax}$" is defined as the angle between two triimidazole ligand planes at the axial palladium. The distances and angles were measured in Mercury 4.2.0 software.

Table S3 reports the structural parameters for crystals of **a1**–**a4** as small molecules vs. homodimeric complexes within cage **C**. Analogous parameters for **b1**, **b2**, and **b4** were reported previously.[2] Structural parameters for free **a1**, **a2**, **a3**, and **a4** were extracted from the literature (Refs. 8, 9, 10, and 11, respectively).

| Species | Plane-to-plane distance | Center-to-center distance | Orientation |
|---|---|---|---|
| (**a1**)$_2$⊂**C** | 3.54 Å | 5.30 Å | antiparallel |
| (**a1**)$_\infty$ | 3.53 Å | 3.87 Å | antiparallel |
| (**a2**)$_2$⊂**C** | 3.54 Å | 3.92 Å | antiparallel |
| (**a2**)$_\infty$ | 3.46 Å | 3.94 Å | parallel |
| (**a3**)$_2$⊂**C** | 3.45 Å | 3.85 Å | antiparallel |
| (**a3**)$_2$⊂ **C′** | 3.46 Å | 3.95 Å | antiparallel |
| (**a3**)$_\infty$ | 3.37 Å | 5.21 Å | parallel |
| (**a4**)$_2$⊂**C** | 3.46 Å | 4.08 Å | – |
| (**a4**)$_2$⊂ **C′** | 3.42 Å | 4.37 Å | – |
| (**a4**)$_\infty$ | 3.53 Å | 3.95 Å | – |

**Table S3.** Structural parameters for **a1**, **a2**, **a3**, and **a4** within single crystals, i.e., **a**$_\infty$, and inside cage **C**, i.e., (**a**)$_2$⊂**C**. To calculate plane-to-plane distances between two neighboring guests, individual planes were defined as the planes formed by the central ring or, in the case of **a4**, the two central fused rings. For calculating the center-to-center distances of **a1**, **a2**, and **a3**, centroids of the central rings were calculated. For **a4**, the centroid was defined as the middle point of the **a4**$_5$–**a4**$_5$ bond.

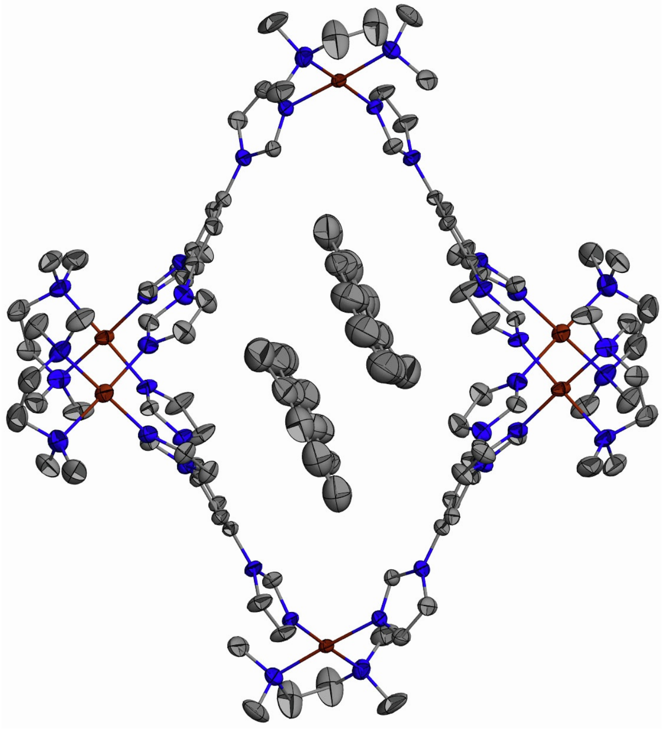

**Figure S75.** ORTEP representation of the X-ray structure of inclusion complex (**a1**)$_2$⊂**C** (displacement ellipsoids at a 50% probability level). Hydrogens, anions, and solvent molecules were eliminated for clarity. Pd, brown; C, gray; N, blue.

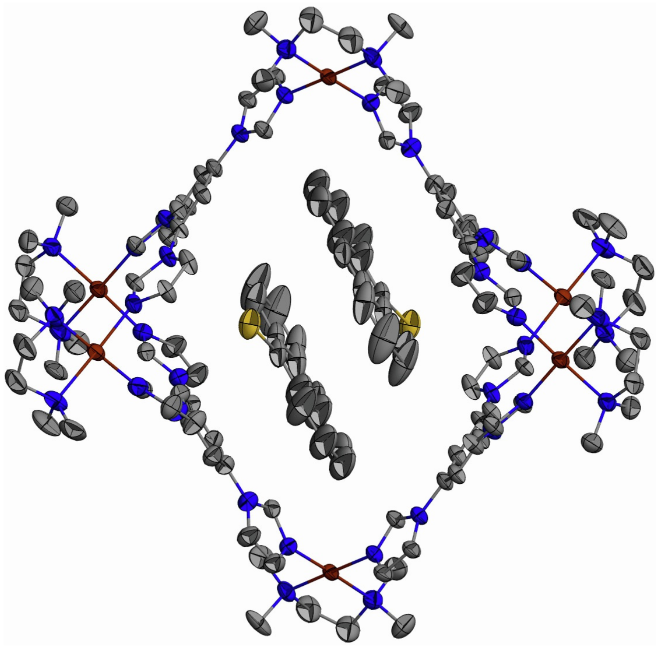

**Figure S76.** ORTEP representation of the X-ray structure of inclusion complex (**a2**)$_2$⊂**C** (displacement ellipsoids at a 50% probability level). Hydrogens, anions, and solvent molecules were eliminated for clarity. Pd, brown; C, gray; N, blue; Br, yellow.

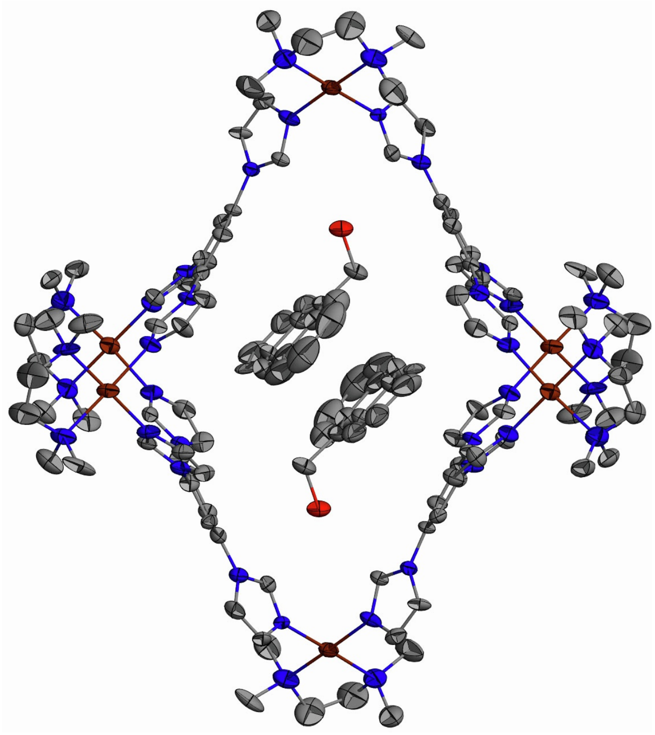

**Figure S77.** ORTEP representation of the X-ray structure of one of two conformers of inclusion complex (**a3**)$_2$⊂**C** (displacement ellipsoids at a 50% probability level; the structure of the second conformer is very similar; see the CIF file).). Hydrogens, anions, and solvent molecules were eliminated for clarity. Pd, brown; C, gray; N, blue; O, red.

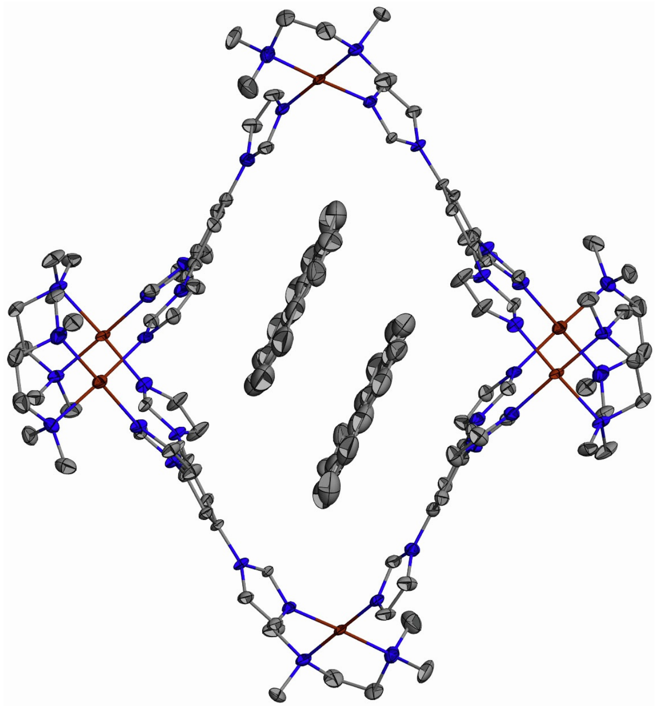

**Figure S78.** ORTEP representation of the X-ray structure of one of two conformers of inclusion complex (**a4**)$_2$⊂**C** (displacement ellipsoids at a 50% probability level; the structure of the second conformer is very similar; see the CIF file). Hydrogens, anions, and solvent molecules were eliminated for clarity. Pd, brown; C, gray; N, blue.

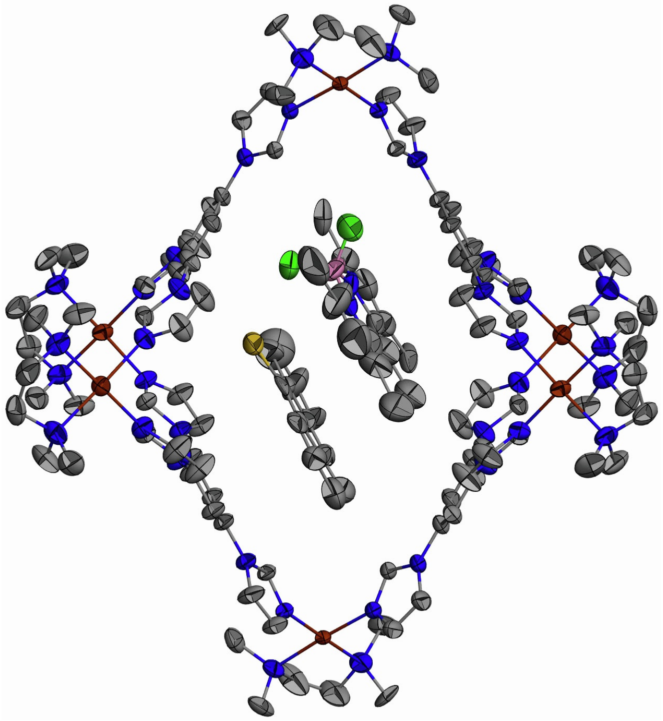

**Figure S79.** ORTEP representation of the X-ray structure of the first conformer of inclusion complex (**a2·b1**)⊂**C** (displacement ellipsoids at a 50% probability level). Hydrogens, anions, and solvent molecules were eliminated for clarity. Pd, brown; C, gray; N, blue; B, pink, F, green; Br, yellow.

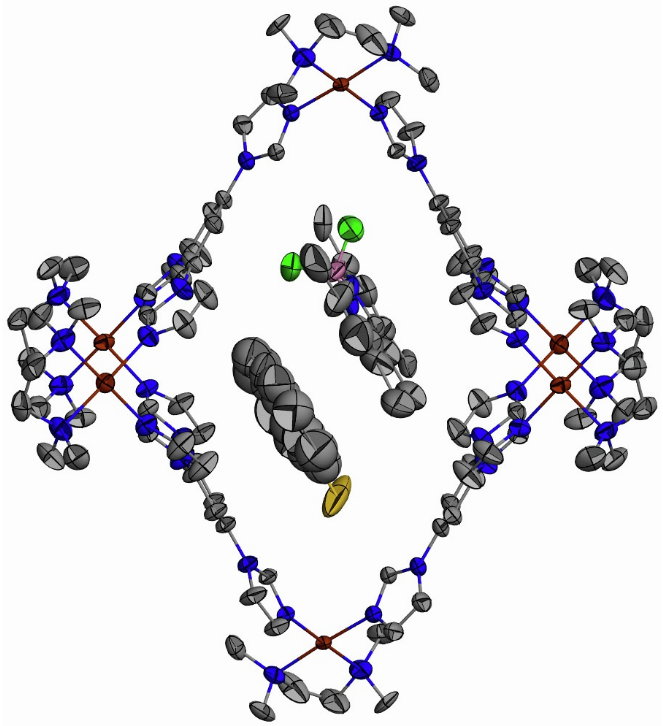

**Figure S80.** ORTEP representation of the X-ray structure of the second conformer of inclusion complex (**a2·b1**)⊂**C** (displacement ellipsoids at a 50% probability level). Hydrogens, anions, and solvent molecules were eliminated for clarity. Pd, brown; C, gray; N, blue; B, pink, F, green; Br, yellow.

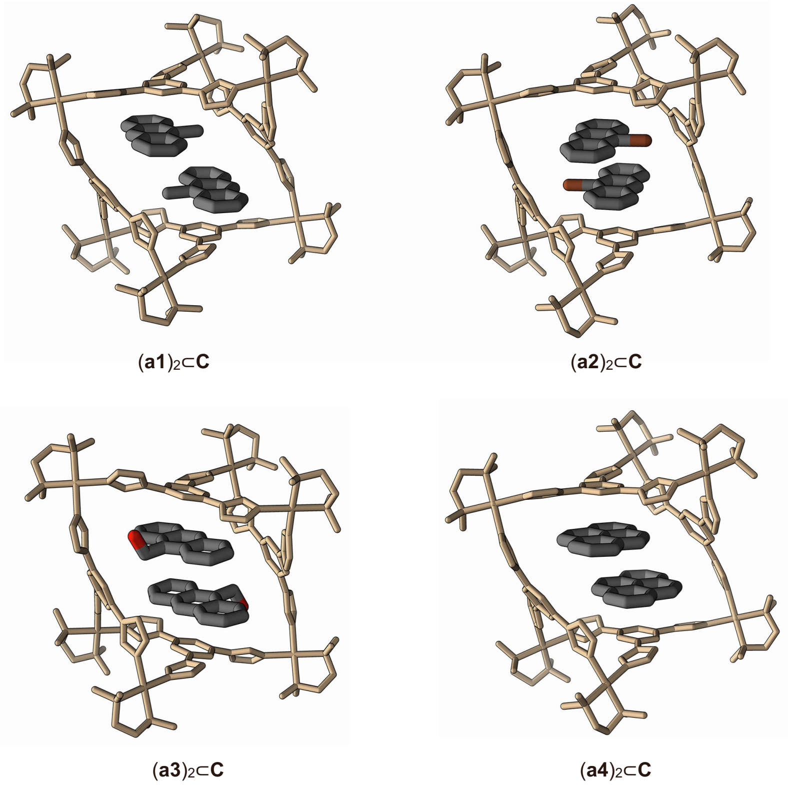


**Figure S81.** Comparison of the X-ray structures of (**a1**)$_2$⊂**C**, (**a2**)$_2$⊂**C**, (**a3**)$_2$⊂**C**, and (**a4**)$_2$⊂**C** (note that the X-ray structures of both (**a3**)$_2$⊂**C** and (**a4**)$_2$⊂**C** contained two slightly different conformations in a 1:1 ratio; only one of each is shown here; for the other conformation, see the CIF files).

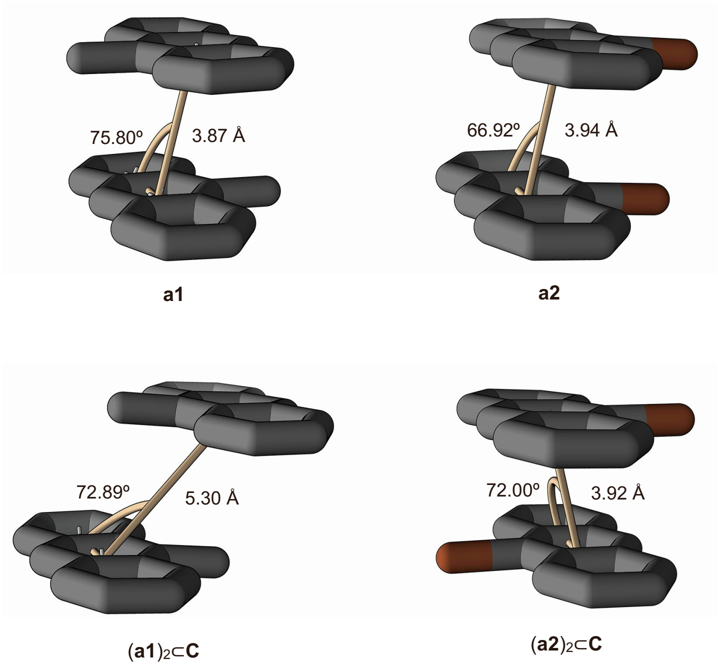


**Figure S82.** Comparison of the X-ray structures of **a1** and **a2** as free molecules (top) and encapsulated by **C** (bottom). The center-to-center distances and slip angles for free **a1** and **a2** are taken from Refs. 8 and 9, respectively.

## 8. DFT calculations of the (**a2**·**b1**)⊂**C** heterodimer

To verify the feasibility of two different arrangements of the **b1** guest within the heterodimer, DFT calculations were performed at the B3LYP-D3/6-31G(d,p)/LANL2DZ(Pd) level of theory[12,13] using the Gaussian 16[14] software. The structures were optimized as dications to preserve the symmetric distribution of counterions, with the initial geometries and distributions of nitrates as in the crystal structure. No imaginary frequencies were found, which confirmed achieving the energetic minima. The energy difference (with zero-point correction included) between the optimized structures (5.1 kcal/mol) suggests that the isomer with **a2**'s Br atom pointing towards the axial Pd (Figure S83B) is slightly more stable (the energy gap could be reduced by including the solvent (water) in the optimization process). Both complexes show some degree of nonplanarity of the guests under energy-optimized conditions, in contrast to restrained models from X-ray diffraction analysis.

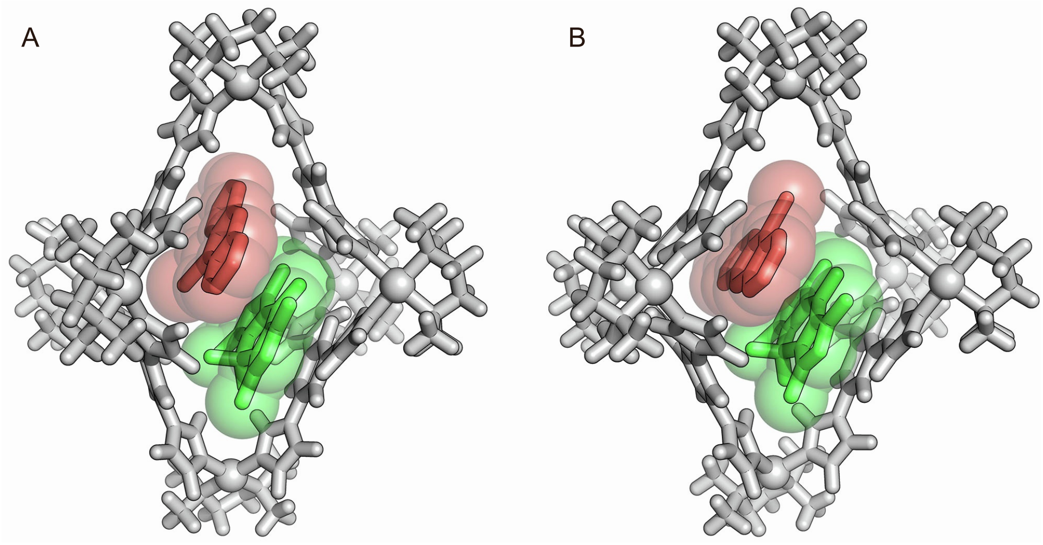


**Figure S83.** Energy-optimized structures of the two isomers of the (**a2**·**b1**)⊂**C** heterodimer.

## 9. Steady-state optical properties of **a1**, **a2**, **a3**, and **a4** and their inclusion complexes

Unless reported otherwise, UV-vis absorption measurements were carried out on 0.031 mM solutions of inclusion complexes (the concentration in terms of cage units) at ambient temperature. The solution of free cage **C** is transparent in the visible range, with an absorption onset at ~380 nm. The optical properties of BODIPYs **b1**, **b2**, and **b4** dissolved in MeCN or encapsulated within **C** in water were discussed previously.[2]

Solutions of **a1**, **a2**, **a3**, and **a4** in MeCN are colorless, but their UV-vis absorption spectra all show characteristic absorbance patterns in the near-UV area (between 300 nm and 400 nm; see the red traces in Figure S84). Compounds **a1** and **a2** exhibit similar spectra with a 'finger'-type pattern typical for various anthracene derivatives.[15] Peak maxima of **a1** in MeCN can be found at 348 nm, 366 nm, and 386 nm; those for **a2** appear at 350 nm, 368 nm, and 388 nm, and those for **a3** – at 349 nm, 367 nm, and 387 nm. The spectrum of **a4** in MeCN is dominated by two sharp absorption bands in the near-UV region, centered at 318 nm and 335 nm.

Upon encapsulation, the characteristic shapes of the absorption spectra of the guests are retained, albeit the peaks are slightly broader and red-shifted (see the black spectra in Figure S84A–D). For $(\mathbf{a1})_2\subset\mathbf{C}$, peak maxima are located at 357 nm, 376 nm, and 397 nm; for $(\mathbf{a2})_2\subset\mathbf{C}$, at 361 nm, 380 nm, and 401 nm; for $(\mathbf{a3})_2\subset\mathbf{C}$, at 356 nm, 373 nm, and 393 nm; and for $(\mathbf{a4})_2\subset\mathbf{C}$, at 332 nm and 348 nm. These changes can be attributed to a combination of solvatochromic and confinement effects (i.e., the proximity of the π systems of the encapsulated guests and the cage's walls).

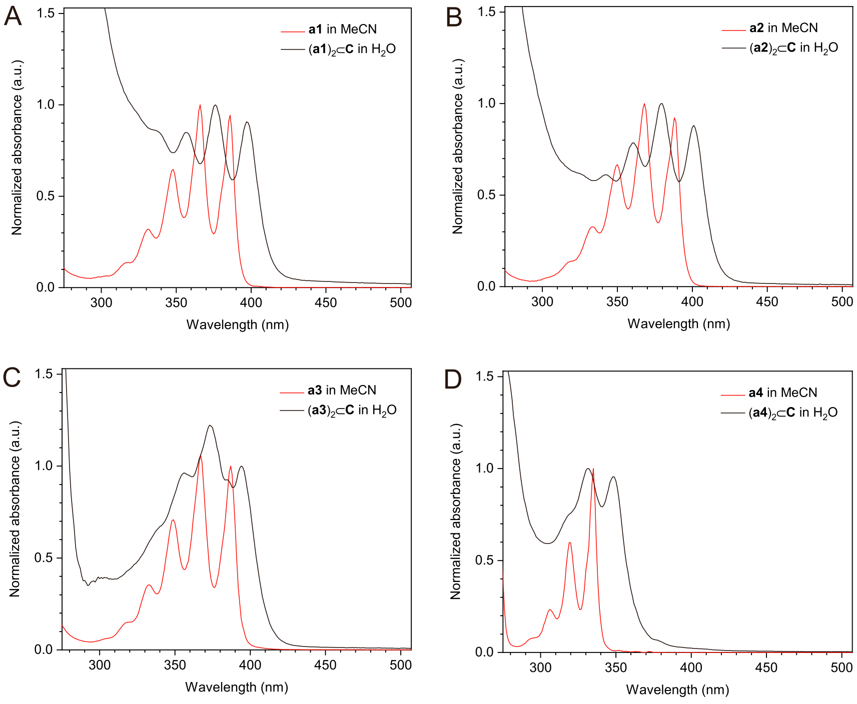


**Figure S84.** Normalized absorption spectra of (A) **a1**, (B) **a2**, (C) **a3**, and (D) **a4** encapsulated in **C** (black) or free in MeCN (red).

Notably, the extinction coefficients $\varepsilon$ of the guests decrease strongly upon encapsulation. This effect can be visualized by adding MeCN to the aqueous solutions of the complexes, as exemplified for $(\mathbf{a2})_2{\subset}\mathbf{C}$ and $(\mathbf{a4})_2{\subset}\mathbf{C}$ in Figure S85 (which plots normalized absorption). As shown previously for BODIPY dyes,[2] adding polar organic solvents to aqueous solutions of inclusion complexes drastically decreases the affinity of guests to host **C**, resulting in guest release. By monitoring the absorption spectra during titration with MeCN, gradual release of guests can be followed conveniently (Figure S85C).

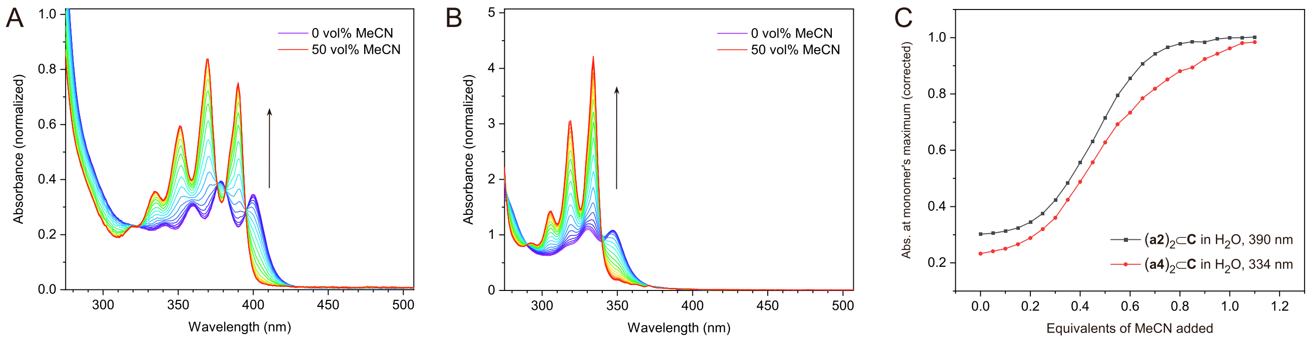


**Figure S85.** Release of **a2** from $(\mathbf{a2})_2{\subset}\mathbf{C}$ (A) and **a4** from $(\mathbf{a4})_2{\subset}\mathbf{C}$ (B) by stepwise addition of MeCN. The absorbance was normalized with respect to the volume of MeCN added. (C) The gradual release of **a2** and **a4** from cage **C** in the presence of increasing amounts of MeCN (molar equivalents MeCN with respect to water).

While **a1**–**a4** are relatively strong fluorophores (emitting in the near-UV and blue region), their fluorescence is mostly quenched upon encapsulation within **C**. Figure S86 (panels A–D) compares the emission spectra of free **a1**–**a4** in DCM with the spectra of encapsulated **a1**–**a4**; in all cases, concentrations of the free and encapsulated guest were the same.

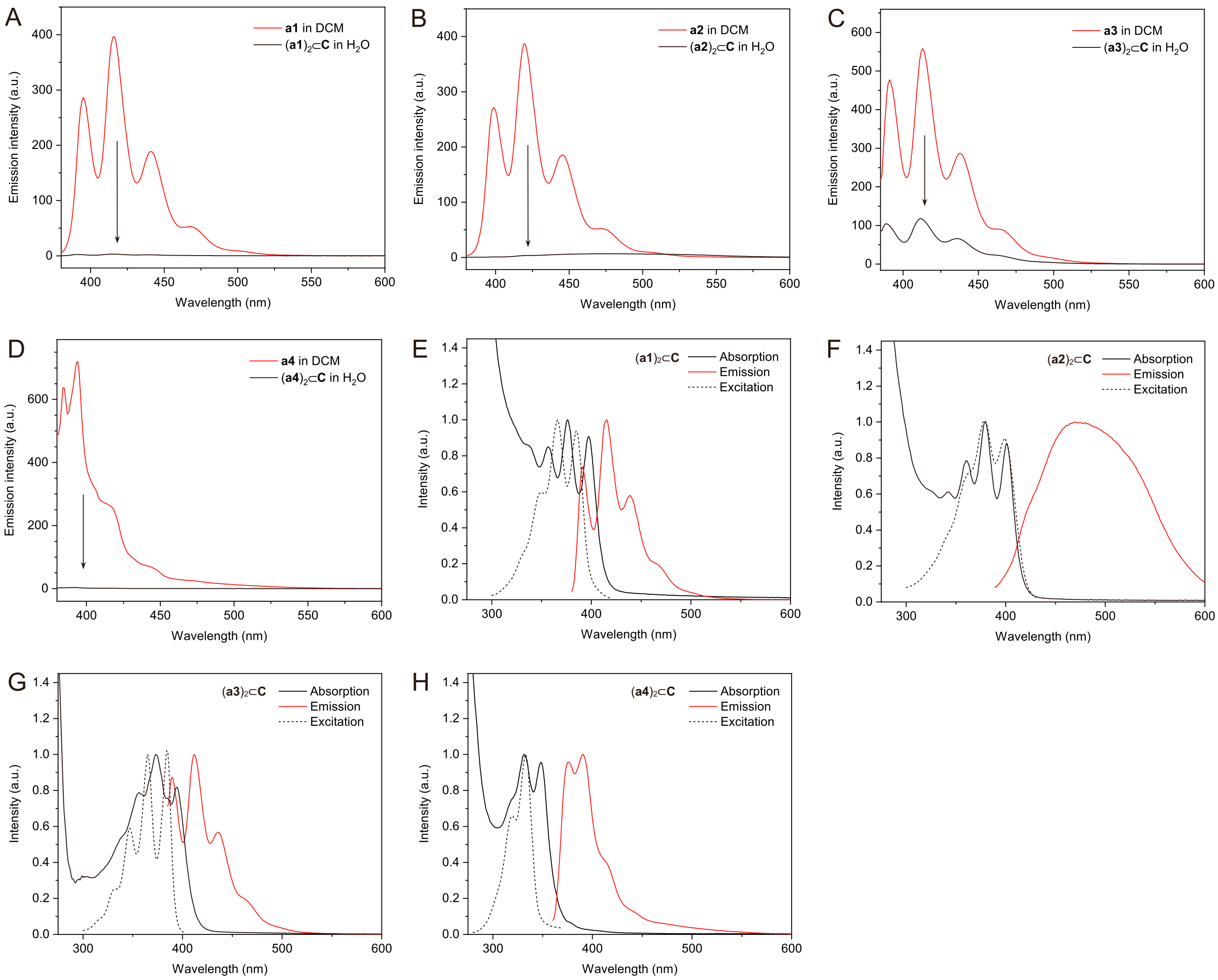


**Figure S86.** (A–D) Emission spectra of **a1**, **a2**, **a3**, and **a4** dissolved in DCM (red) or as homodimers within **C** in water (black) ($\lambda_{exc}$ = 380 nm for **a1**– **a3**; $\lambda_{exc}$ = 340 nm for **a4**). In all cases, the concentration of the guest was the same (95 μM). The emission observed for (**a3**)$_2$⊂**C** (panel C) originates from a small amount of unencapsulated **a3** in equilibrium with (**a3**)$_2$⊂**C**; note that this anthracene has a relatively good solubility in water. (E–H) Normalized absorption, emission, and excitation spectra for **a1**–**a4** after encapsulation within **C** ($\lambda_{exc}$ = 380 nm for **a1**–**a3**; $\lambda_{exc}$ = 340 nm for **a4**).

## 10. Formation of heterodimeric inclusion complexes and their steady-state optical properties

Heterodimers comprising both **a** (**a1**, **a2**, **a3**, or **a4** and **b** (**b1**, **b2**, **b3**, or **b4**) were formed by mixing aqueous solutions of the respective homodimers. Depending on the guest's identity, heterodimers formed either instantly or over periods of up to several minutes. We confirmed that all sixteen possible **a·b** heterodimer combinations could be obtained by mixing the respective homodimers; several representative examples were characterized in detail and are discussed below.

The initial experiments were based on the **a2** + **b1** combination. First, we prepared two solutions of cage **C** (0.5 mL; $c$ = 1.57 μM) and saturated them with guests **a2** and **b1**, respectively (note that the uptake of **b1** is limited to ~50% of cages). The resulting spectra of (**a2**)$_2$⊂**C** and (**b1**)$_2$⊂**C**, after removing excess (unbound) guests, are shown as the blue trace in Figure S87A and the red trace in Figure S87B, respectively.

As reported previously[2], the complex (**b1**)$_2$⊂**C** shows a strong absorption band centered at 480 nm, which originates from the S$_1$←S$_0$ transition of **b1** within the homodimer. Compared with dye **b1** dissolved in MeCN, this band is blue-shifted by 11 nm due to H-aggregation (in this case, H-dimerization) resulting from noncovalent complexation within **C**. For the same reason, the emission band is red-shifted (from 502 nm for **b1** in MeCN to 544 nm, giving rise to a significant Stokes shift of 64 nm, compared with 11 nm for **b1** in MeCN).[2]

Next, the (**a2**)$_2$⊂**C** solution was treated with solid **b1** (10 eq with respect to **C**). The mixture was stirred overnight, the solids were removed by filtration, and the supernatant was analyzed by UV-vis absorption spectroscopy. The resulting spectrum (Figure S87A) showed encapsulated **a2** and a new peak centered at 511 nm. This result demonstrates the ability of **b1** to partially displace **a2** from (**a2**)$_2$⊂**C**. This new 511 nm peak is distinct from the absorption of (**b1**)$_2$⊂**C** (red in Figure S87B) and it can be attributed to the (**a2·b1**)⊂**C** heterodimer.

In the reverse experiment, we treated the solution of (**b1**)$_2$⊂**C** with 10 eq of solid **a2** (also 10 eq is with respect to **C**). We observed that the white solid **a2** turned red, indicating the expulsion of **b1** from the cage, followed by its precipitation from water. After stirring overnight and filtration, a spectrum similar to that in the previous experiment was obtained in that the ratios of absorbance at ~510 nm and ~380 nm were similar (compare the green spectrum in Figure S87A to the black spectrum in Figure S87B; note the different scales on the $y$-axes). The similarities of the final spectra, despite the different ratios of the two guests (1:10 vs. 10:1), suggest that the (**a2·b1**)⊂**C** heterodimer forms more preferentially than either of the homodimers.

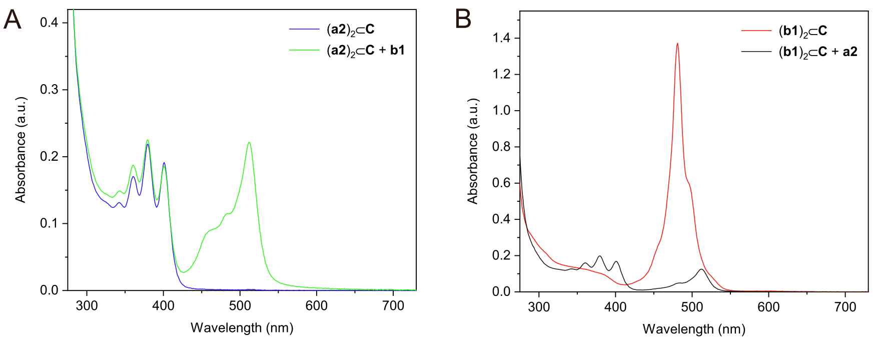


**Figure S87.** (A) UV-vis absorption spectrum of an aqueous solution of (**a2**)$_2$⊂**C** before (blue) and after (green) stirring with 10 eq of **b1** overnight. (B) UV-vis spectrum of an aqueous solution of (**b1**)$_2$⊂**C** before (red) and after (black) stirring with 10 eq of **a2** overnight.

Next, we proceeded to titration experiments. Titrating an aqueous solution of (**b1**)$_2$⊂**C** with (**a2**)$_2$⊂**C** results in changes in the visible part of the UV-vis absorption spectrum (Figure S88A; note that (**a2**)$_2$⊂**C** is transparent in the visible region, with no absorption above 420 nm). While the main absorbance band at 480 nm (due to the homodimer (**b1**)$_2$⊂**C**) gradually decreases, a new absorption peak centered at 511 nm appears (Figure S88A). The intensities of both bands are similar at ~1.5 eq of (**a2**)$_2$⊂**C** added (Figure S88B). Upon adding 4 eq (**a2**)$_2$⊂**C**, the 511 nm band prevails, with the original 480 nm band visible as a shoulder (Figure S88A).

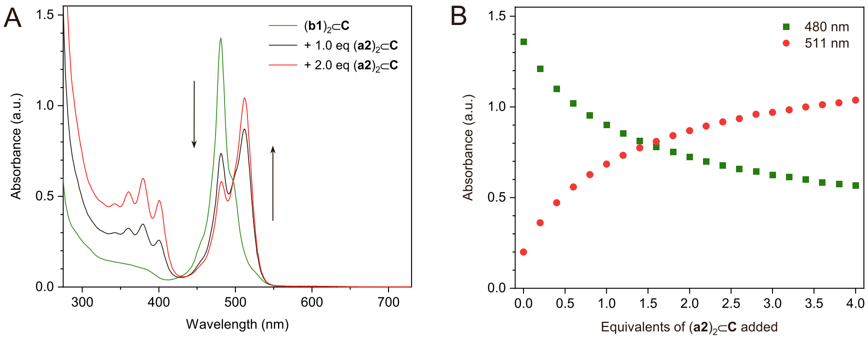


**Figure S88.** (A) Titration of (**b1**)$_2$⊂**C** with (**a2**)$_2$⊂**C** followed by UV-vis absorption spectroscopy. (C) Absorbance of 480 nm and 511 nm followed during the titration plotted in A.

We also followed the titration by fluorescence spectroscopy. Inclusion complex (**b1**)$_2$⊂**C** exhibits an unusually weak and broad emission band (in contrast to the sharp emission of free BODIPY dyes), with an emission maximum at 544 nm and the fluorescence quantum yield $\Phi_F$ of 0.13.[2] Upon adding (**a2**)$_2$⊂**C**, we observed the appearance and increase of a relatively sharp and intense emission band centered at 528 nm, which can be attributed to (**a2·b1**)$_2$⊂**C**. The $\Phi_F$ for a 1:20 mixture of (**b1**)$_2$⊂**C** and (**a2**)$_2$⊂**C** was determined to be 0.21, which is a significant increase compared with $\Phi_F = 0.13$ for (**b1**)$_2$⊂**C**, although still far from the strong fluorescence of **b1** in organic solvents (e.g., $\Phi_F = 1.00$ for **b1** in $CHCl_3$[16]). We also note that the Stokes shift decreased from 64 nm for (**b1**)$_2$⊂**C** to 17 nm, much closer to the value for **b1** in MeCN (11 nm). Excitation spectra clearly showed that the strong emission was connected to the emergence of the new absorbance band at 511 nm (Figure S89A), a conclusion that can also be reached by following the emission at 528 nm and the absorption at 511 nm in parallel (see Figure S89B).

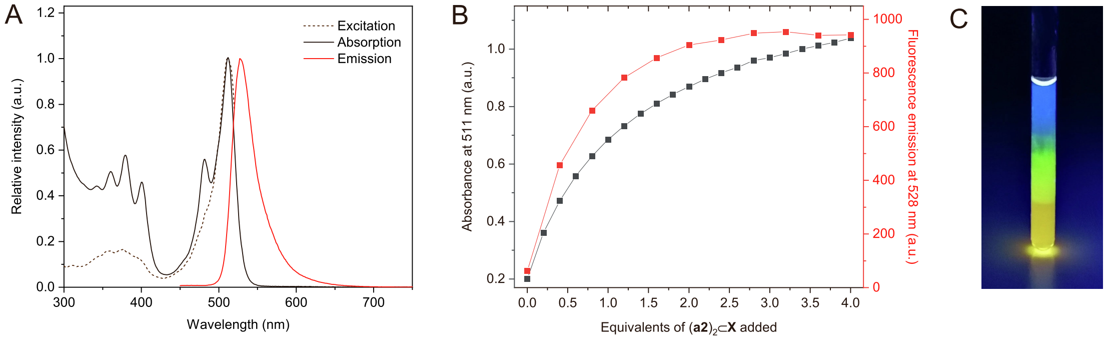


**Figure S89.** (A) UV-vis absorption spectrum (solid black line), excitation spectrum (dashed black line), and emission spectrum (red line, $\lambda_{exc}$ = 460 nm) of a 4:1 mixture of (**a2**)$_2$⊂**C** and (**b1**)$_2$⊂**C**. (B) Absorbance at 511 nm (black) and emission intensity at 528 nm (red, $\lambda_{exc}$ = 460 nm) followed during titration of (**b1**)$_2$⊂**C** with (**a2**)$_2$⊂**C**. (C) Photograph demonstrating the change in emission color during mixing of (**a2**)$_2$⊂**C** and (**b1**)$_2$⊂**C**. Initially, the tube contained an aqueous solution (**b1**)$_2$⊂**C** (bottom layer; weak yellow emission). Then, an aqueous

solution of (**a2**)$_2$⊂**C** was gently layered on top (weak blue emission). The middle layer forms as a result of mixing and an intense green emission due to the (**a2**·**b1**)$_2$⊂**C** heterodimer is observed.

Guest rearrangement between cages following mixing (**a2**)$_2$⊂**C** with (**b1**)$_2$⊂**C** can also be observed by the naked eye under ambient light. When (**a2**)$_2$⊂**C** (1 eq) was added to (**b1**)$_2$⊂**C** all at once, a rapid color change from dark orange to red-orange was observed. Under UV (365 nm) light excitation, the visual differences were even more pronounced: an immediate emission color change from weak yellow-orange to bright green occurred upon injecting (**a2**)$_2$⊂**C** into (**b1**)$_2$⊂**C** (see Figure 3A in the main text).

Similar results were observed upon treating (**b1**)$_2$⊂**C** with the other (**a**)$_2$⊂**C** complexes—(**a1**)$_2$⊂**C**, (**a3**)$_2$⊂**C**, and (**a4**)$_2$⊂**C**—indicating that the structure of anthracene/pyrene co-encapsulated with **b1** has little influence on its optical properties. These findings are in sharp contrast to those of Yoshizawa et al., who reported significant differences in the emission spectra of **b1** co-encapsulated with different aromatic hydrocarbons (e.g., phenanthrene and 9-methylanthracene) within a Pt-based coordination cage.[17]

Figure S90 shows the results of titration experiments in which aqueous solutions of (**b1**)$_2$⊂**C** were titrated with (**a1**)$_2$⊂**C**, (**a3**)$_2$⊂**C**, and (**a4**)$_2$⊂**C**. The visible-light regions of the UV-vis absorption spectra are very similar to each other and to that of (**b1**)$_2$⊂**C** titrated with (**a2**)$_2$⊂**C** (Figure 2A). Moreover, **b1**'s absorption and emission maxima (511 nm and 528 nm, respectively) remain unchanged for the different co-guests (**a1**, **a2**, **a3**, or **a4**).

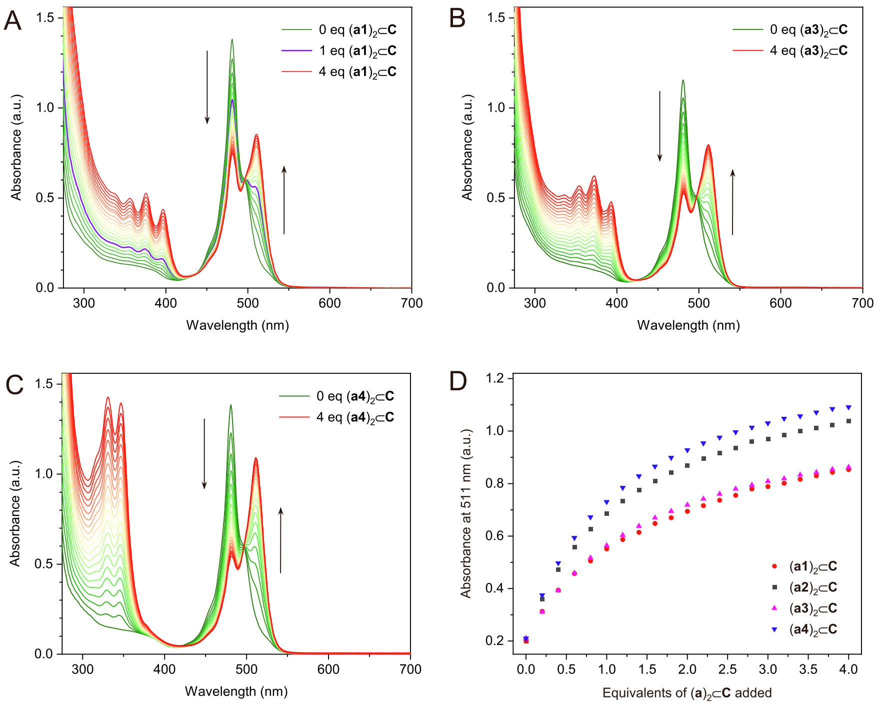


**Figure S90.** (A–C) UV-vis absorption spectra recorded during the titration of (**b1**)$_2$⊂**C** with: (A) (**a1**)$_2$⊂**C**, (B) (**a3**)$_2$⊂**C**, and (C) (**a4**)$_2$⊂**C**. (D) Comparison of all four titration experiments, whereby (**b1**)$_2$⊂**C** was treated with (**a1**)$_2$⊂**C**, (**a2**)$_2$⊂**C**, (**a3**)$_2$⊂**C**, or (**a4**)$_2$⊂**C**.

Despite the similar steady-state absorption and emission spectra, the fluorescence quantum yields $\Phi_F$ of the different heterodimer complexes varied significantly by changing **b1**'s partner within cage **C**. Specifically, we determined the values of $\Phi_F$ as 0.31 for (**a1**·**b1**)⊂**C**, 0.21 for (**a2**·**b1**)⊂**C**, 0.41 for (**a3**·**b1**)⊂**C**, and 0.50 for (**a4**·**b1**)⊂**C**. In addition, **b1**'s partners affected its transient fluorescence properties; see below (Section 12).

Analogous results were obtained with other BODIPY dyes. BODIPY **b2** is known for its ability to J-aggregate in mixtures of water and organic solvents.[16] Due to the electron-withdrawing $CF_3$ group in the *meso* position, the main absorbance band of **b2** dissolved in MeCN is red-shifted (compared to **b1**) to 548 nm. As reported previously,[2] dimerization of **b2** within **C** and the formation of ($\mathbf{b2}$)$_2$⊂**C** induced a blue-shift of the main absorption band to 523 nm due to H-aggregation (analogously to the behavior of **b1** within **C**).

Titrating an aqueous solution of ($\mathbf{b2}$)$_2$⊂**C** with an encapsulated anthracene (here, ($\mathbf{a1}$)$_2$⊂**C**) resulted in the appearance and growth of a red-shifted band at ~565 nm (Figure S91A). The spectrum obtained upon the addition of 3.2 eq of ($\mathbf{a1}$)$_2$⊂**C** (red in Figure S91A) shows little residual absorption at 523 nm, indicating a high tendency of **b2** (compared with, e.g., **b1**; see Figure S90A) to form a heterodimer with **a1**. These findings are in agreement with our NMR results (Figure S73), which similarly showed that the equilibrium in a near-stoichiometric mixture of ($\mathbf{a1}$)$_2$⊂**C** and ($\mathbf{b2}$)$_2$⊂**C** is shifted heavily in the direction of the heterodimer.

We have recently demonstrated that the controlled decomposition of cage **C** with KCN can be used to turn the H-dimers of **b2** (within ($\mathbf{b2}$)$_2$⊂**C**) into its J-aggregates.[2] The partial release of **b2** from the cage can also be induced by adding free **a1** (compare with Figure S87B). Indeed, upon titrating ($\mathbf{b2}$)$_2$⊂**C** with **a1** (dissolved in a small volume of MeCN), the formation of J-aggregates of **b2** was observed (see the sharp peak at >600 nm in Figure S91B and the yellow spectrum in Figure S91C). If the resulting solution is treated with 3 eq of free **C**, the expelled **b2** is re-encapsulated, and the >600 nm band disappears (green trace in Figure S91C). Thus, the above addition sequence allows us to obtain solutions with three distinct absorption features using a single dye **b2** by varying its aggregation mode.

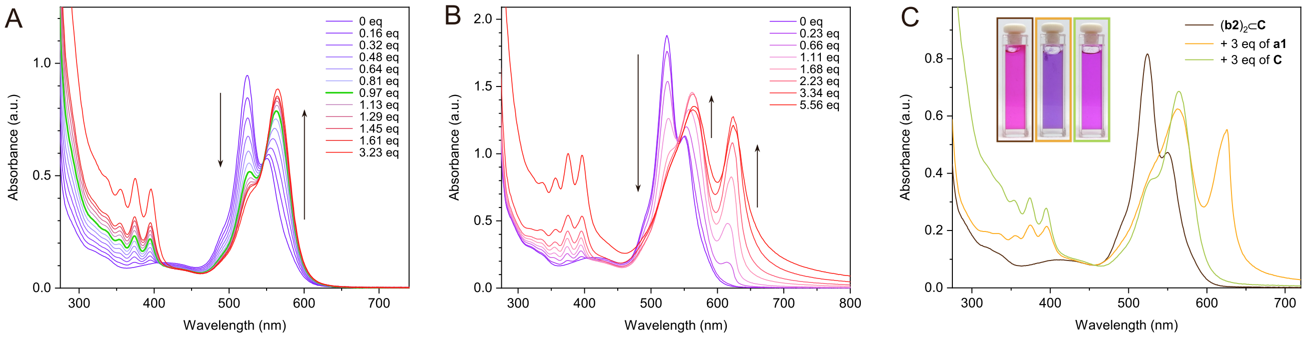


**Figure S91.** (A) UV-vis absorption spectra recorded during the titration of ($\mathbf{b2}$)$_2$⊂**C** with ($\mathbf{a1}$)$_2$⊂**C**. (B) UV-vis absorption spectra during the titration of aqueous ($\mathbf{b2}$)$_2$⊂**C** with **a1** in MeCN. (C) Photographs of solutions of **b2** in the three different aggregation states and the corresponding UV-vis absorption spectra.

Next, we focused on BODIPY **b3**. Similar to **b1**, **b3** has a sharp absorption peak at 516 nm in MeCN (slightly red-shifted compared to **b1** ($\lambda$ = 492 nm) due to two additional methyl groups). **b3** is highly emissive, with a sharp emission band centered at 531 nm (in MeCN; excited at 516 nm). Upon dimerization within **C**, we observed a blue-shift of the main absorption band of **b3** to 500 nm and significant emission quenching, with a red-shift of the emission band to 569 nm. These changes give rise to a Stokes shift of 69 nm (compared with 15 nm for **b3** in MeCN), in analogy with **b1**.

Upon treating an aqueous (**b3**)$_2$⊂**C** solution with any of the four (**a**)$_2$⊂**C** complexes, a new absorbance peak centered at 534 nm arose, accompanied by a decrease of the 500 nm band. Figure S92A shows the results of a representative titration of (**b3**)$_2$⊂**C** with (**a4**)$_2$⊂**C**. However, in contrast to titrations involving (**b1**)$_2$⊂**C**, where equilibration was instantaneous, it took many seconds for the spectra to stabilize after each injection of (**a4**)$_2$⊂**C** aliquot. When following the titration by fluorescence spectroscopy, the emission was found to increase dramatically, with a new and intense emission band centered at 549 nm (Figure S92B) (corresponding to a small Stokes shift of 15 nm). We found that the increase in emission intensity at 549 nm was strongly correlated with the increase in absorbance at 534 nm (Figure S92C).

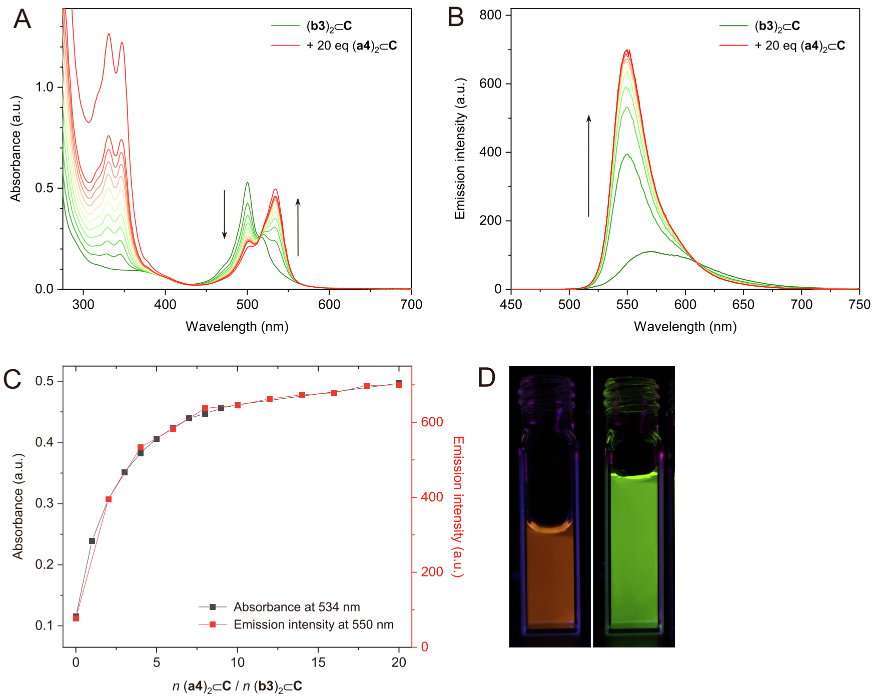


**Figure S92.** (A) UV-vis absorption spectra accompanying the titration of (**b3**)$_2$⊂**C** with (**a4**)$_2$⊂**C**. After each addition of the titrant, the spectra were allowed to stabilize before the next aliquot of the titrant was added. Here, the time interval between two consecutive additions of the titrant was 2 min. (B) Emission spectra accompanying the titration of (**b3**)$_2$⊂**C** with (**a4**)$_2$⊂**C** ($\lambda_{exc}$ = 480 nm). (C) Comparison of UV-vis absorption spectroscopy and emission spectroscopy results of the titration of (**b3**)$_2$⊂**C** with (**a4**)$_2$⊂**C**. (D) Photographs of (**b3**)$_2$⊂**C** before (left) and after (right) the addition of a caged anthracene (here, (**a1**)$_2$⊂**C**; i.e., a substantial amount of (**a1**·**b3**)⊂**C**) has formed). The photographs were under the same excitation conditions with $\lambda_{exc}$ = 365 nm.

To confirm that the observed changes in the optical properties are due to noncovalent interactions, we studied guest release from the cage using an organic solvent. To this end, (**a2**)$_2$⊂**C** and (**b1**)$_2$⊂**C** were first premixed in a 1:4 molar ratio and then titrated with MeCN. Before the titration, UV-vis absorption spectroscopy showed a prominent absorption peak at 511 nm (due to (**a2**·**b1**)⊂**C**), in addition to smaller peaks at 480 nm (due to (**b1**)$_2$⊂**C**), and the characteristic pattern due to anthracene in the near-UV region (purple trace in Figure S93A). The addition of MeCN resulted in a single absorption band in the visible area, centered at 491 nm (red trace in Figure S93A), which can be assigned to free **b1** solvated by MeCN. Similarly, the addition of MeCN induced the release of **a2** (characteristic peaks at 350, 368, and 388 nm; compare with Figure S85A). The same experiment was repeated with other (**a**)$_2$⊂**C** + (**b**)$_2$⊂**C** homodimer combinations (data not shown).

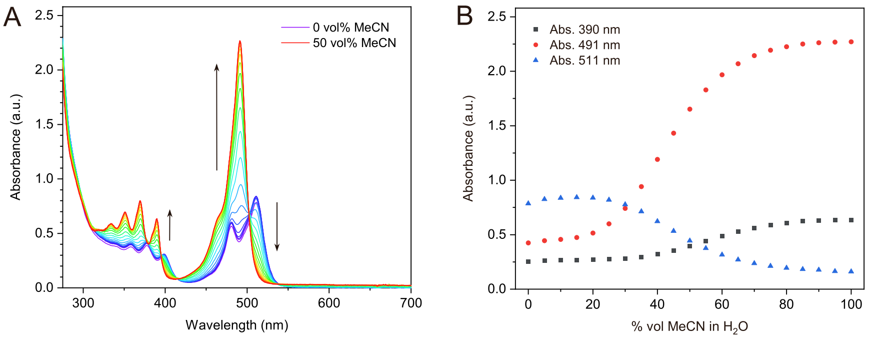


**Figure S93.** (A) UV-vis absorption spectra (concentration-corrected) recorded during stepwise addition of MeCN to a mixture of (**a2**)$_2$⊂**C** and (**b1**)$_2$⊂**C** to release free **a2** and **b1**. (B) Following the three characteristic wavelengths during the addition of MeCN to premixed (**a2**)$_2$⊂**C** and (**b1**)$_2$⊂**C**.

## 11. Kinetics of heterodimer formation

Upon mixing homodimeric inclusion complexes of **b1**, **b2**, **b3**, and **b4** with those of **a1**, **a2**, **a3**, and **a4**, heterodimeric complexes (**a**·**b**)⊂**C** formed spontaneously through guest exchange. The kinetics of heterodimer formation depended strongly on the identity of / substitution pattern on guests **a** and **b**.

In a typical experiment, 5 µL of an aqueous solution of (**a2**)$_2$⊂**C** was added to a cuvette containing the same amount of (**b1**)$_2$⊂**C** (in terms of **C**) dissolved in 800 µL of water. The cuvette was quickly inverted twice and placed inside a UV-vis absorption spectrometer, where the initial spectrum (after ~6 s) was recorded. Consecutive spectra were recorded in an automated fashion, one every six seconds. For the **a2**/**b1** combination, we found that the system was equilibrated by the time the first spectrum after mixing was recorded (Figure S94A).

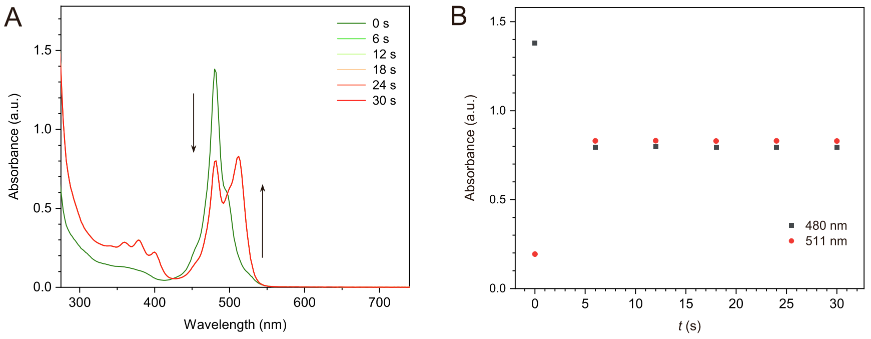


**Figure S94.** (A) UV-vis absorption spectra recorded at various times after mixing (**a2**)$_2$⊂**C** with (**b1**)$_2$⊂**C** (the spectrum at t = 6 s and the subsequent spectra at 12 s, 18 s, 24 s, and 30 s overlap). (B) Absorbance at 480 nm (due to (**b1**)$_2$⊂**C**) and 511 nm (due to (**a2**·**b1**)⊂**C**) followed by time; replotted from (A).

For the (**a4**)$_2$⊂**C** + (**b3**)$_2$⊂**C** mixture, the spectra took significantly longer to stabilize (Figure 3C in the main text). The characteristic peak of homodimer (**b3**)$_2$⊂**C** at 500 nm gradually decreased, while the 534 nm peak due to heterodimer (**a4·b3**)⊂**C** increased over >2 min (Figure S95).

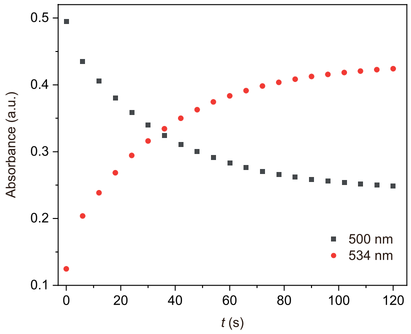


**Figure S95.** Kinetics of equilibration of (**b3**)$_2$⊂**C** + (**a4**)$_2$⊂**C**; absorbance at 500 nm (due to (**b3**)$_2$⊂**C**) and 534 nm (due to (**a4·b3**)⊂**C**) followed by time.

To determine if the slower formation of (**a4·b3**)⊂**C** (compared with (**a2·b1**)⊂**C**) was caused by replacing **a2** with **a4** or **b1** with **b3**, we replaced each guest individually and thus studied two additional **a**/**b** pairs, namely, (**a4**)$_2$⊂**C** + (**b1**)$_2$⊂**C** and (**a2**)$_2$⊂**C** + (**b3**)$_2$⊂**C**. As Figure S96A shows, equilibration within the former mixture was completed within the initial 6 s, indicating that replacing **a2** with **a4** did not affect equilibration (within the initial 6 s). However, heterodimer (**a2·b3**)⊂**C** took significantly longer (~2 min) to form; these observations allow us to conclude that increasing the bulkiness on the BODIPY guest by installing two additional methyl groups has a significant effect on guest exchange kinetics. Similar to the case of encapsulated pyrene (Figure 4C in the main text), the anthracene absorption pattern in Figure S96B was the same after 6 s and after 120 s, despite a small fraction of heterodimer within 6 s of reaction time; this observation indicates that the absorption of anthracene **a2** within the (**a2**)$_2$⊂**C** homodimer and the (**a2·b3**)⊂**C** heterodimer is practically the same. We also compared the formation kinetics of (**a2·b3**)⊂**C** (Figure S97) vs. (**a4·b3**)⊂**C** and found that the former formed slightly faster.

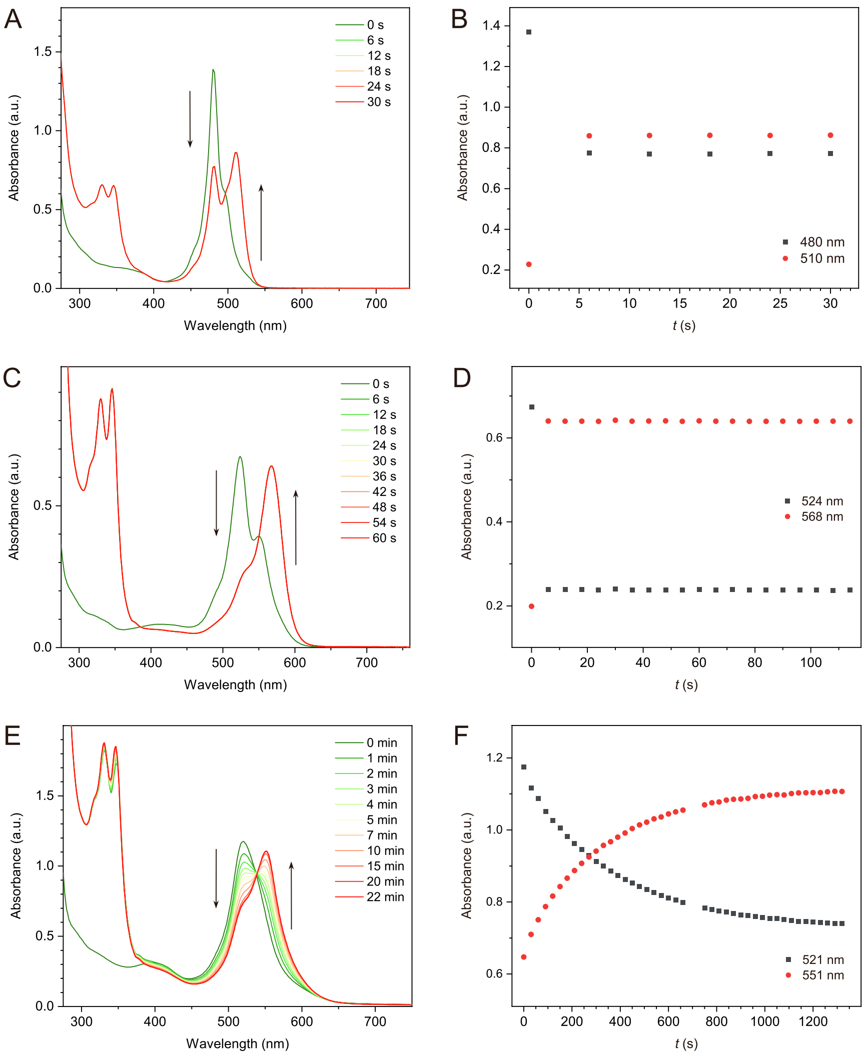


**Figure S96.** (A) Evolution of UV-vis absorption spectra after addition of (**a4**)$_2$⊂**C** to (**b1**)$_2$⊂**C** (the spectrum at t = 6 s and the subsequent spectra overlap). (B) Absorbance at 480 nm (due to (**b1**)$_2$⊂**C**) and 510 nm (due to (**a4**·**b1**)⊂**C**) followed by time; replotted from (A). (C) Evolution of UV-vis spectra after addition of (**a4**)$_2$⊂**C** to (**b2**)$_2$⊂**C**. (D) Absorbance at 524 nm (due to (**b2**)$_2$⊂**C**) and 568 nm (due to (**a4**·**b2**)⊂**C**) followed by time; replotted from (C). (E) Evolution of UV-vis spectra after addition of (**a4**)$_2$⊂**C** to (**b4**)$_2$⊂**C**. (F) Absorbance at 521 nm (due to (**b4**)$_2$⊂**C**) and 551 nm (due to (**a4**·**b4**)⊂**C**) followed by time; replotted from (E).

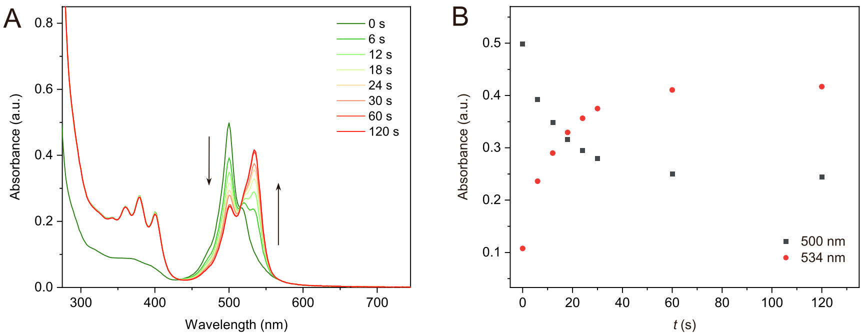


**Figure S97.** (A) Evolution of UV-vis absorption spectra after addition of (**a2**)$_2$⊂**C** to (**b3**)$_2$⊂**C**. (B) Absorbance at 500 nm (due to (**b3**)$_2$⊂**C**) and 534 nm (due to (**a2**·**b3**)⊂**C**) followed by time; replotted from (A).

**Derivation of the kinetic rate equation**

For the reaction,

$$(\mathbf{a})_2{\subset}\mathbf{C} + (\mathbf{b})_2{\subset}\mathbf{C} \underset{k_{-1}}{\overset{k_1}{\rightleftharpoons}} (\mathbf{a}{\cdot}\mathbf{b}){\subset}\mathbf{C} + (\mathbf{a}{\cdot}\mathbf{b}){\subset}\mathbf{C} \qquad (1)$$

let us define the concentrations of species $(\mathbf{a})_2{\subset}\mathbf{C}$, $(\mathbf{b})_2{\subset}\mathbf{C}$, and $(\mathbf{a}{\cdot}\mathbf{b}){\subset}\mathbf{C}$ as [homo], [homo'], and [hetero], respectively.

The above reaction equation represents the equilibrium between the two homodimers $(\mathbf{a})_2{\subset}\mathbf{C}$ and $(\mathbf{b})_2{\subset}\mathbf{C}$ forming two molecules of the heterodimer $(\mathbf{a}{\cdot}\mathbf{b})_2{\subset}\mathbf{C}$ as a result of guest exchange. With [hetero] = 2([homo]$_0$ – [homo]) (where [homo]$_0$ is [homo] at $t = 0$), the decay of $(\mathbf{a})_2{\subset}\mathbf{C}$ upon the addition of 1 eq of $(\mathbf{b})_2{\subset}\mathbf{C}$ can be written as:

$$\frac{d[\text{homo}]}{dt} = -k_1[\text{homo}]^2 + k_{-1}(2([\text{homo}]_0 - [\text{homo}]))^2 =$$

$$= -k_1[\text{homo}]^2 + 4k_{-1}[\text{homo}]_0^2 - 8k_{-1}[\text{homo}]_0[\text{homo}] + 4k_{-1}[\text{homo}]^2$$

Therefore, $$\frac{d[\text{homo}]}{dt} = [\text{homo}]^2(4k_{-1} - k_1) - 8k_{-1}[\text{homo}]_0[\text{homo}] + 4k_{-1}[\text{homo}]_0^2. \qquad (2)$$

Assuming a statistical distribution of guests among cages, the ratio of the equilibrium concentrations,

[homo]:[homo']:[hetero] = 1:1:2.

Let us define $K_{\text{eq}} = \frac{k_{-1}}{k_1}$; at the same time, $K_{\text{eq}} = \frac{[\text{hetero}]^2}{[\text{homo}][\text{homo'}]}$; therefore, $k_1 = 4k_{-1}$ (the factor of 4 originates from considerations of collision probabilities and probabilities of productive guest exchanges. The number of collisions between $(\mathbf{a})_2{\subset}\mathbf{C}$ and $(\mathbf{b})_2{\subset}\mathbf{C}$ (the left-hand side of Equation 1) is twice the number of collisions between $(\mathbf{a}{\cdot}\mathbf{b}){\subset}\mathbf{C}$ (the right-hand side of Equation 1), resulting in a factor of 2. Furthermore, all productive collisions between $(\mathbf{a})_2{\subset}\mathbf{C}$ and $(\mathbf{b})_2{\subset}\mathbf{C}$ result in $(\mathbf{a}{\cdot}\mathbf{b}){\subset}\mathbf{C}$, but only half of all the productive collisions between two copies of $(\mathbf{a}{\cdot}\mathbf{b}){\subset}\mathbf{C}$ result in one of the two homodimers, leading to the second factor of 2). Therefore, Equation 2 then gives:

$$\frac{d[\text{homo}]}{dt} = -8k_{-1}[\text{homo}]_0[\text{homo}] + 4k_{-1}[\text{homo}]_0^2$$

$$dt = \frac{d[\text{homo}]}{-8k_{-1}[\text{homo}][\text{homo}]_0 + 4k_{-1}[\text{homo}]_0^2} = \frac{1}{4k_{-1}[\text{homo}]_0} \cdot \frac{d[\text{homo}]}{[\text{homo}]_0 - 2[\text{homo}]}$$

$$-dt = \frac{1}{4k_{-1}[\text{homo}]_0} \cdot \frac{d[\text{homo}]}{2[\text{homo}] - [\text{homo}]_0}.$$

Integrating both sides of the equation,

$$-\int 1dt = \frac{1}{4k_{-1}[\text{homo}]_0} \cdot \int \frac{1}{2[\text{homo}] - [\text{homo}]_0} d[\text{homo}] + c$$

gives:

$$\frac{1}{8k_{-1}[\text{homo}]_0} \cdot \ln(2[\text{homo}] - [\text{homo}]_0) = -t + c$$

and

$$2[\text{homo}]-[\text{homo}]_0 = c'\cdot e^{-8k_{-1}[\text{homo}]_0 t}.$$

Therefore, with the initial conditions $t = 0$ and [homo] = $[\text{homo}]_0$,

$$[\text{homo}] = \frac{1}{2}[\text{homo}]_0 \cdot (1+e^{-8k_{-1}[\text{homo}]_0 t}).$$

As explained above,

$$[\text{hetero}] = 2\cdot([\text{homo}]_0 - [\text{homo}]);$$

therefore, [hetero] can be written as:

$$[\text{hetero}] = 2\cdot([\text{homo}]_0 - \frac{1}{2}[\text{homo}]_0 \cdot (1+e^{-8k_{-1}[\text{homo}]_0 t})).$$

Finally,

$$[\text{hetero}] = [\text{homo}]_0 \cdot (1-e^{-8k_{-1}[\text{homo}]_0 t}),$$

which can be written as,

$$y = \frac{[\text{hetero}]}{[\text{homo}]_0} = 1-e^{-kt}.$$

## 12. Time-resolved fluorescence spectroscopy of heterodimeric inclusion complexes

The samples were excited by a frequency-tripled Nd:YAG Q-switched laser, pumping an optical parametric oscillator (Ekspla NT342/C/3/UVE) with a pulse duration of 5 ns and a repetition rate of 10 Hz. The fluorescence spectra were collected in the direction orthogonal to the laser incident beam using a 20×0.4 NA objective and spectrally filtered using a longpass filter onto a monochromator (Acton SpectraPro2150i), coupled to a photomultiplier (PMT) tube (Hamamatsu R10699). Transient emission measurements were recorded using a 600 MHz digital oscilloscope (LeCroy Wavesurfer 62Xs). Emission spectra were plotted by integrating over transient emission curve at each wavelength. The excitation pulse energy was measured by a pyroelectric sensor (PE9-C, Ophir Optronics). The spectra of homodimers $(\mathbf{b1})_2$⊂**C**, $(\mathbf{b2})_2$⊂**C**, and $(\mathbf{b4})_2$⊂**C** were reported before.[2]

For sample preparation, $(\mathbf{b1})_2$⊂**C** was mixed with 40 equivalents of $(\mathbf{a1})_2$⊂**C**, $(\mathbf{a2})_2$⊂**C**, or $(\mathbf{a4})_2$⊂**C** in order to maximize the fraction of **b1** within the heterodimer. We verified that the relatively high concentration of the anthracene guests and the cage did not interfere with the measurements (note that they are transparent in the visible area of the spectrum).

The decay for excited (**a1**·**b1**)⊂**C** and (**a4**·**b1**)⊂**C** is shown in the time-resolved emission spectra in Figure S98; additional results (including decay constants) for these two heterodimers as well as for (**a2**·**b1**)⊂**C** can be found in the main text.

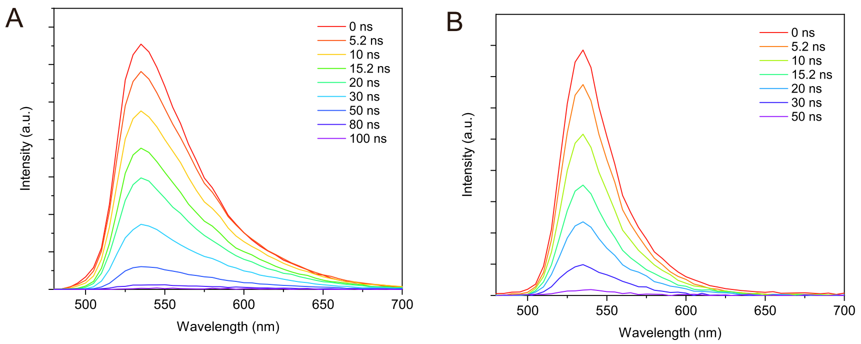


**Figure S98.** (A) Time-resolved fluorescence spectra of (**a1**·**b1**)⊂**C** in $H_2O$. (B) Time-resolved fluorescence spectra of (**a4**·**b1**)⊂**C** in $H_2O$.

## 13. Photodimerization of encapsulated **a1**–**a4**

For photoirradiation experiments, we used a Prizmatix Mic-LED 365 nm light-emitting diode (LED), a 4-W hand-held 365 nm UV lamp (UVP, LLC; model number UVGL-25), or a pE-4000 LED illumination system (CoolLED; wavelength 385 nm) as the UV light sources. The samples were irradiated in quartz cuvettes at a concentration of 0.031 mM (in terms of cage units) and a fixed distance of 20 cm.

First, the complex $(\mathbf{a2})_2$⊂**C** in aqueous solution was irradiated, and the progress of the reaction was monitored by UV-vis absorption spectroscopy by following the decrease of the characteristic absorbance pattern in the near-UV region (340–420 nm). The reaction was complete within five minutes (see Figure 4B, C in the main text). In contrast, free **a2** dissolved in DCM at the same concentration reacted much slower under the same irradiation conditions; the reaction was not complete after 30 minutes (Figure S99).

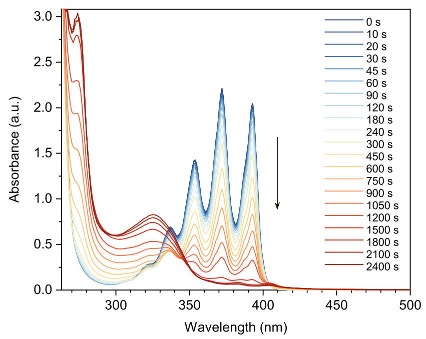


**Figure S99.** Photodimerization of **a2** in DCM with 365 nm UV light. For $(\mathbf{a2})_2$⊂**C** in an aqueous solution, see Figure 4B, C in the main text.

From this experiment, we concluded that the prearrangement of anthracene units within the cavity of **C** allowed for very fast conversion into the covalent dimer, which we denote **a2a2**. To confirm the identity of the product, we followed the same reaction using $^1$H NMR, working with a 3 mM solution of $(\mathbf{a2})_2$⊂**C** in $D_2O$ (Figure S100; in this experiment, we used a 385 nm light source, which allowed for a faster reaction under the increased concentration).

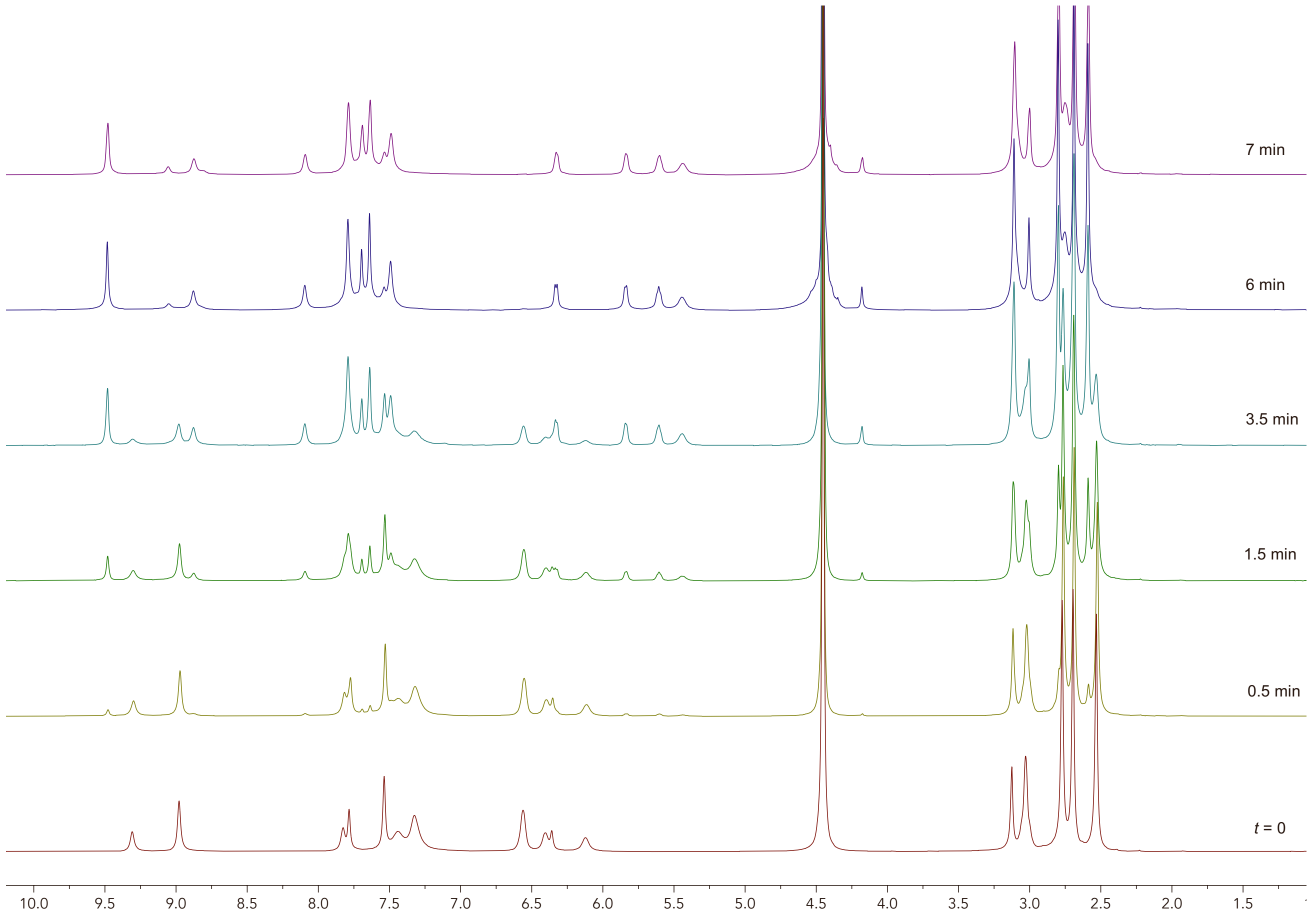


**Figure S100.** Changes in the $^{1}H$ NMR spectra of (**a2**)$_2$⊂**C** during the irradiation with 385 nm UV light, indicating transformation into encapsulated dianthracene (**a2a2**)⊂**C** (500 MHz, $D_2O$, 330 K).

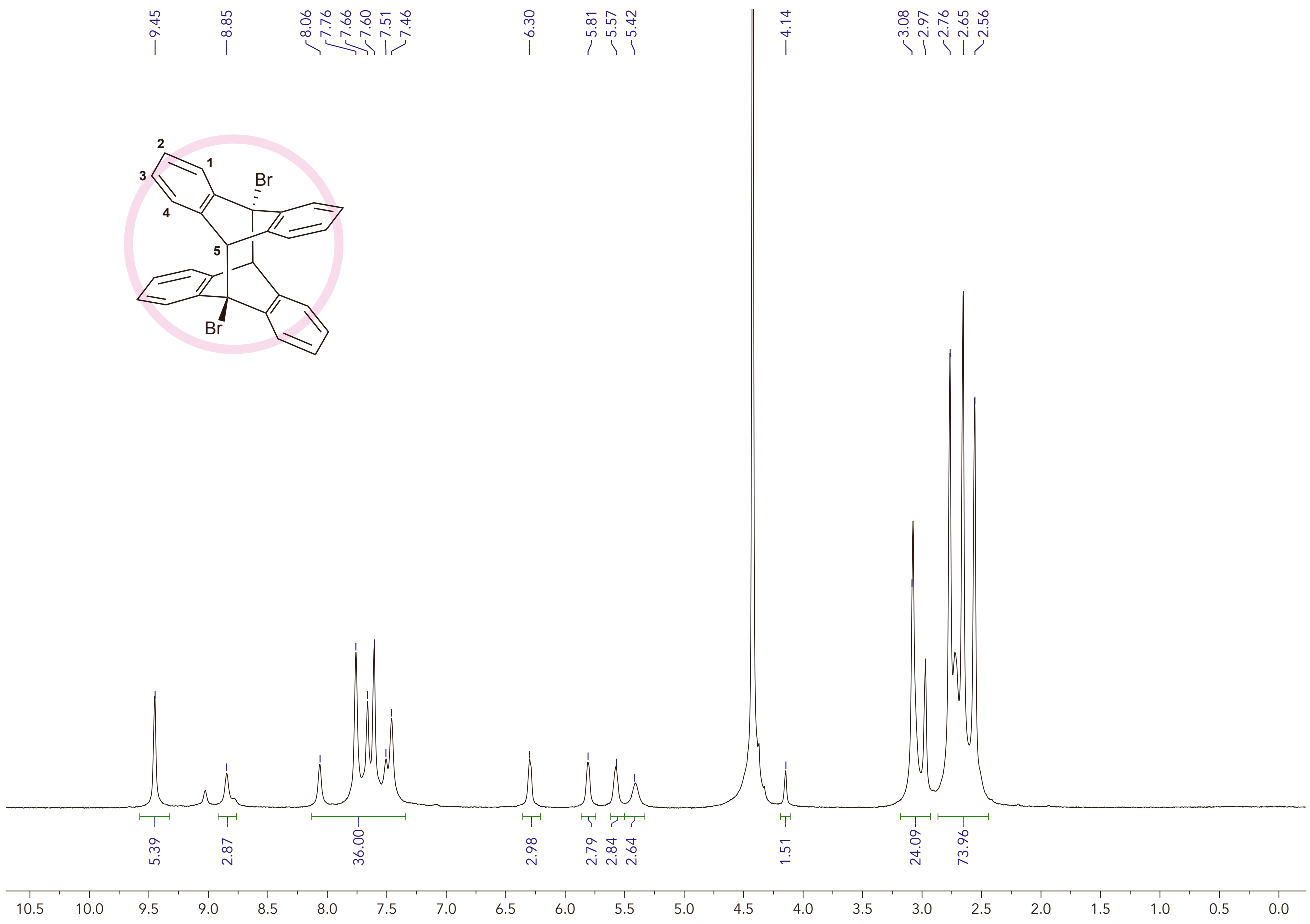


**Figure S101.** $^1$H NMR spectrum of the metastable inclusion complex (**a2a2**)⊂**C** (500 MHz, $D_2O$, 330 K).

Upon irradiation, we observed new peaks that were upfield-shifted compared to encapsulated **a2** ($\delta$ = 6.33, 5.83, 5.61, 5.45, and 4.17 ppm; Figures S100, S101). The presence of a single peak at ~4.14 ppm indicates that only one isomer of dianthracene **a2a2** (head-to-tail vs. head-to-head) was formed. The peaks due to encapsulated **a2** ($\delta$ = 6.08, 6.32, 6.36, and 6.52 ppm) disappeared during the experiment, indicating a near-quantitative reaction. The dimerization reaction was accompanied by pronounced changes in the chemical shifts of the cage's peaks, suggesting that the cage needs to undergo a large conformational change to accommodate **a2a2**. Despite this change, **C** was unable to complex the dianthracene, as evidenced by gradual precipitation of a colorless solid, which we identified by NMR spectroscopy as **a2a2** (no precipitation was observed in the micromolar concentration range typical of UV-vis absorption measurements).

Based on a COSY experiment (data not shown), the peaks of **a2a2** within **C** could be assigned as follows:

$^1$H NMR (500 MHz, $D_2O$, 330 K): $\delta$ = 6.30 (4H, d, **a2a2$_1$**), 5.81 (4H, d, **a2a2$_4$**), 5.57 (4H, t, **a2a2$_2$**), 5.42 (4H, t, **a2a2$_3$**), 4.14 (2H, s, **a2a2$_5$**).

After 7 min of irradiation, $CDCl_3$ was added to the NMR tube to extract the photoreaction product. NMR analysis of the organic phase revealed the presence of the head-to-tail (*ht*) isomer of **a2a2** (see Figure S102, which compares the obtained spectrum to that of *ht*-**a2a2**[18]). The identity of *ht*-**a2a2** was further confirmed by 2D NMR experiments (data not shown).

Exposing a solution of **a2** in $CDCl_3$ to 365 nm UV light resulted in a mixture of **a2a2** and unreacted **a2**, even at much longer irradiation times (2.5 h in Figure S102C). Like for encapsulated **a2**, a single upfield-shifted (5.36 ppm) peak emerges, indicating the formation of only one isomer of dianthracene (*ht*, as reported before[18]). In addition, the formation of side products was evident, in contrast to the confined system.

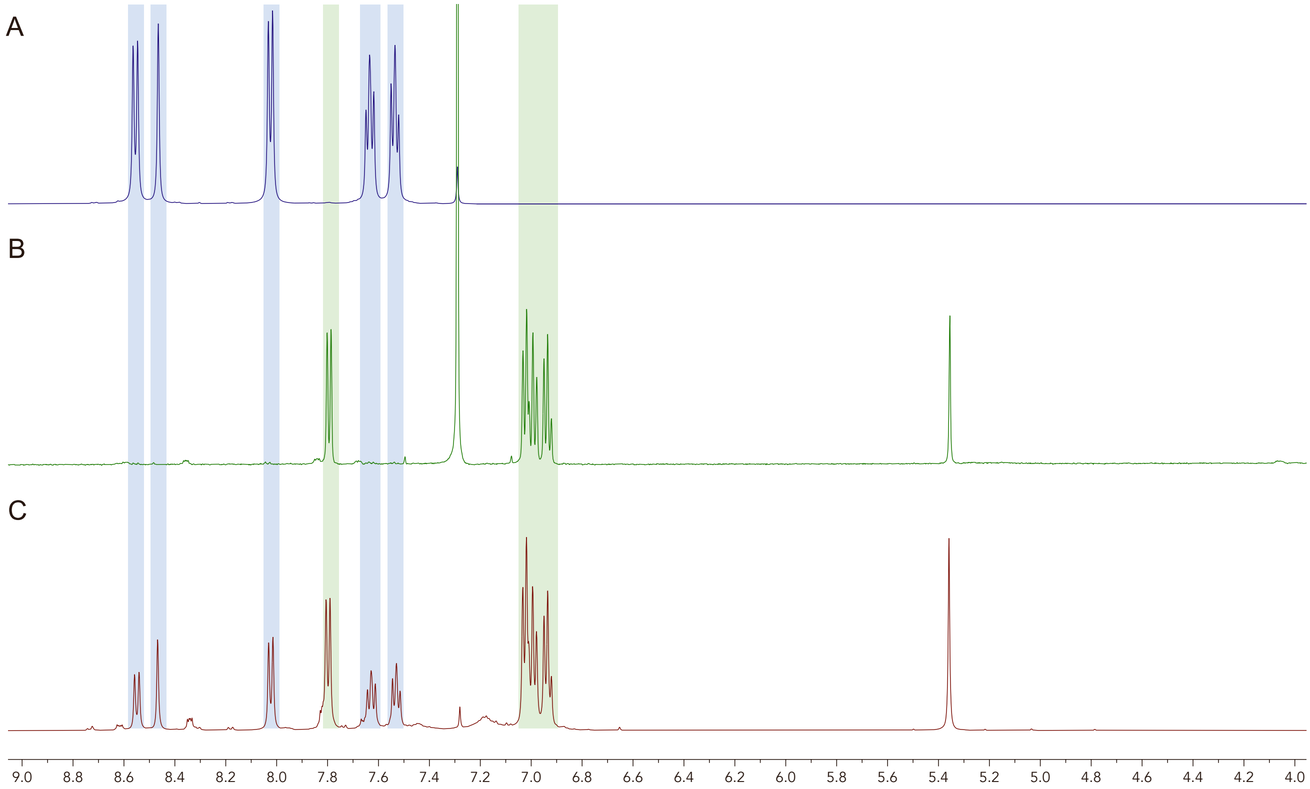


**Figure S102.** (A) $^1$H NMR spectrum of **a2** in $CDCl_3$ before irradiation. (B) $^1$H NMR spectrum of the organic phase ($CDCl_3$) after irradiating an aqueous solution of $(\mathbf{a2})_2\subset\mathbf{C}$ with 385 nm UV light for 7 min followed by extraction. (C) $^1$H NMR spectrum of **a2** in $CDCl_3$ after irradiation for 160 min with UV light. The spectrum shows the presence of **a2** (blue shades) and **a2a2** (green shades), in addition to side products.

Next, we irradiated a solution of $(\mathbf{a2a2})\subset\mathbf{C}$ (at a concentration in the range of μM) with shorter wavelengths of UV light (254 nm; we used a 4-W hand-held UV lamp; UVP, LLC; model number UVGL-25). The [4+4] photodimerization could partially be reversed, as evidenced by the reappearance of the UV absorption pattern characteristic of anthracene derivatives. However, the initial absorbance of **a2** was not reached even after long irradiation times, indicating the limited reversibility of this reaction. The broadening of the peaks in the UV-vis absorption spectra was observed, suggesting the occurrence of side reactions—such as photolysis of the C–Br bond[18]—which can explain the limited reversibility.

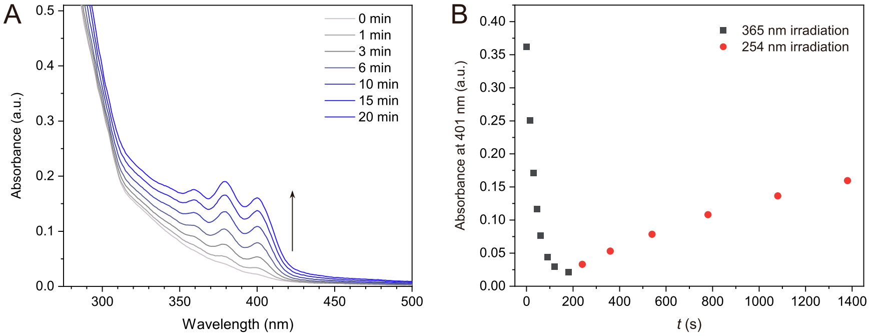


**Figure S103.** (A) UV-vis absorption spectra accompanying irradiation of an aqueous solution of (**a2a2**)⊂**C** at 254 nm (the initial solution was obtained by exposing (**a2**)$_2$⊂**C** to 365 nm light for 3 min). (B) Following the reversible photodimerization of **a2** within **C** upon irradiation with UV light at 365 nm, followed by 254 nm.

Next, we studied the photoresponsiveness of (**a1**)$_2$⊂**C** by exposing its aqueous solution to 365 nm UV light, analogously to (**a2**)$_2$⊂**C** (see above). However, hardly any changes in the 340–420 nm absorption pattern occurred (Figure S104A), indicating no dimerization of **a1** within **C** (in contrast to (**a2**)$_2$⊂**C**). This result is particularly surprising given that **a1** dissolved in DCM photodimerized much faster than **a2** under the same conditions (see Figure 4C and E in the main text). Anthracene **a3** within **C** photodimerized efficiently (Figure 4G), albeit slower than **a2**.

We also subjected an aqueous solution of (**a4**)$_2$⊂**C** to photoirradiation; although free **a4** does not undergo a photoreaction under 365 nm light, various unexpected [2+2] and [2+4] photodimerization reactions were previously reported in confined systems.[19] However, no indication of a reaction was found (Figure S104B).

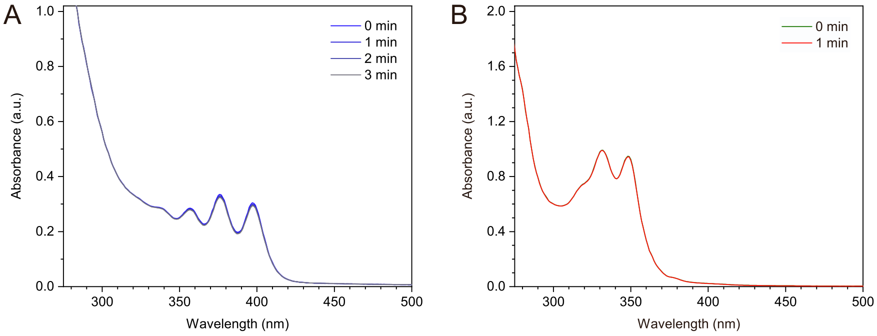


**Figure S104.** (A) UV-vis absorption spectra accompanying photoirradiation of an aqueous solution of (**a1**)$_2$⊂**C** with 365 nm UV light. (B) UV-vis absorption spectra accompanying photoirradiation of an aqueous solution of (**a4**)$_2$⊂**C** with 365 nm UV light.

## 14. Photoresponsiveness of heterodimeric inclusion complexes

As shown in Section 13, exposing homodimeric complexes (**a2**)$_2$⊂**C** and (**a3**)$_2$⊂**C** to 365 nm light converts them rapidly to the corresponding dianthracenes (denoted as (**a2a2**)⊂**C** and (**a3a3**)⊂**C**, respectively) as a result of a [4+4] photoreaction. Given the fast guest exchange between inclusion complexes of cage **C**, we hypothesized that the [4+4] reaction could also proceed rapidly in a dynamic mixture of several different complexes. To this end, we mixed aqueous solutions of (**a2**)$_2$⊂**C** (4 eq) and (**b1**)$_2$⊂**C** (1 eq) (equivalents in terms of the cage) to afford a mixture of (**a2**)$_2$⊂**C**, (**b1**)$_2$⊂**C**, and (**a2**·**b1**)⊂**C**. Exposing this mixture to 365 nm light led to a decrease of the characteristic anthracene absorbance pattern in the near UV area (340–420 nm; see Figure 5B in the main text), confirming dianthracene formation. Notably, the photoreaction proceeded somewhat slower than for pure (**a2**)$_2$⊂**C**, which can be explained by the presence of other species—(**b1**)$_2$⊂**C** and (**a2**·**b1**)⊂**C**—absorbing in the near-UV region and lowering the overall quantum efficiency of the [4+4] reaction.

In addition, exposure to UV light induced large changes in the visible part of the spectrum (where the reactive species, **a2**, does not absorb). In particular, the intensity of the heterodimer's band at 511 nm decreased at the expense of the 480 nm band, indicative of the formation of homodimer (**b1**)$_2$⊂**C** (Figure S105A). After 8 min of irradiation, the absorption spectrum was almost indistinguishable from that of (**b1**)$_2$⊂**C** (Figure S105B), indicating that the equilibrium between the homodimers and the heterodimer was strongly shifted to favor (**b1**)$_2$⊂**C** and disfavor (**a2**·**b1**)⊂**C** (Figure 5B).

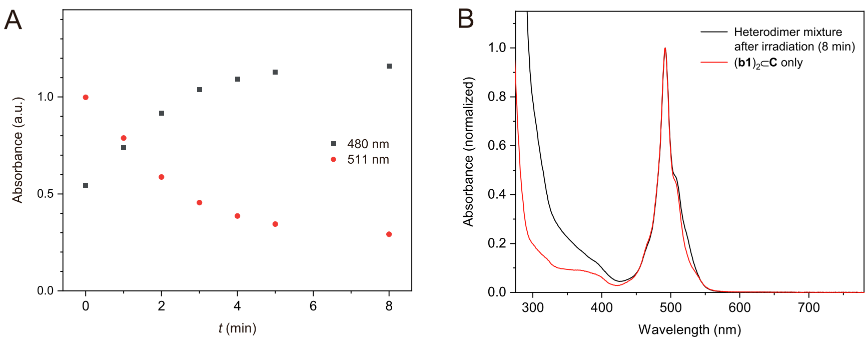


**Figure S105.** (A) Changes in absorbance at 480 nm (due to (**b1**)$_2$⊂**C**) and 511 nm (due to (**a2**·**b1**)⊂**C**) during irradiation with 365 nm UV light over time; for the spectra, see Figure 5B in the main text. (B) Comparison of the UV-vis absorption spectra of aqueous (**b1**)$_2$⊂**C** (red) and a mixture of (**a2**)$_2$⊂**C**, (**b1**)$_2$⊂**C**, and (**b1**)$_2$⊂**C** after irradiation for 8 min (black).

The reaction was also followed by fluorescence spectroscopy, which showed a substantial decrease in emission during the experiment (Figure S106A). Fluorescence quenching could also be appreciated by the naked eye (Figure S106B).

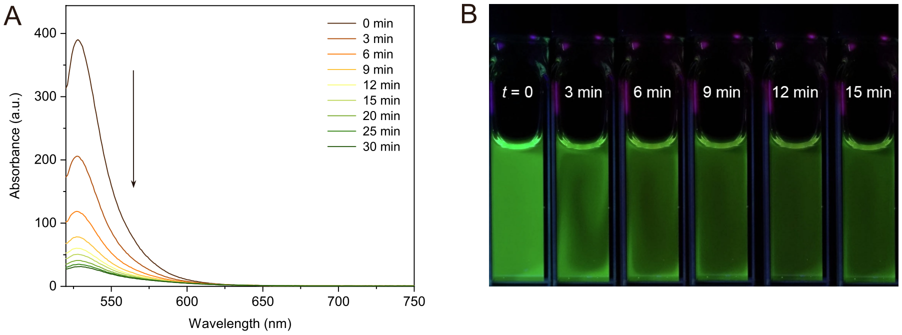


**Figure S106.** (A) Changes in the fluorescence spectra of a mixture of (**a2**)$_2$⊂**C**, (**b1**)$_2$⊂**C**, and (**b1**)$_2$⊂**C** upon irradiation with 365 nm UV light. (B) Photographs of a mixture of (**a2**)$_2$⊂**C**, (**b1**)$_2$⊂**C**, and (**b1**)$_2$⊂**C** taken at various times during UV irradiation (note that a UV light source different than in the previous experiment (Figure S105) was applied). All the photographs were under the same excitation conditions with $\lambda_{exc}$ = 365 nm.

Replacing (**a2**)$_2$⊂**C** with either (**a1**)$_2$⊂**C** or (**a4**)$_2$⊂**C** rendered the system non-photoresponsive (as expected from the lack of photoresponsiveness of both (**a1**)$_2$⊂**C** and (**a4**)$_2$⊂**C**) – see Figure S107A and B, respectively. If, however, (**b1**)$_2$⊂**C** was replaced by any other BODIPY homodimer—(**b2**)$_2$⊂**C**, (**b3**)$_2$⊂**C**, or (**b4**)$_2$⊂**C**—the system retained its photoresponsiveness (Figure S107C and D).

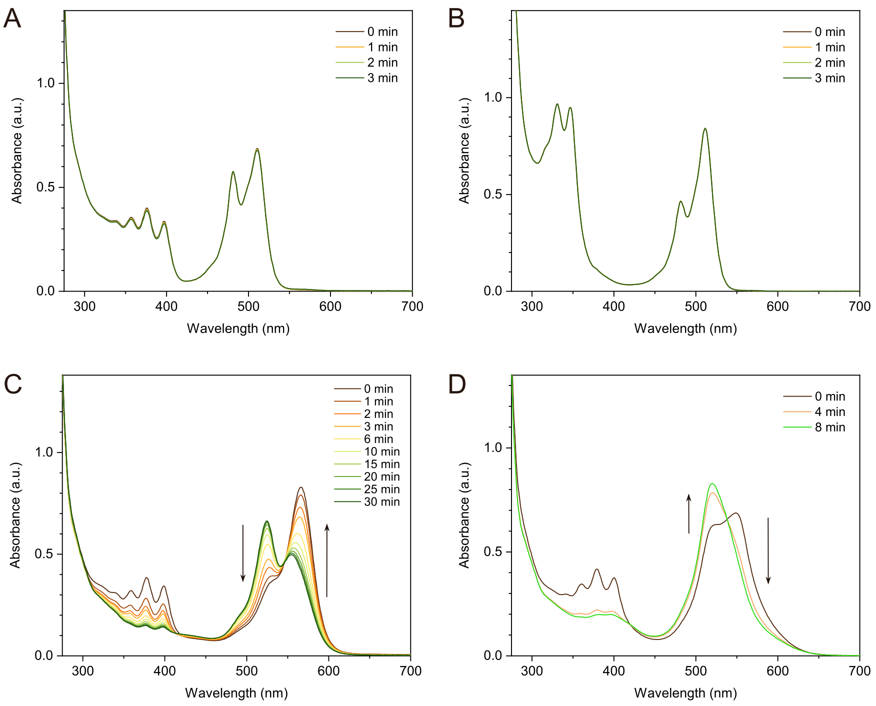


**Figure S107.** (A) No changes in the UV-vis absorption spectra were observed upon exposing an (**a1**·**b1**)⊂**C** + (**a1**)$_2$⊂**C** + (**b1**)$_2$⊂**C** mixture to 365 nm light. (B) Similarly, no changes were observed when an (**a4**·**b1**)⊂**C** + (**a4**)$_2$⊂**C** and (**b1**)$_2$⊂**C** mixture was exposed to 365 nm light. (C) Changes in the UV-vis spectra of an (**a2**·**b2**)⊂**C** + (**a2**)$_2$⊂**C** and (**b2**)$_2$⊂**C** mixture exposed to 365 nm light; a fast reaction was observed. (D) Changes in the UV-vis spectra of an (**a2**·**b4**)⊂**C** + (**a2**)$_2$⊂**C** and (**b4**)$_2$⊂**C** mixture exposed to 365 nm light; a fast reaction was observed.

Exposing dianthracenes to 254 nm UV light induces the dedimerization reaction to afford the starting anthracene. Irradiating the (**a2a2**)$\subset$**C** + $(\mathbf{b1})_2\subset\mathbf{C}$ mixture at 254 nm induced the reappearance of the characteristic anthracene pattern in the near-UV region (Figure S108A). At the same time, the ratio of homodimer $(\mathbf{b1})_2\subset\mathbf{C}$ to heterodimer (**a2·b1**)$\subset$**C** changed in favor of the latter (Figure S108A). However, due to the limited reversibility of cycloreversion (Section 13), the system did not reach its initial state fully (see Figure S108B).

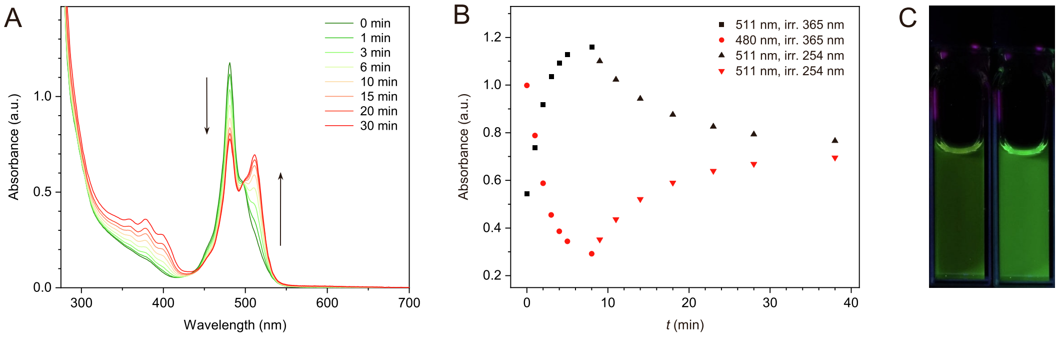


**Figure S108.** (A) Changes in the UV-vis absorption spectra of an (**a2·b1**)$\subset$**C** + $(\mathbf{a2})_2\subset\mathbf{C}$ + $(\mathbf{b1})_2\subset\mathbf{C}$ mixture that had been irradiated with 365 nm light (8 min) during irradiation with 254 nm light. (B) Reversible changes in the absorbance at 480 nm (due to $(\mathbf{b1})_2\subset\mathbf{C}$) and 511 nm (due to (**a2·b1**)$\subset$**C**) during irradiation with 365 nm and then 254 nm light. (C) Photographs of a (**a2·b1**)$\subset$**C** + $(\mathbf{a2})_2\subset\mathbf{C}$ + $(\mathbf{b1})_2\subset\mathbf{C}$ mixture that had been irradiated with 365 nm light for 15 min before (left) and after (right) exposure to 254 nm light for 3 min. The photographs were under the same excitation conditions with $\lambda_{exc}$ = 365 nm.

## 15. Preparation of photoresponsive gels based on heterodimeric inclusion complexes

Agarose (1 g) was added to an Erlenmeyer flask containing 50 mL of distilled water. The mixture was heated in an oil bath; once water started to boil, heating was continued for an additional 3 min. Then, the flask was removed from the oil bath, and the resulting colorless, homogeneous solution was poured (while hot) between two glass slides separated by 1 mm spacers. After having been cooled to room temperature, the solidified agarose gel was removed from the template and cut into rectangular pieces (25×35×1 mm or 10×10×1 mm) using a cutter knife. The gels were transferred to a Petri dish or a vial containing aqueous solutions of inclusion complexes for soaking (60 min).

## II. Supplemental references

1. Mizuno, K., Tamiya, Y., and Mekata, M. (2004). External double reference method to study concentration and temperature dependences of chemical shifts determined on a unified scale. Pure Appl. Chem. *76*, 105–114.
2. Gemen, J., Ahrens, J., Shimon, L. J. W., and Klajn, R. (2020). Modulating the optical properties of BODIPY dyes by noncovalent dimerization within a flexible coordination cage. J. Am. Chem. Soc. *142*, 17721–17729.
3. Nepomnyashchii, A. B., Bröring, M., Ahrens, J., and Bard, A. J. (2011). Synthesis, photophysical, electrochemical, and electrogenerated chemiluminescence studies. Multiple sequential electron transfers in BODIPY monomers, dimers, trimers, and polymer. J. Am. Chem. Soc. *133*, 8633–8645.

4. Dolomanov, O. V., Bourhis, L. J., Gildea, R. J., Howard, J. A. K., and Puschmann, H. (2009). *OLEX2*: a complete structure solution, refinement and analysis program. J. Appl. Crystallogr. *42*, 339–341.
5. Sheldrick, G. M. (2008). A short history of *SHELX*. Acta Crystallogr. Sect. A *64*, 112–122.
6. Spek, A. L. (2015). *PLATON* SQUEEZE: a tool for the calculation of the disordered solvent contribution to the calculated structure factors. Acta Crystallogr. Sect. C *71*, 9–18.
7. Samanta, D., Galaktionova, D., Gemen, J., Shimon, L. J. W., Diskin-Posner, Y., Avram, L., Král, P., and Klajn, R. (2018). Reversible chromism of spiropyran in the cavity of a flexible coordination cage. Nat. Commun. *9*, 641.
8. Turowska-Tyrk, I., and Trzop, E. (2003). Monitoring structural transformations in crystals. 6. The [4 + 4] photodimerization of 9-methyl-anthracene. Acta Crystallogr. B *59*, 779–786.
9. Mondal, R., Nesterov, E. E., and Fronczek, F. R. (2012). CCDC 864332: Experimental crystal structure determination. DOI: 10.5517/ccy0dp0.
10. Sweeting, L. M., and Rheingold, A. L. (1988). Crystal structure and triboluminescence. 1. 9-Anthryl carbinols. J. Phys. Chem. *92*, 5648–5655.
11. Camerman, A., and Trotter, J. (1965). The crystal and molecular structure of pyrene. Acta. Cryst. *18*, 636–643.
12. Becke, A. D. (1993). Density-functional thermochemistry. III. The role of exact exchange. J. Chem. Phys. *98*, 5648–5652.
13. Lee, C., Yang, W., and Parr, R. G. (1988). Development of the Colle-Salvetti correlation-energy formula into a functional of the electron density. Phys. Rev. B *37*, 785–789.
14. Frisch, M. J., Trucks, G. W., Schlegel, H. B., Scuseria, G. E., Robb, M. A., Cheeseman, J. R., Scalmani, G., Barone, V., Petersson, G. A., Nakatsuji, H., Li, X., Caricato, M., Marenich, A. V., Bloino, J., Janesko, B. G., Gomperts, R., Mennucci, B., Hratchian, H. P., Ortiz, J. V., Izmaylov, A. F., Sonnenberg, J. L., Williams-Young, D., Ding, F., Lipparini, F., Egidi, F., Goings, J., Peng, B., Petrone, A., Henderson, T., Ranasinghe, D., Zakrzewski, V. G., Gao, J., Rega, N., Zheng, G., Liang, W., Hada, M., Ehara, M., Toyota, K., Fukuda, R., Hasegawa, J., Ishida, M., Nakajima, T., Honda, Y., Kitao, O., Nakai, H., Vreven, T., Throssell, K., Montgomery, J. A., Jr., Peralta, J. E., Ogliaro, F., Bearpark, M. J., Heyd, J. J., Brothers, E. N., Kudin, K. N., Staroverov, V. N., Keith, T. A., Kobayashi, R., Normand, J., Raghavachari, K., Rendell, A. P., Burant, J. C., Iyengar, S. S., Tomasi, J., Cossi, M., Millam, J. M., Klene, M., Adamo, C., Cammi, R., Ochterski, J. W., Martin, R. L., Morokuma, K., Farkas, O., Foresman, J. B., and Fox, D. J. Gaussian 16, Revision C.01. Gaussian, Inc., Wallingford CT, 2016.
15. Jones, R. N. (1947). The ultraviolet absorption spectra of anthracene derivatives. Chem. Rev. *41*, 353–371.
16. Choi, S., Bouffard, J., and Kim, Y. (2014). Aggregation-induced emission enhancement of a *meso*-trifluoromethyl BODIPY *via* J-aggregation. Chem. Sci. *5*, 751–755.
17. Yamashina, M., Sartin, M. M., Sei, Y., Akita, M., Takeuchi, S., Tahara, T., and Yoshizawa, M. (2015). Preparation of highly fluorescent host–guest complexes with tunable color upon encapsulation. J. Am. Chem. Soc. *137*, 9266–9269.
18. Fulara, J., and Latowski, T. (1981). Photochemical reactions of bromoanthracenes with N,N-dimethylamine in solution. Z. Naturforsch. B *36*, 846–851.
19. Nishioka, Y., Yamaguchi, T., Yoshizawa, M., and Fujita, M. (2007). Unusual [2+4] and [2+2] cycloadditions of arenes in the confined cavity of self-assembled cages. J. Am. Chem. Soc. *129*, 7000–7001.
