# Supplementary material for: Ternary host-guest complexes with rapid exchange kinetics and photoswitchable fluorescence
Source: Chem. 2022 Sep 8;8(9):2362–79. doi: 10.1016/j.chempr.2022.05.008 (PMC9473544; doi:10.1016/j.chempr.2022.05.008)
Supplement: Document S2. Article plus supplemental information [file mmc2.pdf]

Chem

CellPress
OPEN ACCESS

Article

Ternary host-guest complexes with rapid exchange kinetics and photoswitchable fluorescence

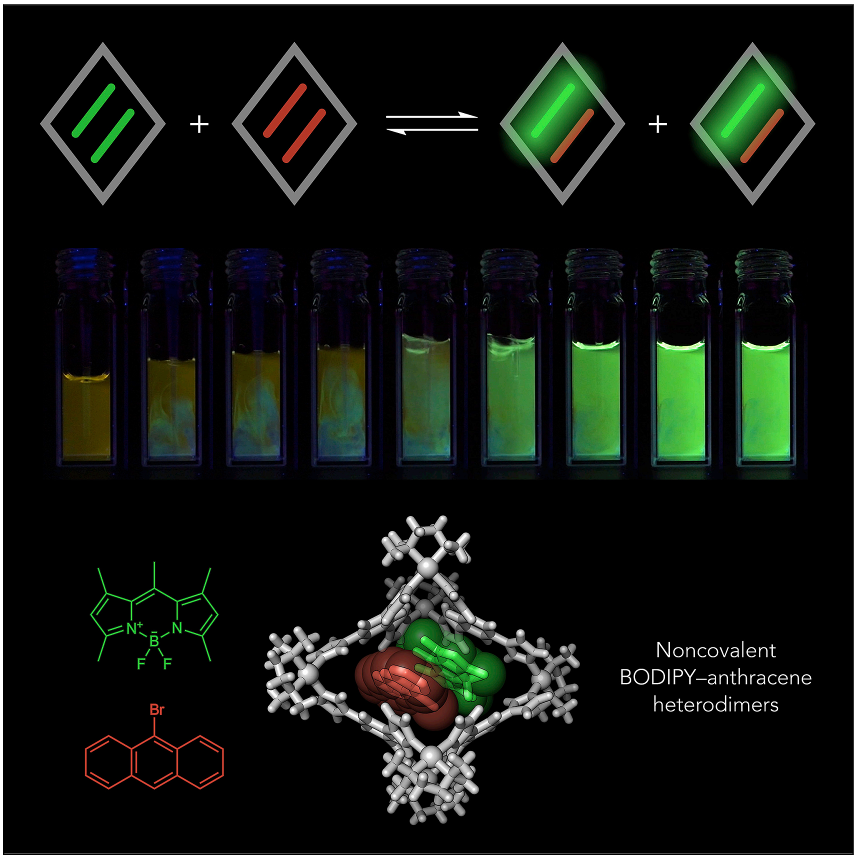

Julius Gemen, Michał J. Białek, Miri Kazes, ..., Yael Diskin-Posner, Dan Oron, Rafal Klajn

rafal.klajn@weizmann.ac.il

Highlights

A coordination cage encapsulates various anthracenes and BODIPY dyes as homodimers

Mixing the two homodimers leads to the formation of anthracene-BODIPY heterodimers

Encapsulation can either promote or suppress the photodimerization of anthracenes

The homodimer/heterodimer equilibrium can be tuned by light-induced guest exchange

The optical properties of dyes are strongly dependent on their immediate environment. For example, the emission of fluorescent dyes can be effectively quenched upon noncovalent dimer formation. Here, we show that combinations of fluorescent BODIPY dyes and photodimerizable anthracenes can bind as heterodimers inside the cavity of a water-soluble coordination cage. Exposure to light triggers anthracene dimerization, which shifts the homodimer-heterodimer equilibrium and consequently controls the optical properties of the system.

9 INDUSTRY, INNOVATION AND INFRASTRUCTURE

Gemen et al., Chem 8, 2362–2379
September 8, 2022 © 2022 The Author(s).
Published by Elsevier Inc.
https://doi.org/10.1016/j.chempr.2022.05.008

CellPress
OPEN ACCESS

Chem

Article

Ternary host-guest complexes with rapid exchange kinetics and photoswitchable fluorescence

Julius Gemen,[1] Michał J. Białek,[1,2] Miri Kazes,[1] Linda J.W. Shimon,[3] Moran Feller,[1] Sergey N. Semenov,[1] Yael Diskin-Posner,[3] Dan Oron,[1] and Rafal Klajn[1,4,*]

SUMMARY

Confinement within molecular cages can dramatically modify the physicochemical properties of the encapsulated guest molecules, but such host-guest complexes have mainly been studied in a static context. Combining confinement effects with fast guest exchange kinetics could pave the way toward stimuli-responsive supramolecular systems—and ultimately materials—whose desired properties could be tailored "on demand" rapidly and reversibly. Here, we demonstrate rapid guest exchange between inclusion complexes of an open-window coordination cage that can simultaneously accommodate two guest molecules. Working with two types of guests, anthracene derivatives and BODIPY dyes, we show that the former can substantially modify the optical properties of the latter upon noncovalent heterodimer formation. We also studied the light-induced covalent dimerization of encapsulated anthracenes and found large effects of confinement on reaction rates. By coupling the photodimerization with the rapid guest exchange, we developed a new way to modulate fluorescence using external irradiation.

THE BIGGER PICTURE

Confinement of small molecules within the cavities of natural and synthetic hosts can greatly affect the physicochemical properties of the bound species; however, to date, such host-guest complexes have been studied mainly in a static context. An important direction is the development of host-guest systems, whereby encapsulation and release of guest molecules can be reversibly controlled using light. Here, we report ternary inclusion complexes comprising an open-window coordination cage and two kinds of photoactive guests, namely, the photodimerizable anthracenes and BODIPY dyes. Alternating exposure to two different colors of light shifts the equilibrium between the encapsulated homodimers and heterodimers, thus dramatically affecting the system's optical properties. We also find that the rates of both processes—anthracene dimerization and guest exchange—strongly depend on the substitution pattern on both types of guests, which highlights the importance of confinement effects.

INTRODUCTION

Confining molecules in spaces not much larger than the molecules themselves can profoundly affect their physical and chemical properties.[1] Diverse types of nanoconfinement have been shown to modulate chemical reactivity of the encapsulated species.[2,3] For example, Rebek and co-workers showed that co-encapsulation of phenylacetylene and phenyl azide within a hydrogen-bonded capsule accelerates the 1,3-dipolar cycloaddition reaction between them by a factor of >200.[4] The same system enabled complete regioselectivity, with only one of two possible triazole isomers formed, in contrast to a 1:1 mixture obtained in a solution of free molecules.[4] Among other examples, confinement between long alkyl chains of thiolate self-assembled monolayers on gold accelerated a silane alcoholysis reaction,[5] and confinement between densely packed nanoparticles induced an unusual regioselectivity in a [4+4] cycloaddition, along with rate acceleration.[6] Conversely, confinement can also decelerate chemical reactions by stabilizing otherwise reactive and/or unstable species. Since Cram's seminal report on "taming"[7] cyclobutadiene within a hemicarcerand host, numerous other species were stabilized within—but made to react upon release from—molecular cages, including white phosphorus,[8] silanol oligomers,[9] the C_{60} radical anion,[10] and radical initiators,[11,12] whose on-demand release can be used to trigger a free-radical polymerization reaction.[12] Similarly, encapsulation within molecular containers was found to increase the

 © 2022 The Author(s). Published by Elsevier Inc.
This is an open access article under the CC BY license (http://creativecommons.org/licenses/by/4.0/).

CellPress
OPEN ACCESS

photochemical stability of fluorescent dyes (such as rhodamine[13]) and to reduce fatigue during the reversible isomerization of the dihydropyrene photoswitch.[14]

Nanoconfinement can also modulate the optical properties of the encapsulated species.[15,16] Both absorption and emission of dyes can be altered by encapsulation within the cavities of metal-organic frameworks,[17] molecular cages,[18] and protein molecules.[19] For example, a water-soluble Pt-based coordination cage stabilizes the monomeric form of tetraazaporphine, thus preventing undesired aggregation and enabling strong fluorescence in aqueous media.[20] Confinement can also promote emission of the bound guest by restricting its conformation.[21] Hosts with larger cavities can simultaneously encapsulate two guest molecules; such noncovalent dimerization was shown to red-shift and suppress the emission of BODIPY dyes.[22,23] In a related study, host-guest interactions were used to assemble coumarin dyes into either H- or J-dimers, depending on the substitution pattern on the coumarin scaffold.[24] Interestingly, confinement of isomerizable dyes can reverse the relative stability of two isomers, as was demonstrated for phenolphthalein[18] and a donor-acceptor Stenhouse adduct,[25] both within Pd-based coordination cages. The properties of confined guests can further be tuned by co-encapsulating them with other guests as ternary complexes of the form (guest•guest′)⊂host.[26–30]

However, relatively little attention has been devoted to tuning the above properties rapidly and reversibly by means of repeated encapsulation and release of the guests. This deficiency is most likely due to the closed structure of many molecular hosts, which necessitates a partial disassembly of the host for guest encapsulation/release to take place.[31–37] The ability to combine fast guest exchange with encapsulation-induced change in physicochemical properties could facilitate the development of new stimuli-responsive supramolecular systems—and ultimately materials—with rapid response times.

To this end, we worked with a flexible coordination cage assembled from six *cis*-blocked Pd^{2+} ions and four triimidazolylbenzene (TImB) panels (**C** in Scheme 1A).[38] Similar to many other cages based on metal-ligand coordination, **C** combines excellent aqueous solubility with the presence of a hydrophobic cavity, which enables it to effectively solubilize various nonpolar molecules in water.[14,23,38] However, **C** offers two additional advantages: first, it contains two large windows, which makes the hydrophobic cavity readily accessible; second, the cage is flexible (owing to the rotation around the C–N bonds connecting the imidazole groups to the panel's central benzene ring[39]) and can adopt a variety of conformations. This structural flexibility has enabled encapsulation of a variety of structurally diverse guests[23,39–42] and efficient photoisomerization reactions of the encapsulated molecules, even when accompanied by large structural changes.[43,44]

As a proof-of-concept, we worked with combinations of (1) aromatic compounds **a1–a4** (Scheme 1B), which we collectively refer to as polycyclic aromatic hydrocarbons (PAHs; native or substituted), and (2) BODIPY dyes **b1–b4** (Scheme 1C). We hypothesized that cage **C** should randomly bind two copies of each of these structurally similar molecules, making the formation of ternary complexes feasible (Scheme 1D). It is well known that, on the one hand, the optical properties of BODIPYs[23,45–48] (and other dyes[49–53]) are strongly dependent on their supramolecular environment; on the other hand, when placed in close proximity, anthracenes can undergo a fast [4+4] cycloaddition reaction,[54–58] making the BODIPY/PAH combination ideally suited to investigate the exchange dynamics in host-guest inclusion complexes.

[1]Department of Molecular Chemistry & Materials Science, Weizmann Institute of Science, Rehovot 76100, Israel

[2]Department of Chemistry, University of Wrocław, 14 F. Joliot-Curie St., 50383 Wrocław, Poland

[3]Department of Chemical Research Support, Weizmann Institute of Science, Rehovot 76100, Israel

[4]Lead contact

*Correspondence: rafal.klajn@weizmann.ac.il

https://doi.org/10.1016/j.chempr.2022.05.008

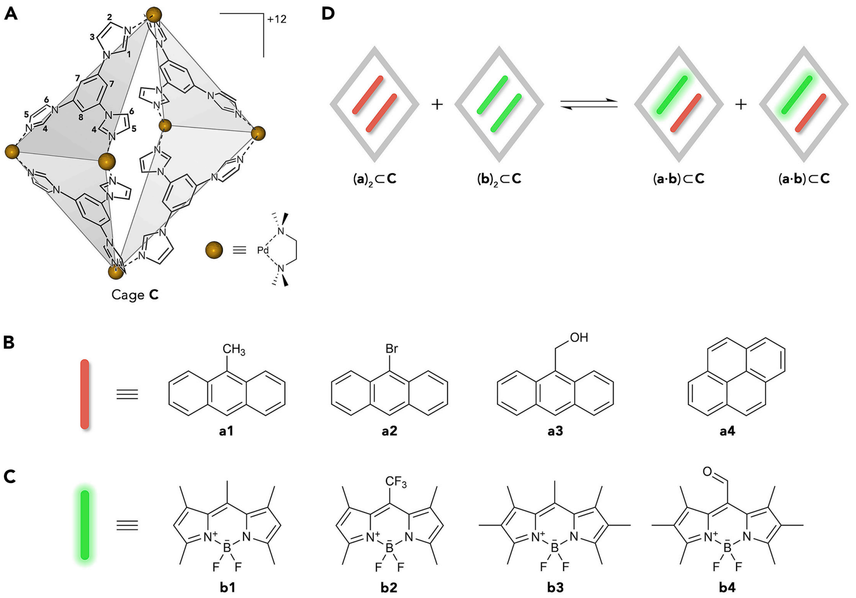

Scheme 1. Building blocks of noncovalent homodimers and BODIPY-PAH heterodimers inside the cavity of a coordination cage

(A) Structural formula of coordination cage **C**. Counterions = 12 NO_3^-.

(B) Structural formulas of PAHs used as guests for cage **C** (PAH = polycyclic aromatic hydrocarbon; parent or substituted).

(C) Structural formulas of BODIPY dyes used for heterodimer formation.

(D) Dynamic equilibrium between weakly fluorescent BODIPY homodimers, weakly fluorescent PAH homodimers, and strongly fluorescent BODIPY-PAH heterodimers.

RESULTS AND DISCUSSION

Encapsulation of polycyclic aromatic hydrocarbons (PAHs)

To encapsulate PAHs **a1**–**a4** within cage **C**, we stirred them (white powders insoluble in water; used in excess) with an aqueous solution of **C**. Encapsulation could be followed by UV-vis absorption spectroscopy, which showed a gradual increase of absorption patterns characteristic of the four PAHs (Figure S15). After 10 h of stirring, no further increase in the intensity of the absorption peaks was observed; we thus concluded that **C**'s cavities were saturated with the PAH guests. After discarding excess (undissolved) PAHs, the solutions of the inclusion complexes were characterized by NMR spectroscopy. As an example, Figure 1A (middle panel) shows the ^{1}H NMR spectrum of **a1** encapsulated within **C** (in D_2O). A comprehensive analysis using a suite of 2D NMR techniques (see Section 5 of the supplemental information) allowed us to assign all the signals in the 1D spectra of this complex (and the other PAH·**C** complexes). Compared with the spectrum of free **a1** in an organic solvent (Figure 1A, top), all the guest protons were upfield-shifted, which can be explained by their residence inside the hydrophobic cavity of the cage, where they experience magnetic shielding by the aromatic walls of the cage. Indeed, the largest shift of ~2.95 ppm was observed for the CH_3 group at the central position of the anthracene scaffold. Integrating the signals of the guest versus those of the cage allowed us to confirm that the stoichiometry of the complex is 2:1 and that it forms in a near-quantitative yield—i.e., practically all the cages could be filled with two guest molecules.

Diffusion-ordered NMR spectroscopy (DOSY) showed that all of **a1**'s and **C**'s protons diffused at the same rate, confirming that they constitute a single supramolecular entity (Figure S19). Furthermore, ^{1}H–^{1}H nuclear Overhauser effect spectroscopy

CellPress
OPEN ACCESS

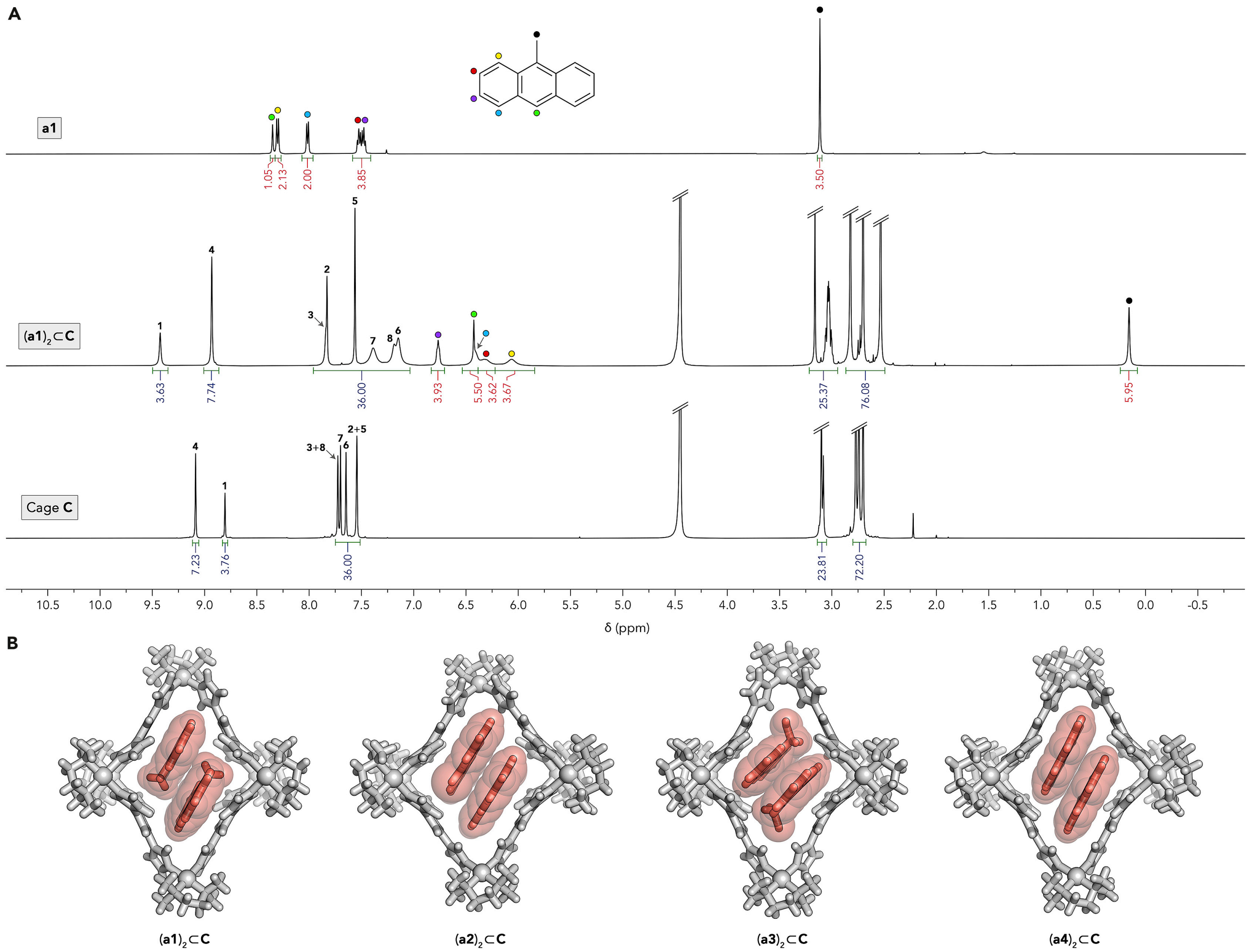

Figure 1. Formation of noncovalent homodimers of PAHs within cage C

(A) (Top) ^{1}H NMR spectrum of **a1** in $CDCl_3$ (500 MHz, 298 K). (Middle) ^{1}H NMR spectrum of (**a1**)$_2$⊂**C** in D_2O (600 MHz, 330 K). The integrals denoted in blue refer to **C**'s protons; those denoted in red refer to **a1**. The signals originating from **C**'s aromatic protons are denoted with numbers according to the numbering in Scheme 1A. (Bottom) ^{1}H NMR spectrum of **C** in D_2O (400 MHz, 330 K).

(B) X-ray crystal structures of homodimeric inclusion complexes (**a1**)$_2$⊂**C**, (**a2**)$_2$⊂**C**, (**a3**)$_2$⊂**C**, and (**a4**)$_2$⊂**C**. Nitrate counterions were omitted for clarity.

(NOESY) revealed multiple through-space interactions between **a1**'s and **C**'s protons. An in-depth analysis of the NOESY spectra provided important insights into (**a1**)$_2$⊂**C**'s solution structure. For example, we found that **a1**'s proton at position 10 (green in Figure 1A) showed a correlation with **C**'s acidic axial imidazole protons (denoted **1** in Scheme 1A and Figure 1A), whereas **a1**'s CH_3 correlated with **C**'s equatorial protons (**6** and **8** in Scheme 1A and Figure 1A). Together, these correlations suggest that the two encapsulated **a1** molecules are oriented antiparallel to each other, with their methyl groups residing in the equatorial area of the cage—an arrangement that was confirmed by single-crystal X-ray crystallography (see below). Detailed NMR characterization of (**a1**)$_2$⊂**C** and the other (**a**)$_2$⊂**C** complexes is presented in the supplemental information (Section 5).

Single crystals of all four (**a**)$_2$⊂**C** complexes were obtained by slow water evaporation from aqueous solutions of the respective complexes. Analysis of the X-ray

diffraction data confirmed the presence of two guest molecules inside the hydrophobic pocket of the cage (Figure 1B). The guests' planes were oriented parallel to each other and to two TImB walls of the cage, forming an extended TImB•••a•••a•••TImB π-π stack. Upon encapsulating guest molecules, the cage underwent significant axial elongation, with the distance between the two axial Pd nodes increasing from 16.9 Å (for empty **C**) to up to 18.6 Å (for (**a1**)$_2$⊂**C**), and the TImB-Pd-TImB angle at the axial Pd decreasing from 88.6° to 74.7°. Similar deformation was observed previously in the complexes of **C** with other small-molecule guests.[14,23,38–40,44] For further structural analysis of (**a1**)$_2$⊂**C** and the other (**a**)$_2$⊂**C** complexes, see Section 7 of the supplemental information.

Compared with free PAHs in organic solvents, absorption bands of encapsulated PAHs were broader, red-shifted by ~10 nm, and significantly dampened (Figures S84 and S85). Furthermore, encapsulation was accompanied by a substantial loss of fluorescence (Figure S86), as previously reported for noncovalent dimerization of BODIPY dyes within cage **C**.[23]

Formation and characterization of noncovalent BODIPY-PAH heterodimers

Having established that all four PAHs can form homodimeric inclusion complexes (**a**)$_2$⊂**C**, we focused on heterodimeric complexes incorporating both PAH and BODIPY, i.e., (**a**•**b**)⊂**C** (where "**a**" denotes any of the four PAHs and "**b**" denotes any of the four BODIPYs). In the initial experiments, we titrated aqueous solutions of (**b**)$_2$⊂**C** with (**a**)$_2$⊂**C** (at room temperature) and followed the reaction:

$$(\mathbf{a})_2{\subset}\mathbf{C} + (\mathbf{b})_2{\subset}\mathbf{C} \rightarrow 2\,(\mathbf{a}{\bullet}\mathbf{b}){\subset}\mathbf{C}$$

by UV-vis spectroscopy. For example, Figure 2A shows the results of titrating (**b1**)$_2$⊂**C** with (**a2**)$_2$⊂**C**, where the absorption at 480 nm due to (**b1**)$_2$⊂**C** declined at the expense of a new peak centered at 511 nm. Complex (**a2**)$_2$⊂**C** does not absorb in the visible region, and the new peak can be attributed to the heterodimer (**a2**•**b1**)⊂**C**. To maximize the fraction of **b1** within the heterodimer, we continued the titration until 4 equiv of (**a2**)$_2$⊂**C** were added. We also followed the titration using fluorescence spectroscopy and found a dramatic increase in the emission intensity (Figure 2B), with the emission spectrum of (**a2**•**b1**)⊂**C** resembling that of free **b1** in an organic solvent more than that of the weakly fluorescent (**b1**)$_2$⊂**C** H-dimer.[23]

Alternatively, the heterodimeric inclusion complexes could be formed by treating any (**b**)$_2$⊂**C** homodimer with solid **a** (and vice versa), according to the reaction equation:

$$(\mathbf{b})_2{\subset}\mathbf{C} + \mathbf{a}{\downarrow} \rightarrow (\mathbf{a}{\bullet}\mathbf{b}){\subset}\mathbf{C} + \mathbf{b}{\downarrow}$$

For example, stirring an aqueous solution of (**b1**)$_2$⊂**C** with an excess of **a2** (insoluble in water) resulted in a UV-vis spectrum very similar to that obtained by mixing (**b1**)$_2$⊂**C** with (**a2**)$_2$⊂**C**; at the same time, we observed that the white solid **a2** turned red, indicating partial expulsion of **b1** from the cage, followed by its precipitation from water (supplemental information, Section 10).

Depending on the identity of PAH and BODIPY, the equilibrium between the homodimers and the heterodimer could be shifted in either direction. For example, upon adding 4 equiv of (**a4**)$_2$⊂**C** to a solution of (**b1**)$_2$⊂**C**, an intense heterodimer peak at 510 nm was observed, accompanied by low absorption at 480 nm due to the residual homodimer (Figure S90C). When, however, (**b1**)$_2$⊂**C** was treated with 4 equiv of

CellPress
OPEN ACCESS

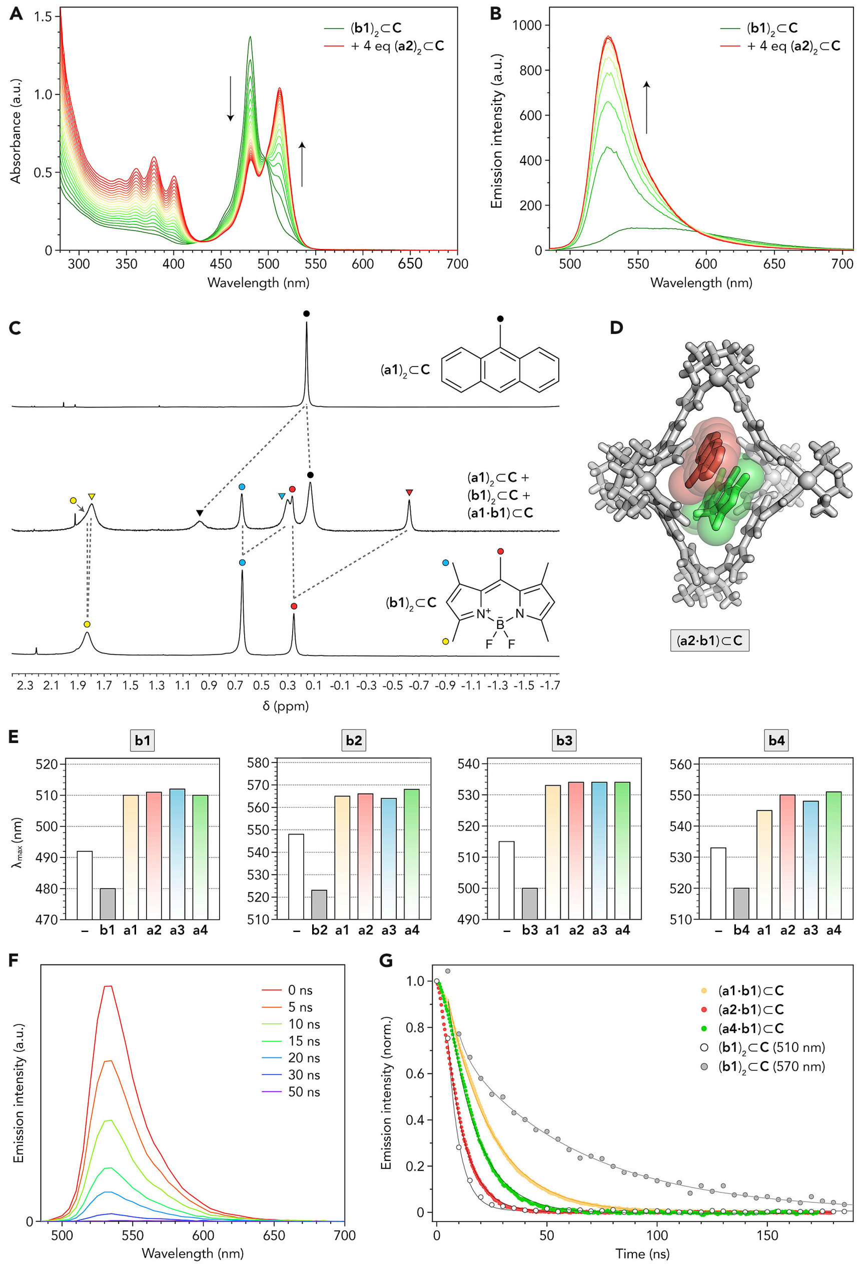

Figure 2. Formation and characterization of noncovalent BODIPY-PAH heterodimers

(A) Changes in the UV-vis absorption spectra of an aqueous solution of (**b1**)$_2$⊂**C** upon titration with (**a2**)$_2$⊂**C** (each spectrum corresponds to an additional 0.2 equiv of (**a2**)$_2$⊂**C**).

(B) Changes in the emission spectra of an aqueous solution of (**b1**)$_2$⊂**C** upon titration with (**a2**)$_2$⊂**C** (λ_{exc} = 460 nm; each spectrum corresponds to an additional 0.4 equiv of (**a2**)$_2$⊂**C**).

(C) Partial NMR spectra of (**a1**)$_2$⊂**C** (top; 600 MHz, 330 K), (**b1**)$_2$⊂**C** (bottom; 500 MHz, 298 K), and their 2:1 mixture (center; 600 MHz, 320 K) (all in D_2O), focusing on the aliphatic protons of the encapsulated guests. The circles denote protons of guests within the homodimers, and the triangles denote protons of guests within the heterodimer.

(D) X-ray crystal structure of (**a2•b1**)⊂**C** (red = **a2**, green = **b1**; counterions and guests' protons omitted for clarity).

(E) Wavelengths of maximum absorption (λ_{max}) of **b1**–**b4** as a function of the PAH co-guest within (**a•b**)⊂**C** ternary complexes (colored bars). Gray bars correspond to (**b**)$_2$⊂**C** homodimers; white bars correspond to free **b1**–**b4** dissolved in MeCN.

Figure 2. *Continued*

(F) Time-resolved fluorescence spectra of (**a2**•**b1**)⊂**C** in water (λ_{exc} = 460 nm) (to maximize the molar fraction of **b1** within the heterodimer, 40 equiv of (**a2**)$_2$⊂**C** with respect to (**b1**)$_2$⊂**C** were used).

(G) Fluorescence decay traces of **b1** (λ_{em} = 540 nm) within (**a1**•**b1**)⊂**C** (yellow), (**a2**•**b1**)⊂**C** (red), (**a4**•**b1**)⊂**C** (green). Also shown are fluorescence decays for (**b1**)$_2$⊂**C** at two different wavelengths (λ_{em} = 510 and 570 nm; empty and solid gray markers, respectively).

(**a1**)$_2$⊂**C** instead, the absorbance values at 480 and 510 nm were similar (despite the large excess of **a1**), indicating that the formation of heterodimer was less favorable (Figure S90A). Similarly, replacing one BODIPY with another for a given PAH significantly affected the homodimer/heterodimer ratio. This effect was particularly pronounced for **b2**, which has a strong tendency to form heterodimers. As shown in Figure S91A, the addition of only ~1 equiv of (**a1**)$_2$⊂**C** converted a vast majority of (**b2**)$_2$⊂**C** into (**a1**•**b2**)⊂**C**, in sharp contrast to the small amount of (**a1**•**b1**)⊂**C** formed by mixing the corresponding homodimers in a 1:1 ratio (Figure S90A).

The formation of encapsulated heterodimers could also be followed by NMR spectroscopy. Starting with homodimeric complexes of four PAHs and four BODIPYs (Scheme 1C), we obtained all 16 heterodimer combinations; NMR characterization of representative examples is shown in the supplemental information, Section 6. As an example, Figure 2C shows a partial ^{1}H NMR spectrum, obtained by mixing (**a1**)$_2$⊂**C** with (**b1**)$_2$⊂**C** in D_2O. In addition to the two homodimers, the spectrum shows a new set of peaks, which can be assigned to both guests residing within (**a1**•**b1**)⊂**C** (see the exchange correlations in Figure S66). Interestingly, upon heterodimer formation, **a1**'s and **b1**'s proton resonances were shifted in opposite directions (Figure 2C). Upon replacing one guest in (**a1**)$_2$⊂**C** with **b1**, the methyl protons of the remaining **a1** moved downfield by ~0.8 ppm. By contrast, **b1**'s methyl protons at the *meso* and β′ positions (red and blue in Figure 2C) shifted upfield (by ~0.9 and ~0.35 ppm, respectively). These results are a manifestation of a higher degree of aromaticity of anthracene compared with BODIPY, and they remind us that chemical shifts of encapsulated guests depend strongly not only on the host (here, cage **C**) but also on the co-guests with which they are co-confined. Notably, the splitting of **b1**'s methyl protons at the α position (yellow in Figure 2C) was much less pronounced—i.e., this singlet does not shift noticeably upon replacing one of the two **b1** within the (**b1**)$_2$⊂**C** homodimer with **a1**. This observation suggests that these protons do not reside directly above **a1**'s aromatic system—a conclusion that was confirmed by an X-ray structure of a similar heterodimer (see below).

To prove the existence of heterodimeric complexes (**a**•**b**)⊂**C** directly, we attempted to determine the X-ray crystal structure of a representative heterodimer. We focused on (**a2**•**b1**)⊂**C** since it contains the prototypical member of the BODIPY family—the penta-methyl-substituted **b1**—and a PAH with a high electron density on the Br substituent to facilitate structure determination. To this end, we mixed aqueous solutions of (**a2**)$_2$⊂**C** and (**b1**)$_2$⊂**C** and left the resulting solution undisturbed. Once most water had evaporated, we observed the formation of a mixture of colorless and orange crystals and manually collected the latter for X-ray diffraction. We found the coexistence of the (**b1**)$_2$⊂**C** homodimer (with a structure nearly identical to that reported previously[23]) and two different conformations of (**a2**•**b1**)⊂**C** in the crystal lattice. Both conformations featured a TImB•••**a2**•••**b1**•••TImB π-π stack, with **b1**'s BF_2 moiety facing the axial Pd nodes (as in the structure of pure (**b1**)$_2$⊂**C**[23]), but they differed in the orientation of **a2**. In the first conformer, **a2**'s Br substituent pointed toward the equatorial area of the cage, as shown in Figure 2D; in the second,

it was oriented in the opposite direction, toward the axial region (Figure S80). Density functional theory (DFT) calculations suggested that the latter conformer is slightly more stable (supplemental information, Section 8). We also attempted to detect the ternary complex (**a2**•**b1**)⊂**C** by electrospray ionization mass spectrometry (ESI-MS), but measurements under various conditions repeatedly showed the empty cage and the two guests as separate species. Similarly, no signals for the intact homodimeric complexes $(\mathbf{a2})_2$⊂**C** and $(\mathbf{b1})_2$⊂**C** were detected. These results are in agreement with the fast expulsion of guests through the large windows of cage **C** during mass spectrometry measurements.

Next, we studied how varying the PAH guest within (**a**•**b**)⊂**C** heterodimers affects the optical properties of the encapsulated BODIPY. As described above, replacing one of two BODIPYs in $(\mathbf{b})_2$⊂**C** homodimers with a PAH dramatically red-shifts the absorption maximum of the remaining BODIPY (by ~30 nm for **b1**, **b3**, and **b4**, and by ~40 nm for **b2**; gray versus colored bars in Figure 2E). Interestingly, however, remarkably little variation was found when one PAH was replaced with another for a given BODIPY; for example, **b1** within all four heterodimers absorbed at 511 nm (±1 nm) (colored bars in Figure 2E). Whereas the fluorescence emission for all (**a**•**b1**)⊂**C** complexes was centered at 528 nm, the PAHs had a large effect on **b1**'s fluorescence quantum yields (Φ_F = 0.31, 0.21, 0.41, and 0.50 for **b1** co-encapsulated with **a1**, **a2**, **a3**, and **a4**, respectively; Φ_F = 0.13 for the $(\mathbf{b1})_2$⊂**C** homodimer[23] and 1.00 for free **b1** in chloroform[59]). Similarly, we observed significant differences in the fluorescence decay profiles. Figure 2F shows a typical series of time-resolved fluorescence spectra recorded after exciting (**a2**•**b1**)⊂**C** with a 5-ns 460 nm pulse. The fluorescence decay can be fitted to a single-exponential curve (Figure 2G) with a time constant, τ = 8.66 (±0.43) ns (the errors correspond to deviations from the ideal single-exponential curve). This behavior is reminiscent of that of free **b1** in MeCN, whose fluorescence decays with τ = 6.71 (±0.15) ns,[23] but stands in sharp contrast with the $(\mathbf{b1})_2$⊂**C** homodimer, which exhibits a more complex decay profile with a slower decay at longer wavelengths (570 nm), indicative of H-aggregation.[23] For (**a1**•**b1**)⊂**C** and (**a4**•**b1**)⊂**C**, we determined τ as 13.26 (±0.07) and 21.83 (±0.13) ns, respectively.

Kinetics of guest exchange

Next, we studied the kinetics of heterodimer formation via guest exchange, $(\mathbf{a})_2$⊂**C** + $(\mathbf{b})_2$⊂**C** → 2 (**a**•**b**)⊂**C**, hypothesizing that changing the bulkiness of either guest might have a large effect on the reaction kinetics. In the initial experiments, we injected aqueous solutions of $(\mathbf{a})_2$⊂**C** into solutions of $(\mathbf{b})_2$⊂**C** and followed the reaction with the naked eye. Working with two relatively small guests **a2** and **b1**, we found that the mixture turned strongly fluorescent instantaneously (Figures 3A and S94). However, upon replacing **a2** and **b1** with the bulkier **a4** and **b3**, respectively, the system required several minutes to equilibrate (Figure 3B; see the gradual change in emission color and the gradual increase of emission intensity, which can be appreciated from the vial's reflection on the benchtop).

The finding that the optical properties of **b3** (and other BODIPY dyes) depend strongly on the identity of the guest with which it shares the cavity of **C** allowed us to conveniently monitor the reaction using UV-vis absorption spectroscopy. Figure 3C shows the evolution of UV-vis spectra observed upon treating $(\mathbf{b3})_2$⊂**C** with 10 equiv of $(\mathbf{a4})_2$⊂**C**; the 500 nm absorption peak due to $(\mathbf{b3})_2$⊂**C** decreased over 2 min, whereas the 534 nm peak due to (**a4**•**b3**)⊂**C** gradually increased. Interestingly, **a4**'s absorbance in the near-UV region remained largely unaltered

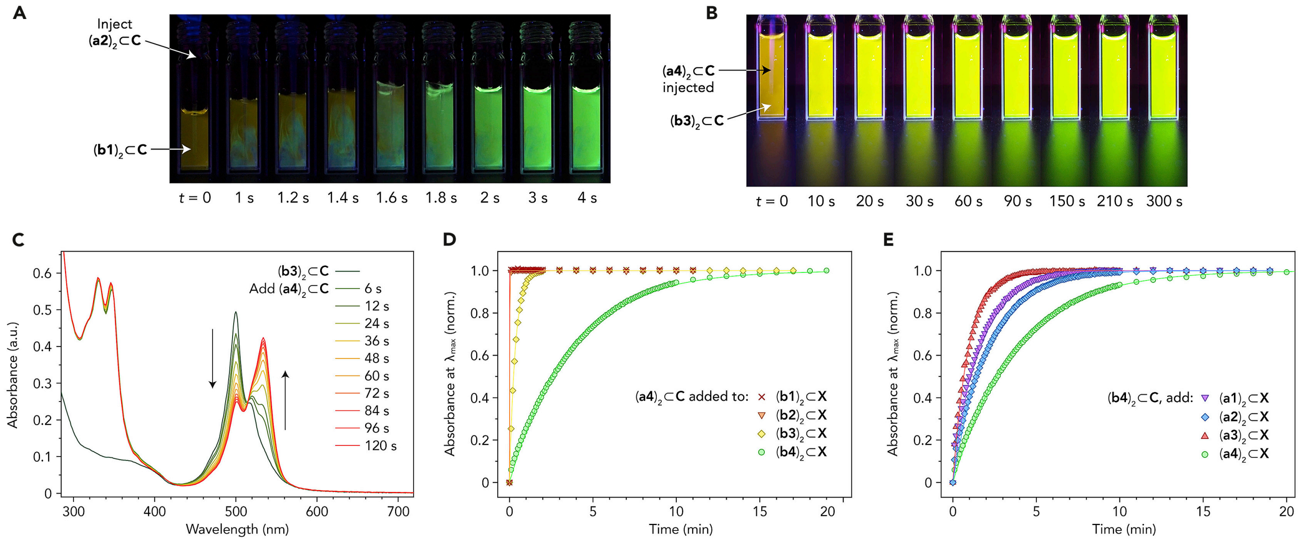

Figure 3. Kinetics of heterodimer formation via guest exchange

(A) Photographs taken during and immediately after the injection of 2 equiv of $(\mathbf{a2})_2{\subset}\mathbf{C}$ in water into an aqueous solution of $(\mathbf{b1})_2{\subset}\mathbf{C}$.

(B) Photographs taken at various times following the injection of 10 equiv of $(\mathbf{a4})_2{\subset}\mathbf{C}$ in water into an aqueous solution of $(\mathbf{b3})_2{\subset}\mathbf{C}$.

(C) Changes in the UV-vis absorption spectra of an aqueous solution $(\mathbf{b3})_2{\subset}\mathbf{C}$ following injection of 10 equiv of $(\mathbf{a4})_2{\subset}\mathbf{C}$ in water.

(D) Monitoring the formation kinetics of the four $(\mathbf{a4}{\bullet}\mathbf{b}){\subset}\mathbf{C}$ heterodimers by following the absorbance at the wavelength of maximum absorption (λ_{max}) of each heterodimer. For **b1** and **b2**, a near-instantaneous equilibration was observed. Markers: experimental data points; lines: fits to an asymptotic exponent $1 - e^{-kt}$.

(E) Monitoring the formation kinetics of the four $(\mathbf{a}{\bullet}\mathbf{b4}){\subset}\mathbf{C}$ heterodimers by following the absorbance at each heterodimer's λ_{max}. Markers: experimental data points; lines: fits to an asymptotic exponent $1 - e^{-kt}$.

throughout the reaction (compare the spectra at 6 versus 120 s). However, the visible part of the spectrum shows that the reaction $(\mathbf{a4})_2{\subset}\mathbf{C} + (\mathbf{b3})_2{\subset}\mathbf{C} \rightarrow 2\,(\mathbf{a4}{\bullet}\mathbf{b3}){\subset}\mathbf{C}$ proceeds to a negligible extent within the initial 6 s. Together, these observations allow us to conclude that the optical properties of pyrene, unlike those of BODIPYs, show little dependence on its co-guest (see also Figure S96E for the **a4**/**b4** combination). The same was found to be true for anthracenes **a1**–**a3**—see, e.g., Figure S97A.

To determine whether the dramatic deceleration of guest exchange (compare Figure 3A with 3B) was caused primarily by increasing the bulkiness of PAH (**a2** to **a4**) or that of BODIPY (**b1** to **b3**), we varied the identity of BODIPY for a given PAH and vice versa. First, we injected $(\mathbf{a4})_2{\subset}\mathbf{C}$ into all four $(\mathbf{b})_2{\subset}\mathbf{C}$ homodimers and monitored the exchange by following the absorbance at the wavelength of maximum absorption of each $(\mathbf{a4}{\bullet}\mathbf{b}){\subset}\mathbf{C}$ complex (Figure 3D). Whereas **b1** and **b2** equilibrated very rapidly and **b3** took ~2 min to equilibrate, the bulkiest **b4** needed more than 20 min to reach equilibrium. By fitting the data to a single exponential function $y = 1 - e^{-kt}$ (see supplemental information, Section 11 for derivation), we determined the equilibration rates as k = 51, 2.8, and 0.26 min^{-1} for **b1**, **b3**, and **b4**, respectively. Notably, with **b2**, the reaction proceeded even faster than it did for the smaller **b1** and reached completion by the time the first data point (t = 6 s) was recorded; this finding can be explained by the relatively low stability of the $(\mathbf{b2})_2{\subset}\mathbf{C}$ homodimer with respect to **b2**'s complexes with PAHs (see above). In the second set of experiments, we injected all four $(\mathbf{a})_2{\subset}\mathbf{C}$ homodimers into four identical solutions of $(\mathbf{b4})_2{\subset}\mathbf{C}$ and monitored the absorbance at the wavelength of maximum absorption of the respective $(\mathbf{a}{\bullet}\mathbf{b4}){\subset}\mathbf{C}$ complexes (Figure 3E). Interestingly, varying the PAH had relatively little influence on guest exchange kinetics

(at least in this series); we found $k = 0.65, 0.48, 0.27$, and $1.1\ min^{-1}$ for **a1**, **a2**, **a3**, and **a4**, respectively.

Having demonstrated that replacing one guest molecule in a $(\mathbf{b})_2\subset\mathbf{C}$ complex with a PAH greatly increased the emission intensity of the encapsulated BODIPY, we hypothesized that selectively removing the PAH component from $(\mathbf{a}\bullet\mathbf{b})\subset\mathbf{C}$ could shift the equilibrium back toward $(\mathbf{b})_2\subset\mathbf{C}$, thus restoring the initial, low emission of the solution. An elegant way to effectively remove an anthracene from the system is to convert it into a covalent dimer, which can be achieved by irradiation with near-UV (~365 nm) light (Figure 4A). We further speculated that the resulting dianthracene would occupy the entire cavity of cage **C**. Thus, covalent dimerization of an anthracene is expected to force noncovalent dimerization of BODIPY dye. To verify this hypothesis, we first studied the ability of anthracenes confined within cage **C** to photodimerize.

Photoresponsiveness of encapsulated PAHs

Upon UV irradiation, anthracenes undergo [4+4] cycloaddition to afford dianthracenes (Figure 4A)—a reaction that has been investigated extensively in solution[60–62] and in confined spaces.[63–68] We hypothesized that encapsulation within the cage will not only increase the effective molarity of anthracenes but might also preorganize them in a way that allows for favorable orbital overlap, thus greatly increasing the reaction kinetics. Figure 4B shows a series of UV-vis spectra recorded after exposing $(\mathbf{a2})_2\subset\mathbf{C}$ in water to increasing periods of near-UV light. After 5 min of irradiation, anthracene's characteristic absorption pattern disappeared, indicating complete conversion to the corresponding dianthracene, which we denote **a2a2**. No precipitation was observed, suggesting that the product remained encapsulated as $(\mathbf{a2a2})\subset\mathbf{C}$. Interestingly, the reaction proceeded significantly faster than the photodimerization of free **a2** dissolved in DCM, which required UV exposure of >30 min under the same irradiation conditions for **a2**'s absorption to fully disappear (Figure S99). By plotting the absorption of **a2** over time and fitting the data to a first-order decay,[69] we found $k = 2.44$ and $59.5\ h^{-1}$ for the free and encapsulated **a2**, respectively, corresponding to reaction acceleration by a factor of ~25 (Figure 4C).

In addition to the increased rate, the reaction proceeded cleaner, leaving behind a nearly featureless spectrum above 320 nm (Figure 4B). By contrast, photodimerization of free **a2** in DCM was accompanied by a gradual increase of absorption at ~325 nm (Figure S99), indicating the formation of a side product, most likely a bianthracenyl.[70] The high selectivity of photodimerization of confined **a2** could also be appreciated from NMR spectra of $(\mathbf{a2})_2\subset\mathbf{C}$ (3 mM in D_2O) exposed to UV light. Following 6 min of UV irradiation, the signals originating from encapsulated **a2** disappeared and were replaced by a new set of signals in a 1:1:1:1 ratio (Figures S100 and S101) due to the aromatic protons of encapsulated **a2a2**. Only one aliphatic signal was observed, indicating the formation of a single isomer of dianthracene (in our case, head-to-tail (*h-t*)-**a2a2**). After a total of 7 min of irradiation, $CDCl_3$ was added, and the reaction product was extracted from the aqueous phase. An NMR spectrum of the organic phase revealed the presence of remarkably clean *h-t*-**a2a2**—in sharp contrast to free **a2** in $CDCl_3$, whose irradiation for a longer time (160 min) led to only ~65% of **a2a2**, in addition to side products (Figure S102).

Interestingly, the reaction $(\mathbf{a2})_2\subset\mathbf{C} \rightarrow (\mathbf{a2a2})\subset\mathbf{C}$ was accompanied by pronounced changes in the chemical shifts of cage **C**'s protons (Figure S100), in particular, the acidic imidazole protons (**1** and **4** in Scheme 1A). These changes indicate a large structural distortion of the cage, which it has to undergo to adapt to **a2a2**, whose

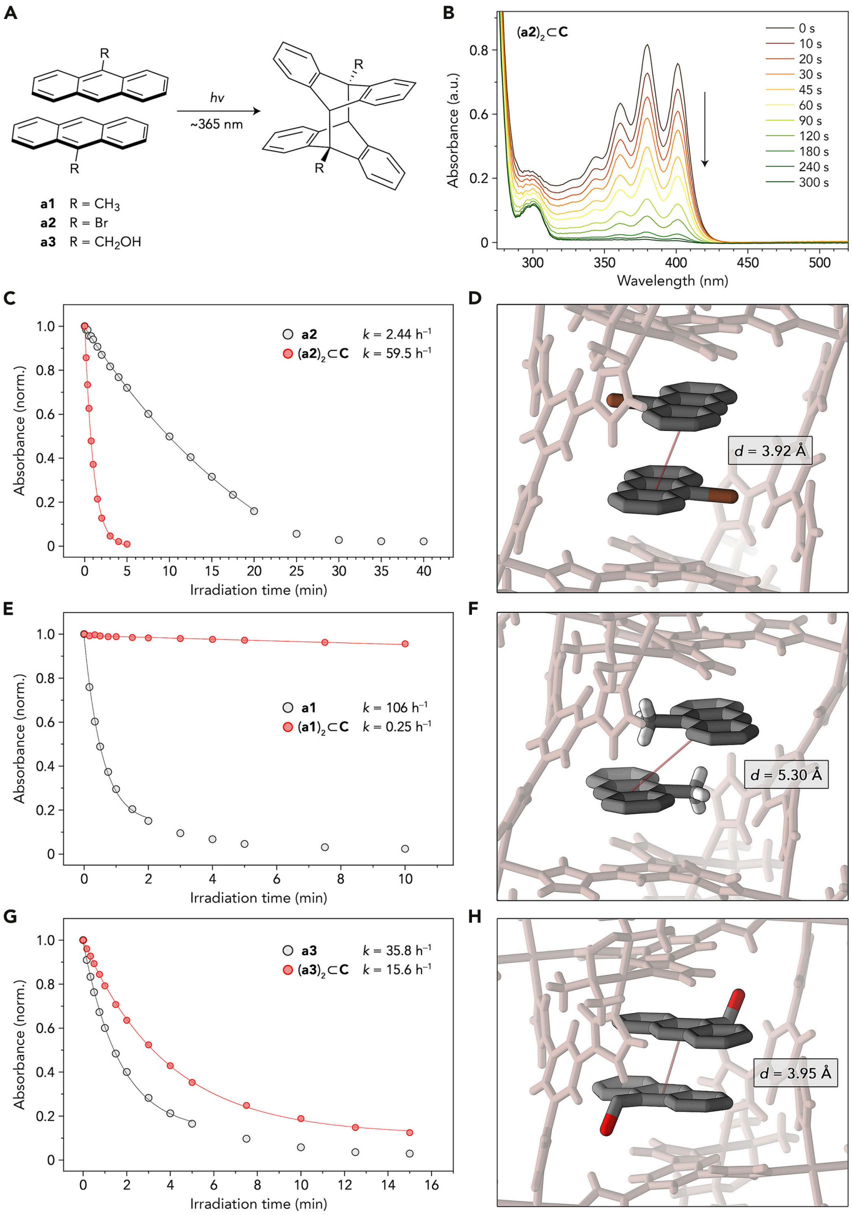

Figure 4. [4+4] photodimerization of confined anthracenes

(A) Reaction scheme.

(B) Changes in the UV-vis absorption spectra of an aqueous solution of (**a2**)$_2$⊂**C** exposed to UV (365 nm) light.

(C) Monitoring the [4+4] photodimerization of free **a2** in DCM (gray) and (**a2**)$_2$⊂**C** in water (red) by following absorbance at 393 and 401 nm, respectively. Markers: experimental data points; lines: fits to the kinetic equation for a first-order reaction ($y = e^{-xk}$; note that photodimerization in DCM was accompanied by a slower, side reaction leading to a byproduct whose absorption overlapped with that of anthracene; therefore, only the initial points were used for obtaining the fits; also for E and G).

(D) Excerpt from the X-ray crystal structure of (**a2**)$_2$⊂**C**, focusing the orientation of guests within **C** (counterions and guests' protons omitted for clarity; also for F and H).

(E) Monitoring the [4+4] photodimerization of free **a1** in DCM (gray) and (**a1**)$_2$⊂**C** in water (red) by following absorbance at 390 and 398 nm, respectively. Markers: experimental data points; lines: fits to a first-order reaction.

(F) Excerpt from the X-ray crystal structure of (**a1**)$_2$⊂**C**, focusing the orientation of guests within **C**.

Figure 4. *Continued*

(G) Monitoring the [4+4] photodimerization of free **a3** in DCM (gray) and $(\mathbf{a3})_2 \subset \mathbf{C}$ in water (red) by following absorbance at 387 and 393 nm, respectively. Markers: experimental data points; lines: fits to a first-order reaction.

(H) Excerpt from the X-ray crystal structure of $(\mathbf{a3})_2 \subset \mathbf{C}$, focusing the orientation of guests within **C** (one of two conformers in the crystal; the structure of the second conformer is very similar, with a center-to-center distance of 3.85 Å; see CCDC: 2103576).

shape is significantly different[71] from that of two stacked **a2** molecules. In fact, following UV irradiation, we observed slow precipitation of a white solid as a result of the expulsion of **a2a2** from **C**. No such precipitation was observed in UV-vis experiments, which were conducted at much lower (micromolar) concentrations. These results explain why, despite extensive efforts, we did not succeed in obtaining single crystals of $(\mathbf{a2a2}) \subset \mathbf{C}$ suitable for X-ray diffraction.

To determine whether the encapsulation-induced acceleration of [4+4] cycloaddition is a general phenomenon, we studied the behavior of encapsulated **a1**. Surprisingly, UV irradiation of $(\mathbf{a1})_2 \subset \mathbf{C}$ resulted in very small changes in the UV-vis spectra, indicating that the cycloaddition reaction is significantly hampered—despite **C**'s ability to bring two copies of **a1** into close proximity. By fitting the collected data points to a first-order decay (Figure 4E), we obtained k = 0.25 h^{-1}, corresponding to reaction deceleration (compared with free **a1** in DCM) by a factor of >400.

The X-ray structures of $(\mathbf{a1})_2 \subset \mathbf{C}$ and $(\mathbf{a2})_2 \subset \mathbf{C}$ provide insights into the contrasting impact of confinement on the photoreactivity of these two complexes (Figures 4D and 4F). In both cases, the guest molecules are oriented antiparallel to each other, with the same plane-to-plane distance of 3.54 Å (the distance between the planes defined by the anthracenes' central rings). However, in the case of $(\mathbf{a1})_2 \subset \mathbf{C}$, the guests were significantly offset with respect to each other, with a center-to-center distance of as much as 5.30 Å (compared with 3.92 Å for **a2**; the center-to-center distance is defined as the distance between the centroids of the central rings of the two anthracenes). The offset orientation in $(\mathbf{a1})_2 \subset \mathbf{C}$, likely stabilized by C–H•••π interactions between the two guest molecules, effectively suppresses the reaction. These results indicate that the ''topochemical postulate,'' originally formulated for the solid state,[72,73] applies to molecules confined within the cavities of soluble cages as well. To support these results, we also worked with **a3**; upon UV irradiation, [4+4] cycloaddition within $(\mathbf{a3})_2 \subset \mathbf{C}$ took place (Figure 4G), albeit ~4 times slower than in the case of $(\mathbf{a2})_2 \subset \mathbf{C}$. The X-ray structure of $(\mathbf{a3})_2 \subset \mathbf{C}$ features two conformers of the inclusion complex, with center-to-center distances of 3.95 (Figure 4H) and 3.85 Å, rendering the photodimerization reaction topochemically allowed.[72,73]

Noncovalent heterodimers with photoswitchable fluorescence

Finally, we integrate the two findings described above—fast guest exchange and efficient cycloaddition of encapsulated anthracenes—to construct a supramolecular system exhibiting light-switchable fluorescence (Figure 5A). Having identified **a2** as an anthracene that undergoes a rapid cyclodimerization reaction and having determined that guests **a2** and **b1** shuttle between different cages rapidly, we speculated that the equilibrium between the highly fluorescent **a2•b1** and the weakly fluorescent $(\mathbf{b1})_2$ H-dimer could be shifted toward the latter by effectively removing **a2** from the system as the covalent dimer **a2a2**:

$$2\,(\mathbf{a2{\bullet}b1}) \subset \mathbf{C} \xrightarrow{\mathrm{UV}} (\mathbf{a2a2}) \subset \mathbf{C} + (\mathbf{b1})_2 \subset \mathbf{C}$$

To this end, we mixed $(a2)_2 \subset C$ with $(b1)_2 \subset C$ in a 4:1 ratio, thus placing a vast majority of **b1** within the $(a2 \cdot b1) \subset C$ heterodimer (red spectrum in Figure 5B). Upon near-UV (365 nm) irradiation, the 511 nm peak decreased, whereas the 480 nm peak grew, indicating regeneration of $(b1)_2 \subset C$. The system reached equilibrium within ~8 min of UV irradiation, corresponding to the time required to complete the [4+4] cycloaddition reaction, thus confirming that the rate-determining step was the cycloaddition rather than guest exchange. As expected, no changes in the UV-vis spectra were observed when **a2** was replaced with either **a1** (which dimerizes very slowly; Figure S107A) or **a4** (photochemically inactive; Figure S107B). However, replacing **b1** with other BODIPYs afforded systems that did exhibit the photoinduced modulation of optical properties—see Figures S107C and S107D for the **a2**/**b2** and **a2**/**b4** combinations, respectively. The reaction could also be followed by fluorescence spectroscopy, where the emission at 528 nm decreased by more than 12-fold (Figure 5C).

Next, we hypothesized that by UV-irradiating a sample containing the highly emissive $(a2 \cdot b1) \subset C$ locally (i.e., through a mask), it should be possible to create fluorescent patterns. To this end, we first prepared three thin (10 × 10 × 1 mm) pieces of agarose hydrogels and soaked them in an aqueous solution of $(b1)_2 \subset C$; the gels exhibited weak orange emission. Then, the gels were transferred into vials containing the following: a solution of $(a2)_2 \subset C$ (sample I), pure water (sample II), and a solution of $(a4)_2 \subset C$ (sample III). After soaking for 1 h, gels I and III assumed bright green fluorescence, owing to the formation of heterodimers $(a2 \cdot b1) \subset C$ and $(a4 \cdot b1) \subset C$, respectively (Figure 5D). Then, all three gels were exposed to near-UV light (365 nm) for 3 min. As expected, UV irradiation had no effect on gels II and III; the former did not contain any PAH, and the latter contained the photochemically inactive **a4**. However, irradiation of gel I triggered the formation of $(a2a2) \subset C$, thus forcing **b1** into the weakly fluorescent $(b1)_2 \subset C$ homodimer; consequently, the emission of this sample, after exposure to UV, resembled that of the control gel II (Figure 5D). To demonstrate the ability to pattern gels containing the light-responsive heterodimer, we prepared a larger (35 × 25 × 1 mm) piece of agarose gel soaked with a 4:1 mixture $(a2)_2 \subset C$ and $(b1)_2 \subset C$ and brought it into conformal contact with a mask. UV irradiation induced the [4+4] cycloaddition coupled with guest exchange only in the exposed regions, decreasing emission intensity and turning the emission color orange (Figure 5E).

Anthracene cyclodimerization can be reversed upon irradiation with UV light of higher energy (<300 nm). Therefore, we hypothesized that the homodimer/heterodimer ratio—and, consequently, the solution's emission intensity—could be tuned reversibly using UV light of two different wavelengths. Indeed, when a 4:1 mixture of encapsulated dianthracene $(a2a2) \subset C$ and $(b1)_2 \subset C$ was exposed to 254 nm light for 30 min, a substantial amount of the original anthracene **a2**—and, consequently, of the fluorescent $(a2 \cdot b1) \subset C$ heterodimer—was regenerated (Figure S108). Unfortunately, the dedimerization reaction did not reach completion, and further deterioration of the system was observed in the subsequent cycles (Figure 5F). It is important to emphasize that the observed fatigue was most likely caused by the inherent instability of **a2**—rather than of host **C**—under 254 nm light (Figure S103); in fact, we previously reported highly reversible switching of another photoresponsive compound (dihydropyrene) within **C**, despite prolonged exposure to 254 nm light.[14] To address the problem of photodegradation induced by 254 nm light, we developed an alternative system based on cycles of (1) irradiation with 365 nm light and (2) addition of a fresh aliquot of $(a2)_2 \subset C$ (4 equiv). Completing each cycle "resets" the system to the initial, highly fluorescent

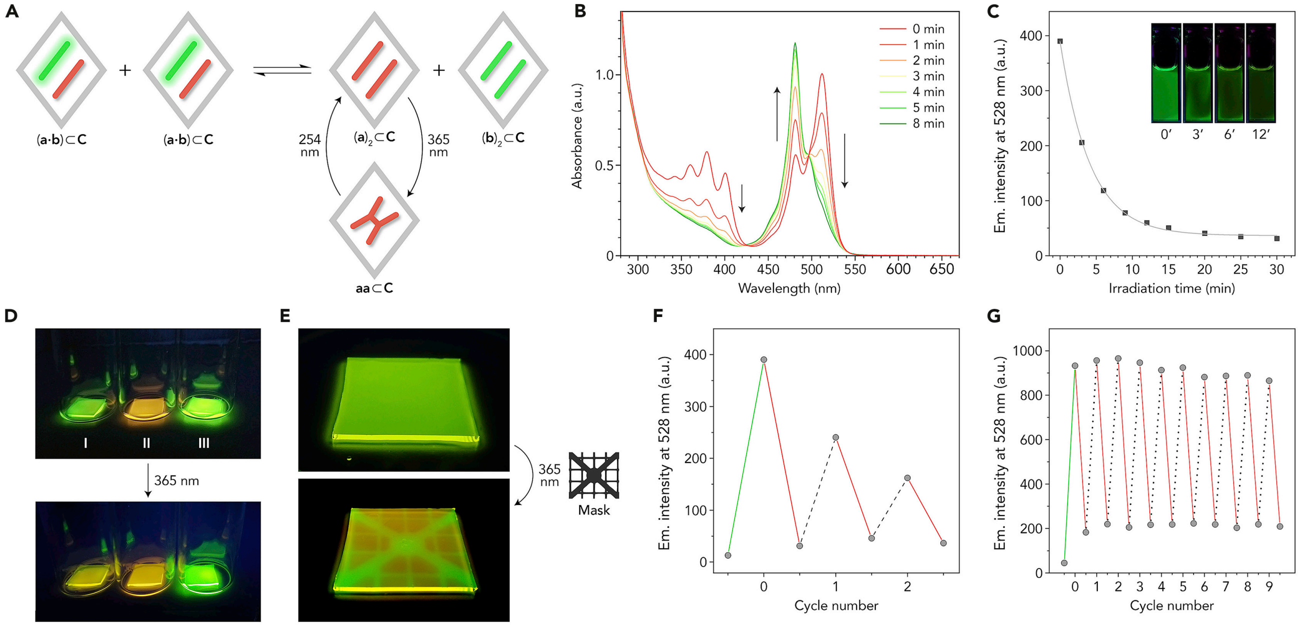

Figure 5. Controlling the homodimer/heterodimer equilibrium with light

(A) Shifting the equilibrium between strongly fluorescent BODIPY heterodimers (left) and weakly fluorescent homodimers (right) using light-responsive anthracenes.

(B) Changes in the UV-vis absorption spectra of a 4:1 mixture of (**a2**)$_2$⊂**C** and (**b1**)$_2$⊂**C** induced by UV (365 nm) irradiation for up to 8 min.

(C) Decrease of emission intensity of a 4:1 mixture of (**a2**)$_2$⊂**C** and (**b1**)$_2$⊂**C** induced by UV (365 nm) irradiation for up to 30 min (see Figure S109 for the original spectra). Markers: experimental data points; curve: fit to a single exponential decay (τ = 4.17 ($\pm$0.11) min). Inset: photographs showing gradual disappearance of green fluorescence during exposure to UV light for up to 12 min.

(D) Photographs of 10 × 10 × 1 mm pieces of agarose gels soaked with: (**a2**)$_2$⊂**C** + (**b1**)$_2$⊂**C** (gel I), (**b1**)$_2$⊂**C** (gel II), and (**a4**)$_2$⊂**C** + (**b1**)$_2$⊂**C** (gel III) before (top) and after (bottom) UV irradiation for 3 min.

(E) Photographs of a 35 × 25 × 1 mm piece of agarose gel soaked with a 4:1 mixture of (**a2**)$_2$⊂**C** and (**b1**)$_2$⊂**C** before (top) and after (bottom) UV irradiation through a mask.

(F) Reversible changes in the emission intensity of a 4:1 mixture of (**a2**)$_2$⊂**C** and (**b1**)$_2$⊂**C** upon alternating irradiation with 254 nm light for 25 min (denoted with dashed black lines) and 365 nm light for 12 min (solid red lines). The first data point corresponds to (**b1**)$_2$⊂**C** and the green line corresponds to addition of 4 equiv (**a2**)$_2$⊂**C**.

(G) Reversible changes in the emission intensity of a 4:1 mixture of (**a2**)$_2$⊂**C** and (**b1**)$_2$⊂**C** upon alternating irradiation with 365 nm light for 25 min (denoted with red lines) and addition of 4 equiv (**a2**)$_2$⊂**C** (dotted black lines). The first data point corresponds to (**b1**)$_2$⊂**C** and the green line—addition of 4 equiv of (**a2**)$_2$⊂**C**.

state: (**b1**)$_2$⊂**C** + (**a2**)$_2$⊂**C** → 2 (**a2**•**b1**)⊂**C**. Although each cycle accumulates (**a2a2**)⊂**C**, this species is not fluorescent, unresponsive to 365 nm UV light, and, being a 1:1 complex, it does not interfere with the **b1** homodimer/heterodimer equilibrium; thus, it behaves as a non-invasive waste. Indeed, this strategy allowed us to perform at least ten on/off cycles without noticeable fatigue (Figure 5G).

Conclusions

In summary, we developed a supramolecular system based on a combination of three components—a coordination cage, BODIPY dyes, and aromatic hydrocarbons (PAHs)—that allowed us to study the dynamics of host-guest inclusion complexes using optical spectroscopies. The cage's cavity is of a size that enables the simultaneous encapsulation of two guest molecules—either two BODIPYs, two PAHs, or a BODIPY-PAH heterodimer. Mixing a BODIPY homodimer with a PAH homodimer initiates guest exchange, resulting in a heterodimer with distinct absorption and fluorescence features. We found that, depending on the identity of BODIPY and PAH, heterodimer formation can be either instantaneous or require tens of minutes to complete. In parallel, we investigated the UV-induced cyclodimerization of the

confined PAHs (anthracenes). Compared with anthracenes dissolved in an organic solvent, the cyclodimerization of confined anthracenes could be either dramatically accelerated or remarkably decelerated, depending on the substitution pattern on the anthracene ring; these results could be rationalized by the "topochemical postulate." Finally, we integrated the UV-induced cyclodimerization reaction with the rapidly equilibrating inclusion complexes; beyond the development of a conceptually novel light-controlled fluorescence switch, this unique combination of covalent and noncovalent reactions brings supramolecular host-guest inclusion complexes closer to the realm of systems chemistry.

EXPERIMENTAL PROCEDURES

Full experimental procedures can be found in the supplemental information.

Resource availability

Lead contact

Further information and requests for resources and reagents should be directed to the lead contact, Rafal Klajn (rafal.klajn@weizmann.ac.il).

Materials availability

This study did not generate unique reagents.

Data and code availability

All data supporting the findings of this study are included within the article and its supplemental information and are also available from the authors upon request. Crystallographic data for the structures reported in this paper have been deposited at the Cambridge Crystallographic Data Centre, under the deposition numbers CCDC 2103596 for $(\mathbf{a1})_2\subset\mathbf{C}$, CCDC 2103597 for $(\mathbf{a2})_2\subset\mathbf{C}$, CCDC 2103576 for $(\mathbf{a3})_2\subset\mathbf{C}$, CCDC 2103598 for $(\mathbf{a4})_2\subset\mathbf{C}$, and CCDC 2103596 for $(\mathbf{a2}\bullet\mathbf{b1})\subset\mathbf{C}$. Copies of these data can be obtained free of charge via www.ccdc.cam.ac.uk/data_request/cif.

General procedure for the formation of homodimeric and heterodimeric inclusion complexes

A solution of cage **C** (10.0 mg, 3.1 μmol) in H_2O or D_2O (0.5 mL) was added to an excess (5–10 equiv) of solid PAH (**a1**, **a2**, **a3**, or **a4**) or BODIPY (**b1**, **b2**, **b3**, or **b4**) and the resulting suspension was stirred overnight at room temperature in the dark (all the PAHs and BODIPYs are insoluble in water in the unencapsulated form). The undissolved solids were removed by several cycles of centrifugation or by filtration through a syringe filter to afford solutions of homodimeric inclusion complexes $(\mathbf{a})_2\subset\mathbf{C}$ and $(\mathbf{b})_2\subset\mathbf{C}$. Heterodimeric inclusion complexes $(\mathbf{a}\bullet\mathbf{b})\subset\mathbf{C}$ were formed by mixing aqueous solutions of the respective homodimeric complexes $(\mathbf{a})_2\subset\mathbf{C}$ and $(\mathbf{b})_2\subset\mathbf{C}$. The resulting solutions of homodimeric and heterodimeric inclusion complexes were stable in the dark at ambient temperature for at least several months.

SUPPLEMENTAL INFORMATION

Supplemental information can be found online at https://doi.org/10.1016/j.chempr.2022.05.008.

ACKNOWLEDGMENTS

We acknowledge funding from the European Union's Horizon 2020 Research and Innovation Program under the European Research Council (ERC) grant agreement no. 820008 and the Marie Skłodowska-Curie grant agreement no. 812868. This

research was supported in part by the "Excellence Initiative – Research University" program at the University of Wrocław. DFT calculations were carried out using resources provided by the Wrocław Center for Networking and Supercomputing, grant 329. We gratefully acknowledge Dr. Miłosz Siczek (Department of Chemistry, University of Wrocław) for collecting X-ray crystallographic data for $(\mathbf{a3})_2{\subset}\mathbf{C}$ and $(\mathbf{a2{\cdot}b1}){\subset}\mathbf{C}$ and Dr. Alla Falkovich (Department of Chemical Research Support, Weizmann Institute of Science) for assistance with mass spectrometry measurements.

AUTHOR CONTRIBUTIONS

J.G. and R.K. conceived the study and designed the experiments. J.G. and M.J.B. performed the experiments. M.J.B., L.J.W.S., and Y.D.-P. determined the X-ray structures of the inclusion complexes. J.G. and M.K. performed the transient spectroscopy experiments. J.G., M.K., and D.O. analyzed the results of the transient spectroscopy experiments. M.J.B. carried out the DFT calculations. S.N.S. derived the kinetic rate equations. J.G. and R.K. wrote the manuscript. All authors discussed the results and commented on the manuscript.

DECLARATION OF INTERESTS

R.K. is a member of *Chem*'s advisory board.

Received: December 23, 2021
Revised: February 18, 2022
Accepted: May 11, 2022
Published: June 30, 2022

REFERENCES

1. Grommet, A.B., Feller, M., and Klajn, R. (2020). Chemical reactivity under nanoconfinement. Nat. Nanotechnol. *15*, 256–271.

2. Yoshizawa, M., Klosterman, J.K., and Fujita, M. (2009). Functional molecular flasks: new properties and reactions within discrete, self-assembled hosts. Angew. Chem. Int. Ed. *48*, 3418–3438.

3. Breiner, B., Clegg, J.K., and Nitschke, J.R. (2011). Reactivity modulation in container molecules. Chem. Sci. *2*, 51–56.

4. Chen, J., and Rebek, J. (2002). Selectivity in an encapsulated cycloaddition reaction. Org. Lett. *4*, 327–329.

5. Taguchi, T., Isozaki, K., and Miki, K. (2012). Enhanced catalytic activity of self-assembled-monolayer-capped gold nanoparticles. Adv. Mater. *24*, 6462–6467.

6. Zhao, H., Sen, S., Udayabhaskararao, T., Sawczyk, M., Kučanda, K., Manna, D., Kundu, P.K., Lee, J.-W., Král, P., and Klajn, R. (2016). Reversible trapping and reaction acceleration within dynamically self-assembling nanoflasks. Nat. Nanotechnol. *11*, 82–88.

7. Cram, D.J., Tanner, M.E., and Thomas, R. (1991). The taming of cyclobutadiene. Angew. Chem. Int. Ed. *30*, 1024–1027.

8. Mal, P., Breiner, B., Rissanen, K., and Nitschke, J.R. (2009). White phosphorus is air-stable within a self-assembled tetrahedral capsule. Science *324*, 1697–1699.

9. Yoshizawa, M., Kusukawa, T., Fujita, M., Sakamoto, S., and Yamaguchi, K. (2001). Cavity-directed synthesis of labile silanol oligomers within self-assembled coordination cages. J. Am. Chem. Soc. *123*, 10454–10459.

10. Hasegawa, S., Meichsner, S.L., Holstein, J.J., Baksi, A., Kasanmascheff, M., and Clever, G.H. (2021). Long-lived C_{60} radical anion stabilized inside an electron-deficient coordination cage. J. Am. Chem. Soc. *143*, 9718–9723.

11. Li, K., Zhang, L.-Y., Yan, C., Wei, S.-C., Pan, M., Zhang, L., and Su, C.-Y. (2014). Stepwise assembly of $Pd_6(RuL_3)_8$ nanoscale rhombododecahedral metal–organic cages via metalloligand strategy for guest trapping and protection. J. Am. Chem. Soc. *136*, 4456–4459.

12. Yamashina, M., Sei, Y., Akita, M., and Yoshizawa, M. (2014). Safe storage of radical initiators within a polyaromatic nanocapsule. Nat. Commun. *5*, 4662.

13. Mohanty, J., and Nau, W.M. (2005). Ultrastable rhodamine with cucurbituril. Angew. Chem. Int. Ed. *44*, 3750–3754.

14. Canton, M., Grommet, A.B., Pesce, L., Gemen, J., Li, S., Diskin-Posner, Y., Credi, A., Pavan, G.M., Andréasson, J., and Klajn, R. (2020). Improving fatigue resistance of dihydropyrene by encapsulation within a coordination cage. J. Am. Chem. Soc. *142*, 14557–14565.

15. Jing, X., He, C., Zhao, L., and Duan, C. (2019). Photochemical properties of host−guest supramolecular systems with structurally confined metal−organic capsules. Acc. Chem. Res. *52*, 100–109.

16. Dsouza, R.N., Pischel, U., and Nau, W.M. (2011). Fluorescent dyes and their supramolecular host/guest complexes with macrocycles in aqueous solution. Chem. Rev. *111*, 7941–7980.

17. Choi, I.-H., Bin Yoon, S., Huh, S., Kim, S.-J., and Kim, Y. (2018). Photophysical properties of cationic dyes captured in the mesoscale channels of micron-sized metal-organic framework crystals. Sci. Rep. *8*, 9838.

18. Takezawa, H., Akiba, S., Murase, T., and Fujita, M. (2015). Cavity-directed chromism of phthalein dyes. J. Am. Chem. Soc. *137*, 7043–7046.

19. Amdursky, N., Kundu, P.K., Ahrens, J., Huppert, D., and Klajn, R. (2016). Noncovalent interactions with proteins modify the physicochemical properties of a molecular switch. ChemPlusChem *81*, 44–48.

20. Ono, K., Klosterman, J.K., Yoshizawa, M., Sekiguchi, K., Tahara, T., and Fujita, M. (2009). ON/OFF red emission from azaporphine in a coordination cage in water. J. Am. Chem. Soc. *131*, 12526–12527.

21. Liang, G., Lam, J.W.Y., Qin, W., Li, J., Xie, N., and Tang, B.Z. (2014). Molecular luminogens based on restriction of intramolecular motions through host–guest inclusion for cell imaging. Chem. Commun. *50*, 1725–1727.

22. Dobashi, H., Catti, L., Tanaka, Y., Akita, M., and Yoshizawa, M. (2020). N-doping of polyaromatic capsules: small cavity modification leads to large change in host–guest interactions. Angew. Chem. Int. Ed. *59*, 11881–11885.

23. Gemen, J., Ahrens, J., Shimon, L.J.W., and Klajn, R. (2020). Modulating the optical properties of BODIPY dyes by noncovalent dimerization within a flexible coordination cage. J. Am. Chem. Soc. *142*, 17721–17729.

24. Barooah, N., Mohanty, J., and Bhasikuttan, A.C. (2015). Cucurbit[8]uril-templated H and J dimers of bichromophoric coumarin dyes: origin of contrasting emission. Chem. Commun. *51*, 13225–13228.

25. Saha, R., Devaraj, A., Bhattacharyya, S., Das, S., Zangrando, E., and Mukherjee, P.S. (2019). Unusual behavior of donor-acceptor Stenhouse adducts in confined space of a water-soluble Pd^{II}_8 molecular vessel. J. Am. Chem. Soc. *141*, 8638–8645.

26. Yamashina, M., Sartin, M.M., Sei, Y., Akita, M., Takeuchi, S., Tahara, T., and Yoshizawa, M. (2015). Preparation of highly fluorescent host–guest complexes with tunable color upon encapsulation. J. Am. Chem. Soc. *137*, 9266–9269.

27. Nishioka, Y., Yamaguchi, T., Kawano, M., and Fujita, M. (2008). Asymmetric [2 + 2] olefin cross photoaddition in a self-assembled host with remote chiral auxiliaries. J. Am. Chem. Soc. *130*, 8160–8161.

28. Kohyama, Y., Murase, T., and Fujita, M. (2014). Metal–organic proximity in a synthetic pocket. J. Am. Chem. Soc. *136*, 2966–2969.

29. Yang, Y., Ronson, T.K., Lu, Z., Zheng, J.Y., Vanthuyne, N., Martinez, A., and Nitschke, J.R. (2021). A curved host and second guest cooperatively inhibit the dynamic motion of corannulene. Nat. Commun. *12*, 4079.

30. Yazaki, K., Akita, M., Prusty, S., Chand, D.K., Kikuchi, T., Sato, H., and Yoshizawa, M. (2017). Polyaromatic molecular peanuts. Nat. Commun. *8*, 15914.

31. Craig, S.L., Lin, S., Chen, J., and Rebek, J. (2002). An NMR study of the rates of single-molecule exchange in a cylindrical host capsule. J. Am. Chem. Soc. *124*, 8780–8781.

32. Wang, X., and Houk, K.N. (1999). Gating and entropy in guest exchange by Rebek's sportsballs. Theoretical studies of one-door, side-door, and back-door gating. Org. Lett. *1*, 591–594.

33. Davis, A.V., and Raymond, K.N. (2005). The big squeeze: guest exchange in an M_4L_6 supramolecular host. J. Am. Chem. Soc. *127*, 7912–7919.

34. Escobar, L., Escudero-Adán, E.C., and Ballester, P. (2019). Guest exchange mechanisms in mono-metallic Pd^{II}/Pt^{II}-cages based on a tetra-pyridyl calix[4]pyrrole ligand. Angew. Chem. Int. Ed. *58*, 16105–16109.

35. Meng, W., Breiner, B., Rissanen, K., Thoburn, J.D., Clegg, J.K., and Nitschke, J.R. (2011). A self-assembled M_8L_6 cubic cage that selectively encapsulates large aromatic guests. Angew. Chem. Int. Ed. *50*, 3479–3483.

36. Vysotsky, M.O., Thondorf, I., and Böhmer, V. (2000). Self-assembled hydrogen-bonded dimeric capsules with high kinetic stability. Angew. Chem. Int. Ed. *39*, 1264–1267.

37. Sakata, Y., Okada, M., and Akine, S. (2021). Guest recognition control accompanied by stepwise gate closing and opening of a macrocyclic metallohost. Chem. Eur. J. *27*, 2284–2288.

38. Samanta, D., Mukherjee, S., Patil, Y.P., and Mukherjee, P.S. (2012). Self-assembled Pd_6 open cage with triimidazole walls and the use of its confined nanospace for catalytic Knoevenagel- and Diels–Alder reactions in aqueous medium. Chem. Eur. J. *18*, 12322–12329.

39. Samanta, D., Galaktionova, D., Gemen, J., Shimon, L.J.W., Diskin-Posner, Y., Avram, L., Král, P., and Klajn, R. (2018). Reversible chromism of spiropyran in the cavity of a flexible coordination cage. Nat. Commun. *9*, 641.

40. Hanopolskyi, A.I., De, S., Białek, M.J., Diskin-Posner, Y., Avram, L., Feller, M., and Klajn, R. (2019). Reversible switching of arylazopyrazole within a metal-organic cage. Beilstein J. Org. Chem. *15*, 2398–2407.

41. Yanshyna, O., Białek, M.J., Chashchikhin, O.V., and Klajn, R. (2022). Encapsulation within a coordination cage modulates the reactivity of redox-active dyes. Commun. Chem. *5*, 44.

42. Yanshyna, O., Avram, L., Shimon, L.J.W., and Klajn, R. (2022). Coexistence of 1:1 and 2:1 inclusion complexes of indigo carmine. Chem. Commun. *58*, 3461–3464.

43. Pesce, L., Perego, C., Grommet, A.B., Klajn, R., and Pavan, G.M. (2020). Molecular factors controlling the isomerization of azobenzenes in the cavity of a flexible coordination cage. J. Am. Chem. Soc. *142*, 9792–9802.

44. Samanta, D., Gemen, J., Chu, Z., Diskin-Posner, Y., Shimon, L.J.W., and Klajn, R. (2018). Reversible photoswitching of encapsulated azobenzenes in water. Proc. Natl. Acad. Sci. USA *115*, 9379–9384.

45. Zhou, J., Zhang, Y., Yu, G., Crawley, M.R., Fulong, C.R.P., Friedman, A.E., Sengupta, S., Sun, J., Li, Q., Huang, F., et al. (2018). Highly emissive self-assembled BODIPY-platinum supramolecular triangles. J. Am. Chem. Soc. *140*, 7730–7736.

46. Kumar, P.P.P., Rahman, A., Goswami, T., Ghosh, H.N., and Neelakandan, P.P. (2021). Fine-tuning plasmon-molecule interactions in gold-BODIPY nanocomposites: the role of chemical structure and noncovalent interactions. ChemPlusChem *86*, 87–94.

47. Matarranz, B., and Fernández, G. (2021). BODIPY dyes: versatile building blocks to construct multiple types of self-assembled structures. Chem. Physiol. Rev. *2*, 041304.

48. Seo, C., Kim, M., Lee, J., Lee, C.Y., and Kim, J. (2020). Spectroscopic evidence of energy transfer in BODIPY-incorporated nanoporphyrinic metal-organic frameworks. Nanomaterials *10*, 1925.

49. Würthner, F., Saha-Möller, C.R., Fimmel, B., Ogi, S., Leowanawat, P., and Schmidt, D. (2016). Perylene bisimide dye assemblies as archetype functional supramolecular materials. Chem. Rev. *116*, 962–1052.

50. Regeni, I., Chen, B., Frank, M., Baksi, A., Holstein, J.J., and Clever, G.H. (2021). Coal-tar dye-based coordination cages and helicates. Angew. Chem. Int. Ed. *60*, 5673–5678.

51. Baram, J., Weissman, H., Tidhar, Y., Pinkas, I., and Rybtchinski, B. (2014). Hydrophobic self-assembly affords robust noncovalent polymer isomers. Angew. Chem. Int. Ed. *53*, 4123–4126.

52. Zhou, Z., Hauke, C.E., Song, B., Li, X., Stang, P.J., and Cook, T.R. (2019). Understanding the effects of coordination and self-assembly on an emissive phenothiazine. J. Am. Chem. Soc. *141*, 3717–3722.

53. Hardy, M., and Lützen, A. (2020). Better together: functional heterobimetallic macrocyclic and cage-like assemblies. Chem. Eur. J. *26*, 13332–13346.

54. Karthikeyan, S., and Ramamurthy, V. (2005). Self-assembled coordination cage as a reaction vessel: triplet sensitized [2+2] photodimerization of acenaphthylene, and [4+4] photodimerization of 9-anthraldehyde. Tetrahedron Lett. *46*, 4495–4498.

55. Carvalho, C.P., Domínguez, Z., Da Silva, J.P., and Pischel, U. (2015). A supramolecular keypad lock. Chem. Commun. *51*, 2698–2701.

56. Biedermann, F., Ross, I., and Scherman, O.A. (2014). Host-guest accelerated photodimerisation of anthracene-labeled macromolecules in water. Polym. Chem. *5*, 5375–5382.

57. Hu, X., Liu, F., Zhang, X., Zhao, Z., and Liu, S. (2020). Expected and unexpected photoreactions of 9-(10-)substituted anthracene derivatives in cucurbit[*n*]uril hosts. Chem. Sci. *11*, 4779–4785.

58. Zhou, W., Chen, Y., Yu, Q., Li, P., Chen, X., and Liu, Y. (2019). Photo-responsive cyclodextrin/anthracene/Eu^{3+} supramolecular assembly for a tunable photochromic multicolor cell label and fluorescent ink. Chem. Sci. *10*, 3346–3352.

59. Choi, S., Bouffard, J., and Kim, Y. (2014). Aggregation-induced emission enhancement of a *meso*-trifluoromethyl BODIPY *via* J-aggregation. Chem. Sci. *5*, 751–755.

60. Fritzsche. (1867). Ueber die festen Kohlenwasserstoffe des Steinkohlentheers. J. Prakt. Chem. *101*, 333–343.

61. Bouas-Laurent, H., Desvergne, J., Castellan, A., and Lapouyade, R. (2000). Photodimerization of anthracenes in fluid solution: structural aspects. Chem. Soc. Rev. *29*, 43–55.

62. Tron, A., Jacquot de Rouville, H.P., Ducrot, A., Tucker, J.H.R., Baroncini, M., Credi, A., et al. (2015). Photodriven [2]rotaxane–[2]catenane interconversion. Chem. Commun. *51*, 2810–2813.

63. Cohen, M.D., Ludmer, Z., Thomas, J.M., and Williams, J.O. (1971). The role of structural imperfections in the photodimerization of 9-cyanoanthracene. Proc. R. Soc. Lond. A *324*, 459–468.

64. Tu, M., Reinsch, H., Rodríguez-Hermida, S., Verbeke, R., Stassin, T., Egger, W., et al. (2019). Reversible optical writing and data storage in an anthracene-loaded metal–organic

CellPress
OPEN ACCESS

framework. Angew. Chem. Int. Ed. *58*, 2423–2427.

65. Bhola, R., Payamyar, P., Murray, D.J., Kumar, B., Teator, A.J., Schmidt, M.U., Hammer, S.M., Saha, A., Sakamoto, J., Schlüter, A.D., and King, B.T. (2013). A two-dimensional polymer from the anthracene dimer and triptycene motifs. J. Am. Chem. Soc. *135*, 14134–14141.

66. Kim, M., Hohman, J.N., Cao, Y., Houk, K.N., Ma, H., Jen, A.K.-Y., and Weiss, P.S. (2011). Creating favorable geometries for directing organic photoreactions in alkanethiolate monolayers. Science *331*, 1312–1315.

67. Zdobinsky, T., Maiti, P.S., and Klajn, R. (2014). Support curvature and conformational freedom control chemical reactivity of immobilized species. J. Am. Chem. Soc. *136*, 2711–2714.

68. Chen, X.-Y., Chen, H., Đorđević, L., Guo, Q.-H., Wu, H., Wang, Y., Zhang, L., Jiao, Y., Cai, K., Chen, H., et al. (2021). Selective photodimerization in a cyclodextrin metal–organic framework. J. Am. Chem. Soc. *143*, 9129–9139.

69. Logan, S.R. (1997). Does a photochemical reaction have a reaction order? J. Chem. Educ. *74*, 1303.

70. Fulara, J., and Latowski, T. (1981). Photochemical reactions of bromoanthracenes with N,N-dimethylamine in solution. Z. Naturforsch. B *36*, 846–851.

71. Grommet, A.B., Lee, L.M., and Klajn, R. (2020). Molecular photoswitching in confined spaces. Acc. Chem. Res. *53*, 2600–2610.

72. Cohen, M.D., and Schmidt, G.M.J. (1964). Topochemistry. Part I. A survey (J. Chem. Soc.), pp. 1996–2000.

73. Schmidt, G.M.J. (1971). Photodimerization in the solid state. Pure Appl. Chem. *27*, 647–678.

Chem, Volume *8*

Supplemental information

Ternary host-guest complexes
with rapid exchange kinetics
and photoswitchable fluorescence

Julius Gemen, Michał J. Białek, Miri Kazes, Linda J.W. Shimon, Moran Feller, Sergey N. Semenov, Yael Diskin-Posner, Dan Oron, and Rafal Klajn

Table of contents

I. Supplemental experimental procedures 2

1. Materials and methods 2
2. Synthesis and characterization of cage **C** and guests **a1**–**a4** and **b1**–**b4** 3
3. Following the frmation of homodimeric complexes 17
4. NMR characterization of BODIPY homodimers 18
5. NMR characterization of homodimers (**a1**)$_2$⊂**C**, (**a2**)$_2$⊂**C**, (**a3**)$_2$⊂**C**, and (**a4**)$_2$⊂**C** 19
6. NMR characterization of heterodimers (**a1**·**b1**)⊂**C**, (**a2**·**b1**)⊂**C**, (**a1**·**b2**)⊂**C**, and (**a4**·**b4**)⊂**C** 63
7. X-ray data collection and structure refinement 77
8. DFT calculations of the (**a2**·**b1**)⊂**C** heterodimer 84
9. Steady-state optical properties of **a1**, **a2**, **a3**, and **a4** and their inclusion complexes 85
10. Formation of heterodimeric inclusion complexes and their steady-state optical properties 88
11. Kinetics of heterodimer formation 93
12. Time-resolved fluorescence spectroscopy of heterodimeric inclusion complexes 97
13. Photodimerization of encapsulated **a1**–**a4** 98
14. Photoresponsiveness of heterodimeric inclusion complexes 103
15. Preparation of photoresponsive gels based on heterodimeric inclusion complexes 105

II. Supplemental references 105

I. Supplemental experimental procedures

1. Materials and methods

All commercial chemicals were used as received unless stated otherwise. NMR spectra were recorded on a Bruker Avance III 400 MHz spectrometer, a Bruker Avance III HD 500 MHz spectrometer, or a Bruker Avance III 600 MHz spectrometer. Chemical shifts (δ) are given in ppm relative to residual proton solvent resonances (4.79 ppm for D_2O and 7.26 ppm for $CDCl_3$). For spectra recorded in D_2O at higher temperatures, the resonance of residual solvent was set to 4.55 ppm for 320 K, 4.45 for 330 K, and 4.35 ppm for 340 K, according to Ref. 1. ^{1}H DOSY measurements were performed on a Bruker Avance III 500 MHz spectrometer or a Bruker Avance III 600 MHz spectrometer at room temperature. Solution-state UV-vis absorption spectra were recorded with an Agilent Cary 60 spectrophotometer. Emission and excitation spectra were recorded with a Shimadzu spectrofluorophotometer RF-5301 PC. Fluorescence quantum yields were determined on a Quantaurus-QY Absolute PL quantum yield spectrometer. Mass spectra were recorded on a Waters Xevo G2-XS quadrupole time-of-flight high-resolution mass spectrometer with an electrospray ionization ion source, operated under MassLynx software. For details on the X-ray data collection and refinement, see Section 7. For details on density functional theory (DFT) calculations, see Section 8. For details on time-resolved fluorescence spectroscopy, see Section 12.

2. Synthesis and characterization of cage **C** and guests **a1**–**a4** and **b1**–**b4**

Cage **C** was synthesized according to a previously reported literature procedure.[2]

^{1}H NMR (500 MHz, D_2O, 298 K): δ = 9.12 (s, 8H, $\mathbf{C_4}$), 8.83 (s, 4H, $\mathbf{C_1}$), 7.75 (s, 4H, $\mathbf{C_8}$), 7.73 (s, 4H, $\mathbf{C_3}$), 7.71 (s, 8H, $\mathbf{C_7}$), 7.66 (s, 8H, $\mathbf{C_6}$), 7.56 (s, 12H, $\mathbf{C_{2+5}}$), 3.16–3.08 (s, 24H, $\mathbf{C_9}$), 2.82–2.69 (m, 72H, $\mathbf{C_{10}}$).

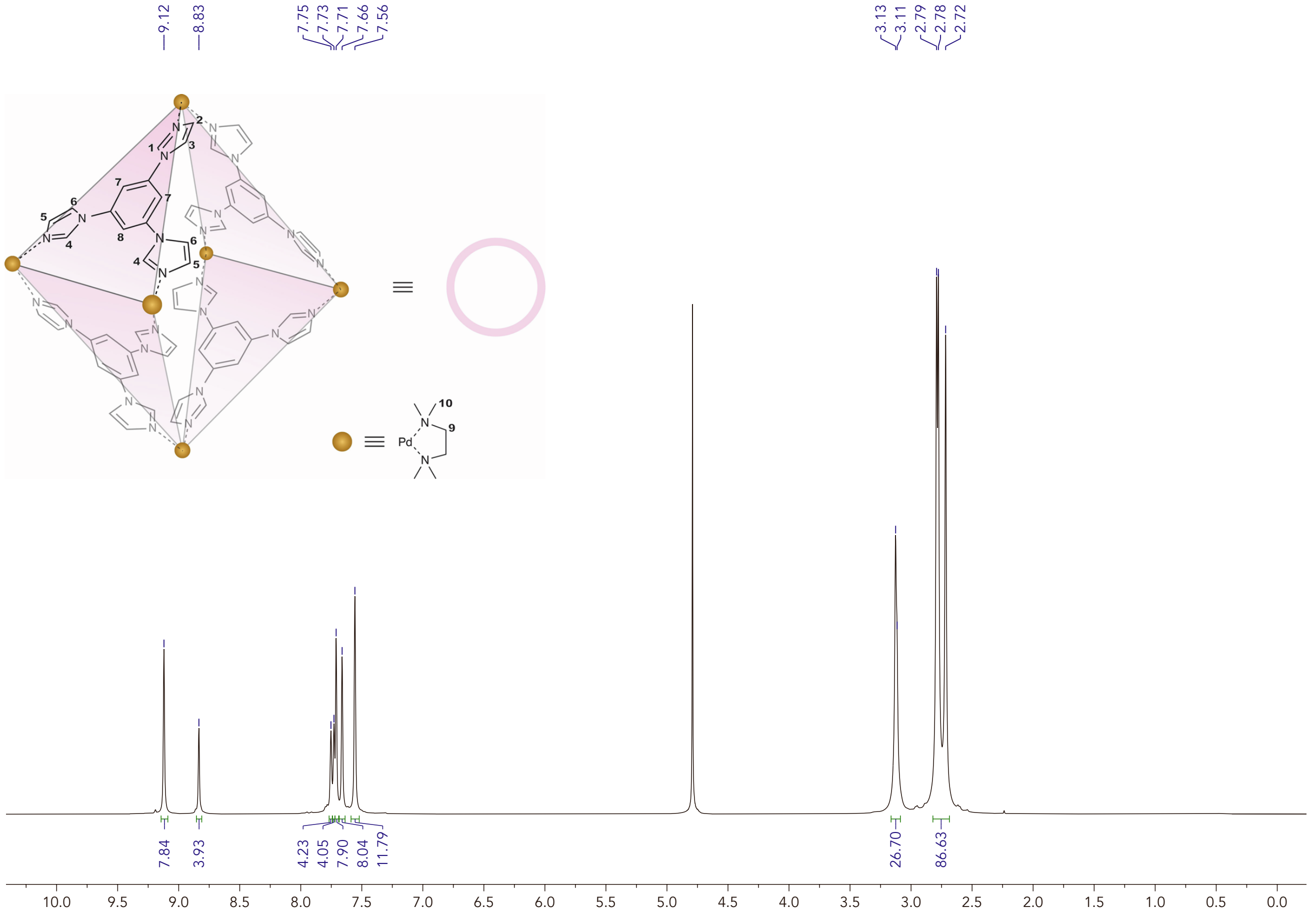

Figure S1. ^{1}H NMR spectrum of cage **C** (500 MHz, D_2O, 298 K).

Anthracenes **a1**–**a3** and pyrene (**a4**) are commercially available compounds. Their NMR characterization is provided below to facilitate the characterization of their inclusion complexes (Sections 5 and 6).

a1:

^{1}H NMR (500 MHz, $CDCl_3$, 298 K): δ = 8.35 (s, 1H, **a1**$_5$), 8.30 (d, 2H, **a1**$_1$), 8.01 (d, 2H, **a1**$_4$), 7.52 (t, 2H, **a1**$_2$), 7.48 (t, 2H, **a1**$_3$), 3.11 (s, 3H, **a1**$_{CH_3}$).

^{13}C NMR (100 MHz, $CDCl_3$, 298 K): δ = 131.6 (**a1**$_8$), 130.2 (**a1**$_7$), 130.2 (**a1**$_6$), 129.2 (**a1**$_4$), 125.4 (**a1**$_5$), 125.3 (**a1**$_3$), 124.9 (**a1**$_2$), 124.8 (**a1**$_1$), 14.0 (**a1**$_{CH_3}$).

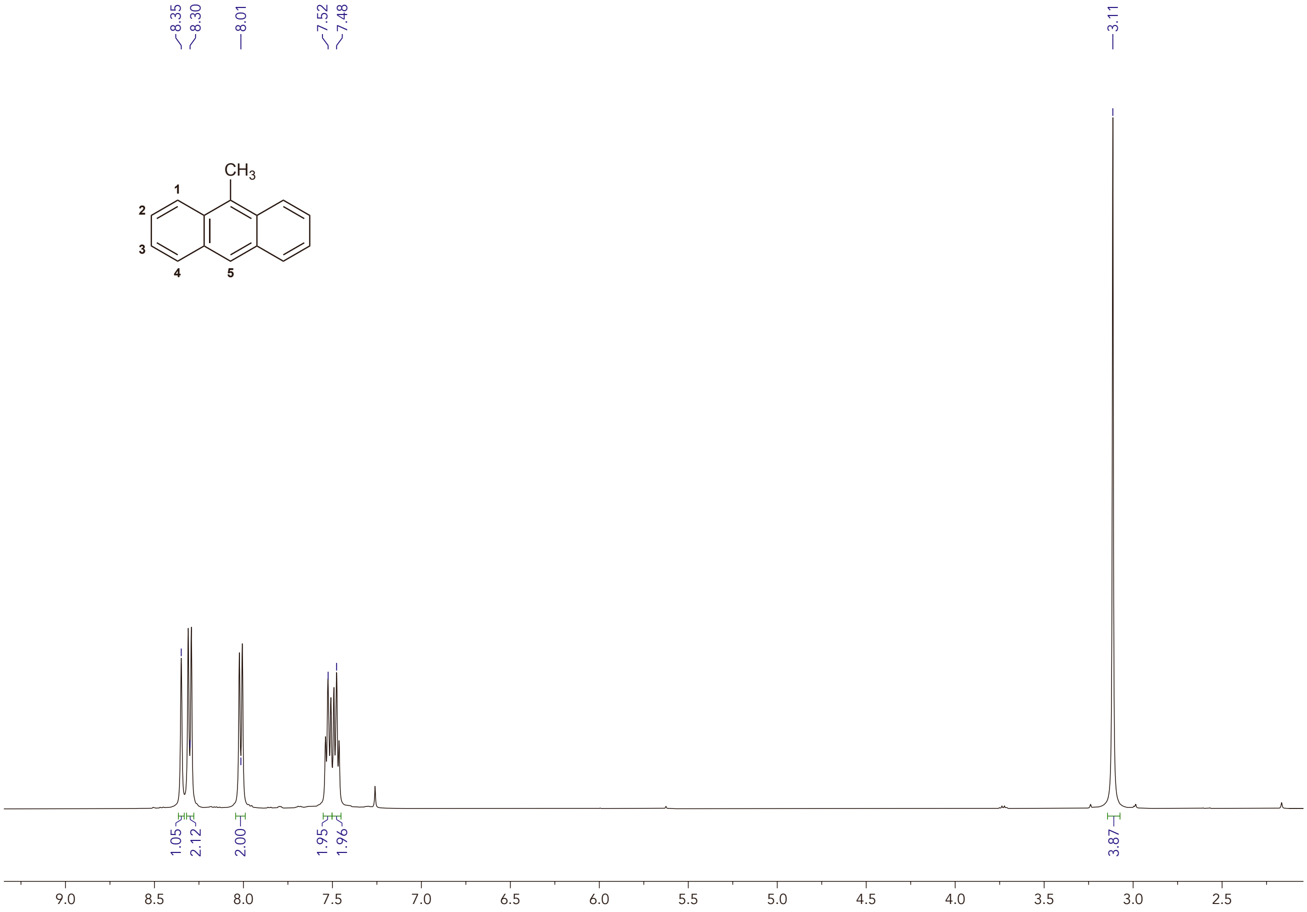

Figure S2. ^{1}H NMR spectrum of **a1** (500 MHz, $CDCl_3$, 298 K).

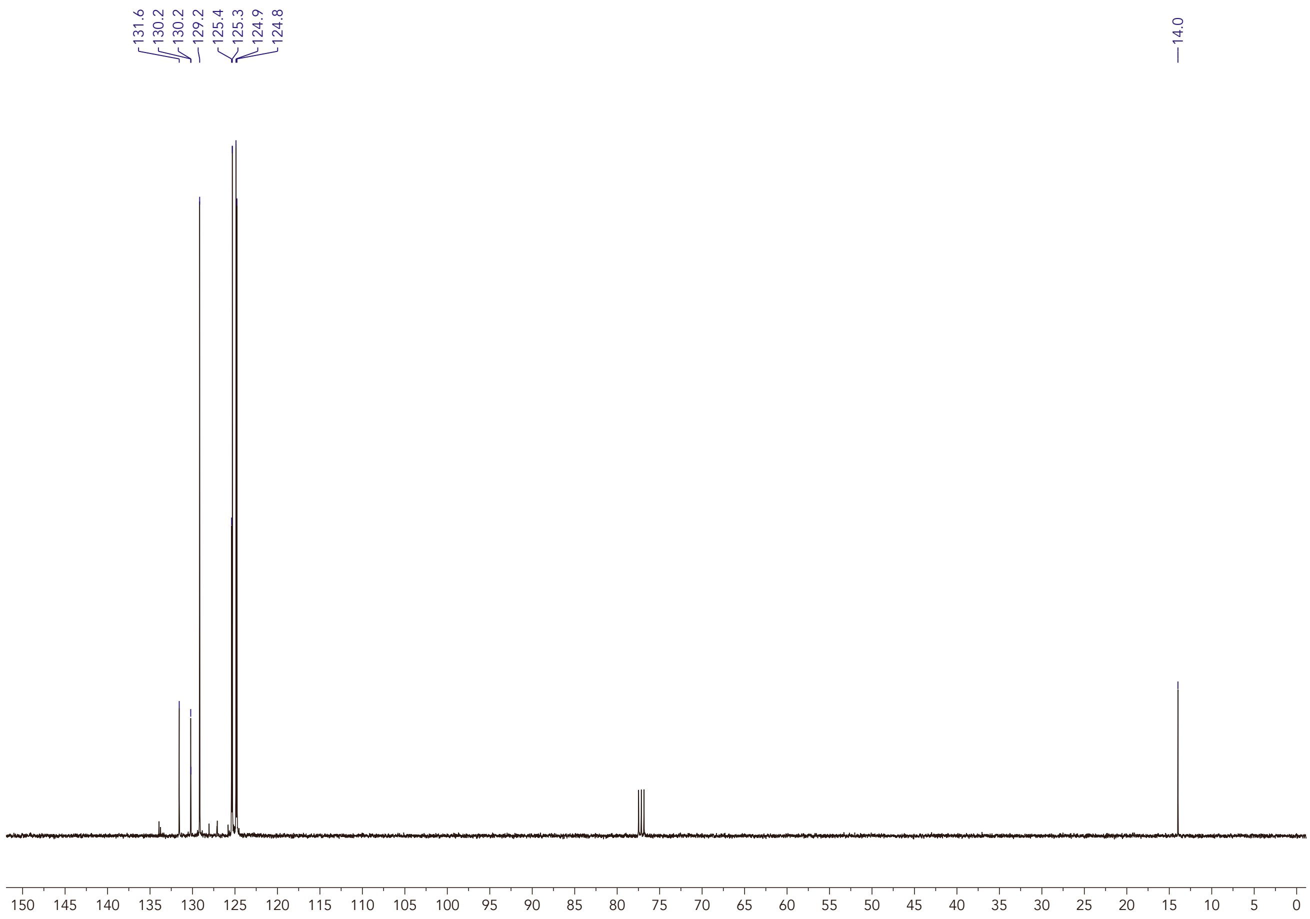

Figure S3. ^{13}C NMR spectrum of **a1** (100 MHz, $CDCl_3$, 298 K).

a2:

^{1}H NMR (500 MHz, $CDCl_3$, 298 K): δ = 8.53 (d, 2H, **a2$_1$**), 8.43 (s, 1H, **a2$_5$**), 7.99 (d, 2H, **a2$_4$**), 7.61 (t, 2H, **a2$_3$**), 7.51 (t, 2H, **a2$_2$**).

^{13}C NMR (100 MHz, $CDCl_3$, 298 K): δ = 132.2 (**a2$_8$**), 130.6 (**a2$_7$**), 128.7 (**a2$_4$**), 127.7 (**a2$_1$**), 127.3 (**a2$_2$**), 127.2 (**a2$_5$**), 125.7 (**a2$_3$**), 122.4 (**a2$_6$**).

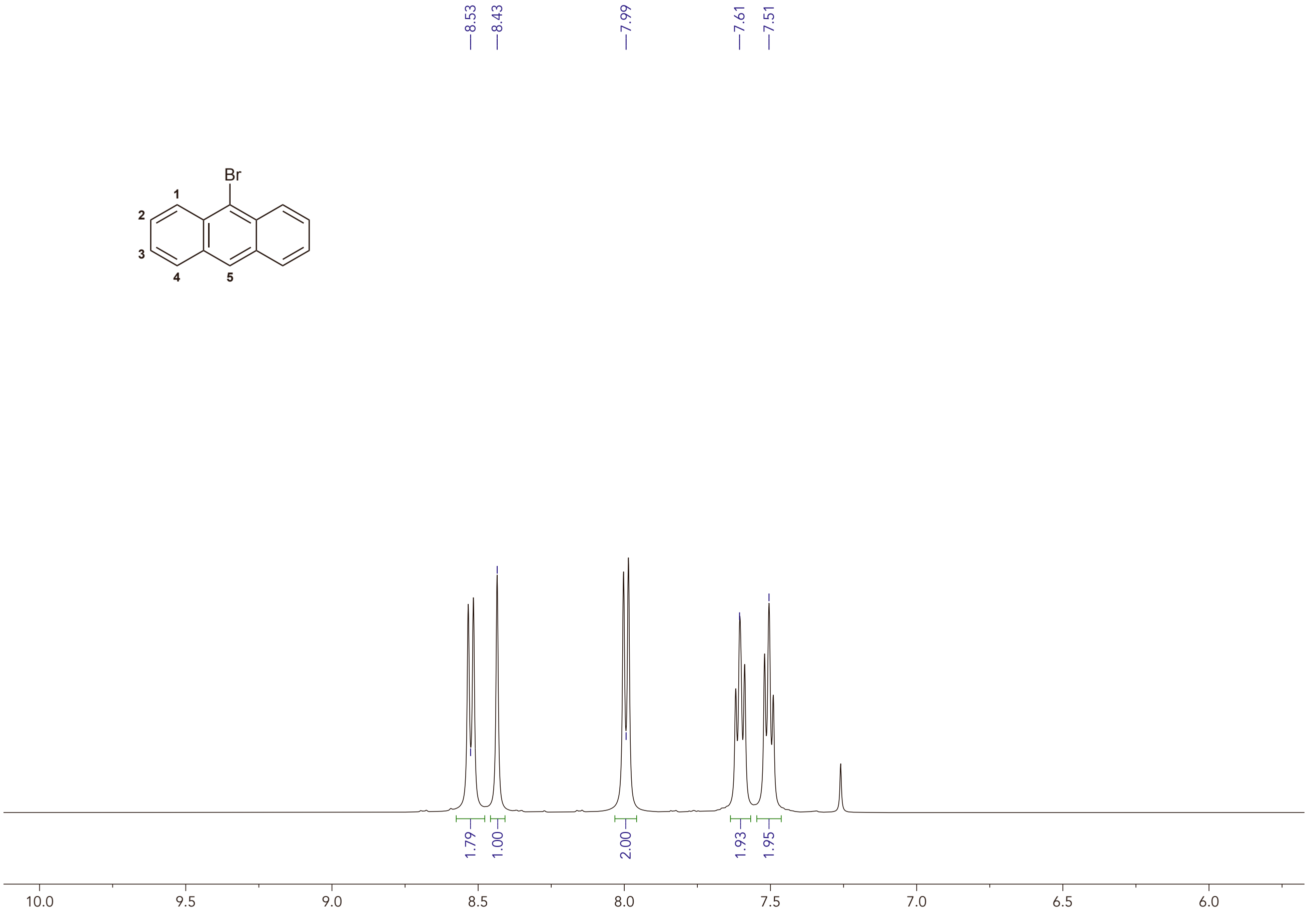

Figure S4. ^{1}H NMR spectrum of **a2** (500 MHz, $CDCl_3$, 298 K).

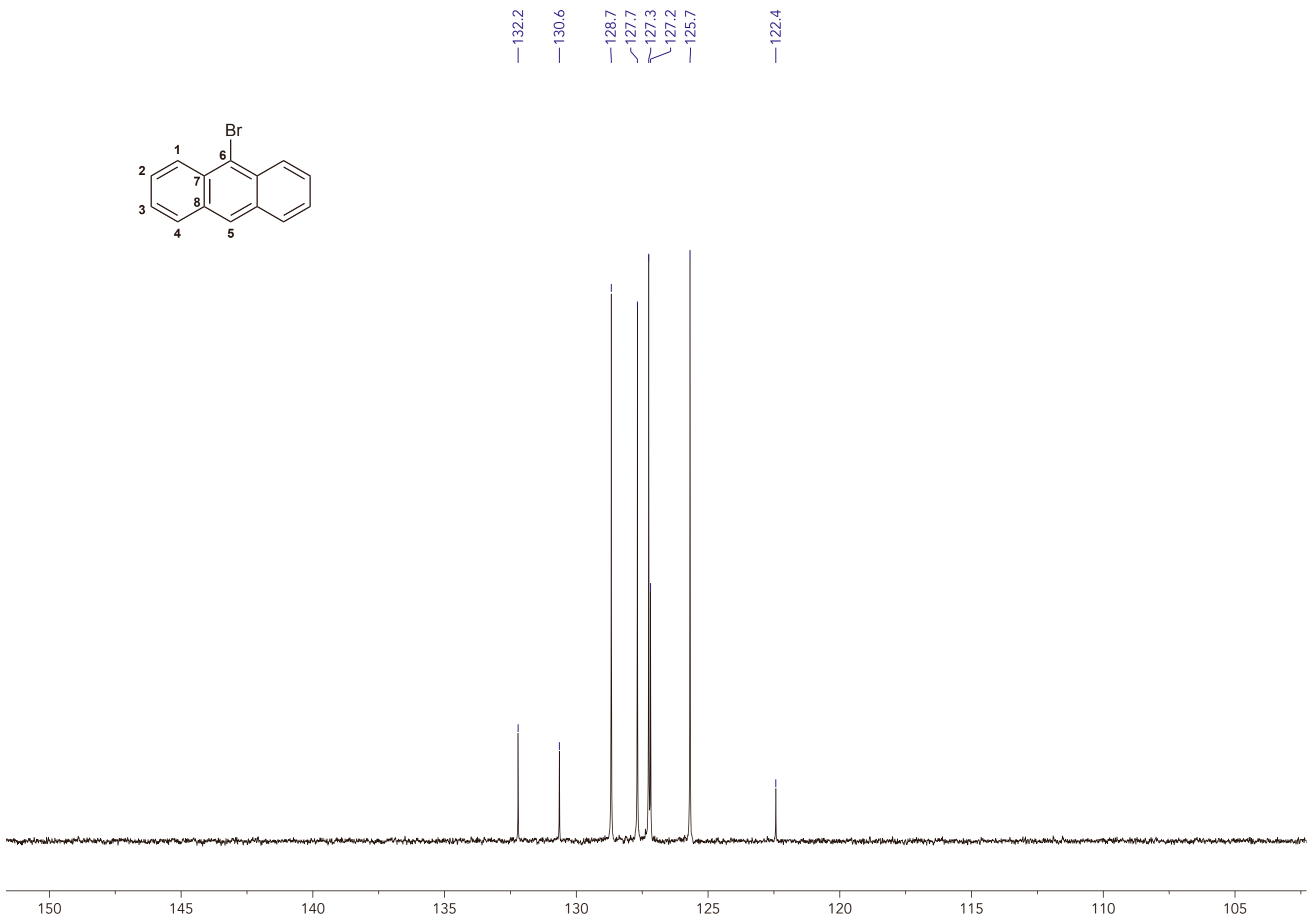

Figure S5. ^{13}C NMR spectrum of **a2** (100 MHz, $CDCl_3$, 298 K).

a3:

^{1}H NMR (400 MHz, $CDCl_3$, 298 K): δ = 8.45 (s, 1H, $\mathbf{a3}_{5}$), 8.39 (d, 2H, $\mathbf{a3}_{1}$), 8.02 (d, 2H, $\mathbf{a3}_{4}$), 7.56 (t, 2H, $\mathbf{a3}_{2}$), 7.48 (t, 2H, $\mathbf{a3}_{3}$), 5.64 (s, 2H, $\mathbf{a3}_{CH_2}$).

^{13}C NMR (100 MHz, $CDCl_3$, 298 K): δ = 131.7 ($\mathbf{a3}_{6}$), 131.1 ($\mathbf{a3}_{7}$), 130.4 ($\mathbf{a3}_{8}$), 129.3 ($\mathbf{a3}_{4}$), 128.5 ($\mathbf{a3}_{5}$), 126.6 ($\mathbf{a3}_{3}$), 125.2 ($\mathbf{a3}_{2}$), 124.0 ($\mathbf{a3}_{1}$), 57.5 ($\mathbf{a3}_{CH_2}$).

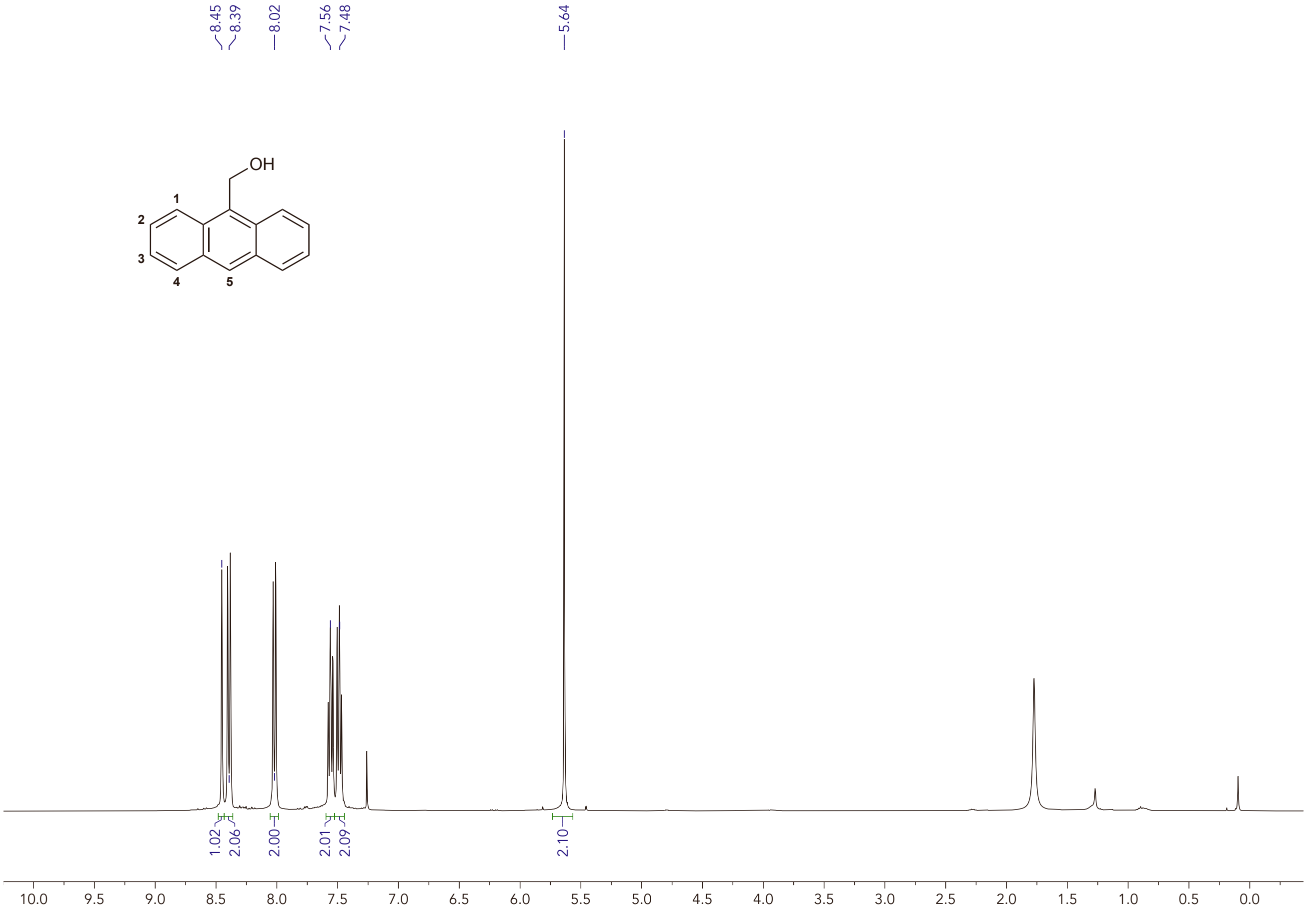

Figure S6. ^{1}H NMR spectrum of **a3** (400 MHz, $CDCl_3$, 298 K).

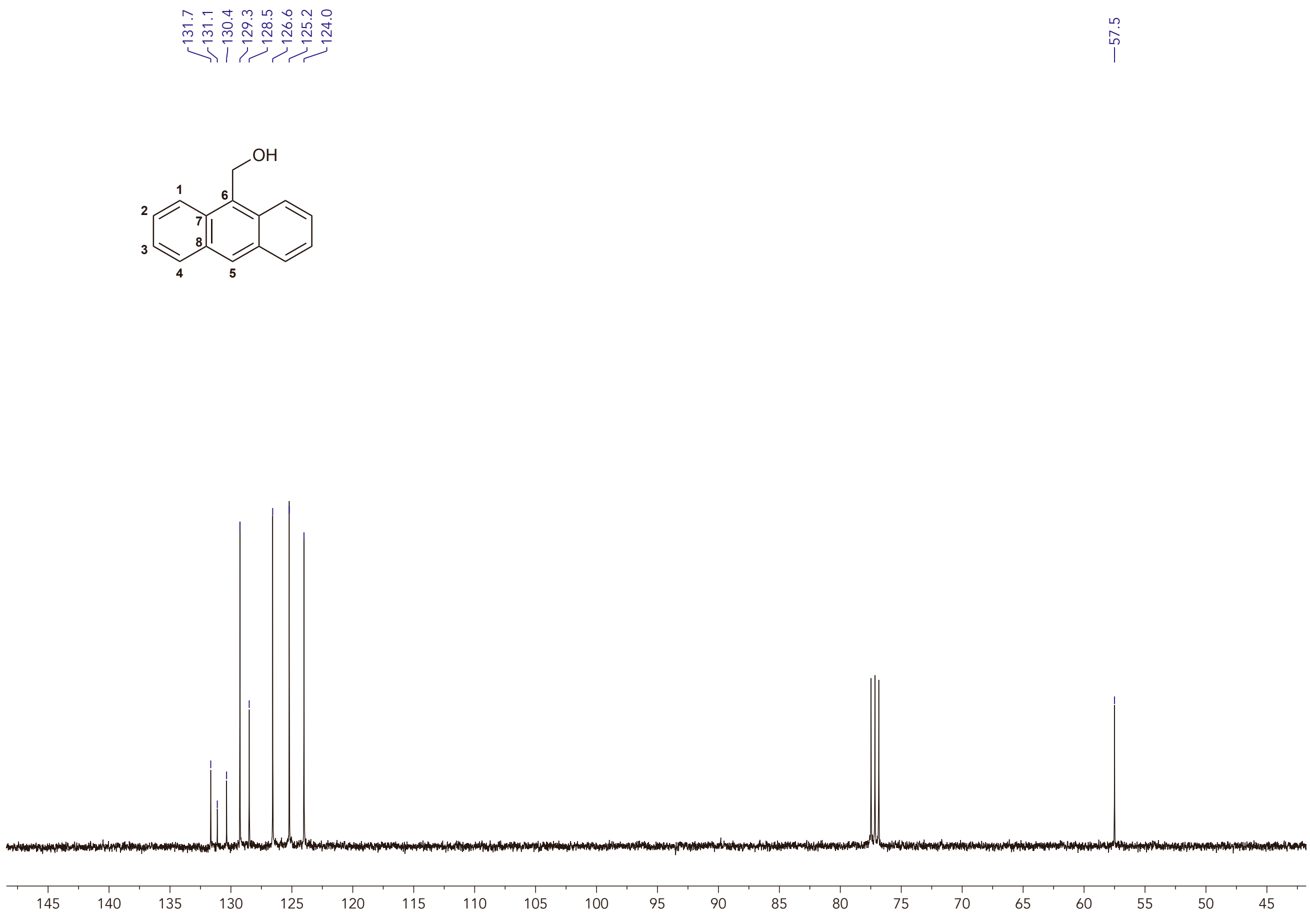

Figure S7. ^{13}C NMR spectrum of **a3** (100 MHz, $CDCl_3$, 298 K).

a4:

^{1}H NMR (500 MHz, $CDCl_3$, 298 K): δ = 8.20 (d, 4H, $\mathbf{a4_1}$), 8.09 (s, 4H, $\mathbf{a4_3}$), 8.02 (t, 2H, $\mathbf{a4_2}$).

^{13}C NMR (100 MHz, $CDCl_3$, 298 K): δ = 131.2 ($\mathbf{a4_4}$), 127.4 ($\mathbf{a4_3}$), 125.9 ($\mathbf{a4_2}$), 125.0 ($\mathbf{a4_1}$), 124.7 ($\mathbf{a4_5}$).

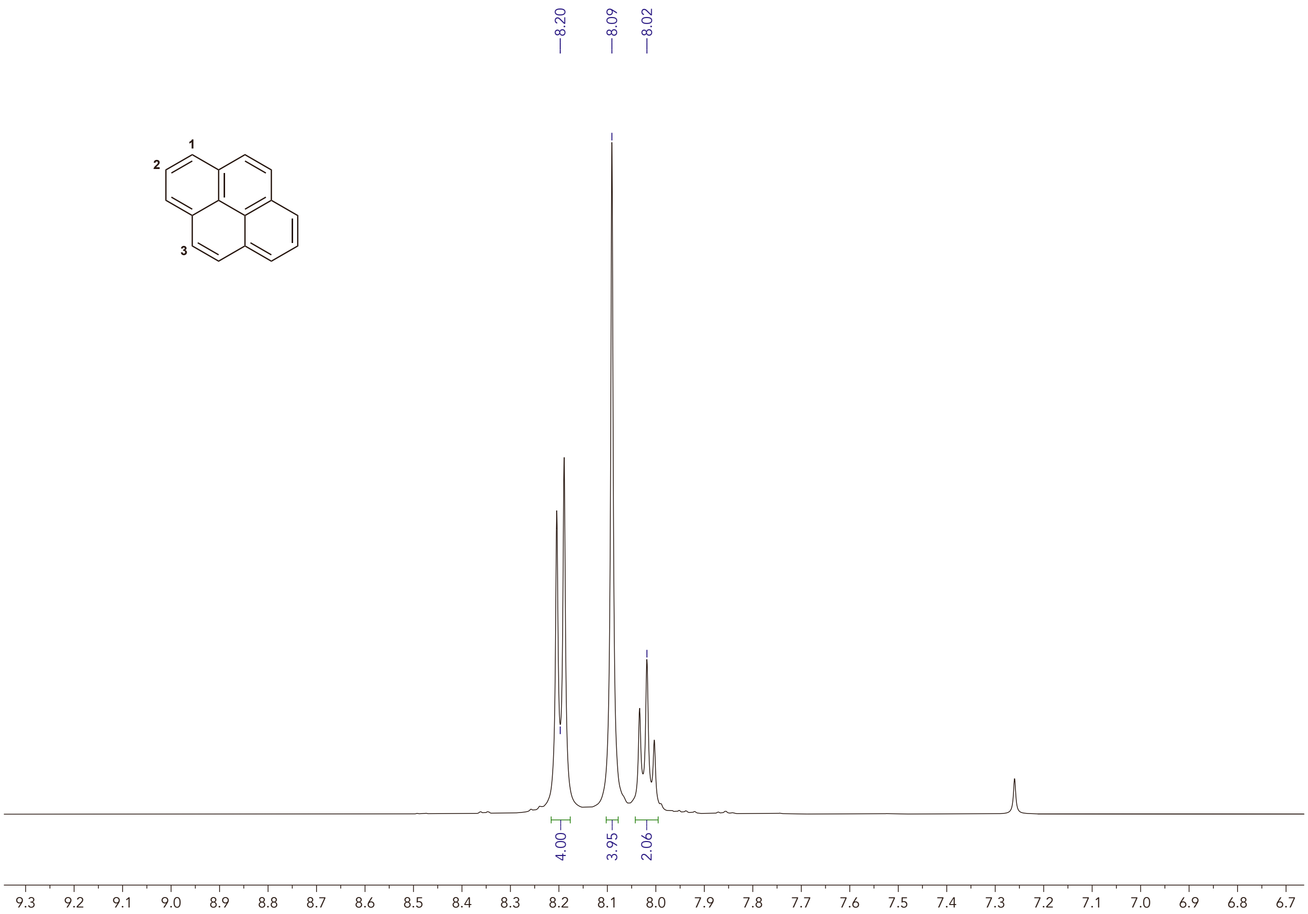

Figure S8. ^{1}H NMR spectrum of **a4** (500 MHz, $CDCl_3$, 298 K).

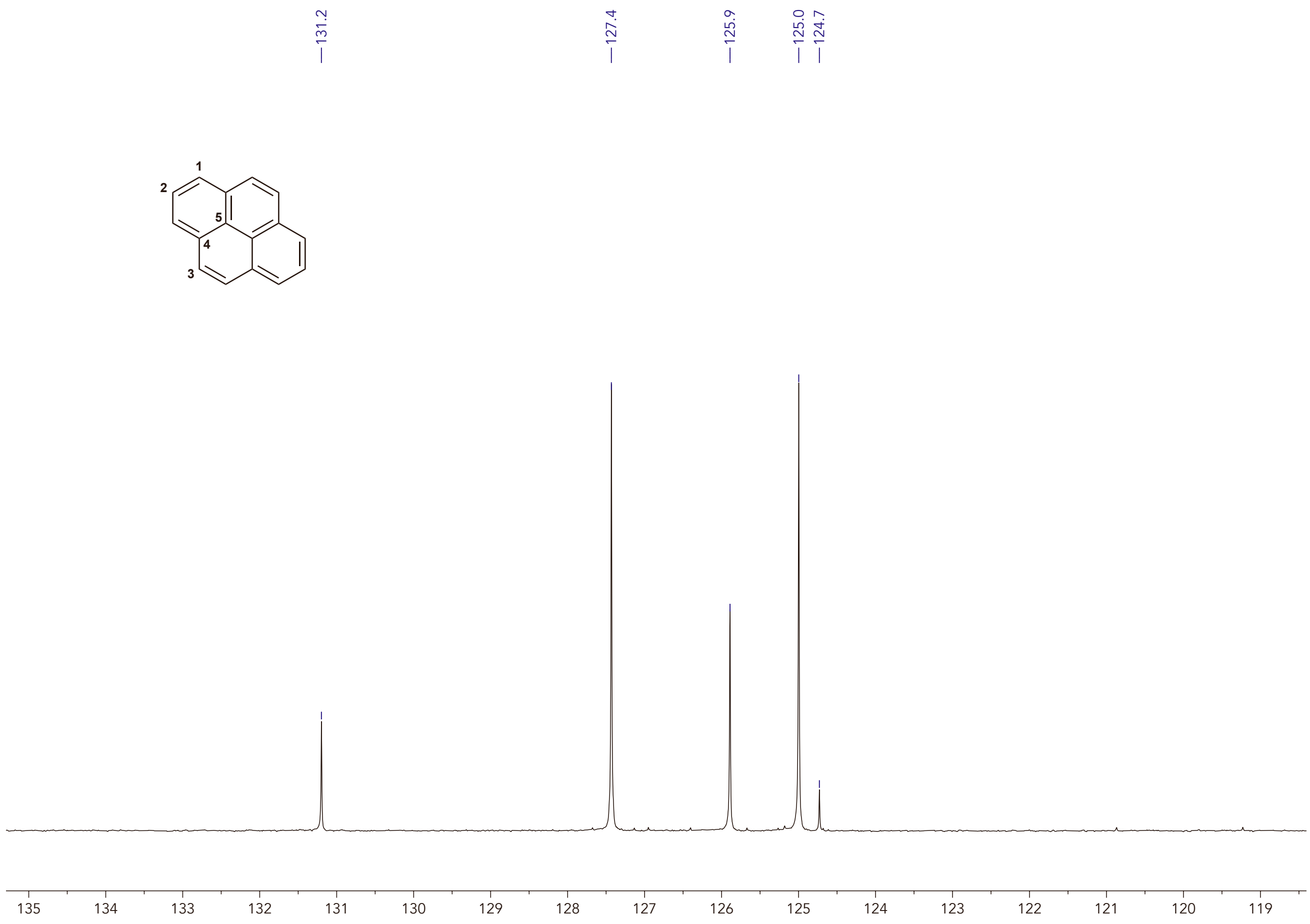

Figure S9. ^{13}C NMR spectrum of **a4** (100 MHz, $CDCl_3$, 298 K).

BODIPYs **b1**, **b2**, and **b4** were synthesized as reported previously.[2] BODIPY **b3** was synthesized analogously to a previous synthesis of **b1**,[3] but using 2,3,4-trimethyl-1*H*-pyrrole as the precursor.

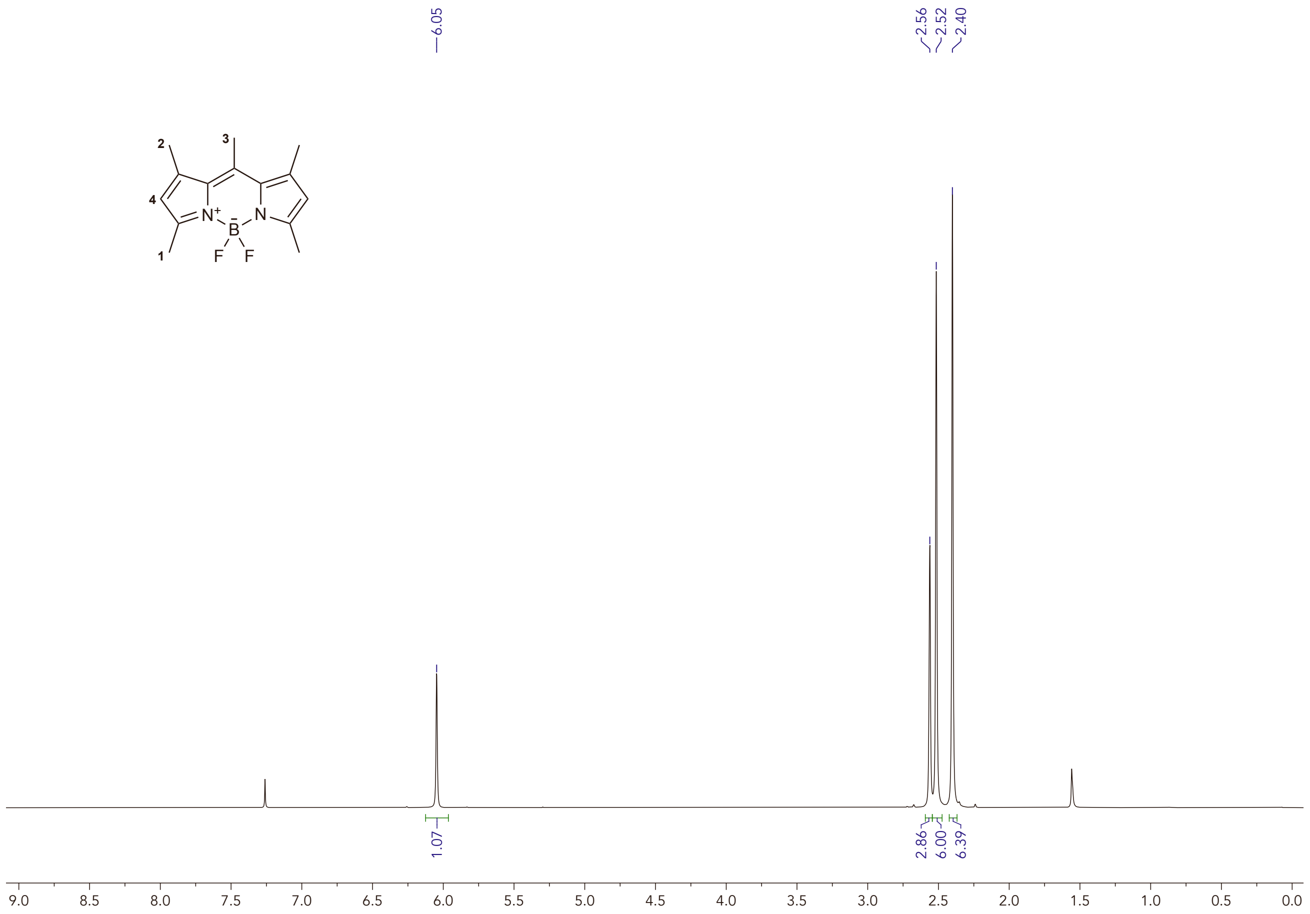

Figure S10. ^{1}H NMR spectrum of **b1** (400 MHz, $CDCl_3$, 298 K).

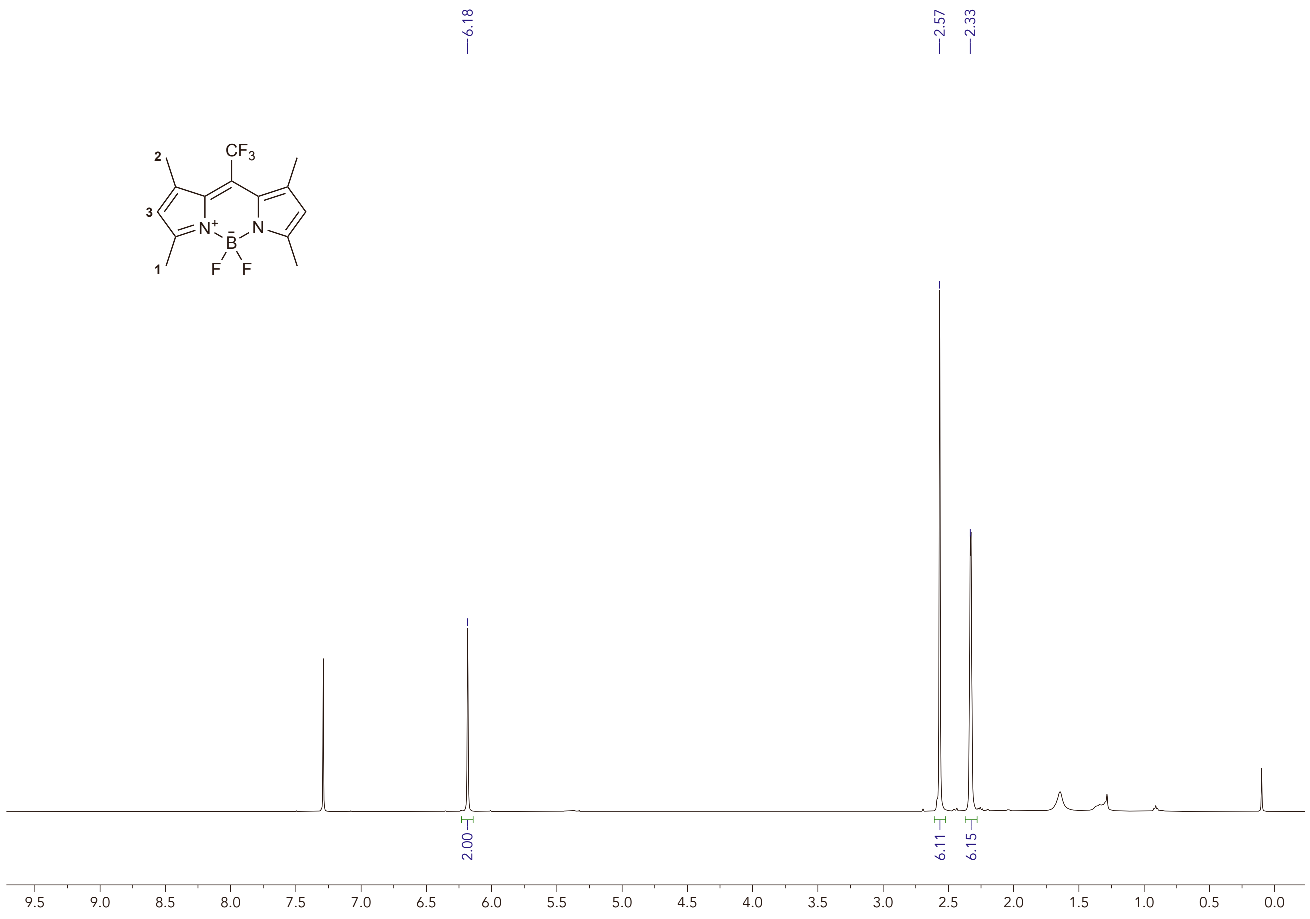

Figure S11. ^{1}H NMR spectrum of **b2** (400 MHz, $CDCl_3$, 298 K).

b3:

^{1}H NMR (400 MHz, $CDCl_3$, 298 K): δ = 2.59 (s, 3H, **b3**$_3$), 2.48 (s, 6H, **b3**$_1$), 2.31 (s, 6H, **b3**$_2$), 1.94 (s, 6H, **b3**$_4$).

^{13}C NMR (100 MHz, $CDCl_3$, 298 K): δ = 152.2 (**b3**$_5$), 139.7 (**b3**$_9$), 137.0 (**b3**$_7$), 131.7 (**b3**$_8$), 126.0 (**b3**$_6$), 17.0 (**b3**$_3$), 14.7 (**b3**$_2$), 12.7 (**b3**$_1$), 9.2 (**b3**$_4$).

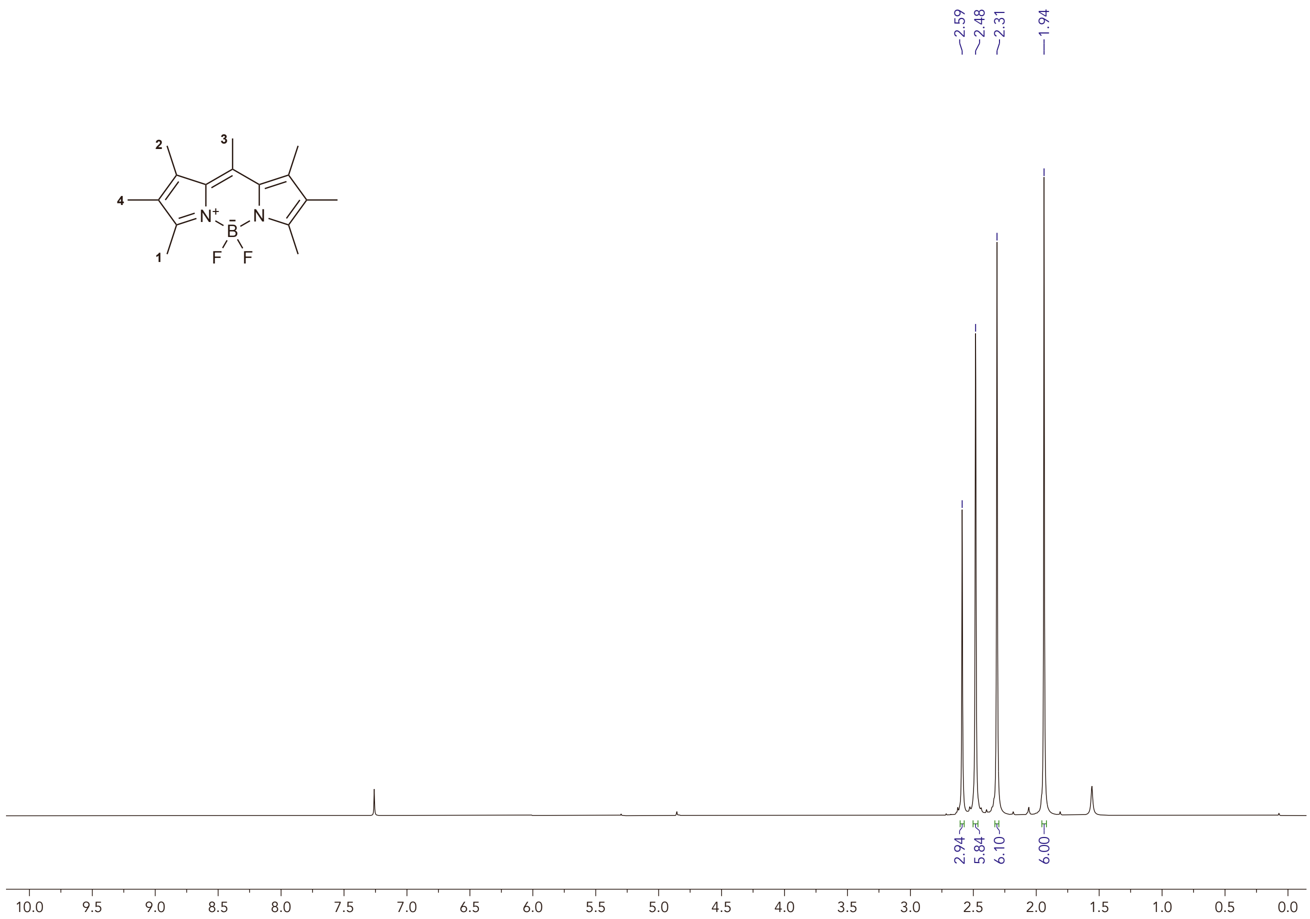

Figure S12. ^{1}H NMR spectrum of **b3** (400 MHz, $CDCl_3$, 298 K).

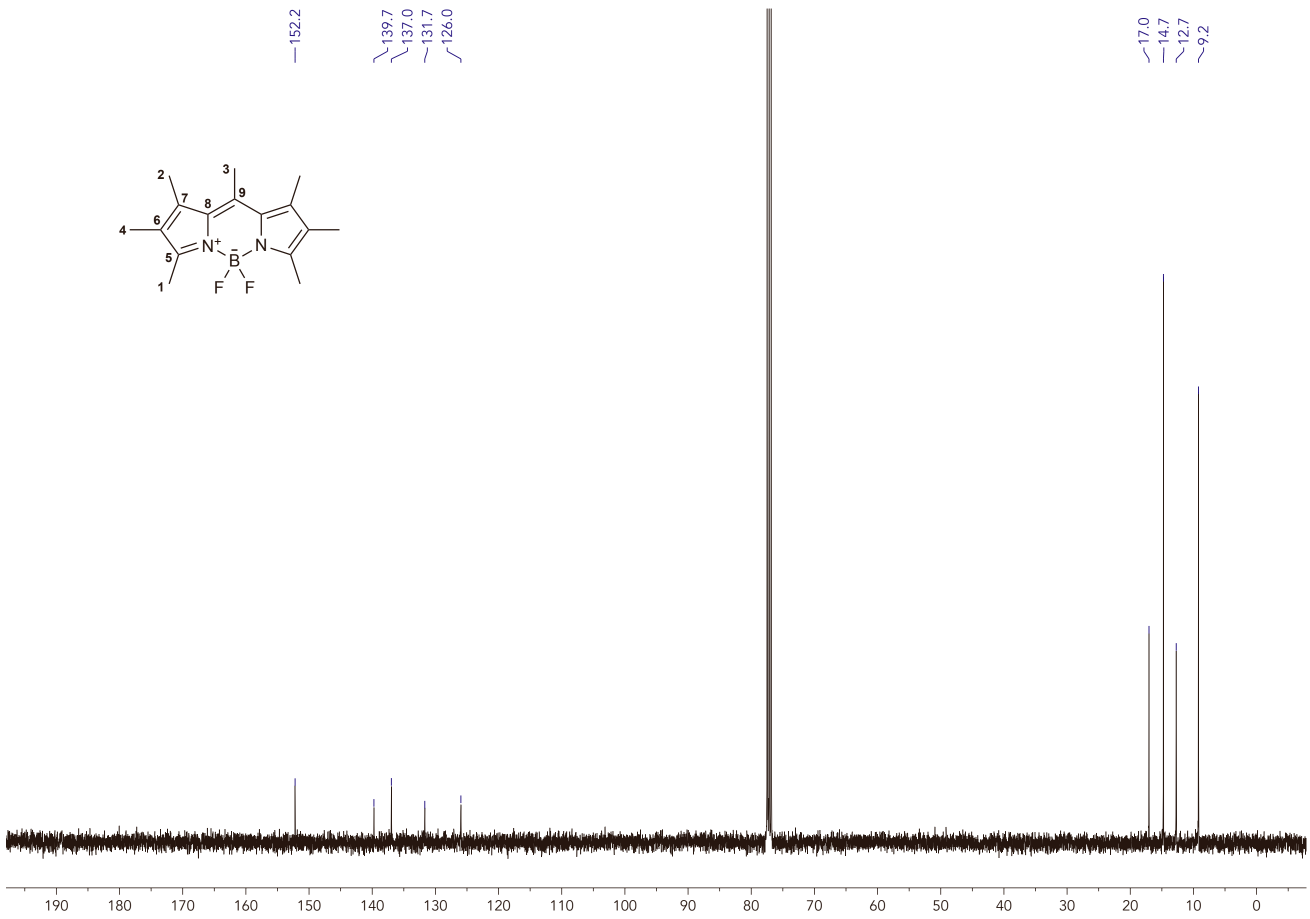

Figure S13. ^{13}C NMR spectrum of **b3** (100 MHz, $CDCl_3$, 298 K).

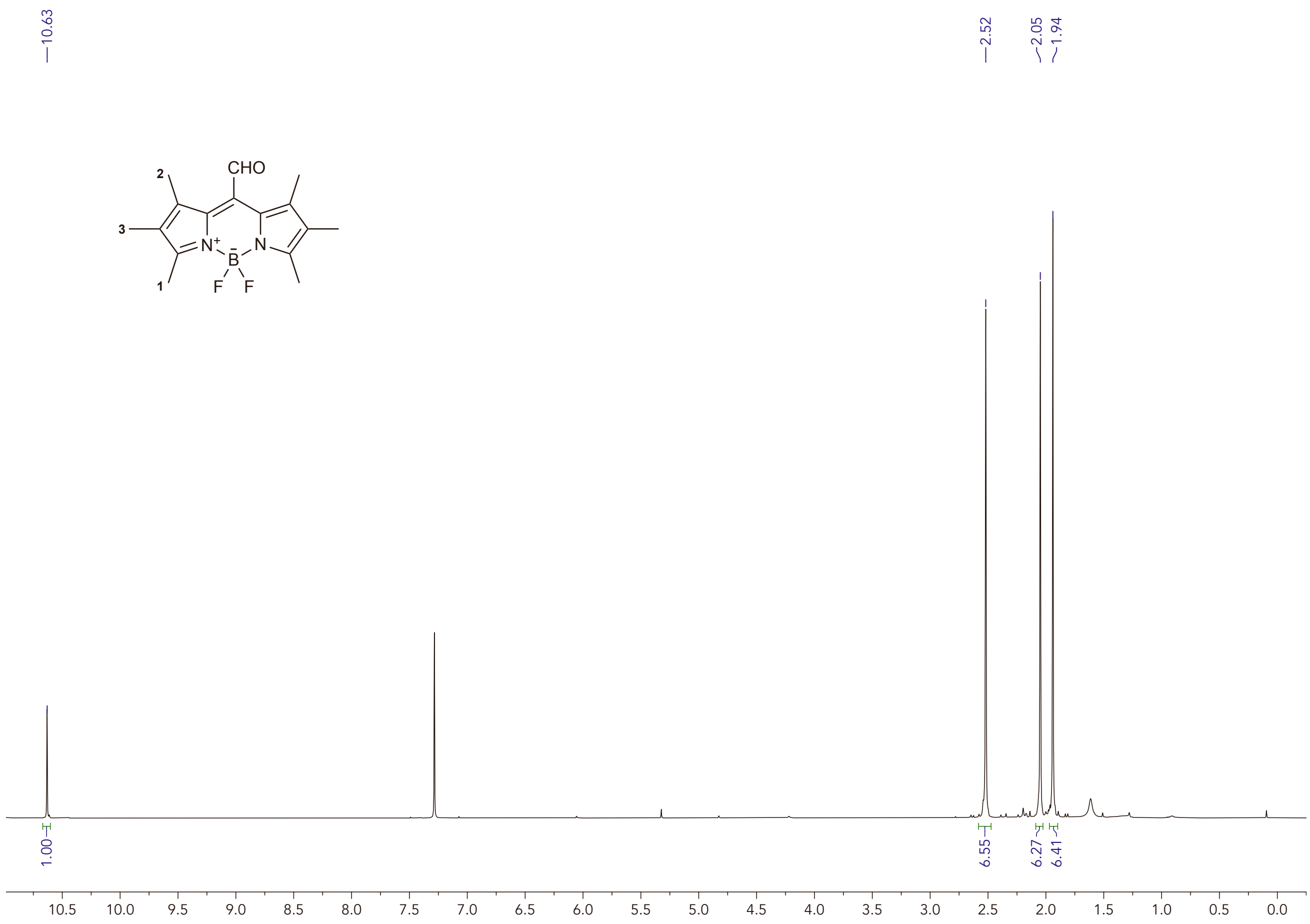

Figure S14. ^{1}H NMR spectrum of **b4** (400 MHz, $CDCl_3$, 298 K).

3. Following the formation of homodimeric complexes

The uptake of guests **a1**, **a2**, and **a4** by the cage over time was monitored by UV-vis absorption spectroscopy. To this end, aliquots from the suspensions were taken at various times, subjected to repeated centrifugations, and analyzed by UV-vis absorption spectroscopy. Representative UV-vis spectra are shown in Figure S15. Based on these results, we concluded that the uptake of all hydrocarbons is largely complete within <10 h. Similar results were obtained for **a3** (not shown). The uptake of BODIPYs was monitored as described previously.[2]

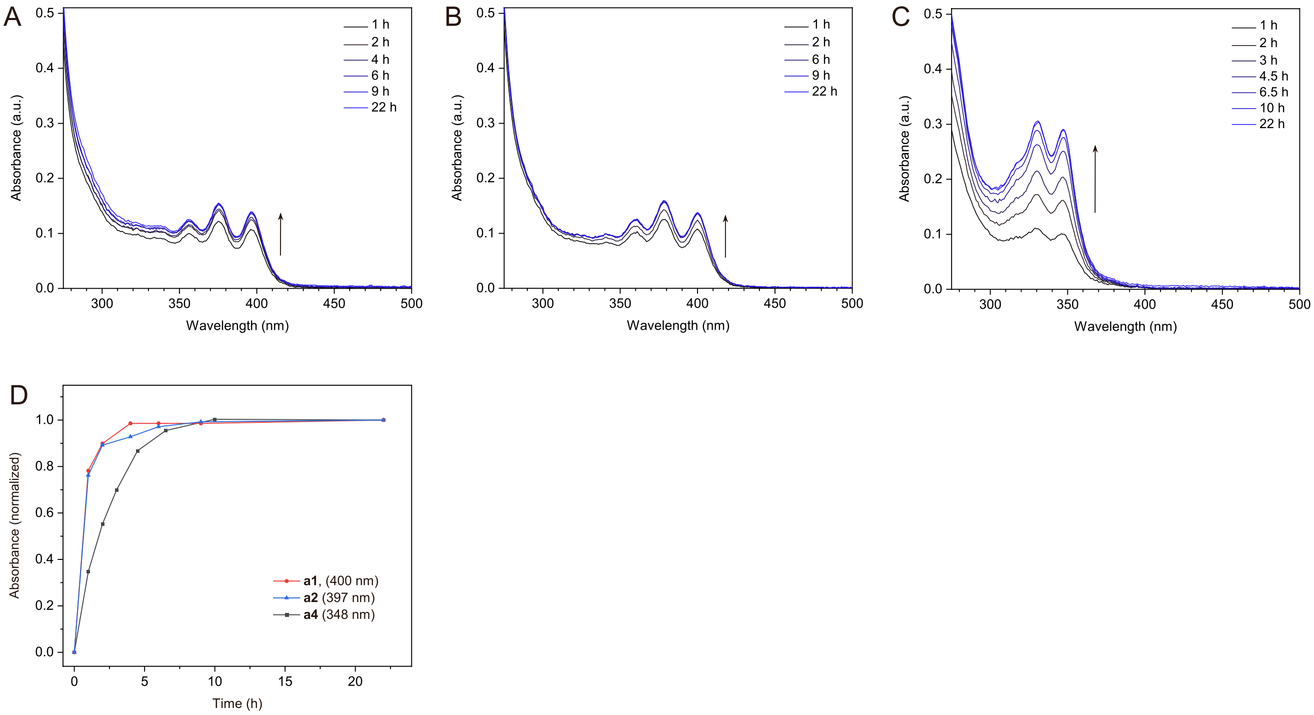

Figure S15. (A–C) Uptake of **a1** (A), **a2** (B), and **a4** (C) by cage **C** followed by UV-vis absorption spectroscopy. (D) Normalized profiles of the uptake of **a1**, **a2**, and **a4** by cage **C**. Absorbance was followed at 400 nm, 397 nm, and 348 nm for **a1**, **a2**, and **a4**, respectively.

4. NMR characterization of BODIPY homodimers

Complexes (**b1**)$_2$⊂**C**, (**b2**)$_2$⊂**C**, (**b3**)$_2$⊂**C**, and (**b4**)$_2$⊂**C** were obtained as described in Section 3. Note that the yield of each complex (i.e., molar fraction of cages that became filled with the guest) depended strongly on the substitution pattern on BODIPY. Specifically, (**b1**)$_2$⊂**C** was obtained with a ~50% yield (i.e., approximately half of the cages remained empty), (**b2**)$_2$⊂**C** was obtained with a ~62% yield, and (**b4**)$_2$⊂**C** was obtained in a near-quantitative yield. The characterization of all three complexes was reported previously.[2]

For (**b3**)$_2$⊂**C**, the encapsulation yield was found to be only ~10% (as determined by NMR; see Figure S16), despite prolonged stirring in the presence of excess of free guest **b3**. The NMR spectrum of the reaction mixture is shown in Figure S16, whereby the high-intensity peaks (e.g., the characteristic peak due to the acidic equatorial imidazole **C**$_4$ at 9.10 ppm) can be assigned to empty cage **C**. The successful formation of (**b3**)$_2$⊂**C** is also evident from the similarity of the ^{1}H NMR spectrum with that of (**b1**)$_2$⊂**C**;[2] in both cases, the characteristic splitting of the filled cage's **C**$_4$ proton is observed[2] (for (**b3**)$_2$⊂**C**, into two singlets at 9.64 and 9.41 ppm). The peaks at 1.58, 0.45, and 0.30 ppm can be assigned to the methyl groups of encapsulated **b3**.

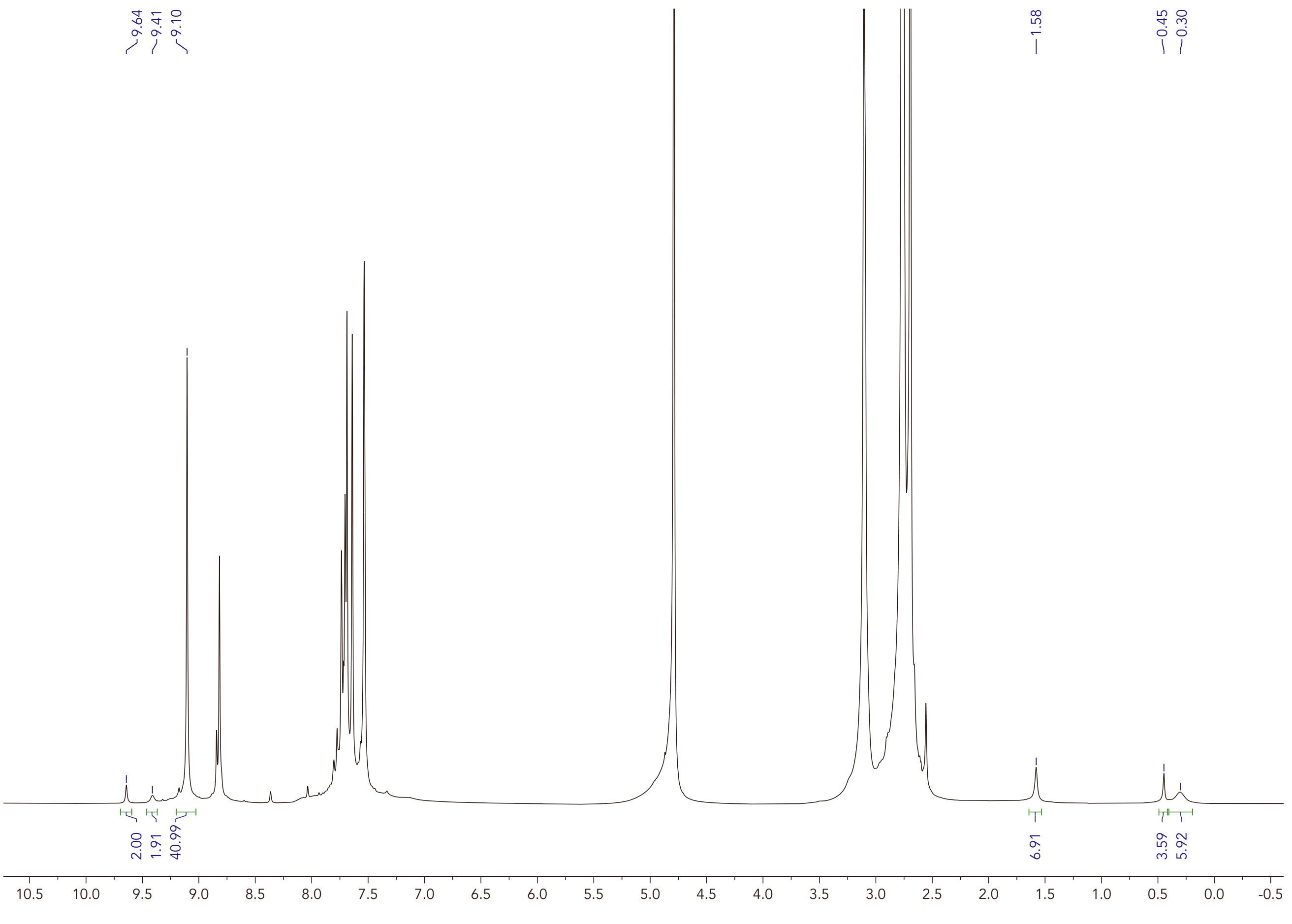

Figure S16. ^{1}H NMR spectrum of (**b3**)$_2$⊂**C** (400 MHz, $CDCl_3$, 298 K).

5. NMR characterization of homodimers (**a1**)$_2$⊂**C**, (**a2**)$_2$⊂**C**, (**a3**)$_2$⊂**C**, and (**a4**)$_2$⊂**C**

Where significant line broadening in the room-temperature ^{1}H NMR spectra was observed (see, e.g., Figure S17; compare with Figure 1A in the main text), the inclusion complexes were characterized at an elevated temperature (typically 330 K).

Inclusion complex (**a1**)$_2$⊂**C** was obtained in a near-quantitative yield, as determined by ^{1}H NMR spectroscopy; the spectrum at 330 K is shown in Figure 1A (main text).

^{1}H NMR (600 MHz, D_2O, 330 K): δ = 9.43 (s, 4H, $\mathbf{C_1}$), 8.93 (s, 8H, $\mathbf{C_4}$), 7.84 (s, 4H, $\mathbf{C_3}$), 7.83 (s, 4H, $\mathbf{C_2}$), 7.56 (s, 8H, $\mathbf{C_5}$), 7.39 (s, 8H, $\mathbf{C_7}$), 7.19 (s, 4H, $\mathbf{C_8}$), 7.15 (s, 8H, $\mathbf{C_6}$), 6.77 (t, 8H, $\mathbf{a1_3}$), 6.43 (s, 6H, $\mathbf{a1_{4+5}}$), 6.32 (br, 4H, $\mathbf{a1_2}$), 6.06 (s, 4H, $\mathbf{a1_1}$), 3.16 (s, 8H, $\mathbf{C_9}$), 3.03 (s, 16H, $\mathbf{C_9}$), 2.85–2.50 (m, 72H, $\mathbf{C_{10}}$), 0.16 (s, 6H, $\mathbf{a1}_{CH_3}$).

^{13}C NMR (150 MHz, D_2O, 330 K): δ = 138.0 ($\mathbf{C_q}$), 137.3 ($\mathbf{C_4}$), 137.2 ($\mathbf{C_{q'}}$), 137.0 ($\mathbf{C_1}$), 130.2 ($\mathbf{C_2}$), 130.0 ($\mathbf{a1_8}$), 129.3 ($\mathbf{C_5}$), 128.2 ($\mathbf{a1_7}$), 128.1 ($\mathbf{a1_6}$), 127.0 ($\mathbf{a1_4}$), 125.5 ($\mathbf{a1_3}$), 125.0 ($\mathbf{a1_2}$), 123.1 ($\mathbf{a1_1}$), 122.6 ($\mathbf{a1_5}$), 121.0 ($\mathbf{C_3}$), 120.6 ($\mathbf{C_6}$), 111.9 ($\mathbf{C_8}$), 111.1 ($\mathbf{C_7}$), 63.2 ($\mathbf{C_9}$), 63.0 ($\mathbf{C_9}$), 50.8 ($\mathbf{C_{10}}$), 50.7 ($\mathbf{C_{10}}$), 50.4 ($\mathbf{C_{10}}$), 11.0 ($\mathbf{a1}_{CH_3}$). (Note: $\mathbf{C_q}$ and $\mathbf{C_{q'}}$ denote H-free C atoms connected to the axial and equatorial imidazoles, respectively.)

^{1}H DOSY NMR (500 MHz, D_2O, 298 K): $D = 0.14\cdot10^{-5}$ cm^2/s.

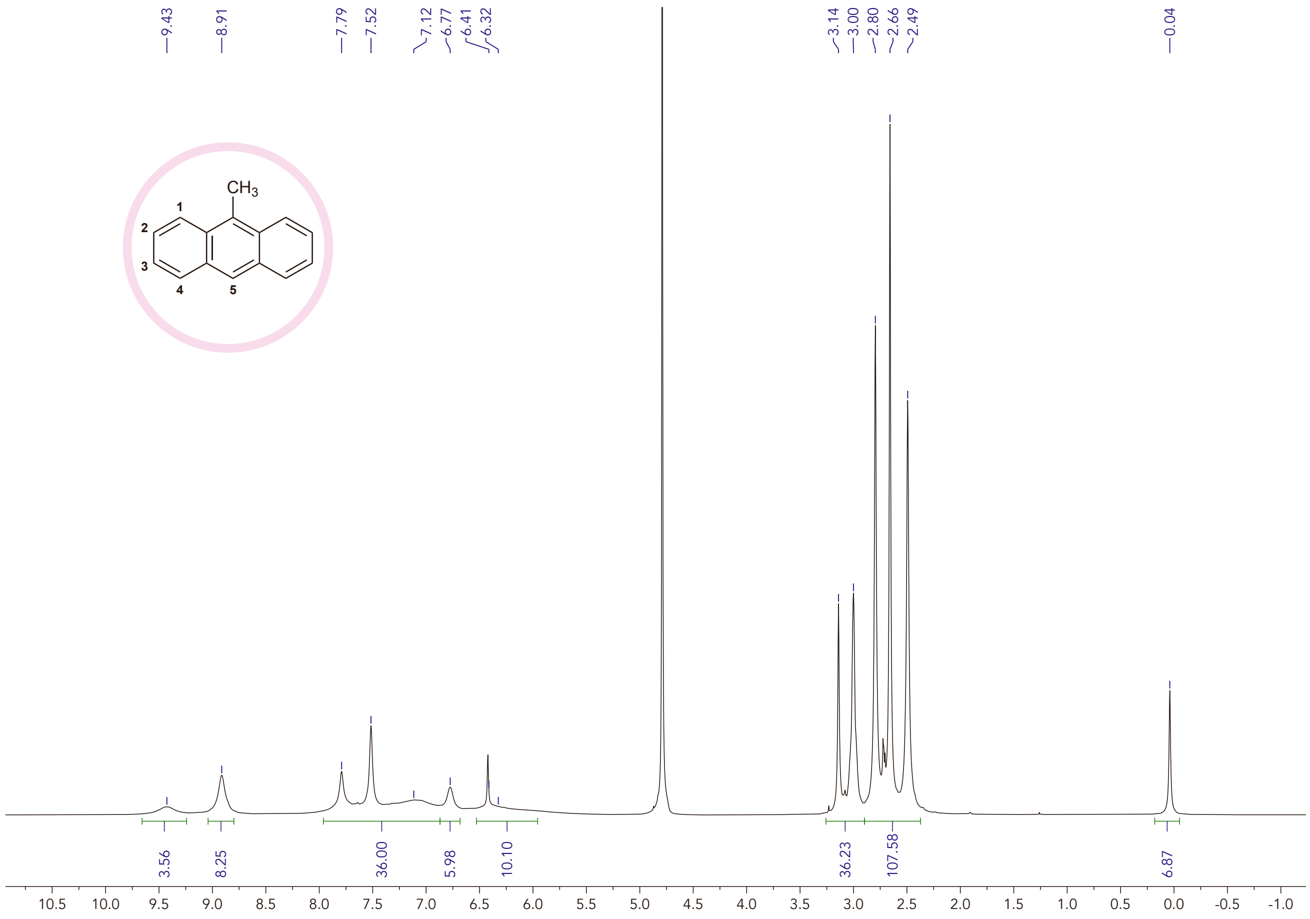

Figure S17. ^{1}H NMR spectrum of (**a1**)$_2$⊂**C** (500 MHz, D_2O, 298 K; for a spectrum at an elevated temperature, see Figure 1A in the main text).

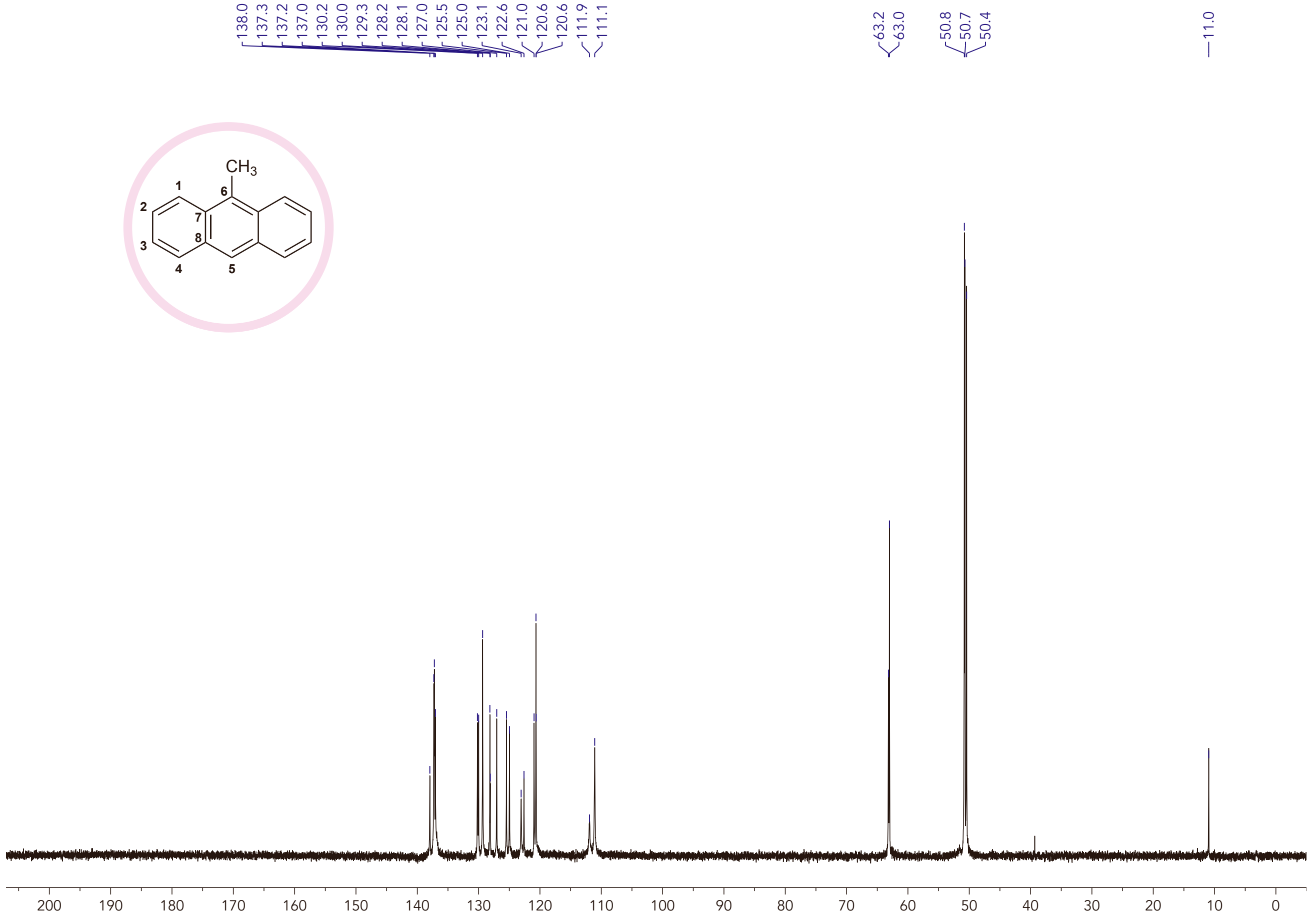

Figure S18. ^{13}C NMR spectrum of (**a1**)$_2$⊂**C** (150 MHz, D_2O, 330 K).

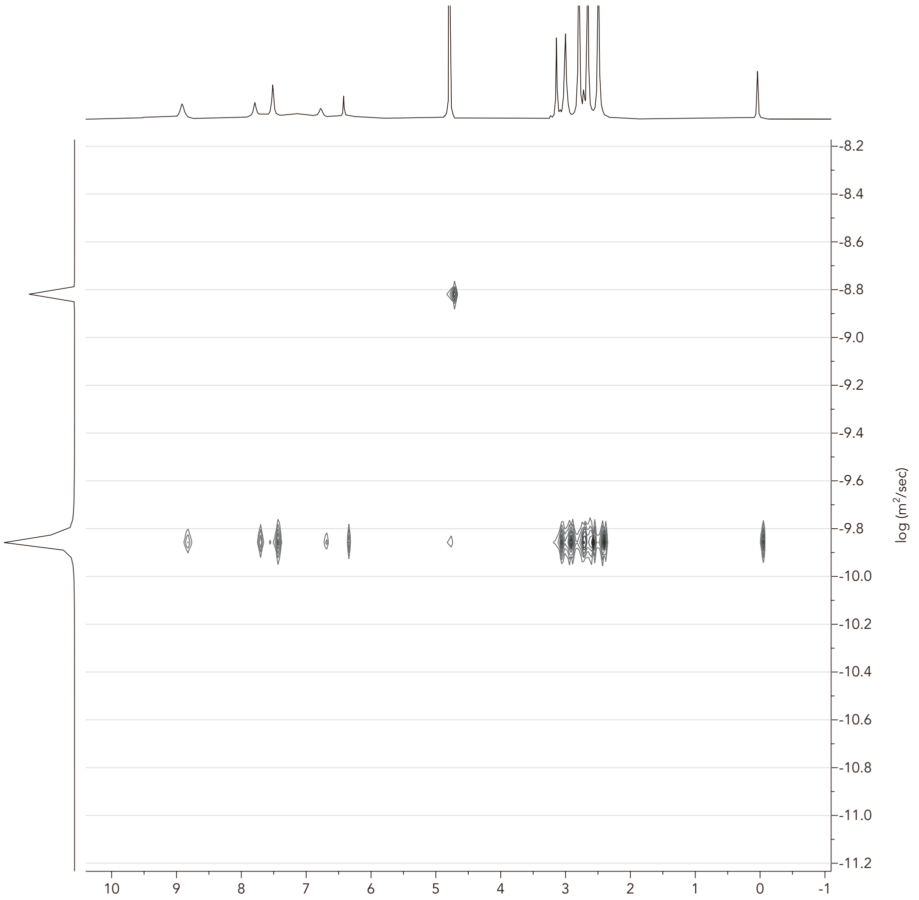

Figure S19. ^{1}H DOSY NMR spectrum of (**a1**)$_2$⊂**C** (500 MHz, D_2O, 298 K).

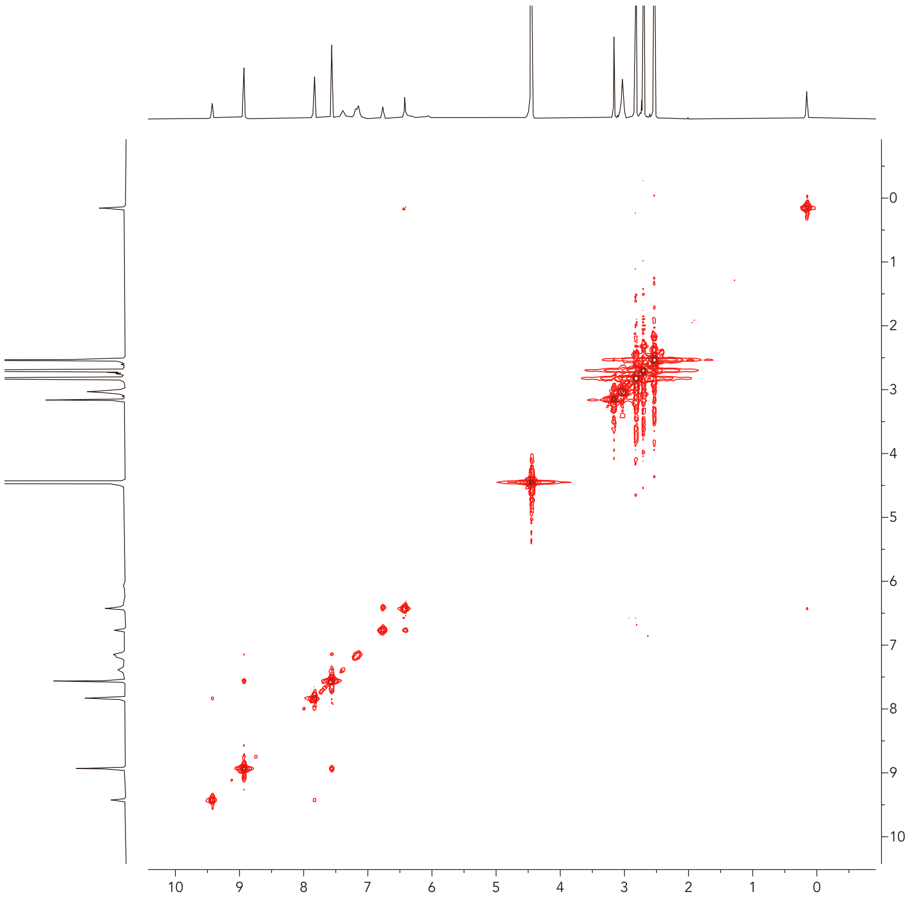

Figure S20. ^{1}H–^{1}H COSY NMR spectrum of (**a1**)$_2$⊂**C** (600 MHz, D_2O, 330 K).

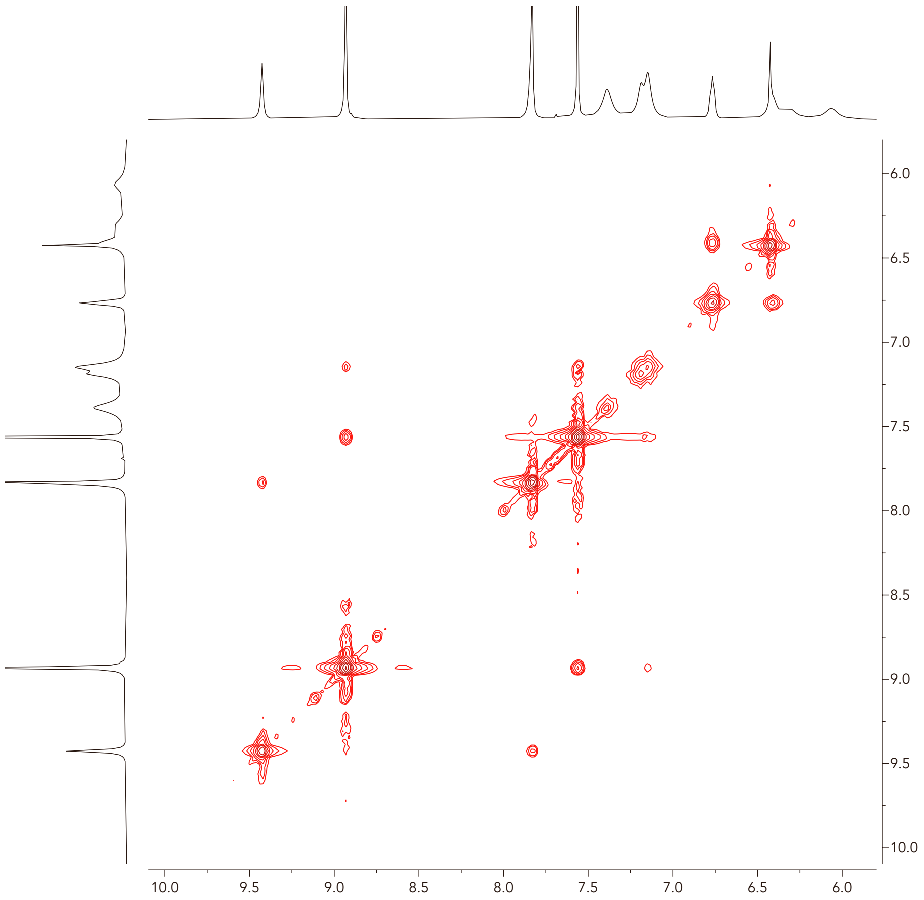

Figure S21. Partial ¹H–¹H COSY NMR spectrum of (**a1**)$_2$⊂**C** (600 MHz, D_2O, 330 K).

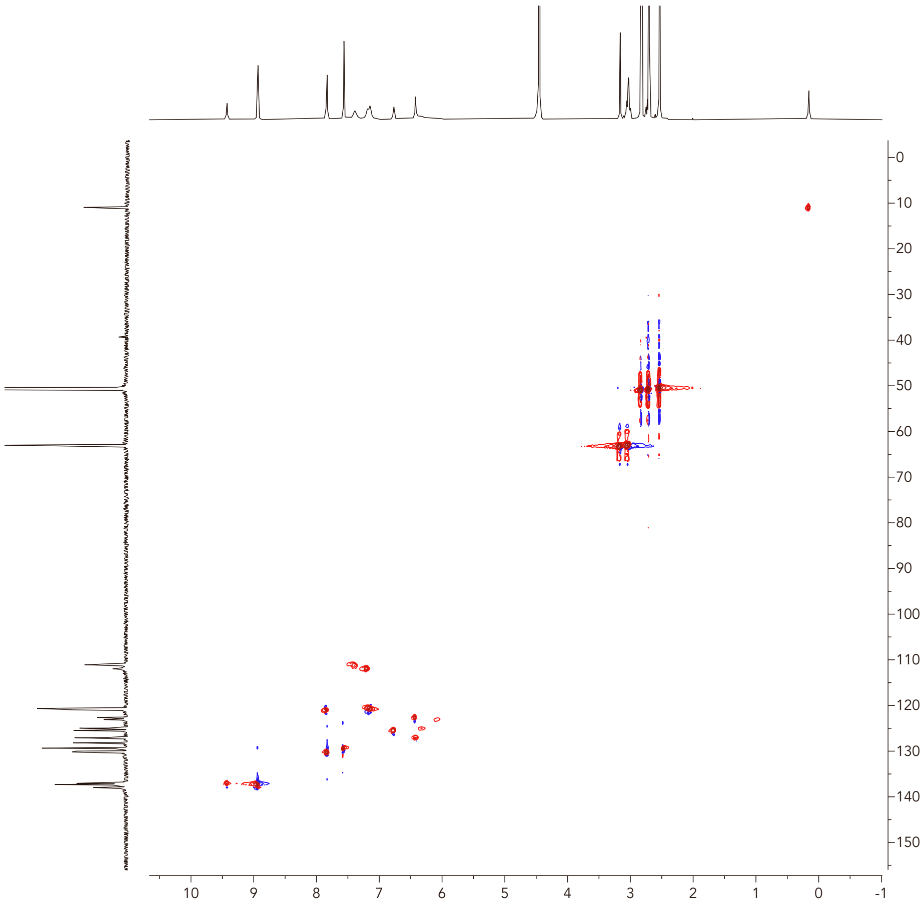

Figure S22. ^{1}H–^{13}C HSQC NMR spectrum of (**a1**)$_2$⊂**C** (600 MHz, D_2O, 330 K).

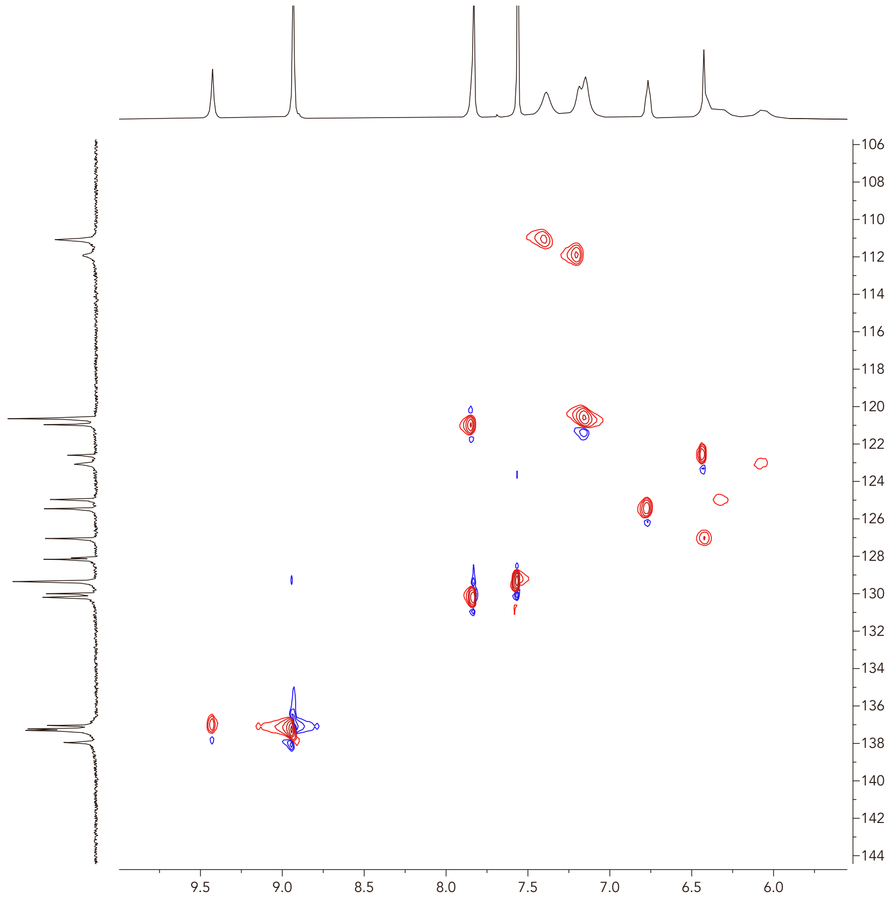

Figure S23. Partial ^{1}H–^{13}C HSQC NMR spectrum of (**a1**)$_2$⊂**C** (600 MHz, D_2O, 330 K).

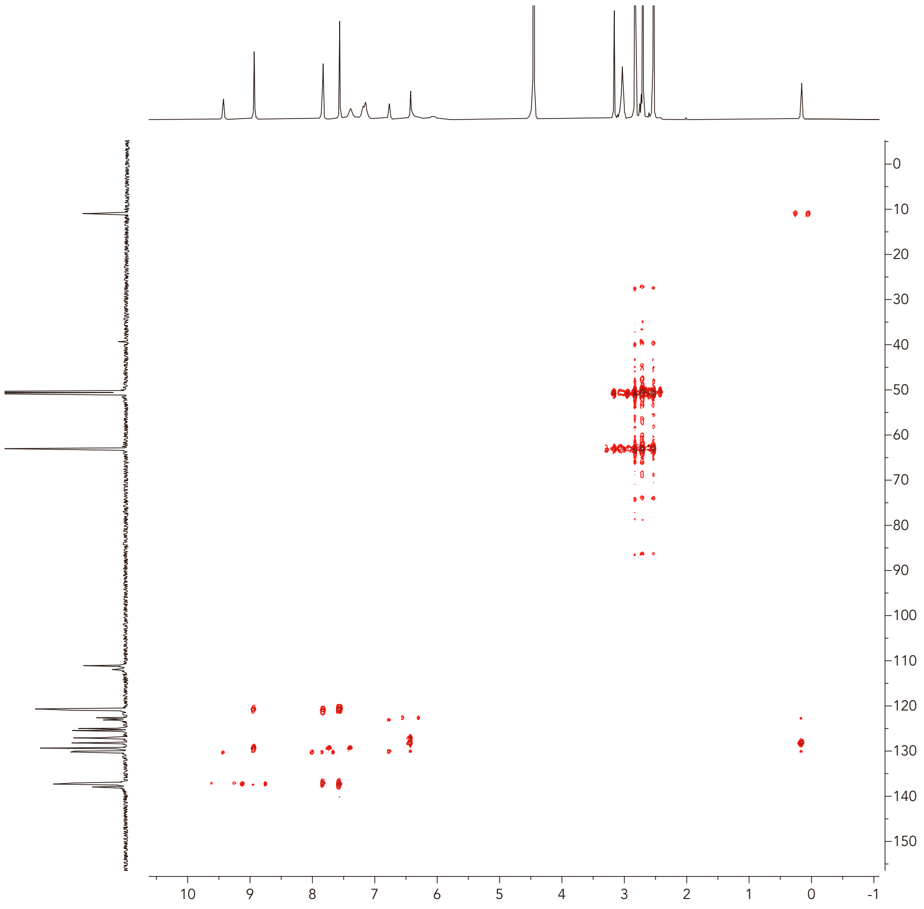

Figure S24. ^{1}H–^{13}C HMBC NMR spectrum of (**a1**)$_2$⊂**C** (600 MHz, D_2O, 330 K).

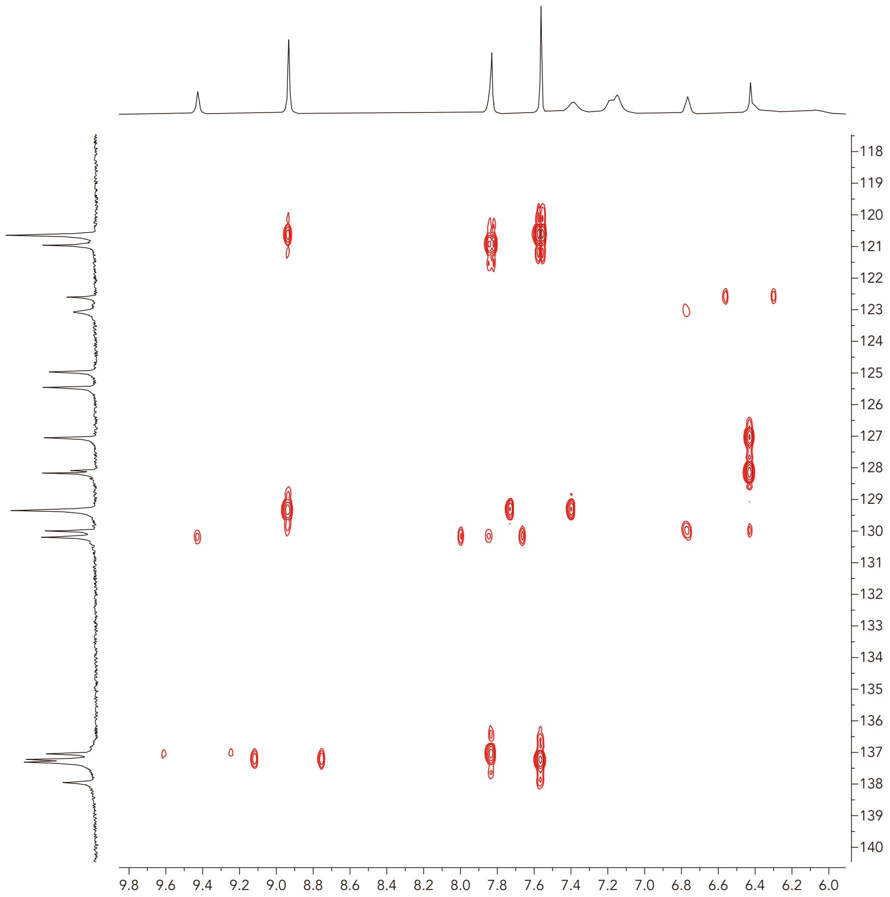

Figure S25. Partial ^{1}H–^{13}C HMBC NMR spectrum of (**a1**)$_2$⊂**C** (600 MHz, D_2O, 330 K).

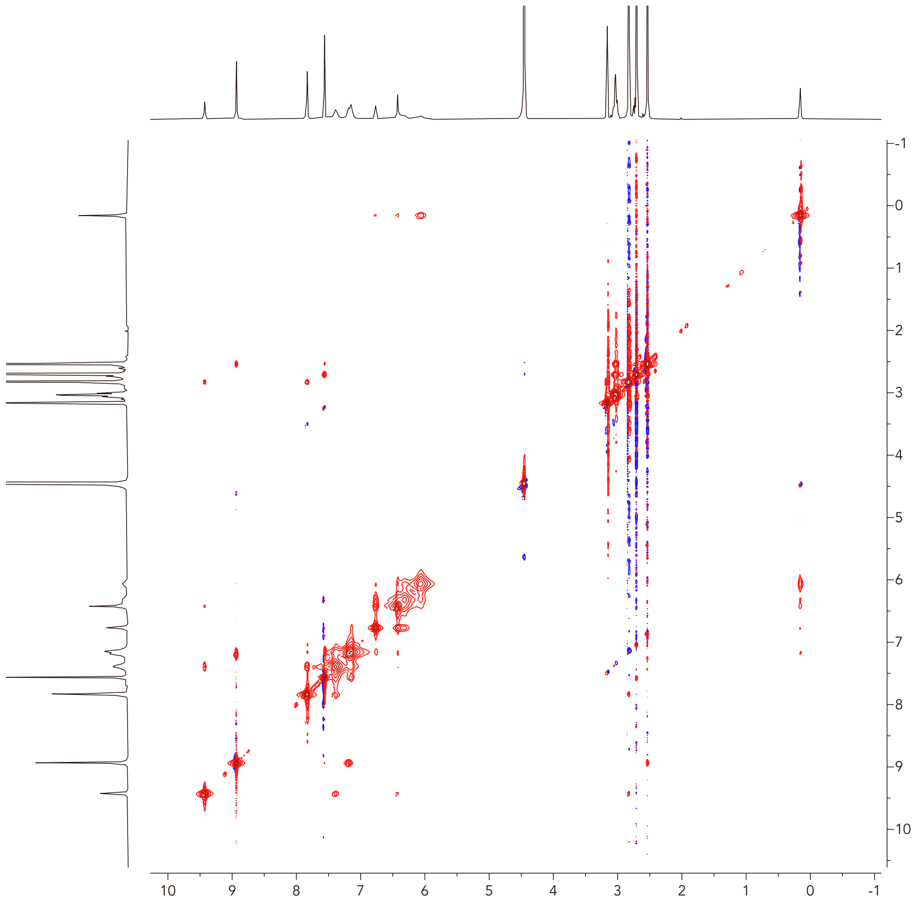

Figure S26. ^{1}H–^{1}H NOESY NMR spectrum of (**a1**)$_2$⊂**C** (600 MHz, D_2O, 330 K).

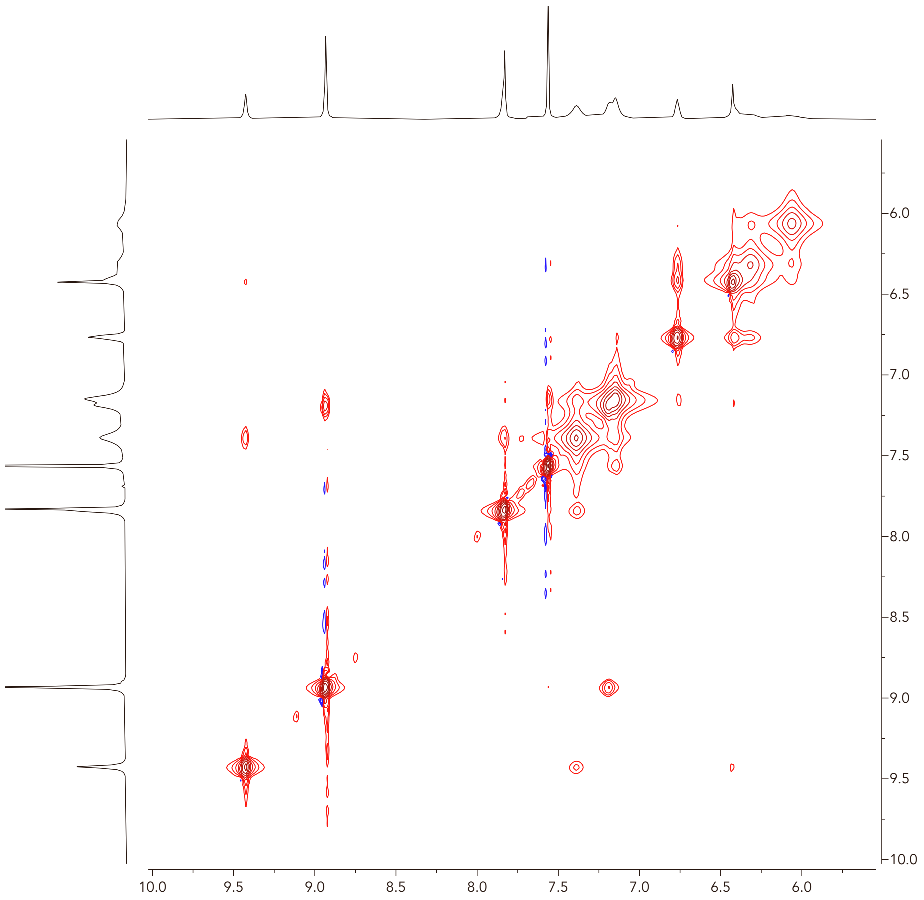

Figure S27. Partial $^1H–^1H$ NOESY NMR spectrum of (**a1**)$_2$⊂**C** (600 MHz, D_2O, 330 K).

Inclusion complex (**a2**)$_2$⊂**C** was obtained in a near-quantitative yield, as determined by ^{1}H NMR spectroscopy.

^{1}H NMR (600 MHz, D_2O, 330 K): δ = 9.32 (s, 4H, **C**$_1$), 8.98 (s, 8H, **C**$_4$), 7.83 (s, 4H, **C**$_3$), 7.79 (s, 4H, **C**$_2$), 7.54 (s, 8H, **C**$_5$), 7.45 (s, 8H, **C**$_7$), 7.33 (s, 12H, **C**$_{6+8}$), 6.56 (s, 8H, **a2**$_{1+3}$), 6.41 (s, 4H, **a2**$_2$), 6.36 (s, 2H, **a2**$_5$), 6.13 (s, 4H, **a2**$_4$), 3.13 (s, 8H, **C**$_9$), 3.03 (s, 16H, **C**$_9$), 2.82–2.47 (m, 72H, **C**$_{10}$).

^{13}C NMR (150 MHz, D_2O, 330 K): δ = 138.1 (**C**$_{q'}$), 137.4 (**C**$_4$), 137.2 (**C**$_1$), 136.9 (**C**$_q$), 130.4 (**a2**$_8$), 130.0 (**C**$_2$), 129.4 (**C**$_5$), 128.3 (**a2**$_7$), 127.0 (**a2**$_3$), 126.6 (**a2**$_4$), 125.7 (**a2**$_1$), 125.6 (**a2**$_2$), 124.6 (**a2**$_5$), 120.8 (**C**$_3$), 120.6 (**a2**$_6$), 120.4 (**C**$_6$), 111.9 (**C**$_8$), 110.8 (**C**$_7$), 63.1 (**C**$_9$), 63.0 (**C**$_9$), 50.8 (**C**$_{10}$), 50.7 (**C**$_{10}$), 50.4 (**C**$_{10}$). (Note: **C**$_q$ and **C**$_{q'}$ denote H-free C atoms connected to the axial and equatorial imidazoles, respectively.)

^{1}H DOSY NMR (500 MHz, D_2O, 298 K): $D = 0.15\cdot10^{-5}$ cm^2/s.

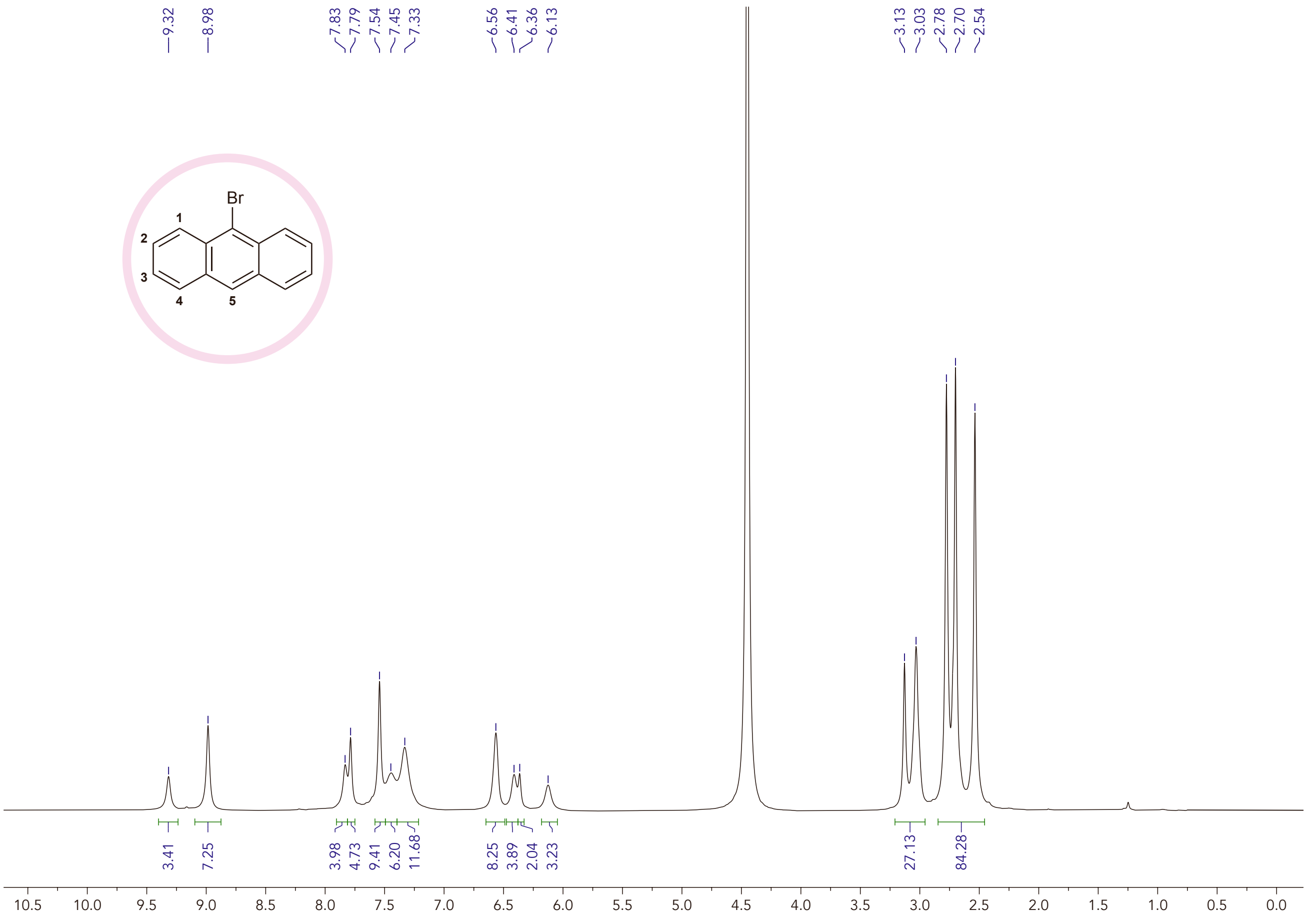

Figure S28. ^{1}H NMR spectrum of (**a2**)$_2$⊂**C** (600 MHz, D_2O, 330 K).

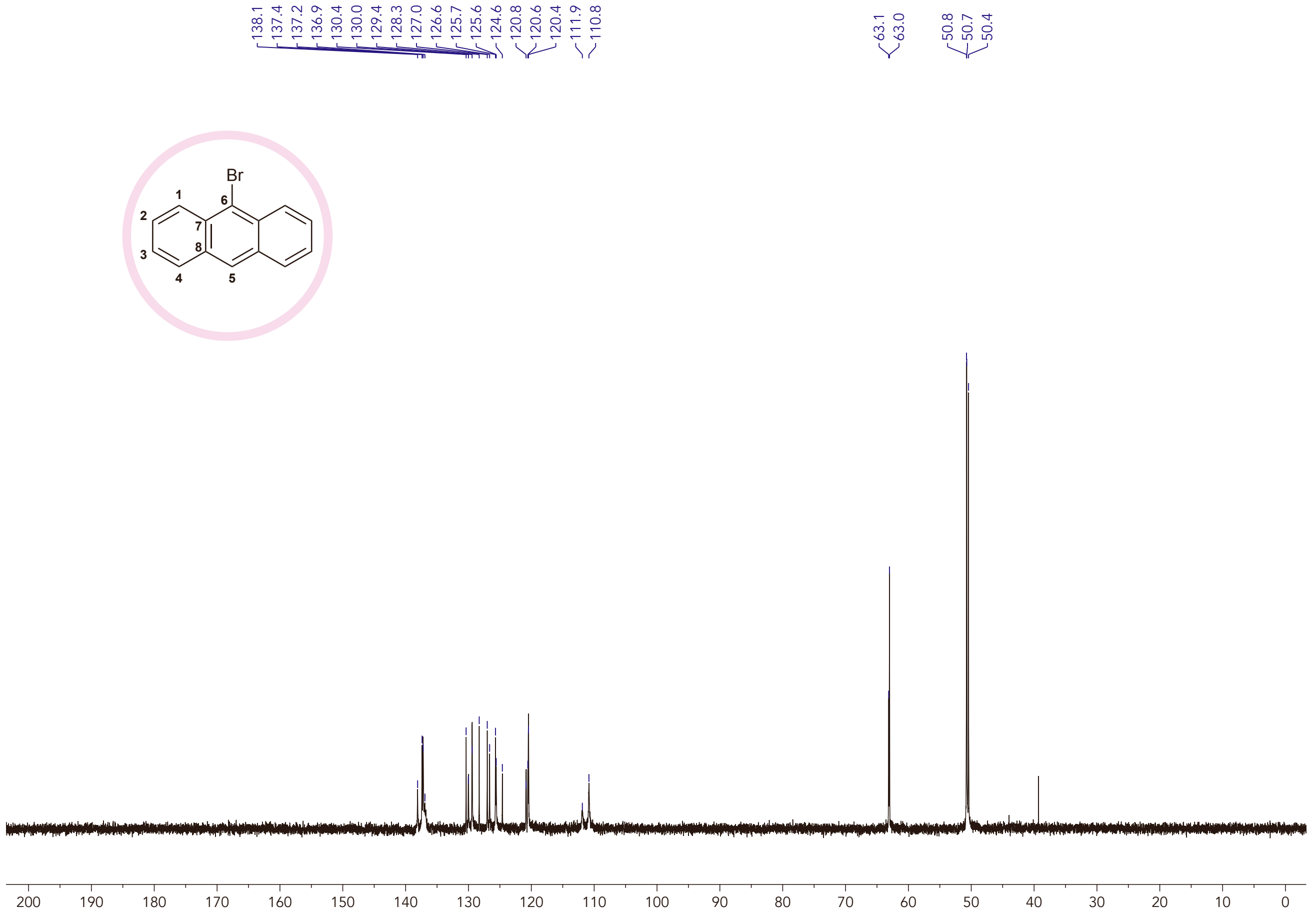

Figure S29. ^{13}C NMR spectrum of (**a2**)$_2$⊂**C** (150 MHz, D_2O, 330 K).

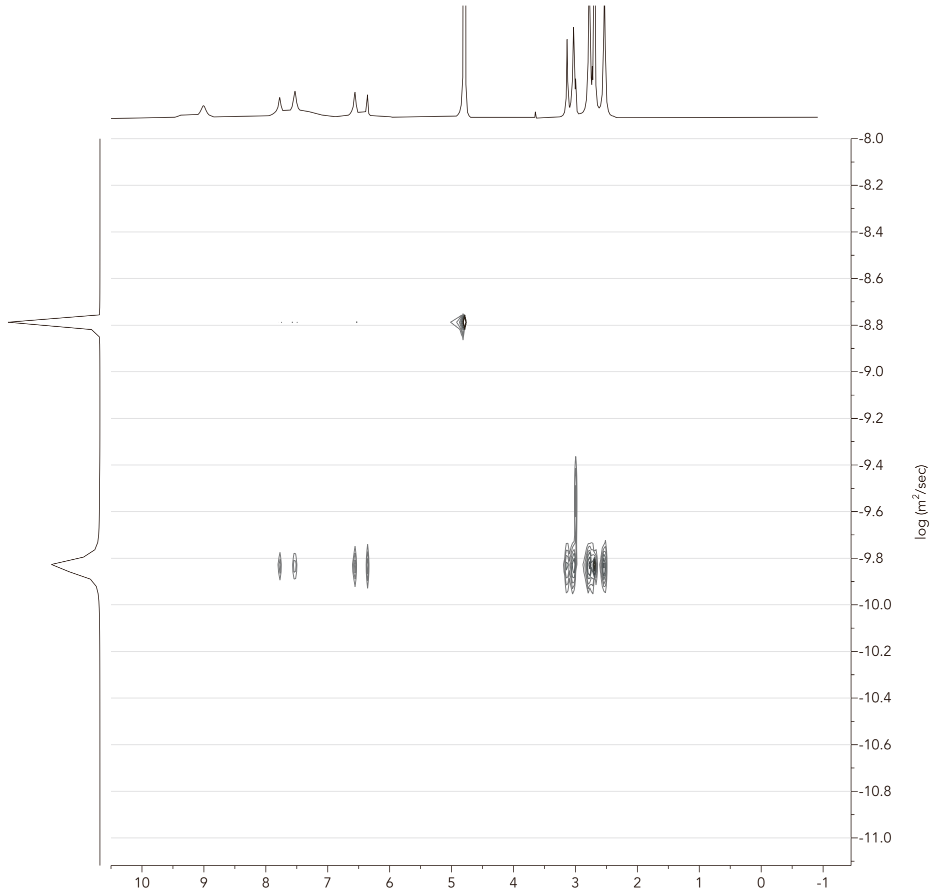

Figure S30. ^{1}H DOSY NMR spectrum of (**a2**)$_2$⊂**C** (500 MHz, D_2O, 298 K).

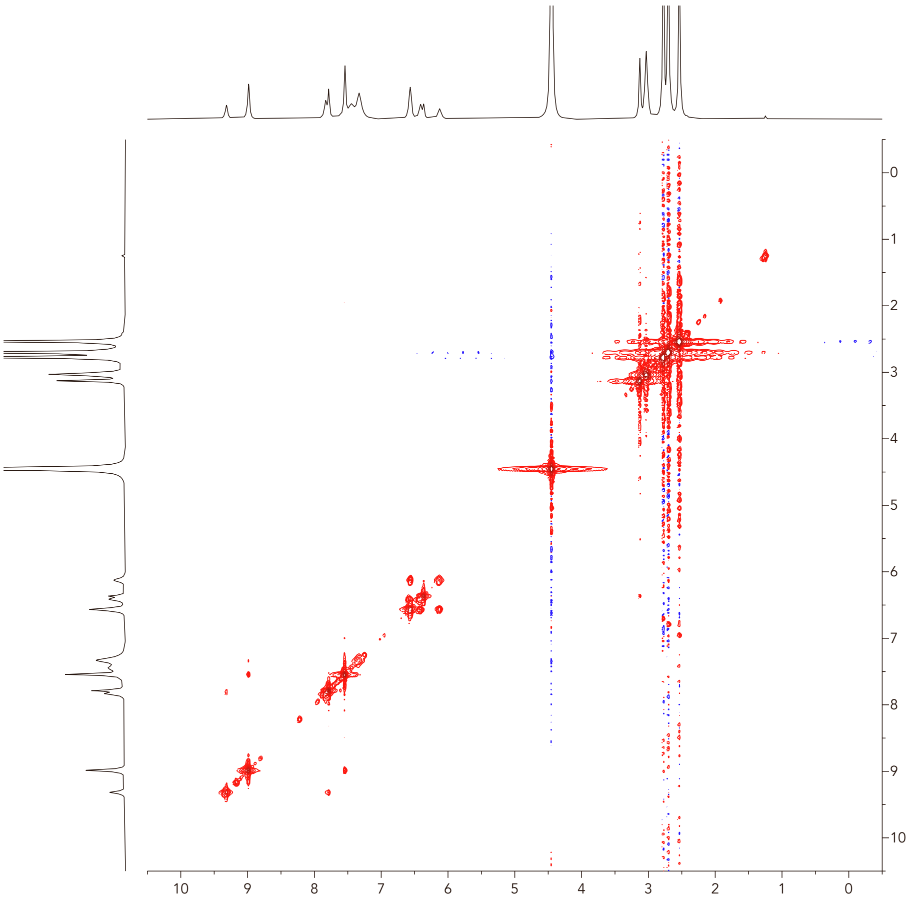

Figure S31. ^{1}H–^{1}H COSY NMR spectrum of (**a2**)$_2$⊂**C** (600 MHz, D_2O, 330 K).

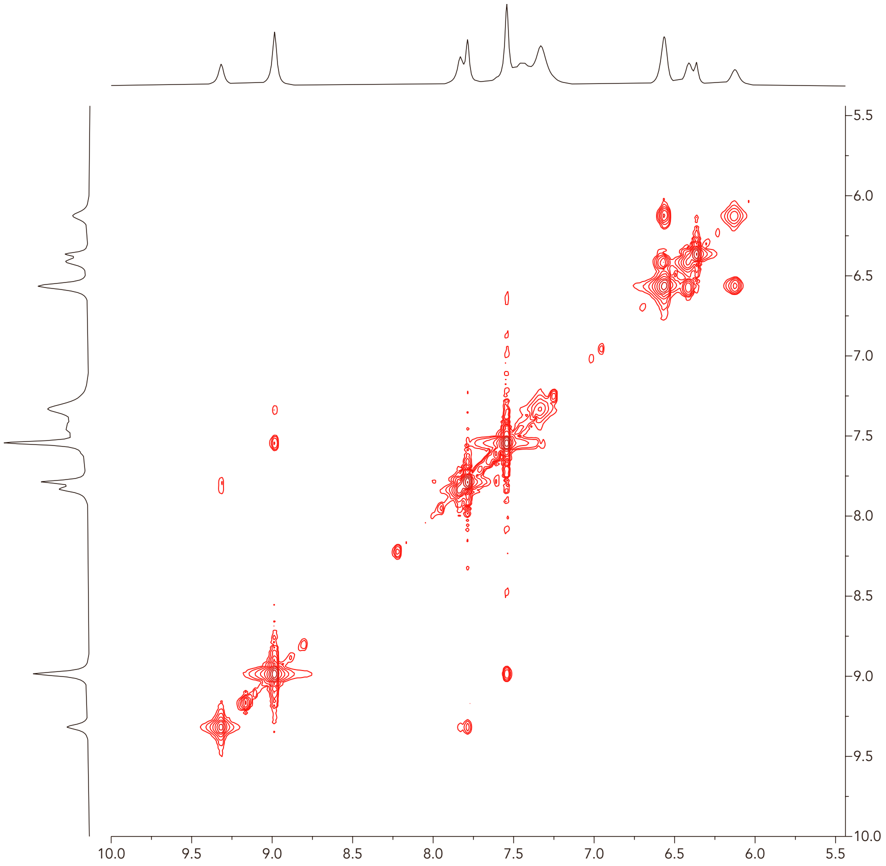

Figure S32. Partial ^{1}H–^{1}H COSY NMR spectrum of (**a2**)$_2$⊂**C** (600 MHz, D_2O, 330 K).

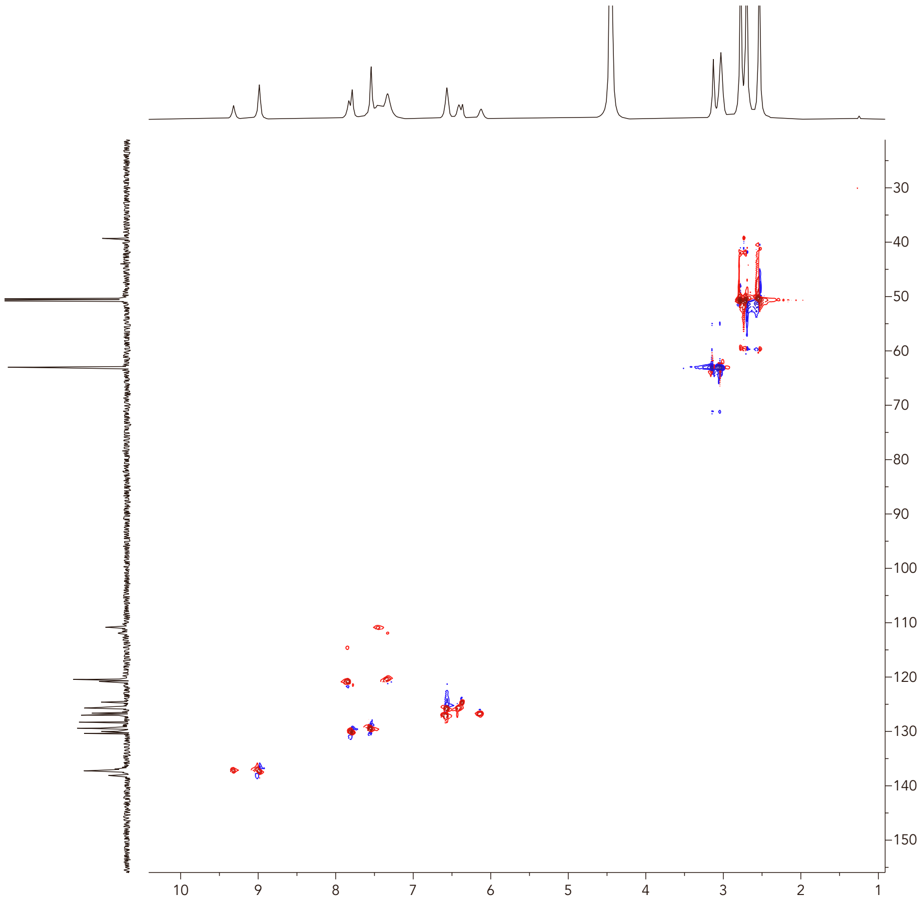

Figure S33. ^{1}H–^{13}C HSQC NMR spectrum of $(\mathbf{a2})_2{\subset}\mathbf{C}$ (600 MHz, D_2O, 330 K).

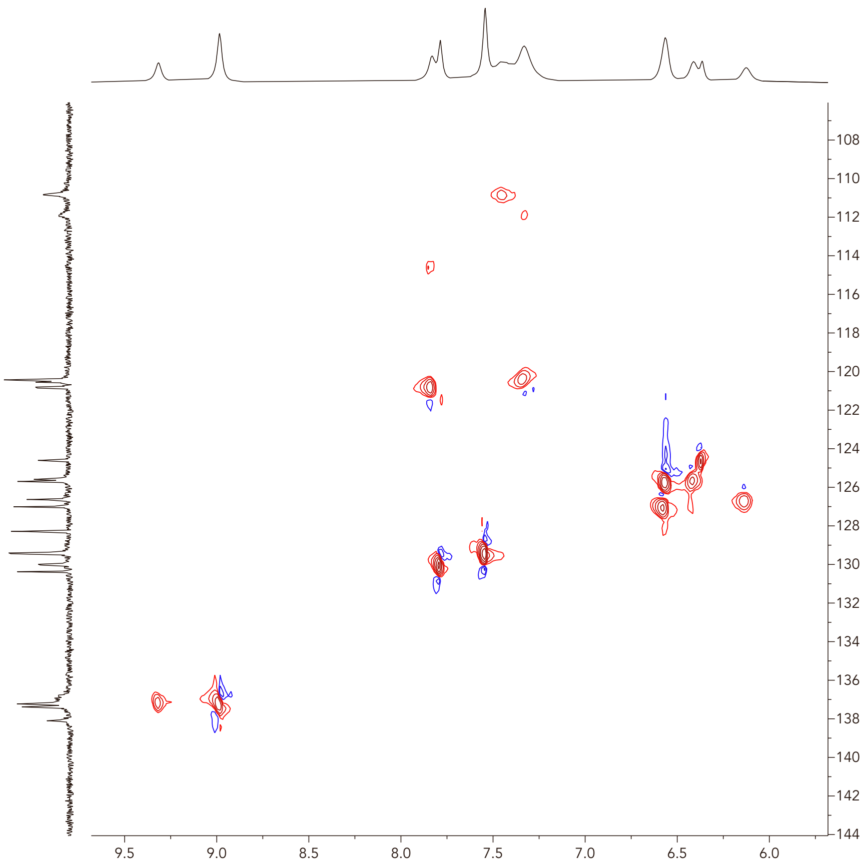

Figure S34. Partial ^{1}H–^{13}C HSQC NMR spectrum of (**a2**)$_2$⊂**C** (600 MHz, D_2O, 330 K).

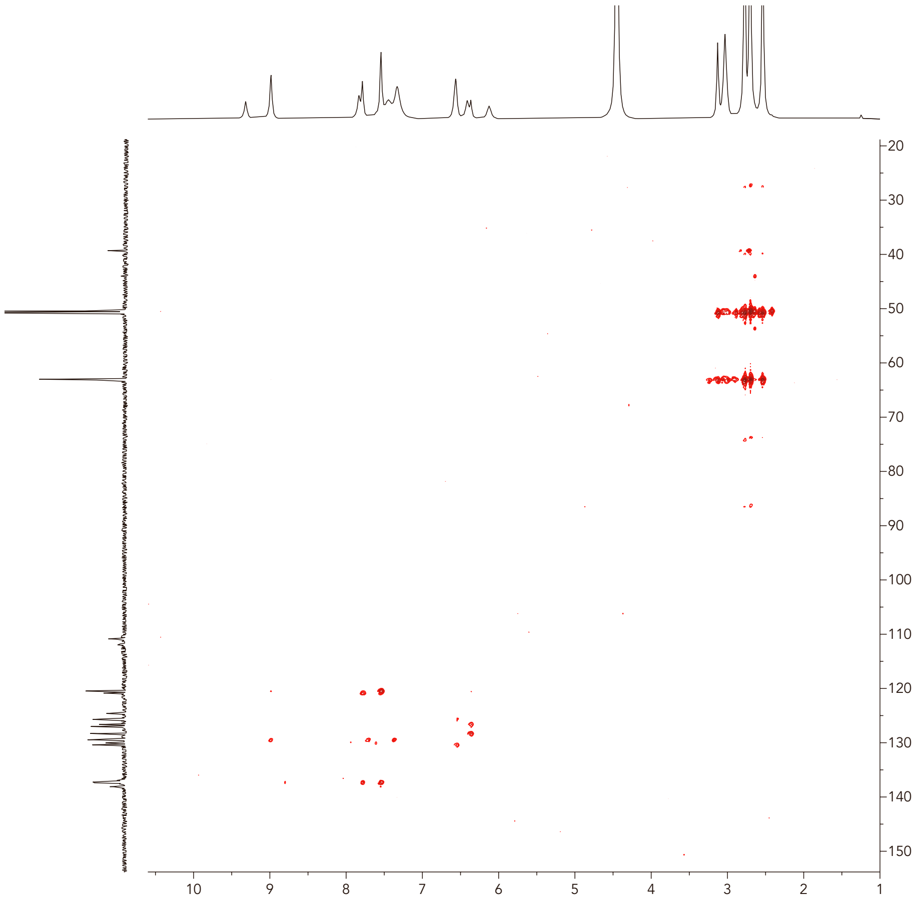

Figure S35. $^{1}H–^{13}C$ HMBC NMR spectrum of (**a2**)$_2$⊂**C** (600 MHz, D_2O, 330 K).

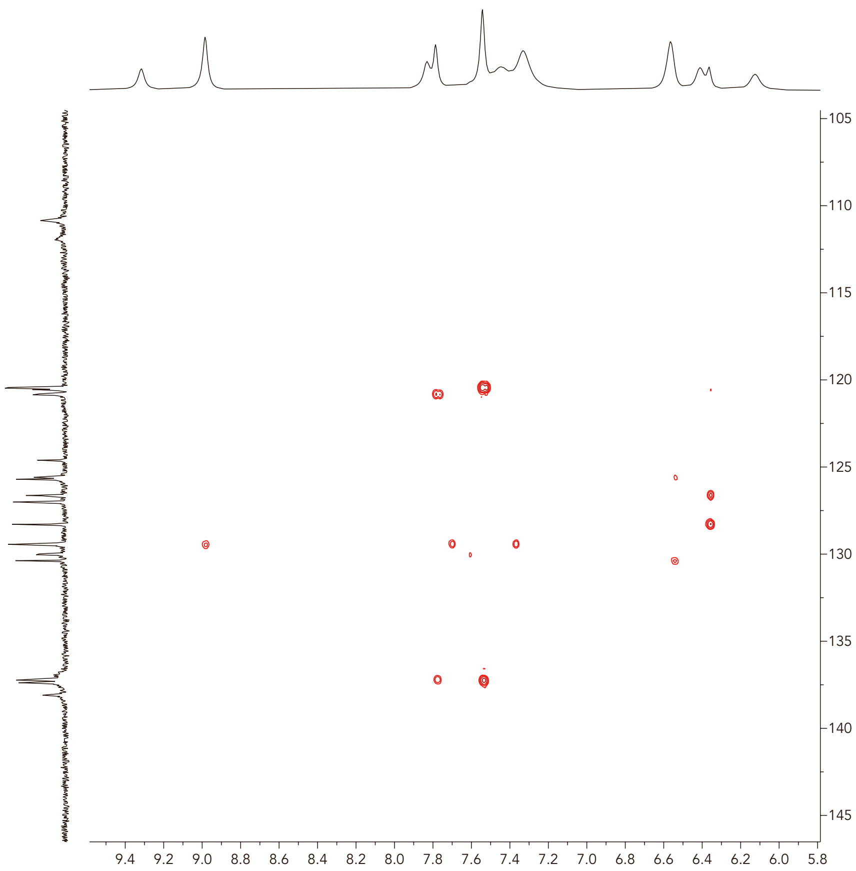

Figure S36. Partial ^{1}H–^{13}C HMBC NMR spectrum of (**a2**)$_2$⊂**C** (600 MHz, D_2O, 330 K).

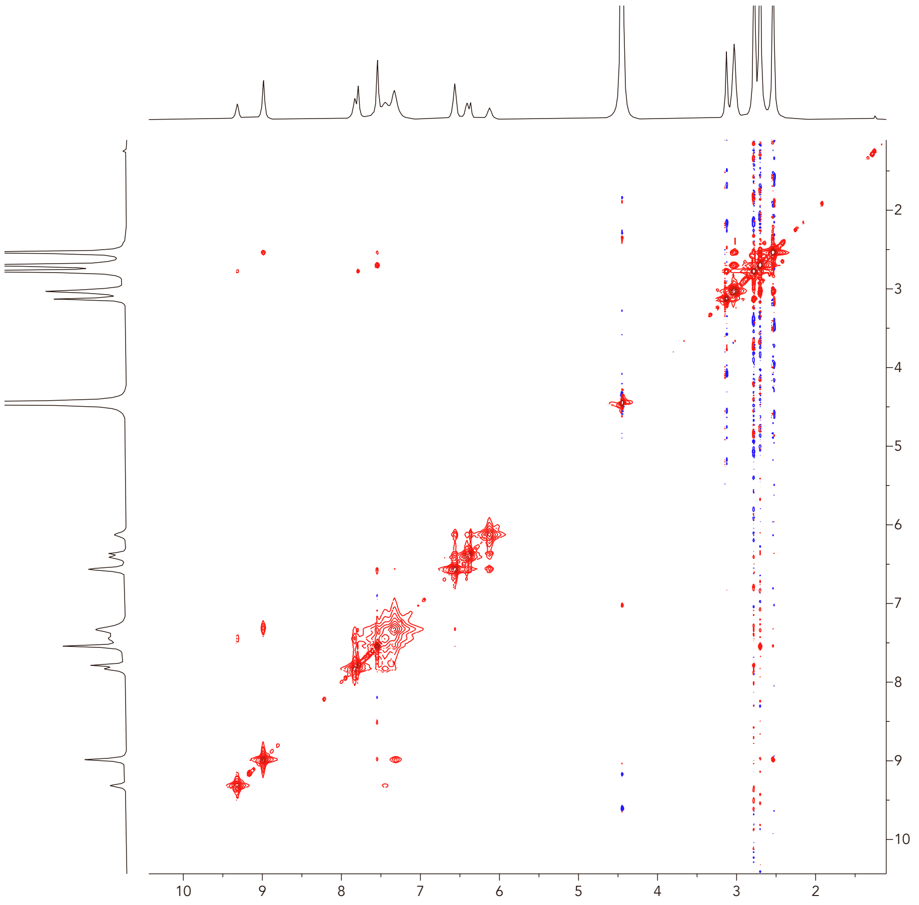

Figure S37. ^{1}H–^{1}H NOESY NMR spectrum of (**a2**)$_2$⊂**C** (600 MHz, D_2O, 330 K).

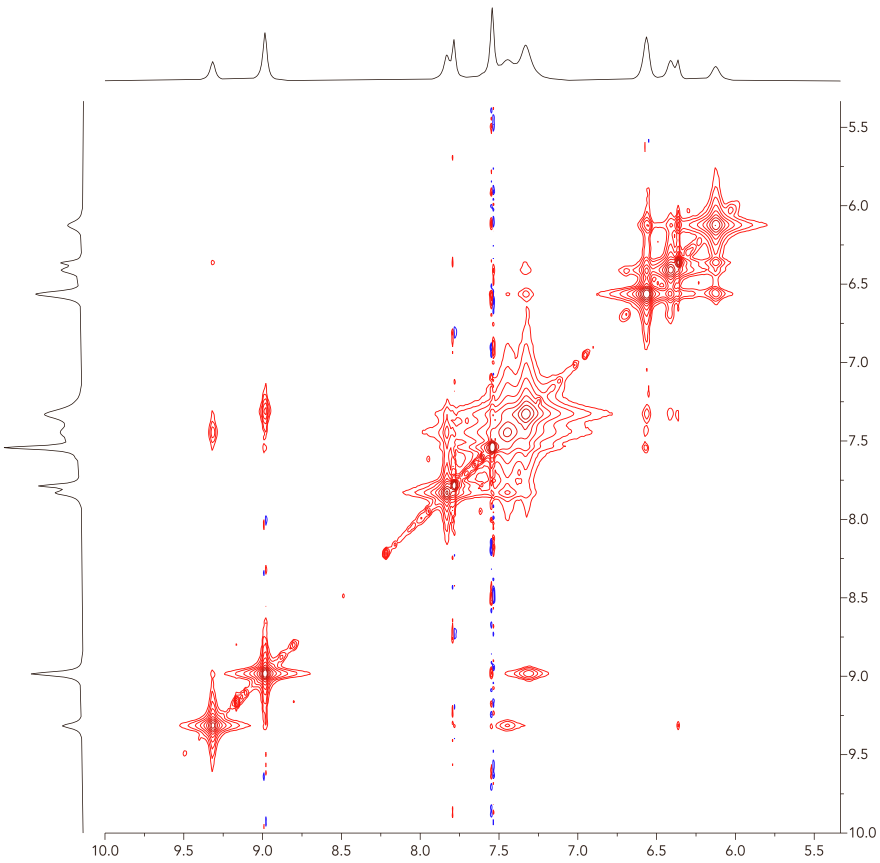

Figure S38. Partial ^{1}H–^{1}H NOESY NMR spectrum of (**a2**)$_2$⊂**C** (600 MHz, D_2O, 330 K).

Inclusion complex $(\mathbf{a3})_2\subset\mathbf{C}$ was obtained in a near-quantitative yield, as determined by ^{1}H NMR spectroscopy.

^{1}H NMR (500 MHz, D_2O, 298 K): δ = 9.22 (s, 4H, $\mathbf{C_1}$), 9.02 (s, 8H, $\mathbf{C_4}$), 7.73 (s, 4H, $\mathbf{C_3}$), 7.68 (s, 4H, $\mathbf{C_2}$), 7.57 (s, 8H, $\mathbf{C_5}$), 7.47 (s, 8H, $\mathbf{C_7}$), 7.37 (s, 8H, $\mathbf{C_6}$), 7.32 (s, 4H, $\mathbf{C_8}$), 6.83 (s, 4H, $\mathbf{a3_1}$), 6.45 (s, 4H, $\mathbf{a3_2}$), 6.16 (s, 4H, $\mathbf{a3_3}$), 6.00 (s, 2H, $\mathbf{a3_5}$), 5.78 (s, 4H, $\mathbf{a3_4}$), 3.64 (s, 4H, $\mathbf{a3}_{CH_2}$), 3.12 (s, 8H, $\mathbf{C_9}$), 3.05 (s, 16H, $\mathbf{C_9}$), 2.76–2.56 (m, 72H, $\mathbf{C_{10}}$).

^{13}C NMR (150 MHz, D_2O, 300 K): δ = 138.0 ($\mathbf{C_{q+q'}}$), 137.1 ($\mathbf{C_1}$), 137.1 ($\mathbf{C_4}$), 129.3 ($\mathbf{a4_7}$), 129.2 ($\mathbf{C_2}$), 129.0 ($\mathbf{C_5}$+$\mathbf{a4_6}$), 127.8 ($\mathbf{a3_8}$), 126.4 ($\mathbf{a3_4}$), 125.2 ($\mathbf{a3_2}$), 124.6 ($\mathbf{a3_5}$), 124.3 ($\mathbf{a3_3}$), 122.3 ($\mathbf{a3_1}$), 120.5 ($\mathbf{C_3}$), 120.3 ($\mathbf{C_6}$), 112.5 ($\mathbf{C_8}$), 111.4 ($\mathbf{C_7}$), 62.6 ($\mathbf{C_9}$), 62.5 ($\mathbf{C_9}$), 54.4 ($\mathbf{a3}_{CH_2}$), 50.3 ($\mathbf{C_{10}}$), 50.0 ($\mathbf{C_{10}}$). (Note: $\mathbf{C_q}$ and $\mathbf{C_{q'}}$ denote H-free C atoms connected to the axial and equatorial imidazoles, respectively.)

^{1}H DOSY NMR (500 MHz, D_2O, 300 K): $D = 0.18\cdot10^{-5}$ cm^2/s.

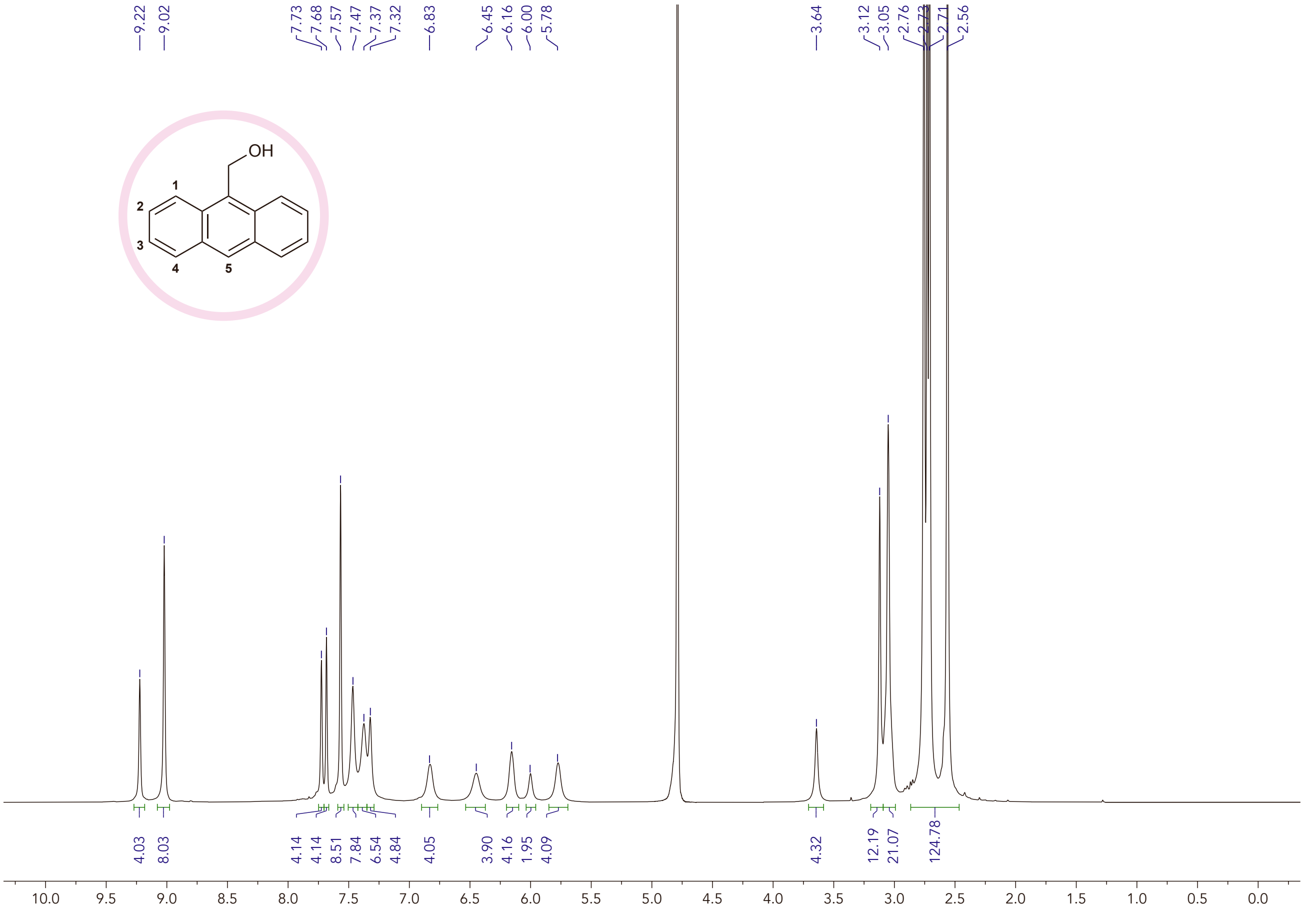

Figure S39. ^{1}H NMR spectrum of $(\mathbf{a3})_2\subset\mathbf{C}$ (500 MHz, D_2O, 298 K).

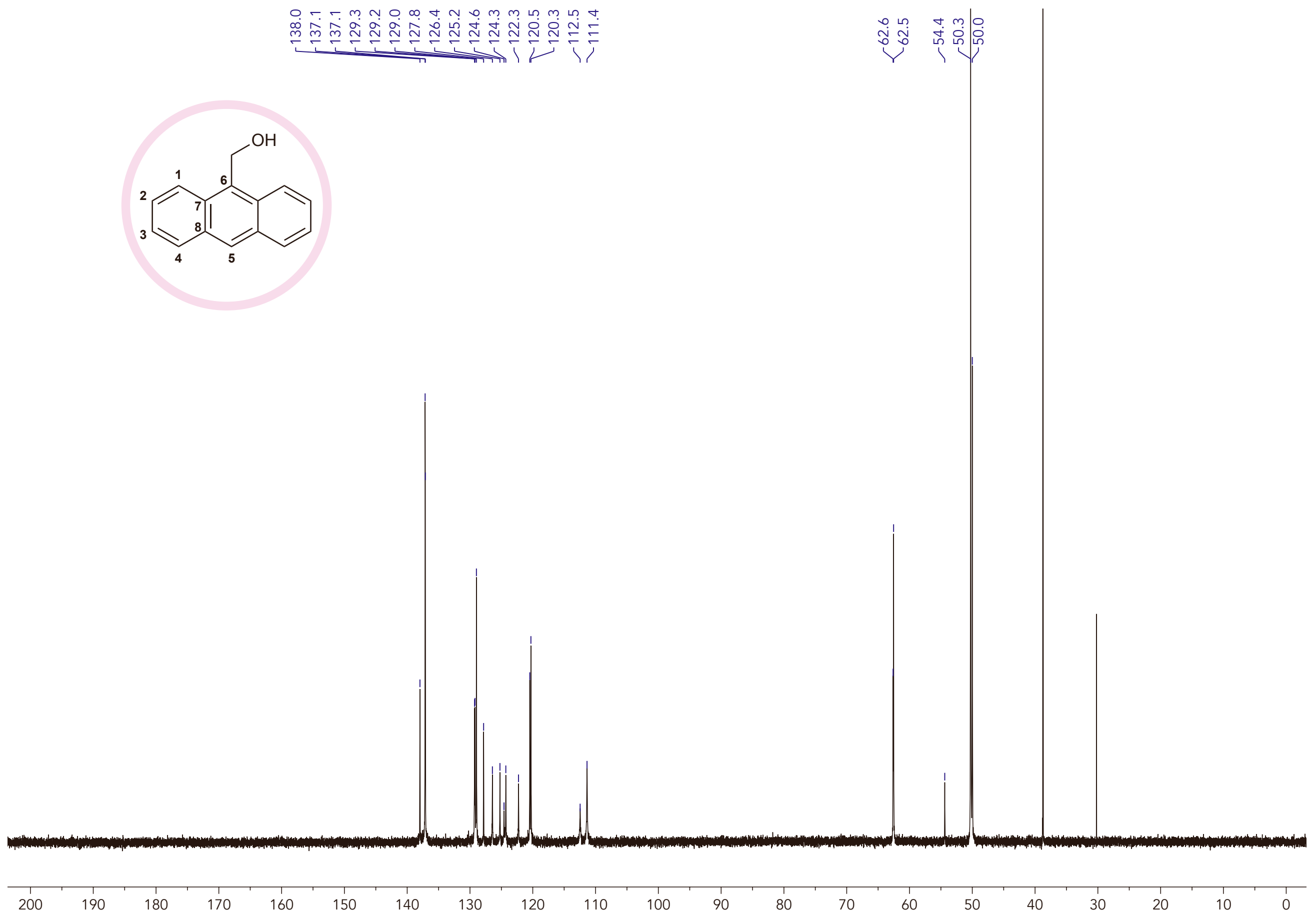

Figure S40. ^{13}C NMR spectrum of ($\mathbf{a3}$)$_2$⊂**C** (150 MHz, D_2O, 300 K).

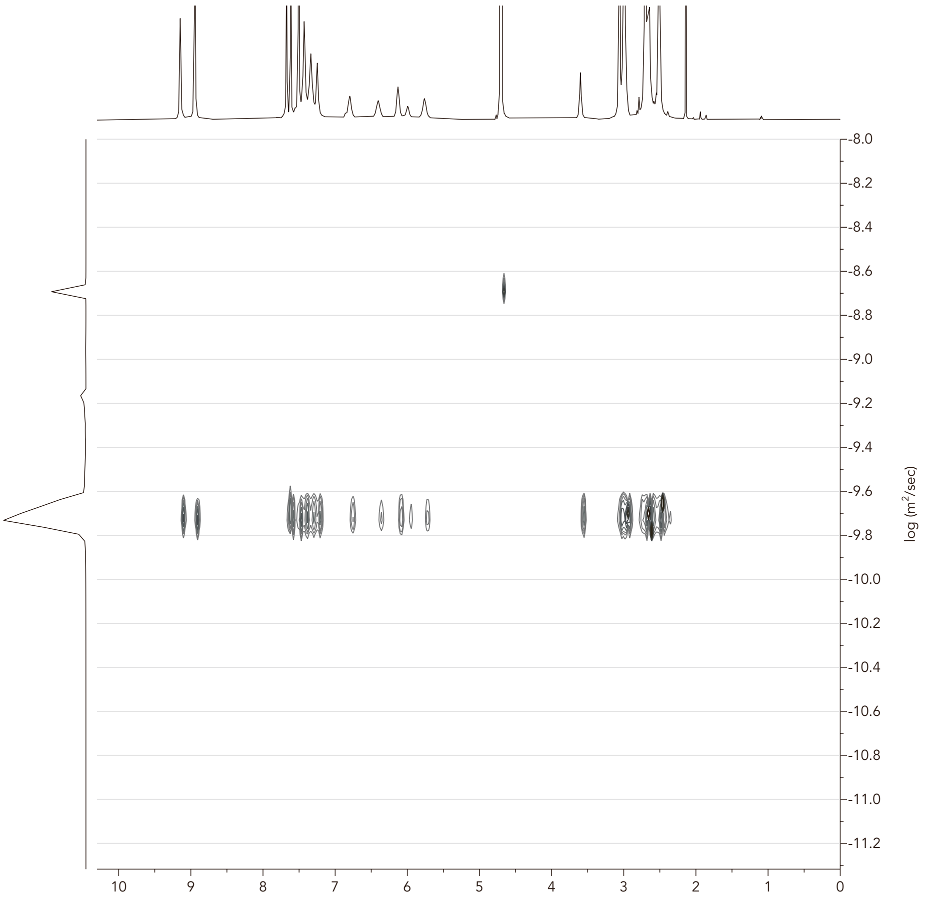

Figure S41. ^{1}H DOSY NMR spectrum of $(\mathbf{a3})_2 \subset \mathbf{C}$ (500 MHz, D_2O, 300 K).

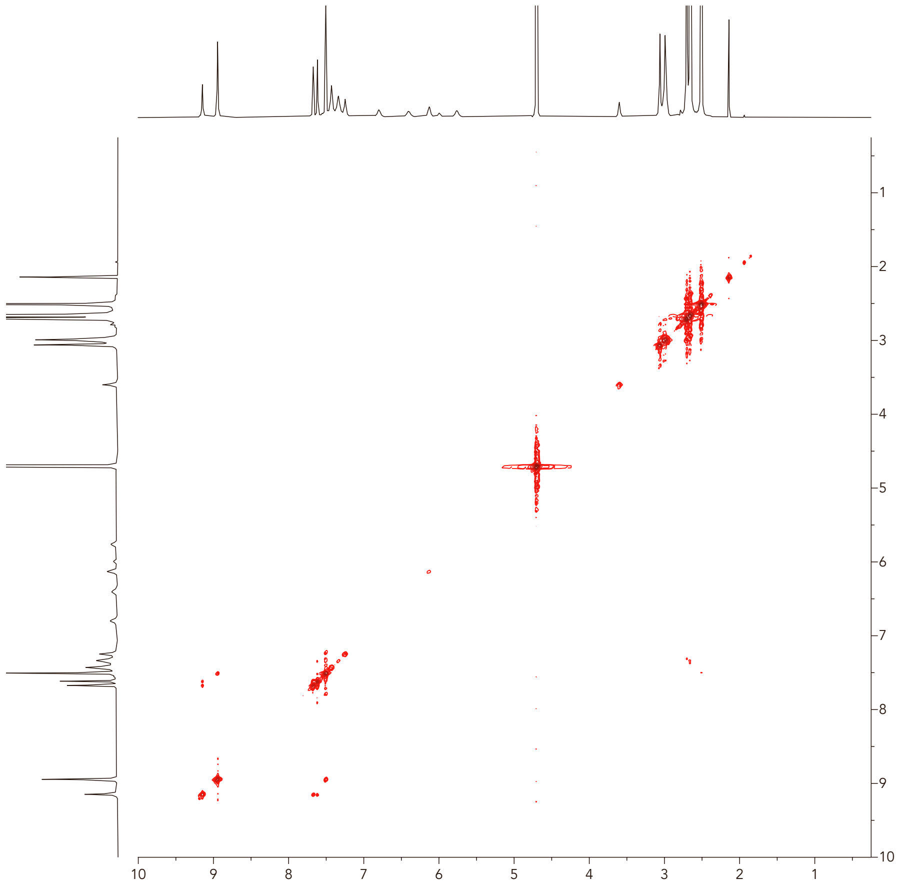

Figure S42. ^{1}H–^{1}H COSY NMR spectrum of (**a3**)$_2$⊂**C** (600 MHz, D_2O, 300 K).

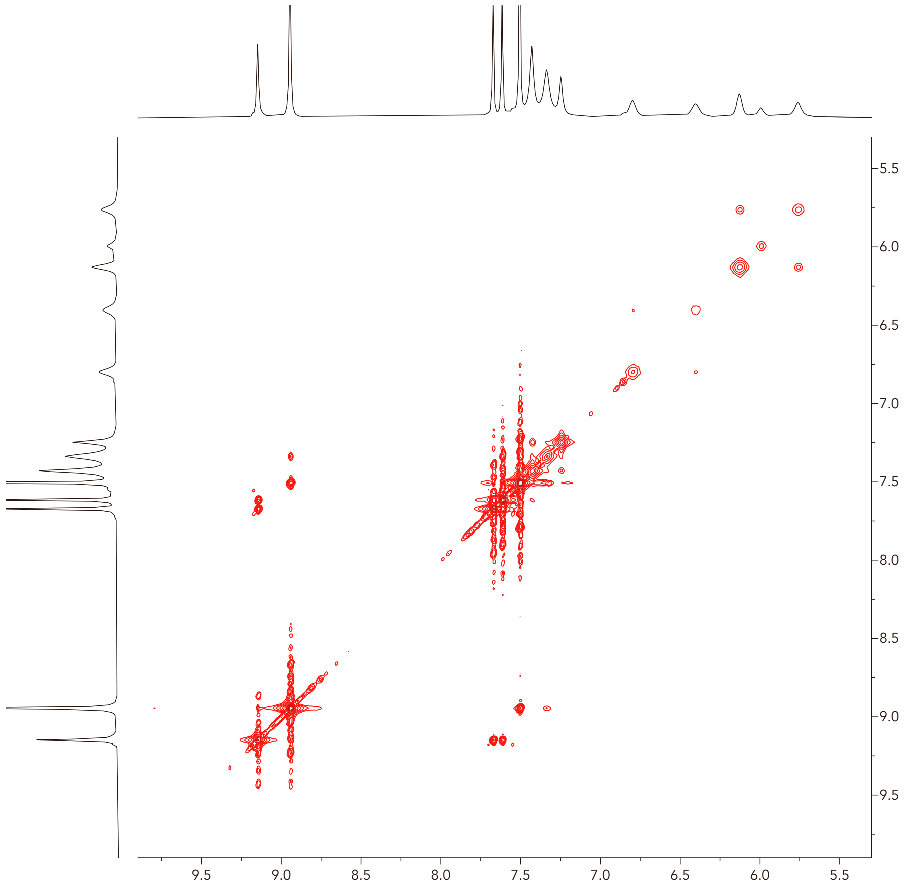

Figure S43. Partial ^{1}H–^{1}H COSY NMR spectrum of (**a3**)$_2$⊂**C** (600 MHz, D_2O, 300 K).

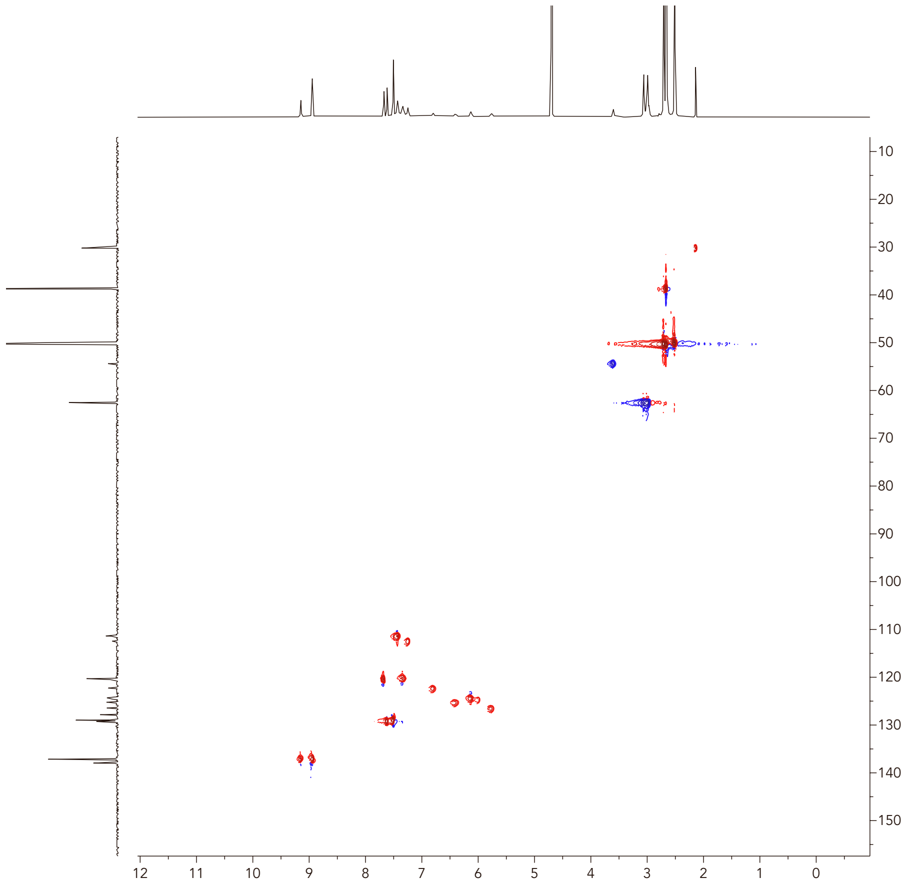

Figure S44. ^1H–^{13}C HSQC NMR spectrum of (**a3**)$_2$⊂**C** (600 MHz, D_2O, 300 K).

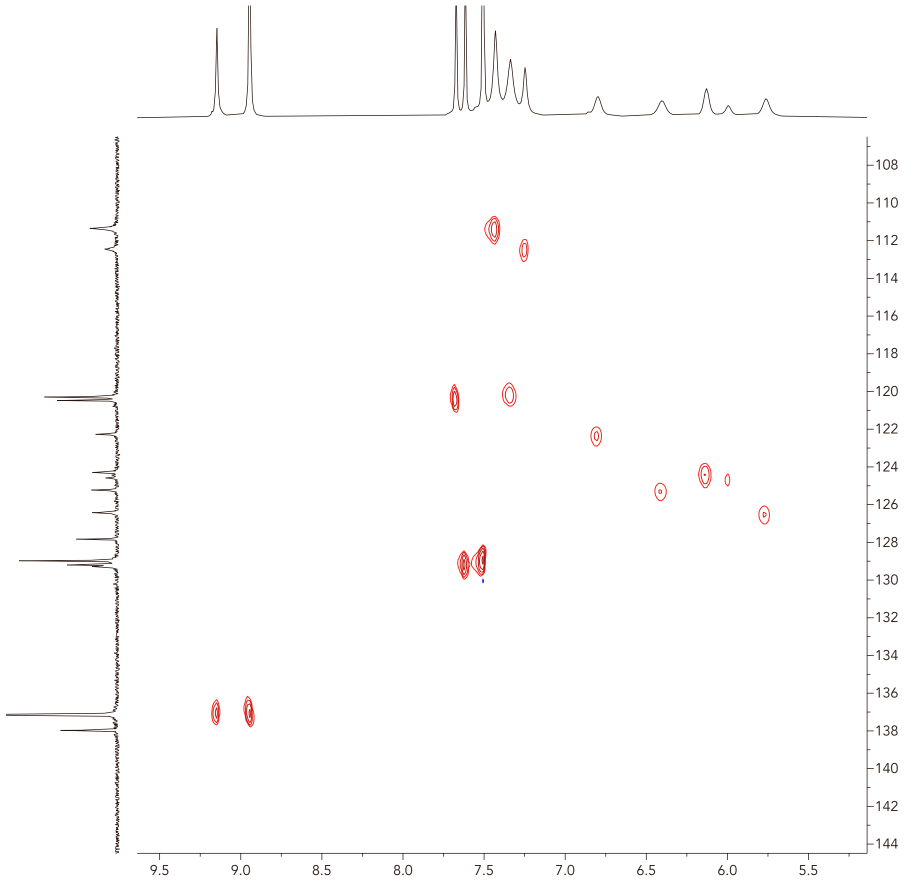

Figure S45. Partial ^{1}H–^{13}C HSQC NMR spectrum of (**a3**)$_2$⊂**C** (600 MHz, D_2O, 300 K).

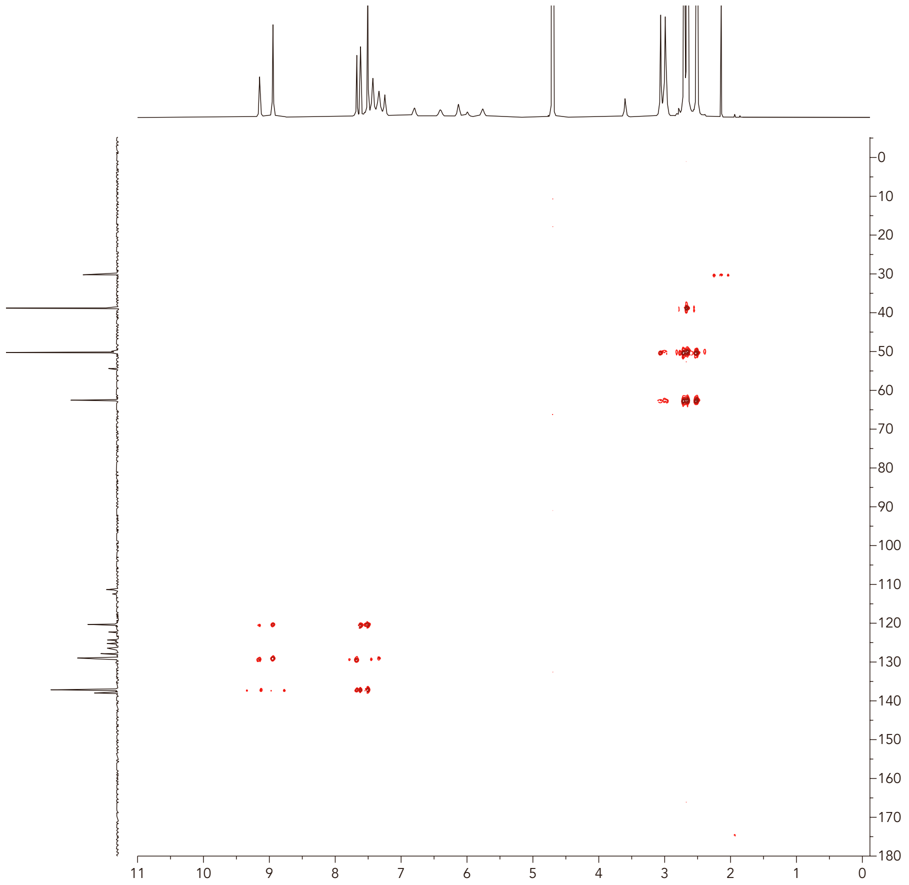

Figure S46. ^{1}H–^{13}C HMBC NMR spectrum of (**a3**)$_2$⊂**C** (600 MHz, D_2O, 300 K).

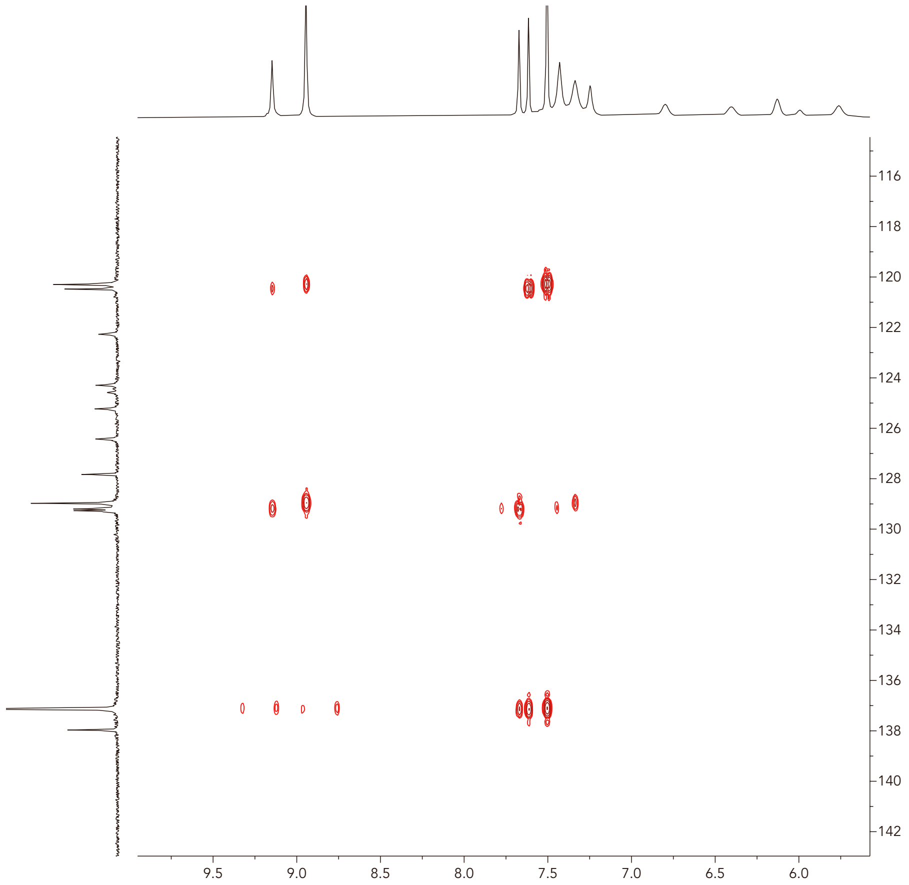

Figure S47. Partial ^{1}H–^{13}C HMBC NMR spectrum of (**a3**)$_2$⊂**C** (600 MHz, D_2O, 300 K).

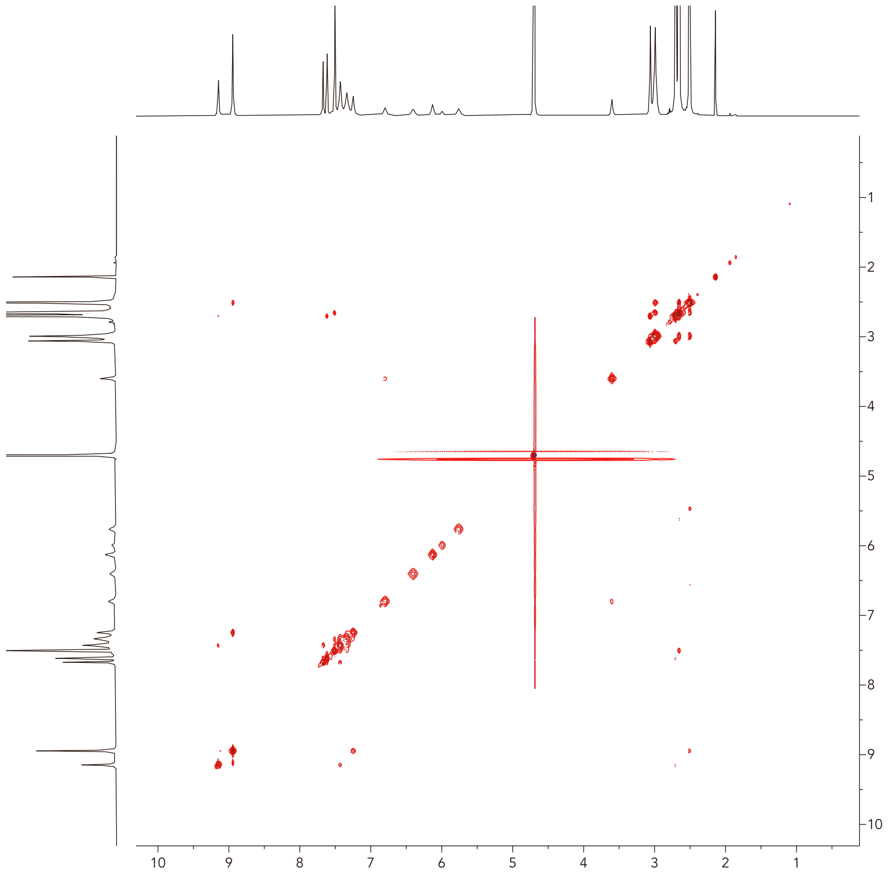

Figure S48. ¹H–¹H NOESY NMR spectrum of (**a3**)$_2$⊂**C** (600 MHz, D_2O, 300 K).

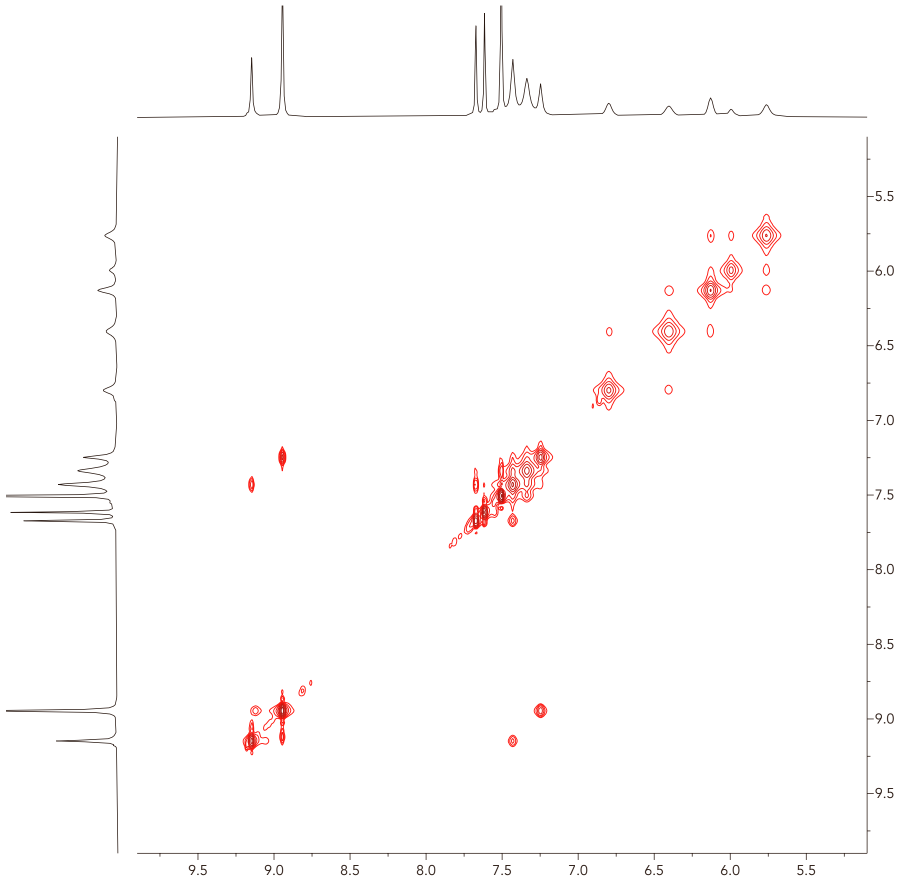

Figure S49. Partial ^{1}H–^{1}H NOESY NMR spectrum of **(a3)**$_2$⊂**C** (600 MHz, D_2O, 300 K).

Inclusion complex $(\mathbf{a4})_2\subset\mathbf{C}$ was obtained in a near-quantitative yield, as determined by ^{1}H NMR spectroscopy.

^{1}H NMR (600 MHz, D_2O, 340 K): δ = 9.30 (s, 4H, $\mathbf{C_1}$), 8.78 (s, 8H, $\mathbf{C_4}$), 7.82 (br, 8H, $\mathbf{C_{3+2}}$), 7.58 (s, 8H, $\mathbf{C_5}$), 7.30 (s, 16H, $\mathbf{C_{6+7}}$), 6.90 (br, 4H, $\mathbf{C_8}$), 6.81 (s, 4H, $\mathbf{a4_2}$), 6.46 (s, 8H, $\mathbf{a4_1}$), 5.74 (s, 8H, $\mathbf{a4_3}$), 3.14 (s, 8H, $\mathbf{C_9}$), 3.00 (s, 16H, $\mathbf{C_9}$), 2.84–2.44 (m, 72H, $\mathbf{C_{10}}$).

^{13}C NMR (150 MHz, D_2O, 340 K): δ = 138.0 ($\mathbf{C_{q'}}$), 137.2 ($\mathbf{C_4}$), 137.0 ($\mathbf{C_{1+q}}$), 130.2 ($\mathbf{C_2}$), 129.4 ($\mathbf{C_5}$), 128.9 ($\mathbf{a4_4}$), 125.2 ($\mathbf{a4_2}$), 124.8 ($\mathbf{a4_3}$), 123.9 ($\mathbf{a4_1}$), 122.5 ($\mathbf{a4_5}$), 120.8 ($\mathbf{C_3}$), 120.7 ($\mathbf{C_6}$), 111.9 ($\mathbf{C_8}$), 110.4 ($\mathbf{C_7}$), 63.3 ($\mathbf{C_9}$), 63.2 ($\mathbf{C_9}$), 50.9 ($\mathbf{C_{10}}$), 50.9 ($\mathbf{C_{10}}$), 50.5 ($\mathbf{C_{10}}$). (Note: $\mathbf{C_q}$ and $\mathbf{C_{q'}}$ denote H-free C atoms connected to the axial and equatorial imidazoles, respectively.)

^{1}H DOSY NMR (500 MHz, D_2O, 298 K): $D = 0.15\cdot10^{-5}$ cm^2/s.

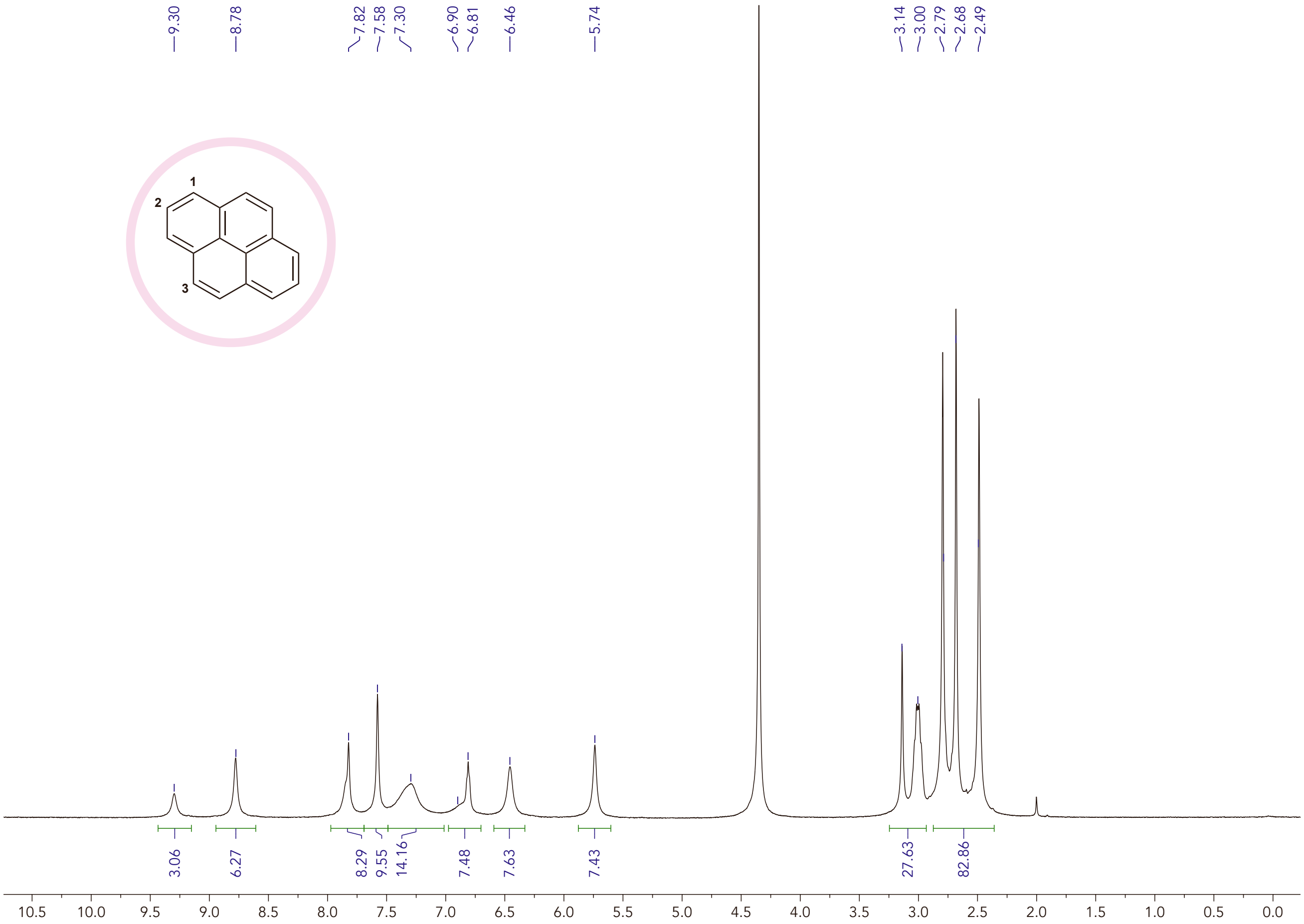

Figure S50. ^{1}H NMR spectrum of $(\mathbf{a4})_2\subset\mathbf{C}$ (600 MHz, D_2O, 340 K).

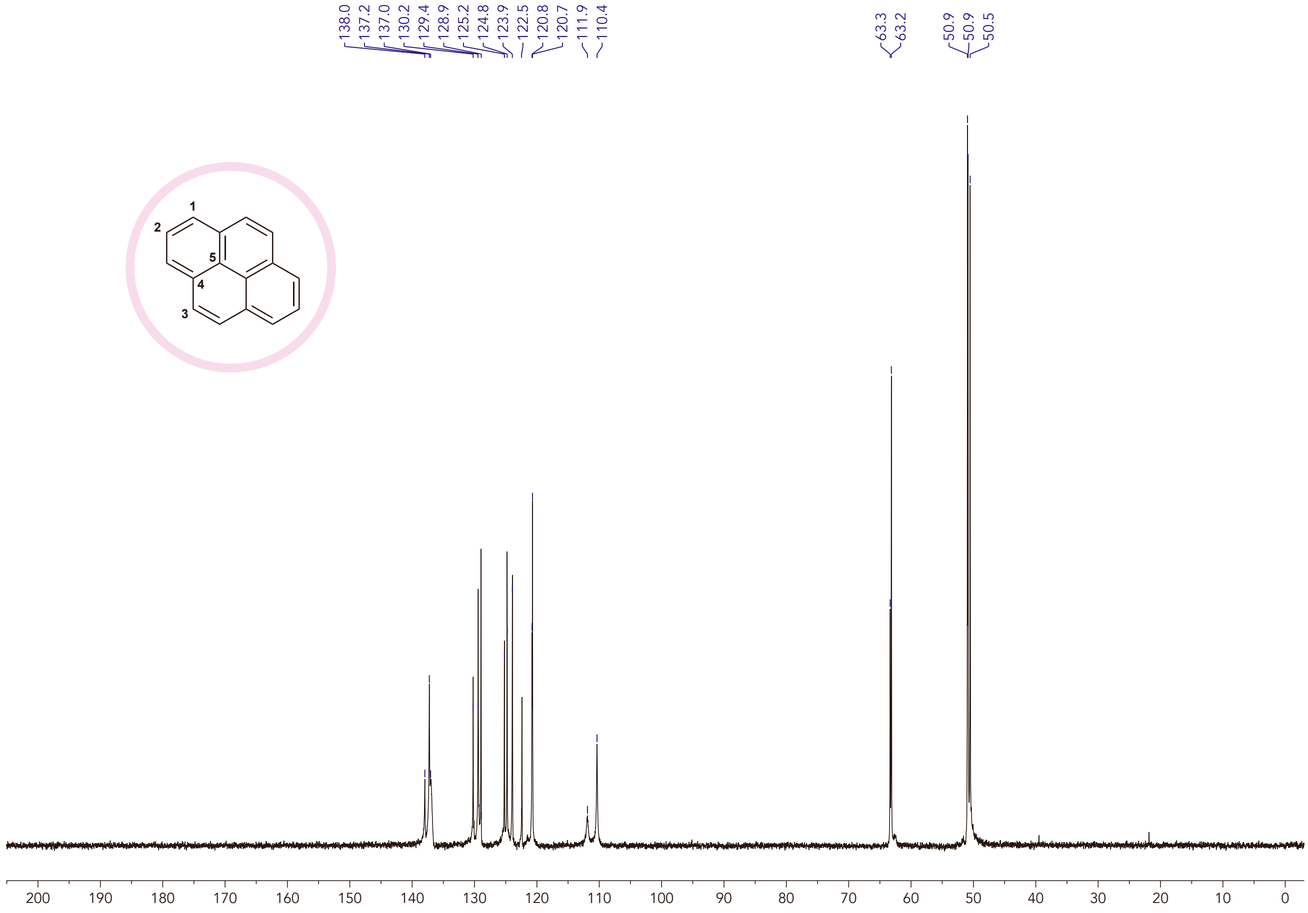

Figure S51. ^{13}C NMR spectrum of (**a4**)$_2$⊂**C** (150 MHz, D_2O, 340 K).

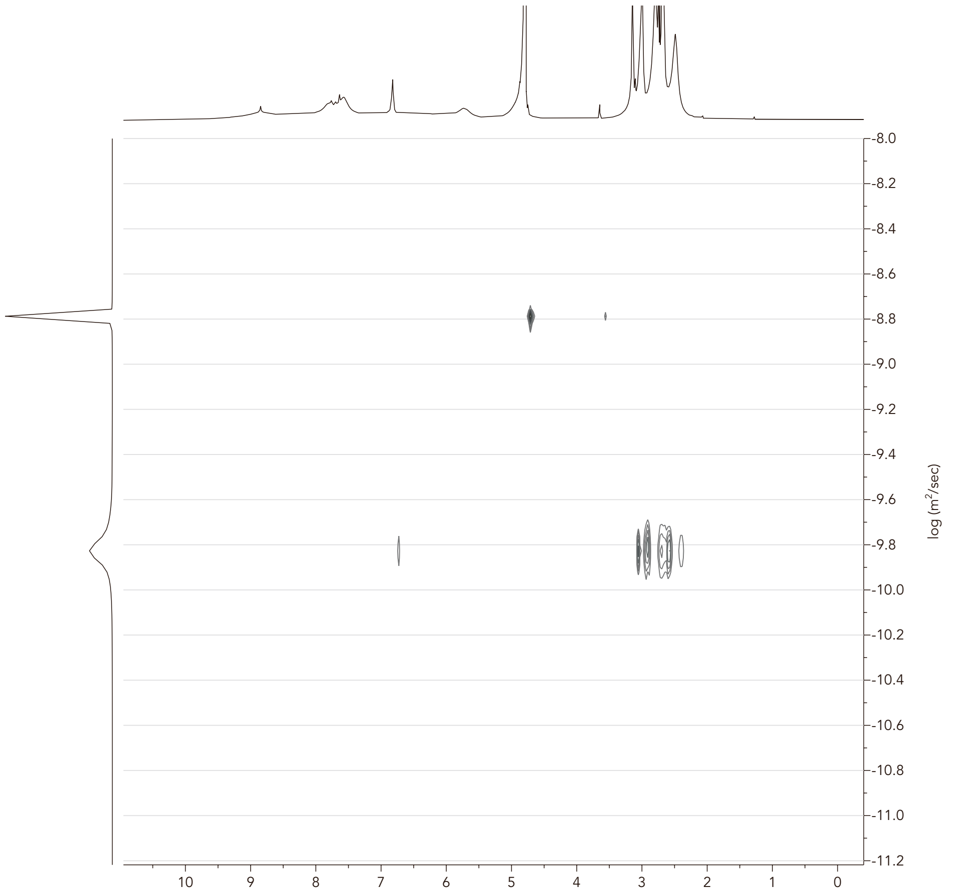

Figure S52. ^{1}H DOSY NMR spectrum of (**a4**)$_2$⊂**C** (500 MHz, D_2O, 298 K). Note that the guest peaks are hardly visible due to the significant signals broadening at room temperature.

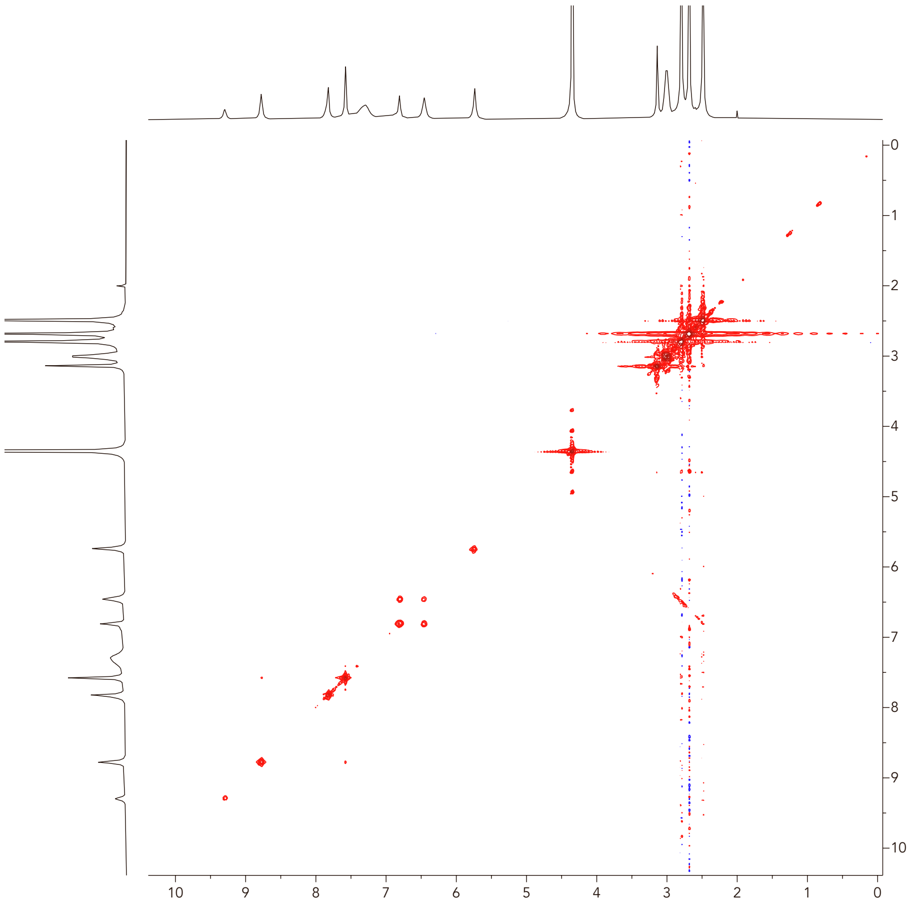

Figure S53. ^{1}H–^{1}H COSY NMR spectrum of (**a4**)$_2$⊂**C** (600 MHz, D_2O, 340 K).

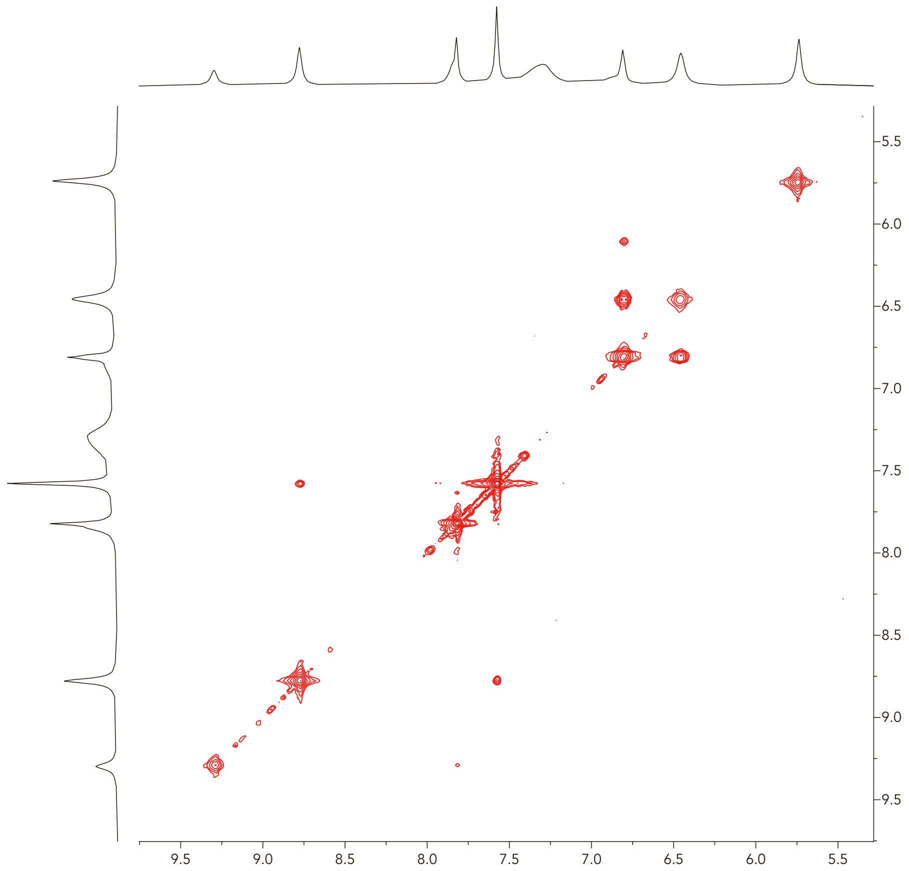

Figure S54. Partial ^{1}H–^{1}H COSY NMR spectrum of (**a4**)$_2$⊂**C** (600 MHz, D_2O, 340 K).

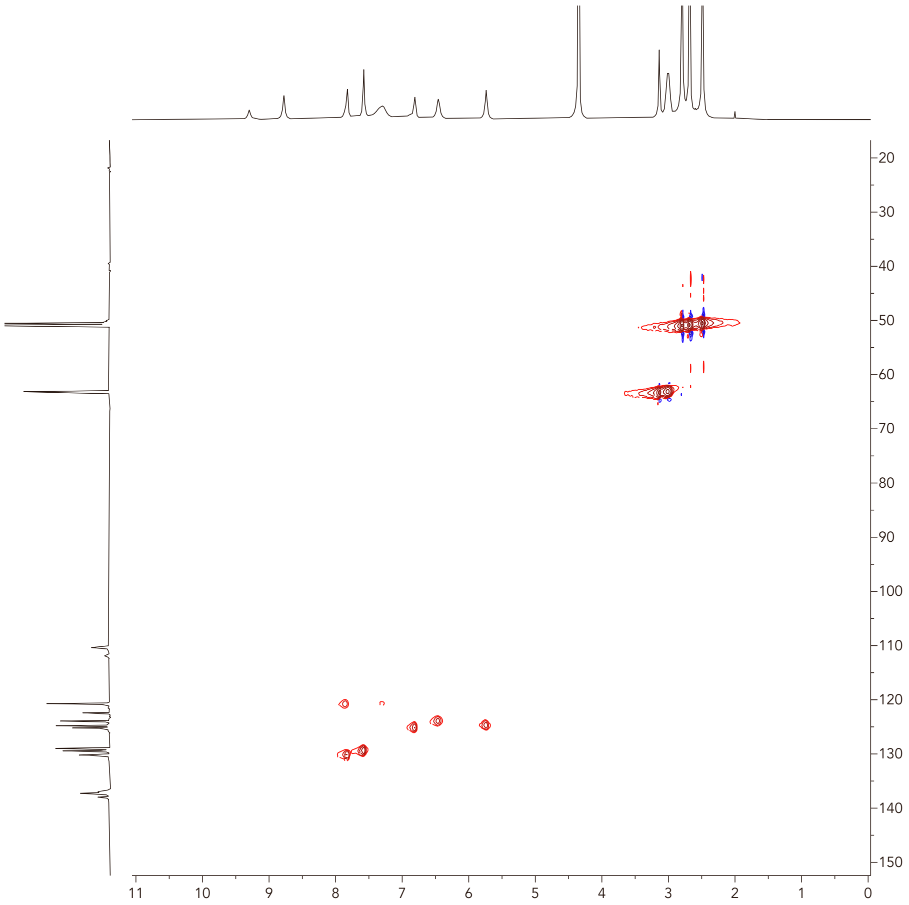

Figure S55. ^{1}H–^{13}C HSQC NMR spectrum of $(\mathbf{a4})_2{\subset}\mathbf{C}$ (600 MHz, D_2O, 340 K).

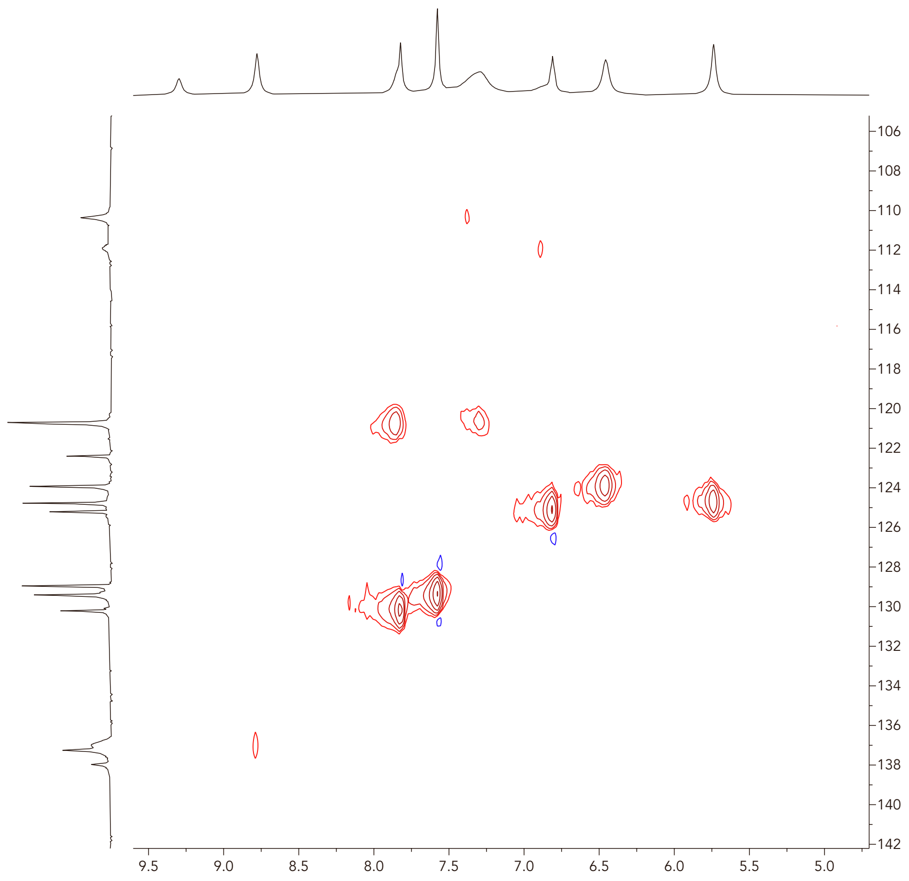

Figure S56. Partial ^{1}H–^{13}C HSQC NMR spectrum of (**a4**)$_2$⊂**C** (600 MHz, D_2O, 340 K).

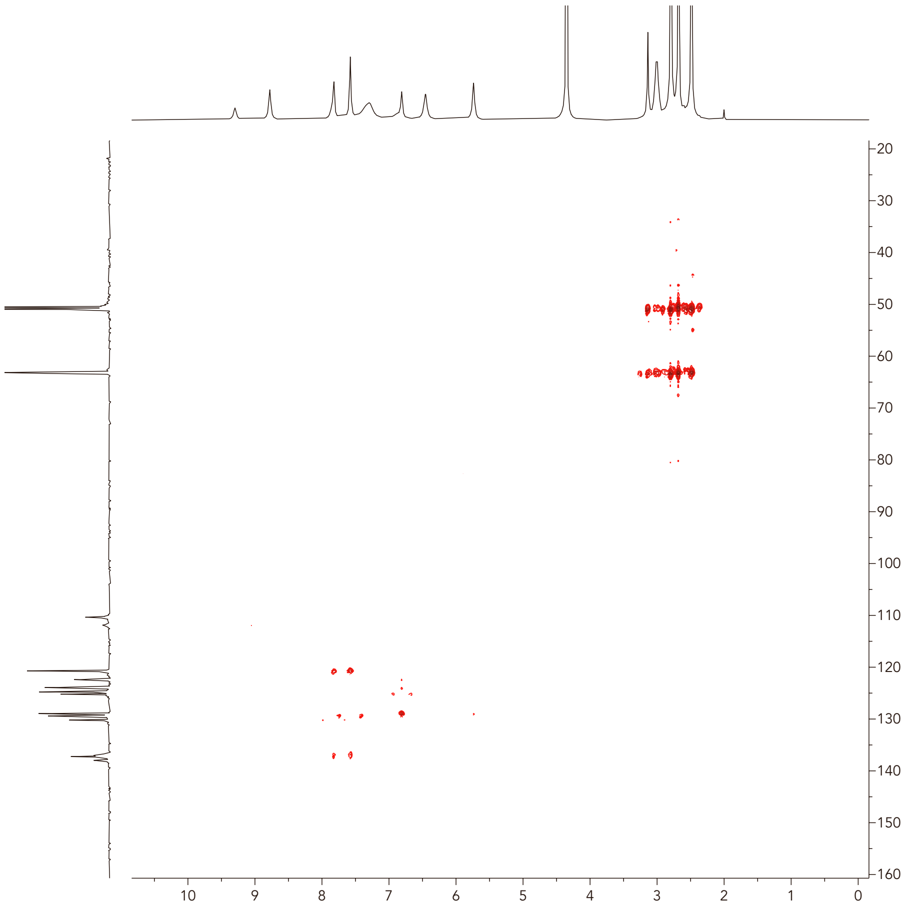

Figure S57. ^{1}H–^{13}C HMBC NMR spectrum of (**a4**)$_2$⊂**C** (600 MHz, D_2O, 340 K).

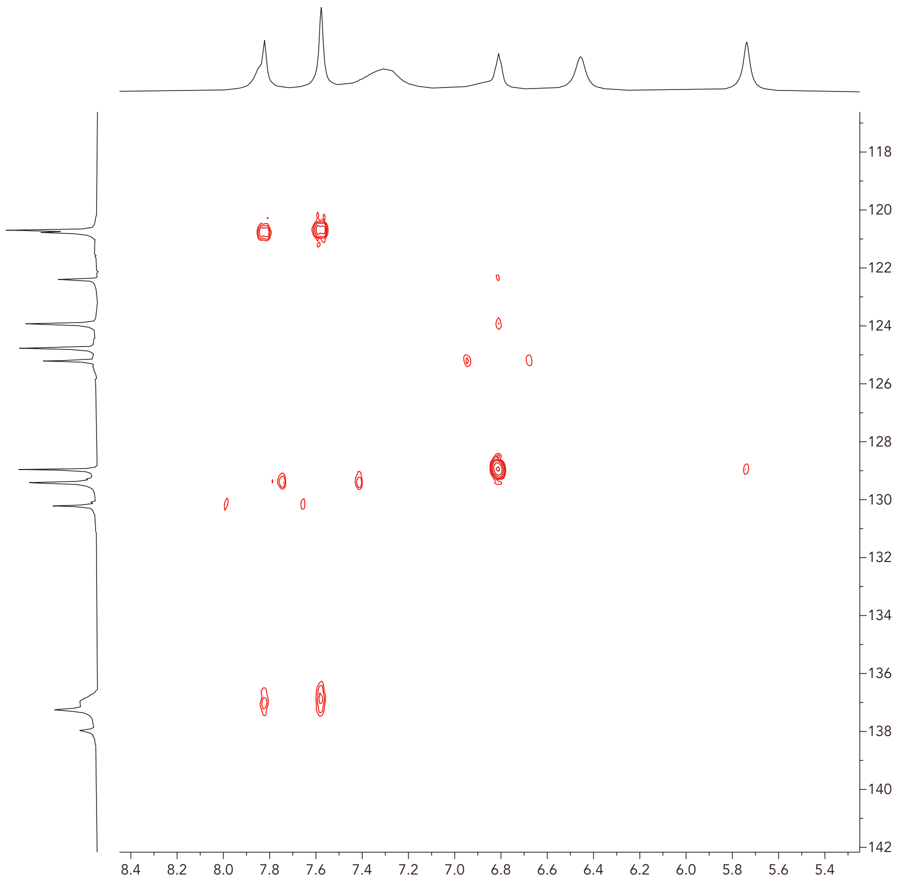

Figure S58. Partial ^{1}H–^{13}C HMBC NMR spectrum of (**a4**)$_2$⊂**C** (600 MHz, D_2O, 340 K).

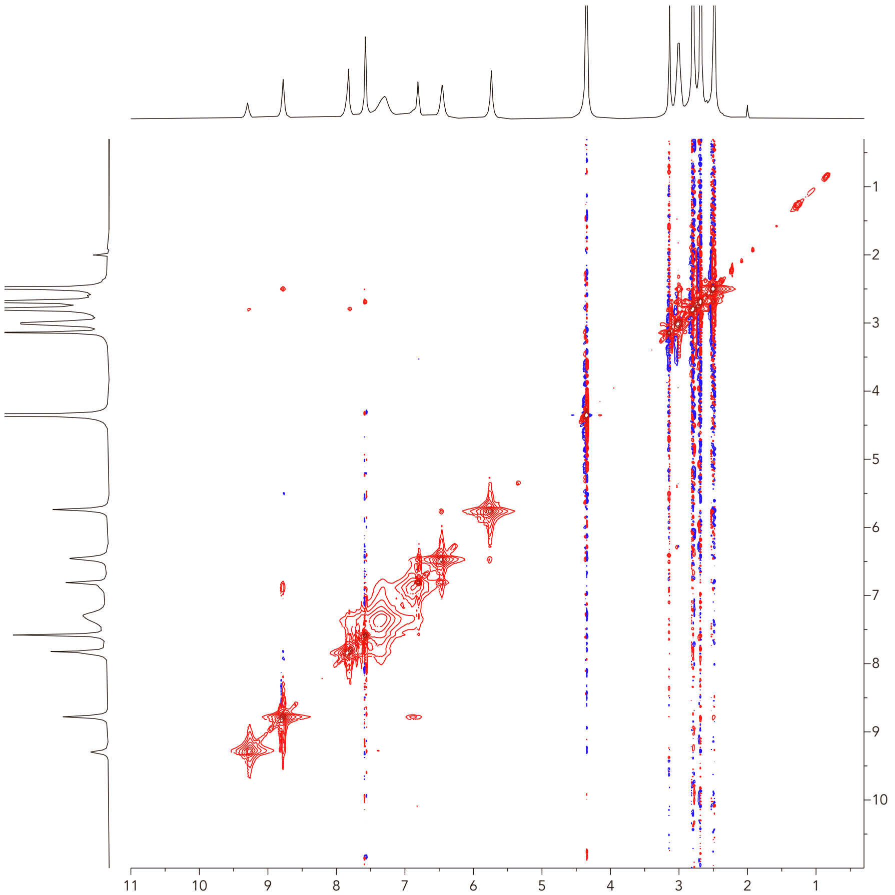

Figure S59. ${}^{1}H$–${}^{1}H$ NOESY NMR spectrum of (**a4**)$_2$⊂**C** (600 MHz, D_2O, 340 K).

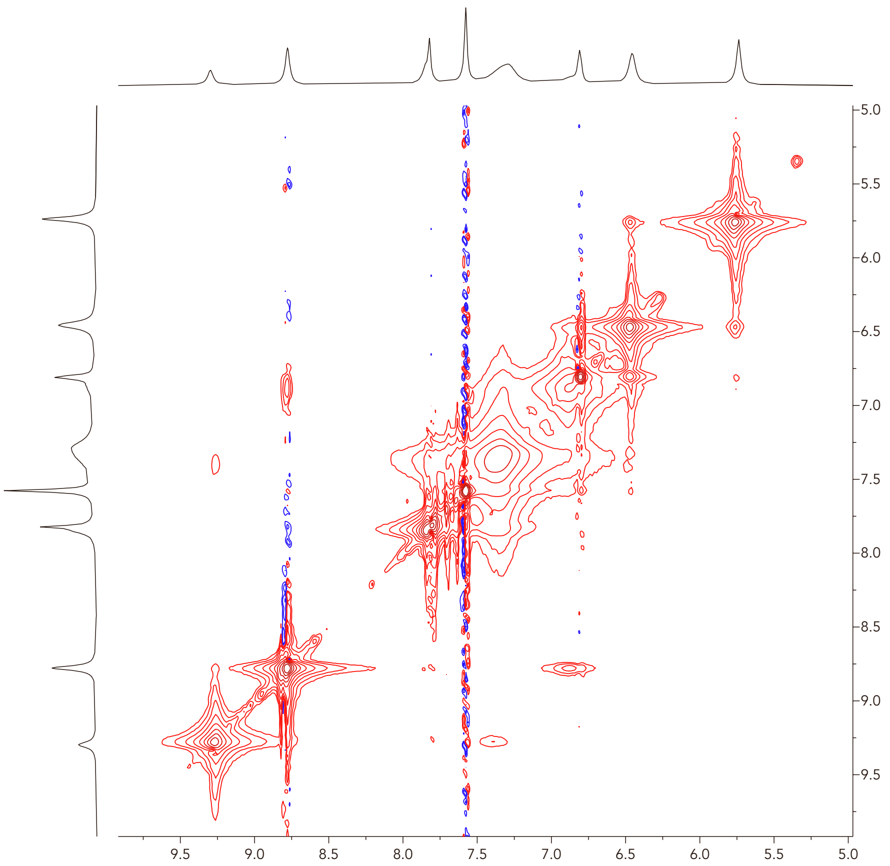

Figure S60. Partial ^{1}H–^{1}H NOESY NMR spectrum of (**a4**)$_2$⊂**C** (600 MHz, D_2O, 340 K).

6. NMR characterization of heterodimers (**a1·b1**)⊂**C**, (**a2·b1**)⊂**C**, (**a1·b2**)⊂**C**, and (**a4·b4**)⊂**C**

Heterodimeric inclusion complex (**a1·b1**)⊂**C** was obtained upon mixing homodimers (**a1**)$_2$⊂**C** and (**b1**)$_2$⊂**C** in a 1:1 ratio (with respect to the cage; note: the encapsulation yield for (**b1**)$_2$⊂**C** is ~50%, thus ~25% of the cage present in the system is unoccupied). The coexistence of the heterodimer with the two homodimers and free cage resulted in a complex NMR spectrum; consequently, only characteristic peaks originating from the guests are listed below (note: guest protons with an apostrophe denote guests encapsulated within heterodimer (**a1·b1**)⊂**C**; those without an apostrophe refer to guests within homodimers (**a1**)$_2$⊂**C** and (**b1**)$_2$⊂**C**[2]):

^{1}H NMR (600 MHz, D_2O, 320 K); guest peaks: δ = 5.59 (s, **b1**$_4$), 5.32 (s, **b1**$_{4'}$), 1.83−1.80 (s, **b1**$_1$+**b1**$_{1'}$), 0.97 (s, **a1**$_{CH_3'}$), 0.65 (s, **b1**$_2$), 0.31 (s, **b1**$_{2'}$), 0.27 (s, **b1**$_3$), 0.13 (s, **a1**$_{CH_3}$), –0.62 (s, **b1**$_{3'}$).

^{1}H DOSY NMR (500 MHz, D_2O, 300 K): $D = 0.22\cdot10^{-5}$ cm^2/s.

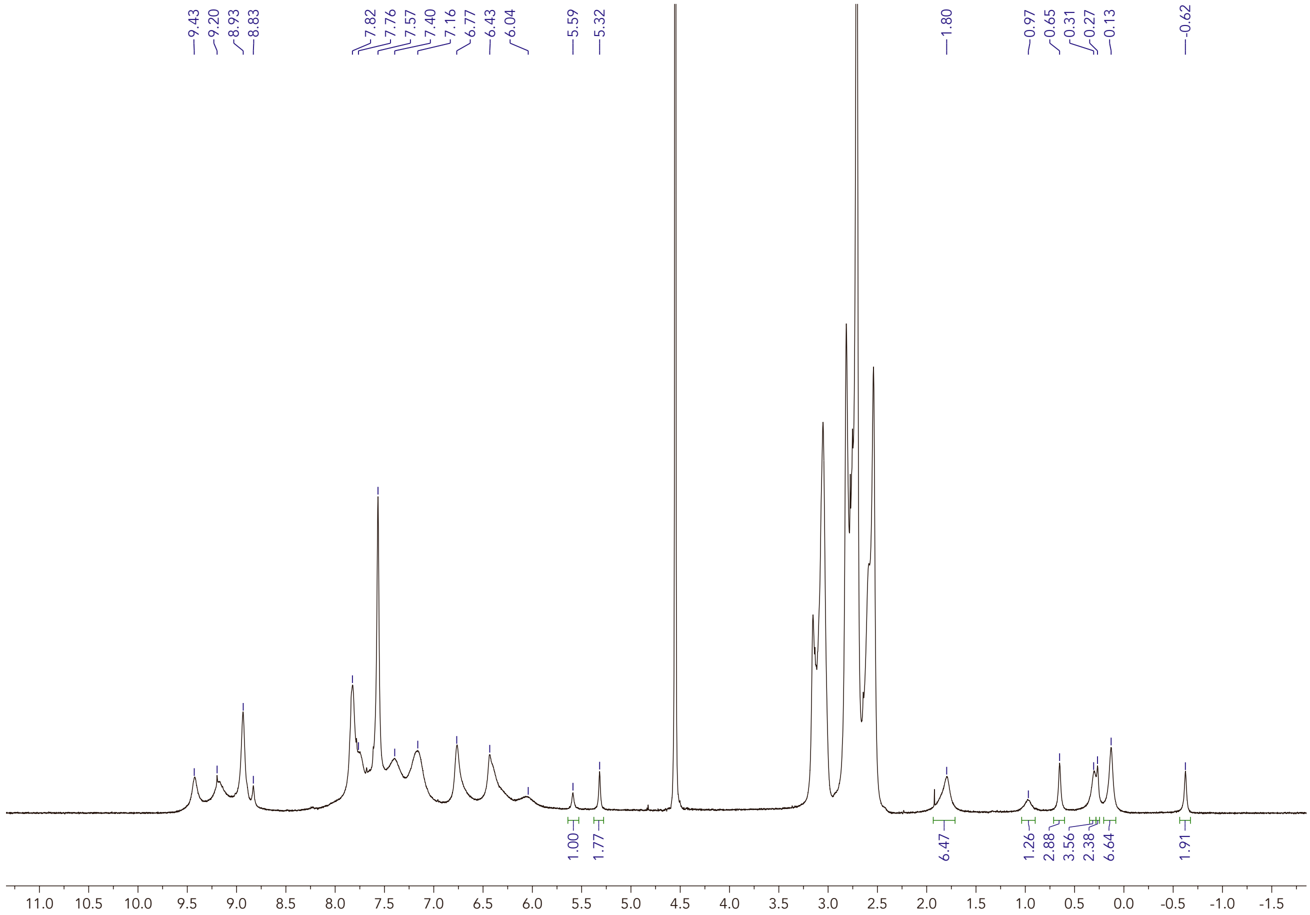

Figure S61. ^{1}H NMR spectrum of (**a1·b1**)⊂**C** (in the presence of (**a1**)$_2$⊂**C**, (**b1**)$_2$⊂**C**, and free **C**) (600 MHz, D_2O, 320 K).

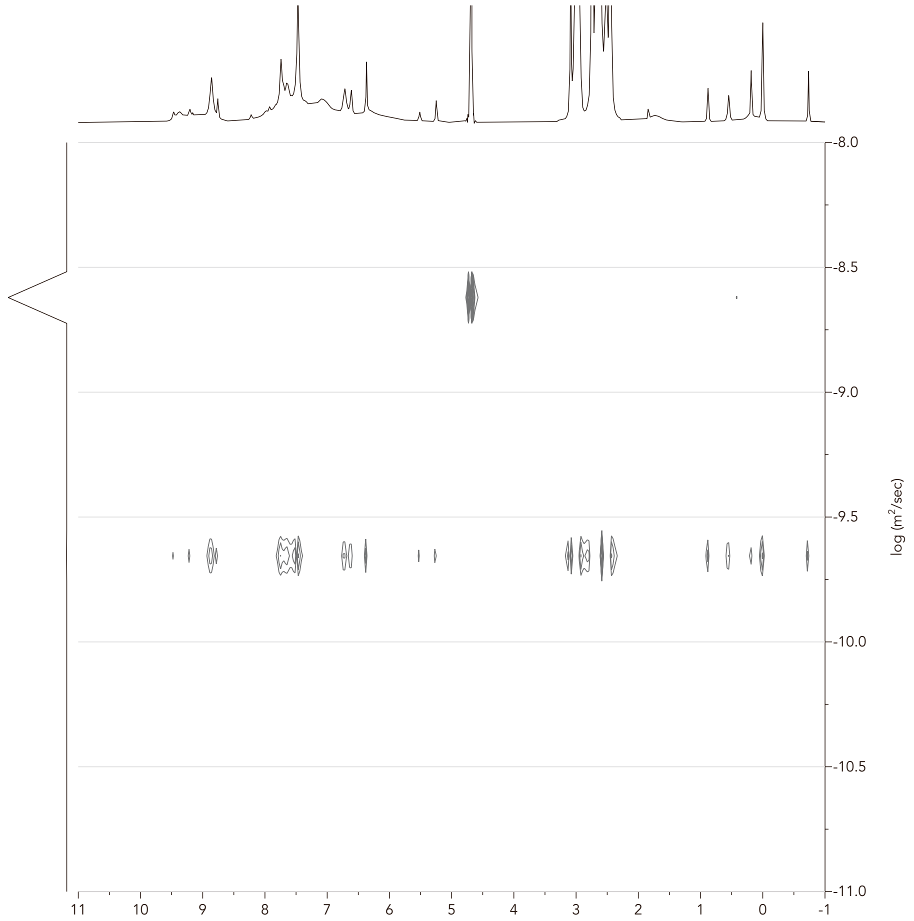

Figure S62. ^{1}H DOSY NMR spectrum of (**a1**·**b1**)⊂**C** (in the presence of (**a1**)$_2$⊂**C**, (**b1**)$_2$⊂**C**, and free **C**) (500 MHz, D_2O, 300 K).

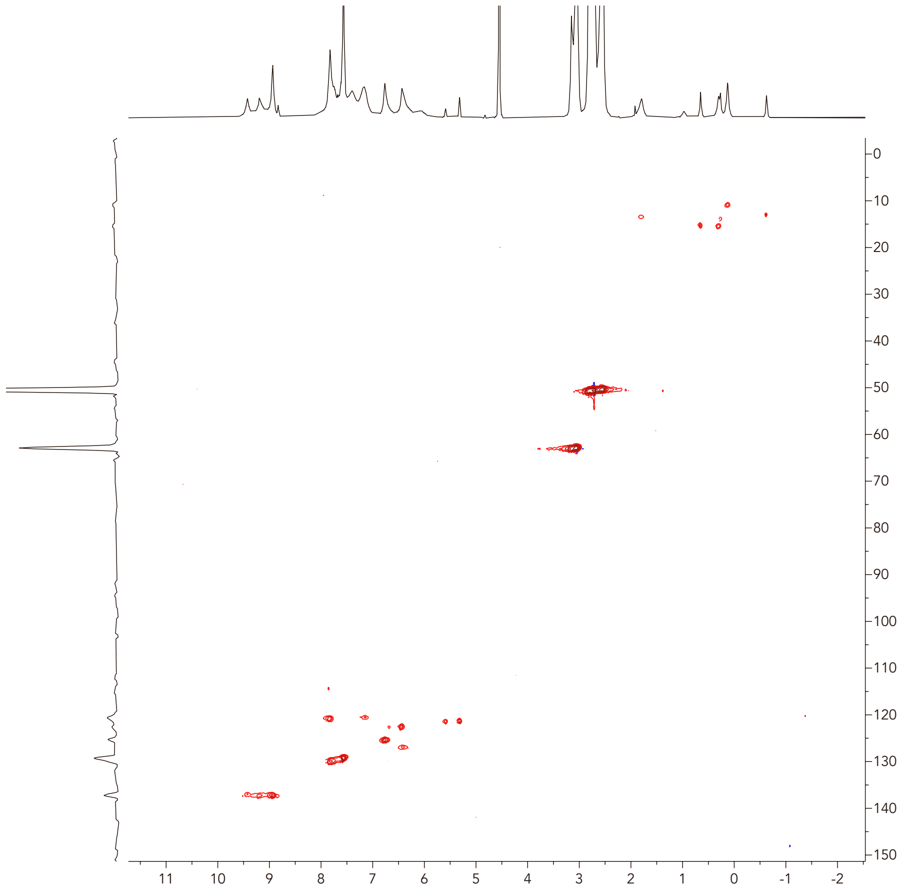

Figure S63. ^{1}H–^{13}C HSQC NMR spectrum of (**a1**·**b1**)⊂**C** (in the presence of (**a1**)$_2$⊂**C**, (**b1**)$_2$⊂**C**, and free **C**) (600 MHz, D_2O, 320 K).

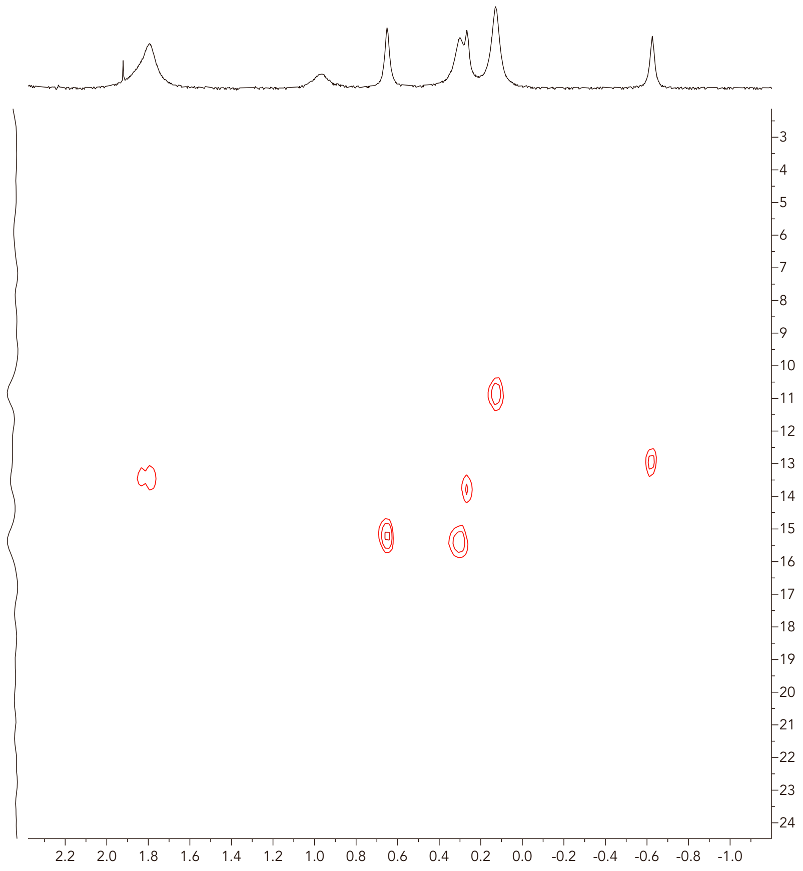

Figure S64. Partial ^{1}H–^{13}C HSQC NMR spectrum of (**a1**·**b1**)⊂**C** (in the presence of (**a1**)$_2$⊂**C**, (**b1**)$_2$⊂**C**, and free **C**) (600 MHz, D_2O, 320 K).

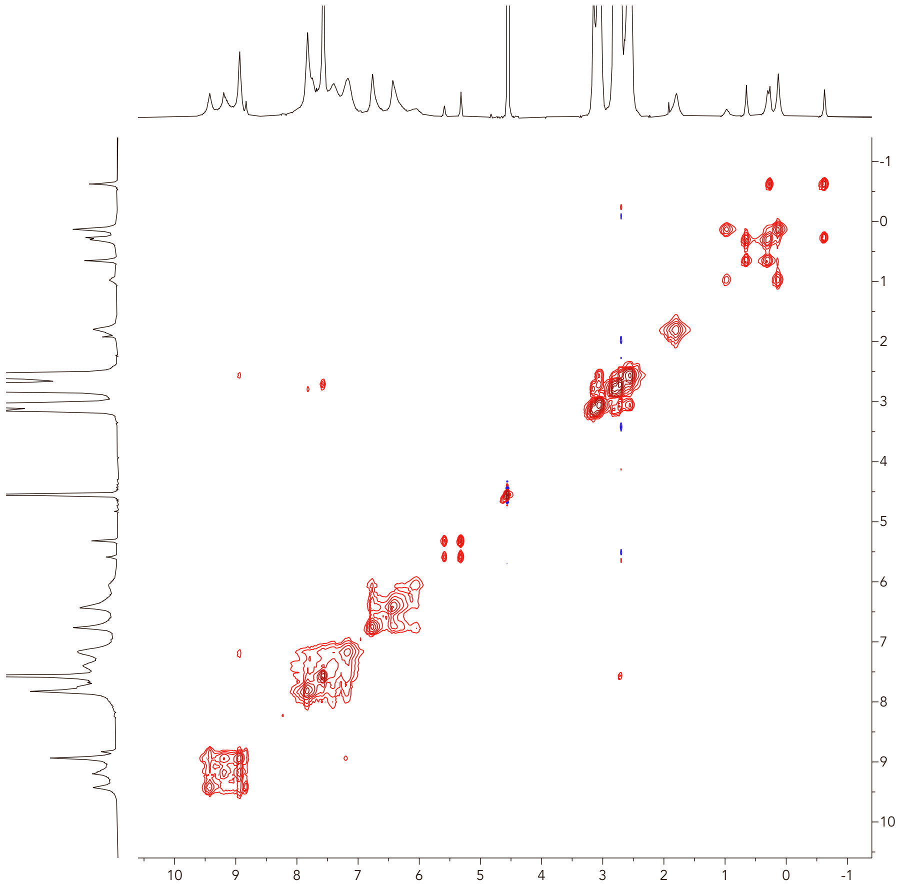

Figure S65. ¹H–¹H NOESY NMR spectrum of (**a1**·**b1**)⊂**C** (in the presence of (**a1**)$_2$⊂**C**, (**b1**)$_2$⊂**C**, and free **C**) (600 MHz, D_2O, 320 K).

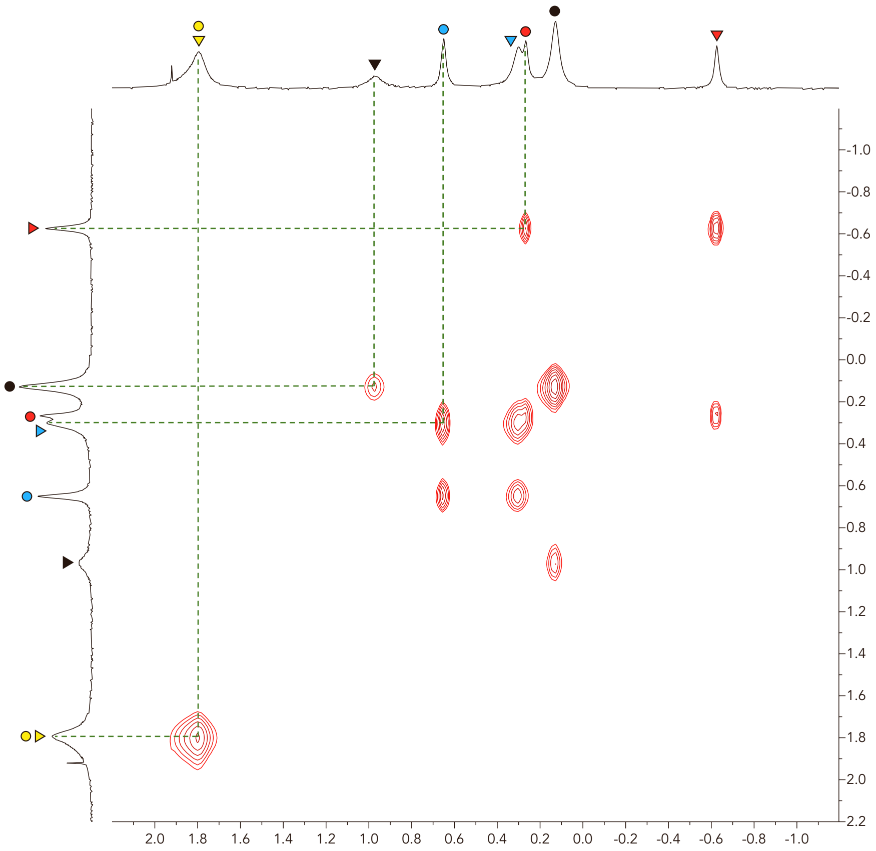

Figure S66. Partial ^{1}H–^{1}H EXSY NMR spectrum of (**a1**·**b1**)⊂**C** (in the presence of (**a1**)$_2$⊂**C** and (**b1**)$_2$⊂**C**) (600 MHz, D_2O, 320 K). Peak labels as in Figure 2C of the main text.

Heterodimeric inclusion complex (**a2**·**b1**)⊂**C** was obtained upon mixing homodimers (**a2**)$_2$⊂**C** and (**b1**)$_2$⊂**C** in a 1:1 ratio (with respect to the cage; note: the encapsulation yield for (**b1**)$_2$⊂**C** is ~50%, hence ~25% of the cage present in the system is unoccupied). The coexistence of the heterodimer with the two homodimers resulted in a complex NMR spectrum; consequently, only characteristic peaks originating from the guests are listed below (note: guest protons with an apostrophe denote guests encapsulated within heterodimer (**a2**·**b1**)⊂**C**; those without an apostrophe refer to guests within homodimer (**b1**)$_2$⊂**C** (Ref. 2)):

^{1}H NMR (600 MHz, D_2O, 320 K); guest peaks: δ = 5.59 (s, **b1$_4$**), 5.15 (s, **b1$_{4'}$**), 1.85 (s, **b1$_1$**), 1.75 (s, **b1$_{1'}$**), 0.65 (s, **b1$_2$**), 0.40 (s, **b1$_{2'}$**), 0.26 (s, **b1$_3$**), –0.37 (s, **b1$_{3'}$**).

^{1}H DOSY NMR (500 MHz, D_2O, 300 K): $D = 0.20\cdot10^{-5}$ cm^2/s.

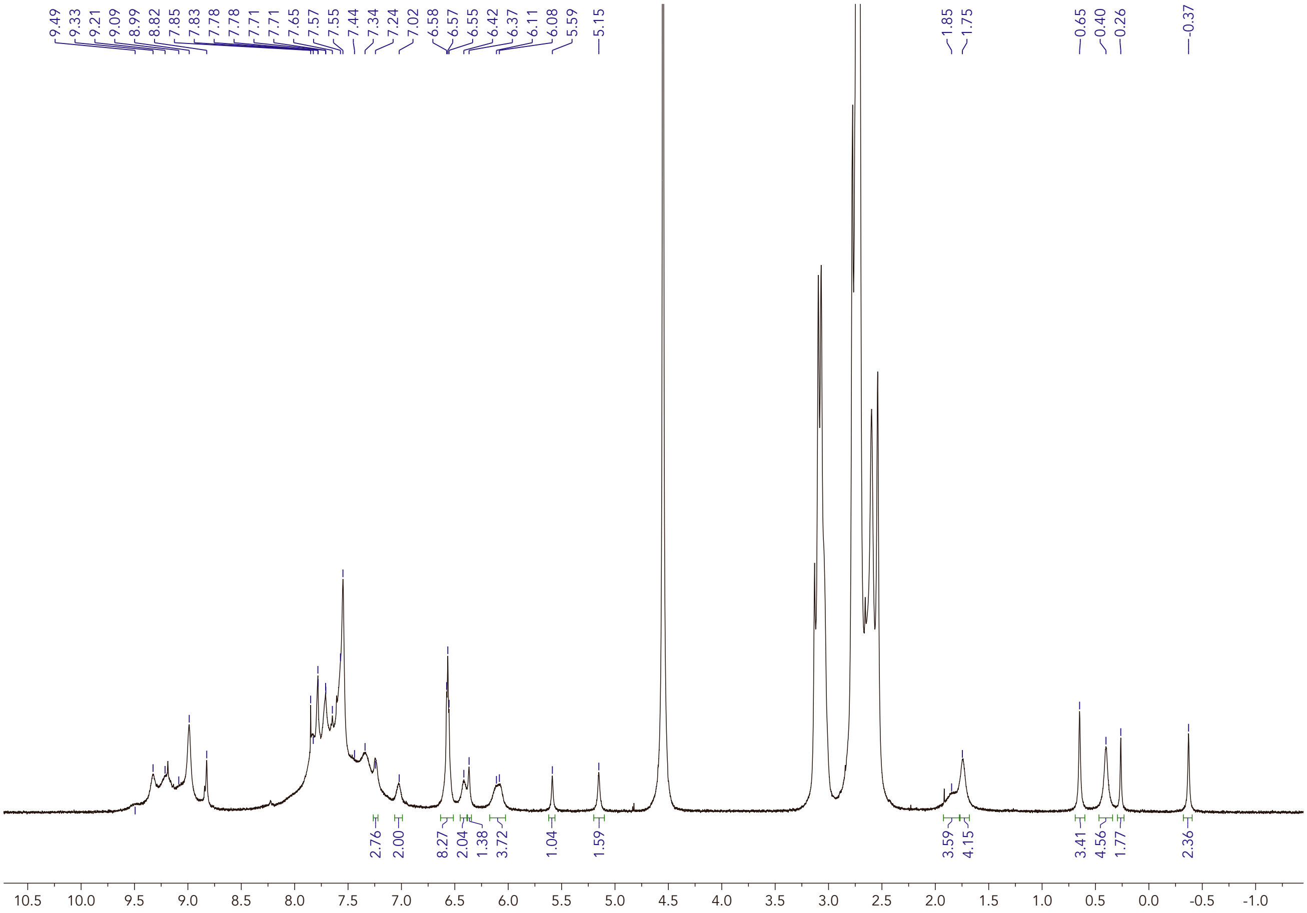

Figure S67. ^{1}H NMR spectrum of (**a2**·**b1**)⊂**C** (in the presence of (**a2**)$_2$⊂**C**, (**b1**)$_2$⊂**C**, and free **C**) (600 MHz, D_2O, 320 K).

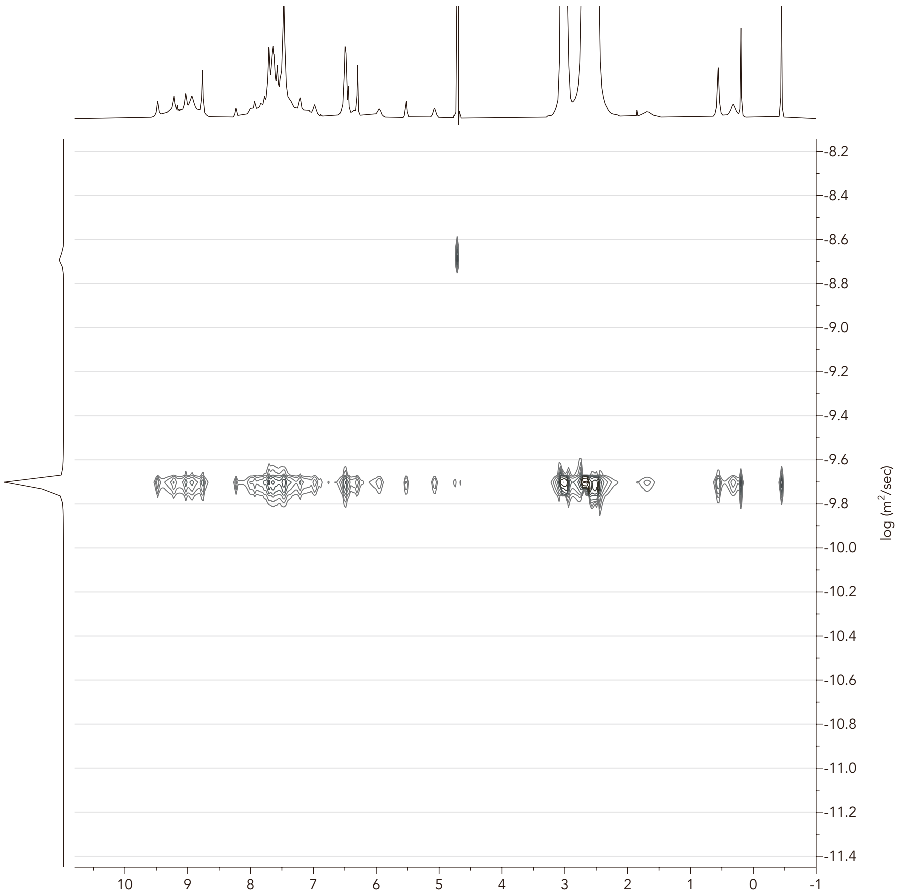

Figure S68. ^{1}H DOSY NMR spectrum of (**a2**·**b1**)⊂**C** (in the presence of (**a2**)$_2$⊂**C**, (**b1**)$_2$⊂**C**, and free **C**) (500 MHz, D_2O, 300 K).

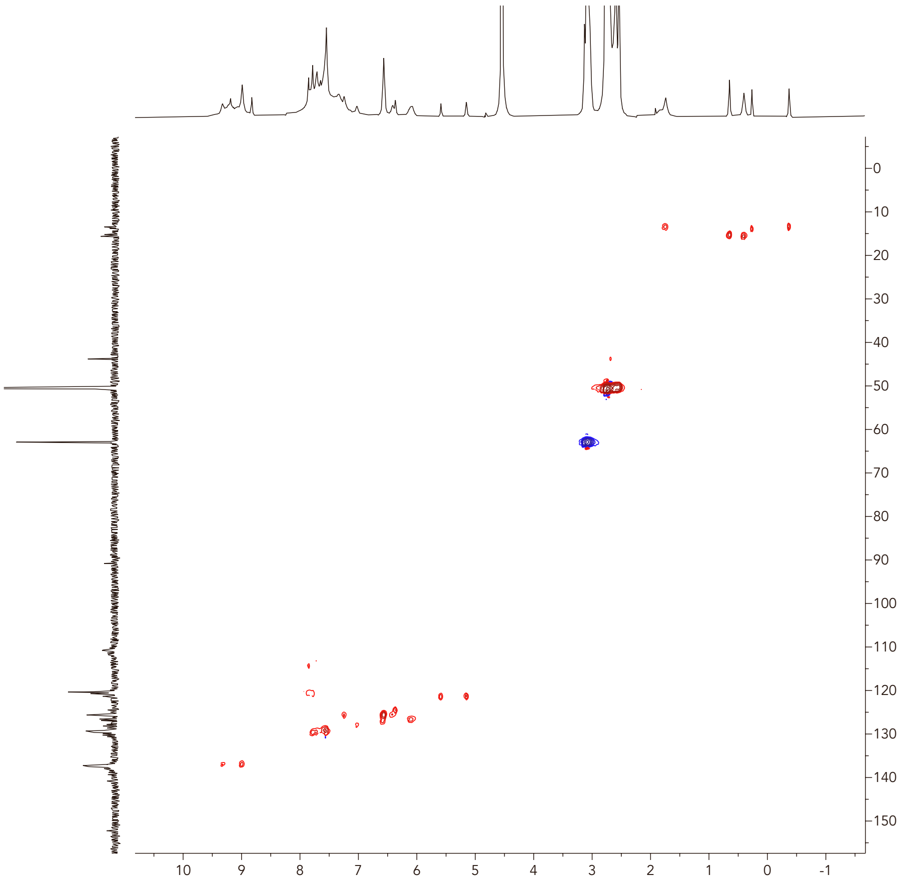

Figure S69. ^{1}H–^{13}C HSQC NMR spectrum of (**a2**·**b1**)⊂**C** (in the presence of (**a2**)$_2$⊂**C**, (**b1**)$_2$⊂**C**, and free **C**) (600 MHz, D_2O, 320 K).

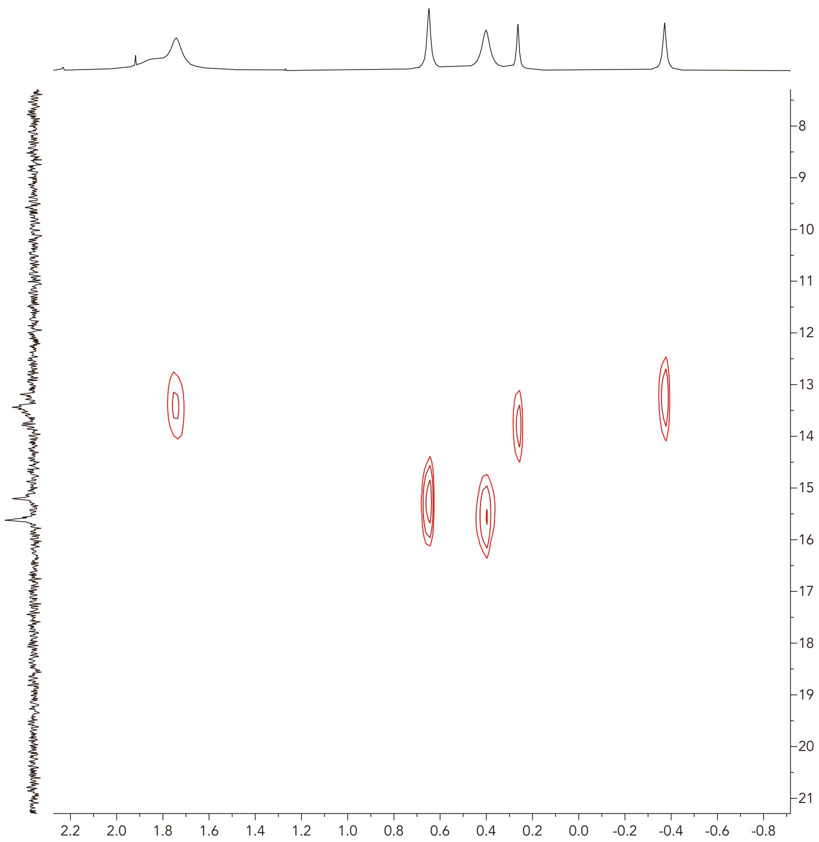

Figure S70. Partial ^{1}H–^{13}C HSQC NMR spectrum of (**a2**·**b1**)⊂**C** (in the presence of (**a2**)$_2$⊂**C**, (**b1**)$_2$⊂**C**, and free **C**) (600 MHz, D_2O, 320 K).

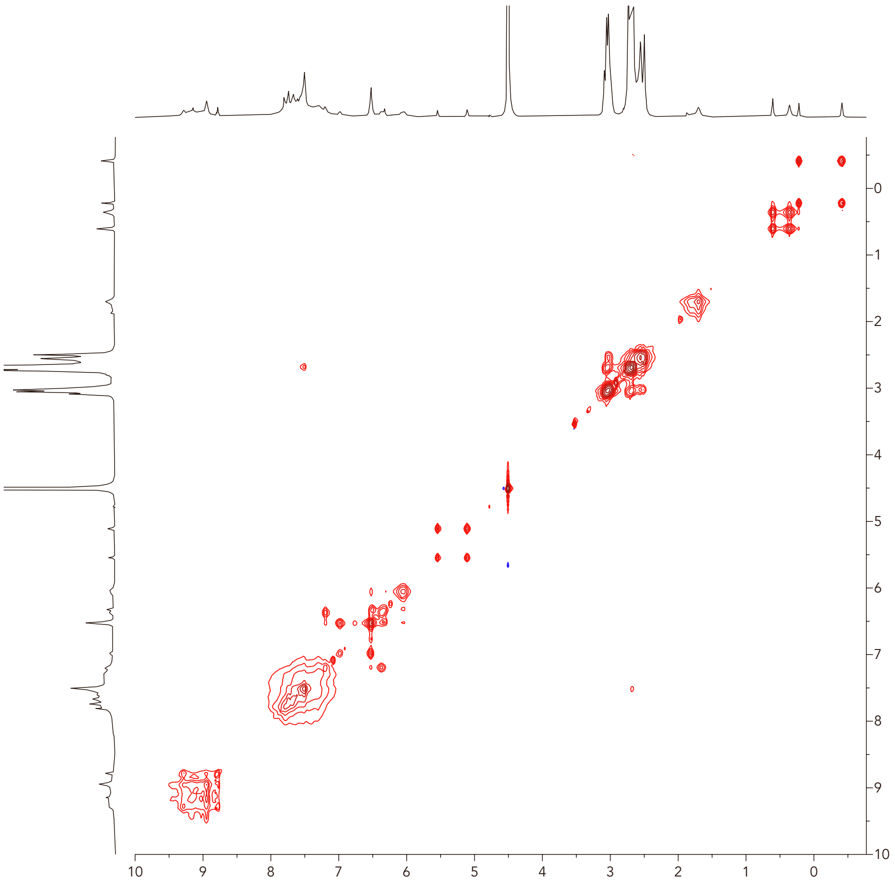

Figure S71. ^{1}H–^{1}H NOESY NMR spectrum of (**a2·b1**)⊂**C** (in the presence of (**a2**)$_2$⊂**C**, (**b1**)$_2$⊂**C**, and free **C**) (600 MHz, D_2O, 320 K).

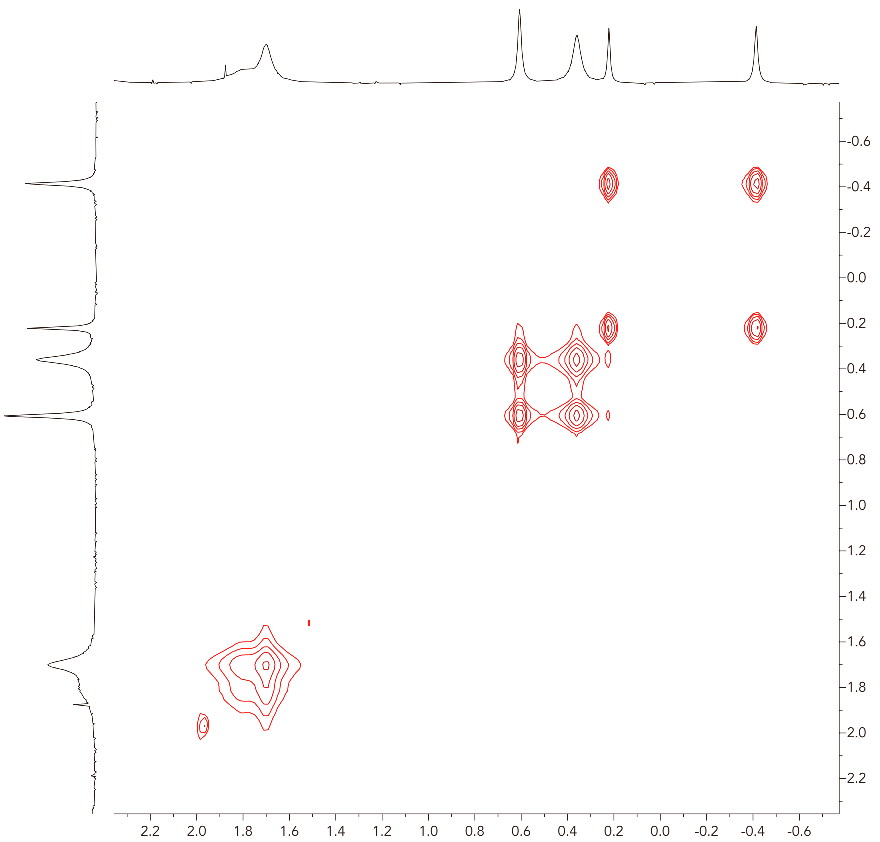

Figure S72. Partial ^{1}H–^{1}H NOESY NMR spectrum of (**a2**·**b1**)⊂**C** (in the presence of (**a2**)$_2$⊂**C**, (**b1**)$_2$⊂**C**, and free **C**) (600 MHz, D_2O, 320 K).

Heterodimeric inclusion complex (**a1**·**b2**)⊂**C** was obtained upon mixing homodimers (**a1**)$_2$⊂**C** and (**b2**)$_2$⊂**C** in a 1:1 ratio (with respect to the cage; note: the encapsulation yield for (**b2**)$_2$⊂**C** is ~60%, thus ~20% of the cage present in the system is unoccupied). The coexistence of the heterodimer with the (**a1**)$_2$⊂**C** homodimer (note: the spectrum does not show the presence of residual (**b2**)$_2$⊂**C**) resulted in a complex NMR spectrum; consequently, only characteristic peaks originating from the guests are listed below (guest protons with an apostrophe denote guests encapsulated within heterodimer (**a1**·**b2**)⊂**C**; those without an apostrophe refer to **a1** within homodimer (**a1**)$_2$⊂**C**):

^{1}H NMR (600 MHz, D_2O, 323 K); guest peaks: δ = 5.56 (s, **b2**$_{3'}$), 1.72 (s, **b2**$_{1'}$), 1.00 (s, **a1**$_{CH_3}$′), 0.26 (s, **b2**$_{2'}$), 0.13 (s, **a1**$_{CH_3}$).

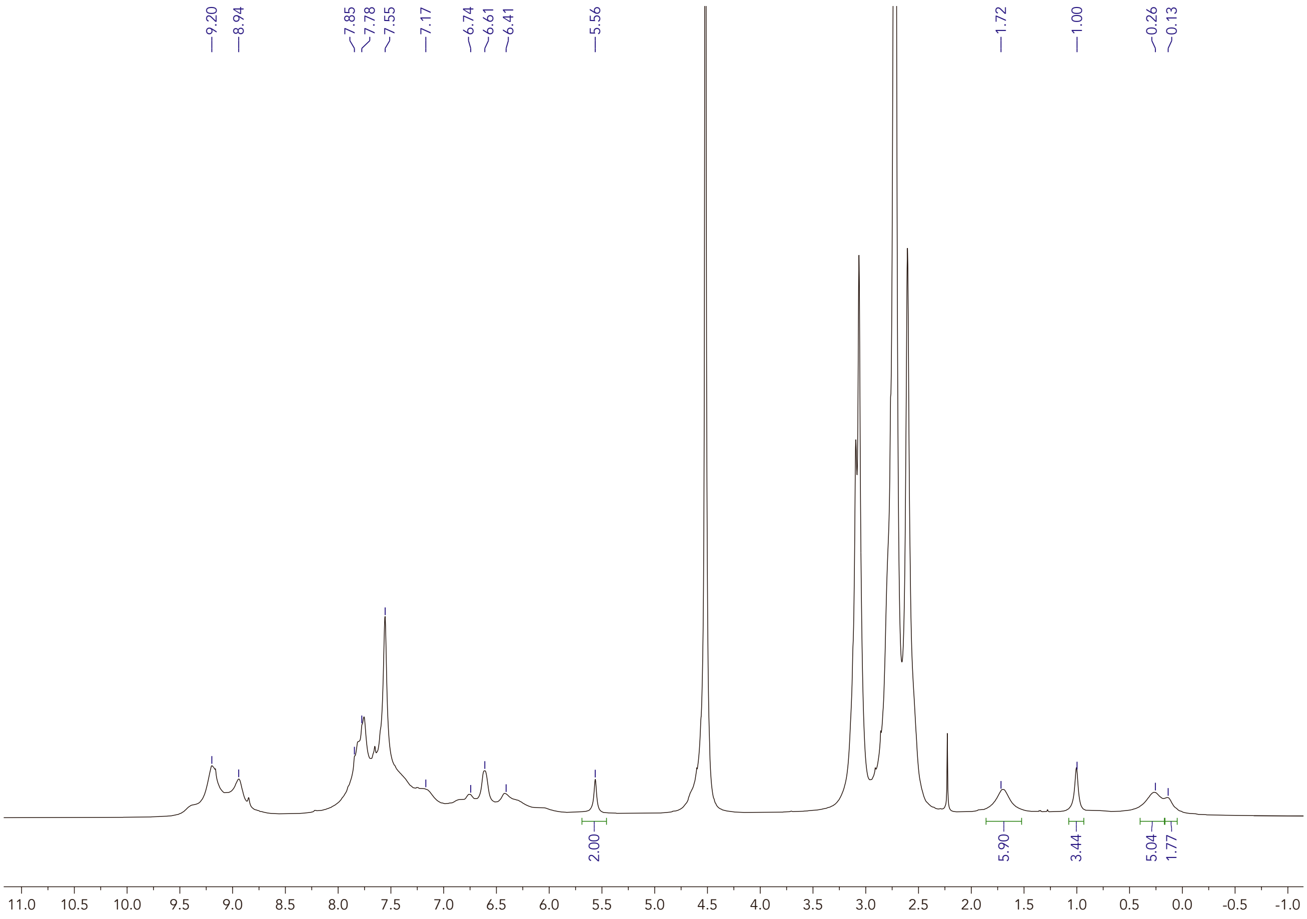

Figure S73. ^{1}H NMR spectrum of (**a1**·**b2**)⊂**C** (in the presence of (**a1**)$_2$⊂**C** and free **C**) (500 MHz, D_2O, 323 K).

Heterodimeric inclusion complex (**a4·b4**)$\subset$**C** was obtained upon mixing homodimers (**a4**)$_2\subset$**C** and (**b4**)$_2\subset$**C** in a 1:1 ratio. The coexistence of the heterodimer with the two homodimers resulted in a complex NMR spectrum; consequently, only characteristic peaks originating from the guests are listed below (note: guest protons with an apostrophe denote guests encapsulated within heterodimer (**a4·b4**)$\subset$**C**; those without an apostrophe refer to guests within homodimer (**b4**)$_2\subset$**C** (Ref. 2)):

^{1}H NMR (600 MHz, D_2O, 328 K); guest peaks: δ = 1.84 (s, $\mathbf{b4_1}$+$\mathbf{b4_{1'}}$), 1.56 (s, $\mathbf{b4_4}$), 1.51 (s, $\mathbf{b4_{4'}}$), –0.05 (s, $\mathbf{b4_2}$), –0.65 (s, $\mathbf{b4_{2'}}$).

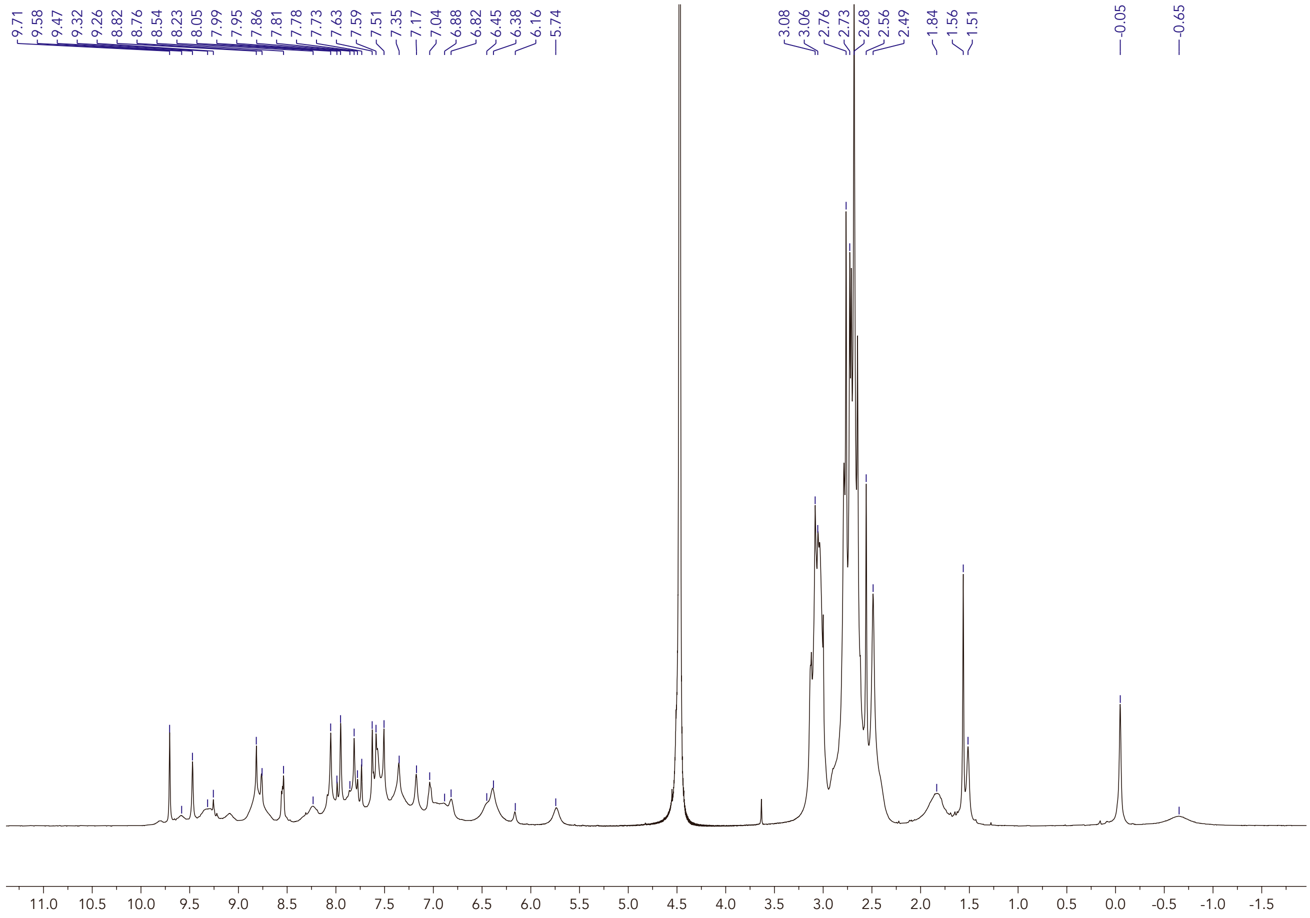

Figure S74. ^{1}H NMR spectrum of (**a4·b4**)$\subset$**C** (in the presence of (**a4**)$_2\subset$**C** and (**b4**)$_2\subset$**C**) (500 MHz, D_2O, 328 K).

7. X-ray data collection and structure refinement

Single crystals of homodimer complexes (**a1**)$_2$⊂**C**, (**a2**)$_2$⊂**C**, (**a3**)$_2$⊂**C**, and (**a4**)$_2$⊂**C** were obtained by slow evaporation of water from the respective aqueous solutions. Single crystals of the (**a2**·**b1**)⊂**C** heterodimer were obtained after water evaporation from a mixture of (**a2**)$_2$⊂**C** and (**b1**)$_2$⊂**C** (a mixture of colorless and orange crystals was obtained; the latter were collected for further analysis). All crystals were coated in oil (as they tend to lose crystalline water quickly) and flash-frozen in a liquid nitrogen stream. Data were collected at either 80 K or 100 K. The diffraction data for (**a1**)$_2$⊂**C** and (**a2**)$_2$⊂**C** were collected on a Rigaku XtaLAB PRO diffractometer equipped with a Dectris Pilatus S200K detector and processed with CrysAlisPRO. The diffraction data for (**a3**)$_2$⊂**C** and (**a2**·**b1**)⊂**C** were collected on a Rigaku XtaLAB Synergy-R DW equipped with a HyPix ARC 150º detector and processed with CrysAlis^{PRO}. The diffraction data of (**a4**)$_2$⊂**C** were collected on a Bruker APEX-II Kappa CCD diffractometer and processed with Bruker SAINT. The structures were solved by direct methods using SHELXT as implemented in Olex2 GUI.[4] All non-hydrogen atoms were further refined by SHELXL[5] with anisotropic displacement coefficients. Hydrogens were placed in calculated positions and refined in a riding mode.

The unit cell parameters of (**a2**·**b1**)⊂**C** are the same as for previously published (**b1**)$_2$⊂**C**,[2] however, with a significantly higher number of electron density maxima. The refinement of (**a2**·**b1**)⊂**C** required assigning the additional electron density maxima to the atoms of **a2** molecules (which occupied the positions of **b1** within some cages). The electron density maxima originating from bromine atoms were treated as anchors, with the remainder of the **a2** molecule restrained. Two recognized anthracene frames were freely refined to yield ~41% occupancy of the guest positions (50% would be expected for each **a2** and **b1** in an ideal heterodimer). This result means either that the crystal is composed of the heterodimer and **b1** homodimer units and/or the residual disordered **b1** positions remain unaccounted for. A more complicated disorder is also possible because free refinement of **a2** occupancy yielded 44%. Contributions from disordered solvent and/or counterion molecules were removed with the SQUEEZE protocol of Platon[6]/Olex2.[4] The crystal data and structure refinement data are summarized in Table S1.

Species	(**a1**)$_2$⊂**C**	(**a2**)$_2$⊂**C**	(**a3**)$_2$⊂**C**	(**a4**)$_2$⊂**C**	(**a2**·**b1**)⊂**C**
**CCDC No.**	2103596	2103597	2103576	2103598	2103577
**Formula***	$C_{126}H_{168}N_{46}O_{30}Pd_6$	$C_{124}H_{162}Br_2N_{48}O_{36}Pd_6$	$C_{126}H_{168}N_{46}O_{47}Pd_6$	$C_{128}H_{166}N_{48}O_{37}Pd_6$	$C_{118.9}H_{165.56}B_{0.85}Br_{0.78}F_{1.71}N_{47.71}O_{30}Pd_6$
**Molecular weight***	3445.47	3699.23	3477.47	3607.48	3485.88
**Crystal system**	Monoclinic	Monoclinic	Triclinic	Triclinic	Monoclinic
**Space group**	$P2_1$/c	$P2_1$/c	$P\bar{1}$	$P\bar{1}$	$P2_1$/c
**Crystal size (mm)**	0.15×0.09×0.08	0.20×0.13×0.02	0.23×0.03×0.02	0.20×0.20×0.16	0.46×0.31×0.28
**Crystal color and shape**	Yellow tablet	Colorless plate	Colorless needle	Colorless prism	Orange block
**Temperature (K)**	100	100	100	100	80
**Wavelength (Å)**	0.71073	1.54184	1.54184	0.71073	0.71073
**a (Å)**	15.1278(3)	21.8482(2)	18.6116(4)	18.6926(6)	15.07752(13)
**b (Å)**	41.2674(9)	29.3112(3)	19.4974(4)	18.8109(7)	41.6864(4)
**c (Å)**	15.2858(3)	15.2471(1)	23.2282(5)	27.1571(9)	15.37691(14)
**α (°)**	90	90	83.0525(18)	101.046(2)	90
**β (°)**	95.371(2)	91.592(1)	82.6694(18)	100.108(2)	95.1968(8)
**γ (°)**	90	90	85.9915(18)	90.272(2)	90
**Volume (Å^3)**	9500.8(3)	9760.43(15)	8286.0(3)	9219.4(6)	9625.08(15)
**Z**	2	2	2	1	2
**r_{calcd} (g·cm^{-1})**	1.204	1.259	1.394	1.299	1.203
**µ (mm^{-1})**	0.623	5.436	5.801	0.648	0.778
**No. of reflections (unique)**	140752 (25542)	303369 (19930)	105235 (30069)	293398 (37628)	489814 (83039)
**R_{int}**	0.0512	0.1333	0.1147	0.0635	0.0440
**Completeness to θ (%)**	99.6	99.9	99.6	99.7	99.8
**Data / restraints / parameters**	25542 / 178 / 920	19930 / 97 / 1009	30069 / 50 / 1957	37628 / 171 / 2009	83039 / 1231 / 1340
**Goodness-of-fit on F^2**	1.053	1.043	1.041	1.067	1.042
**Final R_1 and wR_2 indices [I>2s(I)]**	0.0743, 0.1751	0.0751, 0.2004	0.0937, 0.2302	0.0679, 0.1546	0.0836, 0.2528
**R_1 and wR_2 indices (all data)**	0.0840, 0.1794	0.0805, 0.2065	0.1311, 0.2512	0.0940, 0.1685	0.1722, 0.2899

Table S1. Crystallographic data. (*Derived from the crystal structure; masked molecules not included)

Table S2 reports the structural parameters of cage **C** (empty and with guests **a1**, **a2**, **a3**, **a4**, **b1**, **b2**, and **b4**; crystal structures of **C**, (**b1**)$_2$⊂**C**, (**b2**)$_2$⊂**C** and (**b4**)$_2$⊂**C** were reported previously[2,7]) (note that whereas single crystals of inclusion complexes (**a1**)$_2$⊂**C** and (**a2**)$_2$⊂**C** contained a single conformation within one unit cell; those of (**a3**)$_2$⊂**C** and (**a4**)$_2$⊂**C** contained two different conformations of the inclusion complex).

Species	Pd_{ax}–Pd_{ax} distance	Pd_{eq}–Pd_{eq} distance	Angle at Pd_{ax}
**C**	16.86 Å	18.23 Å	88.59°
(**b1**)$_2$⊂**C**	18.44 Å	16.84 Å	76.38°
(**b2**)$_2$⊂**C**	18.42 Å	16.91 Å	76.89°
(**b4**)$_2$⊂**C**	18.16 Å	17.09 Å	78.65°
(**b4**)$_2$⊂**C′**	18.59 Å	16.70 Å	75.37°
(**a1**)$_2$⊂**C**	18.57 Å	16.61 Å	74.72°
(**a2**)$_2$⊂**C**	17.65 Å	17.55 Å	82.10°
(**a3**)$_2$⊂**C**	18.62 Å	16.34 Å	82.69°
(**a3**)$_2$⊂**C′**	18.72 Å	16.52 Å	74.13°
(**a4**)$_2$⊂**C**	17.70 Å	17.37 Å	81.79°
(**a4**)$_2$⊂**C′**	18.32 Å	16.76 Å	76.77°

Table S2. Structural parameters for empty **C** and **C** encapsulating different guests. Pd_{ax} and Pd_{eq} denote axial and equatorial palladium nodes, respectively. "Pd_{ax}–Pd_{ax} distance" is defined as the distance between two axial palladium nodes; "Pd_{eq}–Pd_{eq} distance" is defined as the average distance between two opposite equatorial palladium nodes. "Angle at Pd_{ax}" is defined as the angle between two triimidazole ligand planes at the axial palladium. The distances and angles were measured in Mercury 4.2.0 software.

Table S3 reports the structural parameters for crystals of **a1**–**a4** as small molecules vs. homodimeric complexes within cage **C**. Analogous parameters for **b1**, **b2**, and **b4** were reported previously.[2] Structural parameters for free **a1**, **a2**, **a3**, and **a4** were extracted from the literature (Refs. 8, 9, 10, and 11, respectively).

Species	Plane-to-plane distance	Center-to-center distance	Orientation
(**a1**)$_2$⊂**C**	3.54 Å	5.30 Å	antiparallel
(**a1**)$_\infty$	3.53 Å	3.87 Å	antiparallel
(**a2**)$_2$⊂**C**	3.54 Å	3.92 Å	antiparallel
(**a2**)$_\infty$	3.46 Å	3.94 Å	parallel
(**a3**)$_2$⊂**C**	3.45 Å	3.85 Å	antiparallel
(**a3**)$_2$⊂ **C′**	3.46 Å	3.95 Å	antiparallel
(**a3**)$_\infty$	3.37 Å	5.21 Å	parallel
(**a4**)$_2$⊂**C**	3.46 Å	4.08 Å	–
(**a4**)$_2$⊂ **C′**	3.42 Å	4.37 Å	–
(**a4**)$_\infty$	3.53 Å	3.95 Å	–

Table S3. Structural parameters for **a1**, **a2**, **a3**, and **a4** within single crystals, i.e., **a**$_\infty$, and inside cage **C**, i.e., (**a**)$_2$⊂**C**. To calculate plane-to-plane distances between two neighboring guests, individual planes were defined as the planes formed by the central ring or, in the case of **a4**, the two central fused rings. For calculating the center-to-center distances of **a1**, **a2**, and **a3**, centroids of the central rings were calculated. For **a4**, the centroid was defined as the middle point of the **a4**$_5$–**a4**$_5$ bond.

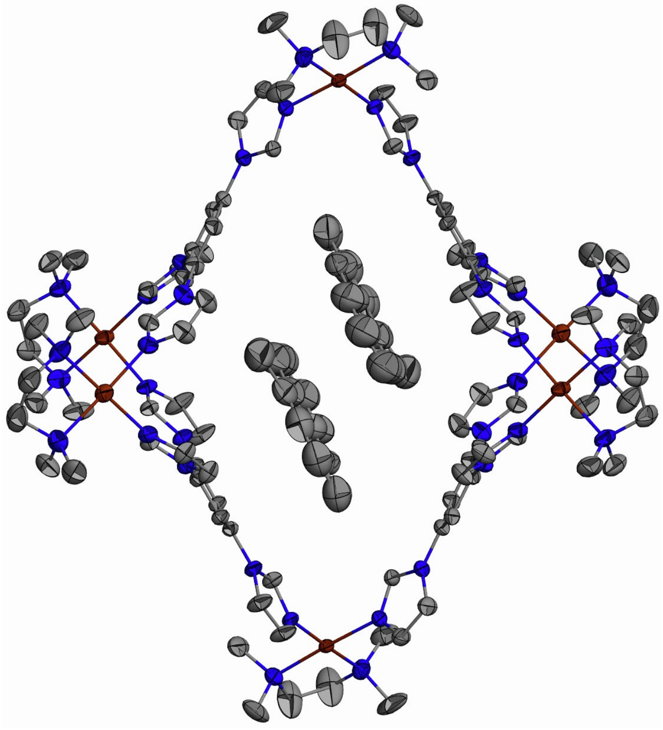

Figure S75. ORTEP representation of the X-ray structure of inclusion complex (**a1**)$_2$⊂**C** (displacement ellipsoids at a 50% probability level). Hydrogens, anions, and solvent molecules were eliminated for clarity. Pd, brown; C, gray; N, blue.

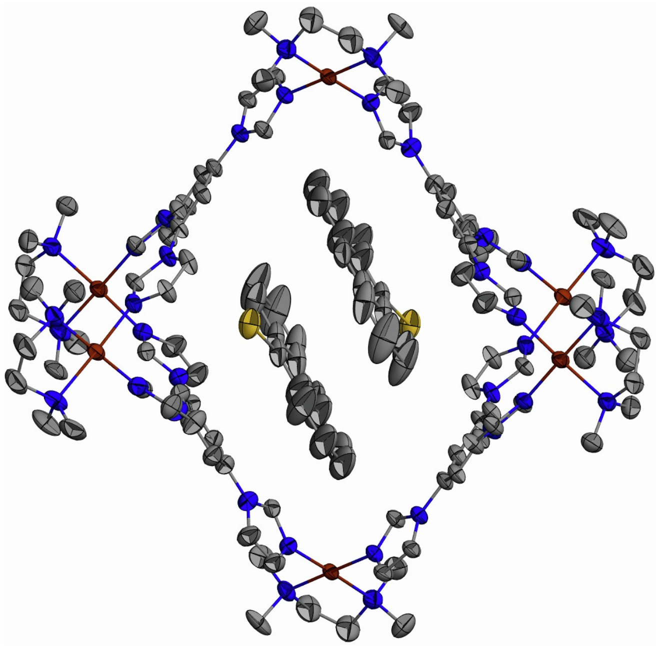

Figure S76. ORTEP representation of the X-ray structure of inclusion complex (**a2**)$_2$⊂**C** (displacement ellipsoids at a 50% probability level). Hydrogens, anions, and solvent molecules were eliminated for clarity. Pd, brown; C, gray; N, blue; Br, yellow.

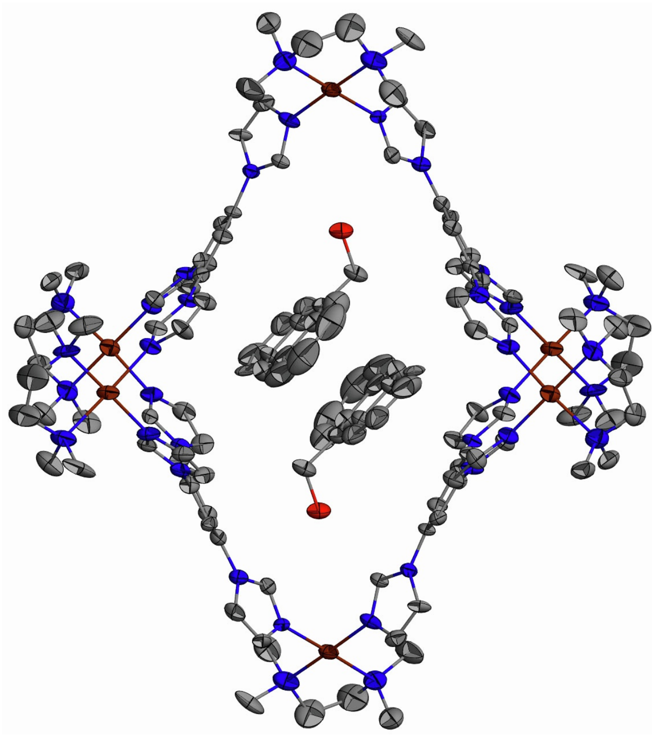

Figure S77. ORTEP representation of the X-ray structure of one of two conformers of inclusion complex (**a3**)$_2$⊂**C** (displacement ellipsoids at a 50% probability level; the structure of the second conformer is very similar; see the CIF file).). Hydrogens, anions, and solvent molecules were eliminated for clarity. Pd, brown; C, gray; N, blue; O, red.

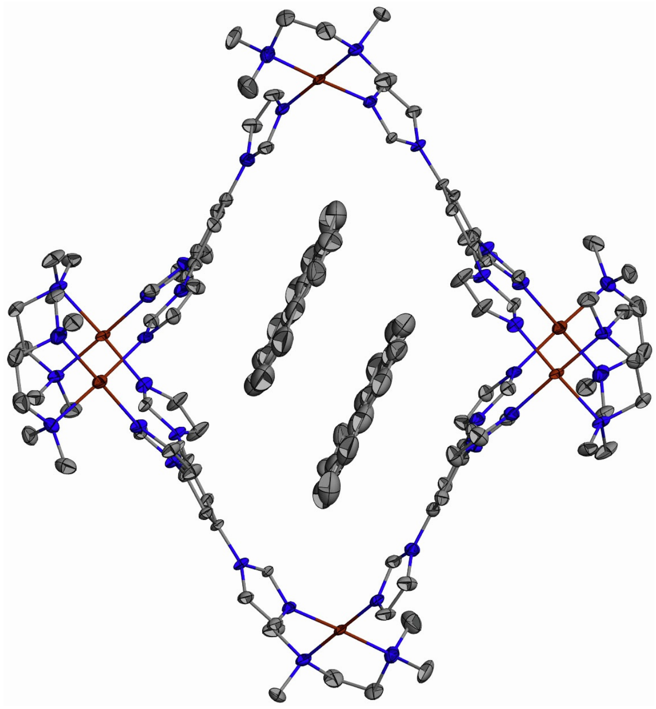

Figure S78. ORTEP representation of the X-ray structure of one of two conformers of inclusion complex (**a4**)$_2$⊂**C** (displacement ellipsoids at a 50% probability level; the structure of the second conformer is very similar; see the CIF file). Hydrogens, anions, and solvent molecules were eliminated for clarity. Pd, brown; C, gray; N, blue.

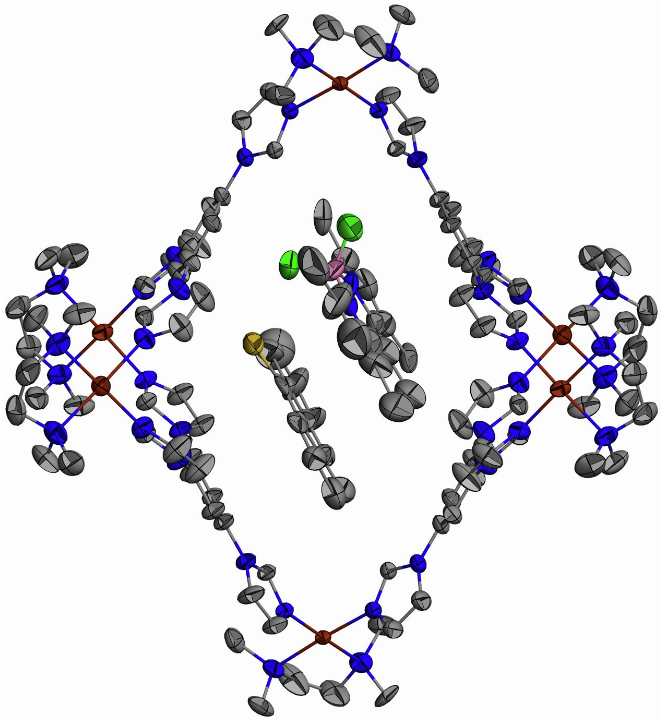

Figure S79. ORTEP representation of the X-ray structure of the first conformer of inclusion complex (**a2·b1**)⊂**C** (displacement ellipsoids at a 50% probability level). Hydrogens, anions, and solvent molecules were eliminated for clarity. Pd, brown; C, gray; N, blue; B, pink, F, green; Br, yellow.

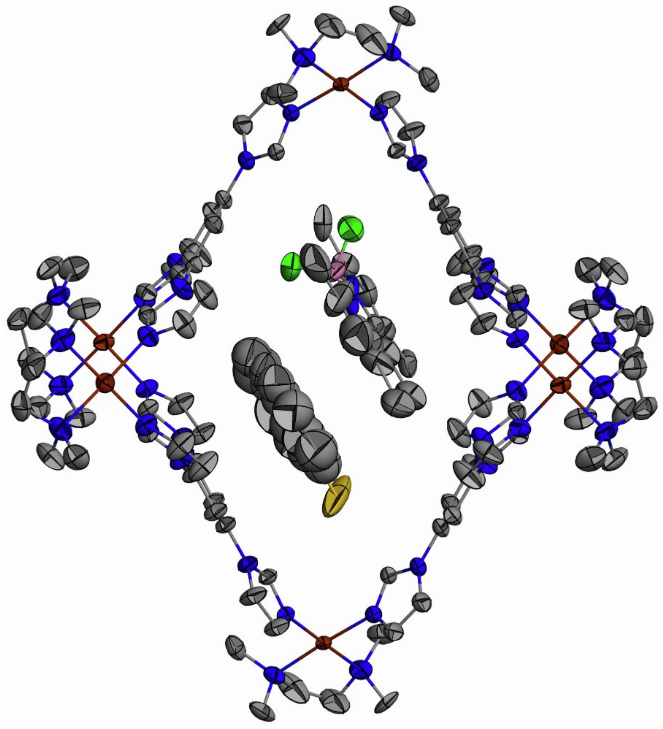

Figure S80. ORTEP representation of the X-ray structure of the second conformer of inclusion complex (**a2·b1**)⊂**C** (displacement ellipsoids at a 50% probability level). Hydrogens, anions, and solvent molecules were eliminated for clarity. Pd, brown; C, gray; N, blue; B, pink, F, green; Br, yellow.

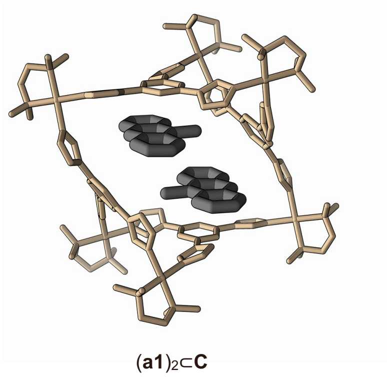

(**a1**)$_2$⊂**C**

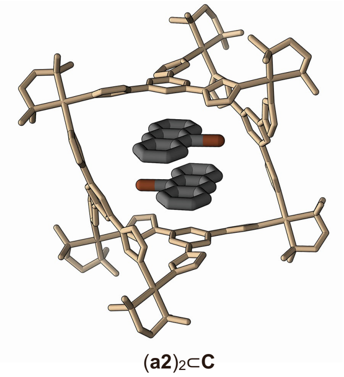

(**a2**)$_2$⊂**C**

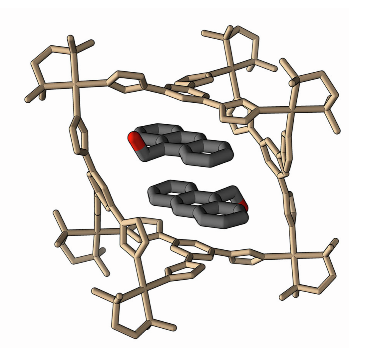

(**a3**)$_2$⊂**C**

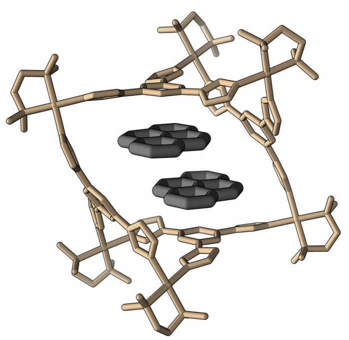

(**a4**)$_2$⊂**C**

Figure S81. Comparison of the X-ray structures of (**a1**)$_2$⊂**C**, (**a2**)$_2$⊂**C**, (**a3**)$_2$⊂**C**, and (**a4**)$_2$⊂**C** (note that the X-ray structures of both (**a3**)$_2$⊂**C** and (**a4**)$_2$⊂**C** contained two slightly different conformations in a 1:1 ratio; only one of each is shown here; for the other conformation, see the CIF files).

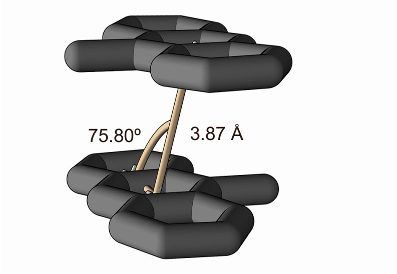

a1

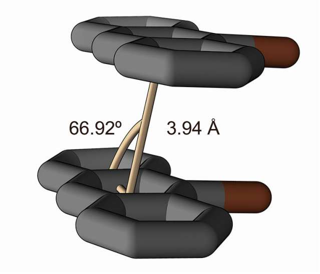

a2

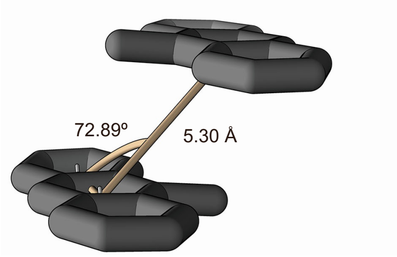

(**a1**)$_2$⊂**C**

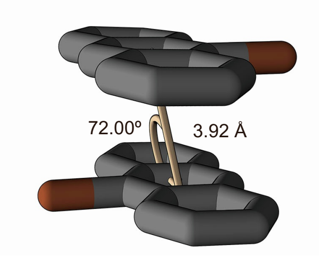

(**a2**)$_2$⊂**C**

Figure S82. Comparison of the X-ray structures of **a1** and **a2** as free molecules (top) and encapsulated by **C** (bottom). The center-to-center distances and slip angles for free **a1** and **a2** are taken from Refs. 8 and 9, respectively.

8. DFT calculations of the (**a2**·**b1**)⊂**C** heterodimer

To verify the feasibility of two different arrangements of the **b1** guest within the heterodimer, DFT calculations were performed at the B3LYP-D3/6-31G(d,p)/LANL2DZ(Pd) level of theory[12,13] using the Gaussian 16[14] software. The structures were optimized as dications to preserve the symmetric distribution of counterions, with the initial geometries and distributions of nitrates as in the crystal structure. No imaginary frequencies were found, which confirmed achieving the energetic minima. The energy difference (with zero-point correction included) between the optimized structures (5.1 kcal/mol) suggests that the isomer with **a2**'s Br atom pointing towards the axial Pd (Figure S83B) is slightly more stable (the energy gap could be reduced by including the solvent (water) in the optimization process). Both complexes show some degree of nonplanarity of the guests under energy-optimized conditions, in contrast to restrained models from X-ray diffraction analysis.

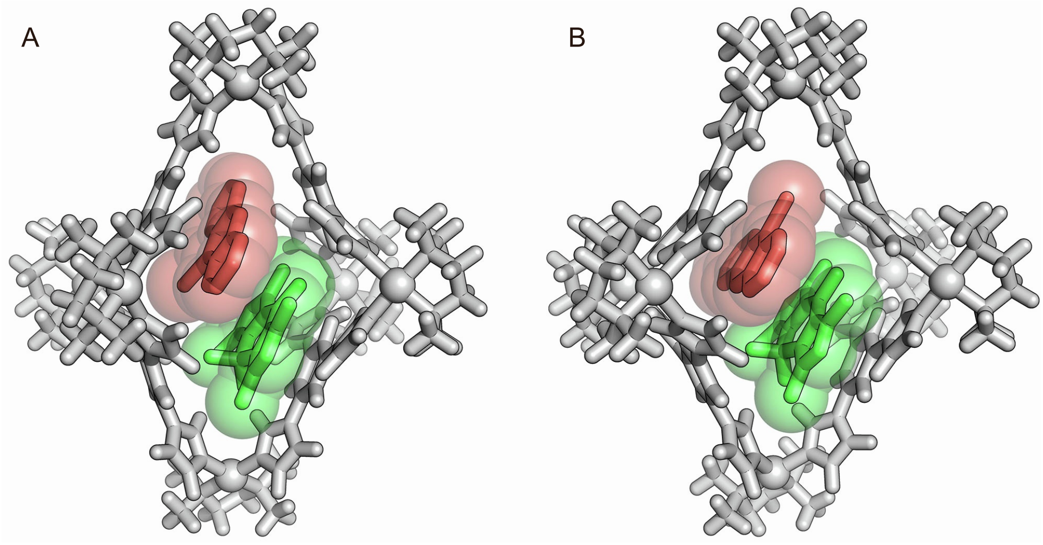

Figure S83. Energy-optimized structures of the two isomers of the (**a2**·**b1**)⊂**C** heterodimer.

9. Steady-state optical properties of **a1**, **a2**, **a3**, and **a4** and their inclusion complexes

Unless reported otherwise, UV-vis absorption measurements were carried out on 0.031 mM solutions of inclusion complexes (the concentration in terms of cage units) at ambient temperature. The solution of free cage **C** is transparent in the visible range, with an absorption onset at ~380 nm. The optical properties of BODIPYs **b1**, **b2**, and **b4** dissolved in MeCN or encapsulated within **C** in water were discussed previously.[2]

Solutions of **a1**, **a2**, **a3**, and **a4** in MeCN are colorless, but their UV-vis absorption spectra all show characteristic absorbance patterns in the near-UV area (between 300 nm and 400 nm; see the red traces in Figure S84). Compounds **a1** and **a2** exhibit similar spectra with a 'finger'-type pattern typical for various anthracene derivatives.[15] Peak maxima of **a1** in MeCN can be found at 348 nm, 366 nm, and 386 nm; those for **a2** appear at 350 nm, 368 nm, and 388 nm, and those for **a3** – at 349 nm, 367 nm, and 387 nm. The spectrum of **a4** in MeCN is dominated by two sharp absorption bands in the near-UV region, centered at 318 nm and 335 nm.

Upon encapsulation, the characteristic shapes of the absorption spectra of the guests are retained, albeit the peaks are slightly broader and red-shifted (see the black spectra in Figure S84A–D). For $(\mathbf{a1})_2\subset\mathbf{C}$, peak maxima are located at 357 nm, 376 nm, and 397 nm; for $(\mathbf{a2})_2\subset\mathbf{C}$, at 361 nm, 380 nm, and 401 nm; for $(\mathbf{a3})_2\subset\mathbf{C}$, at 356 nm, 373 nm, and 393 nm; and for $(\mathbf{a4})_2\subset\mathbf{C}$, at 332 nm and 348 nm. These changes can be attributed to a combination of solvatochromic and confinement effects (i.e., the proximity of the π systems of the encapsulated guests and the cage's walls).

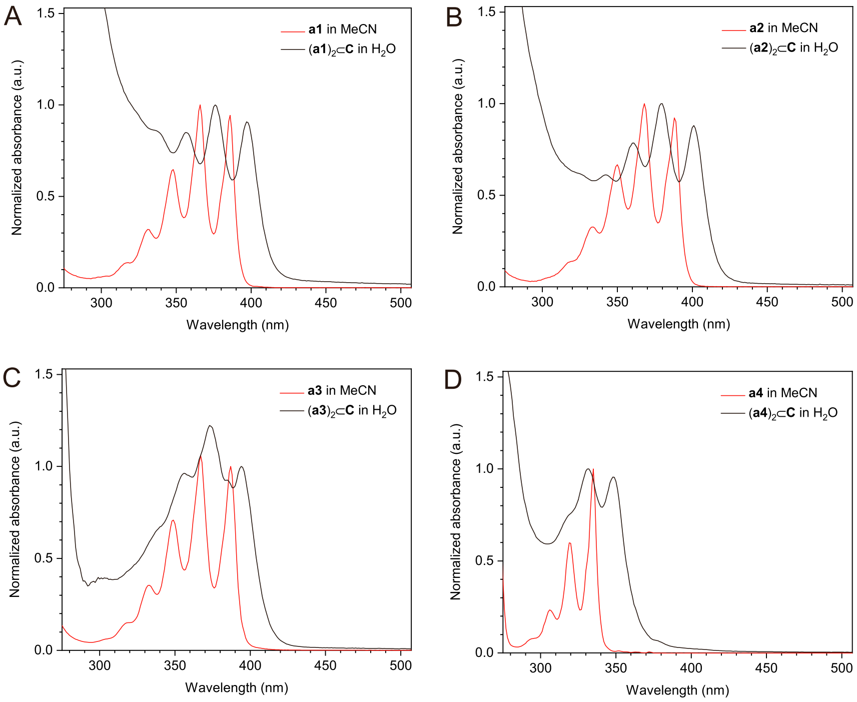

Figure S84. Normalized absorption spectra of (A) **a1**, (B) **a2**, (C) **a3**, and (D) **a4** encapsulated in **C** (black) or free in MeCN (red).

Notably, the extinction coefficients ε of the guests decrease strongly upon encapsulation. This effect can be visualized by adding MeCN to the aqueous solutions of the complexes, as exemplified for (**a2**)$_2$⊂**C** and (**a4**)$_2$⊂**C** in Figure S85 (which plots normalized absorption). As shown previously for BODIPY dyes,[2] adding polar organic solvents to aqueous solutions of inclusion complexes drastically decreases the affinity of guests to host **C**, resulting in guest release. By monitoring the absorption spectra during titration with MeCN, gradual release of guests can be followed conveniently (Figure S85C).

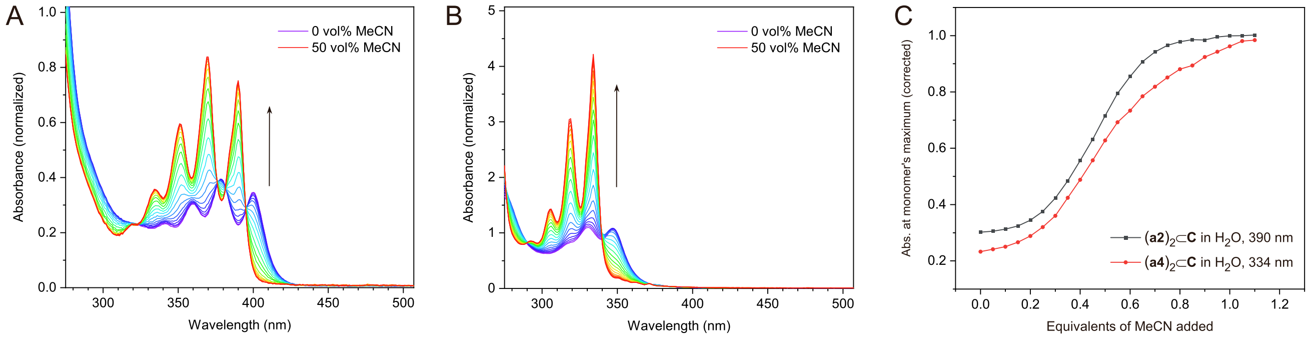

Figure S85. Release of **a2** from (**a2**)$_2$⊂**C** (A) and **a4** from (**a4**)$_2$⊂**C** (B) by stepwise addition of MeCN. The absorbance was normalized with respect to the volume of MeCN added. (C) The gradual release of **a2** and **a4** from cage **C** in the presence of increasing amounts of MeCN (molar equivalents MeCN with respect to water).

While **a1**–**a4** are relatively strong fluorophores (emitting in the near-UV and blue region), their fluorescence is mostly quenched upon encapsulation within **C**. Figure S86 (panels A–D) compares the emission spectra of free **a1**–**a4** in DCM with the spectra of encapsulated **a1**–**a4**; in all cases, concentrations of the free and encapsulated guest were the same.

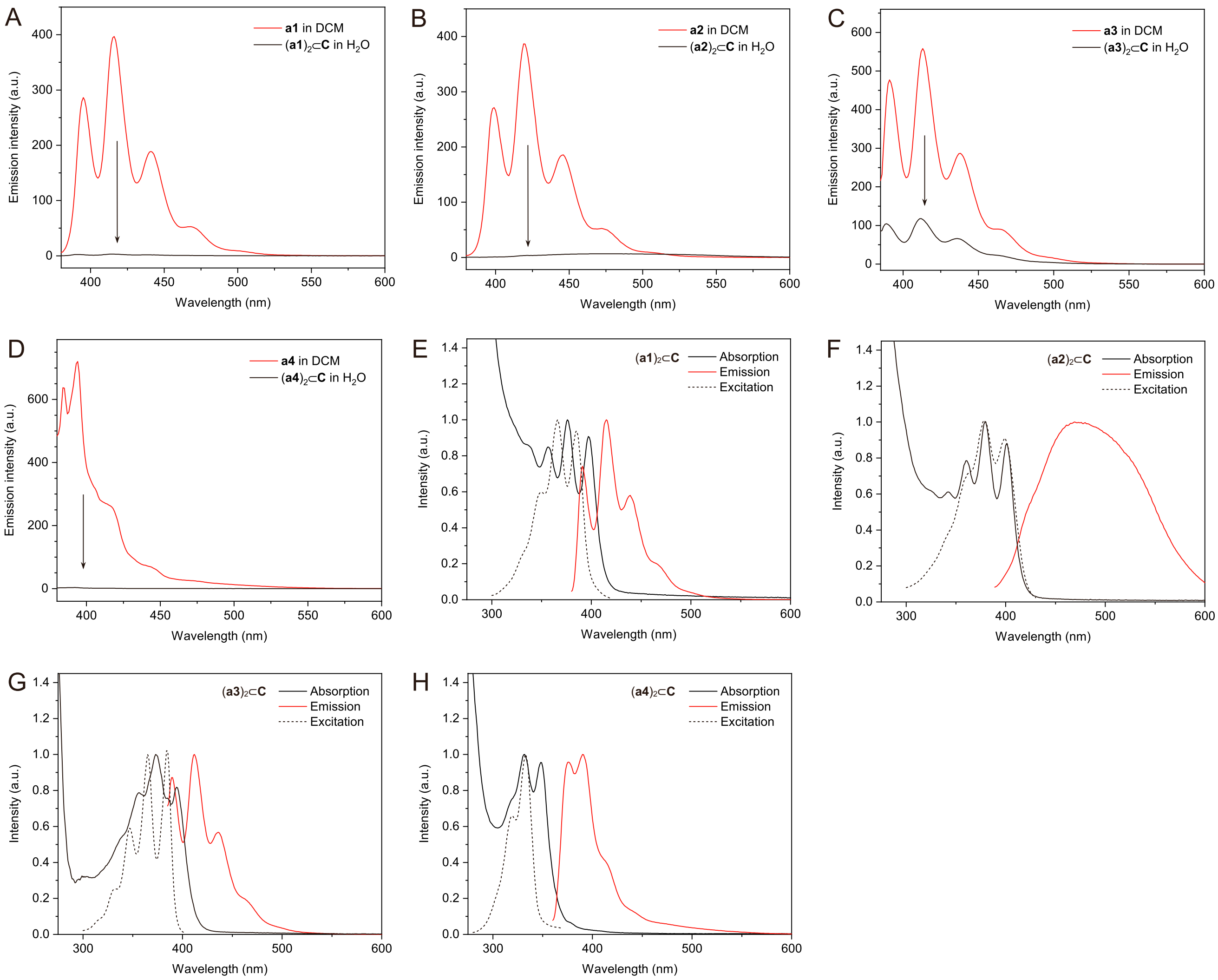

Figure S86. (A–D) Emission spectra of **a1**, **a2**, **a3**, and **a4** dissolved in DCM (red) or as homodimers within **C** in water (black) (λ_{exc} = 380 nm for **a1**– **a3**; λ_{exc} = 340 nm for **a4**). In all cases, the concentration of the guest was the same (95 μM). The emission observed for (**a3**)$_2$⊂**C** (panel C) originates from a small amount of unencapsulated **a3** in equilibrium with (**a3**)$_2$⊂**C**; note that this anthracene has a relatively good solubility in water. (E–H) Normalized absorption, emission, and excitation spectra for **a1**–**a4** after encapsulation within **C** (λ_{exc} = 380 nm for **a1**–**a3**; λ_{exc} = 340 nm for **a4**).

10. Formation of heterodimeric inclusion complexes and their steady-state optical properties

Heterodimers comprising both **a** (**a1**, **a2**, **a3**, or **a4** and **b** (**b1**, **b2**, **b3**, or **b4**) were formed by mixing aqueous solutions of the respective homodimers. Depending on the guest's identity, heterodimers formed either instantly or over periods of up to several minutes. We confirmed that all sixteen possible **a·b** heterodimer combinations could be obtained by mixing the respective homodimers; several representative examples were characterized in detail and are discussed below.

The initial experiments were based on the **a2** + **b1** combination. First, we prepared two solutions of cage **C** (0.5 mL; c = 1.57 μM) and saturated them with guests **a2** and **b1**, respectively (note that the uptake of **b1** is limited to ~50% of cages). The resulting spectra of (**a2**)$_2$⊂**C** and (**b1**)$_2$⊂**C**, after removing excess (unbound) guests, are shown as the blue trace in Figure S87A and the red trace in Figure S87B, respectively.

As reported previously[2], the complex (**b1**)$_2$⊂**C** shows a strong absorption band centered at 480 nm, which originates from the $S_1 \leftarrow S_0$ transition of **b1** within the homodimer. Compared with dye **b1** dissolved in MeCN, this band is blue-shifted by 11 nm due to H-aggregation (in this case, H-dimerization) resulting from noncovalent complexation within **C**. For the same reason, the emission band is red-shifted (from 502 nm for **b1** in MeCN to 544 nm, giving rise to a significant Stokes shift of 64 nm, compared with 11 nm for **b1** in MeCN).[2]

Next, the (**a2**)$_2$⊂**C** solution was treated with solid **b1** (10 eq with respect to **C**). The mixture was stirred overnight, the solids were removed by filtration, and the supernatant was analyzed by UV-vis absorption spectroscopy. The resulting spectrum (Figure S87A) showed encapsulated **a2** and a new peak centered at 511 nm. This result demonstrates the ability of **b1** to partially displace **a2** from (**a2**)$_2$⊂**C**. This new 511 nm peak is distinct from the absorption of (**b1**)$_2$⊂**C** (red in Figure S87B) and it can be attributed to the (**a2·b1**)⊂**C** heterodimer.

In the reverse experiment, we treated the solution of (**b1**)$_2$⊂**C** with 10 eq of solid **a2** (also 10 eq is with respect to **C**). We observed that the white solid **a2** turned red, indicating the expulsion of **b1** from the cage, followed by its precipitation from water. After stirring overnight and filtration, a spectrum similar to that in the previous experiment was obtained in that the ratios of absorbance at ~510 nm and ~380 nm were similar (compare the green spectrum in Figure S87A to the black spectrum in Figure S87B; note the different scales on the *y*-axes). The similarities of the final spectra, despite the different ratios of the two guests (1:10 vs. 10:1), suggest that the (**a2·b1**)⊂**C** heterodimer forms more preferentially than either of the homodimers.

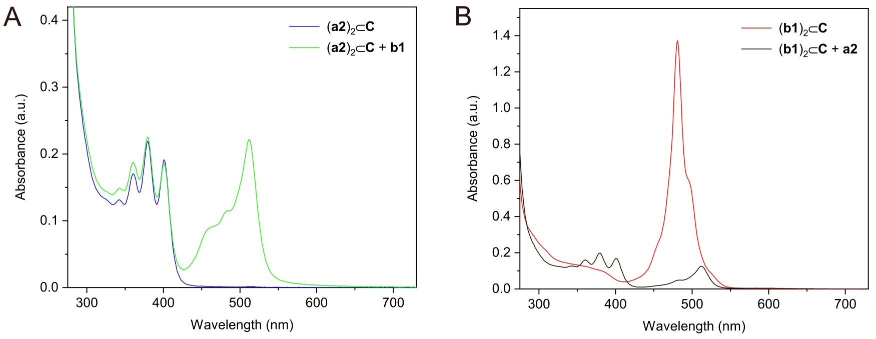

Figure S87. (A) UV-vis absorption spectrum of an aqueous solution of (**a2**)$_2$⊂**C** before (blue) and after (green) stirring with 10 eq of **b1** overnight. (B) UV-vis spectrum of an aqueous solution of (**b1**)$_2$⊂**C** before (red) and after (black) stirring with 10 eq of **a2** overnight.

Next, we proceeded to titration experiments. Titrating an aqueous solution of (**b1**)$_2$⊂**C** with (**a2**)$_2$⊂**C** results in changes in the visible part of the UV-vis absorption spectrum (Figure S88A; note that (**a2**)$_2$⊂**C** is transparent in the visible region, with no absorption above 420 nm). While the main absorbance band at 480 nm (due to the homodimer (**b1**)$_2$⊂**C**) gradually decreases, a new absorption peak centered at 511 nm appears (Figure S88A). The intensities of both bands are similar at ~1.5 eq of (**a2**)$_2$⊂**C** added (Figure S88B). Upon adding 4 eq (**a2**)$_2$⊂**C**, the 511 nm band prevails, with the original 480 nm band visible as a shoulder (Figure S88A).

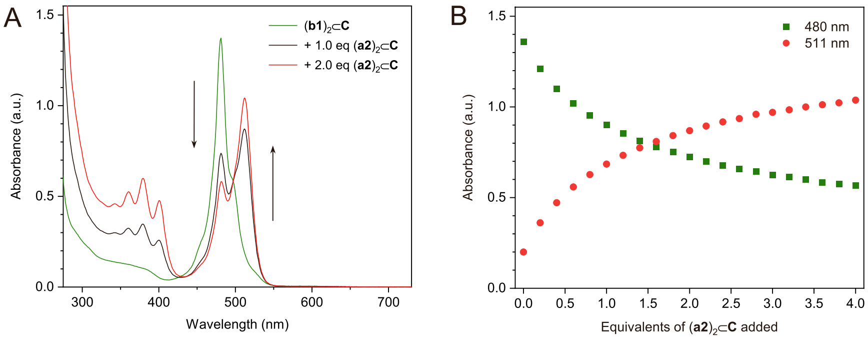

Figure S88. (A) Titration of (**b1**)$_2$⊂**C** with (**a2**)$_2$⊂**C** followed by UV-vis absorption spectroscopy. (C) Absorbance of 480 nm and 511 nm followed during the titration plotted in A.

We also followed the titration by fluorescence spectroscopy. Inclusion complex (**b1**)$_2$⊂**C** exhibits an unusually weak and broad emission band (in contrast to the sharp emission of free BODIPY dyes), with an emission maximum at 544 nm and the fluorescence quantum yield Φ_F of 0.13.[2] Upon adding (**a2**)$_2$⊂**C**, we observed the appearance and increase of a relatively sharp and intense emission band centered at 528 nm, which can be attributed to (**a2**·**b1**)$_2$⊂**C**. The Φ_F for a 1:20 mixture of (**b1**)$_2$⊂**C** and (**a2**)$_2$⊂**C** was determined to be 0.21, which is a significant increase compared with $\Phi_F = 0.13$ for (**b1**)$_2$⊂**C**, although still far from the strong fluorescence of **b1** in organic solvents (e.g., $\Phi_F = 1.00$ for **b1** in $CHCl_3$[16]). We also note that the Stokes shift decreased from 64 nm for (**b1**)$_2$⊂**C** to 17 nm, much closer to the value for **b1** in MeCN (11 nm). Excitation spectra clearly showed that the strong emission was connected to the emergence of the new absorbance band at 511 nm (Figure S89A), a conclusion that can also be reached by following the emission at 528 nm and the absorption at 511 nm in parallel (see Figure S89B).

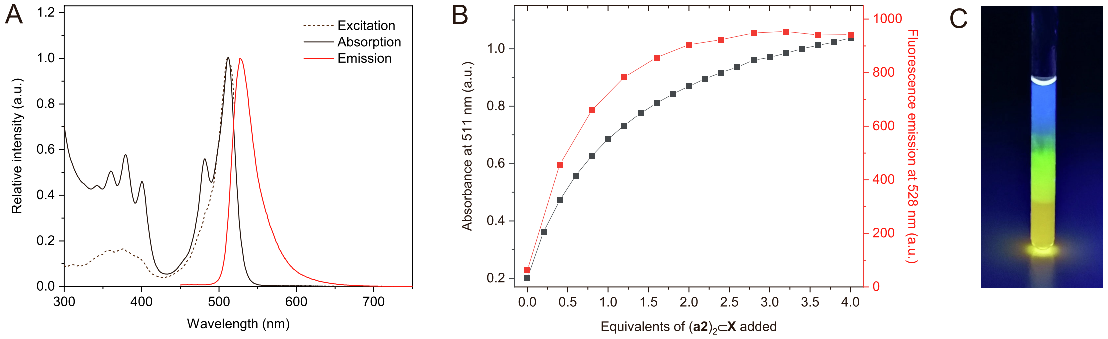

Figure S89. (A) UV-vis absorption spectrum (solid black line), excitation spectrum (dashed black line), and emission spectrum (red line, $\lambda_{exc} = 460$ nm) of a 4:1 mixture of (**a2**)$_2$⊂**C** and (**b1**)$_2$⊂**C**. (B) Absorbance at 511 nm (black) and emission intensity at 528 nm (red, $\lambda_{exc} = 460$ nm) followed during titration of (**b1**)$_2$⊂**C** with (**a2**)$_2$⊂**C**. (C) Photograph demonstrating the change in emission color during mixing of (**a2**)$_2$⊂**C** and (**b1**)$_2$⊂**C**. Initially, the tube contained an aqueous solution (**b1**)$_2$⊂**C** (bottom layer; weak yellow emission). Then, an aqueous

solution of (**a2**)$_2$⊂**C** was gently layered on top (weak blue emission). The middle layer forms as a result of mixing and an intense green emission due to the (**a2**·**b1**)$_2$⊂**C** heterodimer is observed.

Guest rearrangement between cages following mixing (**a2**)$_2$⊂**C** with (**b1**)$_2$⊂**C** can also be observed by the naked eye under ambient light. When (**a2**)$_2$⊂**C** (1 eq) was added to (**b1**)$_2$⊂**C** all at once, a rapid color change from dark orange to red-orange was observed. Under UV (365 nm) light excitation, the visual differences were even more pronounced: an immediate emission color change from weak yellow-orange to bright green occurred upon injecting (**a2**)$_2$⊂**C** into (**b1**)$_2$⊂**C** (see Figure 3A in the main text).

Similar results were observed upon treating (**b1**)$_2$⊂**C** with the other (**a**)$_2$⊂**C** complexes—(**a1**)$_2$⊂**C**, (**a3**)$_2$⊂**C**, and (**a4**)$_2$⊂**C**—indicating that the structure of anthracene/pyrene co-encapsulated with **b1** has little influence on its optical properties. These findings are in sharp contrast to those of Yoshizawa et al., who reported significant differences in the emission spectra of **b1** co-encapsulated with different aromatic hydrocarbons (e.g., phenanthrene and 9-methylanthracene) within a Pt-based coordination cage.[17]

Figure S90 shows the results of titration experiments in which aqueous solutions of (**b1**)$_2$⊂**C** were titrated with (**a1**)$_2$⊂**C**, (**a3**)$_2$⊂**C**, and (**a4**)$_2$⊂**C**. The visible-light regions of the UV-vis absorption spectra are very similar to each other and to that of (**b1**)$_2$⊂**C** titrated with (**a2**)$_2$⊂**C** (Figure 2A). Moreover, **b1**'s absorption and emission maxima (511 nm and 528 nm, respectively) remain unchanged for the different co-guests (**a1**, **a2**, **a3**, or **a4**).

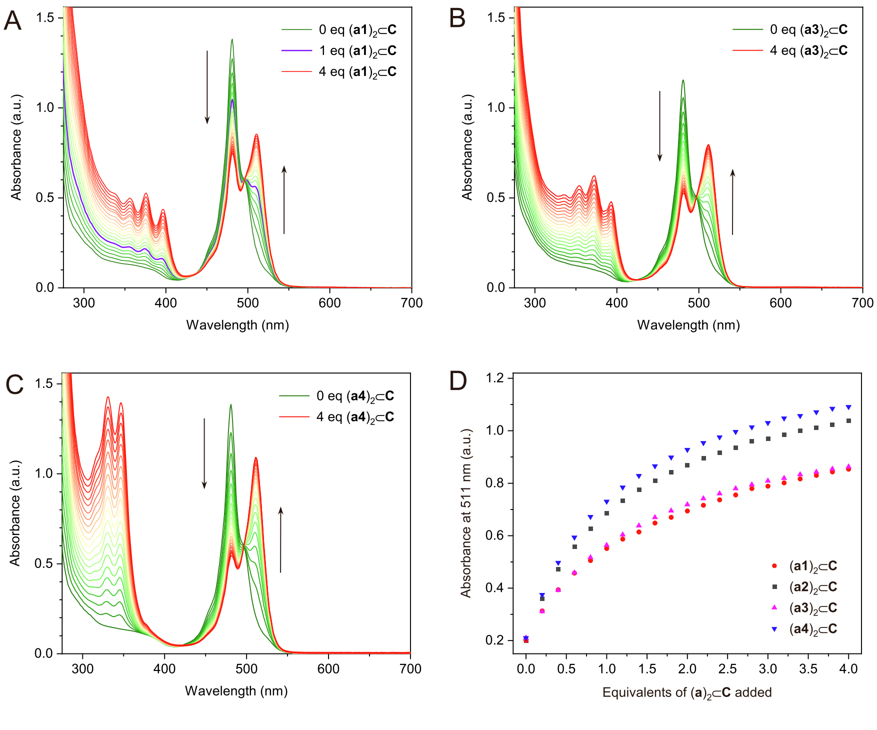

Figure S90. (A–C) UV-vis absorption spectra recorded during the titration of (**b1**)$_2$⊂**C** with: (A) (**a1**)$_2$⊂**C**, (B) (**a3**)$_2$⊂**C**, and (C) (**a4**)$_2$⊂**C**. (D) Comparison of all four titration experiments, whereby (**b1**)$_2$⊂**C** was treated with (**a1**)$_2$⊂**C**, (**a2**)$_2$⊂**C**, (**a3**)$_2$⊂**C**, or (**a4**)$_2$⊂**C**.

Despite the similar steady-state absorption and emission spectra, the fluorescence quantum yields Φ_F of the different heterodimer complexes varied significantly by changing **b1**’s partner within cage **C**. Specifically, we determined the values of Φ_F as 0.31 for (**a1**·**b1**)⊂**C**, 0.21 for (**a2**·**b1**)⊂**C**, 0.41 for (**a3**·**b1**)⊂**C**, and 0.50 for (**a4**·**b1**)⊂**C**. In addition, **b1**’s partners affected its transient fluorescence properties; see below (Section 12).

Analogous results were obtained with other BODIPY dyes. BODIPY **b2** is known for its ability to J-aggregate in mixtures of water and organic solvents.[16] Due to the electron-withdrawing CF_3 group in the *meso* position, the main absorbance band of **b2** dissolved in MeCN is red-shifted (compared to **b1**) to 548 nm. As reported previously,[2] dimerization of **b2** within **C** and the formation of (**b2**)$_2$⊂**C** induced a blue-shift of the main absorption band to 523 nm due to H-aggregation (analogously to the behavior of **b1** within **C**).

Titrating an aqueous solution of (**b2**)$_2$⊂**C** with an encapsulated anthracene (here, (**a1**)$_2$⊂**C**) resulted in the appearance and growth of a red-shifted band at ~565 nm (Figure S91A). The spectrum obtained upon the addition of 3.2 eq of (**a1**)$_2$⊂**C** (red in Figure S91A) shows little residual absorption at 523 nm, indicating a high tendency of **b2** (compared with, e.g., **b1**; see Figure S90A) to form a heterodimer with **a1**. These findings are in agreement with our NMR results (Figure S73), which similarly showed that the equilibrium in a near-stoichiometric mixture of (**a1**)$_2$⊂**C** and (**b2**)$_2$⊂**C** is shifted heavily in the direction of the heterodimer.

We have recently demonstrated that the controlled decomposition of cage **C** with KCN can be used to turn the H-dimers of **b2** (within (**b2**)$_2$⊂**C**) into its J-aggregates.[2] The partial release of **b2** from the cage can also be induced by adding free **a1** (compare with Figure S87B). Indeed, upon titrating (**b2**)$_2$⊂**C** with **a1** (dissolved in a small volume of MeCN), the formation of J-aggregates of **b2** was observed (see the sharp peak at >600 nm in Figure S91B and the yellow spectrum in Figure S91C). If the resulting solution is treated with 3 eq of free **C**, the expelled **b2** is re-encapsulated, and the >600 nm band disappears (green trace in Figure S91C). Thus, the above addition sequence allows us to obtain solutions with three distinct absorption features using a single dye **b2** by varying its aggregation mode.

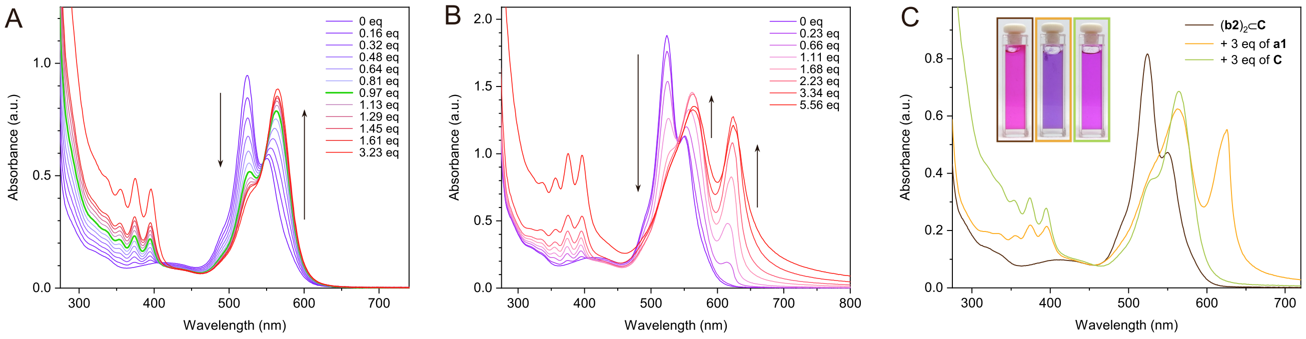

Figure S91. (A) UV-vis absorption spectra recorded during the titration of (**b2**)$_2$⊂**C** with (**a1**)$_2$⊂**C**. (B) UV-vis absorption spectra during the titration of aqueous (**b2**)$_2$⊂**C** with **a1** in MeCN. (C) Photographs of solutions of **b2** in the three different aggregation states and the corresponding UV-vis absorption spectra.

Next, we focused on BODIPY **b3**. Similar to **b1**, **b3** has a sharp absorption peak at 516 nm in MeCN (slightly red-shifted compared to **b1** (λ = 492 nm) due to two additional methyl groups). **b3** is highly emissive, with a sharp emission band centered at 531 nm (in MeCN; excited at 516 nm). Upon dimerization within **C**, we observed a blue-shift of the main absorption band of **b3** to 500 nm and significant emission quenching, with a red-shift of the emission band to 569 nm. These changes give rise to a Stokes shift of 69 nm (compared with 15 nm for **b3** in MeCN), in analogy with **b1**.

Upon treating an aqueous (**b3**)$_2$⊂**C** solution with any of the four (**a**)$_2$⊂**C** complexes, a new absorbance peak centered at 534 nm arose, accompanied by a decrease of the 500 nm band. Figure S92A shows the results of a representative titration of (**b3**)$_2$⊂**C** with (**a4**)$_2$⊂**C**. However, in contrast to titrations involving (**b1**)$_2$⊂**C**, where equilibration was instantaneous, it took many seconds for the spectra to stabilize after each injection of (**a4**)$_2$⊂**C** aliquot. When following the titration by fluorescence spectroscopy, the emission was found to increase dramatically, with a new and intense emission band centered at 549 nm (Figure S92B) (corresponding to a small Stokes shift of 15 nm). We found that the increase in emission intensity at 549 nm was strongly correlated with the increase in absorbance at 534 nm (Figure S92C).

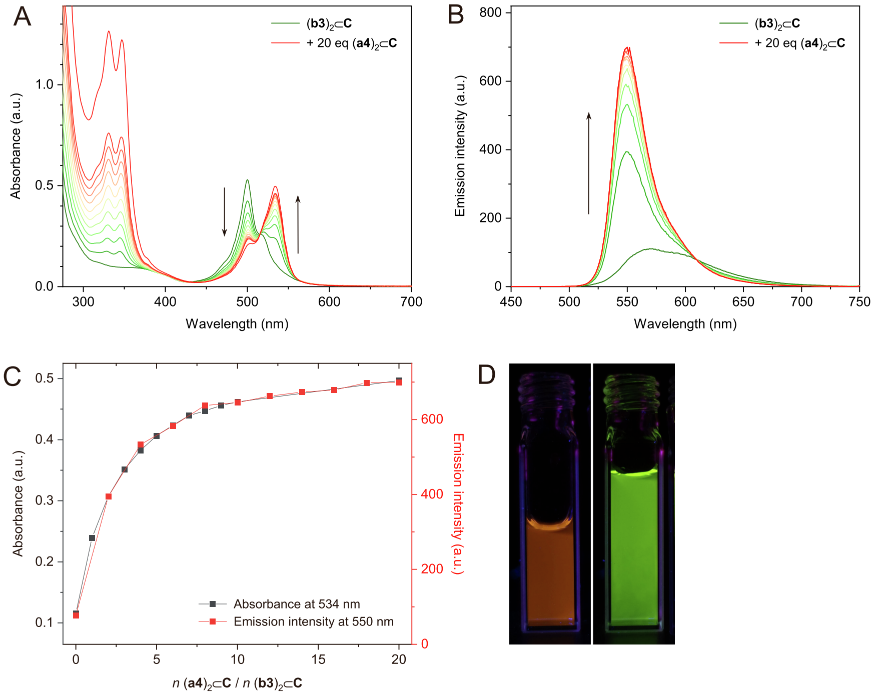

Figure S92. (A) UV-vis absorption spectra accompanying the titration of (**b3**)$_2$⊂**C** with (**a4**)$_2$⊂**C**. After each addition of the titrant, the spectra were allowed to stabilize before the next aliquot of the titrant was added. Here, the time interval between two consecutive additions of the titrant was 2 min. (B) Emission spectra accompanying the titration of (**b3**)$_2$⊂**C** with (**a4**)$_2$⊂**C** (λ_{exc} = 480 nm). (C) Comparison of UV-vis absorption spectroscopy and emission spectroscopy results of the titration of (**b3**)$_2$⊂**C** with (**a4**)$_2$⊂**C**. (D) Photographs of (**b3**)$_2$⊂**C** before (left) and after (right) the addition of a caged anthracene (here, (**a1**)$_2$⊂**C**; i.e., a substantial amount of (**a1**·**b3**)⊂**C**) has formed). The photographs were under the same excitation conditions with λ_{exc} = 365 nm.

To confirm that the observed changes in the optical properties are due to noncovalent interactions, we studied guest release from the cage using an organic solvent. To this end, (**a2**)$_2$⊂**C** and (**b1**)$_2$⊂**C** were first premixed in a 1:4 molar ratio and then titrated with MeCN. Before the titration, UV-vis absorption spectroscopy showed a prominent absorption peak at 511 nm (due to (**a2**·**b1**)⊂**C**), in addition to smaller peaks at 480 nm (due to (**b1**)$_2$⊂**C**), and the characteristic pattern due to anthracene in the near-UV region (purple trace in Figure S93A). The addition of MeCN resulted in a single absorption band in the visible area, centered at 491 nm (red trace in Figure S93A), which can be assigned to free **b1** solvated by MeCN. Similarly, the addition of MeCN induced the release of **a2** (characteristic peaks at 350, 368, and 388 nm; compare with Figure S85A). The same experiment was repeated with other (**a**)$_2$⊂**C** + (**b**)$_2$⊂**C** homodimer combinations (data not shown).

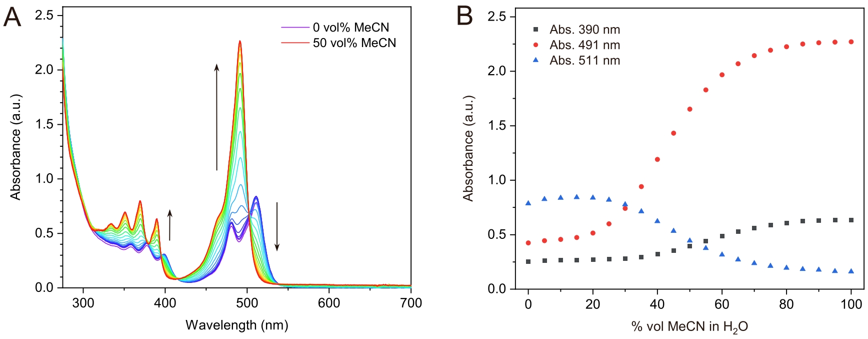

Figure S93. (A) UV-vis absorption spectra (concentration-corrected) recorded during stepwise addition of MeCN to a mixture of (**a2**)$_2$⊂**C** and (**b1**)$_2$⊂**C** to release free **a2** and **b1**. (B) Following the three characteristic wavelengths during the addition of MeCN to premixed (**a2**)$_2$⊂**C** and (**b1**)$_2$⊂**C**.

11. Kinetics of heterodimer formation

Upon mixing homodimeric inclusion complexes of **b1**, **b2**, **b3**, and **b4** with those of **a1**, **a2**, **a3**, and **a4**, heterodimeric complexes (**a**·**b**)⊂**C** formed spontaneously through guest exchange. The kinetics of heterodimer formation depended strongly on the identity of / substitution pattern on guests **a** and **b**.

In a typical experiment, 5 μL of an aqueous solution of (**a2**)$_2$⊂**C** was added to a cuvette containing the same amount of (**b1**)$_2$⊂**C** (in terms of **C**) dissolved in 800 μL of water. The cuvette was quickly inverted twice and placed inside a UV-vis absorption spectrometer, where the initial spectrum (after ~6 s) was recorded. Consecutive spectra were recorded in an automated fashion, one every six seconds. For the **a2**/**b1** combination, we found that the system was equilibrated by the time the first spectrum after mixing was recorded (Figure S94A).

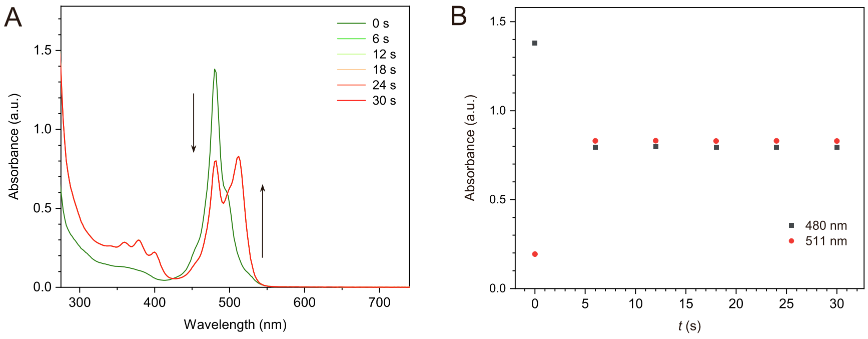

Figure S94. (A) UV-vis absorption spectra recorded at various times after mixing (**a2**)$_2$⊂**C** with (**b1**)$_2$⊂**C** (the spectrum at t = 6 s and the subsequent spectra at 12 s, 18 s, 24 s, and 30 s overlap). (B) Absorbance at 480 nm (due to (**b1**)$_2$⊂**C**) and 511 nm (due to (**a2**·**b1**)⊂**C**) followed by time; replotted from (A).

For the ($\mathbf{a4}$)$_2$⊂**C** + ($\mathbf{b3}$)$_2$⊂**C** mixture, the spectra took significantly longer to stabilize (Figure 3C in the main text). The characteristic peak of homodimer ($\mathbf{b3}$)$_2$⊂**C** at 500 nm gradually decreased, while the 534 nm peak due to heterodimer (**a4·b3**)⊂**C** increased over >2 min (Figure S95).

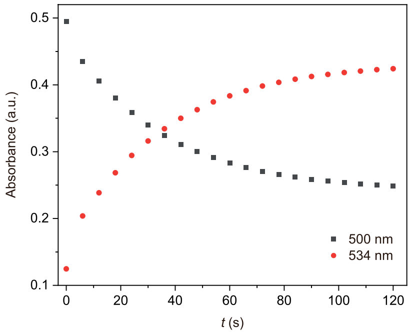

Figure S95. Kinetics of equilibration of ($\mathbf{b3}$)$_2$⊂**C** + ($\mathbf{a4}$)$_2$⊂**C**; absorbance at 500 nm (due to ($\mathbf{b3}$)$_2$⊂**C**) and 534 nm (due to (**a4·b3**)⊂**C**) followed by time.

To determine if the slower formation of (**a4·b3**)⊂**C** (compared with (**a2·b1**)⊂**C**) was caused by replacing **a2** with **a4** or **b1** with **b3**, we replaced each guest individually and thus studied two additional **a**/**b** pairs, namely, ($\mathbf{a4}$)$_2$⊂**C** + ($\mathbf{b1}$)$_2$⊂**C** and ($\mathbf{a2}$)$_2$⊂**C** + ($\mathbf{b3}$)$_2$⊂**C**. As Figure S96A shows, equilibration within the former mixture was completed within the initial 6 s, indicating that replacing **a2** with **a4** did not affect equilibration (within the initial 6 s). However, heterodimer (**a2·b3**)⊂**C** took significantly longer (~2 min) to form; these observations allow us to conclude that increasing the bulkiness on the BODIPY guest by installing two additional methyl groups has a significant effect on guest exchange kinetics. Similar to the case of encapsulated pyrene (Figure 4C in the main text), the anthracene absorption pattern in Figure S96B was the same after 6 s and after 120 s, despite a small fraction of heterodimer within 6 s of reaction time; this observation indicates that the absorption of anthracene **a2** within the ($\mathbf{a2}$)$_2$⊂**C** homodimer and the (**a2·b3**)⊂**C** heterodimer is practically the same. We also compared the formation kinetics of (**a2·b3**)⊂**C** (Figure S97) vs. (**a4·b3**)⊂**C** and found that the former formed slightly faster.

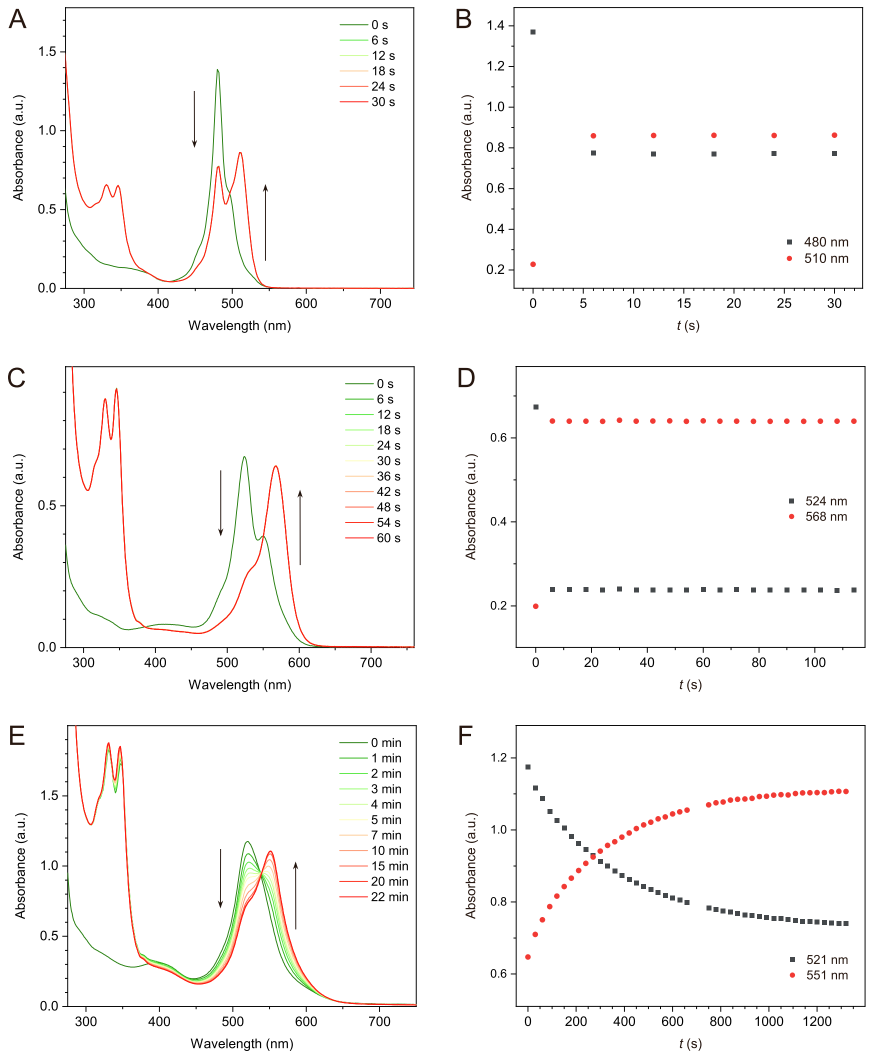

Figure S96. (A) Evolution of UV-vis absorption spectra after addition of (**a4**)$_2$⊂**C** to (**b1**)$_2$⊂**C** (the spectrum at t = 6 s and the subsequent spectra overlap). (B) Absorbance at 480 nm (due to (**b1**)$_2$⊂**C**) and 510 nm (due to (**a4·b1**)⊂**C**) followed by time; replotted from (A). (C) Evolution of UV-vis spectra after addition of (**a4**)$_2$⊂**C** to (**b2**)$_2$⊂**C**. (D) Absorbance at 524 nm (due to (**b2**)$_2$⊂**C**) and 568 nm (due to (**a4·b2**)⊂**C**) followed by time; replotted from (C). (E) Evolution of UV-vis spectra after addition of (**a4**)$_2$⊂**C** to (**b4**)$_2$⊂**C**. (F) Absorbance at 521 nm (due to (**b4**)$_2$⊂**C**) and 551 nm (due to (**a4·b4**)⊂**C**) followed by time; replotted from (E).

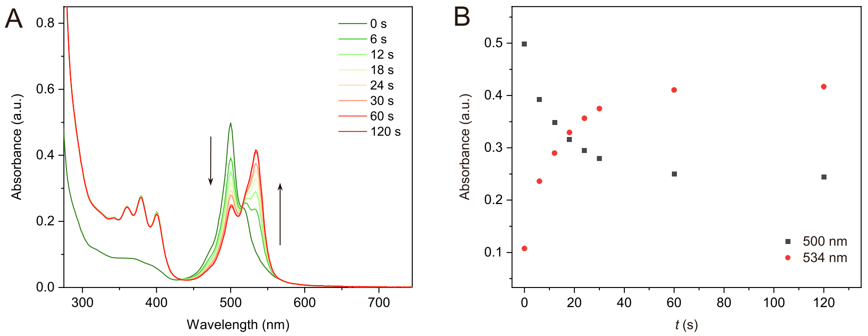

Figure S97. (A) Evolution of UV-vis absorption spectra after addition of (**a2**)$_2$⊂**C** to (**b3**)$_2$⊂**C**. (B) Absorbance at 500 nm (due to (**b3**)$_2$⊂**C**) and 534 nm (due to (**a2·b3**)⊂**C**) followed by time; replotted from (A).

Derivation of the kinetic rate equation

For the reaction,

$$(\mathbf{a})_2\subset\mathbf{C} + (\mathbf{b})_2\subset\mathbf{C} \underset{k_{-1}}{\overset{k_1}{\rightleftharpoons}} (\mathbf{a}\cdot\mathbf{b})\subset\mathbf{C} + (\mathbf{a}\cdot\mathbf{b})\subset\mathbf{C} \qquad (1)$$

let us define the concentrations of species $(\mathbf{a})_2\subset\mathbf{C}$, $(\mathbf{b})_2\subset\mathbf{C}$, and $(\mathbf{a}\cdot\mathbf{b})\subset\mathbf{C}$ as [homo], [homo'], and [hetero], respectively.

The above reaction equation represents the equilibrium between the two homodimers $(\mathbf{a})_2\subset\mathbf{C}$ and $(\mathbf{b})_2\subset\mathbf{C}$ forming two molecules of the heterodimer $(\mathbf{a}\cdot\mathbf{b})_2\subset\mathbf{C}$ as a result of guest exchange. With [hetero] = 2([homo]$_0$ – [homo]) (where [homo]$_0$ is [homo] at $t = 0$), the decay of $(\mathbf{a})_2\subset\mathbf{C}$ upon the addition of 1 eq of $(\mathbf{b})_2\subset\mathbf{C}$ can be written as:

$$\frac{d[\text{homo}]}{dt} = -k_1[\text{homo}]^2 + k_{-1}(2([\text{homo}]_0 - [\text{homo}]))^2 =$$

$$= -k_1[\text{homo}]^2 + 4k_{-1}[\text{homo}]_0^2 - 8k_{-1}[\text{homo}]_0[\text{homo}] + 4k_{-1}[\text{homo}]^2$$

Therefore, $$\frac{d[\text{homo}]}{dt} = [\text{homo}]^2(4k_{-1} - k_1) - 8k_{-1}[\text{homo}]_0[\text{homo}] + 4k_{-1}[\text{homo}]_0^2. \qquad (2)$$

Assuming a statistical distribution of guests among cages, the ratio of the equilibrium concentrations,

[homo]:[homo']:[hetero] = 1:1:2.

Let us define $K_{\text{eq}} = \frac{k_{-1}}{k_1}$; at the same time, $K_{\text{eq}} = \frac{[\text{hetero}]^2}{[\text{homo}][\text{homo'}]}$; therefore, $k_1 = 4k_{-1}$ (the factor of 4 originates from considerations of collision probabilities and probabilities of productive guest exchanges. The number of collisions between $(\mathbf{a})_2\subset\mathbf{C}$ and $(\mathbf{b})_2\subset\mathbf{C}$ (the left-hand side of Equation 1) is twice the number of collisions between $(\mathbf{a}\cdot\mathbf{b})\subset\mathbf{C}$ (the right-hand side of Equation 1), resulting in a factor of 2. Furthermore, all productive collisions between $(\mathbf{a})_2\subset\mathbf{C}$ and $(\mathbf{b})_2\subset\mathbf{C}$ result in $(\mathbf{a}\cdot\mathbf{b})\subset\mathbf{C}$, but only half of all the productive collisions between two copies of $(\mathbf{a}\cdot\mathbf{b})\subset\mathbf{C}$ result in one of the two homodimers, leading to the second factor of 2). Therefore, Equation 2 then gives:

$$\frac{d[\text{homo}]}{dt} = -8k_{-1}[\text{homo}]_0[\text{homo}] + 4k_{-1}[\text{homo}]_0^2$$

$$dt = \frac{d[\text{homo}]}{-8k_{-1}[\text{homo}][\text{homo}]_0 + 4k_{-1}[\text{homo}]_0^2} = \frac{1}{4k_{-1}[\text{homo}]_0} \cdot \frac{d[\text{homo}]}{[\text{homo}]_0 - 2[\text{homo}]}$$

$$-dt = \frac{1}{4k_{-1}[\text{homo}]_0} \cdot \frac{d[\text{homo}]}{2[\text{homo}] - [\text{homo}]_0}.$$

Integrating both sides of the equation,

$$-\int 1dt = \frac{1}{4k_{-1}[\text{homo}]_0} \cdot \int \frac{1}{2[\text{homo}] - [\text{homo}]_0} d[\text{homo}] + c$$

gives:

$$\frac{1}{8k_{-1}[\text{homo}]_0} \cdot \ln(2[\text{homo}] - [\text{homo}]_0) = -t + c$$

and

$$2[\mathrm{homo}]-[\mathrm{homo}]_0 = c'\cdot e^{-8k_{-1}[\mathrm{homo}]_0 t}.$$

Therefore, with the initial conditions $t = 0$ and [homo] = $[\mathrm{homo}]_0$,

$$[\mathrm{homo}] = \frac{1}{2}[\mathrm{homo}]_0 \cdot (1+e^{-8k_{-1}[\mathrm{homo}]_0 t}).$$

As explained above,

$$[\mathrm{hetero}] = 2\cdot([\mathrm{homo}]_0 - [\mathrm{homo}]);$$

therefore, [hetero] can be written as:

$$[\mathrm{hetero}] = 2\cdot([\mathrm{homo}]_0 - \frac{1}{2}[\mathrm{homo}]_0 \cdot (1+e^{-8k_{-1}[\mathrm{homo}]_0 t})).$$

Finally,

$$[\mathrm{hetero}] = [\mathrm{homo}]_0 \cdot (1-e^{-8k_{-1}[\mathrm{homo}]_0 t}),$$

which can be written as,

$$y = \frac{[\mathrm{hetero}]}{[\mathrm{homo}]_0} = 1-e^{-kt}.$$

12. Time-resolved fluorescence spectroscopy of heterodimeric inclusion complexes

The samples were excited by a frequency-tripled Nd:YAG Q-switched laser, pumping an optical parametric oscillator (Ekspla NT342/C/3/UVE) with a pulse duration of 5 ns and a repetition rate of 10 Hz. The fluorescence spectra were collected in the direction orthogonal to the laser incident beam using a 20×0.4 NA objective and spectrally filtered using a longpass filter onto a monochromator (Acton SpectraPro2150i), coupled to a photomultiplier (PMT) tube (Hamamatsu R10699). Transient emission measurements were recorded using a 600 MHz digital oscilloscope (LeCroy Wavesurfer 62Xs). Emission spectra were plotted by integrating over transient emission curve at each wavelength. The excitation pulse energy was measured by a pyroelectric sensor (PE9-C, Ophir Optronics). The spectra of homodimers $(\mathbf{b1})_2{\subset}\mathbf{C}$, $(\mathbf{b2})_2{\subset}\mathbf{C}$, and $(\mathbf{b4})_2{\subset}\mathbf{C}$ were reported before.[2]

For sample preparation, $(\mathbf{b1})_2{\subset}\mathbf{C}$ was mixed with 40 equivalents of $(\mathbf{a1})_2{\subset}\mathbf{C}$, $(\mathbf{a2})_2{\subset}\mathbf{C}$, or $(\mathbf{a4})_2{\subset}\mathbf{C}$ in order to maximize the fraction of **b1** within the heterodimer. We verified that the relatively high concentration of the anthracene guests and the cage did not interfere with the measurements (note that they are transparent in the visible area of the spectrum).

The decay for excited (**a1**·**b1**)⊂**C** and (**a4**·**b1**)⊂**C** is shown in the time-resolved emission spectra in Figure S98; additional results (including decay constants) for these two heterodimers as well as for (**a2**·**b1**)⊂**C** can be found in the main text.

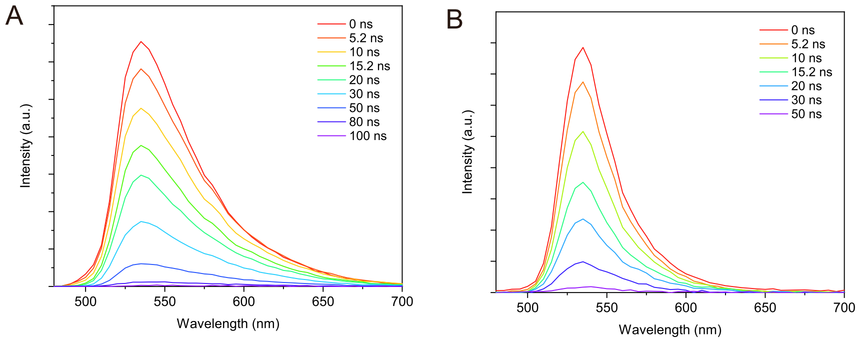

Figure S98. (A) Time-resolved fluorescence spectra of (**a1**·**b1**)⊂**C** in H_2O. (B) Time-resolved fluorescence spectra of (**a4**·**b1**)⊂**C** in H_2O.

13. Photodimerization of encapsulated **a1**–**a4**

For photoirradiation experiments, we used a Prizmatix Mic-LED 365 nm light-emitting diode (LED), a 4-W hand-held 365 nm UV lamp (UVP, LLC; model number UVGL-25), or a pE-4000 LED illumination system (CoolLED; wavelength 385 nm) as the UV light sources. The samples were irradiated in quartz cuvettes at a concentration of 0.031 mM (in terms of cage units) and a fixed distance of 20 cm.

First, the complex ($\mathbf{a2}$)$_2$⊂**C** in aqueous solution was irradiated, and the progress of the reaction was monitored by UV-vis absorption spectroscopy by following the decrease of the characteristic absorbance pattern in the near-UV region (340–420 nm). The reaction was complete within five minutes (see Figure 4B, C in the main text). In contrast, free **a2** dissolved in DCM at the same concentration reacted much slower under the same irradiation conditions; the reaction was not complete after 30 minutes (Figure S99).

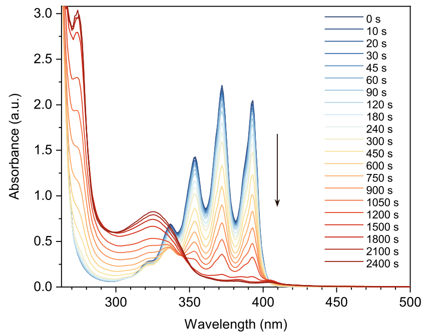

Figure S99. Photodimerization of **a2** in DCM with 365 nm UV light. For ($\mathbf{a2}$)$_2$⊂**C** in an aqueous solution, see Figure 4B, C in the main text.

From this experiment, we concluded that the prearrangement of anthracene units within the cavity of **C** allowed for very fast conversion into the covalent dimer, which we denote **a2a2**. To confirm the identity of the product, we followed the same reaction using ^{1}H NMR, working with a 3 mM solution of ($\mathbf{a2}$)$_2$⊂**C** in D_2O (Figure S100; in this experiment, we used a 385 nm light source, which allowed for a faster reaction under the increased concentration).

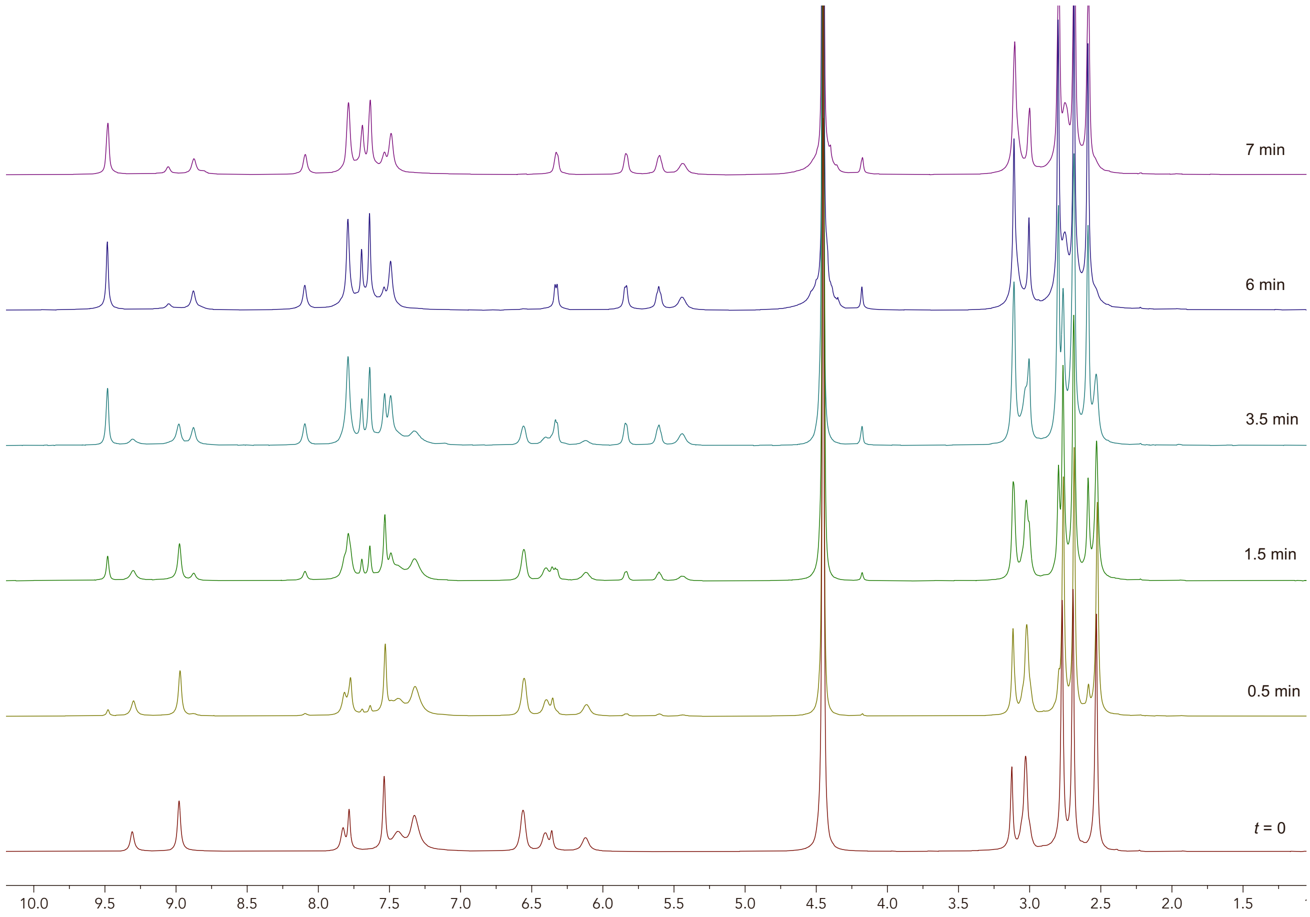

Figure S100. Changes in the ^{1}H NMR spectra of (**a2**)$_2$⊂**C** during the irradiation with 385 nm UV light, indicating transformation into encapsulated dianthracene (**a2a2**)⊂**C** (500 MHz, D_2O, 330 K).

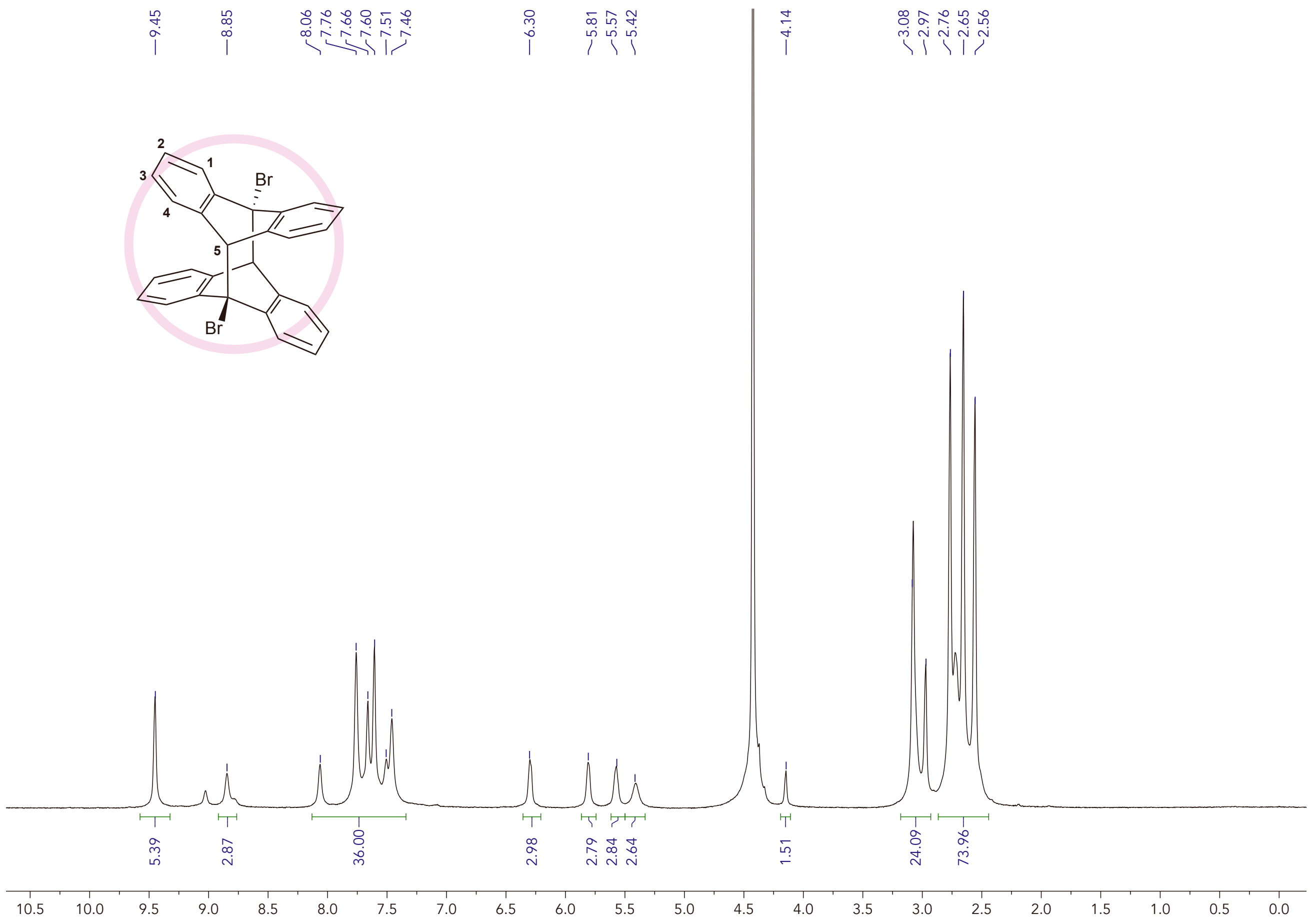

Figure S101. ^{1}H NMR spectrum of the metastable inclusion complex (**a2a2**)⊂**C** (500 MHz, D_2O, 330 K).

Upon irradiation, we observed new peaks that were upfield-shifted compared to encapsulated **a2** (δ = 6.33, 5.83, 5.61, 5.45, and 4.17 ppm; Figures S100, S101). The presence of a single peak at ~4.14 ppm indicates that only one isomer of dianthracene **a2a2** (head-to-tail vs. head-to-head) was formed. The peaks due to encapsulated **a2** (δ = 6.08, 6.32, 6.36, and 6.52 ppm) disappeared during the experiment, indicating a near-quantitative reaction. The dimerization reaction was accompanied by pronounced changes in the chemical shifts of the cage's peaks, suggesting that the cage needs to undergo a large conformational change to accommodate **a2a2**. Despite this change, **C** was unable to complex the dianthracene, as evidenced by gradual precipitation of a colorless solid, which we identified by NMR spectroscopy as **a2a2** (no precipitation was observed in the micromolar concentration range typical of UV-vis absorption measurements).

Based on a COSY experiment (data not shown), the peaks of **a2a2** within **C** could be assigned as follows:

^{1}H NMR (500 MHz, D_2O, 330 K): δ = 6.30 (4H, d, **a2a2$_1$**), 5.81 (4H, d, **a2a2$_4$**), 5.57 (4H, t, **a2a2$_2$**), 5.42 (4H, t, **a2a2$_3$**), 4.14 (2H, s, **a2a2$_5$**).

After 7 min of irradiation, $CDCl_3$ was added to the NMR tube to extract the photoreaction product. NMR analysis of the organic phase revealed the presence of the head-to-tail (*ht*) isomer of **a2a2** (see Figure S102, which compares the obtained spectrum to that of *ht*-**a2a2**[18]). The identity of *ht*-**a2a2** was further confirmed by 2D NMR experiments (data not shown).

Exposing a solution of **a2** in $CDCl_3$ to 365 nm UV light resulted in a mixture of **a2a2** and unreacted **a2**, even at much longer irradiation times (2.5 h in Figure S102C). Like for encapsulated **a2**, a single upfield-shifted (5.36 ppm) peak emerges, indicating the formation of only one isomer of dianthracene (*ht*, as reported before[18]). In addition, the formation of side products was evident, in contrast to the confined system.

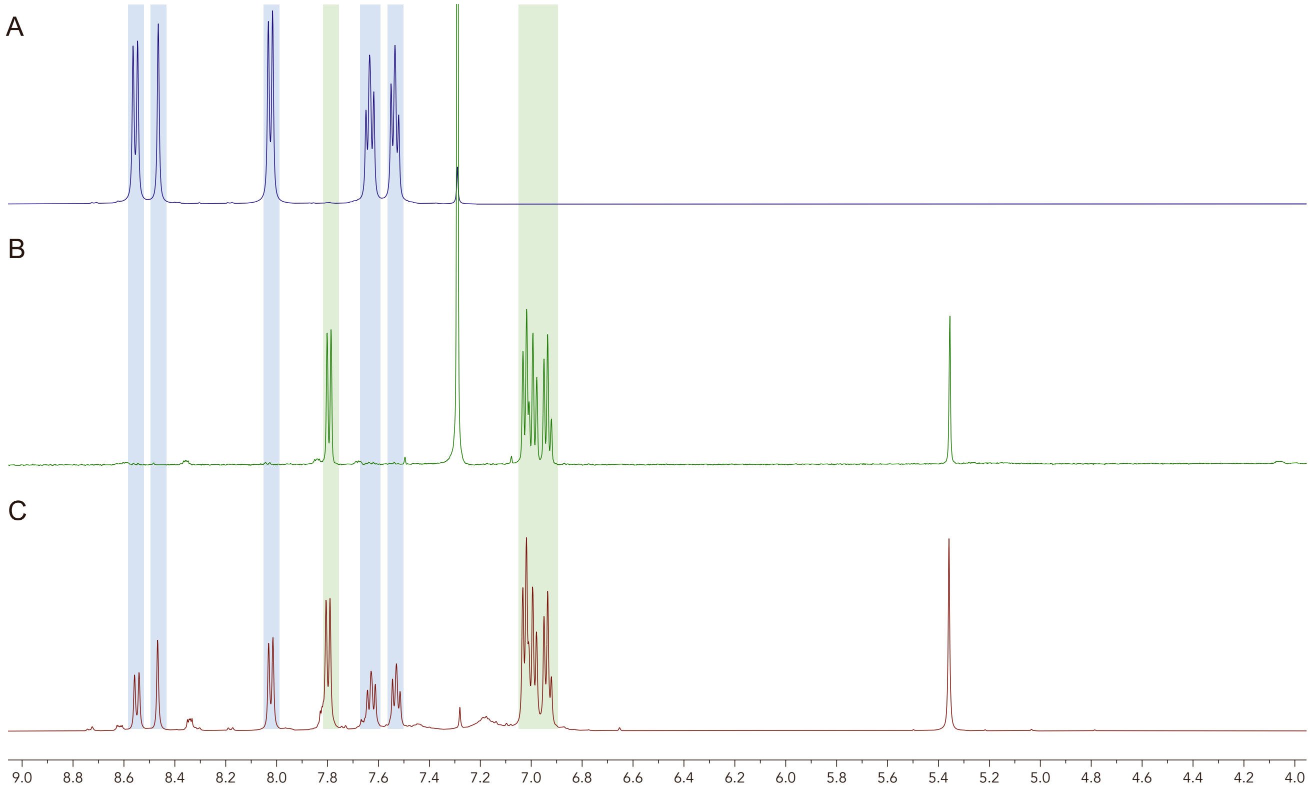

Figure S102. (A) ^{1}H NMR spectrum of **a2** in $CDCl_3$ before irradiation. (B) ^{1}H NMR spectrum of the organic phase ($CDCl_3$) after irradiating an aqueous solution of $(\mathbf{a2})_2\subset\mathbf{C}$ with 385 nm UV light for 7 min followed by extraction. (C) ^{1}H NMR spectrum of **a2** in $CDCl_3$ after irradiation for 160 min with UV light. The spectrum shows the presence of **a2** (blue shades) and **a2a2** (green shades), in addition to side products.

Next, we irradiated a solution of $(\mathbf{a2a2})\subset\mathbf{C}$ (at a concentration in the range of μM) with shorter wavelengths of UV light (254 nm; we used a 4-W hand-held UV lamp; UVP, LLC; model number UVGL-25). The [4+4] photodimerization could partially be reversed, as evidenced by the reappearance of the UV absorption pattern characteristic of anthracene derivatives. However, the initial absorbance of **a2** was not reached even after long irradiation times, indicating the limited reversibility of this reaction. The broadening of the peaks in the UV-vis absorption spectra was observed, suggesting the occurrence of side reactions—such as photolysis of the C–Br bond[18]—which can explain the limited reversibility.

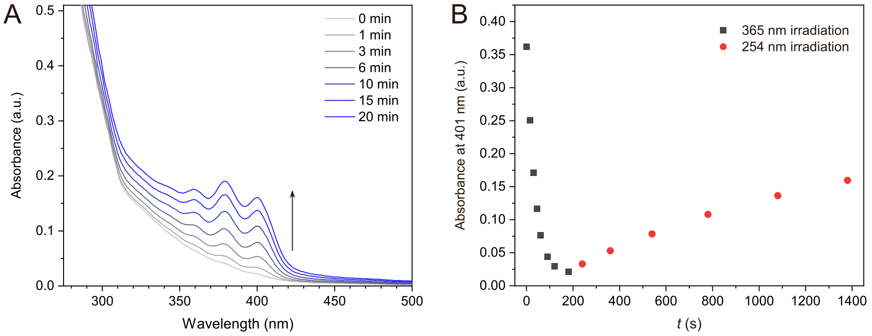

Figure S103. (A) UV-vis absorption spectra accompanying irradiation of an aqueous solution of (**a2a2**)⊂**C** at 254 nm (the initial solution was obtained by exposing (**a2**)$_2$⊂**C** to 365 nm light for 3 min). (B) Following the reversible photodimerization of **a2** within **C** upon irradiation with UV light at 365 nm, followed by 254 nm.

Next, we studied the photoresponsiveness of (**a1**)$_2$⊂**C** by exposing its aqueous solution to 365 nm UV light, analogously to (**a2**)$_2$⊂**C** (see above). However, hardly any changes in the 340–420 nm absorption pattern occurred (Figure S104A), indicating no dimerization of **a1** within **C** (in contrast to (**a2**)$_2$⊂**C**). This result is particularly surprising given that **a1** dissolved in DCM photodimerized much faster than **a2** under the same conditions (see Figure 4C and E in the main text). Anthracene **a3** within **C** photodimerized efficiently (Figure 4G), albeit slower than **a2**.

We also subjected an aqueous solution of (**a4**)$_2$⊂**C** to photoirradiation; although free **a4** does not undergo a photoreaction under 365 nm light, various unexpected [2+2] and [2+4] photodimerization reactions were previously reported in confined systems.[19] However, no indication of a reaction was found (Figure S104B).

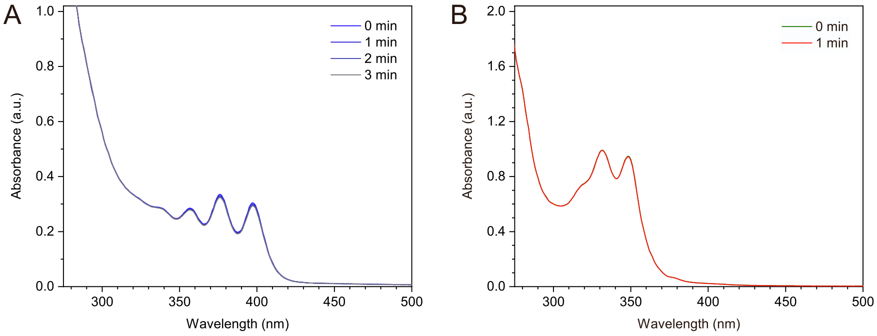

Figure S104. (A) UV-vis absorption spectra accompanying photoirradiation of an aqueous solution of (**a1**)$_2$⊂**C** with 365 nm UV light. (B) UV-vis absorption spectra accompanying photoirradiation of an aqueous solution of (**a4**)$_2$⊂**C** with 365 nm UV light.

14. Photoresponsiveness of heterodimeric inclusion complexes

As shown in Section 13, exposing homodimeric complexes (**a2**)$_2$⊂**C** and (**a3**)$_2$⊂**C** to 365 nm light converts them rapidly to the corresponding dianthracenes (denoted as (**a2a2**)⊂**C** and (**a3a3**)⊂**C**, respectively) as a result of a [4+4] photoreaction. Given the fast guest exchange between inclusion complexes of cage **C**, we hypothesized that the [4+4] reaction could also proceed rapidly in a dynamic mixture of several different complexes. To this end, we mixed aqueous solutions of (**a2**)$_2$⊂**C** (4 eq) and (**b1**)$_2$⊂**C** (1 eq) (equivalents in terms of the cage) to afford a mixture of (**a2**)$_2$⊂**C**, (**b1**)$_2$⊂**C**, and (**a2**·**b1**)⊂**C**. Exposing this mixture to 365 nm light led to a decrease of the characteristic anthracene absorbance pattern in the near UV area (340–420 nm; see Figure 5B in the main text), confirming dianthracene formation. Notably, the photoreaction proceeded somewhat slower than for pure (**a2**)$_2$⊂**C**, which can be explained by the presence of other species—(**b1**)$_2$⊂**C** and (**a2**·**b1**)⊂**C**—absorbing in the near-UV region and lowering the overall quantum efficiency of the [4+4] reaction.

In addition, exposure to UV light induced large changes in the visible part of the spectrum (where the reactive species, **a2**, does not absorb). In particular, the intensity of the heterodimer's band at 511 nm decreased at the expense of the 480 nm band, indicative of the formation of homodimer (**b1**)$_2$⊂**C** (Figure S105A). After 8 min of irradiation, the absorption spectrum was almost indistinguishable from that of (**b1**)$_2$⊂**C** (Figure S105B), indicating that the equilibrium between the homodimers and the heterodimer was strongly shifted to favor (**b1**)$_2$⊂**C** and disfavor (**a2**·**b1**)⊂**C** (Figure 5B).

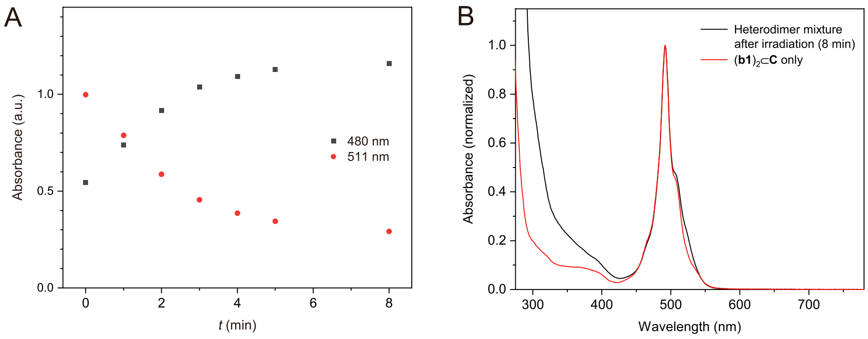

Figure S105. (A) Changes in absorbance at 480 nm (due to (**b1**)$_2$⊂**C**) and 511 nm (due to (**a2**·**b1**)⊂**C**) during irradiation with 365 nm UV light over time; for the spectra, see Figure 5B in the main text. (B) Comparison of the UV-vis absorption spectra of aqueous (**b1**)$_2$⊂**C** (red) and a mixture of (**a2**)$_2$⊂**C**, (**b1**)$_2$⊂**C**, and (**b1**)$_2$⊂**C** after irradiation for 8 min (black).

The reaction was also followed by fluorescence spectroscopy, which showed a substantial decrease in emission during the experiment (Figure S106A). Fluorescence quenching could also be appreciated by the naked eye (Figure S106B).

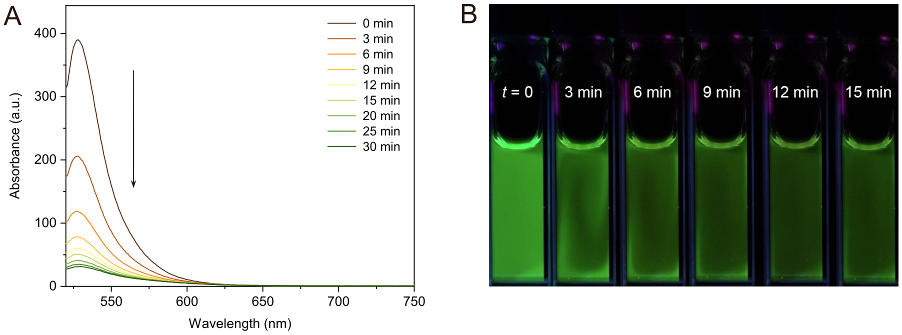

Figure S106. (A) Changes in the fluorescence spectra of a mixture of (**a2**)$_2$⊂**C**, (**b1**)$_2$⊂**C**, and (**b1**)$_2$⊂**C** upon irradiation with 365 nm UV light. (B) Photographs of a mixture of (**a2**)$_2$⊂**C**, (**b1**)$_2$⊂**C**, and (**b1**)$_2$⊂**C** taken at various times during UV irradiation (note that a UV light source different than in the previous experiment (Figure S105) was applied). All the photographs were under the same excitation conditions with λ_{exc} = 365 nm.

Replacing (**a2**)$_2$⊂**C** with either (**a1**)$_2$⊂**C** or (**a4**)$_2$⊂**C** rendered the system non-photoresponsive (as expected from the lack of photoresponsiveness of both (**a1**)$_2$⊂**C** and (**a4**)$_2$⊂**C**) – see Figure S107A and B, respectively. If, however, (**b1**)$_2$⊂**C** was replaced by any other BODIPY homodimer—(**b2**)$_2$⊂**C**, (**b3**)$_2$⊂**C**, or (**b4**)$_2$⊂**C**—the system retained its photoresponsiveness (Figure S107C and D).

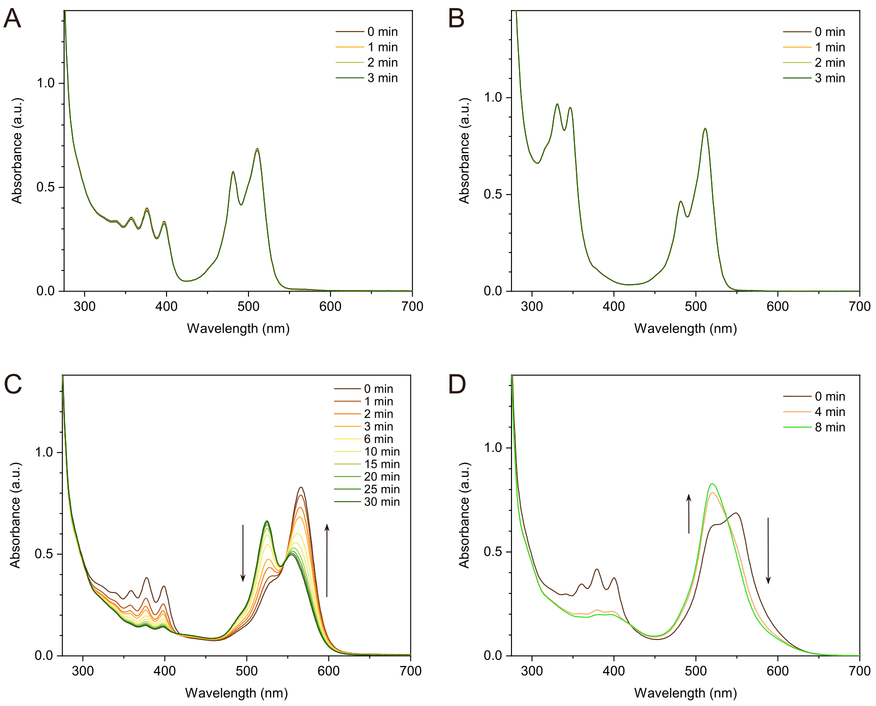

Figure S107. (A) No changes in the UV-vis absorption spectra were observed upon exposing an (**a1·b1**)⊂**C** + (**a1**)$_2$⊂**C** + (**b1**)$_2$⊂**C** mixture to 365 nm light. (B) Similarly, no changes were observed when an (**a4·b1**)⊂**C** + (**a4**)$_2$⊂**C** and (**b1**)$_2$⊂**C** mixture was exposed to 365 nm light. (C) Changes in the UV-vis spectra of an (**a2·b2**)⊂**C** + (**a2**)$_2$⊂**C** and (**b2**)$_2$⊂**C** mixture exposed to 365 nm light; a fast reaction was observed. (D) Changes in the UV-vis spectra of an (**a2·b4**)⊂**C** + (**a2**)$_2$⊂**C** and (**b4**)$_2$⊂**C** mixture exposed to 365 nm light; a fast reaction was observed.

Exposing dianthracenes to 254 nm UV light induces the dedimerization reaction to afford the starting anthracene. Irradiating the (**a2a2**)⊂**C** + (**b1**)$_2$⊂**C** mixture at 254 nm induced the reappearance of the characteristic anthracene pattern in the near-UV region (Figure S108A). At the same time, the ratio of homodimer (**b1**)$_2$⊂**C** to heterodimer (**a2**·**b1**)⊂**C** changed in favor of the latter (Figure S108A). However, due to the limited reversibility of cycloreversion (Section 13), the system did not reach its initial state fully (see Figure S108B).

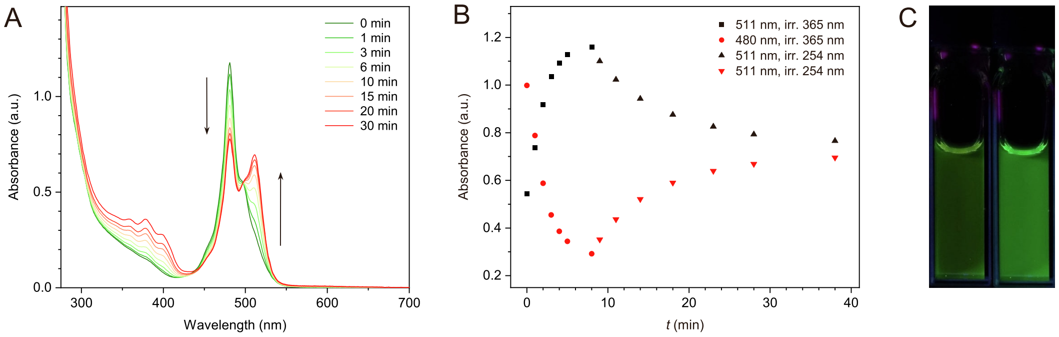

Figure S108. (A) Changes in the UV-vis absorption spectra of an (**a2**·**b1**)⊂**C** + (**a2**)$_2$⊂**C** + (**b1**)$_2$⊂**C** mixture that had been irradiated with 365 nm light (8 min) during irradiation with 254 nm light. (B) Reversible changes in the absorbance at 480 nm (due to (**b1**)$_2$⊂**C**) and 511 nm (due to (**a2**·**b1**)⊂**C**) during irradiation with 365 nm and then 254 nm light. (C) Photographs of a (**a2**·**b1**)⊂**C** + (**a2**)$_2$⊂**C** + (**b1**)$_2$⊂**C** mixture that had been irradiated with 365 nm light for 15 min before (left) and after (right) exposure to 254 nm light for 3 min. The photographs were under the same excitation conditions with λ_{exc} = 365 nm.

15. Preparation of photoresponsive gels based on heterodimeric inclusion complexes

Agarose (1 g) was added to an Erlenmeyer flask containing 50 mL of distilled water. The mixture was heated in an oil bath; once water started to boil, heating was continued for an additional 3 min. Then, the flask was removed from the oil bath, and the resulting colorless, homogeneous solution was poured (while hot) between two glass slides separated by 1 mm spacers. After having been cooled to room temperature, the solidified agarose gel was removed from the template and cut into rectangular pieces (25×35×1 mm or 10×10×1 mm) using a cutter knife. The gels were transferred to a Petri dish or a vial containing aqueous solutions of inclusion complexes for soaking (60 min).

II. Supplemental references

1. Mizuno, K., Tamiya, Y., and Mekata, M. (2004). External double reference method to study concentration and temperature dependences of chemical shifts determined on a unified scale. Pure Appl. Chem. *76*, 105–114.
2. Gemen, J., Ahrens, J., Shimon, L. J. W., and Klajn, R. (2020). Modulating the optical properties of BODIPY dyes by noncovalent dimerization within a flexible coordination cage. J. Am. Chem. Soc. *142*, 17721–17729.
3. Nepomnyashchii, A. B., Bröring, M., Ahrens, J., and Bard, A. J. (2011). Synthesis, photophysical, electrochemical, and electrogenerated chemiluminescence studies. Multiple sequential electron transfers in BODIPY monomers, dimers, trimers, and polymer. J. Am. Chem. Soc. *133*, 8633–8645.

4. Dolomanov, O. V., Bourhis, L. J., Gildea, R. J., Howard, J. A. K., and Puschmann, H. (2009). *OLEX2*: a complete structure solution, refinement and analysis program. J. Appl. Crystallogr. *42*, 339–341.
5. Sheldrick, G. M. (2008). A short history of *SHELX*. Acta Crystallogr. Sect. A *64*, 112–122.
6. Spek, A. L. (2015). *PLATON* SQUEEZE: a tool for the calculation of the disordered solvent contribution to the calculated structure factors. Acta Crystallogr. Sect. C *71*, 9–18.
7. Samanta, D., Galaktionova, D., Gemen, J., Shimon, L. J. W., Diskin-Posner, Y., Avram, L., Král, P., and Klajn, R. (2018). Reversible chromism of spiropyran in the cavity of a flexible coordination cage. Nat. Commun. *9*, 641.
8. Turowska-Tyrk, I., and Trzop, E. (2003). Monitoring structural transformations in crystals. 6. The [4 + 4] photodimerization of 9-methyl-anthracene. Acta Crystallogr. B *59*, 779–786.
9. Mondal, R., Nesterov, E. E., and Fronczek, F. R. (2012). CCDC 864332: Experimental crystal structure determination. DOI: 10.5517/ccy0dp0.
10. Sweeting, L. M., and Rheingold, A. L. (1988). Crystal structure and triboluminescence. 1. 9-Anthryl carbinols. J. Phys. Chem. *92*, 5648–5655.
11. Camerman, A., and Trotter, J. (1965). The crystal and molecular structure of pyrene. Acta. Cryst. *18*, 636–643.
12. Becke, A. D. (1993). Density-functional thermochemistry. III. The role of exact exchange. J. Chem. Phys. *98*, 5648–5652.
13. Lee, C., Yang, W., and Parr, R. G. (1988). Development of the Colle-Salvetti correlation-energy formula into a functional of the electron density. Phys. Rev. B *37*, 785–789.
14. Frisch, M. J., Trucks, G. W., Schlegel, H. B., Scuseria, G. E., Robb, M. A., Cheeseman, J. R., Scalmani, G., Barone, V., Petersson, G. A., Nakatsuji, H., Li, X., Caricato, M., Marenich, A. V., Bloino, J., Janesko, B. G., Gomperts, R., Mennucci, B., Hratchian, H. P., Ortiz, J. V., Izmaylov, A. F., Sonnenberg, J. L., Williams-Young, D., Ding, F., Lipparini, F., Egidi, F., Goings, J., Peng, B., Petrone, A., Henderson, T., Ranasinghe, D., Zakrzewski, V. G., Gao, J., Rega, N., Zheng, G., Liang, W., Hada, M., Ehara, M., Toyota, K., Fukuda, R., Hasegawa, J., Ishida, M., Nakajima, T., Honda, Y., Kitao, O., Nakai, H., Vreven, T., Throssell, K., Montgomery, J. A., Jr., Peralta, J. E., Ogliaro, F., Bearpark, M. J., Heyd, J. J., Brothers, E. N., Kudin, K. N., Staroverov, V. N., Keith, T. A., Kobayashi, R., Normand, J., Raghavachari, K., Rendell, A. P., Burant, J. C., Iyengar, S. S., Tomasi, J., Cossi, M., Millam, J. M., Klene, M., Adamo, C., Cammi, R., Ochterski, J. W., Martin, R. L., Morokuma, K., Farkas, O., Foresman, J. B., and Fox, D. J. Gaussian 16, Revision C.01. Gaussian, Inc., Wallingford CT, 2016.
15. Jones, R. N. (1947). The ultraviolet absorption spectra of anthracene derivatives. Chem. Rev. *41*, 353–371.
16. Choi, S., Bouffard, J., and Kim, Y. (2014). Aggregation-induced emission enhancement of a *meso*-trifluoromethyl BODIPY *via* J-aggregation. Chem. Sci. *5*, 751–755.
17. Yamashina, M., Sartin, M. M., Sei, Y., Akita, M., Takeuchi, S., Tahara, T., and Yoshizawa, M. (2015). Preparation of highly fluorescent host–guest complexes with tunable color upon encapsulation. J. Am. Chem. Soc. *137*, 9266–9269.
18. Fulara, J., and Latowski, T. (1981). Photochemical reactions of bromoanthracenes with N,N-dimethylamine in solution. Z. Naturforsch. B *36*, 846–851.
19. Nishioka, Y., Yamaguchi, T., Yoshizawa, M., and Fujita, M. (2007). Unusual [2+4] and [2+2] cycloadditions of arenes in the confined cavity of self-assembled cages. J. Am. Chem. Soc. *129*, 7000–7001.
